# Thinning Partially Mitigates the Impact of Atlantic Forest Replacement by Pine Monocultures on the Soil Microbiome

**DOI:** 10.3389/fmicb.2020.01491

**Published:** 2020-07-03

**Authors:** Carolina Paola Trentini, Paula Inés Campanello, Mariana Villagra, Julian Ferreras, Martin Hartmann

**Affiliations:** ^1^Laboratorio de Ecología Forestal y Ecofisiología, Instituto de Biología Subtropical, CONICET-UNaM, Puerto Iguazú, Misiones, Argentina; ^2^Centro de Estudios Ambientales Integrados, Facultad de Ingeniería, Universidad Nacional de la Patagonia San Juan Bosco, CONICET, Esquel, Argentina; ^3^Grupo de Investigación en Genética Aplicada, Instituto de Biología Subtropical, CONICET-UNaM, Posadas, Misiones, Argentina; ^4^Sustainable Agroecosystems, Department of Environmental Systems Science, Institute of Agricultural Sciences, ETH Zürich, Zurich, Switzerland

**Keywords:** atlantic forest, *Pinus taeda*, thinning practice, plant understory, biodiversity, soil microbiome

## Abstract

Forest replacement by exotic plantations drive important changes at the level of the overstory, understory and forest floor. In the Atlantic Forest of northern Argentina, large areas have been replaced by loblolly pine (*Pinus taeda* L.) monocultures. Plant and litter transformation, together with harvesting operations, change microclimatic conditions and edaphic properties. Management practices such as thinning promote the development of native understory vegetation and could counterbalance negative effects of forest replacement on soil. Here, the effects of pine plantations and thinning on physical, chemical and microbiological soil properties were assessed. Bacterial, archaeal, and fungal community structure were analyzed using a metabarcoding approach targeting ribosomal markers. Forest replacement and, to a lesser extent, thinning practices in the pine plantations induced significant changes in soil physico-chemical properties and associated shifts in bacterial and fungal communities. Most measured physical and chemical properties were altered due to forest replacement, but a few of these properties reached values similar to natural forests under the thinning operation. Fungal alpha diversity decreased in pine plantations, whereas bacterial alpha diversity tended to increase but with little statistical support. Shifts in community composition were observed for both fungal and bacterial domains, and were mostly related to changes in plant understory composition, soil carbon, organic matter, water content, pH and bulk density. Among several other changes, highly abundant phyla such as Proteobacteria (driven by many genera) and Mortierellomycota (mainly driven by *Mortierella*) decreased in relative abundance in the plantations, whereas Acidobacteria (mainly driven by *Acidothermus* and *Candidatus Koribacter*) and Basidiomycota (mainly driven by the ectomycorrhiza *Russula*) showed the opposite response. Taken together, these results provide insights into the effects of forest replacement on belowground properties and elucidate the potentially beneficial effect of thinning practices in intensive plantation systems through promoting the understory development. Although thinning did not entirely counterbalance the effects of forest replacement on physical, chemical and biological soil properties, the strategy helped mitigating the effects and might promote resilience of these properties by the end of the rotation cycle, if subsequent management practices compatible with the development of a native understory vegetation are applied.

## Introduction

The economy-driven replacement of natural forests with exotic monospecific plantations has visible effects on plant structure and aboveground biodiversity, but the effects on belowground biodiversity and the ecosystem functions it provides are less clear. Changes in vegetation can alter soil microbial community diversity and function directly through new plant-microbe interactions or indirectly through changes in physico-chemical soil properties and litter layer composition (Zak et al., 2003; Moukoumi et al., 2006; Broeckling et al., 2008). In forest ecosystems, large trees are expected to be the main regulators of soil microbial communities as they contribute the most to litter biomass and rhizodeposition (Prescott and Grayston, 2013; Urbanová et al., 2015) and establish frequent and far-reaching root-microbe interactions throughout the soil (Morris et al., 2009; Beiler et al., 2015; Kutszegi et al., 2015). However, studies on plant removal have shown that the understory vegetation also has a significant effect on the soil microbial community (Wu et al., 2011; Zhao et al., 2013).

Forest management strategies in pine plantations such as thinning promote the development of a native understory vegetation due to the increase in available solar radiation (e.g., Cheng et al., 2017; Trentini et al., 2017; Dang et al., 2018). At the same time, thinning decreases water availability in the forest floor and increases soil compaction (e.g., Picchio et al., 2012; Trentini et al., 2017). Thus, thinning may affect microbial communities due to changes in both understory vegetation and soil characteristics, which in turn influences soil metabolic activity (Boerner et al., 2008; Chen et al., 2015; Dang et al., 2018). For example, soil compaction can favor organisms adapted to anoxic conditions, ultimately changing nutrient cycling and greenhouse gas fluxes in the system (Hartmann et al., 2014). Reducing soil moisture can decrease microbial activity and change the trophic composition of both bacterial and fungal communities, with consequences in carbon mineralization in the system (Hartmann et al., 2017). Therefore, the direct and indirect effects of thinning will impact different groups of microorganisms and potentially alter associated soil processes such as nitrogen fixation (e.g., diazotrophic bacteria), nutrient uptake (mycorrhizae), or carbon utilization (fungal and bacterial saprotrophs) (Dang et al., 2018).

The Atlantic Forest (AF) of South America is one of the most important biodiversity “hotspots” in the world (Myers et al., 2000). In northern Argentina, large areas of these subtropical forests have been replaced by high-yield plantations of loblolly pine (*Pinus taeda* L.) (Izquierdo et al., 2008). The replacement of the AF by pine monocultures has important changes at the soil level due to harvesting, tree planting and management practices. All these operations involve a replacement of the rhizosphere, and litter transformation from a heterogeneous broad-leaf to homogeneous lignin-rich needle litter (Zaninovich et al., 2016; Trentini et al., 2017). This, in turn, can change physical, chemical and biological soil characteristics. A study about the replacement of the AF by slash-and-burn agricultural systems in Brazil has shown a strong impact on soil structure, nutrients, and microbial community composition (Aboim et al., 2008); however, the effects of replacement by pine plantations and their management have been less studied. A global meta-analysis on pine afforestation in grasslands and shrublands showed a decrease in soil pH, carbon and nitrogen, as well as changes in various soil cations (Berthrong et al., 2009). Pine litter and root exudates generate a new environment for microbial community development by changing soil conditions like pH that are known to impact microbial community composition (Fierer and Jackson, 2006; Kutszegi et al., 2015).

Despite the importance of the AF, there is still a significant lack of knowledge of soil microbial diversity and functions in these ecosystems. Most of the microbial studies in the AF focused on bacterial diversity (Bruce et al., 2010, 2012; Faoro et al., 2010, 2013; Catão et al., 2014; Pacchioni et al., 2014). Andrade et al. (2000) were the first to characterize the AF mycorrhizal status. They found that all collected plants harbored arbuscular (AM) or vesicular (VM) but not ecto-mycorrhizal (ECM) associations. So far, most explorations of the AM in the AF were done using techniques such as microscopy, spore counting, root observations, or trap plants (Bonfim et al., 2016; Duarte et al., 2019; Morales-Londoño et al., 2019). The first study using molecular genetic tools to describe AF wood decay fungi was done in 2017 (Vaz et al., 2017). To date, no characterization of both bacterial and fungal soil communities, and the potential impacts of AF conversion and land use regimes in pine plantations has been performed.

The objectives of this study were (1) to evaluate the changes in physico-chemical soil properties and microbial community structure as a result of natural forest replacement by pine plantations, (2) to assess the potential effect of the understory development after thinning to counterbalance the effects of native tree species replacement, and (3) to identify the main environmental factors that drive microbial community composition in these systems. Specifically, we studied changes in soil physical and chemical properties as well as diversity and composition of soil bacterial, archaeal and fungal communities. We hypothesized that the replacement of the subtropical forest by pine plantations will have consequences for soil physical properties through management practices that increase compaction and decrease water retention. Changes in the organic matter supply through alterations in carbon input through root exudation and litter fall, as well as the continuous resources uptake by the harvested plantations will decrease soil nutrient content (P, N, cations). Shifts in vegetation and litter composition will alter microbial community composition and reduce both fungal and bacterial diversity. Thinning treatments that promote stand diversification through the development of a native understory vegetation will at least partially compensate for nutrient losses by generating labile organic matter and providing new niches for microbial colonization when compared to no-thinning stands.

## Materials and Methods

### Study Area

**FIGURE 1:**
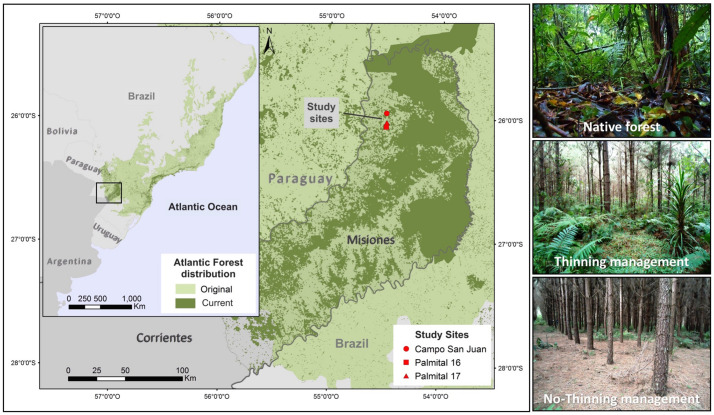
Location of the study area in Northeastern Argentina. The surface shown in dark green corresponds to the remaining natural forest area and in light green the original cover. The photos illustrate the different management treatments in each stand of *Pinus taeda* (Campo San Juan, Palmital 16, Palmital 17) and the remaining Atlantic Forest strips adjacent to each stand.

The study was conducted in plantations of *Pinus taeda* L. in the northern area of Misiones, Argentina (Figure 1). The average rainfall in this area is 2000 mm and is distributed throughout the year. The mean temperature is 21°C and winter frost is rare. Soils derived from basaltic rocks with high concentrations of Fe, Al and Si which include Alfisols, Molisols and Inceptisols (Soil Survey Staff, 2014). Figure 1 shows the contrast between the currently conserved AF area and its original surface. In Argentina there is an important remnant of natural forest when compared with Brazil and Paraguay, where most of its original area was converted to other land uses.

### Experimental Design

A completely randomized block design was established to test the hypotheses in three geographically separated pine plantations (blocks): “Palmital 16” (P16; 26°09′59″ S, 54°26′27″ W), “Palmital 17” (P17; 26°09′43″ S, 54°26′35″ W) and “Campo San Juan” (SJ; 26°05′40″ S, 54°26′48″ W) separated from each other by one and five km (Figure 1). Pine plantations were carefully selected to have the same stand age and current management regimes, while being next to natural forests that serve as a baseline to put the magnitude and direction of the changes induced by forest conversion into context. All plantations belong to the first rotation of the same company and were planted in 2006 with an initial density of 1600 trees per ha. The plantations differ slightly in their land history use (see Trentini et al., 2017). Pine plantations in Misiones are usually managed in two different ways, either for saw timber production where stands receive strong thinning (50%), or for pulp production where no thinning is performed and stands are clear-cut after 12 years. Management regimes for timber production include three successive thinning operations at around 6, 12, and 15 years. More details on the thinning procedures have been reported previously (Trentini et al., 2017). In each block, two plots of 65 × 65 m were installed, one in which 50% thinning was carried out and one control (no-thinning). The block was completed with three transects of 10 × 100 m that were located in the strip of natural forest neighboring each plantation, maintaining the same distance to each plot. In turn, each plot was divided into 15 × 15 m sub-plots in order to avoid the hauling roads.

### Physico-Chemical Analyses

Soil physico-chemical parameters were analyzed from pooled soil samples (300 g) consisting of the first 10 cm taken at three randomly selected locations per plot in February 2016 (10 years after planting and 3 years after the first thinning). Soil texture was measured by Robinson’s pipette method (Robinson, 1922), pH by potentiometric method in soil:water solution (1:2.5), organic carbon by the dry combustion method (Nelson and Sommers, 1996) (Laboratory Carbon Analyzer Organic Inorganic determination – LECO Cr-12), organic matter by the Loss On Ignition method – LOI (Schulte and Hopkins, 1996), total nitrogen by the Kjeldahl method (Bremner, 1960), extractable phosphorus by the Bray–Kurtz method (Bray and Kurtz, 1945), and available and exchangeable cations (Ca, K, Mg) by Inductively Coupled Plasma Mass Spectrometry (ICP-MS) with ammonium acetate and the exchangeable procedure with sodium acetate (Soltanpour et al., 1996). Soil water content was estimated for the first 5 cm soil depth in March 2015 after a no-rain period of 20 days and bulk density was estimated for the first 10 cm soil depth in October 2014. Soil samples were oven-dried at 105°C to a constant weight. In order to determine pine litter biomass and their water content, three samples (0.09 m^2^ each) were taken per treatment plot in June 2015, these samples were weighed and then oven-dried at 70°C to a constant weight. Soil and litter water content was calculated as the difference between the weight of moist and dry samples, divided by the weight of the moist sample.

### Understory Vegetation

Understory data were published previously (Trentini et al., 2017). In brief, plant coverage, richness and diversity was characterized using the interception method (Mueller-Dombois and Ellenberg, 1974) across 108 points systematically located within each plot and along the three transects located in the natural forest strips next to the plantations in each block (see Trentini et al., 2017). The interception method was done with a vertical stick in those points counting and identifying all the species that touch it, up to approximately two meters high (maximum height of the undergrowth). Species that were intercepted only once in the entire sample were not considered. The measurements were made in November 2013, 1 year after the thinning treatments, once the understory species had reached equilibrium in terms of recruitment and development (Trentini et al., 2017).

### Microbiological Analyses

#### DNA Extraction, Target Amplification and Sequencing

For microbiological analyses, total genomic DNA was extracted from 0.25 g fresh soil samples by using the Powersoil DNA Isolation kit (MOBIO Laboratories, Inc., Carlsbad, CA, United States) according to the manufacturer’s protocol. The final DNA sample for each plot consisted of a pool of five DNA subsamples individually extracted from randomly selected locations within each plot. To improve the quality and concentration of the DNA, the final DNA pools were treated with phenol:chloroform: isoamyl alcohol (25:24:1) and precipitated with ethanol. Initial quality controls were performed by using a micro-volume spectrophotometer (MaestroNano, Maestrogen Inc., Taiwan), and then DNA was stored at −80°C until further analysis.

DNA extracts were sent to Indear SA (Rosario, Argentina) for prokaryotic (bacterial and archaeal) and to Macrogen Inc., (Seoul, South Korean) for eukaryotic (fungal and some groups of protists and green algae) marker amplification and paired-end sequencing on the Illumina MiSeq platform using the v3 chemistry. PCR for bacteria and archaea targeted the V3-V4 region of the small-subunit (16S) rRNA gene using the primers 341F (CCTACGGGNGGCWGCAG) and 805R (GACTACHVGGGTATCTAATCC) (Herlemann et al., 2011). PCR for eukaryotes targeted the internal transcribed spacer region 2 (ITS2) of the ribosomal operon using primers ITS3 (GCATCGATGAAGAACGCAGC) and ITS4 (TCCTCCGCTTATTGATATGC) (White et al., 1990). In both cases, Nextera adapter sequences were added to the target-specific primers and libraries were generated using a two-step PCR procedure. For the first PCR, 10 and 5 ng of DNA, respectively, were used to amplify the bacterial/archaeal and fungal amplicons. Briefly, DNA was mixed with 0.2 μM of each primer and with 2× KAPA HiFi HotStart ReadyMix for a 1× final concentration in 25 μl reaction volume. PCR was run for 95°C for 3 min, followed by 25 cycles at 95°C for 30 s, 55°C for 30 s, and 72°C for another 30 s, and including a final extension step at 72°C for 5 min. After amplicons were purified using AMPure XP beads (Beckman Coulter) following manufacturer’s recommendations, a second PCR was done using the Nextera Index Kit (Illumina) following supplier’s recommendations. Briefly 5 μl of PCR product was mixed with 5 μl each of index primers 1 and 2, 10 μl of PCR grade water, and 25 μl 2× KAPA HiFi HotStart ReadyMix for a final reaction volume of 50 μl. PCR was performed with same program as described before but running only 8 cycles instead of 25. Libraries were purified using AMPure XP beads, validated on a Bioanalyzer DNA 1000 (Agilent), and pooled in equimolar concentrations before sequencing.

### Sequence Quality Control, Binning and Taxonomic Assignment

Sequence data were processed using a customized pipeline largely based on VSEARCH v.2.8 (Rognes et al., 2016). In brief, paired-end reads were merged using the *fastq_mergepairs* algorithm (Edgar and Flyvbjerg, 2015) implemented in VSEARCH. Merged reads deriving from PhiX were removed by running Bowtie2 (Langmead and Salzberg, 2012) against the PhiX genome. PCR primers were trimmed using Cutadapt (Martin, 2011) allowing for one mismatch. Trimmed reads were quality filtered using the *fastq_filter* function (Edgar and Flyvbjerg, 2015) implemented in VSEARCH allowing for a maximum expected error of one. Sequences were dereplicated using the *derep_fulllength* function in VSEARCH. Amplicon sequence variants (ASVs) were retrieved from the dereplicated dataset using the UNOISE algorithm (Edgar, 2016a) implemented in VSEARCH with an alpha of 2 and minsize of 4. Potentially chimeric ASVs were identified and removed using the UCHIME algorithm (Edgar, 2016b) implemented in VSEARCH. Remaining ASVs were tested for the presence of ribosomal signatures using Metaxa2 (Bengtsson-Palme et al., 2015) and ITSx (Bengtsson-Palme et al., 2013) for the 16S rRNA gene and ITS2 sequences, respectively, and unsupported sequences were discarded. The final ASVs table was obtained by mapping the quality filtered reads of each sample against the verified ASVs using the *usearch_global* algorithm (Edgar, 2010) implemented in VSEARCH. Taxonomic classification of each verified ASV was performed by running the SINTAX algorithm (Edgar, 2016c) implemented in VSEARCH against the SILVA v.128 database (Quast et al., 2013) for the 16S rRNA gene sequences and against the NCBI and UNITE v.7.2 database (Nilsson et al., 2019) for the 5.8S and ITS2 sequences, respectively, using a bootstrap cutoff of 0.8. Non-fungal ITS2 sequences were identified and removed from the ASVs table by classifying the ASVs against a customized ITS2 database featuring all eukaryotic ITS2 sequences deposited in the NCBI nucleotide database (Benson et al., 2018). Finally, 16S rRNA gene ASVs assigned to organelle structures (chloroplasts, mitochondria) were removed from the ASVs table. Raw sequences were deposited in the European Nucleotide Archive under the accession number PRJEB36362.

### Data Analyses

All analyses were performed in the software package R v.3.2.3 (R Core Team, 2014) using various packages as identified below. The plantation stands determined the sample size in all analyses, significance levels of 5% were used but considering the low number of replicates (i.e., 3) *p*-values lower than 0.1 were considered marginally significant and no corrections for multiple testing were performed. Changes in physico-chemical soil properties were assessed using Linear Mixed-Effects Model (LMM) to assess treatment differences setting the block as a random factor using *lme* function in nlme (Laird and Ware, 1982), a constant variance function structure, *varIdent* in nlme was added to the function in the variables that did not fulfill homoscedasticity assumption (Pinheiro and Bates, 2000). ASV count tables were iteratively (1000-fold) subsampled the lowest sequence count prior to calculating α-diversity (i.e., observed richness, Shannon diversity index) and β-diversity (Bray-Curtis dissimilarity) metrics. Differences in α- and β-diversity across treatment groups were assessed using univariate analysis of variance (ANOVA) implemented by the R function *aov* (Chambers et al., 1992) or multivariate non-parametric analysis of variance (PERMANOVA; Anderson, 2001) as implemented by the *adonis* function in the R package vegan (Oksanen et al., 2016). For ANOVA, parametric assumptions were tested using the Shapiro–Wilk test for normality and the Levene’s test for homogeneity of variance using the R functions *shapiro.test* of the R package stats (R Core Team, 2014) and *leveneTest* of the R package car (Fox and Weisberg, 2019), respectively. Tukey’s HSD test was applied to assess the pairwise comparisons using the *TukeyHSD* function in stats. For PERMANOVA, heteroscedasticity was assessed using permutational analysis of multivariate dispersion (PERMDISP; Anderson, 2006) implemented by the *betadisper* function in vegan. Shifts in β-diversity were analyzed by principal coordinate analysis (PCoA; Gower, 1966) using the *cmdscale* function in R. For understory plant characterization, coverage (total number of plant interceptions), number of species and plant composition (first axis of the plant PCoA) of each site were reported. Correlations between environmental properties (physico-chemical and understory vegetation) and the community-based PCoA ordination scores were determined by using the *envfit* function in vegan. Changes in individual taxonomic groups were assessed by summing up the reads at the phylum and genus levels and testing the changes using ANOVA as outlined above. Pearson correlations between the relative abundance of individual phyla and the physico-chemical soil properties were assessed using function *cor.test* of the stats package.

## Results

### Physical and Chemical Properties

**FIGURE 2:**
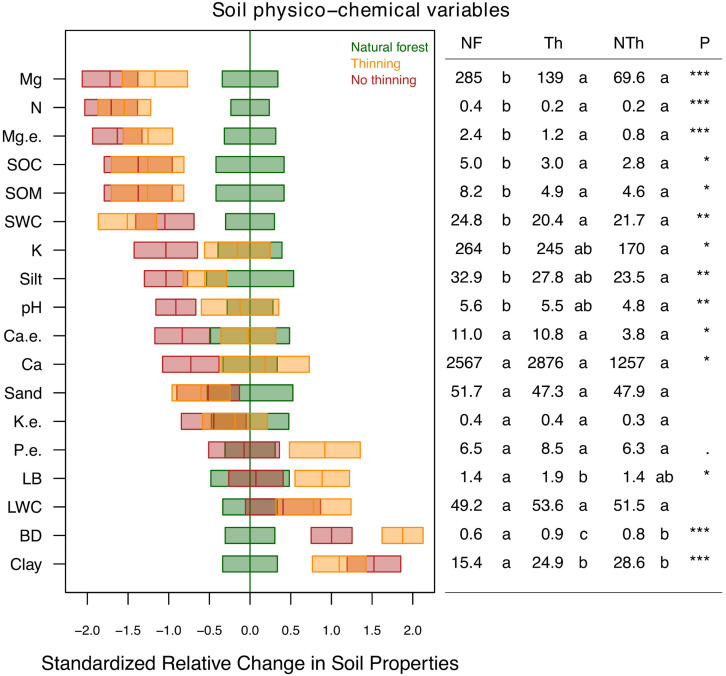
Standardized change in physico-chemical soil properties including clay, silt and sand content (%), pH, soil organic carbon (SOC; %), soil organic matter (SOM; %), soil water content (SWC; %), bulk density (BD; g/cm^3^), litter biomass (LB; kg/m^2^), litter water content (LWC; %), total nitrogen (N; %), extractable phosphorus (P e.; ppm), available cations (Ca, K, Mg; ppm) and exchangeable cations (Ca e., K e., Mg e.; cmol kg-1) across the different pine plantations with thinning (Th, yellow), and no-thinning (NTh, red) treatments in relation to the natural forest strips (NF, green). Boxes represent LMM estimates and standard error (*n* = 3). The table on the right shows the mean values and treatment LMM effect *p*-values (***P < 0.001, **P < 0.01, *P < 0.05,. *P* < 0.1). Different letters indicate significant differences (*P* < 0.05) based on Tukey’s HSD test.

The soil physico-chemical properties changed due to plantation and, to a lower degree, due to thinning practices (Figure 2). Magnesium (available and exchangeable), nitrogen, organic matter, organic carbon and water content was lower in the soils of both plantation treatments compared to natural forests. In general, these differences were slightly lower in thinning plots. In contrast, clay proportion and bulk density were higher in thinning plots. The no-thinning plots showed lower pH, potassium content, and silt proportion compared to natural forest. Litter biomass was higher under thinning treatments compared to natural forest as well as phosphorous but with marginal trends (*F* = 3.2, *P* = 0.06).

### Soil Microbial Communities

**FIGURE 3:**
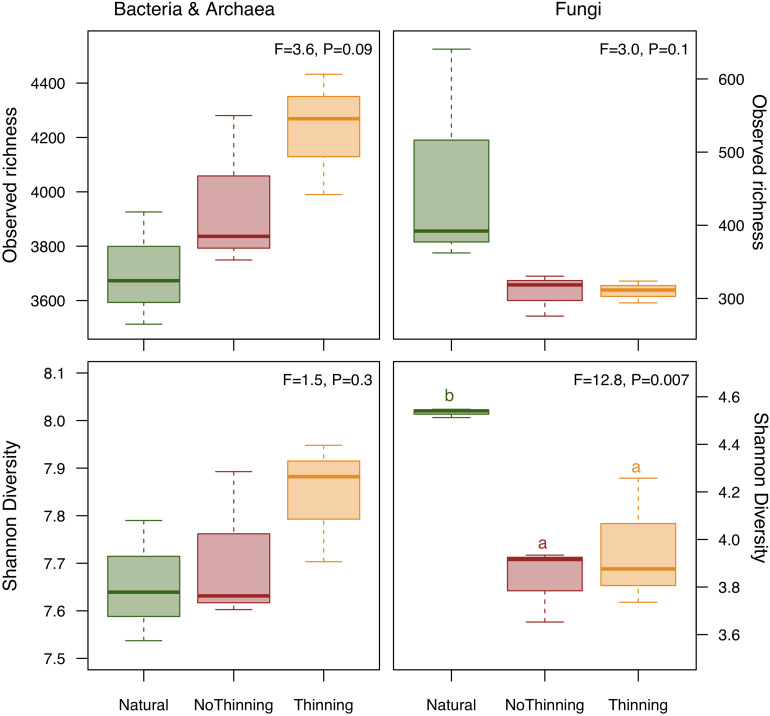
Box plot of differences in observed richness and Shannon diversity index based on bacterial and archaeal **(left)** as well as fungal **(right)** ASVs between natural forest (green), thinning (yellow) and no-thinning (red) pine plantations. ANOVA F-ratios and *P*-values are shown in the upper right corner of each chart. Different letters indicate significant differences (*P* < 0.05) based on Tukey’s HSD test. All normality and homoscedasticity assumptions were met.

A total of 117,414 bacterial 16S rRNA gene sequences and 316,053 fungal ITS2 sequences were obtained for the nine soil samples and binned into 3963 ± 103 and 361 ± 37 ASVs, respectively. Bacterial richness tended to be higher in the pine plantations when compared to the natural forest, with the highest values in the thinned plots (Figure 3). Trends were similar for bacterial Shannon diversity but statistically less clear. Fungal richness tended to decrease in the pine plantations, whereas Shannon diversity was significantly lower in both plantations when compared to the natural forest with no difference between thinning and no-thinning plots.

**FIGURE 4:**
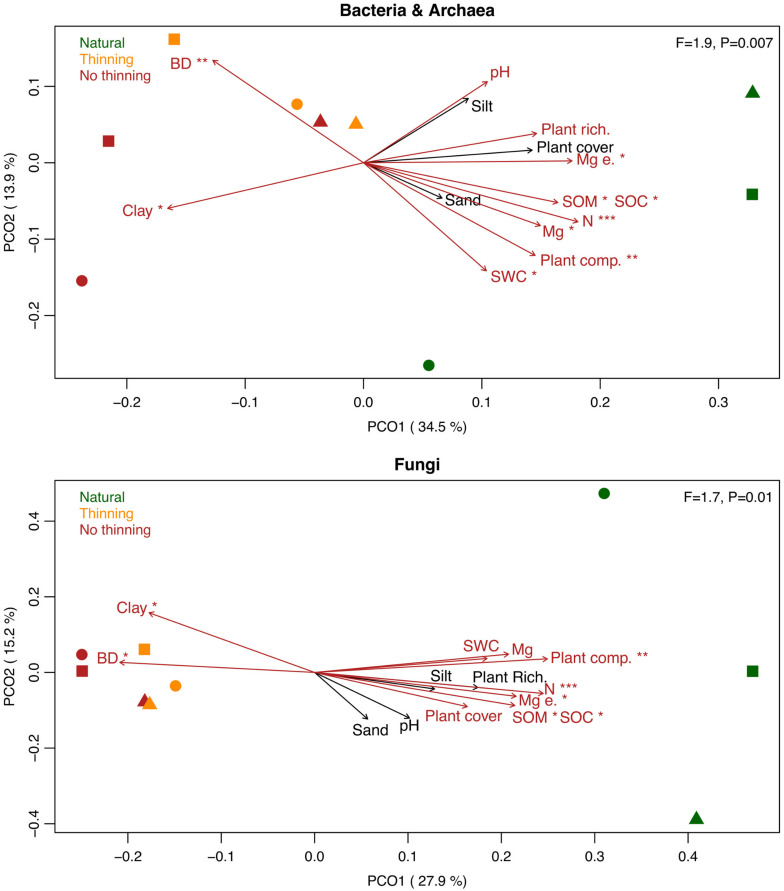
Principal coordinate analysis (PCoA) based on the bacterial/archaeal and fungal Bray-Curtis dissimilarities across samples from the natural forest (green), as well as thinning (yellow), and no-thinning (red) plantations. Different symbols represent the different sites, i.e., P16 (squares), P17 (circles), and SJ (triangles). PERMANOVA F-ratios and *P*-values are provided in the upper right corner. Biplot vectors show correlations between the community ordination scores and the physico-chemical soil properties including clay, sand and silt, pH, soil organic carbon (SOC), soil organic matter (SOM), soil water content (SWC), bulk density (BD), total nitrogen content (N), available and exchangeable magnesium (Mg and Mg e.) and plant understory cover, richness and composition (plant cover, plant rich., and plant comp., respectively). Red arrows represent correlations with *P* < 0.1 and asterisks represent ***P < 0.001, **P < 0.01, *P < 0.05.

Plantations harbored significantly different soil bacterial and fungal communities when compared to the natural forest. Both the non-thinned and thinned plantations were significantly different from the natural forest along the first PCO axis for bacteria (Tukey’s HSD: *P* = 0.02 and *P* = 0.04) and fungi (*P* < 0.001 for both comparisons) (Figure 4). No statistically supported differences were observed between the two pine management treatments. Variability in microbial community composition was smaller in the plantations than in the natural forest, in particular for fungi. The understory plant composition (and to some degree plant richness and cover), and several physico-chemical soil properties including water content, organic matter and carbon, nitrogen and magnesium, bulk density, clay content, and to some extent pH significantly correlated with the shifts in bacterial and fungal community composition (Figure 4).

### Bacterial Taxon-Level Responses

**FIGURE 5:**
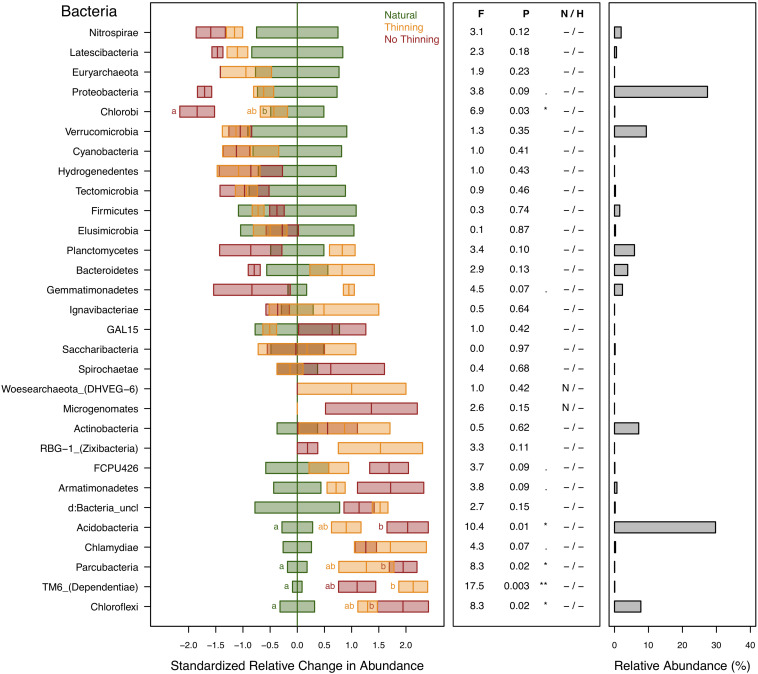
Standardized change in relative abundance of bacterial and archaeal phyla across the different pine plantations (thinning and no-thinning treatments) in relation to the natural forests. Boxes represent means and standard errors (*n* = 3). Different letters indicate significant differences (*P* < 0.05) based on Tukey’s HSD test. The middle panel shows the ANOVA F-ratios and *P*-values and asterisks label different significance levels (**P < 0.01, *P < 0.05,. *P* < 0.1). Violations of assumptions of normality and homoscedasticity are indicated by the presence of a letter N or H, respectively. The panel on the right shows the relative abundance of each phylum in the total community.

Bacterial and archaeal ASVs were assigned to 28 and 2 different phyla, respectively. The most abundant (≥1%) bacterial phyla in the dataset were Acidobacteria (30%), Protobacteria (27%), Verrucomicrobia (9%), Chloroflexi (8%), Actinobacteria (7%), Planctomycetes (6%), Bacteroidetes (4%), Gemmatimonadetes (2.3%), Nitrospirae (2%), and Firmicutes (1.5%). Euryarchaeota and Woesearchaeota were the only archaeal phyla detected and this in low abundance (0.086% of total sequences) and with no statistically supported response to the treatments, even though Euryarchaeota was related to Mg, N, clay, SOC, SOM content and pH (Supplementary Table S1). Woesearchaeota was only found in thinning plots. Several bacterial phyla showed changes in relative abundance due to plantation and thinning (Figure 5). For example, Acidobacteria, Chloroflexi, Dependentiae, and Parcubacteria increased their relative abundances in pine plantations. Some of these groups showed also varying response to thinning, Acidobacteria showed higher values in the no-thinning plots, and Dependentiae in the thinning plots. Chlorobi and, marginally Proteobacteria showed lower abundance in the no-thinning plantations compared to the natural forests (Figure 5) and these phyla were positive related to plant characteristics, SOC and SOM (Supplementary Table S1).

**FIGURE 6:**
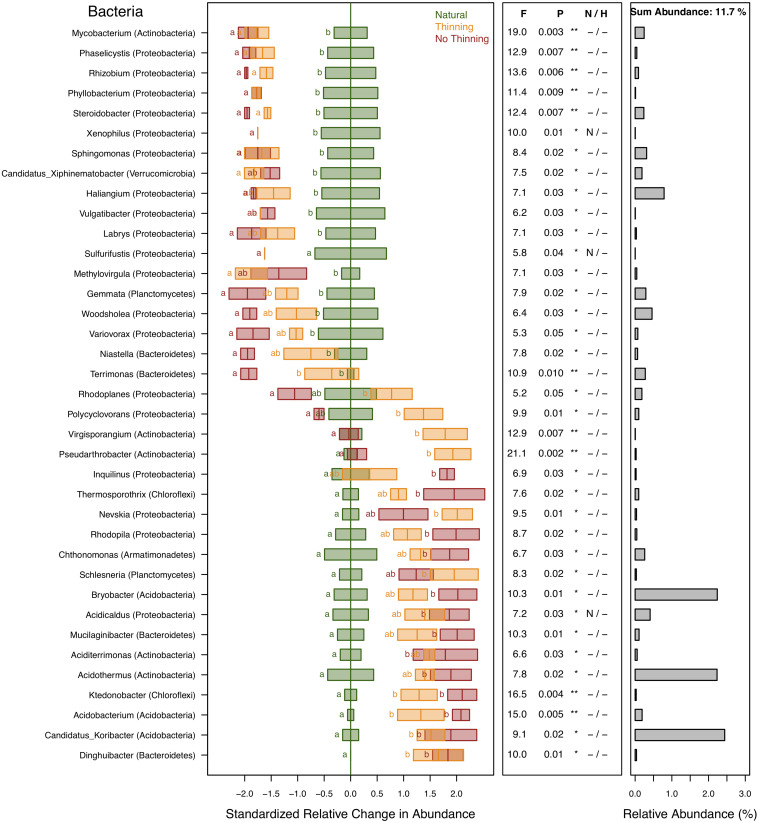
Standardized change in relative abundance of bacterial genera across the different pine plantations (thinning and no-thinning treatments) in relation to the natural forest. Only genera responsive (*P* < 0.05) to the plantation treatments are shown. Boxes represent means and standard errors (*n* = 3). Different letters indicate significant differences based on Tukey’s HSD test. The middle panel shows the ANOVA F-ratios and *P*-values and asterisks labeling different significance levels (**P < 0.01, *P < 0.05). Violations of assumptions of normality and homoscedasticity are indicated by the presence of a letter N or H, respectively. The panel on the right shows the relative abundance of each genus in the total community.

A total of 39% of the bacterial sequences were identified and assigned to 190 different genera of which 37 genera (representing 11.7% of the sequences) changed significantly (*P* < 0.05) due to plantation and, to some degree, management (Figure 6). Almost half of these sequences were predominantly affiliated with only four genera of Acidobacteria (representing 4.9% of the sequences), while most of the remaining genera were distributed between Actinobacteria (2.7%) and Proteobacteria (2.3%). Genera that were reduced in plantations belonged mostly to Proteobacteria including *Phaselicystis, Rhizobium, Phyllobacterium, Xenophilus, Steroidobacter, Sphingomonas, Haliangium, Vulgatibacter, Labrys, Methylovirgula, Sulfurifustis, Woodsholea*, and *Variovorax*, and the rest such as *Mycobacterium, Candidatus Xiphinematobacter*, and *Gemmata* belonged to other phyla, often with a slightly greater response in no-thinning plots. *Niastella* and *Terrimonas* were only reduced in no-thinning plots. *Rhodoplanes* and *Polycyclovorans* presented opposite responses in relation to the management carried out, with lower values in no-thinning and higher in thinning plots, respectively, in relation to the natural forest. *Virgisporangium* and *Pseudarthrobacter* showed only increased in thinning plots, whereas *Inquilinus* only in no-thinning plots. Finally, the greatest response was observed in *Acidothermus, Bryobacter, and Candidatus Koribacter* followed by *Acidobacterium*, *Thermosporothrix*, *Nevskia*, *Rhodoplanes*, *Schlesneria*, *Chthonomonas*, *Acidicaldus*, *Mucilaginibacter*, *Aciditerrimonas*, and *Ktedonobacter* that significant increase in the pine plantations compared to the natural forest, especially in the no-thinning plots except for *Nevskia* and *Schlesneria*. *Dinghuibacter* was only found in pine plantations.

### Fungal Taxon-Level Responses

**FIGURE 7:**
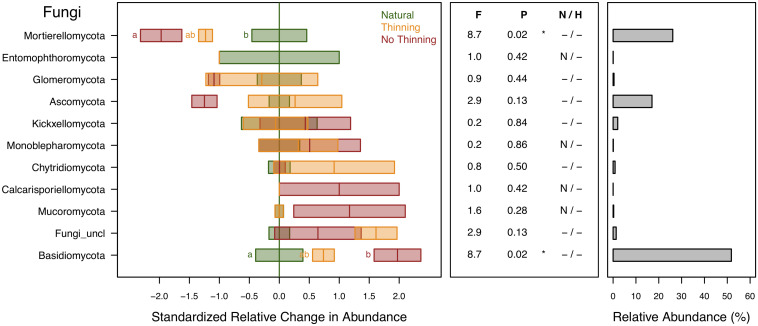
Standardized change in relative abundance of fungal phyla across the different pine plantations (thinning and no-thinning treatments) in relation to the natural forests. Boxes represent means and standard errors (*n* = 3). Different letters indicate significant differences (*P* < 0.05) based on Tukey’s HSD test. The middle panel shows the ANOVA F-ratios and *P*-values are provided in the plot with asterisks labeling different significance levels (*P < 0.05, *P* < 0.1). Violations of assumptions of normality and homoscedasticity are indicated by the presence of a letter N or H, respectively. The right panel shows the relative abundance of each phylum in the total community.

Fungal ASVs were assigned to 10 different phyla, with the most abundant ones being Basidiomycota (52%), Mortierellomycota (26%), and Ascomycota (17%). Relative abundance of Basidiomycota was higher in pine plantations, particularly in the no-thinning plots, whereas Mortierellomycota was lower in the plantations especially in the no-thinning plots (Figure 7). Ascomycota tended to decrease in the no-thinning plots, but the shift was statistically not supported. Moreover, their abundance was strongly related to plant changes (Supplementary Table S2).

**FIGURE 8:**
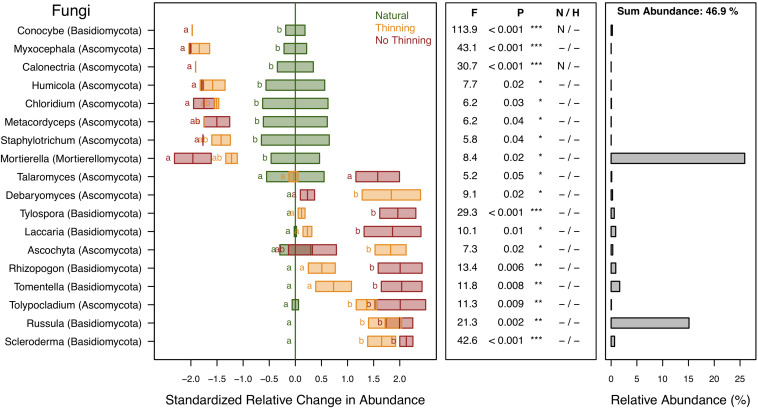
Standardized change in relative abundance of fungal genera across the different pine plantations (thinning and no-thinning treatments) in relation to the natural forest. Only genera responsive (*P* < 0.05) to the plantation treatments are shown. Boxes represent means and standard errors (*n* = 3). Different letters indicate significant differences based on Tukey’s HSD test. The middle panel shows the ANOVA F-ratios and *P*-values are provided in the plot with asterisks labeling different significance levels (***P < 0.001, **P < 0.01, *P < 0.05). Violations of assumptions of normality and homoscedasticity are indicated by the presence of a letter N or H, respectively. The right panel shows the relative abundance of each genus in the total community.

A total of 78.3% of the fungal sequences were identified and assigned to 295 genera of which 18 genera (representing 46.9% of the sequences) changed significantly (*P* < 0.05) due to plantation and, to some degree, management (Figure 8). These genera were predominantly affiliated with the phyla Basidiomycota, Ascomycota, and Mortierellomycota. The genera *Conocybe*, *Myxocephala*, *Calonectria*, *Humicola*, *Chloridium*, *Metacordyceps*, *Staphylotrichum*, and *Mortierella* were all negatively affected by forest conversion. Differences between management types were not statistically supported, although Conocybe and Calonectria were not detected in the no-thinning plantations and Mortierella tended to be further reduced under no-thinning when compared to the thinned plantations. In contrast, *Scleroderma*, *Russula*, *Tolypocladium*, *Tomentella*, *Rhizopogon*, *Ascochyta*, *Laccaria*, *Tylospora*, *Debaryomyces*, and *Talaromyces* increased in relative abundance under both plantation types, whereas several of those genera were not detected in the natural forest (Figure 8). Most of these genera showed greater values under no-thinning, except for *Debaryomyces* and *Ascochyta*.

A complete list of the detected bacterial and fungal taxa, from phylum to ASVs level, including standardized change of relative abundance with respect to the natural forest, relative abundance in the dataset, and test statistics (F ratio and *P*-values), are provided for bacteria (Supplementary Figure 1) and fungi (Supplementary Figure 2).

## Discussion

Forest replacement by exotic pine plantations substantially altered the physical and chemical soil properties. The simplification in vegetation had important consequences for microbial diversity and community composition. Although the different management practices showed subordinate effects in comparison to the forest replacement, soil microbial communities in the thinning treatment were more similar to the natural forest than in no-thinning plantations.

### Physical and Chemical Properties

Almost all measured physico-chemical soil properties changed due to forest replacement and, to some degree, between management practices in the pine plantations (Figure 2). The increase of soil bulk density in pine plantations can mainly be attributed to the use of heavy machinery (Batey, 2009). Higher clay and lower organic matter content, as found in plantation plots, can further facilitate compaction (Hartmann et al., 2014; Murphy, 2015). At the same time, natural forest replacement changed magnesium, nitrogen, carbon and water content in the soil, which is in agreement with previous studies in the Atlantic Forest (Aboim et al., 2008). The lower soil nutrient content in the plantations could be attributed to an increased nutrient uptake by the rapidly growing plantations as well as to increased nutrient leaching associated with lower organic matter content and pH values that affect the capacity to retain nutrients (Johnson et al., 1983; Turner and Lambert, 1988; Berthrong et al., 2009; Murphy, 2015). Major leaching likely occurs before to the establishment of the plantations and during the first years when the vegetation cover is low.

Thinning in pine plantations reportedly changes the microclimatic conditions by increasing radiation and temperature at the soil level and by enhancing plant development in the understory (Trentini et al., 2017). In this study, soil pH dropped in no-thinning plantations. Lack of pH differences between thinned plots and native forest showed that thinning can alleviate soil acidification in comparison to no-thinning plots, maybe due to the buffer capacity of the understory plant cover (Fu et al., 2015). However, Dang et al. (2018) found no differences in pH values after thinning which was probably due to the fact that pH values in that study were almost three orders higher than in our study (8 and 5, respectively). Phosphorus is generally fixed by hydrous oxides of Fe, Al, and Mn, reaching its maximum availability at pH values between 5.5 and 7 (Ahemad et al., 2009). Thus, the slightly higher pH value combined with more litter biomass could be the reason for the marginal increase of phosphorus availability in thinning plots. This result was consistent with other studies (Dang et al., 2018; Xu et al., 2020).

### Soil Microbial Communities

Natural forest replacement influenced fungal and bacterial communities. Fungal alpha diversity decreased whereas bacterial alpha diversity tended to increase in plantations when compared to the natural forest (Figure 3). The sensitivity of fungal alpha diversity to forest replacement with pine plantations has also been shown in a subtropical forest of China (Nie et al., 2012). In contrast to our hypothesis, thinning practices did not alleviate the impact on fungal diversity, which is, however, consistent with recent studies (Cheng et al., 2018; Dang et al., 2018). The trend to higher bacterial alpha diversity in the thinning plots might be attributed to the increase in bulk density that might protect bacteria from predation (Hartmann et al., 2014). Forest replacement by pine plantations was the main factor shaping microbial community composition of both bacteria and fungi, which is in line with previous studies comparing conifer versus deciduous forests (Buée et al., 2009; Kutszegi et al., 2015). In contrast to Nie et al. (2012), change in microbial composition affected fungal and bacterial communities similarly. Community composition in pine plantations were less variable than those from native forests, which could be explained by a more homogeneous vegetation and litter in the plantations (Figure 4). Moreover, thinning management within pine plantations did not significantly influence microbial community composition; however, there was a small but consistent separation of the thinning plots, within each block, toward the native forest direction in the ordination analysis, which is in agreement to previous findings (Dang et al., 2018). The lack of significant differences can be partially attributed to the low statistical power with only three replicated forest stands.

### Environmental Factors That Shape Microbial Community Composition

Microbial community composition was related to both understory plant community and environmental factors. Although it was not possible to disentangle the direct and indirect effects of those two factors because they were not explicitly tested, microbial community composition appeared to be especially related to changes in understory community structure (Figure 4). This is consistent with understory removal studies that have shown changes in microbial community composition upon understory removal (e.g., Wu et al., 2011; Zhao et al., 2013; Murugan et al., 2014). Soil water content was one of the environmental factors that influenced microbial community (Figure 4), as seen in other studies (Castro et al., 2010; de Vries et al., 2018). Shifts in organic matter and carbon content, as a result of the substantial change in aboveground litter quality in pine plantations (Zaninovich et al., 2016), were likely other critical factors that have modulated the microbial community (Figure 4), which might be particularly important drivers for fungi (Baldrian, 2017). Furthermore, soil nitrogen and magnesium content were linked with shifts in forest soil microbial community structure (Figure 4). Magnesium could be linked to microbial composition indirectly as it is part of an essential constituent of plants (chlorophyll) (Levitt, 1954), playing an important role in plant defense and nitrogen uptake (Senbayram et al., 2016), that will enhance plant development and, in turn, will affect the rhizosphere microbiome. At the same time, magnesium could directly affect microbial composition since it is involved in microbial enzyme processes affecting their nutrition and growth (Wyszkowska and Wyszkowski, 2003; Buchachenko et al., 2008). Bulk density and clay content were physical soil properties that shaped microbial community composition, potentially related to changes in oxygen availability. Soil pH appeared to be more important in driving shifts in bacterial rather than in fungal community structure (Figure 4), which is in agreement with other studies (Fierer and Jackson, 2006; Lauber et al., 2008).

### Bacterial Taxon-Level Responses

Shifts in soil biodiversity under forest conversion from AF to pine plantations are poorly studied and assessing the changes of individual taxonomic groups can provide deeper insights into ecological mechanisms of such shifts based on the knowledge about putative lifestyles of the detected taxa in the context of the available literature. Several bacterial phyla showed significant responses to forest replacement and thinning (Figure 5). Proteobacteria and Acidobacteria were the two most abundant phyla in the dataset consistently with previous AF findings (Bruce et al., 2010; Etto et al., 2012; Catão et al., 2014), and showed opposite responses to forest replacement with relative abundance decreasing and increasing, respectively, in the pine plantations. The effect was more pronounced in no-thinning plots. Several major proteobacterial groups are considered to largely exhibit copiotrophic lifestyles and prevalence under high resource availability (Fierer et al., 2007; Hartmann et al., 2017), whereas Acidobacteria are often considered oligotrophic species adapted to resource-limited environments (Fierer et al., 2007; Ward et al., 2009; Hartmann et al., 2017) and lower soil pH values (Fierer and Jackson, 2006; Jones et al., 2009). These different trophic lifestyles might explain the higher relative abundance of Proteobacteria in natural forests and thinned plantations, where more labile carbon and nitrogen compounds might have been available through root exudation and broad-leaf litter fall, as well as the higher relative abundance of Acidobacteria in no-thinning pine plantations that were mainly characterized by recalcitrant litter (i.e., ligning-rich pine needles) and low soil pH values. For example, the acidobacterial genera *Candidatus Koribacter* and *Bryobacter* were the most abundant of all responsive bacterial genera, and both exhibited high relative abundances in the pine plantations especially in no-thinning conditions (Figure 6). Besides, they are able to degrade complex plant polymers and oxidize organic carbon, as well as carry putative genes involved in iron reduction and fermentation pathways, both potentially associated with anaerobic conditions (Eichorst et al., 2018). All these features, together with the capacity of some acidobacterial genera such as *Acidicapsa*, *Acidobacterium*, and *Candidatus Koribacter* to form capsules or develop biofilm (Ward et al., 2009; Kulichevskaya et al., 2012), would allow them to thrive in dry, anoxic, impoverished but iron rich environments, which could explain the increased relative abundance in the plantations.

Besides the fact that copiotrophic life strategies have led to marginal decreases of Protobacteria in pine plantations, also other factors like plant host interaction could explain these shifts. Alphaproteobacteria, for example, was the predominant class within Proteobacteria, representing 16% of the sequences of which 10% corresponded to the order Rhizobiales. Five genera of the Rhizobiales, i.e., *Rhizobium*, *Phyllobacterium*, *Labrys*, *Rhodoplanes*, and *Methylovirgula* exhibited lower values in plantations (Figure 6). The decline of these taxa might have direct consequences for soil nutrient availability since many members of this group are involved in symbiotic relationships with plants and fix atmospheric nitrogen (i.e., Long, 1989; van Rhijn and Vanderleyden, 1995; Mantelin et al., 2006; Ferguson, 2013). Also, members of Rhizobiales have a wide enzymatic capacity to solubilize nutrients in the soil (Vorob’ev et al., 2009; Nguyen et al., 2015). Among the Betaproteobacteria, *Xenophilus* and *Variovorax*, all from the family Comamonadaceae, decreased in no-thinning plantations. The decrease in the availability of host plants in non-thinned plantations could be one of the causes of the lower abundance of *Variovorax*, due to its endophytic lifestyle (Han et al., 2011). In contrast, the genera *Acidicaldus*, *Rhodopila*, and *Inquilinus* of the order Rhodospirillales (Alphaproteobacteria) increased in pine forests, which might be due to their preference for lower pH and higher temperature as reported previously (Ambler et al., 1993; Johnson et al., 2006).

Chlorobi (green sulfur bacteria), probably associated with the phyllosphere (Atamna-Ismaeel et al., 2012), was the only phylum that showed a clear negative response in no-thinning plantations (Figure 5). The low number of native plant species in this treatment might have affected their development; but there is still a lack of information about this group, especially in subtropical forests. Gemmatimonadetes and Planctomycetes tended to increase in the thinned plots that also showed the highest bulk density, which could relate to their ability to tolerate more anoxic and compacted soils as reported previously (Hartmann et al., 2014). On the other side, together with Acidobacteria, the phyla Chloroflexi, Parcubacteria, and Dependentiae increased in relative abundance under the pine plantations. Dependentiae and Parcubacteria belong to candidate phyla radiation (CPR), a monophyletic group highly related to symbiont lifestyles and anoxic conditions (Nelson and Stegen, 2015; Yeoh et al., 2016; Méheust et al., 2019). Both phyla were found to be mainly associated with the phyllosphere of gymnosperm than angiosperm species which together with their anoxic preferences could explain their prevalence in pine plantations (Laforest-Lapointe et al., 2016). In addition, Chloroflexi appeared as one of the six most abundant bacterial phyla in this study in agreement with Hug et al. (2013), who also found that it was involved in acetate fermentation pathways (homoacetogenic lifestyle) for energy generation under more anaerobic conditions. This mechanism allows them to use several plant debris compositions and generates organic acids that acidify the soil, possibly contributing to the pH decline in pine plantations. Furthermore, Chloroflexi phyla were isolated from pine root litter, favored in final litter decay stages (where lignin-rich conditions prevail) and under high moisture conditions, similar to those found in pine plantations under subtropical environment (Herzog et al., 2019).

Several genera of the phylum Actinobacteria, i.e., *Aciditerrimonas*, *Acidothermus*, *Pseudarthrobacter*, *Virgisporangium*, and *Mycobacterium* also changed in relative abundance due to forest replacement (Figure 6). *Acidothermus*, the most abundant actinobacterial genus in the dataset, showed higher relative abundance in pine plantations. This is consistent with its thermophilic and acidophilic capabilities (Mohagheghi et al., 1986) and also attributed to its tolerance toward drier conditions as shown in recent studies (Ullah et al., 2019). *Mycobacterium*, a known root endophyte (Conn and Franco, 2004; Dinesh et al., 2017), is consistently present in the rhizosphere of native crops of the Misiones Province (Bergottini et al., 2017), but it was reduced in the plantations, which may be due to the scarcity of host plants in the understory of no-thinning plantations.

### Fungal Taxon-Level Responses

There were considerable shifts in fungal taxa due to plantation (Figures 7, 8). Among the two most abundant phyla, the decrease of Mortierellomycota along with the increase of Basidiomycota under pine plantations were likely related to changes in litter quality and plant host interactions. The environment in pine plantations favors taxa like Basidiomycota with enzymatic potential to degrade lignin and capable of forming ectomycorrhizal relationships with the pine trees (Boberg et al., 2011; Campi et al., 2015; Vaz et al., 2017). In contrast, Mortierellomycota, which was in this study largely represented by the genus *Mortierella*, live as ubiquitous saprobes in soil and litter. Some species of this genus play a major role in carbon cycling, particularly during the first stage of decay when labile carbohydrates are readily available, although some other species are also able to degrade more complex substrates like chitin and hemicellulose (Richardson, 2009; Dix and Webster, 2012; Taylor and Sinsabaugh, 2015). Furthermore, these fungi thrive on easily available monosaccharides from root exudates (Richardson, 2009; Dix and Webster, 2012) and have been shown to be sensitive to drought (de Vries et al., 2018). Given all these traits, plantations represent less favorable conditions for this fungal group and, consistently, it has not been isolated from needle litter (Brandsberg, 1969). Both phyla, Basidiomycota and Mortierellomycota, revealed much stronger response to the no-thinning treatment.

*Russula*, a common ectomycorrhizal (ECM) fungus associated with pine, was the most abundant genus within the Basidiomycota and only found in the pine plantations. The genus was only represented by *Russula pectinatoides*, a species reported to favor low pH conditions (Kutszegi et al., 2015; Ge et al., 2017) as those occurring under the pine plantations, especially in the no-thinning treatment. The other genera that only occurred in the pine plantations were also common ECM fungi such as *Tylospora*, *Laccaria*, *Rhizopogon*, *Tomentella*, and *Scleroderma*, which is consistent with a previous survey in pine plantations (Campi et al., 2015; Hayward et al., 2015). The fact that these ECM fungi were only found in the pine plantations supports the idea of other studies that they are neither native to Argentina (Nouhra et al., 2012; Hayward et al., 2015; Urcelay et al., 2017) nor to the Atlantic forest (Andrade et al., 2000). Although it is not known whether the pines in this study were actively infected or not, historic inoculation techniques at seedling stage in greenhouse could be responsible for the introduction of ECM into the soil. *Conocybe* is another basidiomycete genus known as a coprophilous fungus on animal dung (Amandeep et al., 2015) and its absence in the pine plantations could reflect a rather low amount of wildlife passing in plantations compared to the natural forest (Iezzi et al., 2018).

Many of the ascomycete genera decreased in pine plantations, especially in no-thinning treatments, including *Myxocephala*, *Calonectria*, *Humicola*, *Chloridium*, *Metacordyceps*, and *Staphylotrichum*. These lower values could be linked to the absence of their specific hosts as for example in case of plant pathogenic fungi such as *Calonectria* (Alfenas et al., 2013), in symbionts (mycorrhizae) such as *Myxocephala* (Weber et al., 1989), in endophytes such as Chloridium (Kharwar et al., 2009), or saprophytes associated to plant debris such as Staphylotrichum (Maharachchikumbura et al., 2016). Also, a decrease of species involved in the biological control of plant disease such as *Humicola phialophoroides* (Ko et al., 2011). However, some ascomycete genera were also promoted under pine plantations, i.e., *Debaryomyces*, *Ascochyta*, and *Tolypocladium*. Species of *Debaryomyces*, a yeast only detected in pine plantations (Figure 8), were reported in previous wood decay surveys (Vaz et al., 2017). Some studies revealed their biological control functions against fungal spoilage of sawn pine timber (Payne and Bruce, 2001) which explains the increases in thinning plots where the largest amount of pine debris were found. Also, the genus *Tolypocladium*, represented only by *T. album*, had higher relative abundance in pine plantations. This species is described as a sapwood endophyte with entomopathogenic potential (Gazis et al., 2014) used in the biological control of insects and mites (Navarro et al., 2019). Despite there is vast information about this species, we have no explanation for the increase in pine plantation.

## Conclusion

The current study highlights the impact of replacing a botanically diverse subtropical native forest by exotic monospecific pine plantations on various soil properties including a detailed assessment of the soil microbiota. Shifting from a rich and diverse to a more impoverished ecosystem resulted in a significant alteration in the composition of both fungi and bacteria. These changes reduced the native, largely copiotrophic, soil microbial community by selecting for specific microbial consortia adapted to a reduced vegetation and more oligotrophic, dry and acidic conditions. Thinning, to some degree, mitigated the effects of plantation on the soil physico-chemical conditions by promoting the development of a more abundant and richer understory vegetation. However, although, some soil microbial taxa showed smaller shifts away from the native community in the thinned plantations, the communities still resembled much more the communities in no-thinning plantations than in the native forests. Although thinning appears not to fully counterbalance changes in the soil microbiome caused by the AF replacement, a partial recovery of the soil microbiota and its associated ecosystem processes could take place by the end of the rotation cycle, if subsequent thinning management promotes the development of native understory species and trees.

## Data Availability Statement

The datasets presented in this study can be found in online repositories. The names of the repository/repositories and accession number(s) can be found below: https://www.ebi.ac.uk/ena, PRJEB36362.

## Author Contributions

CT, MV, and PC designed the study. CT and JF performed the fieldwork and collected the data. JF performed the laboratory analysis. CT and MH conducted the bioinformatics and statistical analyses. CT wrote the first draft of the manuscript. All authors contributed to the manuscript revisions and improvements until the submitted version.

## Conflict of Interest

The authors declare that the research was conducted in the absence of any commercial or financial relationships that could be construed as a potential conflict of interest.
