# Supplementary material for: Thinning Partially Mitigates the Impact of Atlantic Forest Replacement by Pine Monocultures on the Soil Microbiome
Source: Front Microbiol. 2020 Jul 3;11:1491. doi: 10.3389/fmicb.2020.01491 (PMC7350009; doi:10.3389/fmicb.2020.01491)
Supplement: Supplementary file 3 [file Data_Sheet_1.pdf]

## Legends for Supplementary Figures 1 and 2

**Supplementary Figure 1.** Standardized change in relative abundance of archaeal and bacterial taxa arranged by phylum, class, order, family, genus, species and ASV, in thinning (yellow) and no-thinning (red) pine plantations in relation to the natural forests (green). Percentages in brackets indicate the relative abundance of each taxon within the archaeal and bacterial domain separately. Boxes represent means and standard errors ($n = 3$). ANOVA F-ratios and P-values are provided, asterisks labeling different significance levels (** $P < 0.01$, * $P < 0.05$, . $P < 0.1$). Taxa not meeting the assumption of normality and homogeneity variance are labelled by N and H, respectively (Shapiro-Wilk Normality Test and Levene Test, P-values < 0.05).

**Supplementary Figure 2.** Standardized change in relative abundance of fungal taxa arranged by phylum, class, order, family, genus, species and ASV, in thinning (yellow) and no-thinning (red) pine plantations in relation to the natural forests (green). Percentages in brackets indicate the relative abundance of each taxon within the fungal domain. Boxes represent means and standard errors ($n = 3$). ANOVA F-ratios and P-values are provided, asterisks labeling different significance levels (** $P < 0.01$, * $P < 0.05$, . $P < 0.1$). Taxa not meeting the assumption of normality and homogeneity variance are labelled by N and H, respectively (Shapiro-Wilk Normality Test and Levene Test, P-values < 0.05).

**Supplementary Table 1.** Pearson correlation coefficient between the relative abundance of bacterial and archaeal phyla and physico-chemical soil properties including clay, sand and silt, pH, soil organic carbon (SOC), soil organic matter (SOM), soil water content (SWC), bulk density (BD), total nitrogen content (N), available and exchangeable magnesium (Mg and Mg e.) and plant understory cover, richness and composition of all plots (n=9). Pearson correlation coefficient (r) and p-values are provided and grey cells indicate p-values < 0.05.

| Phylum | BD | | Clay | | Sand | | Silt | | Mg | | Mg e. | | N | |
|---|---|---|---|---|---|---|---|---|---|---|---|---|---|---|
| | r | p | r | p | r | p | r | p | r | p | r | p | r | p |
| Acidobacteria | 0.62 | 0.07 | 0.76 | 0.02 | -0.40 | 0.29 | -0.40 | 0.28 | -0.79 | 0.01 | -0.76 | 0.02 | -0.86 | 0.003 |
| Actinobacteria | 0.06 | 0.88 | 0.42 | 0.26 | 0.19 | 0.62 | -0.55 | 0.12 | 0.005 | 0.99 | -0.35 | 0.36 | -0.21 | 0.60 |
| Armatimonadetes | 0.57 | 0.11 | 0.56 | 0.11 | -0.56 | 0.11 | -0.09 | 0.82 | -0.47 | 0.20 | -0.48 | 0.19 | -0.75 | 0.02 |
| Bacteroidetes | 0.35 | 0.36 | -0.14 | 0.72 | -0.54 | 0.13 | 0.56 | 0.12 | 0.34 | 0.37 | 0.31 | 0.42 | -0.04 | 0.92 |
| Chlamydiae | 0.76 | 0.02 | 0.62 | 0.08 | -0.41 | 0.28 | -0.26 | 0.49 | -0.51 | 0.16 | -0.55 | 0.12 | -0.75 | 0.02 |
| Chlorobi | -0.25 | 0.52 | -0.66 | 0.05 | 0.38 | 0.32 | 0.33 | 0.39 | 0.47 | 0.20 | 0.53 | 0.14 | 0.64 | 0.07 |
| Chloroflexi | 0.58 | 0.10 | 0.77 | 0.02 | -0.18 | 0.64 | -0.59 | 0.10 | -0.73 | 0.02 | -0.82 | 0.01 | -0.84 | 0.004 |
| Cyanobacteria | -0.57 | 0.11 | -0.67 | 0.05 | 0.67 | 0.05 | 0.11 | 0.78 | 0.28 | 0.47 | 0.47 | 0.20 | 0.69 | 0.04 |
| d:Bacteria_uncl | 0.73 | 0.03 | 0.87 | 0.002 | -0.57 | 0.11 | -0.37 | 0.32 | -0.41 | 0.27 | -0.67 | 0.05 | -0.78 | 0.01 |
| Elusimicrobia | -0.17 | 0.66 | -0.36 | 0.34 | -0.38 | 0.31 | 0.64 | 0.06 | 0.60 | 0.09 | 0.61 | 0.08 | 0.20 | 0.60 |
| Euryarchaeota | -0.60 | 0.09 | -0.91 | <0.001 | 0.62 | 0.08 | 0.37 | 0.32 | 0.57 | 0.11 | 0.70 | 0.03 | 0.79 | 0.01 |
| FCPU426 | 0.42 | 0.26 | 0.42 | 0.25 | -0.56 | 0.12 | 0.04 | 0.93 | -0.40 | 0.28 | -0.32 | 0.39 | -0.66 | 0.05 |
| Firmicutes | -0.33 | 0.38 | -0.46 | 0.21 | 0.67 | 0.05 | -0.09 | 0.81 | -0.22 | 0.57 | 0.08 | 0.83 | 0.37 | 0.32 |
| GAL15 | 0.04 | 0.91 | 0.51 | 0.17 | -0.08 | 0.84 | -0.42 | 0.26 | -0.30 | 0.43 | -0.46 | 0.22 | -0.28 | 0.46 |
| Gemmatimonadetes | 0.16 | 0.69 | -0.05 | 0.90 | 0.06 | 0.87 | 0.00 | 0.99 | 0.15 | 0.70 | 0.05 | 0.90 | 0.11 | 0.79 |
| Hydrogenedentes | -0.32 | 0.40 | 0.01 | 0.98 | 0.08 | 0.84 | -0.07 | 0.86 | 0.00 | 1.00 | 0.02 | 0.95 | 0.33 | 0.39 |
| Ignavibacteriae | -0.11 | 0.78 | -0.30 | 0.43 | 0.40 | 0.29 | -0.03 | 0.94 | 0.34 | 0.38 | 0.18 | 0.65 | 0.21 | 0.59 |
| Latescibacteria | -0.58 | 0.10 | -0.87 | 0.002 | 0.09 | 0.81 | 0.75 | 0.02 | 0.84 | 0.004 | 0.96 | <0.001 | 0.76 | 0.02 |
| Microgenomates | 0.09 | 0.82 | 0.42 | 0.26 | -0.29 | 0.44 | -0.17 | 0.67 | -0.36 | 0.34 | -0.32 | 0.40 | -0.36 | 0.34 |
| Nitrospirae | -0.60 | 0.09 | -0.89 | 0.001 | 0.47 | 0.20 | 0.47 | 0.20 | 0.54 | 0.14 | 0.75 | 0.02 | 0.82 | 0.01 |
| Parcubacteria | 0.49 | 0.18 | 0.68 | 0.04 | -0.12 | 0.76 | -0.55 | 0.12 | -0.67 | 0.05 | -0.76 | 0.02 | -0.76 | 0.02 |
| Planctomycetes | 0.19 | 0.63 | -0.04 | 0.92 | -0.31 | 0.42 | 0.28 | 0.47 | 0.47 | 0.20 | 0.26 | 0.50 | 0.08 | 0.84 |
| Proteobacteria | -0.33 | 0.39 | -0.25 | 0.52 | 0.43 | 0.25 | -0.10 | 0.79 | 0.29 | 0.45 | 0.17 | 0.67 | 0.54 | 0.13 |
| RBG-1_(Zixibacteria) | 0.57 | 0.11 | 0.13 | 0.73 | -0.25 | 0.52 | 0.07 | 0.86 | -0.14 | 0.72 | -0.20 | 0.61 | -0.37 | 0.33 |
| Saccharibacteria | -0.20 | 0.60 | -0.06 | 0.87 | 0.07 | 0.86 | 0.01 | 0.99 | 0.41 | 0.27 | 0.17 | 0.66 | 0.12 | 0.76 |
| Spirochaetae | -0.17 | 0.66 | 0.13 | 0.74 | -0.18 | 0.63 | 0.02 | 0.95 | 0.08 | 0.84 | 0.04 | 0.91 | -0.03 | 0.93 |
| Tectomicrobia | -0.56 | 0.12 | -0.66 | 0.05 | 0.60 | 0.08 | 0.15 | 0.69 | 0.23 | 0.55 | 0.44 | 0.23 | 0.65 | 0.06 |
| TM6_(Dependentiae) | 0.70 | 0.03 | 0.45 | 0.22 | -0.06 | 0.88 | -0.38 | 0.31 | -0.48 | 0.19 | -0.55 | 0.13 | -0.66 | 0.05 |
| Verrucomicrobia | -0.56 | 0.12 | -0.62 | 0.08 | -0.12 | 0.76 | 0.68 | 0.05 | 0.82 | 0.01 | 0.85 | 0.003 | 0.61 | 0.08 |
| Woesearchaeota_(DHVEG-6) | 0.57 | 0.11 | 0.09 | 0.82 | -0.50 | 0.17 | 0.31 | 0.41 | -0.25 | 0.52 | -0.11 | 0.79 | -0.34 | 0.37 |

**Supplementary Table 1.** continued

| Phylum | pH | | Plant comp. | | Plant cover | | Plant rich. | | SOC | | SOM | | SWC | |
|---|---|---|---|---|---|---|---|---|---|---|---|---|---|---|
| | r | p | r | p | r | p | r | p | r | p | r | p | r | p |
| Acidobacteria | -0.71 | 0.03 | -0.71 | 0.03 | -0.66 | 0.05 | -0.83 | 0.01 | -0.75 | 0.02 | -0.75 | 0.02 | -0.59 | 0.09 |
| Actinobacteria | -0.03 | 0.95 | -0.32 | 0.40 | -0.21 | 0.58 | -0.08 | 0.83 | -0.28 | 0.47 | -0.28 | 0.47 | 0.05 | 0.90 |
| Armatimonadetes | -0.63 | 0.07 | -0.64 | 0.06 | -0.78 | 0.01 | -0.85 | 0.004 | -0.80 | 0.01 | -0.80 | 0.01 | -0.50 | 0.17 |
| Bacteroidetes | 0.36 | 0.34 | -0.09 | 0.82 | -0.08 | 0.84 | 0.33 | 0.38 | -0.21 | 0.58 | -0.21 | 0.58 | -0.13 | 0.74 |
| Chlamydiae | -0.57 | 0.11 | -0.70 | 0.04 | -0.22 | 0.56 | -0.26 | 0.50 | -0.75 | 0.02 | -0.75 | 0.02 | -0.64 | 0.06 |
| Chlorobi | 0.71 | 0.03 | 0.52 | 0.15 | 0.80 | 0.01 | 0.84 | <0.001 | 0.71 | 0.03 | 0.71 | 0.03 | 0.01 | 0.98 |
| Chloroflexi | -0.61 | 0.08 | -0.78 | 0.01 | -0.75 | 0.02 | -0.80 | 0.01 | -0.78 | 0.01 | -0.78 | 0.01 | -0.44 | 0.23 |
| Cyanobacteria | 0.40 | 0.28 | 0.54 | 0.13 | 0.84 | 0.005 | 0.64 | 0.07 | 0.77 | 0.02 | 0.77 | 0.02 | 0.36 | 0.34 |
| d:Bacteria_uncl | -0.45 | 0.22 | -0.64 | 0.06 | -0.49 | 0.18 | -0.33 | 0.38 | -0.78 | 0.01 | -0.78 | 0.01 | -0.56 | 0.12 |
| Elusimicrobia | -0.09 | 0.82 | 0.14 | 0.71 | -0.11 | 0.78 | -0.05 | 0.90 | -0.16 | 0.69 | -0.16 | 0.69 | 0.29 | 0.45 |
| Euryarchaeota | 0.80 | 0.01 | 0.52 | 0.15 | 0.53 | 0.15 | 0.52 | 0.15 | 0.76 | 0.02 | 0.76 | 0.02 | 0.38 | 0.32 |
| FCPU426 | -0.65 | 0.06 | -0.58 | 0.10 | -0.79 | 0.01 | -0.83 | 0.01 | -0.78 | 0.01 | -0.78 | 0.01 | -0.23 | 0.55 |
| Firmicutes | 0.32 | 0.40 | 0.29 | 0.45 | 0.48 | 0.19 | 0.21 | 0.59 | 0.64 | 0.06 | 0.64 | 0.06 | 0.04 | 0.92 |
| GAL15 | -0.56 | 0.11 | -0.03 | 0.93 | -0.39 | 0.29 | -0.54 | 0.13 | -0.20 | 0.61 | -0.20 | 0.61 | -0.08 | 0.83 |
| Gemmatimonadetes | 0.51 | 0.16 | -0.01 | 0.98 | 0.40 | 0.29 | 0.67 | 0.05 | 0.14 | 0.72 | 0.14 | 0.72 | -0.08 | 0.84 |
| Hydrogenedentes | 0.16 | 0.68 | 0.53 | 0.15 | 0.41 | 0.27 | 0.39 | 0.30 | 0.53 | 0.14 | 0.53 | 0.14 | 0.25 | 0.52 |
| Ignavibacteriae | 0.72 | 0.03 | -0.11 | 0.78 | -0.04 | 0.92 | 0.17 | 0.67 | 0.14 | 0.71 | 0.14 | 0.71 | 0.01 | 0.98 |
| Latescibacteria | 0.48 | 0.19 | 0.59 | 0.09 | 0.43 | 0.24 | 0.50 | 0.17 | 0.50 | 0.17 | 0.50 | 0.17 | 0.57 | 0.11 |
| Microgenomates | -0.30 | 0.43 | -0.28 | 0.47 | -0.38 | 0.31 | -0.47 | 0.21 | -0.33 | 0.39 | -0.33 | 0.39 | -0.03 | 0.94 |
| Nitrospirae | 0.69 | 0.04 | 0.66 | 0.05 | 0.74 | 0.02 | 0.69 | 0.04 | 0.84 | 0.005 | 0.84 | 0.005 | 0.39 | 0.30 |
| Parcubacteria | -0.29 | 0.45 | -0.79 | 0.01 | -0.80 | 0.01 | -0.76 | 0.02 | -0.69 | 0.04 | -0.69 | 0.04 | -0.39 | 0.29 |
| Planctomycetes | 0.40 | 0.29 | -0.01 | 0.98 | 0.18 | 0.65 | 0.54 | 0.13 | -0.09 | 0.81 | -0.09 | 0.81 | -0.03 | 0.93 |
| Proteobacteria | 0.52 | 0.15 | 0.58 | 0.10 | 0.61 | 0.08 | 0.71 | 0.03 | 0.70 | 0.03 | 0.70 | 0.03 | 0.18 | 0.64 |
| RBG-1_(Zixibacteria) | 0.48 | 0.19 | -0.51 | 0.16 | -0.36 | 0.34 | 0.005 | 0.99 | -0.35 | 0.36 | -0.35 | 0.36 | -0.49 | 0.18 |
| Saccharibacteria | 0.36 | 0.34 | -0.08 | 0.83 | -0.32 | 0.39 | -0.06 | 0.89 | -0.09 | 0.81 | -0.09 | 0.81 | 0.33 | 0.39 |
| Spirochaetae | -0.02 | 0.95 | -0.07 | 0.86 | -0.21 | 0.58 | -0.14 | 0.71 | -0.15 | 0.69 | -0.15 | 0.69 | 0.33 | 0.38 |
| Tectomicrobia | 0.62 | 0.08 | 0.49 | 0.18 | 0.62 | 0.07 | 0.51 | 0.16 | 0.78 | 0.01 | 0.78 | 0.01 | 0.34 | 0.38 |
| TM6_(Dependentiae) | -0.21 | 0.59 | -0.79 | 0.01 | -0.24 | 0.53 | -0.17 | 0.66 | -0.64 | 0.06 | -0.64 | 0.06 | -0.57 | 0.11 |
| Verrucomicrobia | 0.17 | 0.66 | 0.53 | 0.15 | 0.24 | 0.53 | 0.31 | 0.41 | 0.28 | 0.47 | 0.28 | 0.47 | 0.67 | 0.05 |
| Woesearchaeota_(DHVEG-6) | 0.18 | 0.65 | -0.30 | 0.43 | -0.20 | 0.60 | 0.05 | 0.91 | -0.28 | 0.46 | -0.28 | 0.46 | -0.44 | 0.24 |

**Supplementary Table 2.** Pearson correlation coefficient between the relative abundance of fungal phyla and physico-chemical soil properties including clay, sand and silt, pH, soil organic carbon (SOC), soil organic matter (SOM), soil water content (SWC), bulk density (BD), total nitrogen

content (N), available and exchangeable magnesium (Mg and Mg e.) and plant understory cover, richness and composition of all plots (n=9). Pearson correlation coefficient (r) and p-values are provided and grey cells indicate p-values < 0.05.

| Fungi | BD | | Sand | | Silt | | Clay | | Mg | | Mg e. | | N | |
|---|---|---|---|---|---|---|---|---|---|---|---|---|---|---|
| | r | p | r | p | r | p | r | p | r | p | r | p | r | p |
| Ascomycota | -0.01 | 0.99 | 0.13 | 0.73 | 0.15 | 0.69 | -0.27 | 0.48 | 0.28 | 0.47 | 0.29 | 0.45 | 0.33 | 0.39 |
| Basidiomycota | 0.31 | 0.42 | -0.21 | 0.59 | -0.49 | 0.18 | 0.69 | 0.04 | -0.53 | 0.15 | -0.64 | 0.07 | -0.69 | 0.04 |
| Calcarisporiellomycota | -0.10 | 0.80 | 0.32 | 0.41 | -0.49 | 0.18 | 0.25 | 0.52 | -0.37 | 0.32 | -0.37 | 0.33 | -0.21 | 0.58 |
| Chytridiomycota | 0.51 | 0.16 | -0.38 | 0.32 | 0.28 | 0.47 | 0.02 | 0.96 | -0.30 | 0.43 | -0.11 | 0.79 | -0.30 | 0.44 |
| Entomophthoromycota | -0.44 | 0.23 | -0.25 | 0.52 | 0.79 | 0.01 | -0.63 | 0.07 | 0.80 | 0.01 | 0.86 | <0.001 | 0.53 | 0.15 |
| Fungi_uncl | 0.86 | 0.003 | -0.57 | 0.11 | 0.27 | 0.48 | 0.19 | 0.62 | -0.36 | 0.34 | -0.23 | 0.55 | -0.59 | 0.09 |
| Glomeromycota | -0.38 | 0.32 | 0.26 | 0.49 | 0.20 | 0.60 | -0.43 | 0.24 | 0.75 | 0.02 | 0.49 | 0.18 | 0.49 | 0.18 |
| Kickxellomycota | 0.28 | 0.46 | -0.58 | 0.10 | 0.59 | 0.10 | -0.14 | 0.72 | 0.00 | 1.00 | 0.22 | 0.58 | -0.23 | 0.55 |
| Monoblepharomycota | -0.14 | 0.73 | 0.38 | 0.32 | -0.29 | 0.45 | -0.01 | 0.98 | 0.06 | 0.87 | -0.08 | 0.84 | -0.04 | 0.92 |
| Mortierellomycota | -0.51 | 0.16 | 0.34 | 0.37 | 0.42 | 0.26 | -0.72 | 0.03 | 0.55 | 0.12 | 0.64 | 0.06 | 0.79 | 0.01 |
| Mucoromycota | -0.07 | 0.87 | -0.11 | 0.78 | -0.35 | 0.36 | 0.46 | 0.22 | -0.34 | 0.37 | -0.35 | 0.36 | -0.26 | 0.50 |

**Supplementary Table 2.** continued

| Fungi | pH | | Plant comp. | | Plant cover | | Plant Rich. | | SOC | | SOM | | SWC | |
|---|---|---|---|---|---|---|---|---|---|---|---|---|---|---|
| | r | p | r | p | r | p | r | p | r | p | r | p | r | p |
| Ascomycota | 0.21 | 0.58 | 0.28 | 0.46 | 0.81 | 0.01 | 0.81 | 0.01 | 0.34 | 0.37 | 0.34 | 0.37 | -0.04 | 0.91 |
| Basidiomycota | -0.68 | 0.04 | -0.64 | 0.07 | -0.81 | 0.01 | -0.87 | 0.002 | -0.74 | 0.02 | -0.74 | 0.02 | -0.12 | 0.76 |
| Calcarisporiellomycota | -0.56 | 0.12 | -0.19 | 0.62 | -0.29 | 0.44 | -0.50 | 0.17 | -0.19 | 0.62 | -0.19 | 0.62 | 0.19 | 0.62 |
| Chytridiomycota | 0.19 | 0.63 | -0.27 | 0.48 | -0.13 | 0.74 | 0.06 | 0.88 | -0.20 | 0.61 | -0.20 | 0.61 | -0.42 | 0.26 |
| Entomophthoromycota | 0.18 | 0.65 | 0.45 | 0.23 | 0.10 | 0.80 | 0.22 | 0.57 | 0.17 | 0.66 | 0.17 | 0.66 | 0.58 | 0.10 |
| Fungi_uncl | 0.13 | 0.73 | -0.64 | 0.07 | -0.25 | 0.52 | -0.10 | 0.80 | -0.54 | 0.14 | -0.54 | 0.14 | -0.88 | 0.002 |
| Glomeromycota | 0.66 | 0.05 | 0.26 | 0.51 | 0.04 | 0.92 | 0.30 | 0.43 | 0.28 | 0.47 | 0.28 | 0.47 | 0.35 | 0.36 |
| Kickxellomycota | -0.25 | 0.51 | -0.18 | 0.65 | -0.38 | 0.31 | -0.41 | 0.28 | -0.37 | 0.32 | -0.37 | 0.32 | -0.24 | 0.53 |
| Monoblepharomycota | -0.06 | 0.87 | -0.23 | 0.56 | -0.36 | 0.34 | -0.35 | 0.36 | -0.18 | 0.65 | -0.18 | 0.65 | 0.21 | 0.58 |
| Mortierellomycota | 0.74 | 0.02 | 0.75 | 0.02 | 0.71 | 0.03 | 0.75 | 0.02 | 0.87 | 0.002 | 0.87 | 0.002 | 0.27 | 0.49 |
| Mucoromycota | -0.38 | 0.31 | -0.17 | 0.66 | -0.22 | 0.57 | -0.30 | 0.43 | -0.23 | 0.55 | -0.23 | 0.55 | 0.23 | 0.55 |
