# Supplementary material for: Thinning Partially Mitigates the Impact of Atlantic Forest Replacement by Pine Monocultures on the Soil Microbiome
Source: Front Microbiol. 2020 Jul 3;11:1491. doi: 10.3389/fmicb.2020.01491 (PMC7350009; doi:10.3389/fmicb.2020.01491)
Supplement: Supplementary file 1 [file Image_1.PDF]

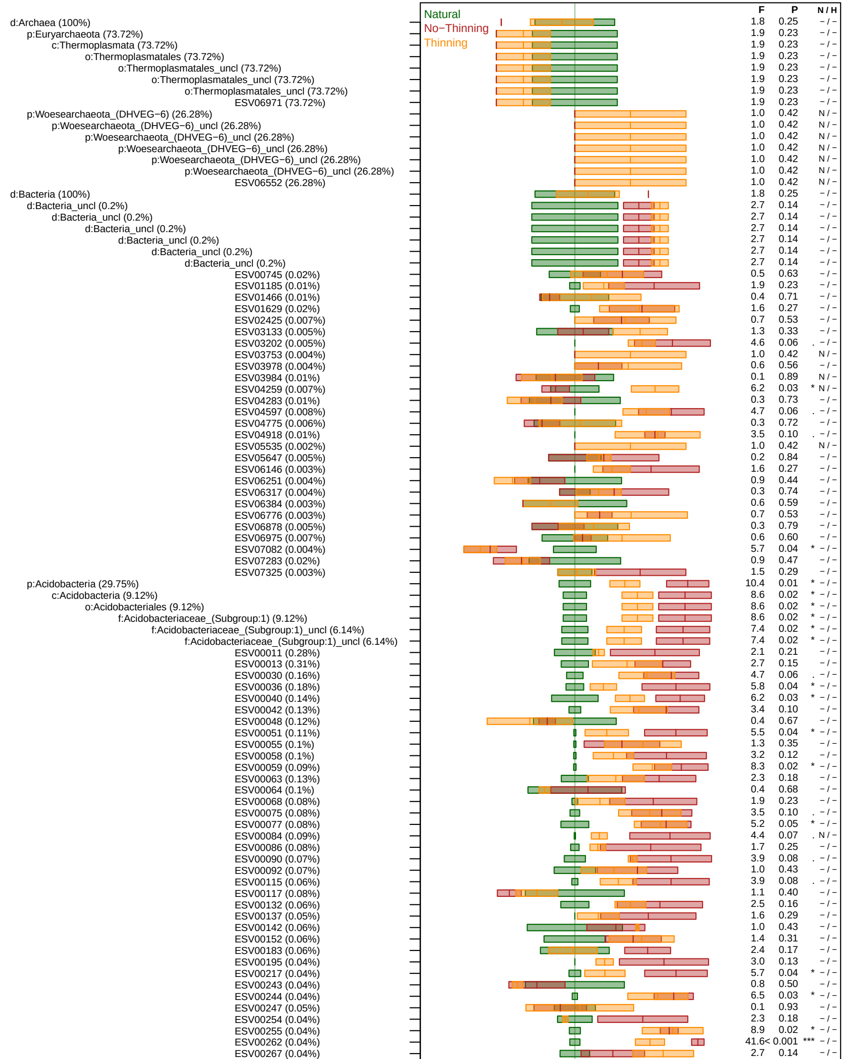
Natural
No−Thinning
Thinning
F P N / H
d:Archaea (100%) 1.8 0.25 - / -
p:Euryarchaeota (73.72%) 1.9 0.23 - / -
c:Thermoplasmata (73.72%) 1.9 0.23 - / -
o:Thermoplasmatales (73.72%) 1.9 0.23 - / -
o:Thermoplasmatales_uncl (73.72%) 1.9 0.23 - / -
o:Thermoplasmatales_uncl (73.72%) 1.9 0.23 - / -
o:Thermoplasmatales_uncl (73.72%) 1.9 0.23 - / -
ESV06971 (73.72%) 1.9 0.23 - / -
p:Woesearchaeota_(DHVEG−6) (26.28%) 1.0 0.42 N / -
p:Woesearchaeota_(DHVEG−6)_uncl (26.28%) 1.0 0.42 N / -
p:Woesearchaeota_(DHVEG−6)_uncl (26.28%) 1.0 0.42 N / -
p:Woesearchaeota_(DHVEG−6)_uncl (26.28%) 1.0 0.42 N / -
p:Woesearchaeota_(DHVEG−6)_uncl (26.28%) 1.0 0.42 N / -
p:Woesearchaeota_(DHVEG−6)_uncl (26.28%) 1.0 0.42 N / -
ESV06552 (26.28%) 1.0 0.42 N / -
d:Bacteria (100%) 1.8 0.25 - / -
d:Bacteria_uncl (0.2%) 2.7 0.14 - / -
d:Bacteria_uncl (0.2%) 2.7 0.14 - / -
d:Bacteria_uncl (0.2%) 2.7 0.14 - / -
d:Bacteria_uncl (0.2%) 2.7 0.14 - / -
d:Bacteria_uncl (0.2%) 2.7 0.14 - / -
d:Bacteria_uncl (0.2%) 2.7 0.14 - / -
ESV00745 (0.02%) 0.5 0.63 - / -
ESV01185 (0.01%) 1.9 0.23 - / -
ESV01466 (0.01%) 0.4 0.71 - / -
ESV01629 (0.02%) 1.6 0.27 - / -
ESV02425 (0.007%) 0.7 0.53 - / -
ESV03133 (0.005%) 1.3 0.33 - / -
ESV03202 (0.005%) 4.6 0.06 . - / -
ESV03753 (0.004%) 1.0 0.42 N / -
ESV03978 (0.004%) 0.6 0.56 - / -
ESV03984 (0.01%) 0.1 0.89 N / -
ESV04259 (0.007%) 6.2 0.03 * N / -
ESV04283 (0.01%) 0.3 0.73 - / -
ESV04597 (0.008%) 4.7 0.06 . - / -
ESV04775 (0.006%) 0.3 0.72 - / -
ESV04918 (0.01%) 3.5 0.10 . - / -
ESV05535 (0.002%) 1.0 0.42 N / -
ESV05647 (0.005%) 0.2 0.84 - / -
ESV06146 (0.003%) 1.6 0.27 - / -
ESV06251 (0.004%) 0.9 0.44 - / -
ESV06317 (0.004%) 0.3 0.74 - / -
ESV06384 (0.003%) 0.6 0.59 - / -
ESV06776 (0.003%) 0.7 0.53 - / -
ESV06878 (0.005%) 0.3 0.79 - / -
ESV06975 (0.007%) 0.6 0.60 - / -
ESV07082 (0.004%) 5.7 0.04 * - / -
ESV07283 (0.02%) 0.9 0.47 - / -
ESV07325 (0.003%) 1.5 0.29 - / -
p:Acidobacteria (29.75%) 10.4 0.01 * - / -
c:Acidobacteria (9.12%) 8.6 0.02 * - / -
o:Acidobacteriales (9.12%) 8.6 0.02 * - / -
f:Acidobacteriaceae_(Subgroup:1) (9.12%) 8.6 0.02 * - / -
f:Acidobacteriaceae_(Subgroup:1)_uncl (6.14%) 7.4 0.02 * - / -
f:Acidobacteriaceae_(Subgroup:1)_uncl (6.14%) 7.4 0.02 * - / -
ESV00011 (0.28%) 2.1 0.21 - / -
ESV00013 (0.31%) 2.7 0.15 - / -
ESV00030 (0.16%) 4.7 0.06 . - / -
ESV00036 (0.18%) 5.8 0.04 * - / -
ESV00040 (0.14%) 6.2 0.03 * - / -
ESV00042 (0.13%) 3.4 0.10 - / -
ESV00048 (0.12%) 0.4 0.67 - / -
ESV00051 (0.11%) 5.5 0.04 * - / -
ESV00055 (0.1%) 1.3 0.35 - / -
ESV00058 (0.1%) 3.2 0.12 - / -
ESV00059 (0.09%) 8.3 0.02 * - / -
ESV00063 (0.13%) 2.3 0.18 - / -
ESV00064 (0.1%) 0.4 0.68 - / -
ESV00068 (0.08%) 1.9 0.23 - / -
ESV00075 (0.08%) 3.5 0.10 . - / -
ESV00077 (0.08%) 5.2 0.05 * - / -
ESV00084 (0.09%) 4.4 0.07 . N / -
ESV00086 (0.08%) 1.7 0.25 - / -
ESV00090 (0.07%) 3.9 0.08 . - / -
ESV00092 (0.07%) 1.0 0.43 - / -
ESV00115 (0.06%) 3.9 0.08 . - / -
ESV00117 (0.08%) 1.1 0.40 - / -
ESV00132 (0.06%) 2.5 0.16 - / -
ESV00137 (0.05%) 1.6 0.29 - / -
ESV00142 (0.06%) 1.0 0.43 - / -
ESV00152 (0.06%) 1.4 0.31 - / -
ESV00183 (0.06%) 2.4 0.17 - / -
ESV00195 (0.04%) 3.0 0.13 - / -
ESV00217 (0.04%) 5.7 0.04 * - / -
ESV00243 (0.04%) 0.8 0.50 - / -
ESV00244 (0.04%) 6.5 0.03 * - / -
ESV00247 (0.05%) 0.1 0.93 - / -
ESV00254 (0.04%) 2.3 0.18 - / -
ESV00255 (0.04%) 8.9 0.02 * - / -
ESV00262 (0.04%) 41.6< 0.001 *** - / -
ESV00267 (0.04%) 2.7 0.14 - / -

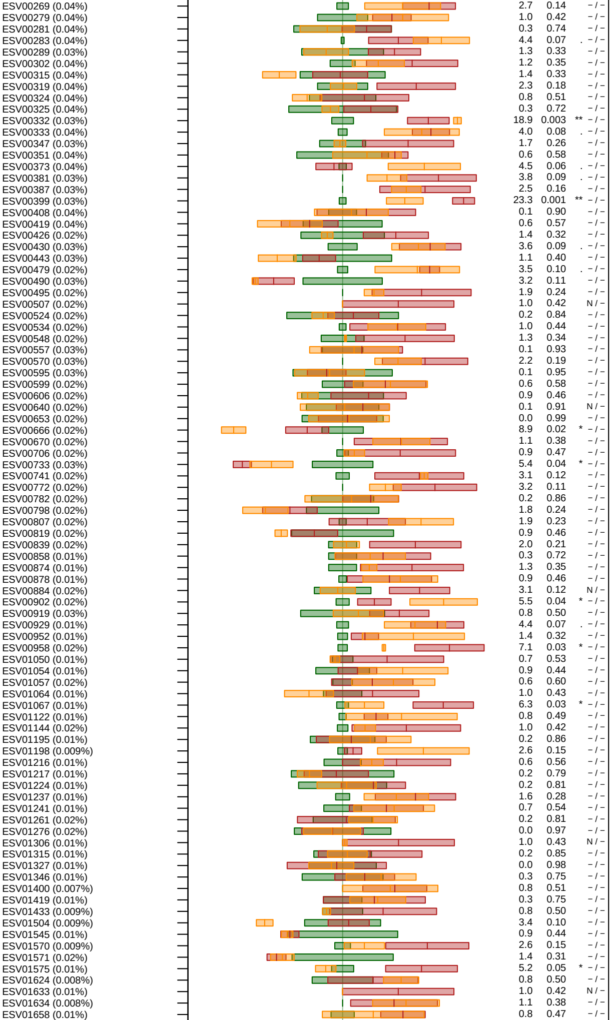
ESV00269 (0.04%) 2.7 0.14 – / –
ESV00279 (0.04%) 1.0 0.42 – / –
ESV00281 (0.04%) 0.3 0.74 – / –
ESV00283 (0.04%) 4.4 0.07 . – / –
ESV00289 (0.03%) 1.3 0.33 – / –
ESV00302 (0.04%) 1.2 0.35 – / –
ESV00315 (0.04%) 1.4 0.33 – / –
ESV00319 (0.04%) 2.3 0.18 – / –
ESV00324 (0.04%) 0.8 0.51 – / –
ESV00325 (0.04%) 0.3 0.72 – / –
ESV00332 (0.03%) 18.9 0.003 ** – / –
ESV00333 (0.04%) 4.0 0.08 . – / –
ESV00347 (0.03%) 1.7 0.26 – / –
ESV00351 (0.04%) 0.6 0.58 – / –
ESV00373 (0.04%) 4.5 0.06 . – / –
ESV00381 (0.03%) 3.8 0.09 . – / –
ESV00387 (0.03%) 2.5 0.16 – / –
ESV00399 (0.03%) 23.3 0.001 ** – / –
ESV00408 (0.04%) 0.1 0.90 – / –
ESV00419 (0.04%) 0.6 0.57 – / –
ESV00426 (0.02%) 1.4 0.32 – / –
ESV00430 (0.03%) 3.6 0.09 . – / –
ESV00443 (0.03%) 1.1 0.40 – / –
ESV00479 (0.02%) 3.5 0.10 . – / –
ESV00490 (0.03%) 3.2 0.11 – / –
ESV00495 (0.02%) 1.9 0.24 – / –
ESV00507 (0.02%) 1.0 0.42 N / –
ESV00524 (0.02%) 0.2 0.84 – / –
ESV00534 (0.02%) 1.0 0.44 – / –
ESV00548 (0.02%) 1.3 0.34 – / –
ESV00557 (0.03%) 0.1 0.93 – / –
ESV00570 (0.03%) 2.2 0.19 – / –
ESV00595 (0.03%) 0.1 0.95 – / –
ESV00599 (0.02%) 0.6 0.58 – / –
ESV00606 (0.02%) 0.9 0.46 – / –
ESV00640 (0.02%) 0.1 0.91 N / –
ESV00653 (0.02%) 0.0 0.99 – / –
ESV00666 (0.02%) 8.9 0.02 * – / –
ESV00670 (0.02%) 1.1 0.38 – / –
ESV00706 (0.02%) 0.9 0.47 – / –
ESV00733 (0.03%) 5.4 0.04 * – / –
ESV00741 (0.02%) 3.1 0.12 – / –
ESV00772 (0.02%) 3.2 0.11 – / –
ESV00782 (0.02%) 0.2 0.86 – / –
ESV00798 (0.02%) 1.8 0.24 – / –
ESV00807 (0.02%) 1.9 0.23 – / –
ESV00819 (0.02%) 0.9 0.46 – / –
ESV00839 (0.02%) 2.0 0.21 – / –
ESV00858 (0.01%) 0.3 0.72 – / –
ESV00874 (0.01%) 1.3 0.35 – / –
ESV00878 (0.01%) 0.9 0.46 – / –
ESV00884 (0.02%) 3.1 0.12 N / –
ESV00902 (0.02%) 5.5 0.04 * – / –
ESV00919 (0.03%) 0.8 0.50 – / –
ESV00929 (0.01%) 4.4 0.07 . – / –
ESV00952 (0.01%) 1.4 0.32 – / –
ESV00958 (0.02%) 7.1 0.03 * – / –
ESV01050 (0.01%) 0.7 0.53 – / –
ESV01054 (0.01%) 0.9 0.44 – / –
ESV01057 (0.02%) 0.6 0.60 – / –
ESV01064 (0.01%) 1.0 0.43 – / –
ESV01067 (0.01%) 6.3 0.03 * – / –
ESV01122 (0.01%) 0.8 0.49 – / –
ESV01144 (0.02%) 1.0 0.42 – / –
ESV01195 (0.01%) 0.2 0.86 – / –
ESV01198 (0.009%) 2.6 0.15 – / –
ESV01216 (0.01%) 0.6 0.56 – / –
ESV01217 (0.01%) 0.2 0.79 – / –
ESV01224 (0.01%) 0.2 0.81 – / –
ESV01237 (0.01%) 1.6 0.28 – / –
ESV01241 (0.01%) 0.7 0.54 – / –
ESV01261 (0.02%) 0.2 0.81 – / –
ESV01276 (0.02%) 0.0 0.97 – / –
ESV01306 (0.01%) 1.0 0.43 N / –
ESV01315 (0.01%) 0.2 0.85 – / –
ESV01327 (0.01%) 0.0 0.98 – / –
ESV01346 (0.01%) 0.3 0.75 – / –
ESV01400 (0.007%) 0.8 0.51 – / –
ESV01419 (0.01%) 0.3 0.75 – / –
ESV01433 (0.009%) 0.8 0.50 – / –
ESV01504 (0.009%) 3.4 0.10 – / –
ESV01545 (0.01%) 0.9 0.44 – / –
ESV01570 (0.009%) 2.6 0.15 – / –
ESV01571 (0.02%) 1.4 0.31 – / –
ESV01575 (0.01%) 5.2 0.05 * – / –
ESV01624 (0.008%) 0.8 0.50 – / –
ESV01633 (0.01%) 1.0 0.42 N / –
ESV01634 (0.008%) 1.1 0.38 – / –
ESV01658 (0.01%) 0.8 0.47 – / –

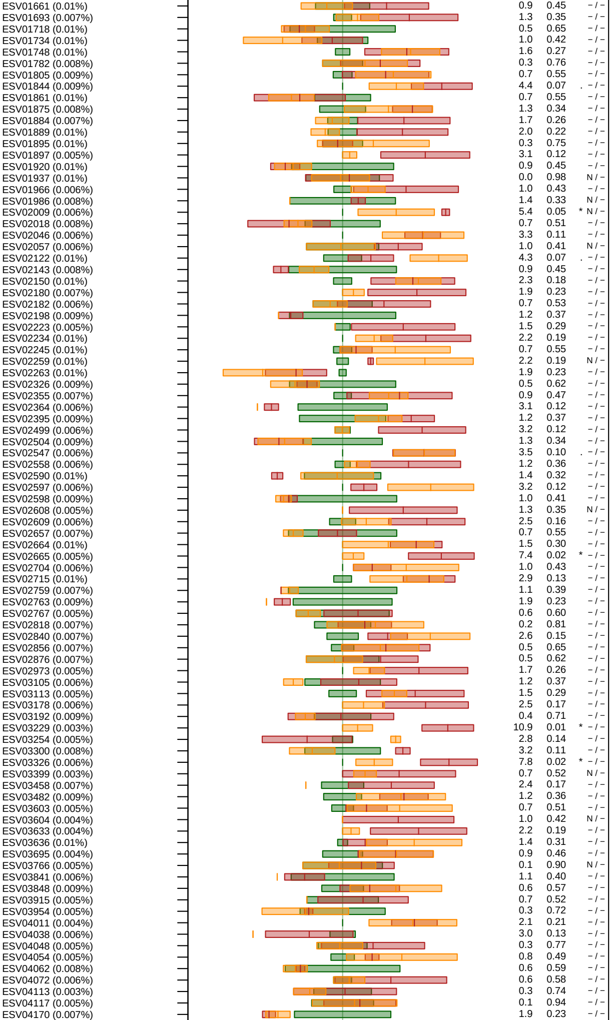
ESV01661 (0.01%) 0.9 0.45 - / -
ESV01693 (0.007%) 1.3 0.35 - / -
ESV01718 (0.01%) 0.5 0.65 - / -
ESV01734 (0.01%) 1.0 0.42 - / -
ESV01748 (0.01%) 1.6 0.27 - / -
ESV01782 (0.008%) 0.3 0.76 - / -
ESV01805 (0.009%) 0.7 0.55 - / -
ESV01844 (0.009%) 4.4 0.07 . - / -
ESV01861 (0.01%) 0.7 0.55 - / -
ESV01875 (0.008%) 1.3 0.34 - / -
ESV01884 (0.007%) 1.7 0.26 - / -
ESV01889 (0.01%) 2.0 0.22 - / -
ESV01895 (0.01%) 0.3 0.75 - / -
ESV01897 (0.005%) 3.1 0.12 - / -
ESV01920 (0.01%) 0.9 0.45 - / -
ESV01937 (0.01%) 0.0 0.98 N / -
ESV01966 (0.006%) 1.0 0.43 - / -
ESV01986 (0.008%) 1.4 0.33 N / -
ESV02009 (0.006%) 5.4 0.05 * N / -
ESV02018 (0.008%) 0.7 0.51 - / -
ESV02046 (0.006%) 3.3 0.11 - / -
ESV02057 (0.006%) 1.0 0.41 N / -
ESV02122 (0.01%) 4.3 0.07 . - / -
ESV02143 (0.008%) 0.9 0.45 - / -
ESV02150 (0.01%) 2.3 0.18 - / -
ESV02180 (0.007%) 1.9 0.23 - / -
ESV02182 (0.006%) 0.7 0.53 - / -
ESV02198 (0.009%) 1.2 0.37 - / -
ESV02223 (0.005%) 1.5 0.29 - / -
ESV02234 (0.01%) 2.2 0.19 - / -
ESV02245 (0.01%) 0.7 0.55 - / -
ESV02259 (0.01%) 2.2 0.19 N / -
ESV02263 (0.01%) 1.9 0.23 - / -
ESV02326 (0.009%) 0.5 0.62 - / -
ESV02355 (0.007%) 0.9 0.47 - / -
ESV02364 (0.006%) 3.1 0.12 - / -
ESV02395 (0.009%) 1.2 0.37 - / -
ESV02499 (0.006%) 3.2 0.12 - / -
ESV02504 (0.009%) 1.3 0.34 - / -
ESV02547 (0.006%) 3.5 0.10 . - / -
ESV02558 (0.006%) 1.2 0.36 - / -
ESV02590 (0.01%) 1.4 0.32 - / -
ESV02597 (0.006%) 3.2 0.12 - / -
ESV02598 (0.009%) 1.0 0.41 - / -
ESV02608 (0.005%) 1.3 0.35 N / -
ESV02609 (0.006%) 2.5 0.16 - / -
ESV02657 (0.007%) 0.7 0.55 - / -
ESV02664 (0.01%) 1.5 0.30 - / -
ESV02665 (0.005%) 7.4 0.02 * - / -
ESV02704 (0.006%) 1.0 0.43 - / -
ESV02715 (0.01%) 2.9 0.13 - / -
ESV02759 (0.007%) 1.1 0.39 - / -
ESV02763 (0.009%) 1.9 0.23 - / -
ESV02767 (0.005%) 0.6 0.60 - / -
ESV02818 (0.007%) 0.2 0.81 - / -
ESV02840 (0.007%) 2.6 0.15 - / -
ESV02856 (0.007%) 0.5 0.65 - / -
ESV02876 (0.007%) 0.5 0.62 - / -
ESV02973 (0.005%) 1.7 0.26 - / -
ESV03105 (0.006%) 1.2 0.37 - / -
ESV03113 (0.005%) 1.5 0.29 - / -
ESV03178 (0.006%) 2.5 0.17 - / -
ESV03192 (0.009%) 0.4 0.71 - / -
ESV03229 (0.003%) 10.9 0.01 * - / -
ESV03254 (0.005%) 2.8 0.14 - / -
ESV03300 (0.008%) 3.2 0.11 - / -
ESV03326 (0.006%) 7.8 0.02 * - / -
ESV03399 (0.003%) 0.7 0.52 N / -
ESV03458 (0.007%) 2.4 0.17 - / -
ESV03482 (0.009%) 1.2 0.36 - / -
ESV03603 (0.005%) 0.7 0.51 - / -
ESV03604 (0.004%) 1.0 0.42 N / -
ESV03633 (0.004%) 2.2 0.19 - / -
ESV03636 (0.01%) 1.4 0.31 - / -
ESV03695 (0.004%) 0.9 0.46 - / -
ESV03766 (0.005%) 0.1 0.90 N / -
ESV03841 (0.006%) 1.1 0.40 - / -
ESV03848 (0.009%) 0.6 0.57 - / -
ESV03915 (0.005%) 0.7 0.52 - / -
ESV03954 (0.005%) 0.3 0.72 - / -
ESV04011 (0.004%) 2.1 0.21 - / -
ESV04038 (0.006%) 3.0 0.13 - / -
ESV04048 (0.005%) 0.3 0.77 - / -
ESV04054 (0.005%) 0.8 0.49 - / -
ESV04062 (0.008%) 0.6 0.59 - / -
ESV04072 (0.006%) 0.6 0.58 - / -
ESV04113 (0.003%) 0.3 0.74 - / -
ESV04117 (0.005%) 0.1 0.94 - / -
ESV04170 (0.007%) 1.9 0.23 - / -

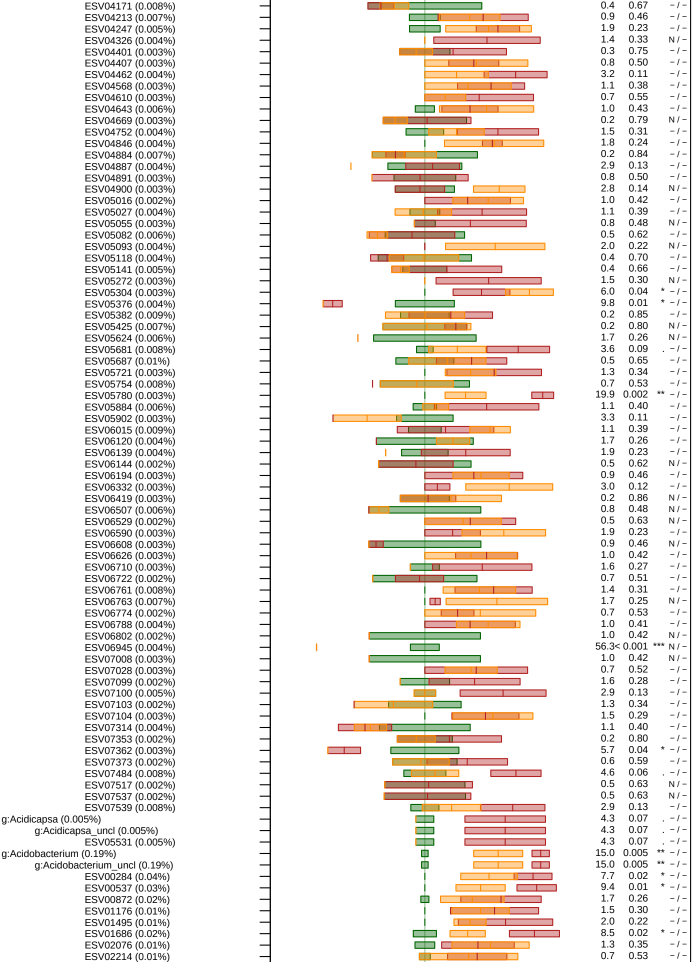

ESV04171 (0.008%) 0.4 0.67 – / –
ESV04213 (0.007%) 0.9 0.46 – / –
ESV04247 (0.005%) 1.9 0.23 – / –
ESV04326 (0.004%) 1.4 0.33 N / –
ESV04401 (0.003%) 0.3 0.75 – / –
ESV04407 (0.003%) 0.8 0.50 – / –
ESV04462 (0.004%) 3.2 0.11 – / –
ESV04568 (0.003%) 1.1 0.38 – / –
ESV04610 (0.003%) 0.7 0.55 – / –
ESV04643 (0.006%) 1.0 0.43 – / –
ESV04669 (0.003%) 0.2 0.79 N / –
ESV04752 (0.004%) 1.5 0.31 – / –
ESV04846 (0.004%) 1.8 0.24 – / –
ESV04884 (0.007%) 0.2 0.84 – / –
ESV04887 (0.004%) 2.9 0.13 – / –
ESV04891 (0.003%) 0.8 0.50 – / –
ESV04900 (0.003%) 2.8 0.14 N / –
ESV05016 (0.002%) 1.0 0.42 – / –
ESV05027 (0.004%) 1.1 0.39 – / –
ESV05055 (0.003%) 0.8 0.48 N / –
ESV05082 (0.006%) 0.5 0.62 – / –
ESV05093 (0.004%) 2.0 0.22 N / –
ESV05118 (0.004%) 0.4 0.70 – / –
ESV05141 (0.005%) 0.4 0.66 – / –
ESV05272 (0.003%) 1.5 0.30 N / –
ESV05304 (0.003%) 6.0 0.04 * – / –
ESV05376 (0.004%) 9.8 0.01 * – / –
ESV05382 (0.009%) 0.2 0.85 – / –
ESV05425 (0.007%) 0.2 0.80 N / –
ESV05624 (0.006%) 1.7 0.26 N / –
ESV05681 (0.008%) 3.6 0.09 . – / –
ESV05687 (0.01%) 0.5 0.65 – / –
ESV05721 (0.003%) 1.3 0.34 – / –
ESV05754 (0.008%) 0.7 0.53 – / –
ESV05780 (0.003%) 19.9 0.002 ** – / –
ESV05884 (0.006%) 1.1 0.40 – / –
ESV05902 (0.003%) 3.3 0.11 – / –
ESV06015 (0.009%) 1.1 0.39 – / –
ESV06120 (0.004%) 1.7 0.26 – / –
ESV06139 (0.004%) 1.9 0.23 – / –
ESV06144 (0.002%) 0.5 0.62 N / –
ESV06194 (0.003%) 0.9 0.46 – / –
ESV06332 (0.003%) 3.0 0.12 – / –
ESV06419 (0.003%) 0.2 0.86 N / –
ESV06507 (0.006%) 0.8 0.48 N / –
ESV06529 (0.002%) 0.5 0.63 N / –
ESV06590 (0.003%) 1.9 0.23 – / –
ESV06608 (0.003%) 0.9 0.46 N / –
ESV06626 (0.003%) 1.0 0.42 – / –
ESV06710 (0.003%) 1.6 0.27 – / –
ESV06722 (0.002%) 0.7 0.51 – / –
ESV06761 (0.008%) 1.4 0.31 – / –
ESV06763 (0.007%) 1.7 0.25 N / –
ESV06774 (0.002%) 0.7 0.53 – / –
ESV06788 (0.004%) 1.0 0.41 – / –
ESV06802 (0.002%) 1.0 0.42 N / –
ESV06945 (0.004%) 56.3 < 0.001 *** N / –
ESV07008 (0.003%) 1.0 0.42 N / –
ESV07028 (0.003%) 0.7 0.52 – / –
ESV07099 (0.002%) 1.6 0.28 – / –
ESV07100 (0.005%) 2.9 0.13 – / –
ESV07103 (0.002%) 1.3 0.34 – / –
ESV07104 (0.003%) 1.5 0.29 – / –
ESV07314 (0.004%) 1.1 0.40 – / –
ESV07353 (0.002%) 0.2 0.80 – / –
ESV07362 (0.003%) 5.7 0.04 * – / –
ESV07373 (0.002%) 0.6 0.59 – / –
ESV07484 (0.008%) 4.6 0.06 . – / –
ESV07517 (0.002%) 0.5 0.63 N / –
ESV07537 (0.002%) 0.5 0.63 N / –
ESV07539 (0.008%) 2.9 0.13 – / –
g:Acidicapsa (0.005%) 4.3 0.07 . – / –
g:Acidicapsa_uncl (0.005%) 4.3 0.07 . – / –
ESV05531 (0.005%) 4.3 0.07 . – / –
g:Acidobacterium (0.19%) 15.0 0.005 ** – / –
g:Acidobacterium_uncl (0.19%) 15.0 0.005 ** – / –
ESV00284 (0.04%) 7.7 0.02 * – / –
ESV00537 (0.03%) 9.4 0.01 * – / –
ESV00872 (0.02%) 1.7 0.26 – / –
ESV01176 (0.01%) 1.5 0.30 – / –
ESV01495 (0.01%) 2.0 0.22 – / –
ESV01686 (0.02%) 8.5 0.02 * – / –
ESV02076 (0.01%) 1.3 0.35 – / –
ESV02214 (0.01%) 0.7 0.53 – / –

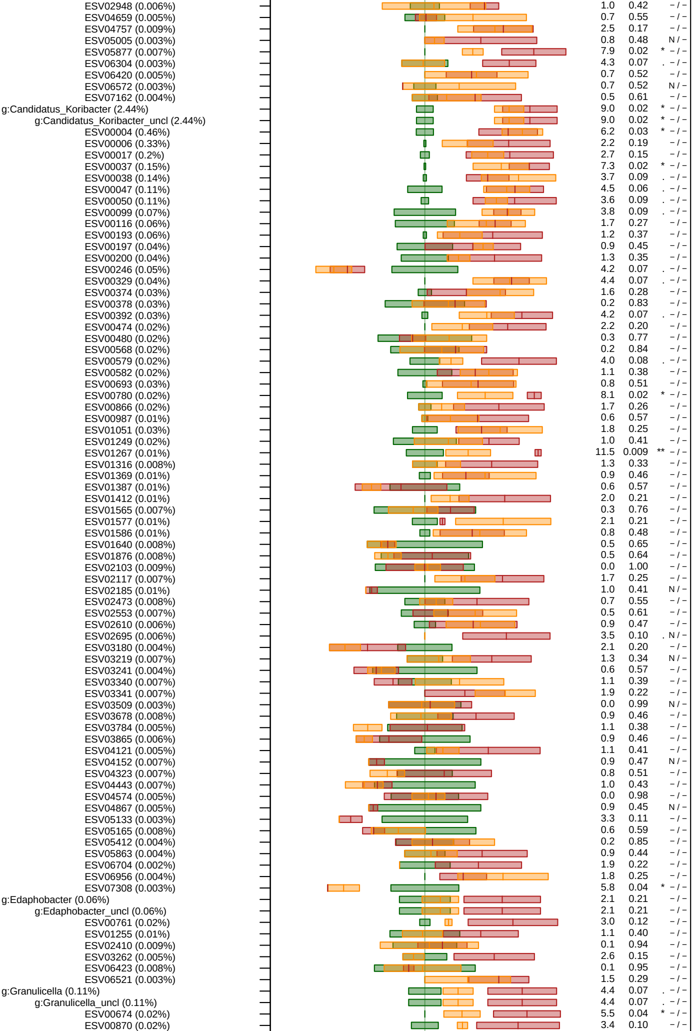
ESV02948 (0.006%) 1.0 0.42 – / –
ESV04659 (0.005%) 0.7 0.55 – / –
ESV04757 (0.009%) 2.5 0.17 – / –
ESV05005 (0.003%) 0.8 0.48 N / –
ESV05877 (0.007%) 7.9 0.02 * – / –
ESV06304 (0.003%) 4.3 0.07 . – / –
ESV06420 (0.005%) 0.7 0.52 – / –
ESV06572 (0.003%) 0.7 0.52 N / –
ESV07162 (0.004%) 0.5 0.61 – / –
g:Candidatus_Koribacter (2.44%) 9.0 0.02 * – / –
g:Candidatus_Koribacter_uncl (2.44%) 9.0 0.02 * – / –
ESV00004 (0.46%) 6.2 0.03 * – / –
ESV00006 (0.33%) 2.2 0.19 – / –
ESV00017 (0.2%) 2.7 0.15 – / –
ESV00037 (0.15%) 7.3 0.02 * – / –
ESV00038 (0.14%) 3.7 0.09 . – / –
ESV00047 (0.11%) 4.5 0.06 . – / –
ESV00050 (0.11%) 3.6 0.09 . – / –
ESV00099 (0.07%) 3.8 0.09 . – / –
ESV00116 (0.06%) 1.7 0.27 – / –
ESV00193 (0.06%) 1.2 0.37 – / –
ESV00197 (0.04%) 0.9 0.45 – / –
ESV00200 (0.04%) 1.3 0.35 – / –
ESV00246 (0.05%) 4.2 0.07 . – / –
ESV00329 (0.04%) 4.4 0.07 . – / –
ESV00374 (0.03%) 1.6 0.28 – / –
ESV00378 (0.03%) 0.2 0.83 – / –
ESV00392 (0.03%) 4.2 0.07 . – / –
ESV00474 (0.02%) 2.2 0.20 – / –
ESV00480 (0.02%) 0.3 0.77 – / –
ESV00568 (0.02%) 0.2 0.84 – / –
ESV00579 (0.02%) 4.0 0.08 . – / –
ESV00582 (0.02%) 1.1 0.38 – / –
ESV00693 (0.03%) 0.8 0.51 – / –
ESV00780 (0.02%) 8.1 0.02 * – / –
ESV00866 (0.02%) 1.7 0.26 – / –
ESV00987 (0.01%) 0.6 0.57 – / –
ESV01051 (0.03%) 1.8 0.25 – / –
ESV01249 (0.02%) 1.0 0.41 – / –
ESV01267 (0.01%) 11.5 0.009 ** – / –
ESV01316 (0.008%) 1.3 0.33 – / –
ESV01369 (0.01%) 0.9 0.46 – / –
ESV01387 (0.01%) 0.6 0.57 – / –
ESV01412 (0.01%) 2.0 0.21 – / –
ESV01565 (0.007%) 0.3 0.76 – / –
ESV01577 (0.01%) 2.1 0.21 – / –
ESV01586 (0.01%) 0.8 0.48 – / –
ESV01640 (0.008%) 0.5 0.65 – / –
ESV01876 (0.008%) 0.5 0.64 – / –
ESV02103 (0.009%) 0.0 1.00 – / –
ESV02117 (0.007%) 1.7 0.25 – / –
ESV02185 (0.01%) 1.0 0.41 N / –
ESV02473 (0.008%) 0.7 0.55 – / –
ESV02553 (0.007%) 0.5 0.61 – / –
ESV02610 (0.006%) 0.9 0.47 – / –
ESV02695 (0.006%) 3.5 0.10 . N / –
ESV03180 (0.004%) 2.1 0.20 – / –
ESV03219 (0.007%) 1.3 0.34 N / –
ESV03241 (0.004%) 0.6 0.57 – / –
ESV03340 (0.007%) 1.1 0.39 – / –
ESV03341 (0.007%) 1.9 0.22 – / –
ESV03509 (0.003%) 0.0 0.99 N / –
ESV03678 (0.008%) 0.9 0.46 – / –
ESV03784 (0.005%) 1.1 0.38 – / –
ESV03865 (0.006%) 0.9 0.46 – / –
ESV04121 (0.005%) 1.1 0.41 – / –
ESV04152 (0.007%) 0.9 0.47 N / –
ESV04323 (0.007%) 0.8 0.51 – / –
ESV04443 (0.007%) 1.0 0.43 – / –
ESV04574 (0.005%) 0.0 0.98 – / –
ESV04867 (0.005%) 0.9 0.45 N / –
ESV05133 (0.003%) 3.3 0.11 – / –
ESV05165 (0.008%) 0.6 0.59 – / –
ESV05412 (0.004%) 0.2 0.85 – / –
ESV05863 (0.004%) 0.9 0.44 – / –
ESV06704 (0.002%) 1.9 0.22 – / –
ESV06956 (0.004%) 1.8 0.25 – / –
ESV07308 (0.003%) 5.8 0.04 * – / –
g:Edaphobacter (0.06%) 2.1 0.21 – / –
g:Edaphobacter_uncl (0.06%) 2.1 0.21 – / –
ESV00761 (0.02%) 3.0 0.12 – / –
ESV01255 (0.01%) 1.1 0.40 – / –
ESV02410 (0.009%) 0.1 0.94 – / –
ESV03262 (0.005%) 2.6 0.15 – / –
ESV06423 (0.008%) 0.1 0.95 – / –
ESV06521 (0.003%) 1.5 0.29 – / –
g:Granulicella (0.11%) 4.4 0.07 . – / –
g:Granulicella_uncl (0.11%) 4.4 0.07 . – / –
ESV00674 (0.02%) 5.5 0.04 * – / –
ESV00870 (0.02%) 3.4 0.10 – / –

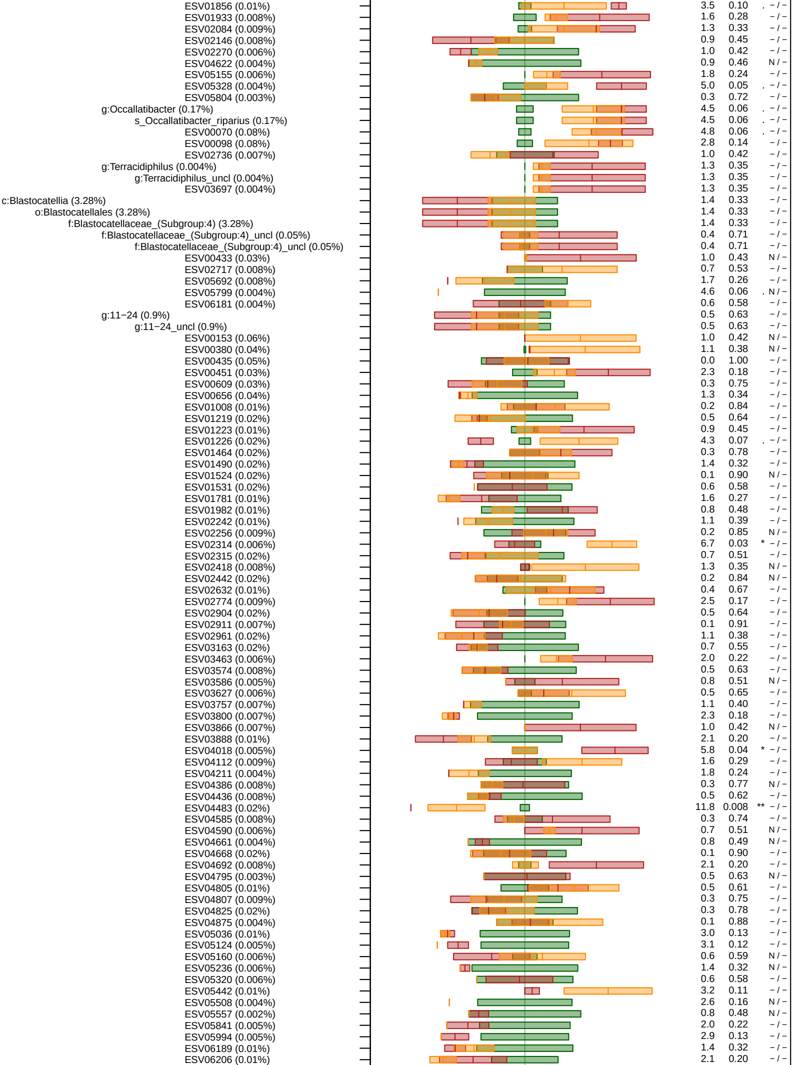

ESV01856 (0.01%) 3.5 0.10 . - / -
ESV01933 (0.008%) 1.6 0.28 - / -
ESV02084 (0.009%) 1.3 0.33 - / -
ESV02146 (0.008%) 0.9 0.45 - / -
ESV02270 (0.006%) 1.0 0.42 - / -
ESV04622 (0.004%) 0.9 0.46 N / -
ESV05155 (0.006%) 1.8 0.24 - / -
ESV05328 (0.004%) 5.0 0.05 . - / -
ESV05804 (0.003%) 0.3 0.72 - / -
g:Occallatibacter (0.17%) 4.5 0.06 . - / -
s_Occallatibacter_riparius (0.17%) 4.5 0.06 . - / -
ESV00070 (0.08%) 4.8 0.06 . - / -
ESV00098 (0.08%) 2.8 0.14 - / -
ESV02736 (0.007%) 1.0 0.42 - / -
g:Terracidiphilus (0.004%) 1.3 0.35 - / -
g:Terracidiphilus_uncl (0.004%) 1.3 0.35 - / -
ESV03697 (0.004%) 1.3 0.35 - / -
c:Blastocatellia (3.28%) 1.4 0.33 - / -
o:Blastocatellales (3.28%) 1.4 0.33 - / -
f:Blastocatellaceae_(Subgroup:4) (3.28%) 1.4 0.33 - / -
f:Blastocatellaceae_(Subgroup:4)_uncl (0.05%) 0.4 0.71 - / -
f:Blastocatellaceae_(Subgroup:4)_uncl (0.05%) 0.4 0.71 - / -
ESV00433 (0.03%) 1.0 0.43 N / -
ESV02717 (0.008%) 0.7 0.53 - / -
ESV05692 (0.008%) 1.7 0.26 - / -
ESV05799 (0.004%) 4.6 0.06 . N / -
ESV06181 (0.004%) 0.6 0.58 - / -
g:11−24 (0.9%) 0.5 0.63 - / -
g:11−24_uncl (0.9%) 0.5 0.63 - / -
ESV00153 (0.06%) 1.0 0.42 N / -
ESV00380 (0.04%) 1.1 0.38 N / -
ESV00435 (0.05%) 0.0 1.00 - / -
ESV00451 (0.03%) 2.3 0.18 - / -
ESV00609 (0.03%) 0.3 0.75 - / -
ESV00656 (0.04%) 1.3 0.34 - / -
ESV01008 (0.01%) 0.2 0.84 - / -
ESV01219 (0.02%) 0.5 0.64 - / -
ESV01223 (0.01%) 0.9 0.45 - / -
ESV01226 (0.02%) 4.3 0.07 . - / -
ESV01464 (0.02%) 0.3 0.78 - / -
ESV01490 (0.02%) 1.4 0.32 - / -
ESV01524 (0.02%) 0.1 0.90 N / -
ESV01531 (0.02%) 0.6 0.58 - / -
ESV01781 (0.01%) 1.6 0.27 - / -
ESV01982 (0.01%) 0.8 0.48 - / -
ESV02242 (0.01%) 1.1 0.39 - / -
ESV02256 (0.009%) 0.2 0.85 N / -
ESV02314 (0.006%) 6.7 0.03 * - / -
ESV02315 (0.02%) 0.7 0.51 - / -
ESV02418 (0.008%) 1.3 0.35 N / -
ESV02442 (0.02%) 0.2 0.84 N / -
ESV02632 (0.01%) 0.4 0.67 - / -
ESV02774 (0.009%) 2.5 0.17 - / -
ESV02904 (0.02%) 0.5 0.64 - / -
ESV02911 (0.007%) 0.1 0.91 - / -
ESV02961 (0.02%) 1.1 0.38 - / -
ESV03163 (0.02%) 0.7 0.55 - / -
ESV03463 (0.006%) 2.0 0.22 - / -
ESV03574 (0.008%) 0.5 0.63 - / -
ESV03586 (0.005%) 0.8 0.51 N / -
ESV03627 (0.006%) 0.5 0.65 - / -
ESV03757 (0.007%) 1.1 0.40 - / -
ESV03800 (0.007%) 2.3 0.18 - / -
ESV03866 (0.007%) 1.0 0.42 N / -
ESV03888 (0.01%) 2.1 0.20 - / -
ESV04018 (0.005%) 5.8 0.04 * - / -
ESV04112 (0.009%) 1.6 0.29 - / -
ESV04211 (0.004%) 1.8 0.24 - / -
ESV04386 (0.008%) 0.3 0.77 N / -
ESV04436 (0.008%) 0.5 0.62 - / -
ESV04483 (0.02%) 11.8 0.008 ** - / -
ESV04585 (0.008%) 0.3 0.74 - / -
ESV04590 (0.006%) 0.7 0.51 N / -
ESV04661 (0.004%) 0.8 0.49 N / -
ESV04668 (0.02%) 0.1 0.90 - / -
ESV04692 (0.008%) 2.1 0.20 - / -
ESV04795 (0.003%) 0.5 0.63 N / -
ESV04805 (0.01%) 0.5 0.61 - / -
ESV04807 (0.009%) 0.3 0.75 - / -
ESV04825 (0.02%) 0.3 0.78 - / -
ESV04875 (0.004%) 0.1 0.88 - / -
ESV05036 (0.01%) 3.0 0.13 - / -
ESV05124 (0.005%) 3.1 0.12 - / -
ESV05160 (0.006%) 0.6 0.59 N / -
ESV05236 (0.006%) 1.4 0.32 N / -
ESV05320 (0.006%) 0.6 0.58 - / -
ESV05442 (0.01%) 3.2 0.11 - / -
ESV05508 (0.004%) 2.6 0.16 N / -
ESV05557 (0.002%) 0.8 0.48 N / -
ESV05841 (0.005%) 2.0 0.22 - / -
ESV05994 (0.005%) 2.9 0.13 - / -
ESV06189 (0.01%) 1.4 0.32 - / -
ESV06206 (0.01%) 2.1 0.20 - / -

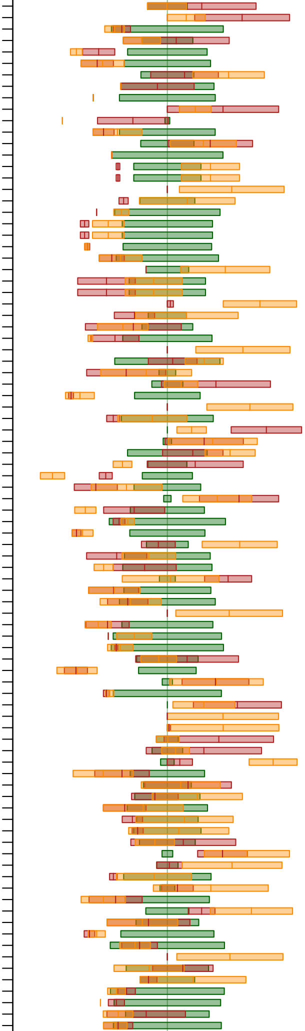

ESV06224 (0.002%)	0.3	0.74		– / –
ESV06326 (0.002%)	1.7	0.25		– / –
ESV06431 (0.009%)	0.7	0.52		– / –
ESV06447 (0.005%)	0.3	0.79		– / –
ESV06588 (0.01%)	3.6	0.09	.	– / –
ESV06706 (0.003%)	1.8	0.25		– / –
ESV06738 (0.01%)	1.0	0.44		– / –
ESV06928 (0.004%)	0.5	0.62		N / –
ESV07073 (0.006%)	2.4	0.17		N / –
ESV07076 (0.004%)	0.7	0.54		– / –
ESV07158 (0.007%)	6.8	0.03	*	N / –
ESV07285 (0.004%)	1.1	0.38		– / –
ESV07347 (0.008%)	0.4	0.66		– / –
ESV07531 (0.002%)	1.0	0.42		N / –
g:Aridibacter (0.04%)	3.2	0.11		– / –
g:Aridibacter_uncl (0.04%)	3.2	0.11		– / –
ESV01309 (0.01%)	1.5	0.29		N / –
ESV02399 (0.01%)	1.0	0.41		– / –
ESV02721 (0.01%)	1.4	0.32		– / –
g:Blastocatella (0.03%)	2.4	0.17		– / –
g:Blastocatella_uncl (0.03%)	2.4	0.17		– / –
ESV06392 (0.02%)	3.3	0.11		N / –
ESV06567 (0.004%)	0.8	0.48		– / –
ESV06882 (0.007%)	2.1	0.21		– / –
g:RB41 (1.98%)	1.0	0.43		– / –
g:RB41_uncl (1.98%)	1.0	0.43		– / –
ESV00237 (0.07%)	6.1	0.04	*	N / –
ESV00335 (0.1%)	0.6	0.58		– / –
ESV00370 (0.05%)	0.5	0.66		– / –
ESV00389 (0.04%)	1.8	0.24		– / –
ESV00417 (0.03%)	2.6	0.16		N / –
ESV00458 (0.05%)	0.3	0.74		– / –
ESV00626 (0.07%)	0.3	0.72		N / –
ESV00651 (0.05%)	0.5	0.61		– / –
ESV00686 (0.06%)	6.6	0.03	*	– / –
ESV00713 (0.03%)	3.7	0.09	.	N / –
ESV00792 (0.03%)	0.7	0.53		– / –
ESV00813 (0.02%)	5.5	0.04	*	– / –
ESV00818 (0.03%)	0.5	0.63		– / –
ESV01078 (0.02%)	1.0	0.44		– / –
ESV01160 (0.02%)	1.5	0.30		– / –
ESV01165 (0.03%)	12.1	0.008	**	– / –
ESV01245 (0.02%)	1.4	0.32		– / –
ESV01273 (0.02%)	1.5	0.30		– / –
ESV01394 (0.03%)	2.1	0.20		– / –
ESV01434 (0.01%)	0.6	0.59		N / –
ESV01508 (0.04%)	5.0	0.05	.	– / –
ESV01603 (0.02%)	2.8	0.14		– / –
ESV01611 (0.03%)	0.6	0.59		– / –
ESV01678 (0.02%)	1.0	0.42		– / –
ESV01797 (0.02%)	1.2	0.37		– / –
ESV01799 (0.02%)	0.9	0.46		– / –
ESV01957 (0.02%)	0.4	0.68		– / –
ESV01958 (0.009%)	1.3	0.33		N / –
ESV02015 (0.02%)	1.6	0.28		– / –
ESV02027 (0.02%)	0.8	0.49		– / –
ESV02032 (0.02%)	0.7	0.52		– / –
ESV02073 (0.01%)	0.2	0.85		– / –
ESV02129 (0.01%)	6.0	0.04	*	– / –
ESV02204 (0.01%)	0.7	0.53		– / –
ESV02237 (0.02%)	1.2	0.37		N / –
ESV02387 (0.01%)	1.3	0.35		– / –
ESV02408 (0.009%)	1.0	0.42		N / –
ESV02454 (0.01%)	1.0	0.43		N / –
ESV02460 (0.007%)	0.8	0.49		– / –
ESV02485 (0.01%)	0.3	0.76		– / –
ESV02531 (0.01%)	12.8	0.007	**	– / –
ESV02541 (0.008%)	1.1	0.40		– / –
ESV02565 (0.01%)	0.1	0.92		– / –
ESV02580 (0.01%)	0.4	0.69		– / –
ESV02678 (0.01%)	0.4	0.70		N / –
ESV02730 (0.01%)	0.6	0.57		– / –
ESV02756 (0.01%)	0.4	0.71		– / –
ESV02772 (0.007%)	0.2	0.86		– / –
ESV02812 (0.01%)	2.0	0.21		– / –
ESV02851 (0.009%)	1.5	0.30		– / –
ESV02916 (0.01%)	0.7	0.53		N / –
ESV02954 (0.008%)	0.4	0.67		– / –
ESV02963 (0.006%)	1.2	0.37		– / –
ESV02993 (0.008%)	2.3	0.18		– / –
ESV03049 (0.01%)	0.1	0.88		N / –
ESV03144 (0.02%)	2.4	0.17		– / –
ESV03157 (0.03%)	0.2	0.79		– / –
ESV03166 (0.004%)	1.4	0.32		N / –
ESV03190 (0.008%)	0.2	0.80		N / –
ESV03246 (0.008%)	0.4	0.68		– / –
ESV03332 (0.03%)	0.6	0.56		– / –
ESV03380 (0.02%)	1.3	0.35		N / –
ESV03452 (0.02%)	0.5	0.62		– / –
ESV03484 (0.007%)	0.8	0.50		– / –

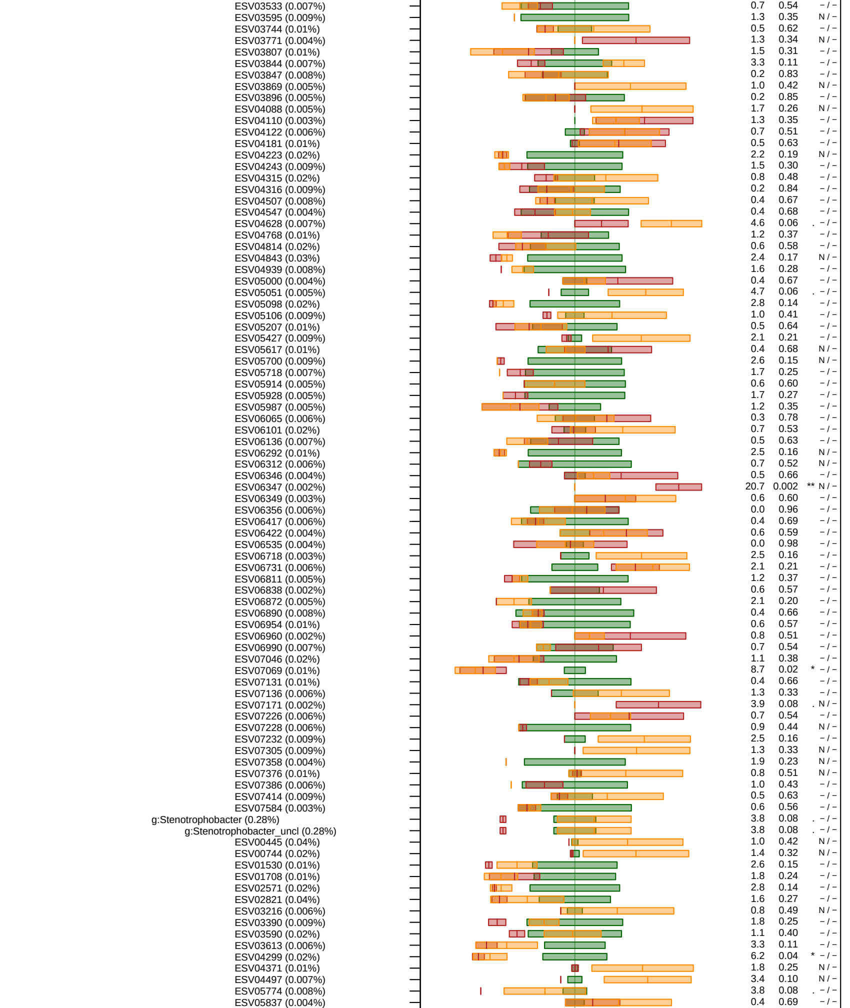
ESV03533 (0.007%) 0.7 0.54 – / –
ESV03595 (0.009%) 1.3 0.35 N / –
ESV03744 (0.01%) 0.5 0.62 – / –
ESV03771 (0.004%) 1.3 0.34 N / –
ESV03807 (0.01%) 1.5 0.31 – / –
ESV03844 (0.007%) 3.3 0.11 – / –
ESV03847 (0.008%) 0.2 0.83 – / –
ESV03869 (0.005%) 1.0 0.42 N / –
ESV03896 (0.005%) 0.2 0.85 – / –
ESV04088 (0.005%) 1.7 0.26 N / –
ESV04110 (0.003%) 1.3 0.35 – / –
ESV04122 (0.006%) 0.7 0.51 – / –
ESV04181 (0.01%) 0.5 0.63 – / –
ESV04223 (0.02%) 2.2 0.19 N / –
ESV04243 (0.009%) 1.5 0.30 – / –
ESV04315 (0.02%) 0.8 0.48 – / –
ESV04316 (0.009%) 0.2 0.84 – / –
ESV04507 (0.008%) 0.4 0.67 – / –
ESV04547 (0.004%) 0.4 0.68 – / –
ESV04628 (0.007%) 4.6 0.06 . – / –
ESV04768 (0.01%) 1.2 0.37 – / –
ESV04814 (0.02%) 0.6 0.58 – / –
ESV04843 (0.03%) 2.4 0.17 N / –
ESV04939 (0.008%) 1.6 0.28 – / –
ESV05000 (0.004%) 0.4 0.67 – / –
ESV05051 (0.005%) 4.7 0.06 . – / –
ESV05098 (0.02%) 2.8 0.14 – / –
ESV05106 (0.009%) 1.0 0.41 – / –
ESV05207 (0.01%) 0.5 0.64 – / –
ESV05427 (0.009%) 2.1 0.21 – / –
ESV05617 (0.01%) 0.4 0.68 N / –
ESV05700 (0.009%) 2.6 0.15 N / –
ESV05718 (0.007%) 1.7 0.25 – / –
ESV05914 (0.005%) 0.6 0.60 – / –
ESV05928 (0.005%) 1.7 0.27 – / –
ESV05987 (0.005%) 1.2 0.35 – / –
ESV06065 (0.006%) 0.3 0.78 – / –
ESV06101 (0.02%) 0.7 0.53 – / –
ESV06136 (0.007%) 0.5 0.63 – / –
ESV06292 (0.01%) 2.5 0.16 N / –
ESV06312 (0.006%) 0.7 0.52 N / –
ESV06346 (0.004%) 0.5 0.66 – / –
ESV06347 (0.002%) 20.7 0.002 ** N / –
ESV06349 (0.003%) 0.6 0.60 – / –
ESV06356 (0.006%) 0.0 0.96 – / –
ESV06417 (0.006%) 0.4 0.69 – / –
ESV06422 (0.004%) 0.6 0.59 – / –
ESV06535 (0.004%) 0.0 0.98 – / –
ESV06718 (0.003%) 2.5 0.16 – / –
ESV06731 (0.006%) 2.1 0.21 – / –
ESV06811 (0.005%) 1.2 0.37 – / –
ESV06838 (0.002%) 0.6 0.57 – / –
ESV06872 (0.005%) 2.1 0.20 – / –
ESV06890 (0.008%) 0.4 0.66 – / –
ESV06954 (0.01%) 0.6 0.57 – / –
ESV06960 (0.002%) 0.8 0.51 – / –
ESV06990 (0.007%) 0.7 0.54 – / –
ESV07046 (0.02%) 1.1 0.38 – / –
ESV07069 (0.01%) 8.7 0.02 * – / –
ESV07131 (0.01%) 0.4 0.66 – / –
ESV07136 (0.006%) 1.3 0.33 – / –
ESV07171 (0.002%) 3.9 0.08 . N / –
ESV07226 (0.006%) 0.7 0.54 – / –
ESV07228 (0.006%) 0.9 0.44 N / –
ESV07232 (0.009%) 2.5 0.16 – / –
ESV07305 (0.009%) 1.3 0.33 N / –
ESV07358 (0.004%) 1.9 0.23 N / –
ESV07376 (0.01%) 0.8 0.51 N / –
ESV07386 (0.006%) 1.0 0.43 – / –
ESV07414 (0.009%) 0.5 0.63 – / –
ESV07584 (0.003%) 0.6 0.56 – / –
g:Stenotrophobacter (0.28%) 3.8 0.08 . – / –
g:Stenotrophobacter_uncl (0.28%) 3.8 0.08 . – / –
ESV00445 (0.04%) 1.0 0.42 N / –
ESV00744 (0.02%) 1.4 0.32 N / –
ESV01530 (0.01%) 2.6 0.15 – / –
ESV01708 (0.01%) 1.8 0.24 – / –
ESV02571 (0.02%) 2.8 0.14 – / –
ESV02821 (0.04%) 1.6 0.27 – / –
ESV03216 (0.006%) 0.8 0.49 N / –
ESV03390 (0.009%) 1.8 0.25 – / –
ESV03590 (0.02%) 1.1 0.40 – / –
ESV03613 (0.006%) 3.3 0.11 – / –
ESV04299 (0.02%) 6.2 0.04 * – / –
ESV04371 (0.01%) 1.8 0.25 N / –
ESV04497 (0.007%) 3.4 0.10 N / –
ESV05774 (0.008%) 3.8 0.08 . – / –
ESV05837 (0.004%) 0.4 0.69 – / –

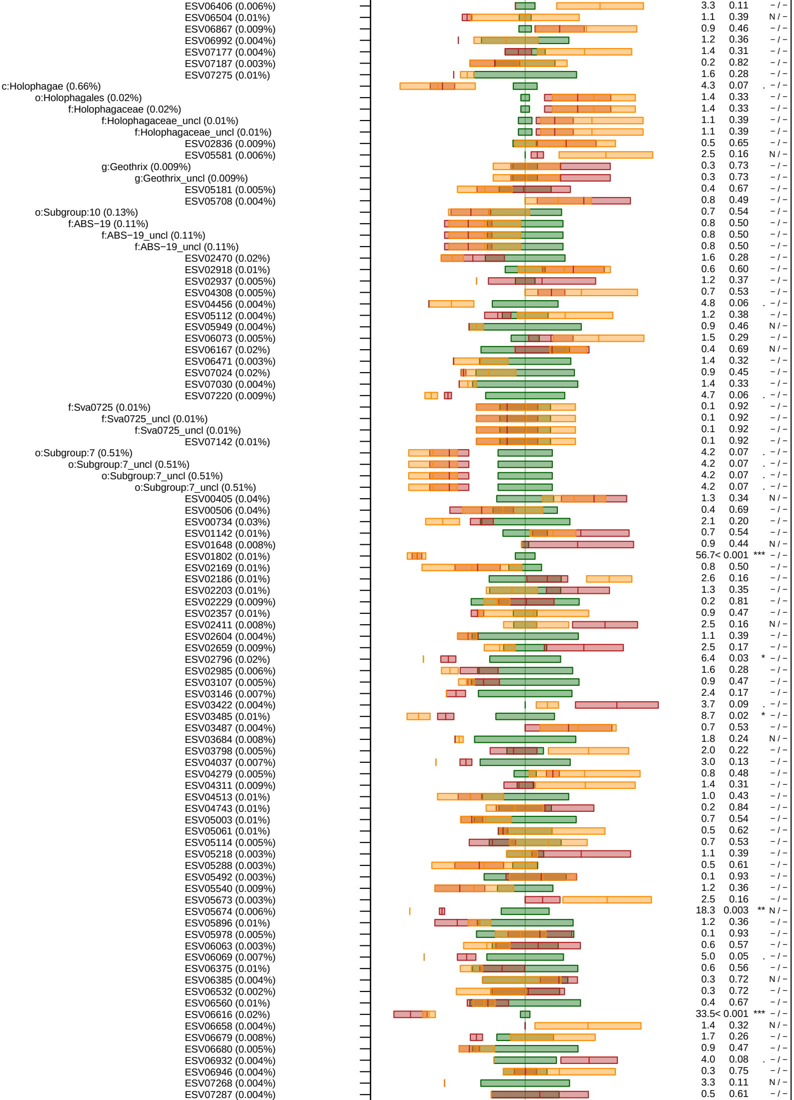

ESV06406 (0.006%) 3.3 0.11 − / −
ESV06504 (0.01%) 1.1 0.39 N / −
ESV06867 (0.009%) 0.9 0.46 − / −
ESV06992 (0.004%) 1.2 0.36 − / −
ESV07177 (0.004%) 1.4 0.31 − / −
ESV07187 (0.003%) 0.2 0.82 − / −
ESV07275 (0.01%) 1.6 0.28 − / −
c:Holophagae (0.66%) 4.3 0.07 . − / −
o:Holophagales (0.02%) 1.4 0.33 − / −
f:Holophagaceae (0.02%) 1.4 0.33 − / −
f:Holophagaceae_uncl (0.01%) 1.1 0.39 − / −
f:Holophagaceae_uncl (0.01%) 1.1 0.39 − / −
ESV02836 (0.009%) 0.5 0.65 − / −
ESV05581 (0.006%) 2.5 0.16 N / −
g:Geothrix (0.009%) 0.3 0.73 − / −
g:Geothrix_uncl (0.009%) 0.3 0.73 − / −
ESV05181 (0.005%) 0.4 0.67 − / −
ESV05708 (0.004%) 0.8 0.49 − / −
o:Subgroup:10 (0.13%) 0.7 0.54 − / −
f:ABS−19 (0.11%) 0.8 0.50 − / −
f:ABS−19_uncl (0.11%) 0.8 0.50 − / −
f:ABS−19_uncl (0.11%) 0.8 0.50 − / −
ESV02470 (0.02%) 1.6 0.28 − / −
ESV02918 (0.01%) 0.6 0.60 − / −
ESV02937 (0.005%) 1.2 0.37 − / −
ESV04308 (0.005%) 0.7 0.53 − / −
ESV04456 (0.004%) 4.8 0.06 . − / −
ESV05112 (0.004%) 1.2 0.38 − / −
ESV05949 (0.004%) 0.9 0.46 N / −
ESV06073 (0.005%) 1.5 0.29 − / −
ESV06167 (0.02%) 0.4 0.69 N / −
ESV06471 (0.003%) 1.4 0.32 − / −
ESV07024 (0.02%) 0.9 0.45 − / −
ESV07030 (0.004%) 1.4 0.33 − / −
ESV07220 (0.009%) 4.7 0.06 . − / −
f:Sva0725 (0.01%) 0.1 0.92 − / −
f:Sva0725_uncl (0.01%) 0.1 0.92 − / −
f:Sva0725_uncl (0.01%) 0.1 0.92 − / −
ESV07142 (0.01%) 0.1 0.92 − / −
o:Subgroup:7 (0.51%) 4.2 0.07 . − / −
o:Subgroup:7_uncl (0.51%) 4.2 0.07 . − / −
o:Subgroup:7_uncl (0.51%) 4.2 0.07 . − / −
o:Subgroup:7_uncl (0.51%) 4.2 0.07 . − / −
ESV00405 (0.04%) 1.3 0.34 N / −
ESV00506 (0.04%) 0.4 0.69 − / −
ESV00734 (0.03%) 2.1 0.20 − / −
ESV01142 (0.01%) 0.7 0.54 − / −
ESV01648 (0.008%) 0.9 0.44 N / −
ESV01802 (0.01%) 56.7< 0.001 *** − / −
ESV02169 (0.01%) 0.8 0.50 − / −
ESV02186 (0.01%) 2.6 0.16 − / −
ESV02203 (0.01%) 1.3 0.35 − / −
ESV02229 (0.009%) 0.2 0.81 − / −
ESV02357 (0.01%) 0.9 0.47 − / −
ESV02411 (0.008%) 2.5 0.16 N / −
ESV02604 (0.004%) 1.1 0.39 − / −
ESV02659 (0.009%) 2.5 0.17 − / −
ESV02796 (0.02%) 6.4 0.03 * − / −
ESV02985 (0.006%) 1.6 0.28 − / −
ESV03107 (0.005%) 0.9 0.47 − / −
ESV03146 (0.007%) 2.4 0.17 − / −
ESV03422 (0.004%) 3.7 0.09 . − / −
ESV03485 (0.01%) 8.7 0.02 * − / −
ESV03487 (0.004%) 0.7 0.53 − / −
ESV03684 (0.008%) 1.8 0.24 N / −
ESV03798 (0.005%) 2.0 0.22 − / −
ESV04037 (0.007%) 3.0 0.13 − / −
ESV04279 (0.005%) 0.8 0.48 − / −
ESV04311 (0.009%) 1.4 0.31 − / −
ESV04513 (0.01%) 1.0 0.43 − / −
ESV04743 (0.01%) 0.2 0.84 − / −
ESV05003 (0.01%) 0.7 0.54 − / −
ESV05061 (0.01%) 0.5 0.62 − / −
ESV05114 (0.005%) 0.7 0.53 − / −
ESV05218 (0.003%) 1.1 0.39 − / −
ESV05288 (0.003%) 0.5 0.61 − / −
ESV05492 (0.003%) 0.1 0.93 − / −
ESV05540 (0.009%) 1.2 0.36 − / −
ESV05673 (0.003%) 2.5 0.16 − / −
ESV05674 (0.006%) 18.3 0.003 ** N / −
ESV05896 (0.01%) 1.2 0.36 − / −
ESV05978 (0.005%) 0.1 0.93 − / −
ESV06063 (0.003%) 0.6 0.57 − / −
ESV06069 (0.007%) 5.0 0.05 . − / −
ESV06375 (0.01%) 0.6 0.56 − / −
ESV06385 (0.004%) 0.3 0.72 N / −
ESV06532 (0.002%) 0.3 0.72 − / −
ESV06560 (0.01%) 0.4 0.67 − / −
ESV06616 (0.02%) 33.5< 0.001 *** − / −
ESV06658 (0.004%) 1.4 0.32 N / −
ESV06679 (0.008%) 1.7 0.26 − / −
ESV06680 (0.005%) 0.9 0.47 − / −
ESV06932 (0.004%) 4.0 0.08 . − / −
ESV06946 (0.004%) 0.3 0.75 − / −
ESV07268 (0.004%) 3.3 0.11 N / −
ESV07287 (0.004%) 0.5 0.61 − / −

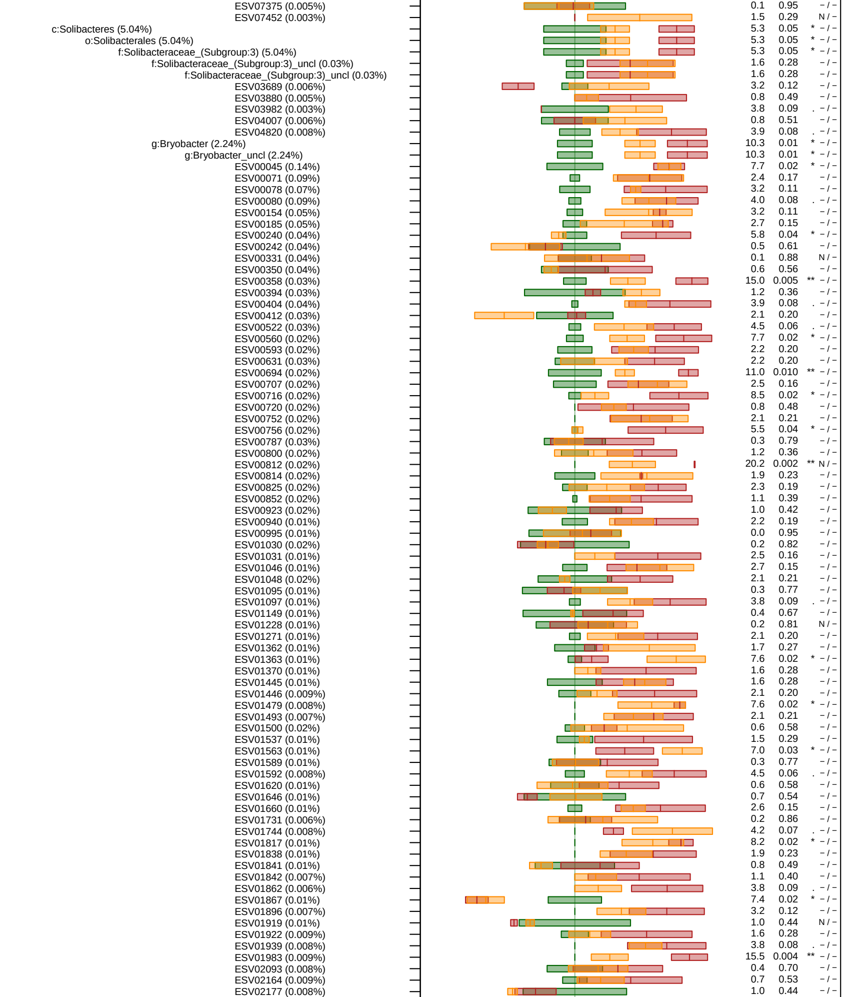
ESV07375 (0.005%) 0.1 0.95 – / –
ESV07452 (0.003%) 1.5 0.29 N / –
c:Solibacteres (5.04%) 5.3 0.05 * – / –
o:Solibacterales (5.04%) 5.3 0.05 * – / –
f:Solibacteraceae_(Subgroup:3) (5.04%) 5.3 0.05 * – / –
f:Solibacteraceae_(Subgroup:3)_uncl (0.03%) 1.6 0.28 – / –
f:Solibacteraceae_(Subgroup:3)_uncl (0.03%) 1.6 0.28 – / –
ESV03689 (0.006%) 3.2 0.12 – / –
ESV03880 (0.005%) 0.8 0.49 – / –
ESV03982 (0.003%) 3.8 0.09 . – / –
ESV04007 (0.006%) 0.8 0.51 – / –
ESV04820 (0.008%) 3.9 0.08 . – / –
g:Bryobacter (2.24%) 10.3 0.01 * – / –
g:Bryobacter_uncl (2.24%) 10.3 0.01 * – / –
ESV00045 (0.14%) 7.7 0.02 * – / –
ESV00071 (0.09%) 2.4 0.17 – / –
ESV00078 (0.07%) 3.2 0.11 – / –
ESV00080 (0.09%) 4.0 0.08 . – / –
ESV00154 (0.05%) 3.2 0.11 – / –
ESV00185 (0.05%) 2.7 0.15 – / –
ESV00240 (0.04%) 5.8 0.04 * – / –
ESV00242 (0.04%) 0.5 0.61 – / –
ESV00331 (0.04%) 0.1 0.88 N / –
ESV00350 (0.04%) 0.6 0.56 – / –
ESV00358 (0.03%) 15.0 0.005 ** – / –
ESV00394 (0.03%) 1.2 0.36 – / –
ESV00404 (0.04%) 3.9 0.08 . – / –
ESV00412 (0.03%) 2.1 0.20 – / –
ESV00522 (0.03%) 4.5 0.06 . – / –
ESV00560 (0.02%) 7.7 0.02 * – / –
ESV00593 (0.02%) 2.2 0.20 – / –
ESV00631 (0.03%) 2.2 0.20 – / –
ESV00694 (0.02%) 11.0 0.010 ** – / –
ESV00707 (0.02%) 2.5 0.16 – / –
ESV00716 (0.02%) 8.5 0.02 * – / –
ESV00720 (0.02%) 0.8 0.48 – / –
ESV00752 (0.02%) 2.1 0.21 – / –
ESV00756 (0.02%) 5.5 0.04 * – / –
ESV00787 (0.03%) 0.3 0.79 – / –
ESV00800 (0.02%) 1.2 0.36 – / –
ESV00812 (0.02%) 20.2 0.002 ** N / –
ESV00814 (0.02%) 1.9 0.23 – / –
ESV00825 (0.02%) 2.3 0.19 – / –
ESV00852 (0.02%) 1.1 0.39 – / –
ESV00923 (0.02%) 1.0 0.42 – / –
ESV00940 (0.01%) 2.2 0.19 – / –
ESV00995 (0.01%) 0.0 0.95 – / –
ESV01030 (0.02%) 0.2 0.82 – / –
ESV01031 (0.01%) 2.5 0.16 – / –
ESV01046 (0.01%) 2.7 0.15 – / –
ESV01048 (0.02%) 2.1 0.21 – / –
ESV01095 (0.01%) 0.3 0.77 – / –
ESV01097 (0.01%) 3.8 0.09 . – / –
ESV01149 (0.01%) 0.4 0.67 – / –
ESV01228 (0.01%) 0.2 0.81 N / –
ESV01271 (0.01%) 2.1 0.20 – / –
ESV01362 (0.01%) 1.7 0.27 – / –
ESV01363 (0.01%) 7.6 0.02 * – / –
ESV01370 (0.01%) 1.6 0.28 – / –
ESV01445 (0.01%) 1.6 0.28 – / –
ESV01446 (0.009%) 2.1 0.20 – / –
ESV01479 (0.008%) 7.6 0.02 * – / –
ESV01493 (0.007%) 2.1 0.21 – / –
ESV01500 (0.02%) 0.6 0.58 – / –
ESV01537 (0.01%) 1.5 0.29 – / –
ESV01563 (0.01%) 7.0 0.03 * – / –
ESV01589 (0.01%) 0.3 0.77 – / –
ESV01592 (0.008%) 4.5 0.06 . – / –
ESV01620 (0.01%) 0.6 0.58 – / –
ESV01646 (0.01%) 0.7 0.54 – / –
ESV01660 (0.01%) 2.6 0.15 – / –
ESV01731 (0.006%) 0.2 0.86 – / –
ESV01744 (0.008%) 4.2 0.07 . – / –
ESV01817 (0.01%) 8.2 0.02 * – / –
ESV01838 (0.01%) 1.9 0.23 – / –
ESV01841 (0.01%) 0.8 0.49 – / –
ESV01842 (0.007%) 1.1 0.40 – / –
ESV01862 (0.006%) 3.8 0.09 . – / –
ESV01867 (0.01%) 7.4 0.02 * – / –
ESV01896 (0.007%) 3.2 0.12 – / –
ESV01919 (0.01%) 1.0 0.44 N / –
ESV01922 (0.009%) 1.6 0.28 – / –
ESV01939 (0.008%) 3.8 0.08 . – / –
ESV01983 (0.009%) 15.5 0.004 ** – / –
ESV02093 (0.008%) 0.4 0.70 – / –
ESV02164 (0.009%) 0.7 0.53 – / –
ESV02177 (0.008%) 1.0 0.44 – / –

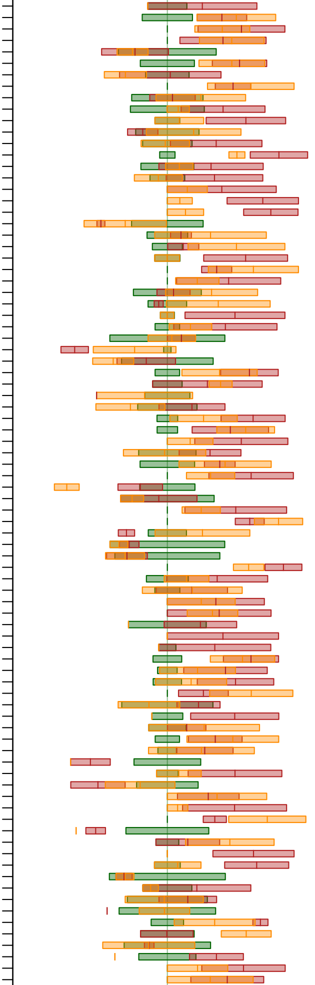

ESV02183 (0.005%)	0.7	0.54		- / -
ESV02239 (0.01%)	1.4	0.31		- / -
ESV02265 (0.008%)	1.7	0.26		- / -
ESV02290 (0.007%)	1.3	0.35		- / -
ESV02308 (0.008%)	0.3	0.75		- / -
ESV02334 (0.007%)	1.8	0.24		- / -
ESV02339 (0.006%)	0.5	0.61		- / -
ESV02445 (0.008%)	2.6	0.15		- / -
ESV02490 (0.007%)	0.3	0.78		- / -
ESV02495 (0.008%)	0.8	0.49		- / -
ESV02545 (0.005%)	2.3	0.18		- / -
ESV02556 (0.009%)	0.6	0.60		- / -
ESV02572 (0.006%)	0.8	0.51		- / -
ESV02627 (0.02%)	10.1	0.01	*	- / -
ESV02794 (0.006%)	0.4	0.67		- / -
ESV02848 (0.005%)	0.8	0.47		- / -
ESV02859 (0.005%)	0.7	0.54		- / -
ESV02881 (0.004%)	5.6	0.04	*	- / -
ESV02914 (0.006%)	8.6	0.02	*	- / -
ESV02998 (0.006%)	1.1	0.38		- / -
ESV03050 (0.01%)	0.4	0.69		- / -
ESV03080 (0.004%)	1.4	0.32		- / -
ESV03099 (0.004%)	3.0	0.13		- / -
ESV03151 (0.005%)	2.5	0.16		- / -
ESV03160 (0.006%)	0.9	0.46		- / -
ESV03179 (0.009%)	0.5	0.64		- / -
ESV03209 (0.01%)	1.0	0.42		- / -
ESV03224 (0.006%)	1.8	0.25		- / -
ESV03225 (0.004%)	0.8	0.50		- / -
ESV03249 (0.02%)	0.0	0.96		- / -
ESV03263 (0.01%)	3.5	0.10	.	- / -
ESV03264 (0.007%)	0.7	0.55		- / -
ESV03270 (0.007%)	2.2	0.20		- / -
ESV03389 (0.004%)	0.7	0.54		- / -
ESV03417 (0.004%)	1.4	0.32		- / -
ESV03475 (0.01%)	0.9	0.45		- / -
ESV03531 (0.007%)	2.4	0.17		- / -
ESV03538 (0.007%)	2.2	0.20		- / -
ESV03648 (0.003%)	1.6	0.28		- / -
ESV03681 (0.007%)	0.6	0.60		- / -
ESV03752 (0.01%)	1.0	0.43		- / -
ESV03767 (0.004%)	2.2	0.19		- / -
ESV03773 (0.01%)	5.7	0.04	*	- / -
ESV03810 (0.004%)	0.3	0.77		N / -
ESV03859 (0.004%)	1.2	0.37		- / -
ESV03902 (0.004%)	12.6	0.007	**	- / -
ESV04055 (0.007%)	1.6	0.27		- / -
ESV04059 (0.004%)	0.6	0.60		- / -
ESV04092 (0.006%)	0.5	0.64		- / -
ESV04097 (0.005%)	18.6	0.003	**	- / -
ESV04101 (0.004%)	0.5	0.61		- / -
ESV04224 (0.005%)	0.2	0.86		- / -
ESV04255 (0.003%)	0.5	0.61		- / -
ESV04260 (0.006%)	0.7	0.53		- / -
ESV04263 (0.003%)	1.4	0.32		- / -
ESV04338 (0.003%)	1.0	0.42		N / -
ESV04387 (0.004%)	0.9	0.47		N / -
ESV04415 (0.007%)	3.2	0.11		- / -
ESV04488 (0.009%)	0.8	0.50		- / -
ESV04553 (0.007%)	1.2	0.36		- / -
ESV04595 (0.006%)	2.3	0.19		- / -
ESV04644 (0.01%)	0.5	0.61		- / -
ESV04648 (0.004%)	2.7	0.15		- / -
ESV04698 (0.003%)	0.3	0.77		- / -
ESV04701 (0.02%)	1.2	0.38		- / -
ESV04702 (0.006%)	0.3	0.72		- / -
ESV04762 (0.004%)	5.2	0.05	*	- / -
ESV04864 (0.005%)	1.4	0.32		- / -
ESV04946 (0.005%)	1.3	0.35		- / -
ESV04973 (0.005%)	0.6	0.57		- / -
ESV05006 (0.004%)	1.4	0.32		- / -
ESV05233 (0.007%)	4.6	0.06	.	- / -
ESV05245 (0.005%)	3.8	0.09	.	- / -
ESV05300 (0.005%)	1.0	0.43		- / -
ESV05312 (0.002%)	4.5	0.06	.	N / -
ESV05418 (0.005%)	4.1	0.07	.	- / -
ESV05555 (0.007%)	0.5	0.62		- / -
ESV05610 (0.005%)	0.5	0.65		- / -
ESV05649 (0.008%)	0.1	0.89		- / -
ESV05656 (0.01%)	1.2	0.37		- / -
ESV05667 (0.005%)	3.3	0.11		- / -
ESV05767 (0.003%)	3.1	0.12		N / -
ESV05842 (0.004%)	0.1	0.92		- / -
ESV05878 (0.003%)	5.0	0.05	.	- / -
ESV05977 (0.003%)	1.7	0.27		- / -
ESV06029 (0.002%)	0.9	0.45		- / -

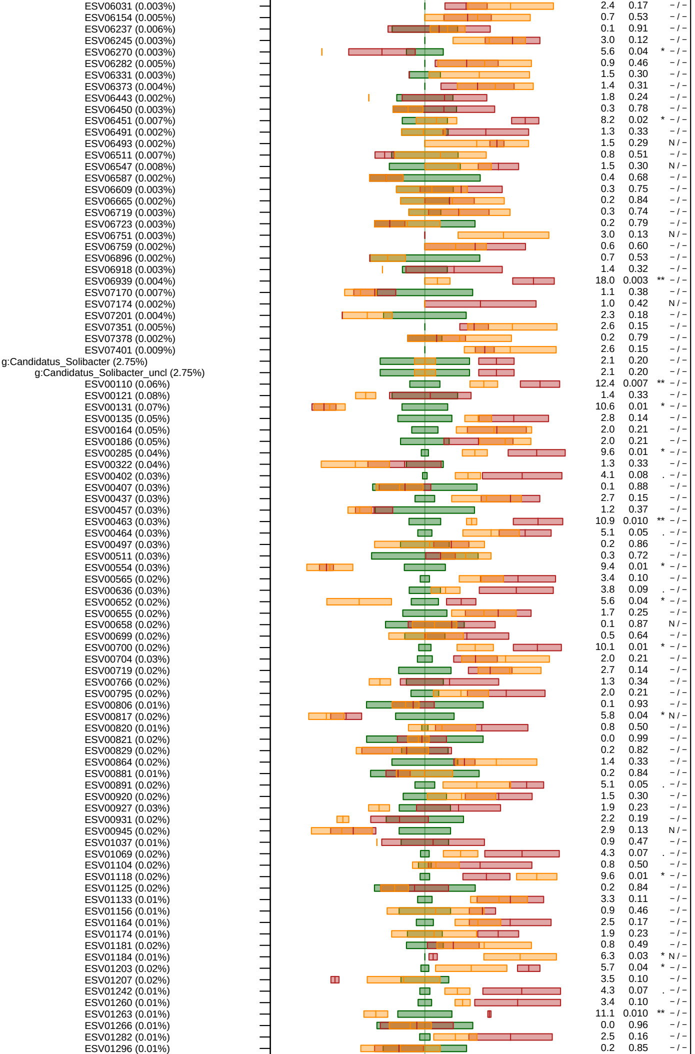
ESV06031 (0.003%) 2.4 0.17 – / –
ESV06154 (0.005%) 0.7 0.53 – / –
ESV06237 (0.006%) 0.1 0.91 – / –
ESV06245 (0.003%) 3.0 0.12 – / –
ESV06270 (0.003%) 5.6 0.04 * – / –
ESV06282 (0.005%) 0.9 0.46 – / –
ESV06331 (0.003%) 1.5 0.30 – / –
ESV06373 (0.004%) 1.4 0.31 – / –
ESV06443 (0.002%) 1.8 0.24 – / –
ESV06450 (0.003%) 0.3 0.78 – / –
ESV06451 (0.007%) 8.2 0.02 * – / –
ESV06491 (0.002%) 1.3 0.33 – / –
ESV06493 (0.002%) 1.5 0.29 N / –
ESV06511 (0.007%) 0.8 0.51 – / –
ESV06547 (0.008%) 1.5 0.30 N / –
ESV06587 (0.002%) 0.4 0.68 – / –
ESV06609 (0.003%) 0.3 0.75 – / –
ESV06665 (0.002%) 0.2 0.84 – / –
ESV06719 (0.003%) 0.3 0.74 – / –
ESV06723 (0.003%) 0.2 0.79 – / –
ESV06751 (0.003%) 3.0 0.13 N / –
ESV06759 (0.002%) 0.6 0.60 – / –
ESV06896 (0.002%) 0.7 0.53 – / –
ESV06918 (0.003%) 1.4 0.32 – / –
ESV06939 (0.004%) 18.0 0.003 ** – / –
ESV07170 (0.007%) 1.1 0.38 – / –
ESV07174 (0.002%) 1.0 0.42 N / –
ESV07201 (0.004%) 2.3 0.18 – / –
ESV07351 (0.005%) 2.6 0.15 – / –
ESV07378 (0.002%) 0.2 0.79 – / –
ESV07401 (0.009%) 2.6 0.15 – / –
g:Candidatus_Solibacter (2.75%) 2.1 0.20 – / –
g:Candidatus_Solibacter_uncl (2.75%) 2.1 0.20 – / –
ESV00110 (0.06%) 12.4 0.007 ** – / –
ESV00121 (0.08%) 1.4 0.33 – / –
ESV00131 (0.07%) 10.6 0.01 * – / –
ESV00135 (0.05%) 2.8 0.14 – / –
ESV00164 (0.05%) 2.0 0.21 – / –
ESV00186 (0.05%) 2.0 0.21 – / –
ESV00285 (0.04%) 9.6 0.01 * – / –
ESV00322 (0.04%) 1.3 0.33 – / –
ESV00402 (0.03%) 4.1 0.08 . – / –
ESV00407 (0.03%) 0.1 0.88 – / –
ESV00437 (0.03%) 2.7 0.15 – / –
ESV00457 (0.03%) 1.2 0.37 – / –
ESV00463 (0.03%) 10.9 0.010 ** – / –
ESV00464 (0.03%) 5.1 0.05 . – / –
ESV00497 (0.03%) 0.2 0.86 – / –
ESV00511 (0.03%) 0.3 0.72 – / –
ESV00554 (0.03%) 9.4 0.01 * – / –
ESV00565 (0.02%) 3.4 0.10 – / –
ESV00636 (0.03%) 3.8 0.09 . – / –
ESV00652 (0.02%) 5.6 0.04 * – / –
ESV00655 (0.02%) 1.7 0.25 – / –
ESV00658 (0.02%) 0.1 0.87 N / –
ESV00699 (0.02%) 0.5 0.64 – / –
ESV00700 (0.02%) 10.1 0.01 * – / –
ESV00704 (0.03%) 2.0 0.21 – / –
ESV00719 (0.02%) 2.7 0.14 – / –
ESV00766 (0.02%) 1.3 0.34 – / –
ESV00795 (0.02%) 2.0 0.21 – / –
ESV00806 (0.01%) 0.1 0.93 – / –
ESV00817 (0.02%) 5.8 0.04 * N / –
ESV00820 (0.01%) 0.8 0.50 – / –
ESV00821 (0.02%) 0.0 0.99 – / –
ESV00829 (0.02%) 0.2 0.82 – / –
ESV00864 (0.02%) 1.4 0.33 – / –
ESV00881 (0.01%) 0.2 0.84 – / –
ESV00891 (0.02%) 5.1 0.05 . – / –
ESV00920 (0.02%) 1.5 0.30 – / –
ESV00927 (0.03%) 1.9 0.23 – / –
ESV00931 (0.02%) 2.2 0.19 – / –
ESV00945 (0.02%) 2.9 0.13 N / –
ESV01037 (0.01%) 0.9 0.47 – / –
ESV01069 (0.02%) 4.3 0.07 . – / –
ESV01104 (0.02%) 0.8 0.50 – / –
ESV01118 (0.02%) 9.6 0.01 * – / –
ESV01125 (0.02%) 0.2 0.84 – / –
ESV01133 (0.01%) 3.3 0.11 – / –
ESV01156 (0.01%) 0.9 0.46 – / –
ESV01164 (0.01%) 2.5 0.17 – / –
ESV01174 (0.01%) 1.9 0.23 – / –
ESV01181 (0.02%) 0.8 0.49 – / –
ESV01184 (0.01%) 6.3 0.03 * N / –
ESV01203 (0.02%) 5.7 0.04 * – / –
ESV01207 (0.02%) 3.5 0.10 – / –
ESV01242 (0.01%) 4.3 0.07 . – / –
ESV01260 (0.01%) 3.4 0.10 – / –
ESV01263 (0.01%) 11.1 0.010 ** – / –
ESV01266 (0.01%) 0.0 0.96 – / –
ESV01282 (0.01%) 2.5 0.16 – / –
ESV01296 (0.01%) 0.2 0.85 – / –

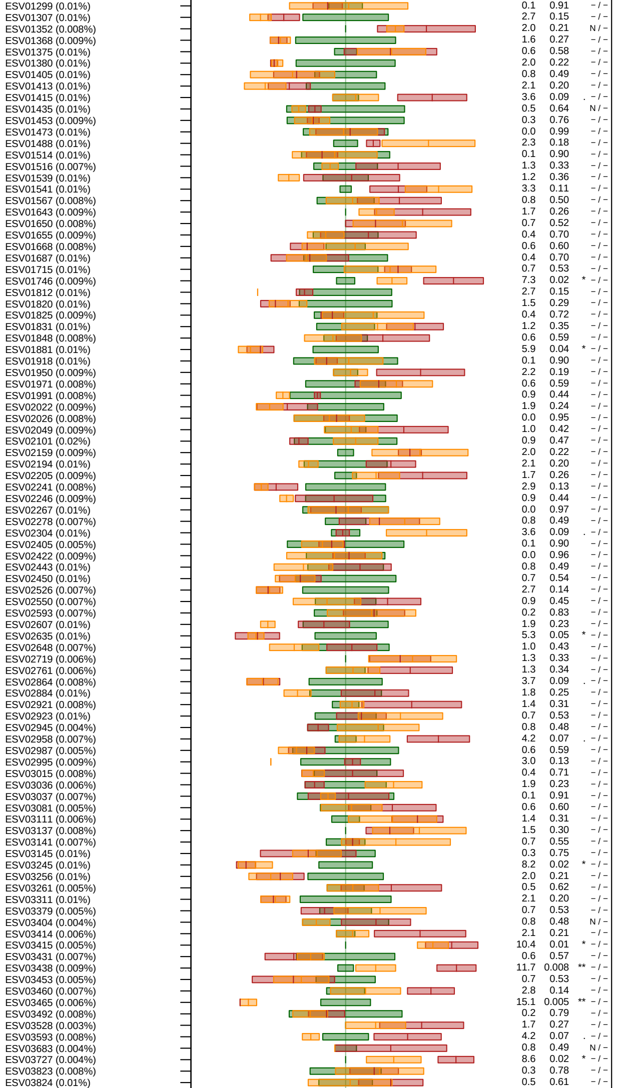
ESV01299 (0.01%) 0.1 0.91 − / −
ESV01307 (0.01%) 2.7 0.15 − / −
ESV01352 (0.008%) 2.0 0.21 N / −
ESV01368 (0.009%) 1.6 0.27 − / −
ESV01375 (0.01%) 0.6 0.58 − / −
ESV01380 (0.01%) 2.0 0.22 − / −
ESV01405 (0.01%) 0.8 0.49 − / −
ESV01413 (0.01%) 2.1 0.20 − / −
ESV01415 (0.01%) 3.6 0.09 . − / −
ESV01435 (0.01%) 0.5 0.64 N / −
ESV01453 (0.009%) 0.3 0.76 − / −
ESV01473 (0.01%) 0.0 0.99 − / −
ESV01488 (0.01%) 2.3 0.18 − / −
ESV01514 (0.01%) 0.1 0.90 − / −
ESV01516 (0.007%) 1.3 0.33 − / −
ESV01539 (0.01%) 1.2 0.36 − / −
ESV01541 (0.01%) 3.3 0.11 − / −
ESV01567 (0.008%) 0.8 0.50 − / −
ESV01643 (0.009%) 1.7 0.26 − / −
ESV01650 (0.008%) 0.7 0.52 − / −
ESV01655 (0.009%) 0.4 0.70 − / −
ESV01668 (0.008%) 0.6 0.60 − / −
ESV01687 (0.01%) 0.4 0.70 − / −
ESV01715 (0.01%) 0.7 0.53 − / −
ESV01746 (0.009%) 7.3 0.02 * − / −
ESV01812 (0.01%) 2.7 0.15 − / −
ESV01820 (0.01%) 1.5 0.29 − / −
ESV01825 (0.009%) 0.4 0.72 − / −
ESV01831 (0.01%) 1.2 0.35 − / −
ESV01848 (0.008%) 0.6 0.59 − / −
ESV01881 (0.01%) 5.9 0.04 * − / −
ESV01918 (0.01%) 0.1 0.90 − / −
ESV01950 (0.009%) 2.2 0.19 − / −
ESV01971 (0.008%) 0.6 0.59 − / −
ESV01991 (0.008%) 0.9 0.44 − / −
ESV02022 (0.009%) 1.9 0.24 − / −
ESV02026 (0.008%) 0.0 0.95 − / −
ESV02049 (0.009%) 1.0 0.42 − / −
ESV02101 (0.02%) 0.9 0.47 − / −
ESV02159 (0.009%) 2.0 0.22 − / −
ESV02194 (0.01%) 2.1 0.20 − / −
ESV02205 (0.009%) 1.7 0.26 − / −
ESV02241 (0.008%) 2.9 0.13 − / −
ESV02246 (0.009%) 0.9 0.44 − / −
ESV02267 (0.01%) 0.0 0.97 − / −
ESV02278 (0.007%) 0.8 0.49 − / −
ESV02304 (0.01%) 3.6 0.09 . − / −
ESV02405 (0.005%) 0.1 0.90 − / −
ESV02422 (0.009%) 0.0 0.96 − / −
ESV02443 (0.01%) 0.8 0.49 − / −
ESV02450 (0.01%) 0.7 0.54 − / −
ESV02526 (0.007%) 2.7 0.14 − / −
ESV02550 (0.007%) 0.9 0.45 − / −
ESV02593 (0.007%) 0.2 0.83 − / −
ESV02607 (0.01%) 1.9 0.23 − / −
ESV02635 (0.01%) 5.3 0.05 * − / −
ESV02648 (0.007%) 1.0 0.43 − / −
ESV02719 (0.006%) 1.3 0.33 − / −
ESV02761 (0.006%) 1.3 0.34 − / −
ESV02864 (0.008%) 3.7 0.09 . − / −
ESV02884 (0.01%) 1.8 0.25 − / −
ESV02921 (0.008%) 1.4 0.31 − / −
ESV02923 (0.01%) 0.7 0.53 − / −
ESV02945 (0.004%) 0.8 0.48 − / −
ESV02958 (0.007%) 4.2 0.07 . − / −
ESV02987 (0.005%) 0.6 0.59 − / −
ESV02995 (0.009%) 3.0 0.13 − / −
ESV03015 (0.008%) 0.4 0.71 − / −
ESV03036 (0.006%) 1.9 0.23 − / −
ESV03037 (0.007%) 0.1 0.91 − / −
ESV03081 (0.005%) 0.6 0.60 − / −
ESV03111 (0.006%) 1.4 0.31 − / −
ESV03137 (0.008%) 1.5 0.30 − / −
ESV03141 (0.007%) 0.7 0.55 − / −
ESV03145 (0.01%) 0.3 0.75 − / −
ESV03245 (0.01%) 8.2 0.02 * − / −
ESV03256 (0.01%) 2.0 0.21 − / −
ESV03261 (0.005%) 0.5 0.62 − / −
ESV03311 (0.01%) 2.1 0.20 − / −
ESV03379 (0.005%) 0.7 0.53 − / −
ESV03404 (0.004%) 0.8 0.48 N / −
ESV03414 (0.006%) 2.1 0.21 − / −
ESV03415 (0.005%) 10.4 0.01 * − / −
ESV03431 (0.007%) 0.6 0.57 − / −
ESV03438 (0.009%) 11.7 0.008 ** − / −
ESV03453 (0.005%) 0.7 0.53 − / −
ESV03460 (0.007%) 2.8 0.14 − / −
ESV03465 (0.006%) 15.1 0.005 ** − / −
ESV03492 (0.008%) 0.2 0.79 − / −
ESV03528 (0.003%) 1.7 0.27 − / −
ESV03593 (0.008%) 4.2 0.07 . − / −
ESV03683 (0.004%) 0.8 0.49 N / −
ESV03727 (0.004%) 8.6 0.02 * − / −
ESV03823 (0.008%) 0.3 0.78 − / −
ESV03824 (0.01%) 0.5 0.61 − / −

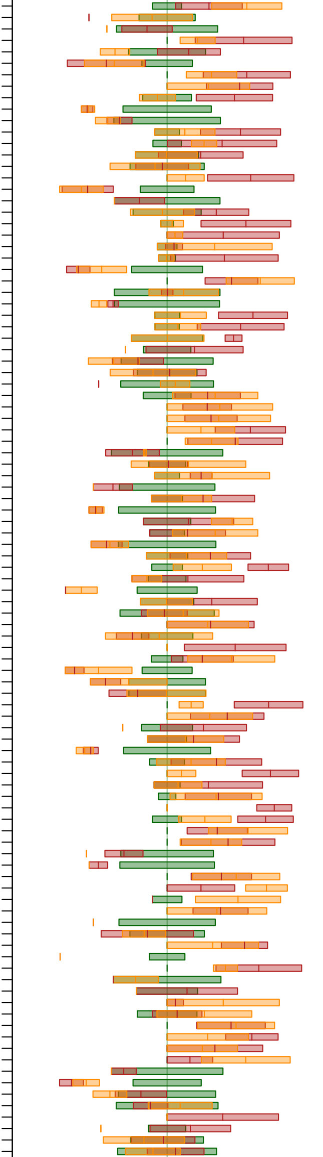

ESV03846 (0.008%)	1.9	0.23		– / –
ESV03886 (0.007%)	2.1	0.20		– / –
ESV03889 (0.007%)	0.9	0.46		– / –
ESV03931 (0.005%)	1.7	0.26		– / –
ESV03952 (0.005%)	1.6	0.28		– / –
ESV04025 (0.01%)	1.1	0.40		– / –
ESV04085 (0.004%)	1.9	0.23		– / –
ESV04087 (0.004%)	1.4	0.31		N / –
ESV04126 (0.005%)	2.2	0.19		– / –
ESV04130 (0.006%)	3.1	0.12		– / –
ESV04144 (0.004%)	0.9	0.44		– / –
ESV04153 (0.007%)	1.6	0.27		– / –
ESV04203 (0.004%)	0.7	0.53		– / –
ESV04210 (0.004%)	0.3	0.74		N / –
ESV04215 (0.005%)	0.0	0.97		– / –
ESV04251 (0.002%)	2.6	0.15		– / –
ESV04257 (0.009%)	3.7	0.09	.	– / –
ESV04362 (0.003%)	0.6	0.57		– / –
ESV04445 (0.01%)	0.8	0.48		– / –
ESV04446 (0.008%)	2.7	0.15		– / –
ESV04460 (0.003%)	0.9	0.47		N / –
ESV04477 (0.004%)	0.6	0.59		– / –
ESV04481 (0.004%)	1.1	0.39		– / –
ESV04508 (0.009%)	3.2	0.12		– / –
ESV04525 (0.005%)	3.7	0.09	.	– / –
ESV04573 (0.004%)	0.1	0.93		– / –
ESV04638 (0.007%)	1.4	0.32		– / –
ESV04713 (0.005%)	3.2	0.11		– / –
ESV04718 (0.004%)	1.8	0.24		– / –
ESV04802 (0.002%)	1.6	0.27		N / –
ESV04840 (0.004%)	1.2	0.35		– / –
ESV04861 (0.003%)	0.6	0.57		– / –
ESV04888 (0.003%)	0.1	0.94		– / –
ESV04936 (0.004%)	2.2	0.19		– / –
ESV04940 (0.006%)	0.6	0.59		– / –
ESV04979 (0.003%)	0.7	0.54		– / –
ESV04981 (0.003%)	0.7	0.54		– / –
ESV05033 (0.003%)	2.2	0.19		N / –
ESV05040 (0.003%)	1.2	0.36		– / –
ESV05041 (0.008%)	0.2	0.79		– / –
ESV05044 (0.006%)	0.1	0.90		– / –
ESV05047 (0.004%)	0.5	0.65		– / –
ESV05067 (0.002%)	1.6	0.27		– / –
ESV05086 (0.003%)	0.3	0.78		– / –
ESV05158 (0.005%)	2.1	0.21		– / –
ESV05216 (0.006%)	1.1	0.40		N / –
ESV05232 (0.003%)	0.5	0.63		– / –
ESV05246 (0.003%)	1.2	0.37		– / –
ESV05252 (0.003%)	0.4	0.70		– / –
ESV05262 (0.005%)	5.3	0.05	*	– / –
ESV05265 (0.003%)	0.3	0.72		– / –
ESV05317 (0.003%)	7.8	0.02	*	– / –
ESV05398 (0.002%)	0.6	0.58		– / –
ESV05417 (0.003%)	0.1	0.93		– / –
ESV05430 (0.003%)	0.5	0.63		N / –
ESV05457 (0.007%)	0.3	0.78		– / –
ESV05476 (0.003%)	1.8	0.25		N / –
ESV05654 (0.004%)	1.0	0.42		– / –
ESV05704 (0.005%)	3.8	0.09	.	– / –
ESV05737 (0.003%)	0.9	0.45		– / –
ESV05813 (0.004%)	0.2	0.81		– / –
ESV05817 (0.005%)	6.4	0.03	*	– / –
ESV05827 (0.002%)	0.9	0.45		– / –
ESV05890 (0.004%)	2.0	0.22		– / –
ESV05935 (0.003%)	0.1	0.87		N / –
ESV05936 (0.007%)	3.1	0.12		– / –
ESV05943 (0.008%)	0.5	0.62		– / –
ESV05995 (0.003%)	9.4	0.01	*	– / –
ESV06012 (0.003%)	0.4	0.70		– / –
ESV06013 (0.007%)	0.8	0.51		– / –
ESV06027 (0.002%)	37.6	< 0.001	***	N / –
ESV06049 (0.006%)	4.4	0.07	.	– / –
ESV06086 (0.004%)	2.0	0.21		– / –
ESV06095 (0.005%)	0.9	0.44		– / –
ESV06142 (0.003%)	2.0	0.22		– / –
ESV06171 (0.003%)	2.3	0.18		N / –
ESV06216 (0.003%)	1.4	0.31		– / –
ESV06218 (0.003%)	4.8	0.06	.	– / –
ESV06247 (0.005%)	3.1	0.12		– / –
ESV06276 (0.002%)	0.8	0.48		– / –
ESV06295 (0.002%)	2.3	0.18		N / –
ESV06303 (0.003%)	0.1	0.89		– / –
ESV06325 (0.005%)	1.7	0.26		– / –
ESV06383 (0.002%)	36.2	< 0.001	***	N / –
ESV06452 (0.006%)	3.3	0.11		– / –
ESV06548 (0.003%)	0.6	0.56		– / –
ESV06595 (0.002%)	0.6	0.60		– / –
ESV06678 (0.003%)	0.9	0.47		N / –
ESV06715 (0.004%)	0.3	0.75		– / –
ESV06780 (0.003%)	1.7	0.27		– / –
ESV06807 (0.003%)	2.3	0.18		– / –
ESV06849 (0.003%)	0.5	0.62		– / –
ESV06863 (0.002%)	2.0	0.22		– / –
ESV06869 (0.002%)	0.8	0.50		– / –
ESV06900 (0.005%)	5.4	0.05	*	– / –
ESV06903 (0.004%)	0.7	0.52		– / –
ESV06937 (0.004%)	0.2	0.85		– / –
ESV06996 (0.002%)	1.0	0.42		N / –
ESV07005 (0.006%)	3.3	0.11		– / –
ESV07016 (0.003%)	0.1	0.89		– / –
ESV07018 (0.008%)	0.1	0.91		– / –

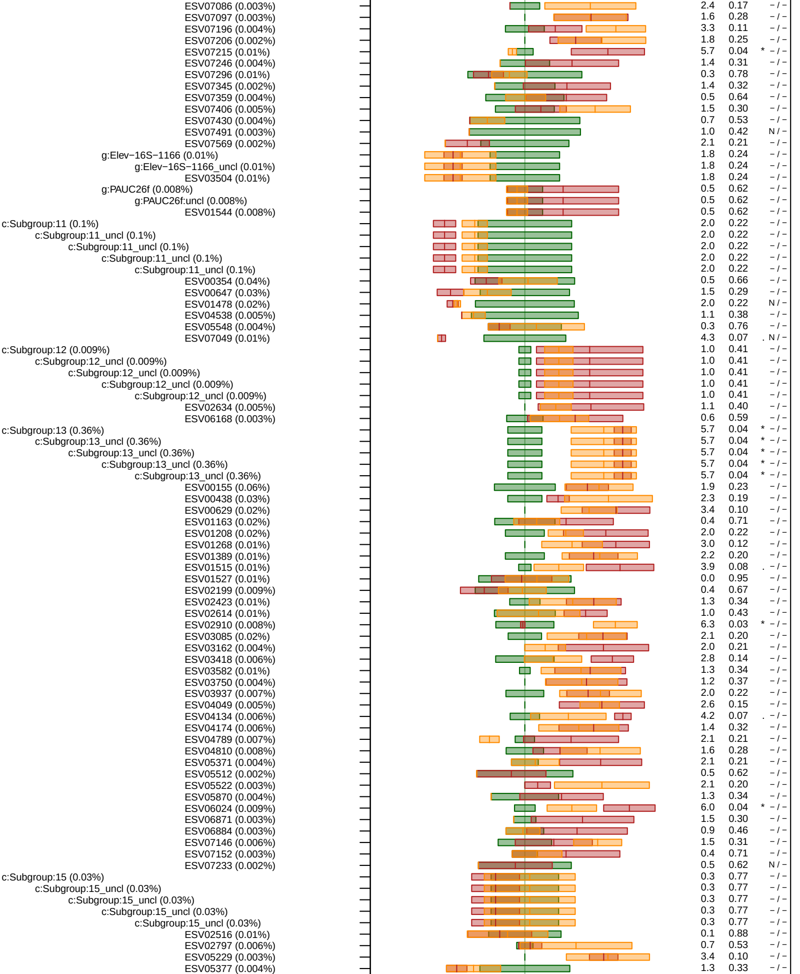
ESV07086 (0.003%) 2.4 0.17 – / –
ESV07097 (0.003%) 1.6 0.28 – / –
ESV07196 (0.004%) 3.3 0.11 – / –
ESV07206 (0.002%) 1.8 0.25 – / –
ESV07215 (0.01%) 5.7 0.04 * – / –
ESV07246 (0.004%) 1.4 0.31 – / –
ESV07296 (0.01%) 0.3 0.78 – / –
ESV07345 (0.002%) 1.4 0.32 – / –
ESV07359 (0.004%) 0.5 0.64 – / –
ESV07406 (0.005%) 1.5 0.30 – / –
ESV07430 (0.004%) 0.7 0.53 – / –
ESV07491 (0.003%) 1.0 0.42 N / –
ESV07569 (0.002%) 2.1 0.21 – / –
g:Elev−16S−1166 (0.01%) 1.8 0.24 – / –
g:Elev−16S−1166_uncl (0.01%) 1.8 0.24 – / –
ESV03504 (0.01%) 1.8 0.24 – / –
g:PAUC26f (0.008%) 0.5 0.62 – / –
g:PAUC26f:uncl (0.008%) 0.5 0.62 – / –
ESV01544 (0.008%) 0.5 0.62 – / –
c:Subgroup:11 (0.1%) 2.0 0.22 – / –
c:Subgroup:11_uncl (0.1%) 2.0 0.22 – / –
c:Subgroup:11_uncl (0.1%) 2.0 0.22 – / –
c:Subgroup:11_uncl (0.1%) 2.0 0.22 – / –
c:Subgroup:11_uncl (0.1%) 2.0 0.22 – / –
ESV00354 (0.04%) 0.5 0.66 – / –
ESV00647 (0.03%) 1.5 0.29 – / –
ESV01478 (0.02%) 2.0 0.22 N / –
ESV04538 (0.005%) 1.1 0.38 – / –
ESV05548 (0.004%) 0.3 0.76 – / –
ESV07049 (0.01%) 4.3 0.07 . N / –
c:Subgroup:12 (0.009%) 1.0 0.41 – / –
c:Subgroup:12_uncl (0.009%) 1.0 0.41 – / –
c:Subgroup:12_uncl (0.009%) 1.0 0.41 – / –
c:Subgroup:12_uncl (0.009%) 1.0 0.41 – / –
c:Subgroup:12_uncl (0.009%) 1.0 0.41 – / –
ESV02634 (0.005%) 1.1 0.40 – / –
ESV06168 (0.003%) 0.6 0.59 – / –
c:Subgroup:13 (0.36%) 5.7 0.04 * – / –
c:Subgroup:13_uncl (0.36%) 5.7 0.04 * – / –
c:Subgroup:13_uncl (0.36%) 5.7 0.04 * – / –
c:Subgroup:13_uncl (0.36%) 5.7 0.04 * – / –
c:Subgroup:13_uncl (0.36%) 5.7 0.04 * – / –
ESV00155 (0.06%) 1.9 0.23 – / –
ESV00438 (0.03%) 2.3 0.19 – / –
ESV00629 (0.02%) 3.4 0.10 – / –
ESV01163 (0.02%) 0.4 0.71 – / –
ESV01208 (0.02%) 2.0 0.22 – / –
ESV01268 (0.01%) 3.0 0.12 – / –
ESV01389 (0.01%) 2.2 0.20 – / –
ESV01515 (0.01%) 3.9 0.08 . – / –
ESV01527 (0.01%) 0.0 0.95 – / –
ESV02199 (0.009%) 0.4 0.67 – / –
ESV02423 (0.01%) 1.3 0.34 – / –
ESV02614 (0.01%) 1.0 0.43 – / –
ESV02910 (0.008%) 6.3 0.03 * – / –
ESV03085 (0.02%) 2.1 0.20 – / –
ESV03162 (0.004%) 2.0 0.21 – / –
ESV03418 (0.006%) 2.8 0.14 – / –
ESV03582 (0.01%) 1.3 0.34 – / –
ESV03750 (0.004%) 1.2 0.37 – / –
ESV03937 (0.007%) 2.0 0.22 – / –
ESV04049 (0.005%) 2.6 0.15 – / –
ESV04134 (0.006%) 4.2 0.07 . – / –
ESV04174 (0.006%) 1.4 0.32 – / –
ESV04789 (0.007%) 2.1 0.21 – / –
ESV04810 (0.008%) 1.6 0.28 – / –
ESV05371 (0.004%) 2.1 0.21 – / –
ESV05512 (0.002%) 0.5 0.62 – / –
ESV05522 (0.003%) 2.1 0.20 – / –
ESV05870 (0.004%) 1.3 0.34 – / –
ESV06024 (0.009%) 6.0 0.04 * – / –
ESV06871 (0.003%) 1.5 0.30 – / –
ESV06884 (0.003%) 0.9 0.46 – / –
ESV07146 (0.006%) 1.5 0.31 – / –
ESV07152 (0.003%) 0.4 0.71 – / –
ESV07233 (0.002%) 0.5 0.62 N / –
c:Subgroup:15 (0.03%) 0.3 0.77 – / –
c:Subgroup:15_uncl (0.03%) 0.3 0.77 – / –
c:Subgroup:15_uncl (0.03%) 0.3 0.77 – / –
c:Subgroup:15_uncl (0.03%) 0.3 0.77 – / –
c:Subgroup:15_uncl (0.03%) 0.3 0.77 – / –
ESV02516 (0.01%) 0.1 0.88 – / –
ESV02797 (0.006%) 0.7 0.53 – / –
ESV05229 (0.003%) 3.4 0.10 – / –
ESV05377 (0.004%) 1.3 0.33 – / –

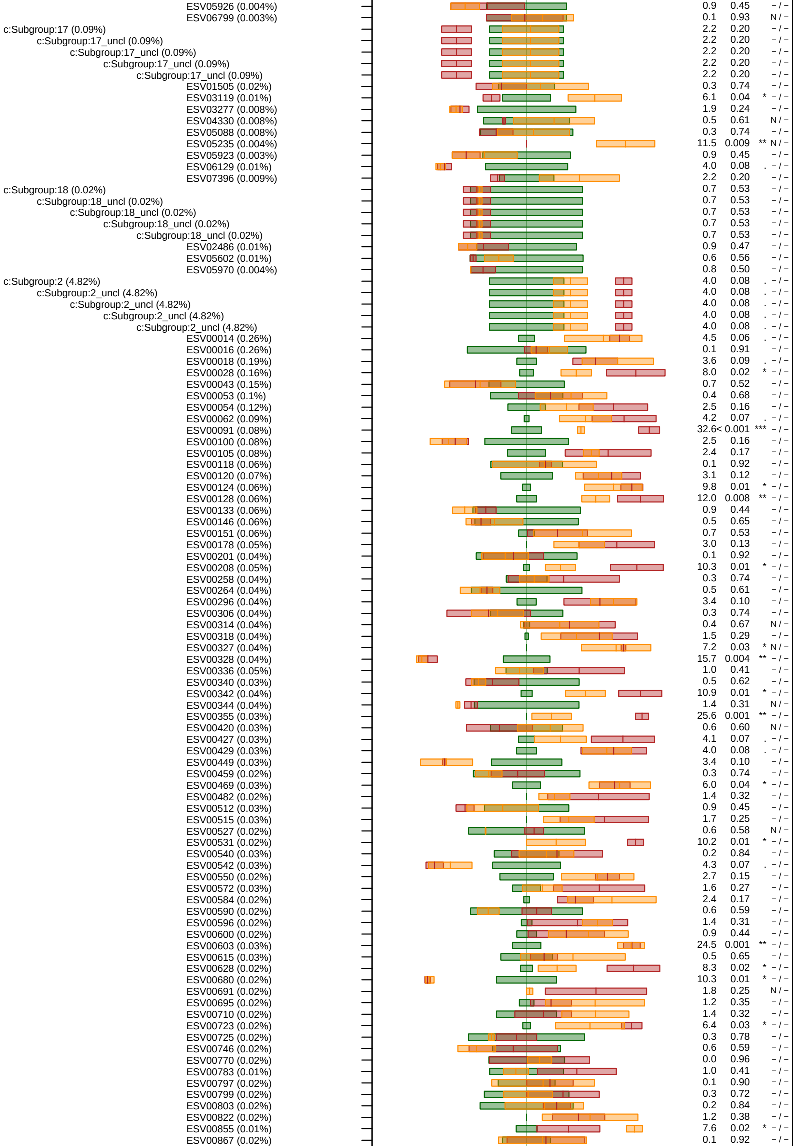
ESV05926 (0.004%) 0.9 0.45 - / -
ESV06799 (0.003%) 0.1 0.93 N / -
c:Subgroup:17 (0.09%) 2.2 0.20 - / -
c:Subgroup:17_uncl (0.09%) 2.2 0.20 - / -
c:Subgroup:17_uncl (0.09%) 2.2 0.20 - / -
c:Subgroup:17_uncl (0.09%) 2.2 0.20 - / -
c:Subgroup:17_uncl (0.09%) 2.2 0.20 - / -
ESV01505 (0.02%) 0.3 0.74 - / -
ESV03119 (0.01%) 6.1 0.04 * - / -
ESV03277 (0.008%) 1.9 0.24 - / -
ESV04330 (0.008%) 0.5 0.61 N / -
ESV05088 (0.008%) 0.3 0.74 - / -
ESV05235 (0.004%) 11.5 0.009 ** N / -
ESV05923 (0.003%) 0.9 0.45 - / -
ESV06129 (0.01%) 4.0 0.08 . - / -
ESV07396 (0.009%) 2.2 0.20 - / -
c:Subgroup:18 (0.02%) 0.7 0.53 - / -
c:Subgroup:18_uncl (0.02%) 0.7 0.53 - / -
c:Subgroup:18_uncl (0.02%) 0.7 0.53 - / -
c:Subgroup:18_uncl (0.02%) 0.7 0.53 - / -
c:Subgroup:18_uncl (0.02%) 0.7 0.53 - / -
ESV02486 (0.01%) 0.9 0.47 - / -
ESV05602 (0.01%) 0.6 0.56 - / -
ESV05970 (0.004%) 0.8 0.50 - / -
c:Subgroup:2 (4.82%) 4.0 0.08 . - / -
c:Subgroup:2_uncl (4.82%) 4.0 0.08 . - / -
c:Subgroup:2_uncl (4.82%) 4.0 0.08 . - / -
c:Subgroup:2_uncl (4.82%) 4.0 0.08 . - / -
c:Subgroup:2_uncl (4.82%) 4.0 0.08 . - / -
ESV00014 (0.26%) 4.5 0.06 . - / -
ESV00016 (0.26%) 0.1 0.91 - / -
ESV00018 (0.19%) 3.6 0.09 . - / -
ESV00028 (0.16%) 8.0 0.02 * - / -
ESV00043 (0.15%) 0.7 0.52 - / -
ESV00053 (0.1%) 0.4 0.68 - / -
ESV00054 (0.12%) 2.5 0.16 - / -
ESV00062 (0.09%) 4.2 0.07 . - / -
ESV00091 (0.08%) 32.6 < 0.001 *** - / -
ESV00100 (0.08%) 2.5 0.16 - / -
ESV00105 (0.08%) 2.4 0.17 - / -
ESV00118 (0.06%) 0.1 0.92 - / -
ESV00120 (0.07%) 3.1 0.12 - / -
ESV00124 (0.06%) 9.8 0.01 * - / -
ESV00128 (0.06%) 12.0 0.008 ** - / -
ESV00133 (0.06%) 0.9 0.44 - / -
ESV00146 (0.06%) 0.5 0.65 - / -
ESV00151 (0.06%) 0.7 0.53 - / -
ESV00178 (0.05%) 3.0 0.13 - / -
ESV00201 (0.04%) 0.1 0.92 - / -
ESV00208 (0.05%) 10.3 0.01 * - / -
ESV00258 (0.04%) 0.3 0.74 - / -
ESV00264 (0.04%) 0.5 0.61 - / -
ESV00296 (0.04%) 3.4 0.10 - / -
ESV00306 (0.04%) 0.3 0.74 - / -
ESV00314 (0.04%) 0.4 0.67 N / -
ESV00318 (0.04%) 1.5 0.29 - / -
ESV00327 (0.04%) 7.2 0.03 * N / -
ESV00328 (0.04%) 15.7 0.004 ** - / -
ESV00336 (0.05%) 1.0 0.41 - / -
ESV00340 (0.03%) 0.5 0.62 - / -
ESV00342 (0.04%) 10.9 0.01 * - / -
ESV00344 (0.04%) 1.4 0.31 N / -
ESV00355 (0.03%) 25.6 0.001 ** - / -
ESV00420 (0.03%) 0.6 0.60 N / -
ESV00427 (0.03%) 4.1 0.07 . - / -
ESV00429 (0.03%) 4.0 0.08 . - / -
ESV00449 (0.03%) 3.4 0.10 - / -
ESV00459 (0.02%) 0.3 0.74 - / -
ESV00469 (0.03%) 6.0 0.04 * - / -
ESV00482 (0.02%) 1.4 0.32 - / -
ESV00512 (0.03%) 0.9 0.45 - / -
ESV00515 (0.03%) 1.7 0.25 - / -
ESV00527 (0.02%) 0.6 0.58 N / -
ESV00531 (0.02%) 10.2 0.01 * - / -
ESV00540 (0.03%) 0.2 0.84 - / -
ESV00542 (0.03%) 4.3 0.07 . - / -
ESV00550 (0.02%) 2.7 0.15 - / -
ESV00572 (0.03%) 1.6 0.27 - / -
ESV00584 (0.02%) 2.4 0.17 - / -
ESV00590 (0.02%) 0.6 0.59 - / -
ESV00596 (0.02%) 1.4 0.31 - / -
ESV00600 (0.02%) 0.9 0.44 - / -
ESV00603 (0.03%) 24.5 0.001 ** - / -
ESV00615 (0.03%) 0.5 0.65 - / -
ESV00628 (0.02%) 8.3 0.02 * - / -
ESV00680 (0.02%) 10.3 0.01 * - / -
ESV00691 (0.02%) 1.8 0.25 N / -
ESV00695 (0.02%) 1.2 0.35 - / -
ESV00710 (0.02%) 1.4 0.32 - / -
ESV00723 (0.02%) 6.4 0.03 * - / -
ESV00725 (0.02%) 0.3 0.78 - / -
ESV00746 (0.02%) 0.6 0.59 - / -
ESV00770 (0.02%) 0.0 0.96 - / -
ESV00783 (0.01%) 1.0 0.41 - / -
ESV00797 (0.02%) 0.1 0.90 - / -
ESV00799 (0.02%) 0.3 0.72 - / -
ESV00803 (0.02%) 0.2 0.84 - / -
ESV00822 (0.02%) 1.2 0.38 - / -
ESV00855 (0.01%) 7.6 0.02 * - / -
ESV00867 (0.02%) 0.1 0.92 - / -

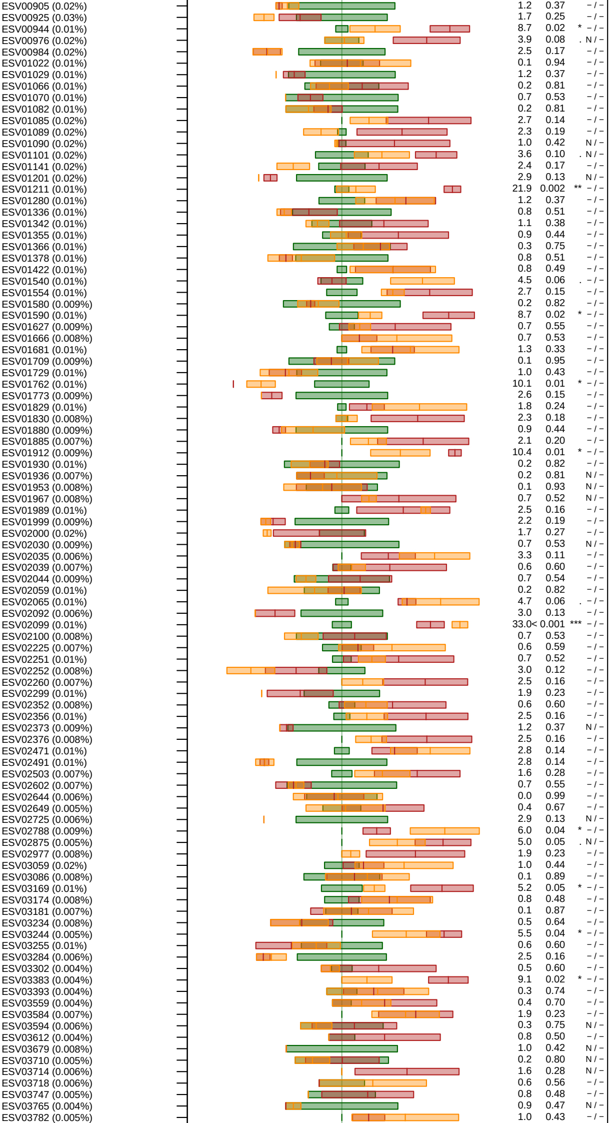
ESV00905 (0.02%) 1.2 0.37 – / –
ESV00925 (0.03%) 1.7 0.25 – / –
ESV00944 (0.01%) 8.7 0.02 * – / –
ESV00976 (0.02%) 3.9 0.08 . N / –
ESV00984 (0.02%) 2.5 0.17 – / –
ESV01022 (0.01%) 0.1 0.94 – / –
ESV01029 (0.01%) 1.2 0.37 – / –
ESV01066 (0.01%) 0.2 0.81 – / –
ESV01070 (0.01%) 0.7 0.53 – / –
ESV01082 (0.01%) 0.2 0.81 – / –
ESV01085 (0.02%) 2.7 0.14 – / –
ESV01089 (0.02%) 2.3 0.19 – / –
ESV01090 (0.02%) 1.0 0.42 N / –
ESV01101 (0.02%) 3.6 0.10 . N / –
ESV01141 (0.02%) 2.4 0.17 – / –
ESV01201 (0.02%) 2.9 0.13 N / –
ESV01211 (0.01%) 21.9 0.002 ** – / –
ESV01280 (0.01%) 1.2 0.37 – / –
ESV01336 (0.01%) 0.8 0.51 – / –
ESV01342 (0.01%) 1.1 0.38 – / –
ESV01355 (0.01%) 0.9 0.44 – / –
ESV01366 (0.01%) 0.3 0.75 – / –
ESV01378 (0.01%) 0.8 0.51 – / –
ESV01422 (0.01%) 0.8 0.49 – / –
ESV01540 (0.01%) 4.5 0.06 . – / –
ESV01554 (0.01%) 2.7 0.15 – / –
ESV01580 (0.009%) 0.2 0.82 – / –
ESV01590 (0.01%) 8.7 0.02 * – / –
ESV01627 (0.009%) 0.7 0.55 – / –
ESV01666 (0.008%) 0.7 0.53 – / –
ESV01681 (0.01%) 1.3 0.33 – / –
ESV01709 (0.009%) 0.1 0.95 – / –
ESV01729 (0.01%) 1.0 0.43 – / –
ESV01762 (0.01%) 10.1 0.01 * – / –
ESV01773 (0.009%) 2.6 0.15 – / –
ESV01829 (0.01%) 1.8 0.24 – / –
ESV01830 (0.008%) 2.3 0.18 – / –
ESV01880 (0.009%) 0.9 0.44 – / –
ESV01885 (0.007%) 2.1 0.20 – / –
ESV01912 (0.009%) 10.4 0.01 * – / –
ESV01930 (0.01%) 0.2 0.82 – / –
ESV01936 (0.007%) 0.2 0.81 N / –
ESV01953 (0.008%) 0.1 0.93 N / –
ESV01967 (0.008%) 0.7 0.52 N / –
ESV01989 (0.01%) 2.5 0.16 – / –
ESV01999 (0.009%) 2.2 0.19 – / –
ESV02000 (0.02%) 1.7 0.27 – / –
ESV02030 (0.009%) 0.7 0.53 N / –
ESV02035 (0.006%) 3.3 0.11 – / –
ESV02039 (0.007%) 0.6 0.60 – / –
ESV02044 (0.009%) 0.7 0.54 – / –
ESV02059 (0.01%) 0.2 0.82 – / –
ESV02065 (0.01%) 4.7 0.06 . – / –
ESV02092 (0.006%) 3.0 0.13 – / –
ESV02099 (0.01%) 33.0 < 0.001 *** – / –
ESV02100 (0.008%) 0.7 0.53 – / –
ESV02225 (0.007%) 0.6 0.59 – / –
ESV02251 (0.01%) 0.7 0.52 – / –
ESV02252 (0.008%) 3.0 0.12 – / –
ESV02260 (0.007%) 2.5 0.16 – / –
ESV02299 (0.01%) 1.9 0.23 – / –
ESV02352 (0.008%) 0.6 0.60 – / –
ESV02356 (0.01%) 2.5 0.16 – / –
ESV02373 (0.009%) 1.2 0.37 N / –
ESV02376 (0.008%) 2.5 0.16 – / –
ESV02471 (0.01%) 2.8 0.14 – / –
ESV02491 (0.01%) 2.8 0.14 – / –
ESV02503 (0.007%) 1.6 0.28 – / –
ESV02602 (0.007%) 0.7 0.55 – / –
ESV02644 (0.006%) 0.0 0.99 – / –
ESV02649 (0.005%) 0.4 0.67 – / –
ESV02725 (0.006%) 2.9 0.13 N / –
ESV02788 (0.009%) 6.0 0.04 * – / –
ESV02875 (0.005%) 5.0 0.05 . N / –
ESV02977 (0.008%) 1.9 0.23 – / –
ESV03059 (0.02%) 1.0 0.44 – / –
ESV03086 (0.008%) 0.1 0.89 – / –
ESV03169 (0.01%) 5.2 0.05 * – / –
ESV03174 (0.008%) 0.8 0.48 – / –
ESV03181 (0.007%) 0.1 0.87 – / –
ESV03234 (0.008%) 0.5 0.64 – / –
ESV03244 (0.005%) 5.5 0.04 * – / –
ESV03255 (0.01%) 0.6 0.60 – / –
ESV03284 (0.006%) 2.5 0.16 – / –
ESV03302 (0.004%) 0.5 0.60 – / –
ESV03383 (0.004%) 9.1 0.02 * – / –
ESV03393 (0.004%) 0.3 0.74 – / –
ESV03559 (0.004%) 0.4 0.70 – / –
ESV03584 (0.007%) 1.9 0.23 – / –
ESV03594 (0.006%) 0.3 0.75 N / –
ESV03612 (0.004%) 0.8 0.50 – / –
ESV03679 (0.008%) 1.0 0.42 N / –
ESV03710 (0.005%) 0.2 0.80 N / –
ESV03714 (0.006%) 1.6 0.28 N / –
ESV03718 (0.006%) 0.6 0.56 – / –
ESV03747 (0.005%) 0.8 0.48 – / –
ESV03765 (0.004%) 0.9 0.47 N / –
ESV03782 (0.005%) 1.0 0.43 – / –

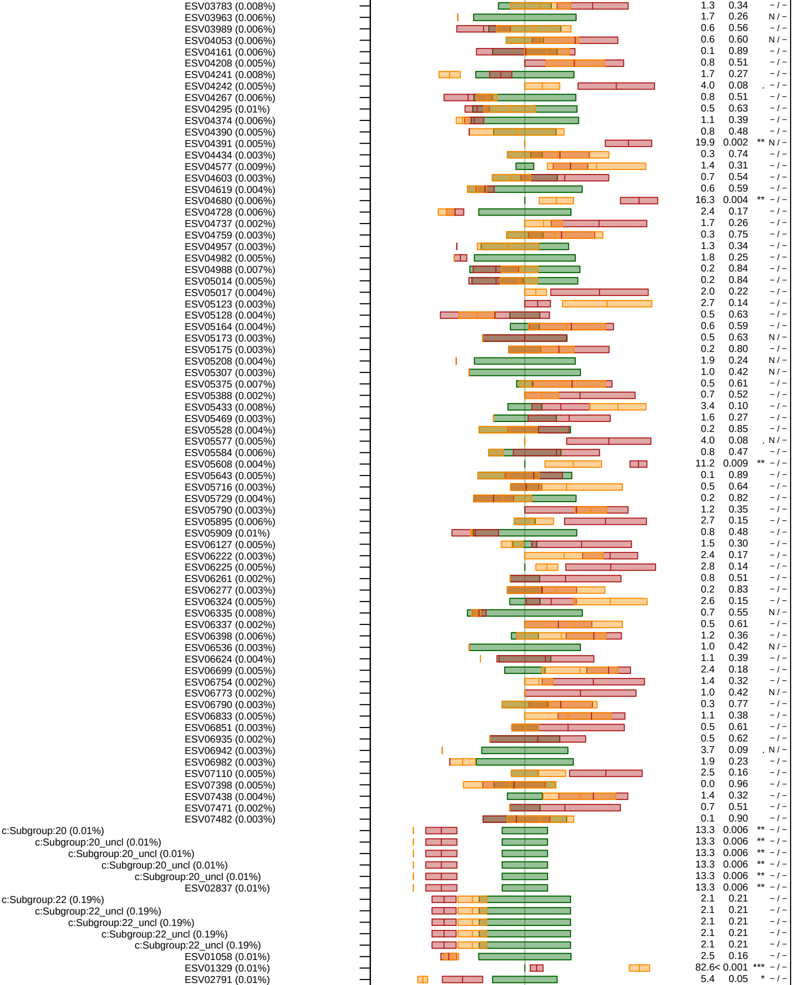
ESV03783 (0.008%) 1.3 0.34 - / -
ESV03963 (0.006%) 1.7 0.26 N / -
ESV03989 (0.006%) 0.6 0.56 - / -
ESV04053 (0.006%) 0.6 0.60 N / -
ESV04161 (0.006%) 0.1 0.89 - / -
ESV04208 (0.005%) 0.8 0.51 - / -
ESV04241 (0.008%) 1.7 0.27 - / -
ESV04242 (0.005%) 4.0 0.08 . - / -
ESV04267 (0.006%) 0.8 0.51 - / -
ESV04295 (0.01%) 0.5 0.63 - / -
ESV04374 (0.006%) 1.1 0.39 - / -
ESV04390 (0.005%) 0.8 0.48 - / -
ESV04391 (0.005%) 19.9 0.002 ** N / -
ESV04434 (0.003%) 0.3 0.74 - / -
ESV04577 (0.009%) 1.4 0.31 - / -
ESV04603 (0.003%) 0.7 0.54 - / -
ESV04619 (0.004%) 0.6 0.59 - / -
ESV04680 (0.006%) 16.3 0.004 ** - / -
ESV04728 (0.006%) 2.4 0.17 - / -
ESV04737 (0.002%) 1.7 0.26 - / -
ESV04759 (0.003%) 0.3 0.75 - / -
ESV04957 (0.003%) 1.3 0.34 - / -
ESV04982 (0.005%) 1.8 0.25 - / -
ESV04988 (0.007%) 0.2 0.84 - / -
ESV05014 (0.005%) 0.2 0.84 - / -
ESV05017 (0.004%) 2.0 0.22 - / -
ESV05123 (0.003%) 2.7 0.14 - / -
ESV05128 (0.004%) 0.5 0.63 - / -
ESV05164 (0.004%) 0.6 0.59 - / -
ESV05173 (0.003%) 0.5 0.63 N / -
ESV05175 (0.003%) 0.2 0.80 - / -
ESV05208 (0.004%) 1.9 0.24 N / -
ESV05307 (0.003%) 1.0 0.42 N / -
ESV05375 (0.007%) 0.5 0.61 - / -
ESV05388 (0.002%) 0.7 0.52 - / -
ESV05433 (0.008%) 3.4 0.10 - / -
ESV05469 (0.003%) 1.6 0.27 - / -
ESV05528 (0.004%) 0.2 0.85 - / -
ESV05577 (0.005%) 4.0 0.08 . N / -
ESV05584 (0.006%) 0.8 0.47 - / -
ESV05608 (0.004%) 11.2 0.009 ** - / -
ESV05643 (0.005%) 0.1 0.89 - / -
ESV05716 (0.003%) 0.5 0.64 - / -
ESV05729 (0.004%) 0.2 0.82 - / -
ESV05790 (0.003%) 1.2 0.35 - / -
ESV05895 (0.006%) 2.7 0.15 - / -
ESV05909 (0.01%) 0.8 0.48 - / -
ESV06127 (0.005%) 1.5 0.30 - / -
ESV06222 (0.003%) 2.4 0.17 - / -
ESV06225 (0.005%) 2.8 0.14 - / -
ESV06261 (0.002%) 0.8 0.51 - / -
ESV06277 (0.003%) 0.2 0.83 - / -
ESV06324 (0.005%) 2.6 0.15 - / -
ESV06335 (0.008%) 0.7 0.55 N / -
ESV06337 (0.002%) 0.5 0.61 - / -
ESV06398 (0.006%) 1.2 0.36 - / -
ESV06536 (0.003%) 1.0 0.42 N / -
ESV06624 (0.004%) 1.1 0.39 - / -
ESV06699 (0.005%) 2.4 0.18 - / -
ESV06754 (0.002%) 1.4 0.32 - / -
ESV06773 (0.002%) 1.0 0.42 N / -
ESV06790 (0.003%) 0.3 0.77 - / -
ESV06833 (0.005%) 1.1 0.38 - / -
ESV06851 (0.003%) 0.5 0.61 - / -
ESV06935 (0.002%) 0.5 0.62 - / -
ESV06942 (0.003%) 3.7 0.09 . N / -
ESV06982 (0.003%) 1.9 0.23 - / -
ESV07110 (0.005%) 2.5 0.16 - / -
ESV07398 (0.005%) 0.0 0.96 - / -
ESV07438 (0.004%) 1.4 0.32 - / -
ESV07471 (0.002%) 0.7 0.51 - / -
ESV07482 (0.003%) 0.1 0.90 - / -
c:Subgroup:20 (0.01%) 13.3 0.006 ** - / -
c:Subgroup:20_uncl (0.01%) 13.3 0.006 ** - / -
c:Subgroup:20_uncl (0.01%) 13.3 0.006 ** - / -
c:Subgroup:20_uncl (0.01%) 13.3 0.006 ** - / -
c:Subgroup:20_uncl (0.01%) 13.3 0.006 ** - / -
ESV02837 (0.01%) 13.3 0.006 ** - / -
c:Subgroup:22 (0.19%) 2.1 0.21 - / -
c:Subgroup:22_uncl (0.19%) 2.1 0.21 - / -
c:Subgroup:22_uncl (0.19%) 2.1 0.21 - / -
c:Subgroup:22_uncl (0.19%) 2.1 0.21 - / -
c:Subgroup:22_uncl (0.19%) 2.1 0.21 - / -
ESV01058 (0.01%) 2.5 0.16 - / -
ESV01329 (0.01%) 82.6< 0.001 *** - / -
ESV02791 (0.01%) 5.4 0.05 * - / -

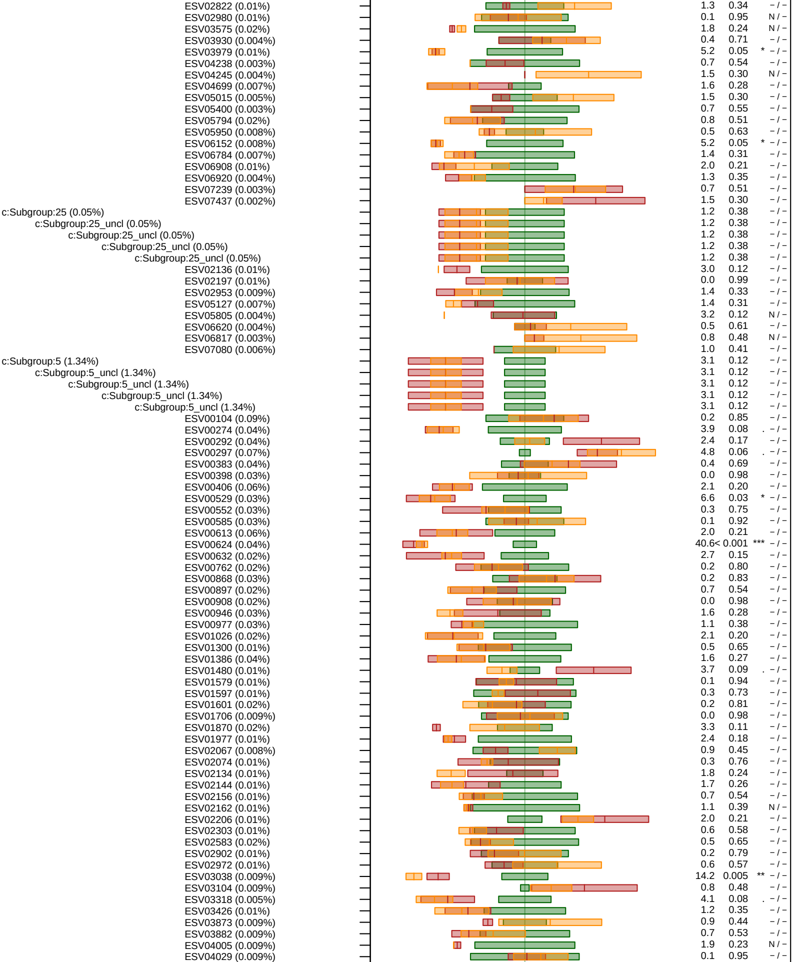
ESV02822 (0.01%) 1.3 0.34 – / –
ESV02980 (0.01%) 0.1 0.95 N / –
ESV03575 (0.02%) 1.8 0.24 N / –
ESV03930 (0.004%) 0.4 0.71 – / –
ESV03979 (0.01%) 5.2 0.05 * – / –
ESV04238 (0.003%) 0.7 0.54 – / –
ESV04245 (0.004%) 1.5 0.30 N / –
ESV04699 (0.007%) 1.6 0.28 – / –
ESV05015 (0.005%) 1.5 0.30 – / –
ESV05400 (0.003%) 0.7 0.55 – / –
ESV05794 (0.02%) 0.8 0.51 – / –
ESV05950 (0.008%) 0.5 0.63 – / –
ESV06152 (0.008%) 5.2 0.05 * – / –
ESV06784 (0.007%) 1.4 0.31 – / –
ESV06908 (0.01%) 2.0 0.21 – / –
ESV06920 (0.004%) 1.3 0.35 – / –
ESV07239 (0.003%) 0.7 0.51 – / –
ESV07437 (0.002%) 1.5 0.30 – / –
c:Subgroup:25 (0.05%) 1.2 0.38 – / –
c:Subgroup:25_uncl (0.05%) 1.2 0.38 – / –
c:Subgroup:25_uncl (0.05%) 1.2 0.38 – / –
c:Subgroup:25_uncl (0.05%) 1.2 0.38 – / –
c:Subgroup:25_uncl (0.05%) 1.2 0.38 – / –
ESV02136 (0.01%) 3.0 0.12 – / –
ESV02197 (0.01%) 0.0 0.99 – / –
ESV02953 (0.009%) 1.4 0.33 – / –
ESV05127 (0.007%) 1.4 0.31 – / –
ESV05805 (0.004%) 3.2 0.12 N / –
ESV06620 (0.004%) 0.5 0.61 – / –
ESV06817 (0.003%) 0.8 0.48 N / –
ESV07080 (0.006%) 1.0 0.41 – / –
c:Subgroup:5 (1.34%) 3.1 0.12 – / –
c:Subgroup:5_uncl (1.34%) 3.1 0.12 – / –
c:Subgroup:5_uncl (1.34%) 3.1 0.12 – / –
c:Subgroup:5_uncl (1.34%) 3.1 0.12 – / –
c:Subgroup:5_uncl (1.34%) 3.1 0.12 – / –
ESV00104 (0.09%) 0.2 0.85 – / –
ESV00274 (0.04%) 3.9 0.08 . – / –
ESV00292 (0.04%) 2.4 0.17 – / –
ESV00297 (0.07%) 4.8 0.06 . – / –
ESV00383 (0.04%) 0.4 0.69 – / –
ESV00398 (0.03%) 0.0 0.98 – / –
ESV00406 (0.06%) 2.1 0.20 – / –
ESV00529 (0.03%) 6.6 0.03 * – / –
ESV00552 (0.03%) 0.3 0.75 – / –
ESV00585 (0.03%) 0.1 0.92 – / –
ESV00613 (0.06%) 2.0 0.21 – / –
ESV00624 (0.04%) 40.6 < 0.001 *** – / –
ESV00632 (0.02%) 2.7 0.15 – / –
ESV00762 (0.02%) 0.2 0.80 – / –
ESV00868 (0.03%) 0.2 0.83 – / –
ESV00897 (0.02%) 0.7 0.54 – / –
ESV00908 (0.02%) 0.0 0.98 – / –
ESV00946 (0.03%) 1.6 0.28 – / –
ESV00977 (0.03%) 1.1 0.38 – / –
ESV01026 (0.02%) 2.1 0.20 – / –
ESV01300 (0.01%) 0.5 0.65 – / –
ESV01386 (0.04%) 1.6 0.27 – / –
ESV01480 (0.01%) 3.7 0.09 . – / –
ESV01579 (0.01%) 0.1 0.94 – / –
ESV01597 (0.01%) 0.3 0.73 – / –
ESV01601 (0.02%) 0.2 0.81 – / –
ESV01706 (0.009%) 0.0 0.98 – / –
ESV01870 (0.02%) 3.3 0.11 – / –
ESV01977 (0.01%) 2.4 0.18 – / –
ESV02067 (0.008%) 0.9 0.45 – / –
ESV02074 (0.01%) 0.3 0.76 – / –
ESV02134 (0.01%) 1.8 0.24 – / –
ESV02144 (0.01%) 1.7 0.26 – / –
ESV02156 (0.01%) 0.7 0.54 – / –
ESV02162 (0.01%) 1.1 0.39 N / –
ESV02206 (0.01%) 2.0 0.21 – / –
ESV02303 (0.01%) 0.6 0.58 – / –
ESV02583 (0.02%) 0.5 0.65 – / –
ESV02902 (0.01%) 0.2 0.79 – / –
ESV02972 (0.01%) 0.6 0.57 – / –
ESV03038 (0.009%) 14.2 0.005 ** – / –
ESV03104 (0.009%) 0.8 0.48 – / –
ESV03318 (0.005%) 4.1 0.08 . – / –
ESV03426 (0.01%) 1.2 0.35 – / –
ESV03873 (0.009%) 0.9 0.44 – / –
ESV03882 (0.009%) 0.7 0.53 – / –
ESV04005 (0.009%) 1.9 0.23 N / –
ESV04029 (0.009%) 0.1 0.95 – / –

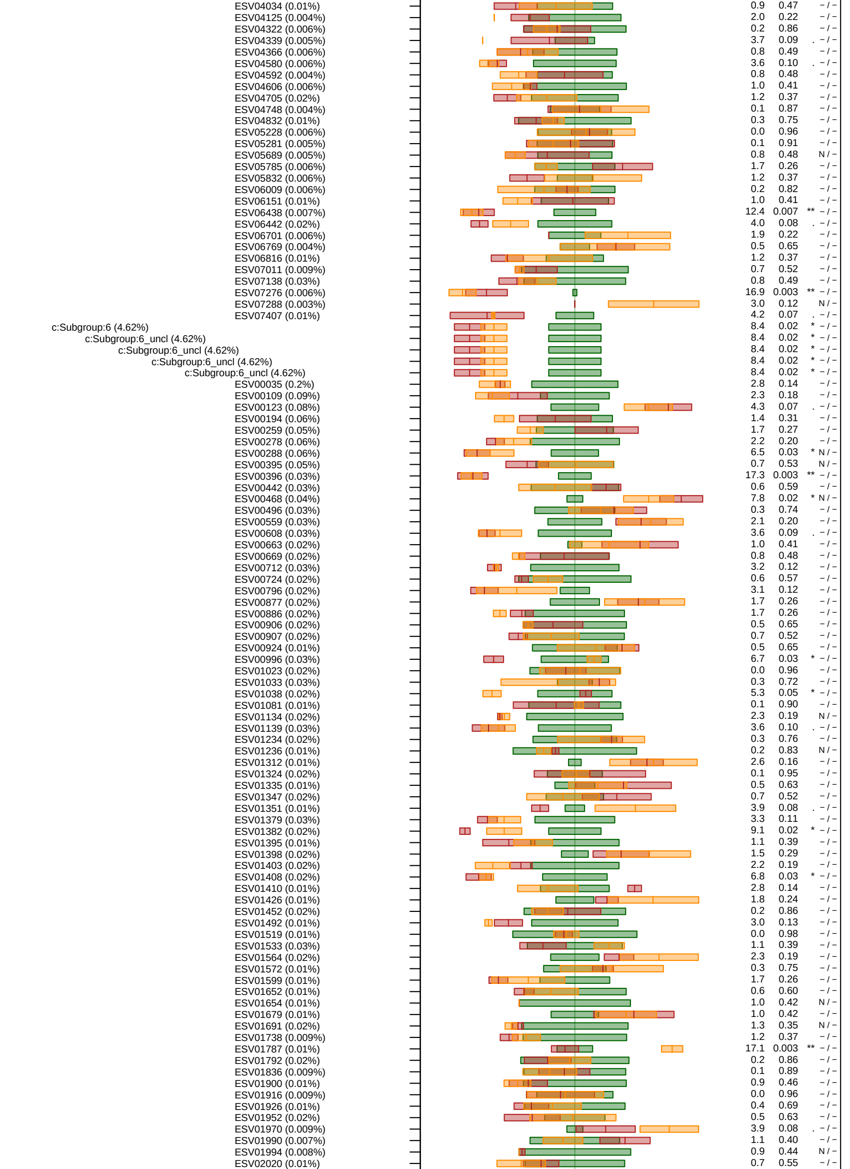
ESV04034 (0.01%) 0.9 0.47 – / –
ESV04125 (0.004%) 2.0 0.22 – / –
ESV04322 (0.006%) 0.2 0.86 – / –
ESV04339 (0.005%) 3.7 0.09 . – / –
ESV04366 (0.006%) 0.8 0.49 – / –
ESV04580 (0.006%) 3.6 0.10 . – / –
ESV04592 (0.004%) 0.8 0.48 – / –
ESV04606 (0.006%) 1.0 0.41 – / –
ESV04705 (0.02%) 1.2 0.37 – / –
ESV04748 (0.004%) 0.1 0.87 – / –
ESV04832 (0.01%) 0.3 0.75 – / –
ESV05228 (0.006%) 0.0 0.96 – / –
ESV05281 (0.005%) 0.1 0.91 – / –
ESV05689 (0.005%) 0.8 0.48 N / –
ESV05785 (0.006%) 1.7 0.26 – / –
ESV05832 (0.006%) 1.2 0.37 – / –
ESV06009 (0.006%) 0.2 0.82 – / –
ESV06151 (0.01%) 1.0 0.41 – / –
ESV06438 (0.007%) 12.4 0.007 ** – / –
ESV06442 (0.02%) 4.0 0.08 . – / –
ESV06701 (0.006%) 1.9 0.22 – / –
ESV06769 (0.004%) 0.5 0.65 – / –
ESV06816 (0.01%) 1.2 0.37 – / –
ESV07011 (0.009%) 0.7 0.52 – / –
ESV07138 (0.03%) 0.8 0.49 – / –
ESV07276 (0.006%) 16.9 0.003 ** – / –
ESV07288 (0.003%) 3.0 0.12 N / –
ESV07407 (0.01%) 4.2 0.07 . – / –
c:Subgroup:6 (4.62%) 8.4 0.02 * – / –
c:Subgroup:6_uncl (4.62%) 8.4 0.02 * – / –
c:Subgroup:6_uncl (4.62%) 8.4 0.02 * – / –
c:Subgroup:6_uncl (4.62%) 8.4 0.02 * – / –
c:Subgroup:6_uncl (4.62%) 8.4 0.02 * – / –
ESV00035 (0.2%) 2.8 0.14 – / –
ESV00109 (0.09%) 2.3 0.18 – / –
ESV00123 (0.08%) 4.3 0.07 . – / –
ESV00194 (0.06%) 1.4 0.31 – / –
ESV00259 (0.05%) 1.7 0.27 – / –
ESV00278 (0.06%) 2.2 0.20 – / –
ESV00288 (0.06%) 6.5 0.03 * N / –
ESV00395 (0.05%) 0.7 0.53 N / –
ESV00396 (0.03%) 17.3 0.003 ** – / –
ESV00442 (0.03%) 0.6 0.59 – / –
ESV00468 (0.04%) 7.8 0.02 * N / –
ESV00496 (0.03%) 0.3 0.74 – / –
ESV00559 (0.03%) 2.1 0.20 – / –
ESV00608 (0.03%) 3.6 0.09 . – / –
ESV00663 (0.02%) 1.0 0.41 – / –
ESV00669 (0.02%) 0.8 0.48 – / –
ESV00712 (0.03%) 3.2 0.12 – / –
ESV00724 (0.02%) 0.6 0.57 – / –
ESV00796 (0.02%) 3.1 0.12 – / –
ESV00877 (0.02%) 1.7 0.26 – / –
ESV00886 (0.02%) 1.7 0.26 – / –
ESV00906 (0.02%) 0.5 0.65 – / –
ESV00907 (0.02%) 0.7 0.52 – / –
ESV00924 (0.01%) 0.5 0.65 – / –
ESV00996 (0.03%) 6.7 0.03 * – / –
ESV01023 (0.02%) 0.0 0.96 – / –
ESV01033 (0.03%) 0.3 0.72 – / –
ESV01038 (0.02%) 5.3 0.05 * – / –
ESV01081 (0.01%) 0.1 0.90 – / –
ESV01134 (0.02%) 2.3 0.19 N / –
ESV01139 (0.03%) 3.6 0.10 . – / –
ESV01234 (0.02%) 0.3 0.76 – / –
ESV01236 (0.01%) 0.2 0.83 N / –
ESV01312 (0.01%) 2.6 0.16 – / –
ESV01324 (0.02%) 0.1 0.95 – / –
ESV01335 (0.01%) 0.5 0.63 – / –
ESV01347 (0.02%) 0.7 0.52 – / –
ESV01351 (0.01%) 3.9 0.08 . – / –
ESV01379 (0.03%) 3.3 0.11 – / –
ESV01382 (0.02%) 9.1 0.02 * – / –
ESV01395 (0.01%) 1.1 0.39 – / –
ESV01398 (0.02%) 1.5 0.29 – / –
ESV01403 (0.02%) 2.2 0.19 – / –
ESV01408 (0.02%) 6.8 0.03 * – / –
ESV01410 (0.01%) 2.8 0.14 – / –
ESV01426 (0.01%) 1.8 0.24 – / –
ESV01452 (0.02%) 0.2 0.86 – / –
ESV01492 (0.01%) 3.0 0.13 – / –
ESV01519 (0.01%) 0.0 0.98 – / –
ESV01533 (0.03%) 1.1 0.39 – / –
ESV01564 (0.02%) 2.3 0.19 – / –
ESV01572 (0.01%) 0.3 0.75 – / –
ESV01599 (0.01%) 1.7 0.26 – / –
ESV01652 (0.01%) 0.6 0.60 – / –
ESV01654 (0.01%) 1.0 0.42 N / –
ESV01679 (0.01%) 1.0 0.42 – / –
ESV01691 (0.02%) 1.3 0.35 N / –
ESV01738 (0.009%) 1.2 0.37 – / –
ESV01787 (0.01%) 17.1 0.003 ** – / –
ESV01792 (0.02%) 0.2 0.86 – / –
ESV01836 (0.009%) 0.1 0.89 – / –
ESV01900 (0.01%) 0.9 0.46 – / –
ESV01916 (0.009%) 0.0 0.96 – / –
ESV01926 (0.01%) 0.4 0.69 – / –
ESV01952 (0.02%) 0.5 0.63 – / –
ESV01970 (0.009%) 3.9 0.08 . – / –
ESV01990 (0.007%) 1.1 0.40 – / –
ESV01994 (0.008%) 0.9 0.44 N / –
ESV02020 (0.01%) 0.7 0.55 – / –

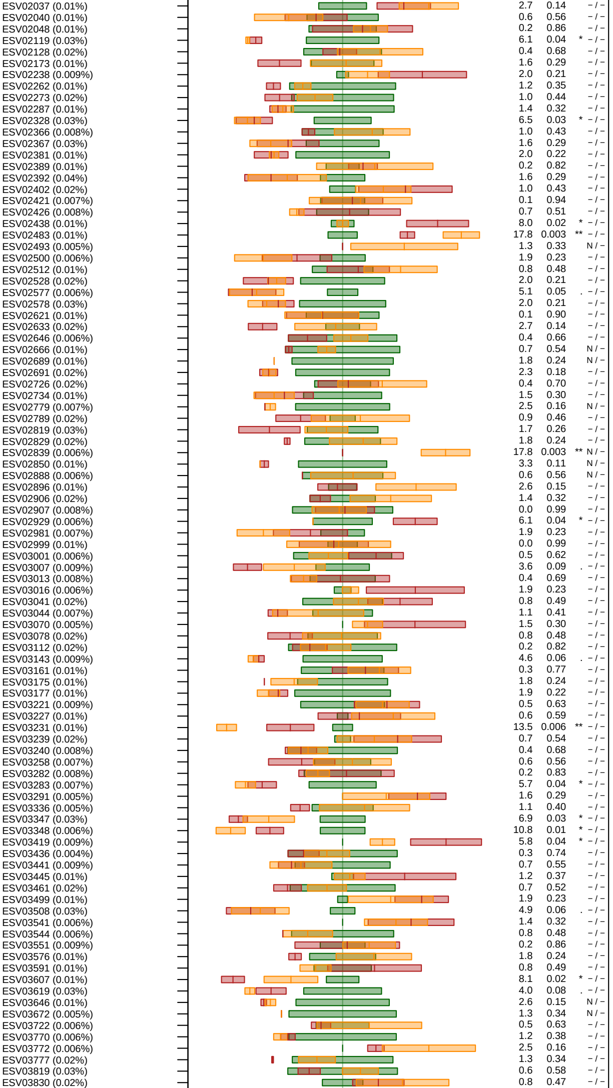
ESV02037 (0.01%) 2.7 0.14 - / -
ESV02040 (0.01%) 0.6 0.56 - / -
ESV02048 (0.01%) 0.2 0.86 - / -
ESV02119 (0.03%) 6.1 0.04 * - / -
ESV02128 (0.02%) 0.4 0.68 - / -
ESV02173 (0.01%) 1.6 0.29 - / -
ESV02238 (0.009%) 2.0 0.21 - / -
ESV02262 (0.01%) 1.2 0.35 - / -
ESV02273 (0.02%) 1.0 0.44 - / -
ESV02287 (0.01%) 1.4 0.32 - / -
ESV02328 (0.03%) 6.5 0.03 * - / -
ESV02366 (0.008%) 1.0 0.43 - / -
ESV02367 (0.03%) 1.6 0.29 - / -
ESV02381 (0.01%) 2.0 0.22 - / -
ESV02389 (0.01%) 0.2 0.82 - / -
ESV02392 (0.04%) 1.6 0.29 - / -
ESV02402 (0.02%) 1.0 0.43 - / -
ESV02421 (0.007%) 0.1 0.94 - / -
ESV02426 (0.008%) 0.7 0.51 - / -
ESV02438 (0.01%) 8.0 0.02 * - / -
ESV02483 (0.01%) 17.8 0.003 ** - / -
ESV02493 (0.005%) 1.3 0.33 N / -
ESV02500 (0.006%) 1.9 0.23 - / -
ESV02512 (0.01%) 0.8 0.48 - / -
ESV02528 (0.02%) 2.0 0.21 - / -
ESV02577 (0.006%) 5.1 0.05 . - / -
ESV02578 (0.03%) 2.0 0.21 - / -
ESV02621 (0.01%) 0.1 0.90 - / -
ESV02633 (0.02%) 2.7 0.14 - / -
ESV02646 (0.006%) 0.4 0.66 - / -
ESV02666 (0.01%) 0.7 0.54 N / -
ESV02689 (0.01%) 1.8 0.24 N / -
ESV02691 (0.02%) 2.3 0.18 - / -
ESV02726 (0.02%) 0.4 0.70 - / -
ESV02734 (0.01%) 1.5 0.30 - / -
ESV02779 (0.007%) 2.5 0.16 N / -
ESV02789 (0.02%) 0.9 0.46 - / -
ESV02819 (0.03%) 1.7 0.26 - / -
ESV02829 (0.02%) 1.8 0.24 - / -
ESV02839 (0.006%) 17.8 0.003 ** N / -
ESV02850 (0.01%) 3.3 0.11 N / -
ESV02888 (0.006%) 0.6 0.56 N / -
ESV02896 (0.01%) 2.6 0.15 - / -
ESV02906 (0.02%) 1.4 0.32 - / -
ESV02907 (0.008%) 0.0 0.99 - / -
ESV02929 (0.006%) 6.1 0.04 * - / -
ESV02981 (0.007%) 1.9 0.23 - / -
ESV02999 (0.01%) 0.0 0.99 - / -
ESV03001 (0.006%) 0.5 0.62 - / -
ESV03007 (0.009%) 3.6 0.09 . - / -
ESV03013 (0.008%) 0.4 0.69 - / -
ESV03016 (0.006%) 1.9 0.23 - / -
ESV03041 (0.02%) 0.8 0.49 - / -
ESV03044 (0.007%) 1.1 0.41 - / -
ESV03070 (0.005%) 1.5 0.30 - / -
ESV03078 (0.02%) 0.8 0.48 - / -
ESV03112 (0.02%) 0.2 0.82 - / -
ESV03143 (0.009%) 4.6 0.06 . - / -
ESV03161 (0.01%) 0.3 0.77 - / -
ESV03175 (0.01%) 1.8 0.24 - / -
ESV03177 (0.01%) 1.9 0.22 - / -
ESV03221 (0.009%) 0.5 0.63 - / -
ESV03227 (0.01%) 0.6 0.59 - / -
ESV03231 (0.01%) 13.5 0.006 ** - / -
ESV03239 (0.02%) 0.7 0.54 - / -
ESV03240 (0.008%) 0.4 0.68 - / -
ESV03258 (0.007%) 0.6 0.56 - / -
ESV03282 (0.008%) 0.2 0.83 - / -
ESV03283 (0.007%) 5.7 0.04 * - / -
ESV03291 (0.005%) 1.6 0.29 - / -
ESV03336 (0.005%) 1.1 0.40 - / -
ESV03347 (0.03%) 6.9 0.03 * - / -
ESV03348 (0.006%) 10.8 0.01 * - / -
ESV03419 (0.009%) 5.8 0.04 * - / -
ESV03436 (0.004%) 0.3 0.74 - / -
ESV03441 (0.009%) 0.7 0.55 - / -
ESV03445 (0.01%) 1.2 0.37 - / -
ESV03461 (0.02%) 0.7 0.52 - / -
ESV03499 (0.01%) 1.9 0.23 - / -
ESV03508 (0.03%) 4.9 0.06 . - / -
ESV03541 (0.006%) 1.4 0.32 - / -
ESV03544 (0.006%) 0.8 0.48 - / -
ESV03551 (0.009%) 0.2 0.86 - / -
ESV03576 (0.01%) 1.8 0.24 - / -
ESV03591 (0.01%) 0.8 0.49 - / -
ESV03607 (0.01%) 8.1 0.02 * - / -
ESV03619 (0.03%) 4.0 0.08 . - / -
ESV03646 (0.01%) 2.6 0.15 N / -
ESV03672 (0.005%) 1.3 0.34 N / -
ESV03722 (0.006%) 0.5 0.63 - / -
ESV03770 (0.006%) 1.2 0.38 - / -
ESV03772 (0.006%) 2.5 0.16 - / -
ESV03777 (0.02%) 1.3 0.34 - / -
ESV03819 (0.03%) 0.6 0.58 - / -
ESV03830 (0.02%) 0.8 0.47 - / -

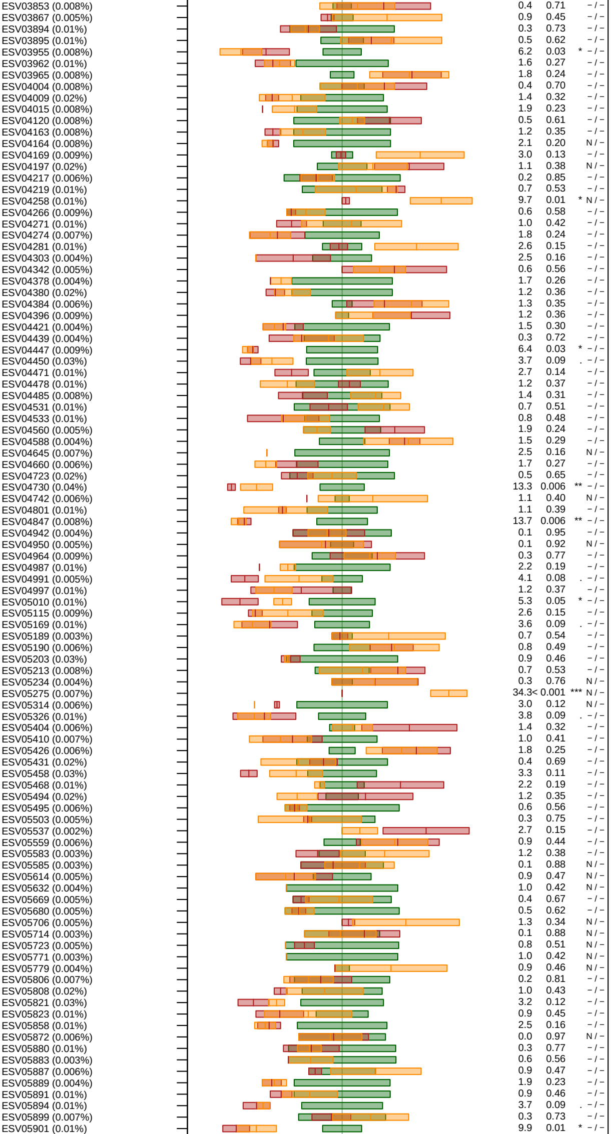

ESV03853 (0.008%) 0.4 0.71 - / -
ESV03867 (0.005%) 0.9 0.45 - / -
ESV03894 (0.01%) 0.3 0.73 - / -
ESV03895 (0.01%) 0.5 0.62 - / -
ESV03955 (0.008%) 6.2 0.03 * - / -
ESV03962 (0.01%) 1.6 0.27 - / -
ESV03965 (0.008%) 1.8 0.24 - / -
ESV04004 (0.008%) 0.4 0.70 - / -
ESV04009 (0.02%) 1.4 0.32 - / -
ESV04015 (0.008%) 1.9 0.23 - / -
ESV04120 (0.008%) 0.5 0.61 - / -
ESV04163 (0.008%) 1.2 0.35 - / -
ESV04164 (0.008%) 2.1 0.20 N / -
ESV04169 (0.009%) 3.0 0.13 - / -
ESV04197 (0.02%) 1.1 0.38 N / -
ESV04217 (0.006%) 0.2 0.85 - / -
ESV04219 (0.01%) 0.7 0.53 - / -
ESV04258 (0.01%) 9.7 0.01 * N / -
ESV04266 (0.009%) 0.6 0.58 - / -
ESV04271 (0.01%) 1.0 0.42 - / -
ESV04274 (0.007%) 1.8 0.24 - / -
ESV04281 (0.01%) 2.6 0.15 - / -
ESV04303 (0.004%) 2.5 0.16 - / -
ESV04342 (0.005%) 0.6 0.56 - / -
ESV04378 (0.004%) 1.7 0.26 - / -
ESV04380 (0.02%) 1.2 0.36 - / -
ESV04384 (0.006%) 1.3 0.35 - / -
ESV04396 (0.009%) 1.2 0.36 - / -
ESV04421 (0.004%) 1.5 0.30 - / -
ESV04439 (0.004%) 0.3 0.72 - / -
ESV04447 (0.009%) 6.4 0.03 * - / -
ESV04450 (0.03%) 3.7 0.09 . - / -
ESV04471 (0.01%) 2.7 0.14 - / -
ESV04478 (0.01%) 1.2 0.37 - / -
ESV04485 (0.008%) 1.4 0.31 - / -
ESV04531 (0.01%) 0.7 0.51 - / -
ESV04533 (0.01%) 0.8 0.48 - / -
ESV04560 (0.005%) 1.9 0.24 - / -
ESV04588 (0.004%) 1.5 0.29 - / -
ESV04645 (0.007%) 2.5 0.16 N / -
ESV04660 (0.006%) 1.7 0.27 - / -
ESV04723 (0.02%) 0.5 0.65 - / -
ESV04730 (0.04%) 13.3 0.006 ** - / -
ESV04742 (0.006%) 1.1 0.40 N / -
ESV04801 (0.01%) 1.1 0.39 - / -
ESV04847 (0.008%) 13.7 0.006 ** - / -
ESV04942 (0.004%) 0.1 0.95 - / -
ESV04950 (0.005%) 0.1 0.92 N / -
ESV04964 (0.009%) 0.3 0.77 - / -
ESV04987 (0.01%) 2.2 0.19 - / -
ESV04991 (0.005%) 4.1 0.08 . - / -
ESV04997 (0.01%) 1.2 0.37 - / -
ESV05010 (0.01%) 5.3 0.05 * - / -
ESV05115 (0.009%) 2.6 0.15 - / -
ESV05169 (0.01%) 3.6 0.09 . - / -
ESV05189 (0.003%) 0.7 0.54 - / -
ESV05190 (0.006%) 0.8 0.49 - / -
ESV05203 (0.03%) 0.9 0.46 - / -
ESV05213 (0.008%) 0.7 0.53 - / -
ESV05234 (0.004%) 0.3 0.76 N / -
ESV05275 (0.007%) 34.3 < 0.001 *** N / -
ESV05314 (0.006%) 3.0 0.12 N / -
ESV05326 (0.01%) 3.8 0.09 . - / -
ESV05404 (0.006%) 1.4 0.32 - / -
ESV05410 (0.007%) 1.0 0.41 - / -
ESV05426 (0.006%) 1.8 0.25 - / -
ESV05431 (0.02%) 0.4 0.69 - / -
ESV05458 (0.03%) 3.3 0.11 - / -
ESV05468 (0.01%) 2.2 0.19 - / -
ESV05494 (0.02%) 1.2 0.35 - / -
ESV05495 (0.006%) 0.6 0.56 - / -
ESV05503 (0.005%) 0.3 0.75 - / -
ESV05537 (0.002%) 2.7 0.15 - / -
ESV05559 (0.006%) 0.9 0.44 - / -
ESV05583 (0.003%) 1.2 0.38 - / -
ESV05585 (0.003%) 0.1 0.88 N / -
ESV05614 (0.005%) 0.9 0.47 N / -
ESV05632 (0.004%) 1.0 0.42 N / -
ESV05669 (0.005%) 0.4 0.67 - / -
ESV05680 (0.005%) 0.5 0.62 - / -
ESV05706 (0.005%) 1.3 0.34 N / -
ESV05714 (0.003%) 0.1 0.88 N / -
ESV05723 (0.005%) 0.8 0.51 N / -
ESV05771 (0.003%) 1.0 0.42 N / -
ESV05779 (0.004%) 0.9 0.46 N / -
ESV05806 (0.007%) 0.2 0.81 - / -
ESV05808 (0.02%) 1.0 0.43 - / -
ESV05821 (0.03%) 3.2 0.12 - / -
ESV05823 (0.01%) 0.9 0.45 - / -
ESV05858 (0.01%) 2.5 0.16 - / -
ESV05872 (0.006%) 0.0 0.97 N / -
ESV05880 (0.01%) 0.3 0.77 - / -
ESV05883 (0.003%) 0.6 0.56 - / -
ESV05887 (0.006%) 0.9 0.47 - / -
ESV05889 (0.004%) 1.9 0.23 - / -
ESV05891 (0.01%) 0.9 0.46 - / -
ESV05894 (0.01%) 3.7 0.09 . - / -
ESV05899 (0.007%) 0.3 0.73 - / -
ESV05901 (0.01%) 9.9 0.01 * - / -

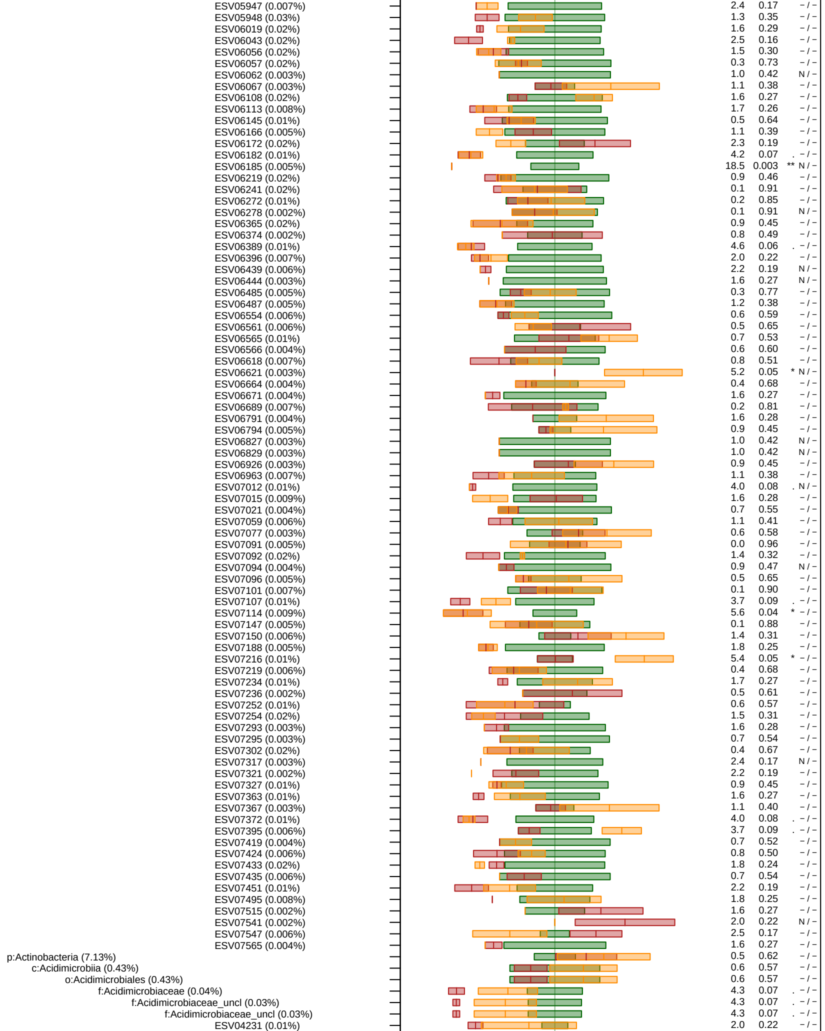
ESV05947 (0.007%) 2.4 0.17 - / -
ESV05948 (0.03%) 1.3 0.35 - / -
ESV06019 (0.02%) 1.6 0.29 - / -
ESV06043 (0.02%) 2.5 0.16 - / -
ESV06056 (0.02%) 1.5 0.30 - / -
ESV06057 (0.02%) 0.3 0.73 - / -
ESV06062 (0.003%) 1.0 0.42 N / -
ESV06067 (0.003%) 1.1 0.38 - / -
ESV06108 (0.02%) 1.6 0.27 - / -
ESV06113 (0.008%) 1.7 0.26 - / -
ESV06145 (0.01%) 0.5 0.64 - / -
ESV06166 (0.005%) 1.1 0.39 - / -
ESV06172 (0.02%) 2.3 0.19 - / -
ESV06182 (0.01%) 4.2 0.07 . - / -
ESV06185 (0.005%) 18.5 0.003 ** N / -
ESV06219 (0.02%) 0.9 0.46 - / -
ESV06241 (0.02%) 0.1 0.91 - / -
ESV06272 (0.01%) 0.2 0.85 - / -
ESV06278 (0.002%) 0.1 0.91 N / -
ESV06365 (0.02%) 0.9 0.45 - / -
ESV06374 (0.002%) 0.8 0.49 - / -
ESV06389 (0.01%) 4.6 0.06 . - / -
ESV06396 (0.007%) 2.0 0.22 - / -
ESV06439 (0.006%) 2.2 0.19 N / -
ESV06444 (0.003%) 1.6 0.27 N / -
ESV06485 (0.005%) 0.3 0.77 - / -
ESV06487 (0.005%) 1.2 0.38 - / -
ESV06554 (0.006%) 0.6 0.59 - / -
ESV06561 (0.006%) 0.5 0.65 - / -
ESV06565 (0.01%) 0.7 0.53 - / -
ESV06566 (0.004%) 0.6 0.60 - / -
ESV06618 (0.007%) 0.8 0.51 - / -
ESV06621 (0.003%) 5.2 0.05 * N / -
ESV06664 (0.004%) 0.4 0.68 - / -
ESV06671 (0.004%) 1.6 0.27 - / -
ESV06689 (0.007%) 0.2 0.81 - / -
ESV06791 (0.004%) 1.6 0.28 - / -
ESV06794 (0.005%) 0.9 0.45 - / -
ESV06827 (0.003%) 1.0 0.42 N / -
ESV06829 (0.003%) 1.0 0.42 N / -
ESV06926 (0.003%) 0.9 0.45 - / -
ESV06963 (0.007%) 1.1 0.38 - / -
ESV07012 (0.01%) 4.0 0.08 . N / -
ESV07015 (0.009%) 1.6 0.28 - / -
ESV07021 (0.004%) 0.7 0.55 - / -
ESV07059 (0.006%) 1.1 0.41 - / -
ESV07077 (0.003%) 0.6 0.58 - / -
ESV07091 (0.005%) 0.0 0.96 - / -
ESV07092 (0.02%) 1.4 0.32 - / -
ESV07094 (0.004%) 0.9 0.47 N / -
ESV07096 (0.005%) 0.5 0.65 - / -
ESV07101 (0.007%) 0.1 0.90 - / -
ESV07107 (0.01%) 3.7 0.09 . - / -
ESV07114 (0.009%) 5.6 0.04 * - / -
ESV07147 (0.005%) 0.1 0.88 - / -
ESV07150 (0.006%) 1.4 0.31 - / -
ESV07188 (0.005%) 1.8 0.25 - / -
ESV07216 (0.01%) 5.4 0.05 * - / -
ESV07219 (0.006%) 0.4 0.68 - / -
ESV07234 (0.01%) 1.7 0.27 - / -
ESV07236 (0.002%) 0.5 0.61 - / -
ESV07252 (0.01%) 0.6 0.57 - / -
ESV07254 (0.02%) 1.5 0.31 - / -
ESV07293 (0.003%) 1.6 0.28 - / -
ESV07295 (0.003%) 0.7 0.54 - / -
ESV07302 (0.02%) 0.4 0.67 - / -
ESV07317 (0.003%) 2.4 0.17 N / -
ESV07321 (0.002%) 2.2 0.19 - / -
ESV07327 (0.01%) 0.9 0.45 - / -
ESV07363 (0.01%) 1.6 0.27 - / -
ESV07367 (0.003%) 1.1 0.40 - / -
ESV07372 (0.01%) 4.0 0.08 . - / -
ESV07395 (0.006%) 3.7 0.09 . - / -
ESV07419 (0.004%) 0.7 0.52 - / -
ESV07424 (0.006%) 0.8 0.50 - / -
ESV07433 (0.02%) 1.8 0.24 - / -
ESV07435 (0.006%) 0.7 0.54 - / -
ESV07451 (0.01%) 2.2 0.19 - / -
ESV07495 (0.008%) 1.8 0.25 - / -
ESV07515 (0.002%) 1.6 0.27 - / -
ESV07541 (0.002%) 2.0 0.22 N / -
ESV07547 (0.006%) 2.5 0.17 - / -
ESV07565 (0.004%) 1.6 0.27 - / -
p:Actinobacteria (7.13%) 0.5 0.62 - / -
c:Acidimicrobiia (0.43%) 0.6 0.57 - / -
o:Acidimicrobiales (0.43%) 0.6 0.57 - / -
f:Acidimicrobiaceae (0.04%) 4.3 0.07 . - / -
f:Acidimicrobiaceae_uncl (0.03%) 4.3 0.07 . - / -
f:Acidimicrobiaceae_uncl (0.03%) 4.3 0.07 . - / -
ESV04231 (0.01%) 2.0 0.22 - / -

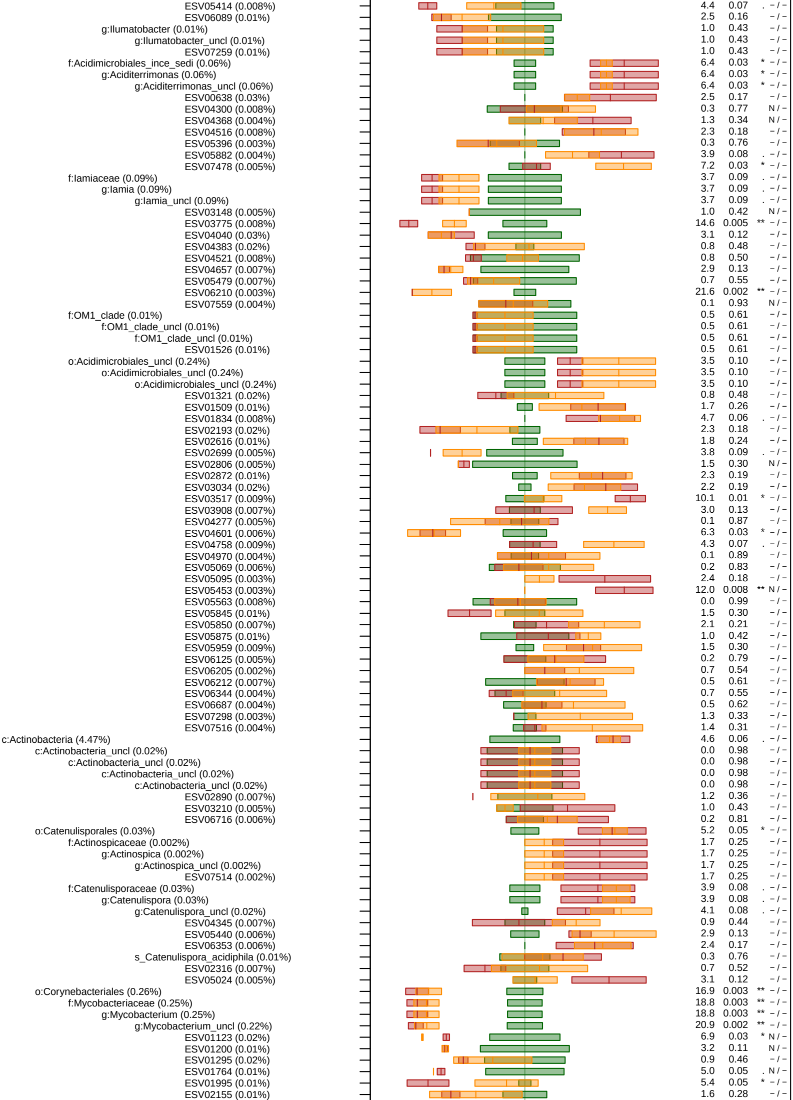
ESV05414 (0.008%) 4.4 0.07 . – / –
ESV06089 (0.01%) 2.5 0.16 – / –
g:Ilumatobacter (0.01%) 1.0 0.43 – / –
g:Ilumatobacter_uncl (0.01%) 1.0 0.43 – / –
ESV07259 (0.01%) 1.0 0.43 – / –
f:Acidimicrobiales_ince_sedi (0.06%) 6.4 0.03 * – / –
g:Aciditerrimonas (0.06%) 6.4 0.03 * – / –
g:Aciditerrimonas_uncl (0.06%) 6.4 0.03 * – / –
ESV00638 (0.03%) 2.5 0.17 – / –
ESV04300 (0.008%) 0.3 0.77 N / –
ESV04368 (0.004%) 1.3 0.34 N / –
ESV04516 (0.008%) 2.3 0.18 – / –
ESV05396 (0.003%) 0.3 0.76 – / –
ESV05882 (0.004%) 3.9 0.08 . – / –
ESV07478 (0.005%) 7.2 0.03 * – / –
f:Iamiaceae (0.09%) 3.7 0.09 . – / –
g:Iamia (0.09%) 3.7 0.09 . – / –
g:Iamia_uncl (0.09%) 3.7 0.09 . – / –
ESV03148 (0.005%) 1.0 0.42 N / –
ESV03775 (0.008%) 14.6 0.005 ** – / –
ESV04040 (0.03%) 3.1 0.12 – / –
ESV04383 (0.02%) 0.8 0.48 – / –
ESV04521 (0.008%) 0.8 0.50 – / –
ESV04657 (0.007%) 2.9 0.13 – / –
ESV05479 (0.007%) 0.7 0.55 – / –
ESV06210 (0.003%) 21.6 0.002 ** – / –
ESV07559 (0.004%) 0.1 0.93 N / –
f:OM1_clade (0.01%) 0.5 0.61 – / –
f:OM1_clade_uncl (0.01%) 0.5 0.61 – / –
f:OM1_clade_uncl (0.01%) 0.5 0.61 – / –
ESV01526 (0.01%) 0.5 0.61 – / –
o:Acidimicrobiales_uncl (0.24%) 3.5 0.10 – / –
o:Acidimicrobiales_uncl (0.24%) 3.5 0.10 – / –
o:Acidimicrobiales_uncl (0.24%) 3.5 0.10 – / –
ESV01321 (0.02%) 0.8 0.48 – / –
ESV01509 (0.01%) 1.7 0.26 – / –
ESV01834 (0.008%) 4.7 0.06 . – / –
ESV02193 (0.02%) 2.3 0.18 – / –
ESV02616 (0.01%) 1.8 0.24 – / –
ESV02699 (0.005%) 3.8 0.09 . – / –
ESV02806 (0.005%) 1.5 0.30 N / –
ESV02872 (0.01%) 2.3 0.19 – / –
ESV03034 (0.02%) 2.2 0.19 – / –
ESV03517 (0.009%) 10.1 0.01 * – / –
ESV03908 (0.007%) 3.0 0.13 – / –
ESV04277 (0.005%) 0.1 0.87 – / –
ESV04601 (0.006%) 6.3 0.03 * – / –
ESV04758 (0.009%) 4.3 0.07 . – / –
ESV04970 (0.004%) 0.1 0.89 – / –
ESV05069 (0.006%) 0.2 0.83 – / –
ESV05095 (0.003%) 2.4 0.18 – / –
ESV05453 (0.003%) 12.0 0.008 ** N / –
ESV05563 (0.008%) 0.0 0.99 – / –
ESV05845 (0.01%) 1.5 0.30 – / –
ESV05850 (0.007%) 2.1 0.21 – / –
ESV05875 (0.01%) 1.0 0.42 – / –
ESV05959 (0.009%) 1.5 0.30 – / –
ESV06125 (0.005%) 0.2 0.79 – / –
ESV06205 (0.002%) 0.7 0.54 – / –
ESV06212 (0.007%) 0.5 0.61 – / –
ESV06344 (0.004%) 0.7 0.55 – / –
ESV06687 (0.004%) 0.5 0.62 – / –
ESV07298 (0.003%) 1.3 0.33 – / –
ESV07516 (0.004%) 1.4 0.31 – / –
c:Actinobacteria (4.47%) 4.6 0.06 . – / –
c:Actinobacteria_uncl (0.02%) 0.0 0.98 – / –
c:Actinobacteria_uncl (0.02%) 0.0 0.98 – / –
c:Actinobacteria_uncl (0.02%) 0.0 0.98 – / –
c:Actinobacteria_uncl (0.02%) 0.0 0.98 – / –
ESV02890 (0.007%) 1.2 0.36 – / –
ESV03210 (0.005%) 1.0 0.43 – / –
ESV06716 (0.006%) 0.2 0.81 – / –
o:Catenulisporales (0.03%) 5.2 0.05 * – / –
f:Actinospicaceae (0.002%) 1.7 0.25 – / –
g:Actinospica (0.002%) 1.7 0.25 – / –
g:Actinospica_uncl (0.002%) 1.7 0.25 – / –
ESV07514 (0.002%) 1.7 0.25 – / –
f:Catenulisporaceae (0.03%) 3.9 0.08 . – / –
g:Catenulispora (0.03%) 3.9 0.08 . – / –
g:Catenulispora_uncl (0.02%) 4.1 0.08 . – / –
ESV04345 (0.007%) 0.9 0.44 – / –
ESV05440 (0.006%) 2.9 0.13 – / –
ESV06353 (0.006%) 2.4 0.17 – / –
s_Catenulispora_acidiphila (0.01%) 0.3 0.76 – / –
ESV02316 (0.007%) 0.7 0.52 – / –
ESV05024 (0.005%) 3.1 0.12 – / –
o:Corynebacteriales (0.26%) 16.9 0.003 ** – / –
f:Mycobacteriaceae (0.25%) 18.8 0.003 ** – / –
g:Mycobacterium (0.25%) 18.8 0.003 ** – / –
g:Mycobacterium_uncl (0.22%) 20.9 0.002 ** – / –
ESV01123 (0.02%) 6.9 0.03 * N / –
ESV01200 (0.01%) 3.2 0.11 N / –
ESV01295 (0.02%) 0.9 0.46 – / –
ESV01764 (0.01%) 5.0 0.05 . N / –
ESV01995 (0.01%) 5.4 0.05 * – / –
ESV02155 (0.01%) 1.6 0.28 – / –

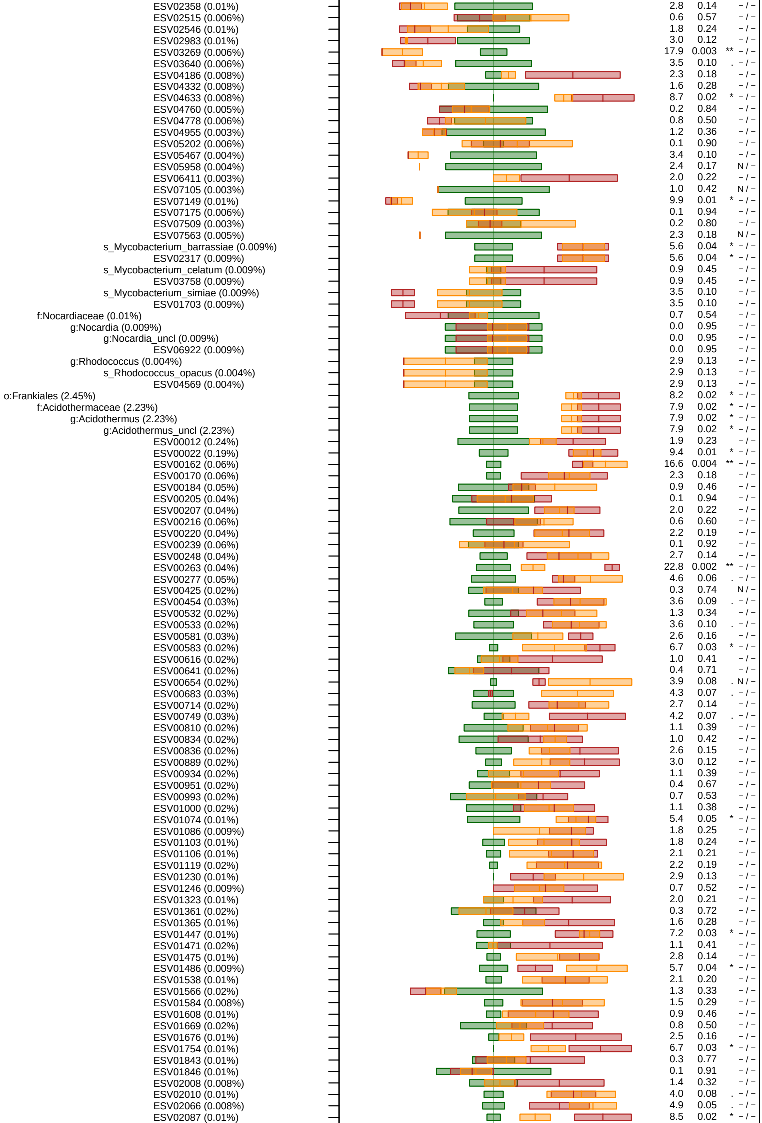
ESV02358 (0.01%) 2.8 0.14 – / –
ESV02515 (0.006%) 0.6 0.57 – / –
ESV02546 (0.01%) 1.8 0.24 – / –
ESV02983 (0.01%) 3.0 0.12 – / –
ESV03269 (0.006%) 17.9 0.003 ** – / –
ESV03640 (0.006%) 3.5 0.10 . – / –
ESV04186 (0.008%) 2.3 0.18 – / –
ESV04332 (0.008%) 1.6 0.28 – / –
ESV04633 (0.008%) 8.7 0.02 * – / –
ESV04760 (0.005%) 0.2 0.84 – / –
ESV04778 (0.006%) 0.8 0.50 – / –
ESV04955 (0.003%) 1.2 0.36 – / –
ESV05202 (0.006%) 0.1 0.90 – / –
ESV05467 (0.004%) 3.4 0.10 – / –
ESV05958 (0.004%) 2.4 0.17 N / –
ESV06411 (0.003%) 2.0 0.22 – / –
ESV07105 (0.003%) 1.0 0.42 N / –
ESV07149 (0.01%) 9.9 0.01 * – / –
ESV07175 (0.006%) 0.1 0.94 – / –
ESV07509 (0.003%) 0.2 0.80 – / –
ESV07563 (0.005%) 2.3 0.18 N / –
s_Mycobacterium_barrassiae (0.009%) 5.6 0.04 * – / –
ESV02317 (0.009%) 5.6 0.04 * – / –
s_Mycobacterium_celatum (0.009%) 0.9 0.45 – / –
ESV03758 (0.009%) 0.9 0.45 – / –
s_Mycobacterium_simiae (0.009%) 3.5 0.10 – / –
ESV01703 (0.009%) 3.5 0.10 – / –
f:Nocardiaceae (0.01%) 0.7 0.54 – / –
g:Nocardia (0.009%) 0.0 0.95 – / –
g:Nocardia_uncl (0.009%) 0.0 0.95 – / –
ESV06922 (0.009%) 0.0 0.95 – / –
g:Rhodococcus (0.004%) 2.9 0.13 – / –
s_Rhodococcus_opacus (0.004%) 2.9 0.13 – / –
ESV04569 (0.004%) 2.9 0.13 – / –
o:Frankiales (2.45%) 8.2 0.02 * – / –
f:Acidothermaceae (2.23%) 7.9 0.02 * – / –
g:Acidothermus (2.23%) 7.9 0.02 * – / –
g:Acidothermus_uncl (2.23%) 7.9 0.02 * – / –
ESV00012 (0.24%) 1.9 0.23 – / –
ESV00022 (0.19%) 9.4 0.01 * – / –
ESV00162 (0.06%) 16.6 0.004 ** – / –
ESV00170 (0.06%) 2.3 0.18 – / –
ESV00184 (0.05%) 0.9 0.46 – / –
ESV00205 (0.04%) 0.1 0.94 – / –
ESV00207 (0.04%) 2.0 0.22 – / –
ESV00216 (0.06%) 0.6 0.60 – / –
ESV00220 (0.04%) 2.2 0.19 – / –
ESV00239 (0.06%) 0.1 0.92 – / –
ESV00248 (0.04%) 2.7 0.14 – / –
ESV00263 (0.04%) 22.8 0.002 ** – / –
ESV00277 (0.05%) 4.6 0.06 . – / –
ESV00425 (0.02%) 0.3 0.74 N / –
ESV00454 (0.03%) 3.6 0.09 . – / –
ESV00532 (0.02%) 1.3 0.34 – / –
ESV00533 (0.02%) 3.6 0.10 . – / –
ESV00581 (0.03%) 2.6 0.16 – / –
ESV00583 (0.02%) 6.7 0.03 * – / –
ESV00616 (0.02%) 1.0 0.41 – / –
ESV00641 (0.02%) 0.4 0.71 – / –
ESV00654 (0.02%) 3.9 0.08 . N / –
ESV00683 (0.03%) 4.3 0.07 . – / –
ESV00714 (0.02%) 2.7 0.14 – / –
ESV00749 (0.03%) 4.2 0.07 . – / –
ESV00810 (0.02%) 1.1 0.39 – / –
ESV00834 (0.02%) 1.0 0.42 – / –
ESV00836 (0.02%) 2.6 0.15 – / –
ESV00889 (0.02%) 3.0 0.12 – / –
ESV00934 (0.02%) 1.1 0.39 – / –
ESV00951 (0.02%) 0.4 0.67 – / –
ESV00993 (0.02%) 0.7 0.53 – / –
ESV01000 (0.02%) 1.1 0.38 – / –
ESV01074 (0.01%) 5.4 0.05 * – / –
ESV01086 (0.009%) 1.8 0.25 – / –
ESV01103 (0.01%) 1.8 0.24 – / –
ESV01106 (0.01%) 2.1 0.21 – / –
ESV01119 (0.02%) 2.2 0.19 – / –
ESV01230 (0.01%) 2.9 0.13 – / –
ESV01246 (0.009%) 0.7 0.52 – / –
ESV01323 (0.01%) 2.0 0.21 – / –
ESV01361 (0.02%) 0.3 0.72 – / –
ESV01365 (0.01%) 1.6 0.28 – / –
ESV01447 (0.01%) 7.2 0.03 * – / –
ESV01471 (0.02%) 1.1 0.41 – / –
ESV01475 (0.01%) 2.8 0.14 – / –
ESV01486 (0.009%) 5.7 0.04 * – / –
ESV01538 (0.01%) 2.1 0.20 – / –
ESV01566 (0.02%) 1.3 0.33 – / –
ESV01584 (0.008%) 1.5 0.29 – / –
ESV01608 (0.01%) 0.9 0.46 – / –
ESV01669 (0.02%) 0.8 0.50 – / –
ESV01676 (0.01%) 2.5 0.16 – / –
ESV01754 (0.01%) 6.7 0.03 * – / –
ESV01843 (0.01%) 0.3 0.77 – / –
ESV01846 (0.01%) 0.1 0.91 – / –
ESV02008 (0.008%) 1.4 0.32 – / –
ESV02010 (0.01%) 4.0 0.08 . – / –
ESV02066 (0.008%) 4.9 0.05 . – / –
ESV02087 (0.01%) 8.5 0.02 * – / –

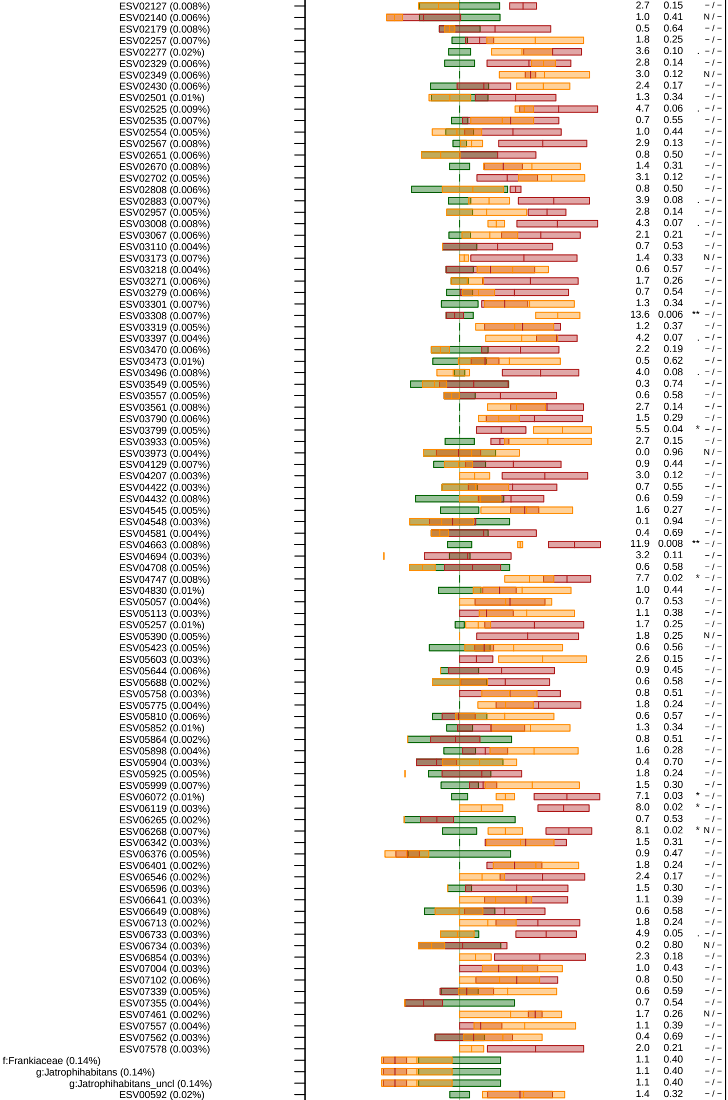
ESV02127 (0.008%) 2.7 0.15 – / –
ESV02140 (0.006%) 1.0 0.41 N / –
ESV02179 (0.008%) 0.5 0.64 – / –
ESV02257 (0.007%) 1.8 0.25 – / –
ESV02277 (0.02%) 3.6 0.10 . – / –
ESV02329 (0.006%) 2.8 0.14 – / –
ESV02349 (0.006%) 3.0 0.12 N / –
ESV02430 (0.006%) 2.4 0.17 – / –
ESV02501 (0.01%) 1.3 0.34 – / –
ESV02525 (0.009%) 4.7 0.06 . – / –
ESV02535 (0.007%) 0.7 0.55 – / –
ESV02554 (0.005%) 1.0 0.44 – / –
ESV02567 (0.008%) 2.9 0.13 – / –
ESV02651 (0.006%) 0.8 0.50 – / –
ESV02670 (0.008%) 1.4 0.31 – / –
ESV02702 (0.005%) 3.1 0.12 – / –
ESV02808 (0.006%) 0.8 0.50 – / –
ESV02883 (0.007%) 3.9 0.08 . – / –
ESV02957 (0.005%) 2.8 0.14 – / –
ESV03008 (0.008%) 4.3 0.07 . – / –
ESV03067 (0.006%) 2.1 0.21 – / –
ESV03110 (0.004%) 0.7 0.53 – / –
ESV03173 (0.007%) 1.4 0.33 N / –
ESV03218 (0.004%) 0.6 0.57 – / –
ESV03271 (0.006%) 1.7 0.26 – / –
ESV03279 (0.006%) 0.7 0.54 – / –
ESV03301 (0.007%) 1.3 0.34 – / –
ESV03308 (0.007%) 13.6 0.006 ** – / –
ESV03319 (0.005%) 1.2 0.37 – / –
ESV03397 (0.004%) 4.2 0.07 . – / –
ESV03470 (0.006%) 2.2 0.19 – / –
ESV03473 (0.01%) 0.5 0.62 – / –
ESV03496 (0.008%) 4.0 0.08 . – / –
ESV03549 (0.005%) 0.3 0.74 – / –
ESV03557 (0.005%) 0.6 0.58 – / –
ESV03561 (0.008%) 2.7 0.14 – / –
ESV03790 (0.006%) 1.5 0.29 – / –
ESV03799 (0.005%) 5.5 0.04 * – / –
ESV03933 (0.005%) 2.7 0.15 – / –
ESV03973 (0.004%) 0.0 0.96 N / –
ESV04129 (0.007%) 0.9 0.44 – / –
ESV04207 (0.003%) 3.0 0.12 – / –
ESV04422 (0.003%) 0.7 0.55 – / –
ESV04432 (0.008%) 0.6 0.59 – / –
ESV04545 (0.005%) 1.6 0.27 – / –
ESV04548 (0.003%) 0.1 0.94 – / –
ESV04581 (0.004%) 0.4 0.69 – / –
ESV04663 (0.008%) 11.9 0.008 ** – / –
ESV04694 (0.003%) 3.2 0.11 – / –
ESV04708 (0.005%) 0.6 0.58 – / –
ESV04747 (0.008%) 7.7 0.02 * – / –
ESV04830 (0.01%) 1.0 0.44 – / –
ESV05057 (0.004%) 0.7 0.53 – / –
ESV05113 (0.003%) 1.1 0.38 – / –
ESV05257 (0.01%) 1.7 0.25 – / –
ESV05390 (0.005%) 1.8 0.25 N / –
ESV05423 (0.005%) 0.6 0.56 – / –
ESV05603 (0.003%) 2.6 0.15 – / –
ESV05644 (0.006%) 0.9 0.45 – / –
ESV05688 (0.002%) 0.6 0.58 – / –
ESV05758 (0.003%) 0.8 0.51 – / –
ESV05775 (0.004%) 1.8 0.24 – / –
ESV05810 (0.006%) 0.6 0.57 – / –
ESV05852 (0.01%) 1.3 0.34 – / –
ESV05864 (0.002%) 0.8 0.51 – / –
ESV05898 (0.004%) 1.6 0.28 – / –
ESV05904 (0.003%) 0.4 0.70 – / –
ESV05925 (0.005%) 1.8 0.24 – / –
ESV05999 (0.007%) 1.5 0.30 – / –
ESV06072 (0.01%) 7.1 0.03 * – / –
ESV06119 (0.003%) 8.0 0.02 * – / –
ESV06265 (0.002%) 0.7 0.53 – / –
ESV06268 (0.007%) 8.1 0.02 * N / –
ESV06342 (0.003%) 1.5 0.31 – / –
ESV06376 (0.005%) 0.9 0.47 – / –
ESV06401 (0.002%) 1.8 0.24 – / –
ESV06546 (0.002%) 2.4 0.17 – / –
ESV06596 (0.003%) 1.5 0.30 – / –
ESV06641 (0.003%) 1.1 0.39 – / –
ESV06649 (0.008%) 0.6 0.58 – / –
ESV06713 (0.002%) 1.8 0.24 – / –
ESV06733 (0.003%) 4.9 0.05 . – / –
ESV06734 (0.003%) 0.2 0.80 N / –
ESV06854 (0.003%) 2.3 0.18 – / –
ESV07004 (0.003%) 1.0 0.43 – / –
ESV07102 (0.006%) 0.8 0.50 – / –
ESV07339 (0.005%) 0.6 0.59 – / –
ESV07355 (0.004%) 0.7 0.54 – / –
ESV07461 (0.002%) 1.7 0.26 N / –
ESV07557 (0.004%) 1.1 0.39 – / –
ESV07562 (0.003%) 0.4 0.69 – / –
ESV07578 (0.003%) 2.0 0.21 – / –
f:Frankiaceae (0.14%) 1.1 0.40 – / –
g:Jatrophihabitans (0.14%) 1.1 0.40 – / –
g:Jatrophihabitans_uncl (0.14%) 1.1 0.40 – / –
ESV00592 (0.02%) 1.4 0.32 – / –

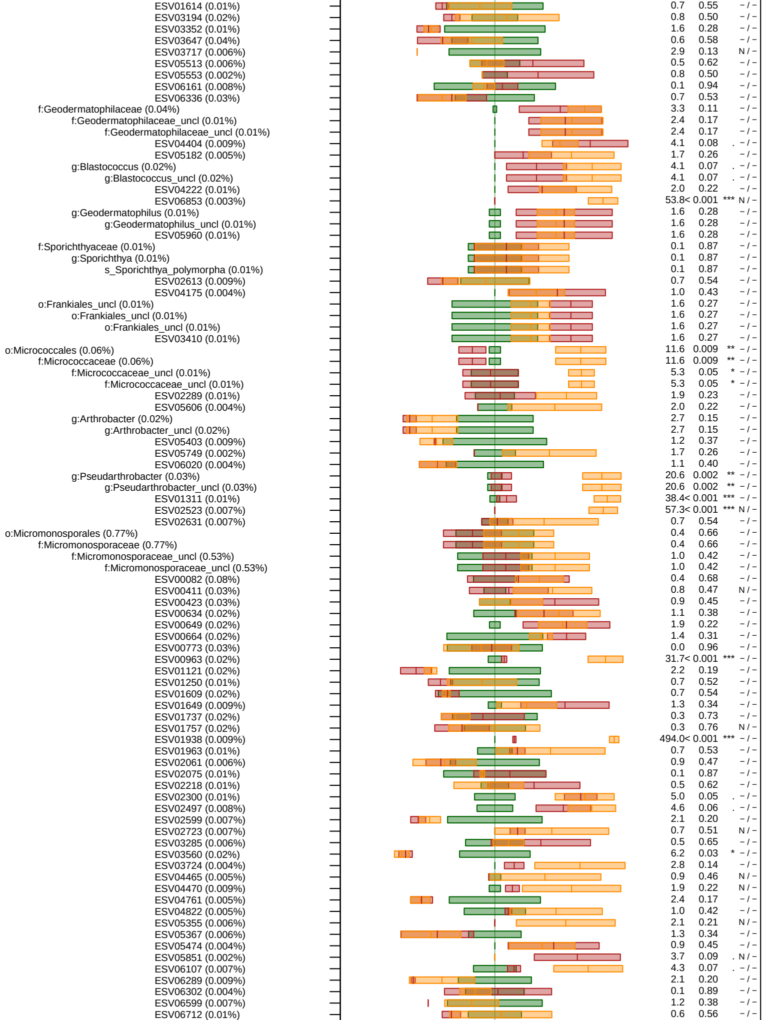
ESV01614 (0.01%) 0.7 0.55 – / –
ESV03194 (0.02%) 0.8 0.50 – / –
ESV03352 (0.01%) 1.6 0.28 – / –
ESV03647 (0.04%) 0.6 0.58 – / –
ESV03717 (0.006%) 2.9 0.13 N / –
ESV05513 (0.006%) 0.5 0.62 – / –
ESV05553 (0.002%) 0.8 0.50 – / –
ESV06161 (0.008%) 0.1 0.94 – / –
ESV06336 (0.03%) 0.7 0.53 – / –
f:Geodermatophilaceae (0.04%) 3.3 0.11 – / –
f:Geodermatophilaceae_uncl (0.01%) 2.4 0.17 – / –
f:Geodermatophilaceae_uncl (0.01%) 2.4 0.17 – / –
ESV04404 (0.009%) 4.1 0.08 . – / –
ESV05182 (0.005%) 1.7 0.26 – / –
g:Blastococcus (0.02%) 4.1 0.07 . – / –
g:Blastococcus_uncl (0.02%) 4.1 0.07 . – / –
ESV04222 (0.01%) 2.0 0.22 – / –
ESV06853 (0.003%) 53.8 < 0.001 *** N / –
g:Geodermatophilus (0.01%) 1.6 0.28 – / –
g:Geodermatophilus_uncl (0.01%) 1.6 0.28 – / –
ESV05960 (0.01%) 1.6 0.28 – / –
f:Sporichthyaceae (0.01%) 0.1 0.87 – / –
g:Sporichthya (0.01%) 0.1 0.87 – / –
s_Sporichthya_polymorpha (0.01%) 0.1 0.87 – / –
ESV02613 (0.009%) 0.7 0.54 – / –
ESV04175 (0.004%) 1.0 0.43 – / –
o:Frankiales_uncl (0.01%) 1.6 0.27 – / –
o:Frankiales_uncl (0.01%) 1.6 0.27 – / –
o:Frankiales_uncl (0.01%) 1.6 0.27 – / –
ESV03410 (0.01%) 1.6 0.27 – / –
o:Micrococcales (0.06%) 11.6 0.009 ** – / –
f:Micrococcaceae (0.06%) 11.6 0.009 ** – / –
f:Micrococcaceae_uncl (0.01%) 5.3 0.05 * – / –
f:Micrococcaceae_uncl (0.01%) 5.3 0.05 * – / –
ESV02289 (0.01%) 1.9 0.23 – / –
ESV05606 (0.004%) 2.0 0.22 – / –
g:Arthrobacter (0.02%) 2.7 0.15 – / –
g:Arthrobacter_uncl (0.02%) 2.7 0.15 – / –
ESV05403 (0.009%) 1.2 0.37 – / –
ESV05749 (0.002%) 1.7 0.26 – / –
ESV06020 (0.004%) 1.1 0.40 – / –
g:Pseudarthrobacter (0.03%) 20.6 0.002 ** – / –
g:Pseudarthrobacter_uncl (0.03%) 20.6 0.002 ** – / –
ESV01311 (0.01%) 38.4 < 0.001 *** – / –
ESV02523 (0.007%) 57.3 < 0.001 *** N / –
ESV02631 (0.007%) 0.7 0.54 – / –
o:Micromonosporales (0.77%) 0.4 0.66 – / –
f:Micromonosporaceae (0.77%) 0.4 0.66 – / –
f:Micromonosporaceae_uncl (0.53%) 1.0 0.42 – / –
f:Micromonosporaceae_uncl (0.53%) 1.0 0.42 – / –
ESV00082 (0.08%) 0.4 0.68 – / –
ESV00411 (0.03%) 0.8 0.47 N / –
ESV00423 (0.03%) 0.9 0.45 – / –
ESV00634 (0.02%) 1.1 0.38 – / –
ESV00649 (0.02%) 1.9 0.22 – / –
ESV00664 (0.02%) 1.4 0.31 – / –
ESV00773 (0.03%) 0.0 0.96 – / –
ESV00963 (0.02%) 31.7 < 0.001 *** – / –
ESV01121 (0.02%) 2.2 0.19 – / –
ESV01250 (0.01%) 0.7 0.52 – / –
ESV01609 (0.02%) 0.7 0.54 – / –
ESV01649 (0.009%) 1.3 0.34 – / –
ESV01737 (0.02%) 0.3 0.73 – / –
ESV01757 (0.02%) 0.3 0.76 N / –
ESV01938 (0.009%) 494.0 < 0.001 *** – / –
ESV01963 (0.01%) 0.7 0.53 – / –
ESV02061 (0.006%) 0.9 0.47 – / –
ESV02075 (0.01%) 0.1 0.87 – / –
ESV02218 (0.01%) 0.5 0.62 – / –
ESV02300 (0.01%) 5.0 0.05 . – / –
ESV02497 (0.008%) 4.6 0.06 . – / –
ESV02599 (0.007%) 2.1 0.20 – / –
ESV02723 (0.007%) 0.7 0.51 N / –
ESV03285 (0.006%) 0.5 0.65 – / –
ESV03560 (0.02%) 6.2 0.03 * – / –
ESV03724 (0.004%) 2.8 0.14 – / –
ESV04465 (0.005%) 0.9 0.46 N / –
ESV04470 (0.009%) 1.9 0.22 N / –
ESV04761 (0.005%) 2.4 0.17 – / –
ESV04822 (0.005%) 1.0 0.42 – / –
ESV05355 (0.006%) 2.1 0.21 N / –
ESV05367 (0.006%) 1.3 0.34 – / –
ESV05474 (0.004%) 0.9 0.45 – / –
ESV05851 (0.002%) 3.7 0.09 . N / –
ESV06107 (0.007%) 4.3 0.07 . – / –
ESV06289 (0.009%) 2.1 0.20 – / –
ESV06302 (0.004%) 0.1 0.89 – / –
ESV06599 (0.007%) 1.2 0.38 – / –
ESV06712 (0.01%) 0.6 0.56 – / –

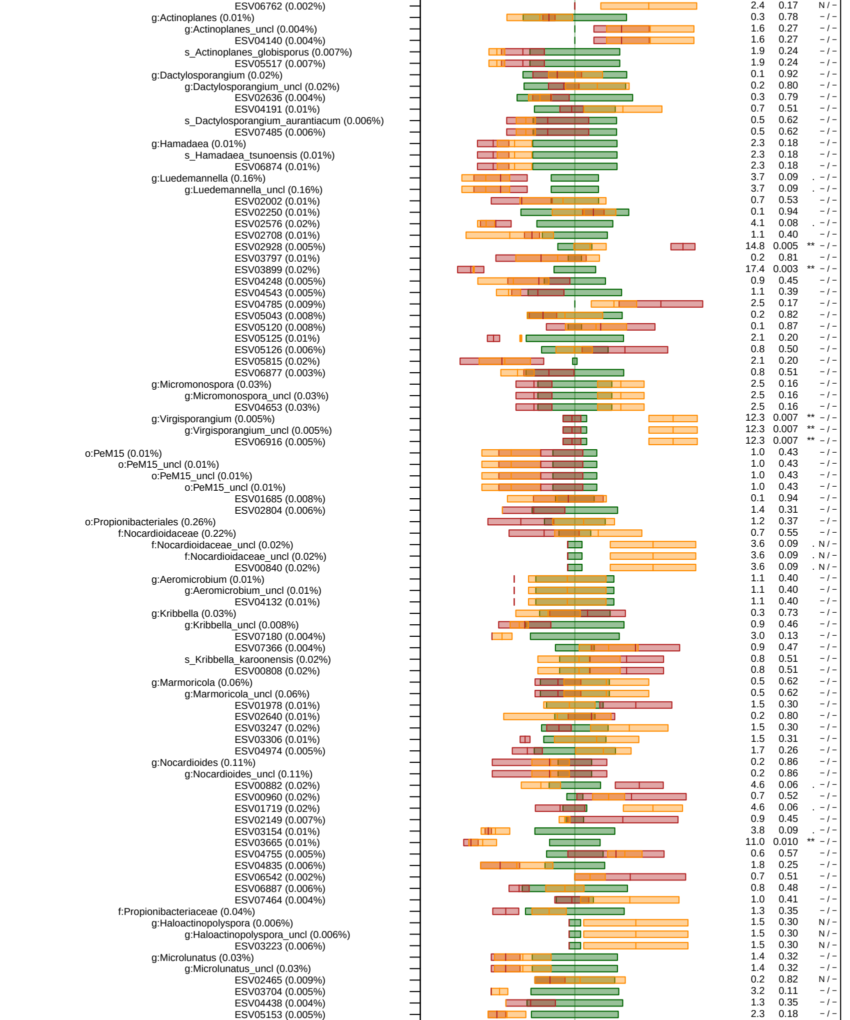
ESV06762 (0.002%) 2.4 0.17 N / –
g:Actinoplanes (0.01%) 0.3 0.78 – / –
g:Actinoplanes_uncl (0.004%) 1.6 0.27 – / –
ESV04140 (0.004%) 1.6 0.27 – / –
s_Actinoplanes_globisporus (0.007%) 1.9 0.24 – / –
ESV05517 (0.007%) 1.9 0.24 – / –
g:Dactylosporangium (0.02%) 0.1 0.92 – / –
g:Dactylosporangium_uncl (0.02%) 0.2 0.80 – / –
ESV02636 (0.004%) 0.3 0.79 – / –
ESV04191 (0.01%) 0.7 0.51 – / –
s_Dactylosporangium_aurantiacum (0.006%) 0.5 0.62 – / –
ESV07485 (0.006%) 0.5 0.62 – / –
g:Hamadaea (0.01%) 2.3 0.18 – / –
s_Hamadaea_tsunoensis (0.01%) 2.3 0.18 – / –
ESV06874 (0.01%) 2.3 0.18 – / –
g:Luedemannella (0.16%) 3.7 0.09 . – / –
g:Luedemannella_uncl (0.16%) 3.7 0.09 . – / –
ESV02002 (0.01%) 0.7 0.53 – / –
ESV02250 (0.01%) 0.1 0.94 – / –
ESV02576 (0.02%) 4.1 0.08 . – / –
ESV02708 (0.01%) 1.1 0.40 – / –
ESV02928 (0.005%) 14.8 0.005 ** – / –
ESV03797 (0.01%) 0.2 0.81 – / –
ESV03899 (0.02%) 17.4 0.003 ** – / –
ESV04248 (0.005%) 0.9 0.45 – / –
ESV04543 (0.005%) 1.1 0.39 – / –
ESV04785 (0.009%) 2.5 0.17 – / –
ESV05043 (0.008%) 0.2 0.82 – / –
ESV05120 (0.008%) 0.1 0.87 – / –
ESV05125 (0.01%) 2.1 0.20 – / –
ESV05126 (0.006%) 0.8 0.50 – / –
ESV05815 (0.02%) 2.1 0.20 – / –
ESV06877 (0.003%) 0.8 0.51 – / –
g:Micromonospora (0.03%) 2.5 0.16 – / –
g:Micromonospora_uncl (0.03%) 2.5 0.16 – / –
ESV04653 (0.03%) 2.5 0.16 – / –
g:Virgisporangium (0.005%) 12.3 0.007 ** – / –
g:Virgisporangium_uncl (0.005%) 12.3 0.007 ** – / –
ESV06916 (0.005%) 12.3 0.007 ** – / –
o:PeM15 (0.01%) 1.0 0.43 – / –
o:PeM15_uncl (0.01%) 1.0 0.43 – / –
o:PeM15_uncl (0.01%) 1.0 0.43 – / –
o:PeM15_uncl (0.01%) 1.0 0.43 – / –
ESV01685 (0.008%) 0.1 0.94 – / –
ESV02804 (0.006%) 1.4 0.31 – / –
o:Propionibacteriales (0.26%) 1.2 0.37 – / –
f:Nocardioidaceae (0.22%) 0.7 0.55 – / –
f:Nocardioidaceae_uncl (0.02%) 3.6 0.09 . N / –
f:Nocardioidaceae_uncl (0.02%) 3.6 0.09 . N / –
ESV00840 (0.02%) 3.6 0.09 . N / –
g:Aeromicrobium (0.01%) 1.1 0.40 – / –
g:Aeromicrobium_uncl (0.01%) 1.1 0.40 – / –
ESV04132 (0.01%) 1.1 0.40 – / –
g:Kribbella (0.03%) 0.3 0.73 – / –
g:Kribbella_uncl (0.008%) 0.9 0.46 – / –
ESV07180 (0.004%) 3.0 0.13 – / –
ESV07366 (0.004%) 0.9 0.47 – / –
s_Kribbella_karoonensis (0.02%) 0.8 0.51 – / –
ESV00808 (0.02%) 0.8 0.51 – / –
g:Marmoricola (0.06%) 0.5 0.62 – / –
g:Marmoricola_uncl (0.06%) 0.5 0.62 – / –
ESV01978 (0.01%) 1.5 0.30 – / –
ESV02640 (0.01%) 0.2 0.80 – / –
ESV03247 (0.02%) 1.5 0.30 – / –
ESV03306 (0.01%) 1.5 0.31 – / –
ESV04974 (0.005%) 1.7 0.26 – / –
g:Nocardioides (0.11%) 0.2 0.86 – / –
g:Nocardioides_uncl (0.11%) 0.2 0.86 – / –
ESV00882 (0.02%) 4.6 0.06 . – / –
ESV00960 (0.02%) 0.7 0.52 – / –
ESV01719 (0.02%) 4.6 0.06 . – / –
ESV02149 (0.007%) 0.9 0.45 – / –
ESV03154 (0.01%) 3.8 0.09 . – / –
ESV03665 (0.01%) 11.0 0.010 ** – / –
ESV04755 (0.005%) 0.6 0.57 – / –
ESV04835 (0.006%) 1.8 0.25 – / –
ESV06542 (0.002%) 0.7 0.51 – / –
ESV06887 (0.006%) 0.8 0.48 – / –
ESV07464 (0.004%) 1.0 0.41 – / –
f:Propionibacteriaceae (0.04%) 1.3 0.35 – / –
g:Haloactinopolyspora (0.006%) 1.5 0.30 N / –
g:Haloactinopolyspora_uncl (0.006%) 1.5 0.30 N / –
ESV03223 (0.006%) 1.5 0.30 N / –
g:Microlunatus (0.03%) 1.4 0.32 – / –
g:Microlunatus_uncl (0.03%) 1.4 0.32 – / –
ESV02465 (0.009%) 0.2 0.82 N / –
ESV03704 (0.005%) 3.2 0.11 – / –
ESV04438 (0.004%) 1.3 0.35 – / –
ESV05153 (0.005%) 2.3 0.18 – / –

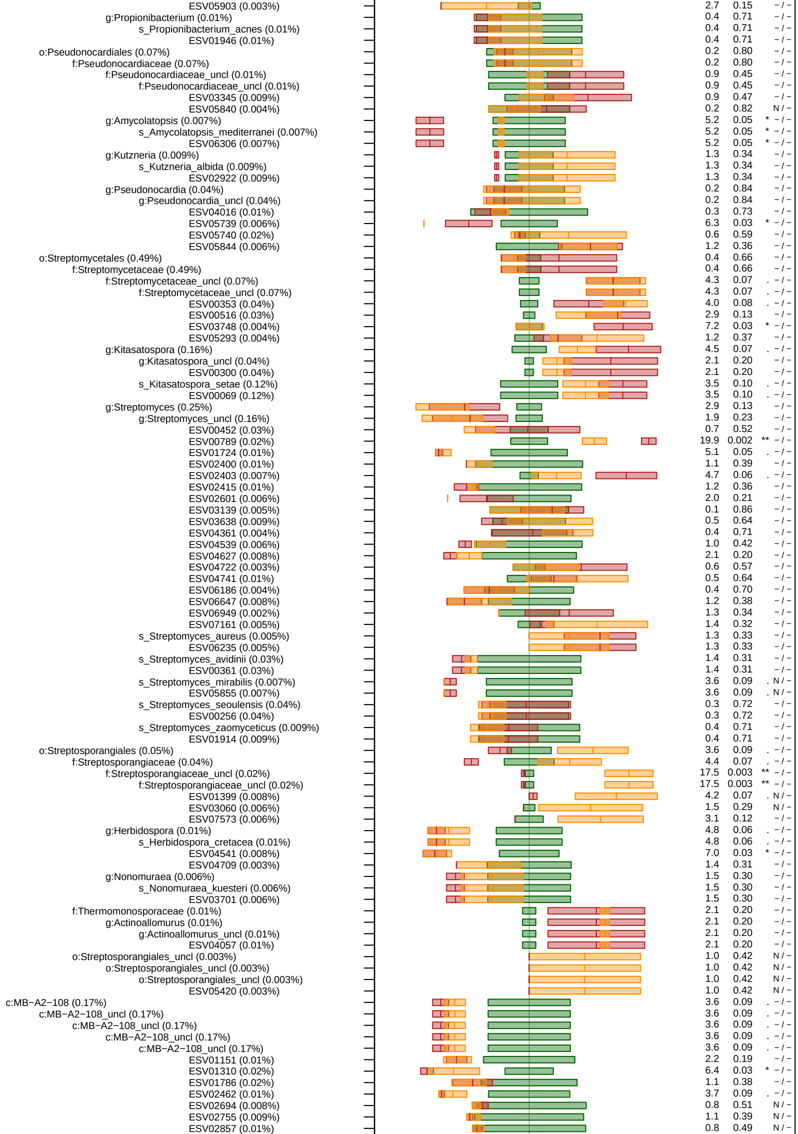
ESV05903 (0.003%) 2.7 0.15 – / –
g:Propionibacterium (0.01%) 0.4 0.71 – / –
s_Propionibacterium_acnes (0.01%) 0.4 0.71 – / –
ESV01946 (0.01%) 0.4 0.71 – / –
o:Pseudonocardiales (0.07%) 0.2 0.80 – / –
f:Pseudonocardiaceae (0.07%) 0.2 0.80 – / –
f:Pseudonocardiaceae_uncl (0.01%) 0.9 0.45 – / –
f:Pseudonocardiaceae_uncl (0.01%) 0.9 0.45 – / –
ESV03345 (0.009%) 0.9 0.47 – / –
ESV05840 (0.004%) 0.2 0.82 N / –
g:Amycolatopsis (0.007%) 5.2 0.05 * – / –
s_Amycolatopsis_mediterranei (0.007%) 5.2 0.05 * – / –
ESV06306 (0.007%) 5.2 0.05 * – / –
g:Kutzneria (0.009%) 1.3 0.34 – / –
s_Kutzneria_albida (0.009%) 1.3 0.34 – / –
ESV02922 (0.009%) 1.3 0.34 – / –
g:Pseudonocardia (0.04%) 0.2 0.84 – / –
g:Pseudonocardia_uncl (0.04%) 0.2 0.84 – / –
ESV04016 (0.01%) 0.3 0.73 – / –
ESV05739 (0.006%) 6.3 0.03 * – / –
ESV05740 (0.02%) 0.6 0.59 – / –
ESV05844 (0.006%) 1.2 0.36 – / –
o:Streptomycetales (0.49%) 0.4 0.66 – / –
f:Streptomycetaceae (0.49%) 0.4 0.66 – / –
f:Streptomycetaceae_uncl (0.07%) 4.3 0.07 . – / –
f:Streptomycetaceae_uncl (0.07%) 4.3 0.07 . – / –
ESV00353 (0.04%) 4.0 0.08 . – / –
ESV00516 (0.03%) 2.9 0.13 – / –
ESV03748 (0.004%) 7.2 0.03 * – / –
ESV05293 (0.004%) 1.2 0.37 – / –
g:Kitasatospora (0.16%) 4.5 0.07 . – / –
g:Kitasatospora_uncl (0.04%) 2.1 0.20 – / –
ESV00300 (0.04%) 2.1 0.20 – / –
s_Kitasatospora_setae (0.12%) 3.5 0.10 . – / –
ESV00069 (0.12%) 3.5 0.10 . – / –
g:Streptomyces (0.25%) 2.9 0.13 – / –
g:Streptomyces_uncl (0.16%) 1.9 0.23 – / –
ESV00452 (0.03%) 0.7 0.52 – / –
ESV00789 (0.02%) 19.9 0.002 ** – / –
ESV01724 (0.01%) 5.1 0.05 . – / –
ESV02400 (0.01%) 1.1 0.39 – / –
ESV02403 (0.007%) 4.7 0.06 . – / –
ESV02415 (0.01%) 1.2 0.36 – / –
ESV02601 (0.006%) 2.0 0.21 – / –
ESV03139 (0.005%) 0.1 0.86 – / –
ESV03638 (0.009%) 0.5 0.64 – / –
ESV04361 (0.004%) 0.4 0.71 – / –
ESV04539 (0.006%) 1.0 0.42 – / –
ESV04627 (0.008%) 2.1 0.20 – / –
ESV04722 (0.003%) 0.6 0.57 – / –
ESV04741 (0.01%) 0.5 0.64 – / –
ESV06186 (0.004%) 0.4 0.70 – / –
ESV06647 (0.008%) 1.2 0.38 – / –
ESV06949 (0.002%) 1.3 0.34 – / –
ESV07161 (0.005%) 1.4 0.32 – / –
s_Streptomyces_aureus (0.005%) 1.3 0.33 – / –
ESV06235 (0.005%) 1.3 0.33 – / –
s_Streptomyces_avidinii (0.03%) 1.4 0.31 – / –
ESV00361 (0.03%) 1.4 0.31 – / –
s_Streptomyces_mirabilis (0.007%) 3.6 0.09 . N / –
ESV05855 (0.007%) 3.6 0.09 . N / –
s_Streptomyces_seoulensis (0.04%) 0.3 0.72 – / –
ESV00256 (0.04%) 0.3 0.72 – / –
s_Streptomyces_zaomyceticus (0.009%) 0.4 0.71 – / –
ESV01914 (0.009%) 0.4 0.71 – / –
o:Streptosporangiales (0.05%) 3.6 0.09 . – / –
f:Streptosporangiaceae (0.04%) 4.4 0.07 . – / –
f:Streptosporangiaceae_uncl (0.02%) 17.5 0.003 ** – / –
f:Streptosporangiaceae_uncl (0.02%) 17.5 0.003 ** – / –
ESV01399 (0.008%) 4.2 0.07 . N / –
ESV03060 (0.006%) 1.5 0.29 N / –
ESV07573 (0.006%) 3.1 0.12 – / –
g:Herbidospora (0.01%) 4.8 0.06 . – / –
s_Herbidospora_cretacea (0.01%) 4.8 0.06 . – / –
ESV04541 (0.008%) 7.0 0.03 * – / –
ESV04709 (0.003%) 1.4 0.31 – / –
g:Nonomuraea (0.006%) 1.5 0.30 – / –
s_Nonomuraea_kuesteri (0.006%) 1.5 0.30 – / –
ESV03701 (0.006%) 1.5 0.30 – / –
f:Thermomonosporaceae (0.01%) 2.1 0.20 – / –
g:Actinoallomurus (0.01%) 2.1 0.20 – / –
g:Actinoallomurus_uncl (0.01%) 2.1 0.20 – / –
ESV04057 (0.01%) 2.1 0.20 – / –
o:Streptosporangiales_uncl (0.003%) 1.0 0.42 N / –
o:Streptosporangiales_uncl (0.003%) 1.0 0.42 N / –
o:Streptosporangiales_uncl (0.003%) 1.0 0.42 N / –
ESV05420 (0.003%) 1.0 0.42 N / –
c:MB−A2−108 (0.17%) 3.6 0.09 . – / –
c:MB−A2−108_uncl (0.17%) 3.6 0.09 . – / –
c:MB−A2−108_uncl (0.17%) 3.6 0.09 . – / –
c:MB−A2−108_uncl (0.17%) 3.6 0.09 . – / –
c:MB−A2−108_uncl (0.17%) 3.6 0.09 . – / –
ESV01151 (0.01%) 2.2 0.19 – / –
ESV01310 (0.02%) 6.4 0.03 * – / –
ESV01786 (0.02%) 1.1 0.38 – / –
ESV02462 (0.01%) 3.7 0.09 . – / –
ESV02694 (0.008%) 0.8 0.51 N / –
ESV02755 (0.009%) 1.1 0.39 N / –
ESV02857 (0.01%) 0.8 0.49 N / –

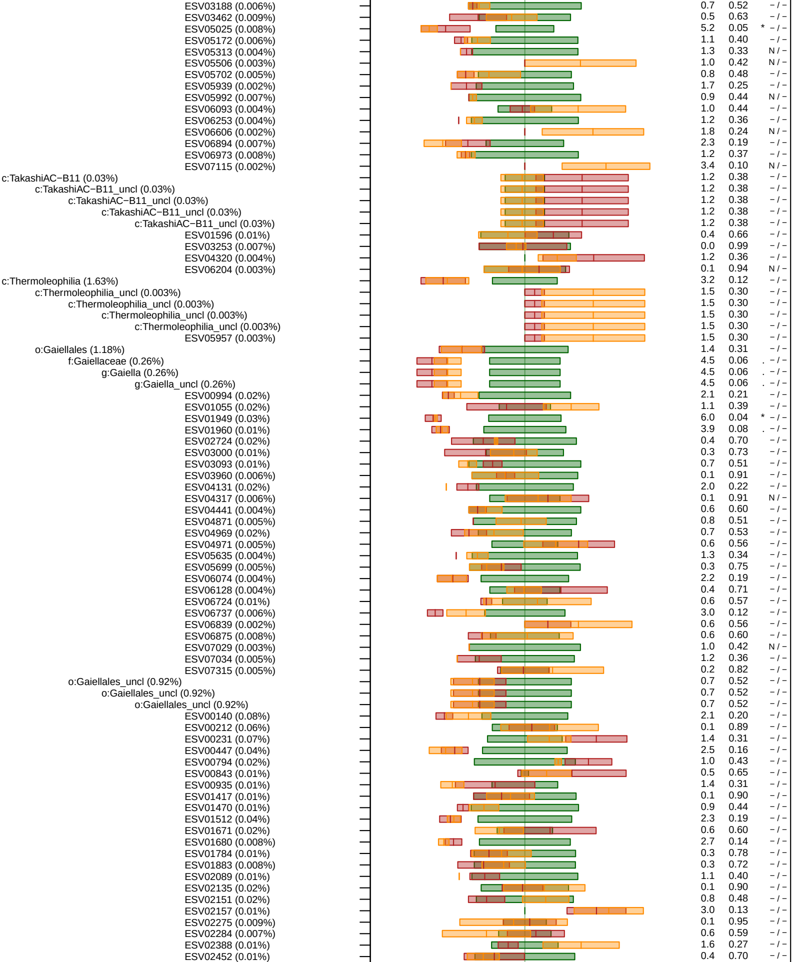
ESV03188 (0.006%) 0.7 0.52 – / –
ESV03462 (0.009%) 0.5 0.63 – / –
ESV05025 (0.008%) 5.2 0.05 * – / –
ESV05172 (0.006%) 1.1 0.40 – / –
ESV05313 (0.004%) 1.3 0.33 N / –
ESV05506 (0.003%) 1.0 0.42 N / –
ESV05702 (0.005%) 0.8 0.48 – / –
ESV05939 (0.002%) 1.7 0.25 – / –
ESV05992 (0.007%) 0.9 0.44 N / –
ESV06093 (0.004%) 1.0 0.44 – / –
ESV06253 (0.004%) 1.2 0.36 – / –
ESV06606 (0.002%) 1.8 0.24 N / –
ESV06894 (0.007%) 2.3 0.19 – / –
ESV06973 (0.008%) 1.2 0.37 – / –
ESV07115 (0.002%) 3.4 0.10 N / –
c:TakashiAC−B11 (0.03%) 1.2 0.38 – / –
c:TakashiAC−B11_uncl (0.03%) 1.2 0.38 – / –
c:TakashiAC−B11_uncl (0.03%) 1.2 0.38 – / –
c:TakashiAC−B11_uncl (0.03%) 1.2 0.38 – / –
c:TakashiAC−B11_uncl (0.03%) 1.2 0.38 – / –
ESV01596 (0.01%) 0.4 0.66 – / –
ESV03253 (0.007%) 0.0 0.99 – / –
ESV04320 (0.004%) 1.2 0.36 – / –
ESV06204 (0.003%) 0.1 0.94 N / –
c:Thermoleophilia (1.63%) 3.2 0.12 – / –
c:Thermoleophilia_uncl (0.003%) 1.5 0.30 – / –
c:Thermoleophilia_uncl (0.003%) 1.5 0.30 – / –
c:Thermoleophilia_uncl (0.003%) 1.5 0.30 – / –
c:Thermoleophilia_uncl (0.003%) 1.5 0.30 – / –
ESV05957 (0.003%) 1.5 0.30 – / –
o:Gaiellales (1.18%) 1.4 0.31 – / –
f:Gaiellaceae (0.26%) 4.5 0.06 . – / –
g:Gaiella (0.26%) 4.5 0.06 . – / –
g:Gaiella_uncl (0.26%) 4.5 0.06 . – / –
ESV00994 (0.02%) 2.1 0.21 – / –
ESV01055 (0.02%) 1.1 0.39 – / –
ESV01949 (0.03%) 6.0 0.04 * – / –
ESV01960 (0.01%) 3.9 0.08 . – / –
ESV02724 (0.02%) 0.4 0.70 – / –
ESV03000 (0.01%) 0.3 0.73 – / –
ESV03093 (0.01%) 0.7 0.51 – / –
ESV03960 (0.006%) 0.1 0.91 – / –
ESV04131 (0.02%) 2.0 0.22 – / –
ESV04317 (0.006%) 0.1 0.91 N / –
ESV04441 (0.004%) 0.6 0.60 – / –
ESV04871 (0.005%) 0.8 0.51 – / –
ESV04969 (0.02%) 0.7 0.53 – / –
ESV04971 (0.005%) 0.6 0.56 – / –
ESV05635 (0.004%) 1.3 0.34 – / –
ESV05699 (0.005%) 0.3 0.75 – / –
ESV06074 (0.004%) 2.2 0.19 – / –
ESV06128 (0.004%) 0.4 0.71 – / –
ESV06724 (0.01%) 0.6 0.57 – / –
ESV06737 (0.006%) 3.0 0.12 – / –
ESV06839 (0.002%) 0.6 0.56 – / –
ESV06875 (0.008%) 0.6 0.60 – / –
ESV07029 (0.003%) 1.0 0.42 N / –
ESV07034 (0.005%) 1.2 0.36 – / –
ESV07315 (0.005%) 0.2 0.82 – / –
o:Gaiellales_uncl (0.92%) 0.7 0.52 – / –
o:Gaiellales_uncl (0.92%) 0.7 0.52 – / –
o:Gaiellales_uncl (0.92%) 0.7 0.52 – / –
ESV00140 (0.08%) 2.1 0.20 – / –
ESV00212 (0.06%) 0.1 0.89 – / –
ESV00231 (0.07%) 1.4 0.31 – / –
ESV00447 (0.04%) 2.5 0.16 – / –
ESV00794 (0.02%) 1.0 0.43 – / –
ESV00843 (0.01%) 0.5 0.65 – / –
ESV00935 (0.01%) 1.4 0.31 – / –
ESV01417 (0.01%) 0.1 0.90 – / –
ESV01470 (0.01%) 0.9 0.44 – / –
ESV01512 (0.04%) 2.3 0.19 – / –
ESV01671 (0.02%) 0.6 0.60 – / –
ESV01680 (0.008%) 2.7 0.14 – / –
ESV01784 (0.01%) 0.3 0.78 – / –
ESV01883 (0.008%) 0.3 0.72 – / –
ESV02089 (0.01%) 1.1 0.40 – / –
ESV02135 (0.02%) 0.1 0.90 – / –
ESV02151 (0.02%) 0.8 0.48 – / –
ESV02157 (0.01%) 3.0 0.13 – / –
ESV02275 (0.009%) 0.1 0.95 – / –
ESV02284 (0.007%) 0.6 0.59 – / –
ESV02388 (0.01%) 1.6 0.27 – / –
ESV02452 (0.01%) 0.4 0.70 – / –

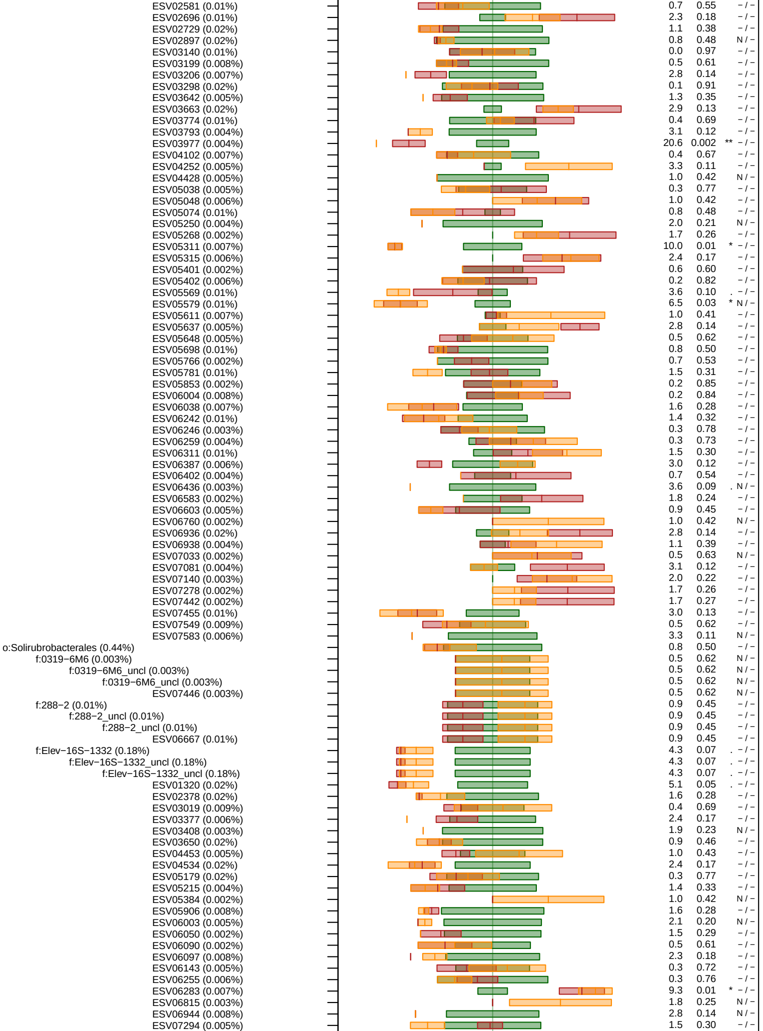
ESV02581 (0.01%) 0.7 0.55 – / –
ESV02696 (0.01%) 2.3 0.18 – / –
ESV02729 (0.02%) 1.1 0.38 – / –
ESV02897 (0.02%) 0.8 0.48 N / –
ESV03140 (0.01%) 0.0 0.97 – / –
ESV03199 (0.008%) 0.5 0.61 – / –
ESV03206 (0.007%) 2.8 0.14 – / –
ESV03298 (0.02%) 0.1 0.91 – / –
ESV03642 (0.005%) 1.3 0.35 – / –
ESV03663 (0.02%) 2.9 0.13 – / –
ESV03774 (0.01%) 0.4 0.69 – / –
ESV03793 (0.004%) 3.1 0.12 – / –
ESV03977 (0.004%) 20.6 0.002 ** – / –
ESV04102 (0.007%) 0.4 0.67 – / –
ESV04252 (0.005%) 3.3 0.11 – / –
ESV04428 (0.005%) 1.0 0.42 N / –
ESV05038 (0.005%) 0.3 0.77 – / –
ESV05048 (0.006%) 1.0 0.42 – / –
ESV05074 (0.01%) 0.8 0.48 – / –
ESV05250 (0.004%) 2.0 0.21 N / –
ESV05268 (0.002%) 1.7 0.26 – / –
ESV05311 (0.007%) 10.0 0.01 * – / –
ESV05315 (0.006%) 2.4 0.17 – / –
ESV05401 (0.002%) 0.6 0.60 – / –
ESV05402 (0.006%) 0.2 0.82 – / –
ESV05569 (0.01%) 3.6 0.10 . – / –
ESV05579 (0.01%) 6.5 0.03 * N / –
ESV05611 (0.007%) 1.0 0.41 – / –
ESV05637 (0.005%) 2.8 0.14 – / –
ESV05648 (0.005%) 0.5 0.62 – / –
ESV05698 (0.01%) 0.8 0.50 – / –
ESV05766 (0.002%) 0.7 0.53 – / –
ESV05781 (0.01%) 1.5 0.31 – / –
ESV05853 (0.002%) 0.2 0.85 – / –
ESV06004 (0.008%) 0.2 0.84 – / –
ESV06038 (0.007%) 1.6 0.28 – / –
ESV06242 (0.01%) 1.4 0.32 – / –
ESV06246 (0.003%) 0.3 0.78 – / –
ESV06259 (0.004%) 0.3 0.73 – / –
ESV06311 (0.01%) 1.5 0.30 – / –
ESV06387 (0.006%) 3.0 0.12 – / –
ESV06402 (0.004%) 0.7 0.54 – / –
ESV06436 (0.003%) 3.6 0.09 . N / –
ESV06583 (0.002%) 1.8 0.24 – / –
ESV06603 (0.005%) 0.9 0.45 – / –
ESV06760 (0.002%) 1.0 0.42 N / –
ESV06936 (0.02%) 2.8 0.14 – / –
ESV06938 (0.004%) 1.1 0.39 – / –
ESV07033 (0.002%) 0.5 0.63 N / –
ESV07081 (0.004%) 3.1 0.12 – / –
ESV07140 (0.003%) 2.0 0.22 – / –
ESV07278 (0.002%) 1.7 0.26 – / –
ESV07442 (0.002%) 1.7 0.27 – / –
ESV07455 (0.01%) 3.0 0.13 – / –
ESV07549 (0.009%) 0.5 0.62 – / –
ESV07583 (0.006%) 3.3 0.11 N / –
o:Solirubrobacterales (0.44%) 0.8 0.50 – / –
f:0319−6M6 (0.003%) 0.5 0.62 N / –
f:0319−6M6_uncl (0.003%) 0.5 0.62 N / –
f:0319−6M6_uncl (0.003%) 0.5 0.62 N / –
ESV07446 (0.003%) 0.5 0.62 N / –
f:288−2 (0.01%) 0.9 0.45 – / –
f:288−2_uncl (0.01%) 0.9 0.45 – / –
f:288−2_uncl (0.01%) 0.9 0.45 – / –
ESV06667 (0.01%) 0.9 0.45 – / –
f:Elev−16S−1332 (0.18%) 4.3 0.07 . – / –
f:Elev−16S−1332_uncl (0.18%) 4.3 0.07 . – / –
f:Elev−16S−1332_uncl (0.18%) 4.3 0.07 . – / –
ESV01320 (0.02%) 5.1 0.05 . – / –
ESV02378 (0.02%) 1.6 0.28 – / –
ESV03019 (0.009%) 0.4 0.69 – / –
ESV03377 (0.006%) 2.4 0.17 – / –
ESV03408 (0.003%) 1.9 0.23 N / –
ESV03650 (0.02%) 0.9 0.46 – / –
ESV04453 (0.005%) 1.0 0.43 – / –
ESV04534 (0.02%) 2.4 0.17 – / –
ESV05179 (0.02%) 0.3 0.77 – / –
ESV05215 (0.004%) 1.4 0.33 – / –
ESV05384 (0.002%) 1.0 0.42 N / –
ESV05906 (0.008%) 1.6 0.28 – / –
ESV06003 (0.005%) 2.1 0.20 N / –
ESV06050 (0.002%) 1.5 0.29 – / –
ESV06090 (0.002%) 0.5 0.61 – / –
ESV06097 (0.008%) 2.3 0.18 – / –
ESV06143 (0.005%) 0.3 0.72 – / –
ESV06255 (0.006%) 0.3 0.76 – / –
ESV06283 (0.007%) 9.3 0.01 * – / –
ESV06815 (0.003%) 1.8 0.25 N / –
ESV06944 (0.008%) 2.8 0.14 N / –
ESV07294 (0.005%) 1.5 0.30 – / –

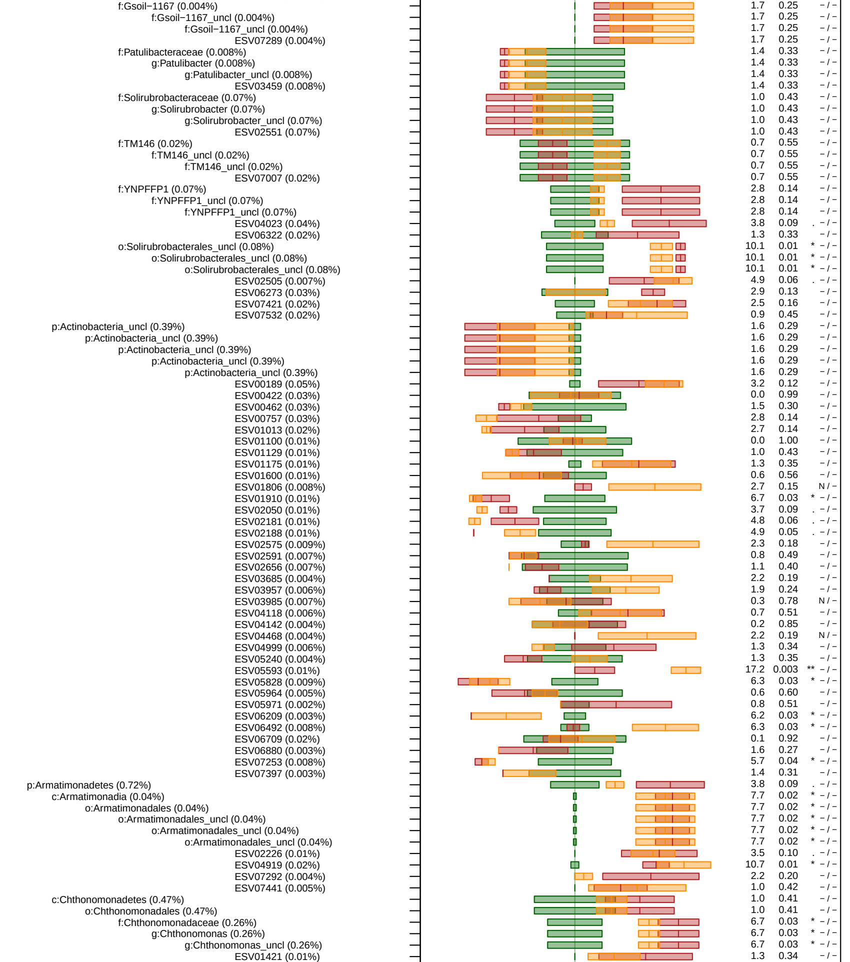
f:Gsoil−1167 (0.004%) 1.7 0.25 – / –
f:Gsoil−1167_uncl (0.004%) 1.7 0.25 – / –
f:Gsoil−1167_uncl (0.004%) 1.7 0.25 – / –
ESV07289 (0.004%) 1.7 0.25 – / –
f:Patulibacteraceae (0.008%) 1.4 0.33 – / –
g:Patulibacter (0.008%) 1.4 0.33 – / –
g:Patulibacter_uncl (0.008%) 1.4 0.33 – / –
ESV03459 (0.008%) 1.4 0.33 – / –
f:Solirubrobacteraceae (0.07%) 1.0 0.43 – / –
g:Solirubrobacter (0.07%) 1.0 0.43 – / –
g:Solirubrobacter_uncl (0.07%) 1.0 0.43 – / –
ESV02551 (0.07%) 1.0 0.43 – / –
f:TM146 (0.02%) 0.7 0.55 – / –
f:TM146_uncl (0.02%) 0.7 0.55 – / –
f:TM146_uncl (0.02%) 0.7 0.55 – / –
ESV07007 (0.02%) 0.7 0.55 – / –
f:YNPFFP1 (0.07%) 2.8 0.14 – / –
f:YNPFFP1_uncl (0.07%) 2.8 0.14 – / –
f:YNPFFP1_uncl (0.07%) 2.8 0.14 – / –
ESV04023 (0.04%) 3.8 0.09 . – / –
ESV06322 (0.02%) 1.3 0.33 – / –
o:Solirubrobacterales_uncl (0.08%) 10.1 0.01 * – / –
o:Solirubrobacterales_uncl (0.08%) 10.1 0.01 * – / –
o:Solirubrobacterales_uncl (0.08%) 10.1 0.01 * – / –
ESV02505 (0.007%) 4.9 0.06 . – / –
ESV06273 (0.03%) 2.9 0.13 – / –
ESV07421 (0.02%) 2.5 0.16 – / –
ESV07532 (0.02%) 0.9 0.45 – / –
p:Actinobacteria_uncl (0.39%) 1.6 0.29 – / –
p:Actinobacteria_uncl (0.39%) 1.6 0.29 – / –
p:Actinobacteria_uncl (0.39%) 1.6 0.29 – / –
p:Actinobacteria_uncl (0.39%) 1.6 0.29 – / –
p:Actinobacteria_uncl (0.39%) 1.6 0.29 – / –
ESV00189 (0.05%) 3.2 0.12 – / –
ESV00422 (0.03%) 0.0 0.99 – / –
ESV00462 (0.03%) 1.5 0.30 – / –
ESV00757 (0.03%) 2.8 0.14 – / –
ESV01013 (0.02%) 2.7 0.14 – / –
ESV01100 (0.01%) 0.0 1.00 – / –
ESV01129 (0.01%) 1.0 0.43 – / –
ESV01175 (0.01%) 1.3 0.35 – / –
ESV01600 (0.01%) 0.6 0.56 – / –
ESV01806 (0.008%) 2.7 0.15 N / –
ESV01910 (0.01%) 6.7 0.03 * – / –
ESV02050 (0.01%) 3.7 0.09 . – / –
ESV02181 (0.01%) 4.8 0.06 . – / –
ESV02188 (0.01%) 4.9 0.05 . – / –
ESV02575 (0.009%) 2.3 0.18 – / –
ESV02591 (0.007%) 0.8 0.49 – / –
ESV02656 (0.007%) 1.1 0.40 – / –
ESV03685 (0.004%) 2.2 0.19 – / –
ESV03957 (0.006%) 1.9 0.24 – / –
ESV03985 (0.007%) 0.3 0.78 N / –
ESV04118 (0.006%) 0.7 0.51 – / –
ESV04142 (0.004%) 0.2 0.85 – / –
ESV04468 (0.004%) 2.2 0.19 N / –
ESV04999 (0.006%) 1.3 0.34 – / –
ESV05240 (0.004%) 1.3 0.35 – / –
ESV05593 (0.01%) 17.2 0.003 ** – / –
ESV05828 (0.009%) 6.3 0.03 * – / –
ESV05964 (0.005%) 0.6 0.60 – / –
ESV05971 (0.002%) 0.8 0.51 – / –
ESV06209 (0.003%) 6.2 0.03 * – / –
ESV06492 (0.008%) 6.3 0.03 * – / –
ESV06709 (0.02%) 0.1 0.92 – / –
ESV06880 (0.003%) 1.6 0.27 – / –
ESV07253 (0.008%) 5.7 0.04 * – / –
ESV07397 (0.003%) 1.4 0.31 – / –
p:Armatimonadetes (0.72%) 3.8 0.09 . – / –
c:Armatimonadia (0.04%) 7.7 0.02 * – / –
o:Armatimonadales (0.04%) 7.7 0.02 * – / –
o:Armatimonadales_uncl (0.04%) 7.7 0.02 * – / –
o:Armatimonadales_uncl (0.04%) 7.7 0.02 * – / –
o:Armatimonadales_uncl (0.04%) 7.7 0.02 * – / –
ESV02226 (0.01%) 3.5 0.10 . – / –
ESV04919 (0.02%) 10.7 0.01 * – / –
ESV07292 (0.004%) 2.2 0.20 – / –
ESV07441 (0.005%) 1.0 0.42 – / –
c:Chthonomonadetes (0.47%) 1.0 0.41 – / –
o:Chthonomonadales (0.47%) 1.0 0.41 – / –
f:Chthonomonadaceae (0.26%) 6.7 0.03 * – / –
g:Chthonomonas (0.26%) 6.7 0.03 * – / –
g:Chthonomonas_uncl (0.26%) 6.7 0.03 * – / –
ESV01421 (0.01%) 1.3 0.34 – / –

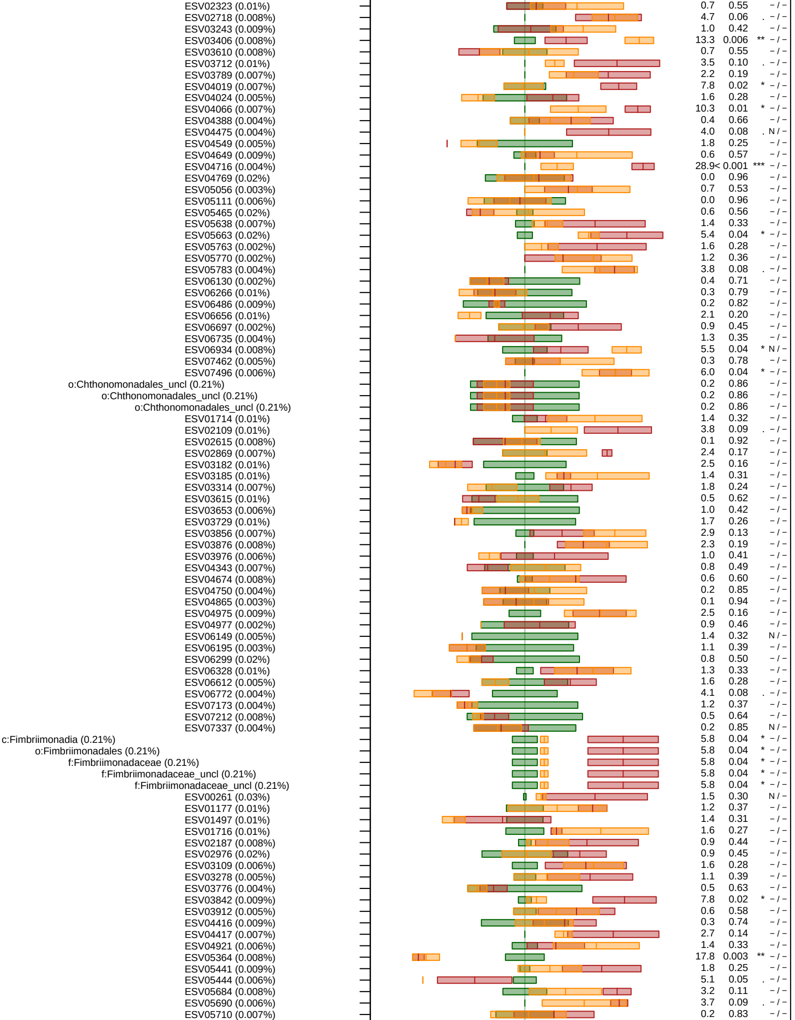
ESV02323 (0.01%) 0.7 0.55 – / –
ESV02718 (0.008%) 4.7 0.06 . – / –
ESV03243 (0.009%) 1.0 0.42 – / –
ESV03406 (0.008%) 13.3 0.006 ** – / –
ESV03610 (0.008%) 0.7 0.55 – / –
ESV03712 (0.01%) 3.5 0.10 . – / –
ESV03789 (0.007%) 2.2 0.19 – / –
ESV04019 (0.007%) 7.8 0.02 * – / –
ESV04024 (0.005%) 1.6 0.28 – / –
ESV04066 (0.007%) 10.3 0.01 * – / –
ESV04388 (0.004%) 0.4 0.66 – / –
ESV04475 (0.004%) 4.0 0.08 . N / –
ESV04549 (0.005%) 1.8 0.25 – / –
ESV04649 (0.009%) 0.6 0.57 – / –
ESV04716 (0.004%) 28.9 < 0.001 *** – / –
ESV04769 (0.02%) 0.0 0.96 – / –
ESV05056 (0.003%) 0.7 0.53 – / –
ESV05111 (0.006%) 0.0 0.96 – / –
ESV05465 (0.02%) 0.6 0.56 – / –
ESV05638 (0.007%) 1.4 0.33 – / –
ESV05663 (0.02%) 5.4 0.04 * – / –
ESV05763 (0.002%) 1.6 0.28 – / –
ESV05770 (0.002%) 1.2 0.36 – / –
ESV05783 (0.004%) 3.8 0.08 . – / –
ESV06130 (0.002%) 0.4 0.71 – / –
ESV06266 (0.01%) 0.3 0.79 – / –
ESV06486 (0.009%) 0.2 0.82 – / –
ESV06656 (0.01%) 2.1 0.20 – / –
ESV06697 (0.002%) 0.9 0.45 – / –
ESV06735 (0.004%) 1.3 0.35 – / –
ESV06934 (0.008%) 5.5 0.04 * N / –
ESV07462 (0.005%) 0.3 0.78 – / –
ESV07496 (0.006%) 6.0 0.04 * – / –
o:Chthonomonadales_uncl (0.21%) 0.2 0.86 – / –
o:Chthonomonadales_uncl (0.21%) 0.2 0.86 – / –
o:Chthonomonadales_uncl (0.21%) 0.2 0.86 – / –
ESV01714 (0.01%) 1.4 0.32 – / –
ESV02109 (0.01%) 3.8 0.09 . – / –
ESV02615 (0.008%) 0.1 0.92 – / –
ESV02869 (0.007%) 2.4 0.17 – / –
ESV03182 (0.01%) 2.5 0.16 – / –
ESV03185 (0.01%) 1.4 0.31 – / –
ESV03314 (0.007%) 1.8 0.24 – / –
ESV03615 (0.01%) 0.5 0.62 – / –
ESV03653 (0.006%) 1.0 0.42 – / –
ESV03729 (0.01%) 1.7 0.26 – / –
ESV03856 (0.007%) 2.9 0.13 – / –
ESV03876 (0.008%) 2.3 0.19 – / –
ESV03976 (0.006%) 1.0 0.41 – / –
ESV04343 (0.007%) 0.8 0.49 – / –
ESV04674 (0.008%) 0.6 0.60 – / –
ESV04750 (0.004%) 0.2 0.85 – / –
ESV04865 (0.003%) 0.1 0.94 – / –
ESV04975 (0.009%) 2.5 0.16 – / –
ESV04977 (0.002%) 0.9 0.46 – / –
ESV06149 (0.005%) 1.4 0.32 N / –
ESV06195 (0.003%) 1.1 0.39 – / –
ESV06299 (0.02%) 0.8 0.50 – / –
ESV06328 (0.01%) 1.3 0.33 – / –
ESV06612 (0.005%) 1.6 0.28 – / –
ESV06772 (0.004%) 4.1 0.08 . – / –
ESV07173 (0.004%) 1.2 0.37 – / –
ESV07212 (0.008%) 0.5 0.64 – / –
ESV07337 (0.004%) 0.2 0.85 N / –
c:Fimbriimonadia (0.21%) 5.8 0.04 * – / –
o:Fimbriimonadales (0.21%) 5.8 0.04 * – / –
f:Fimbriimonadaceae (0.21%) 5.8 0.04 * – / –
f:Fimbriimonadaceae_uncl (0.21%) 5.8 0.04 * – / –
f:Fimbriimonadaceae_uncl (0.21%) 5.8 0.04 * – / –
ESV00261 (0.03%) 1.5 0.30 N / –
ESV01177 (0.01%) 1.2 0.37 – / –
ESV01497 (0.01%) 1.4 0.31 – / –
ESV01716 (0.01%) 1.6 0.27 – / –
ESV02187 (0.008%) 0.9 0.44 – / –
ESV02976 (0.02%) 0.9 0.45 – / –
ESV03109 (0.006%) 1.6 0.28 – / –
ESV03278 (0.005%) 1.1 0.39 – / –
ESV03776 (0.004%) 0.5 0.63 – / –
ESV03842 (0.009%) 7.8 0.02 * – / –
ESV03912 (0.005%) 0.6 0.58 – / –
ESV04416 (0.009%) 0.3 0.74 – / –
ESV04417 (0.007%) 2.7 0.14 – / –
ESV04921 (0.006%) 1.4 0.33 – / –
ESV05364 (0.008%) 17.8 0.003 ** – / –
ESV05441 (0.009%) 1.8 0.25 – / –
ESV05444 (0.006%) 5.1 0.05 . – / –
ESV05684 (0.008%) 3.2 0.11 – / –
ESV05690 (0.006%) 3.7 0.09 . – / –
ESV05710 (0.007%) 0.2 0.83 – / –

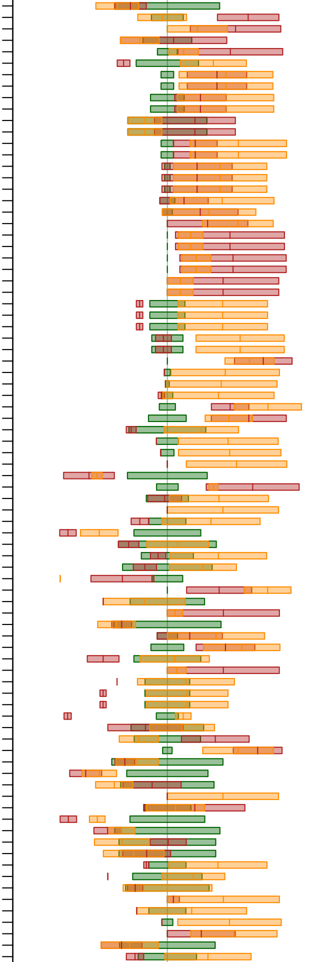

ESV05997 (0.006%)	0.6	0.59		− / −
ESV06551 (0.006%)	3.9	0.08	.	− / −
ESV06857 (0.003%)	1.2	0.37		− / −
ESV07365 (0.003%)	0.3	0.78		− / −
ESV07501 (0.006%)	1.1	0.41		− / −
p:Bacteroidetes (3.89%)	2.9	0.13		− / −
c:Bacteroidia (0.04%)	1.0	0.44		− / −
o:Bacteroidales (0.04%)	1.0	0.44		− / −
f:Bacteroidaceae (0.01%)	0.8	0.50		− / −
g:Bacteroides (0.01%)	0.8	0.50		− / −
s_Bacteroides_dorei (0.003%)	0.5	0.61		− / −
ESV05515 (0.003%)	0.5	0.61		− / −
s_Bacteroides_vulgatus (0.009%)	1.4	0.33		− / −
ESV03004 (0.009%)	1.4	0.33		− / −
f:Prevotellaceae (0.02%)	0.6	0.57		− / −
g:Prevotella_9 (0.02%)	0.6	0.57		− / −
g:Prevotella_9_uncl (0.02%)	0.6	0.57		− / −
ESV04250 (0.006%)	0.7	0.53		− / −
ESV05529 (0.008%)	0.4	0.69		N / −
ESV06835 (0.003%)	1.3	0.34		− / −
f:Rikenellaceae (0.008%)	1.0	0.43		− / −
g:Alistipes (0.008%)	1.0	0.43		− / −
s_Alistipes_putredinis (0.005%)	1.1	0.40		− / −
ESV02570 (0.005%)	1.1	0.40		− / −
s_Alistipes_shahii (0.002%)	0.8	0.50		− / −
ESV07338 (0.002%)	0.8	0.50		− / −
c:Cytophagia (0.66%)	2.3	0.18		− / −
o:Cytophagales (0.66%)	2.3	0.18		− / −
f:Cytophagaceae (0.66%)	2.3	0.18		− / −
f:Cytophagaceae_uncl (0.31%)	2.5	0.16		− / −
f:Cytophagaceae_uncl (0.31%)	2.5	0.16		− / −
ESV00434 (0.03%)	5.6	0.04	*	− / −
ESV00605 (0.02%)	1.2	0.36		N / −
ESV01021 (0.01%)	1.0	0.43		N / −
ESV01080 (0.01%)	0.9	0.44		N / −
ESV01462 (0.01%)	5.1	0.05	.	− / −
ESV01767 (0.01%)	2.3	0.18		− / −
ESV02017 (0.02%)	1.3	0.35		− / −
ESV02078 (0.007%)	1.7	0.26		− / −
ESV03103 (0.005%)	1.6	0.27		− / −
ESV03172 (0.005%)	1.9	0.23		N / −
ESV03493 (0.02%)	2.5	0.17		− / −
ESV03829 (0.01%)	2.4	0.17		− / −
ESV04264 (0.01%)	0.9	0.46		− / −
ESV04402 (0.005%)	1.0	0.42		N / −
ESV04637 (0.005%)	1.4	0.33		− / −
ESV04839 (0.03%)	5.0	0.05	.	− / −
ESV05432 (0.004%)	0.6	0.59		− / −
ESV05472 (0.02%)	1.0	0.41		− / −
ESV05659 (0.006%)	0.8	0.50		− / −
ESV05836 (0.005%)	7.1	0.03	*	− / −
ESV05847 (0.005%)	4.7	0.06	.	− / −
ESV05871 (0.002%)	1.0	0.41		− / −
ESV05986 (0.003%)	0.9	0.47		N / −
ESV06315 (0.01%)	0.7	0.53		− / −
ESV06361 (0.007%)	0.5	0.63		− / −
ESV06430 (0.008%)	1.8	0.25		− / −
ESV06814 (0.02%)	1.8	0.24		− / −
ESV07467 (0.003%)	0.8	0.48		N / −
ESV07493 (0.007%)	1.3	0.33		− / −
g:Chryseolinea (0.33%)	2.6	0.15		− / −
g:Chryseolinea_uncl (0.33%)	2.6	0.15		− / −
ESV00309 (0.04%)	60.0	< 0.001	***	− / −
ESV00563 (0.04%)	0.3	0.77		N / −
ESV00567 (0.03%)	1.7	0.26		− / −
ESV00890 (0.02%)	3.6	0.09	.	− / −
ESV01641 (0.01%)	0.4	0.69		− / −
ESV02011 (0.01%)	2.6	0.15		− / −
ESV02224 (0.01%)	0.7	0.55		− / −
ESV02417 (0.006%)	1.0	0.42		N / −
ESV02629 (0.01%)	0.2	0.86		− / −
ESV02764 (0.009%)	5.1	0.05	.	− / −
ESV02809 (0.01%)	0.9	0.44		− / −
ESV02899 (0.009%)	0.6	0.57		− / −
ESV03276 (0.01%)	0.2	0.80		− / −
ESV03323 (0.01%)	1.5	0.30		− / −
ESV03402 (0.005%)	2.6	0.15		− / −
ESV03466 (0.02%)	0.3	0.77		− / −
ESV03706 (0.005%)	0.9	0.46		N / −
ESV03814 (0.004%)	0.7	0.54		− / −
ESV03862 (0.006%)	1.6	0.28		N / −
ESV04298 (0.003%)	1.1	0.39		− / −
ESV04310 (0.007%)	0.5	0.63		− / −
ESV04571 (0.01%)	1.4	0.31		− / −

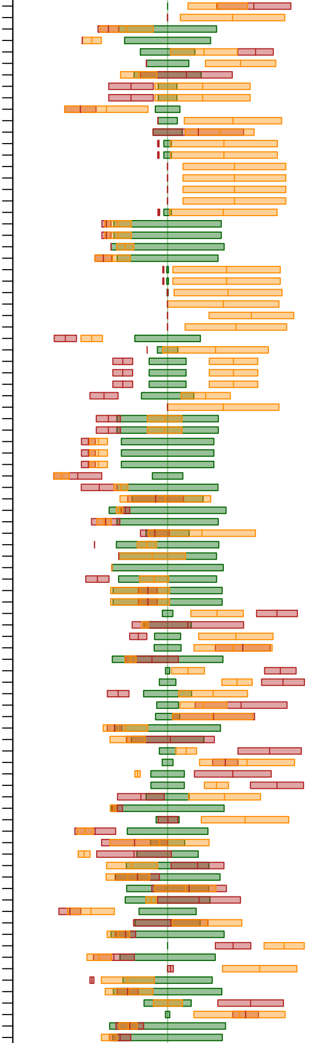

ESV04675 (0.004%)	2.5	0.16		– / –
ESV05042 (0.003%)	1.5	0.29		N / –
ESV05150 (0.006%)	0.9	0.47		– / –
ESV05212 (0.005%)	3.4	0.10		– / –
ESV05572 (0.007%)	2.7	0.15		– / –
ESV06797 (0.004%)	4.3	0.07	.	– / –
ESV07530 (0.005%)	0.5	0.64		– / –
g:Ohtaekwangia (0.02%)	1.3	0.33		– / –
g:Ohtaekwangia_uncl (0.02%)	1.3	0.33		– / –
ESV02894 (0.007%)	2.8	0.14		– / –
ESV05200 (0.006%)	2.0	0.21		– / –
ESV07045 (0.004%)	0.6	0.58		– / –
c:Flavobacteriia (0.22%)	1.3	0.34		N / –
o:Flavobacteriales (0.22%)	1.3	0.34		N / –
f:Cryomorphaceae (0.003%)	1.7	0.26		N / –
g:Fluviicola (0.003%)	1.7	0.26		N / –
g:Fluviicola_uncl (0.003%)	1.7	0.26		N / –
ESV06509 (0.003%)	1.7	0.26		N / –
f:Flavobacteriaceae (0.21%)	1.3	0.35		N / –
g:Chryseobacterium (0.02%)	1.0	0.41		– / –
g:Chryseobacterium_uncl (0.02%)	1.0	0.41		– / –
ESV03628 (0.007%)	0.8	0.49		N / –
ESV06950 (0.01%)	1.2	0.36		– / –
g:Flavobacterium (0.19%)	1.3	0.34		N / –
g:Flavobacterium_uncl (0.19%)	1.3	0.34		N / –
ESV00066 (0.1%)	1.3	0.35		N / –
ESV00213 (0.05%)	1.0	0.42		N / –
ESV00991 (0.01%)	3.9	0.08	.	N / –
ESV01063 (0.01%)	1.8	0.24		N / –
ESV02579 (0.02%)	6.4	0.03	*	– / –
ESV07037 (0.004%)	1.3	0.35		– / –
f:NS9_marine_group (0.009%)	9.1	0.02	*	– / –
f:NS9_marine_group:uncl (0.009%)	9.1	0.02	*	– / –
f:NS9_marine_group:uncl (0.009%)	9.1	0.02	*	– / –
ESV03351 (0.007%)	5.3	0.05	*	– / –
ESV06498 (0.002%)	1.0	0.42		N / –
c:Sphingobacteriia (2.97%)	1.1	0.39		– / –
o:Sphingobacteriales (2.97%)	1.1	0.39		– / –
f:AKYH767 (0.08%)	2.5	0.17		– / –
f:AKYH767_uncl (0.08%)	2.5	0.17		– / –
f:AKYH767_uncl (0.08%)	2.5	0.17		– / –
ESV01506 (0.02%)	11.3	0.009	**	– / –
ESV03135 (0.01%)	1.3	0.35		– / –
ESV03450 (0.006%)	0.0	0.97		– / –
ESV05471 (0.01%)	0.6	0.58		N / –
ESV05725 (0.01%)	1.4	0.32		– / –
ESV06391 (0.004%)	0.5	0.63		– / –
ESV06488 (0.007%)	1.6	0.28		– / –
ESV06640 (0.002%)	0.5	0.61		– / –
ESV07038 (0.002%)	1.0	0.42		N / –
f:Chitinophagaceae (2.5%)	1.5	0.29		– / –
f:Chitinophagaceae_uncl (1.75%)	0.2	0.86		– / –
f:Chitinophagaceae_uncl (1.75%)	0.2	0.86		– / –
ESV00196 (0.06%)	7.9	0.02	*	– / –
ESV00282 (0.05%)	0.4	0.70		– / –
ESV00286 (0.05%)	4.6	0.06	.	– / –
ESV00362 (0.04%)	2.0	0.21		– / –
ESV00545 (0.03%)	0.3	0.77		– / –
ESV00587 (0.02%)	20.6	0.002	**	– / –
ESV00602 (0.02%)	13.4	0.006	**	– / –
ESV00687 (0.03%)	3.6	0.09	.	– / –
ESV00775 (0.02%)	1.5	0.31		– / –
ESV00786 (0.02%)	0.6	0.58		– / –
ESV00830 (0.02%)	0.7	0.53		– / –
ESV00832 (0.02%)	0.5	0.64		– / –
ESV00838 (0.02%)	7.5	0.02	*	– / –
ESV00911 (0.02%)	2.1	0.21		– / –
ESV00967 (0.03%)	4.0	0.08	.	– / –
ESV00986 (0.02%)	8.0	0.02	*	– / –
ESV00997 (0.01%)	2.4	0.17		– / –
ESV00999 (0.02%)	0.8	0.48		N / –
ESV01014 (0.02%)	2.8	0.14		– / –
ESV01110 (0.02%)	2.9	0.13		– / –
ESV01135 (0.02%)	0.2	0.80		– / –
ESV01170 (0.01%)	2.2	0.19		– / –
ESV01199 (0.02%)	1.1	0.40		– / –
ESV01209 (0.04%)	0.3	0.72		– / –
ESV01256 (0.01%)	0.1	0.91		– / –
ESV01297 (0.01%)	0.5	0.63		– / –
ESV01308 (0.02%)	5.3	0.05	*	– / –
ESV01354 (0.01%)	0.4	0.69		– / –
ESV01364 (0.01%)	0.6	0.57		– / –
ESV01367 (0.01%)	14.3	0.005	**	– / –
ESV01381 (0.02%)	1.4	0.32		– / –
ESV01409 (0.01%)	5.9	0.04	*	N / –
ESV01697 (0.02%)	1.6	0.28		– / –
ESV01732 (0.01%)	0.4	0.67		– / –
ESV01811 (0.009%)	3.7	0.09	.	– / –
ESV01892 (0.02%)	2.5	0.16		– / –
ESV01934 (0.009%)	0.4	0.68		– / –
ESV01945 (0.01%)	0.9	0.45		– / –

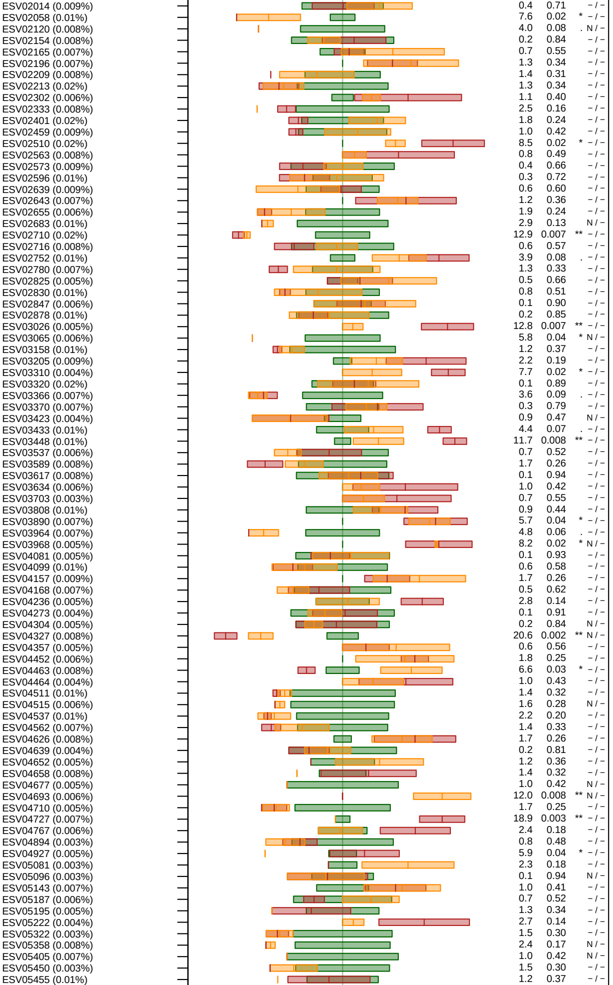

ESV02014 (0.009%) 0.4 0.71 – / –
ESV02058 (0.01%) 7.6 0.02 * – / –
ESV02120 (0.008%) 4.0 0.08 . N / –
ESV02154 (0.008%) 0.2 0.84 – / –
ESV02165 (0.007%) 0.7 0.55 – / –
ESV02196 (0.007%) 1.3 0.34 – / –
ESV02209 (0.008%) 1.4 0.31 – / –
ESV02213 (0.02%) 1.3 0.34 – / –
ESV02302 (0.006%) 1.1 0.40 – / –
ESV02333 (0.008%) 2.5 0.16 – / –
ESV02401 (0.02%) 1.8 0.24 – / –
ESV02459 (0.009%) 1.0 0.42 – / –
ESV02510 (0.02%) 8.5 0.02 * – / –
ESV02563 (0.008%) 0.8 0.49 – / –
ESV02573 (0.009%) 0.4 0.66 – / –
ESV02596 (0.01%) 0.3 0.72 – / –
ESV02639 (0.009%) 0.6 0.60 – / –
ESV02643 (0.007%) 1.2 0.36 – / –
ESV02655 (0.006%) 1.9 0.24 – / –
ESV02683 (0.01%) 2.9 0.13 N / –
ESV02710 (0.02%) 12.9 0.007 ** – / –
ESV02716 (0.008%) 0.6 0.57 – / –
ESV02752 (0.01%) 3.9 0.08 . – / –
ESV02780 (0.007%) 1.3 0.33 – / –
ESV02825 (0.005%) 0.5 0.66 – / –
ESV02830 (0.01%) 0.8 0.51 – / –
ESV02847 (0.006%) 0.1 0.90 – / –
ESV02878 (0.01%) 0.2 0.85 – / –
ESV03026 (0.005%) 12.8 0.007 ** – / –
ESV03065 (0.006%) 5.8 0.04 * N / –
ESV03158 (0.01%) 1.2 0.37 – / –
ESV03205 (0.009%) 2.2 0.19 – / –
ESV03310 (0.004%) 7.7 0.02 * – / –
ESV03320 (0.02%) 0.1 0.89 – / –
ESV03366 (0.007%) 3.6 0.09 . – / –
ESV03370 (0.007%) 0.3 0.79 – / –
ESV03423 (0.004%) 0.9 0.47 N / –
ESV03433 (0.01%) 4.4 0.07 . – / –
ESV03448 (0.01%) 11.7 0.008 ** – / –
ESV03537 (0.006%) 0.7 0.52 – / –
ESV03589 (0.008%) 1.7 0.26 – / –
ESV03617 (0.008%) 0.1 0.94 – / –
ESV03634 (0.006%) 1.0 0.42 – / –
ESV03703 (0.003%) 0.7 0.55 – / –
ESV03808 (0.01%) 0.9 0.44 – / –
ESV03890 (0.007%) 5.7 0.04 * – / –
ESV03964 (0.007%) 4.8 0.06 . – / –
ESV03968 (0.005%) 8.2 0.02 * N / –
ESV04081 (0.005%) 0.1 0.93 – / –
ESV04099 (0.01%) 0.6 0.58 – / –
ESV04157 (0.009%) 1.7 0.26 – / –
ESV04168 (0.007%) 0.5 0.62 – / –
ESV04236 (0.005%) 2.8 0.14 – / –
ESV04273 (0.004%) 0.1 0.91 – / –
ESV04304 (0.005%) 0.2 0.84 N / –
ESV04327 (0.008%) 20.6 0.002 ** N / –
ESV04357 (0.005%) 0.6 0.56 – / –
ESV04452 (0.006%) 1.8 0.25 – / –
ESV04463 (0.008%) 6.6 0.03 * – / –
ESV04464 (0.004%) 1.0 0.43 – / –
ESV04511 (0.01%) 1.4 0.32 – / –
ESV04515 (0.006%) 1.6 0.28 N / –
ESV04537 (0.01%) 2.2 0.20 – / –
ESV04562 (0.007%) 1.4 0.33 – / –
ESV04626 (0.008%) 1.7 0.26 – / –
ESV04639 (0.004%) 0.2 0.81 – / –
ESV04652 (0.005%) 1.2 0.36 – / –
ESV04658 (0.008%) 1.4 0.32 – / –
ESV04677 (0.005%) 1.0 0.42 N / –
ESV04693 (0.006%) 12.0 0.008 ** N / –
ESV04710 (0.005%) 1.7 0.25 – / –
ESV04727 (0.007%) 18.9 0.003 ** – / –
ESV04767 (0.006%) 2.4 0.18 – / –
ESV04894 (0.003%) 0.8 0.48 – / –
ESV04927 (0.005%) 5.9 0.04 * – / –
ESV05081 (0.003%) 2.3 0.18 – / –
ESV05096 (0.003%) 0.1 0.94 N / –
ESV05143 (0.007%) 1.0 0.41 – / –
ESV05187 (0.006%) 0.7 0.52 – / –
ESV05195 (0.005%) 1.3 0.34 – / –
ESV05222 (0.004%) 2.7 0.14 – / –
ESV05322 (0.003%) 1.5 0.30 – / –
ESV05358 (0.008%) 2.4 0.17 N / –
ESV05405 (0.007%) 1.0 0.42 N / –
ESV05450 (0.003%) 1.5 0.30 – / –
ESV05455 (0.01%) 1.2 0.37 – / –

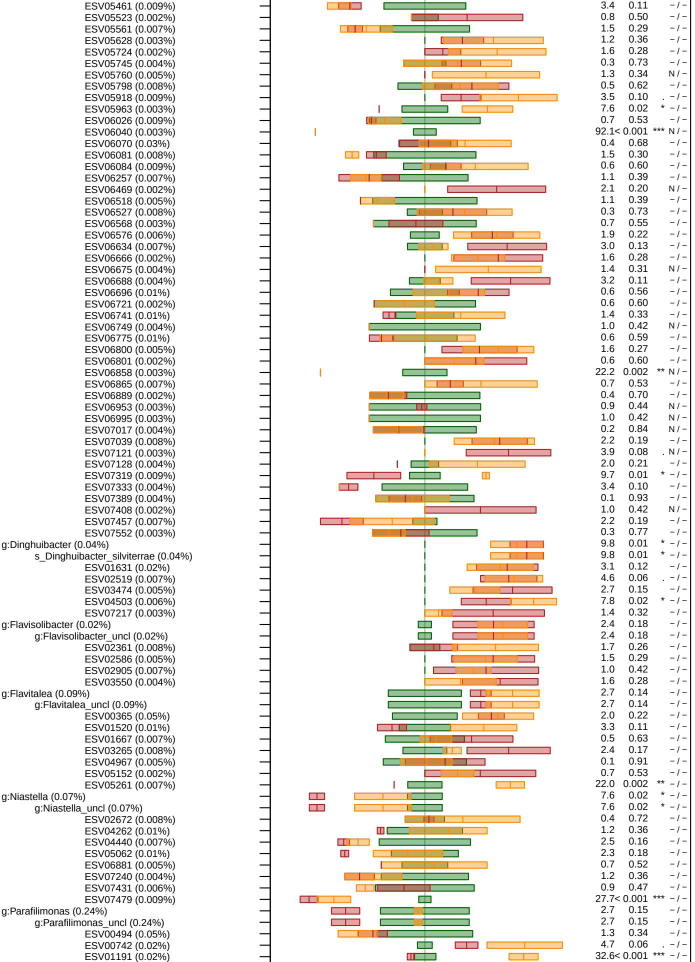

ESV05461 (0.009%) 3.4 0.11 - / -
ESV05523 (0.002%) 0.8 0.50 - / -
ESV05561 (0.007%) 1.5 0.29 - / -
ESV05628 (0.003%) 1.2 0.36 - / -
ESV05724 (0.002%) 1.6 0.28 - / -
ESV05745 (0.004%) 0.3 0.73 - / -
ESV05760 (0.005%) 1.3 0.34 N / -
ESV05798 (0.008%) 0.5 0.62 - / -
ESV05918 (0.009%) 3.5 0.10 . - / -
ESV05963 (0.003%) 7.6 0.02 * - / -
ESV06026 (0.009%) 0.7 0.53 - / -
ESV06040 (0.003%) 92.1< 0.001 *** N / -
ESV06070 (0.03%) 0.4 0.68 - / -
ESV06081 (0.008%) 1.5 0.30 - / -
ESV06084 (0.009%) 0.6 0.60 - / -
ESV06257 (0.007%) 1.1 0.39 - / -
ESV06469 (0.002%) 2.1 0.20 N / -
ESV06518 (0.005%) 1.1 0.39 - / -
ESV06527 (0.008%) 0.3 0.73 - / -
ESV06568 (0.003%) 0.7 0.55 - / -
ESV06576 (0.006%) 1.9 0.22 - / -
ESV06634 (0.007%) 3.0 0.13 - / -
ESV06666 (0.002%) 1.6 0.28 - / -
ESV06675 (0.004%) 1.4 0.31 N / -
ESV06688 (0.004%) 3.2 0.11 - / -
ESV06696 (0.01%) 0.6 0.56 - / -
ESV06721 (0.002%) 0.6 0.60 - / -
ESV06741 (0.01%) 1.4 0.33 - / -
ESV06749 (0.004%) 1.0 0.42 N / -
ESV06775 (0.01%) 0.6 0.59 - / -
ESV06800 (0.005%) 1.6 0.27 - / -
ESV06801 (0.002%) 0.6 0.60 - / -
ESV06858 (0.003%) 22.2 0.002 ** N / -
ESV06865 (0.007%) 0.7 0.53 - / -
ESV06889 (0.002%) 0.4 0.70 - / -
ESV06953 (0.003%) 0.9 0.44 N / -
ESV06995 (0.003%) 1.0 0.42 N / -
ESV07017 (0.004%) 0.2 0.84 N / -
ESV07039 (0.008%) 2.2 0.19 - / -
ESV07121 (0.003%) 3.9 0.08 . N / -
ESV07128 (0.004%) 2.0 0.21 - / -
ESV07319 (0.009%) 9.7 0.01 * - / -
ESV07333 (0.004%) 3.4 0.10 - / -
ESV07389 (0.004%) 0.1 0.93 - / -
ESV07408 (0.002%) 1.0 0.42 N / -
ESV07457 (0.007%) 2.2 0.19 - / -
ESV07552 (0.003%) 0.3 0.77 - / -
g:Dinghuibacter (0.04%) 9.8 0.01 * - / -
s_Dinghuibacter_silviterrae (0.04%) 9.8 0.01 * - / -
ESV01631 (0.02%) 3.1 0.12 - / -
ESV02519 (0.007%) 4.6 0.06 . - / -
ESV03474 (0.005%) 2.7 0.15 - / -
ESV04503 (0.006%) 7.8 0.02 * - / -
ESV07217 (0.003%) 1.4 0.32 - / -
g:Flavisolibacter (0.02%) 2.4 0.18 - / -
g:Flavisolibacter_uncl (0.02%) 2.4 0.18 - / -
ESV02361 (0.008%) 1.7 0.26 - / -
ESV02586 (0.005%) 1.5 0.29 - / -
ESV02905 (0.007%) 1.0 0.42 - / -
ESV03550 (0.004%) 1.6 0.28 - / -
g:Flavitalea (0.09%) 2.7 0.14 - / -
g:Flavitalea_uncl (0.09%) 2.7 0.14 - / -
ESV00365 (0.05%) 2.0 0.22 - / -
ESV01520 (0.01%) 3.3 0.11 - / -
ESV01667 (0.007%) 0.5 0.63 - / -
ESV03265 (0.008%) 2.4 0.17 - / -
ESV04967 (0.005%) 0.1 0.91 - / -
ESV05152 (0.002%) 0.7 0.53 - / -
ESV05261 (0.007%) 22.0 0.002 ** - / -
g:Niastella (0.07%) 7.6 0.02 * - / -
g:Niastella_uncl (0.07%) 7.6 0.02 * - / -
ESV02672 (0.008%) 0.4 0.72 - / -
ESV04262 (0.01%) 1.2 0.36 - / -
ESV04440 (0.007%) 2.5 0.16 - / -
ESV05062 (0.01%) 2.3 0.18 - / -
ESV06881 (0.005%) 0.7 0.52 - / -
ESV07240 (0.004%) 1.2 0.36 - / -
ESV07431 (0.006%) 0.9 0.47 - / -
ESV07479 (0.009%) 27.7< 0.001 *** - / -
g:Parafilimonas (0.24%) 2.7 0.15 - / -
g:Parafilimonas_uncl (0.24%) 2.7 0.15 - / -
ESV00494 (0.05%) 1.3 0.34 - / -
ESV00742 (0.02%) 4.7 0.06 . - / -
ESV01191 (0.02%) 32.6< 0.001 *** - / -

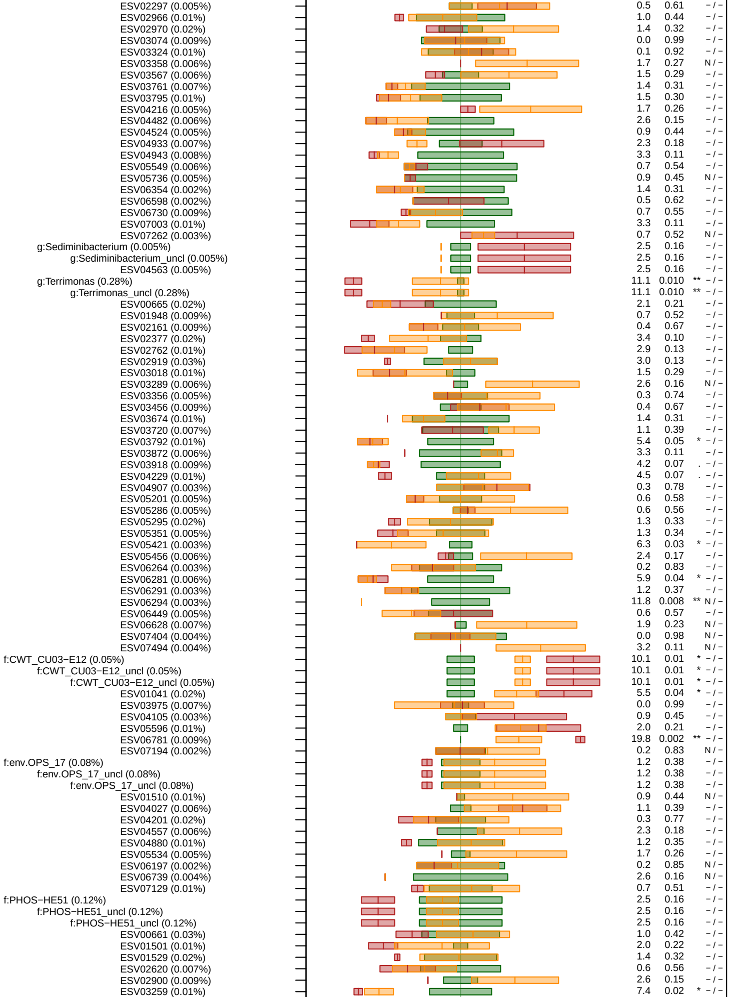

ESV02297 (0.005%) 0.5 0.61 – / –
ESV02966 (0.01%) 1.0 0.44 – / –
ESV02970 (0.02%) 1.4 0.32 – / –
ESV03074 (0.009%) 0.0 0.99 – / –
ESV03324 (0.01%) 0.1 0.92 – / –
ESV03358 (0.006%) 1.7 0.27 N / –
ESV03567 (0.006%) 1.5 0.29 – / –
ESV03761 (0.007%) 1.4 0.31 – / –
ESV03795 (0.01%) 1.5 0.30 – / –
ESV04216 (0.005%) 1.7 0.26 – / –
ESV04482 (0.006%) 2.6 0.15 – / –
ESV04524 (0.005%) 0.9 0.44 – / –
ESV04933 (0.007%) 2.3 0.18 – / –
ESV04943 (0.008%) 3.3 0.11 – / –
ESV05549 (0.006%) 0.7 0.54 – / –
ESV05736 (0.005%) 0.9 0.45 N / –
ESV06354 (0.002%) 1.4 0.31 – / –
ESV06598 (0.002%) 0.5 0.62 – / –
ESV06730 (0.009%) 0.7 0.55 – / –
ESV07003 (0.01%) 3.3 0.11 – / –
ESV07262 (0.003%) 0.7 0.52 N / –
g:Sediminibacterium (0.005%) 2.5 0.16 – / –
g:Sediminibacterium_uncl (0.005%) 2.5 0.16 – / –
ESV04563 (0.005%) 2.5 0.16 – / –
g:Terrimonas (0.28%) 11.1 0.010 ** – / –
g:Terrimonas_uncl (0.28%) 11.1 0.010 ** – / –
ESV00665 (0.02%) 2.1 0.21 – / –
ESV01948 (0.009%) 0.7 0.52 – / –
ESV02161 (0.009%) 0.4 0.67 – / –
ESV02377 (0.02%) 3.4 0.10 – / –
ESV02762 (0.01%) 2.9 0.13 – / –
ESV02919 (0.03%) 3.0 0.13 – / –
ESV03018 (0.01%) 1.5 0.29 – / –
ESV03289 (0.006%) 2.6 0.16 N / –
ESV03356 (0.005%) 0.3 0.74 – / –
ESV03456 (0.009%) 0.4 0.67 – / –
ESV03674 (0.01%) 1.4 0.31 – / –
ESV03720 (0.007%) 1.1 0.39 – / –
ESV03792 (0.01%) 5.4 0.05 * – / –
ESV03872 (0.006%) 3.3 0.11 – / –
ESV03918 (0.009%) 4.2 0.07 . – / –
ESV04229 (0.01%) 4.5 0.07 . – / –
ESV04907 (0.003%) 0.3 0.78 – / –
ESV05201 (0.005%) 0.6 0.58 – / –
ESV05286 (0.005%) 0.6 0.56 – / –
ESV05295 (0.02%) 1.3 0.33 – / –
ESV05351 (0.005%) 1.3 0.34 – / –
ESV05421 (0.003%) 6.3 0.03 * – / –
ESV05456 (0.006%) 2.4 0.17 – / –
ESV06264 (0.003%) 0.2 0.83 – / –
ESV06281 (0.006%) 5.9 0.04 * – / –
ESV06291 (0.003%) 1.2 0.37 – / –
ESV06294 (0.003%) 11.8 0.008 ** N / –
ESV06449 (0.005%) 0.6 0.57 – / –
ESV06628 (0.007%) 1.9 0.23 N / –
ESV07404 (0.004%) 0.0 0.98 N / –
ESV07494 (0.004%) 3.2 0.11 N / –
f:CWT_CU03–E12 (0.05%) 10.1 0.01 * – / –
f:CWT_CU03–E12_uncl (0.05%) 10.1 0.01 * – / –
f:CWT_CU03–E12_uncl (0.05%) 10.1 0.01 * – / –
ESV01041 (0.02%) 5.5 0.04 * – / –
ESV03975 (0.007%) 0.0 0.99 – / –
ESV04105 (0.003%) 0.9 0.45 – / –
ESV05596 (0.01%) 2.0 0.21 – / –
ESV06781 (0.009%) 19.8 0.002 ** – / –
ESV07194 (0.002%) 0.2 0.83 N / –
f:env.OPS_17 (0.08%) 1.2 0.38 – / –
f:env.OPS_17_uncl (0.08%) 1.2 0.38 – / –
f:env.OPS_17_uncl (0.08%) 1.2 0.38 – / –
ESV01510 (0.01%) 0.9 0.44 N / –
ESV04027 (0.006%) 1.1 0.39 – / –
ESV04201 (0.02%) 0.3 0.77 – / –
ESV04557 (0.006%) 2.3 0.18 – / –
ESV04880 (0.01%) 1.2 0.35 – / –
ESV05534 (0.005%) 1.7 0.26 – / –
ESV06197 (0.002%) 0.2 0.85 N / –
ESV06739 (0.004%) 2.6 0.16 N / –
ESV07129 (0.01%) 0.7 0.51 – / –
f:PHOS–HE51 (0.12%) 2.5 0.16 – / –
f:PHOS–HE51_uncl (0.12%) 2.5 0.16 – / –
f:PHOS–HE51_uncl (0.12%) 2.5 0.16 – / –
ESV00661 (0.03%) 1.0 0.42 – / –
ESV01501 (0.01%) 2.0 0.22 – / –
ESV01529 (0.02%) 1.4 0.32 – / –
ESV02620 (0.007%) 0.6 0.56 – / –
ESV02900 (0.009%) 2.6 0.15 – / –
ESV03259 (0.01%) 7.4 0.02 * – / –

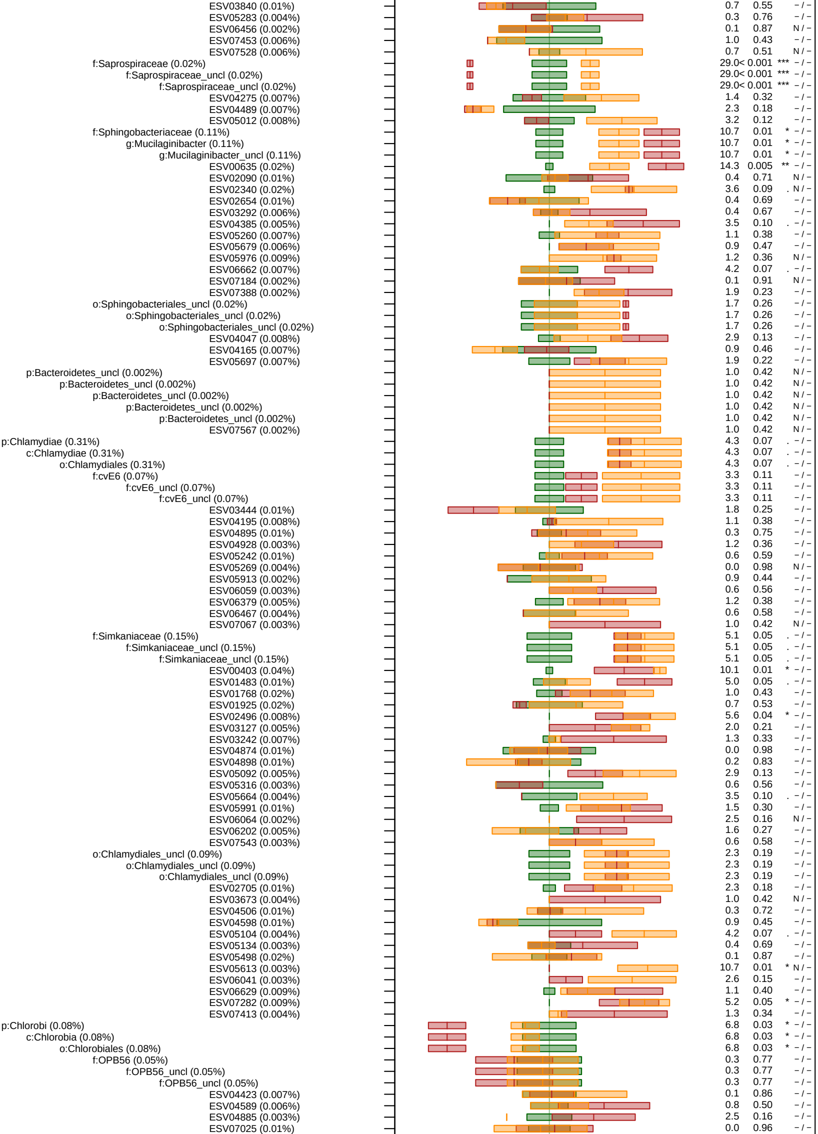
ESV03840 (0.01%) 0.7 0.55 – / –
ESV05283 (0.004%) 0.3 0.76 – / –
ESV06456 (0.002%) 0.1 0.87 N / –
ESV07453 (0.006%) 1.0 0.43 – / –
ESV07528 (0.006%) 0.7 0.51 N / –
f:Saprospiraceae (0.02%) 29.0 < 0.001 *** – / –
f:Saprospiraceae_uncl (0.02%) 29.0 < 0.001 *** – / –
f:Saprospiraceae_uncl (0.02%) 29.0 < 0.001 *** – / –
ESV04275 (0.007%) 1.4 0.32 – / –
ESV04489 (0.007%) 2.3 0.18 – / –
ESV05012 (0.008%) 3.2 0.12 – / –
f:Sphingobacteriaceae (0.11%) 10.7 0.01 * – / –
g:Mucilaginibacter (0.11%) 10.7 0.01 * – / –
g:Mucilaginibacter_uncl (0.11%) 10.7 0.01 * – / –
ESV00635 (0.02%) 14.3 0.005 ** – / –
ESV02090 (0.01%) 0.4 0.71 N / –
ESV02340 (0.02%) 3.6 0.09 . N / –
ESV02654 (0.01%) 0.4 0.69 – / –
ESV03292 (0.006%) 0.4 0.67 – / –
ESV04385 (0.005%) 3.5 0.10 . – / –
ESV05260 (0.007%) 1.1 0.38 – / –
ESV05679 (0.006%) 0.9 0.47 – / –
ESV05976 (0.009%) 1.2 0.36 N / –
ESV06662 (0.007%) 4.2 0.07 . – / –
ESV07184 (0.002%) 0.1 0.91 N / –
ESV07388 (0.002%) 1.9 0.23 – / –
o:Sphingobacteriales_uncl (0.02%) 1.7 0.26 – / –
o:Sphingobacteriales_uncl (0.02%) 1.7 0.26 – / –
o:Sphingobacteriales_uncl (0.02%) 1.7 0.26 – / –
ESV04047 (0.008%) 2.9 0.13 – / –
ESV04165 (0.007%) 0.9 0.46 – / –
ESV05697 (0.007%) 1.9 0.22 – / –
p:Bacteroidetes_uncl (0.002%) 1.0 0.42 N / –
p:Bacteroidetes_uncl (0.002%) 1.0 0.42 N / –
p:Bacteroidetes_uncl (0.002%) 1.0 0.42 N / –
p:Bacteroidetes_uncl (0.002%) 1.0 0.42 N / –
p:Bacteroidetes_uncl (0.002%) 1.0 0.42 N / –
ESV07567 (0.002%) 1.0 0.42 N / –
p:Chlamydiae (0.31%) 4.3 0.07 . – / –
c:Chlamydiae (0.31%) 4.3 0.07 . – / –
o:Chlamydiales (0.31%) 4.3 0.07 . – / –
f:cvE6 (0.07%) 3.3 0.11 – / –
f:cvE6_uncl (0.07%) 3.3 0.11 – / –
f:cvE6_uncl (0.07%) 3.3 0.11 – / –
ESV03444 (0.01%) 1.8 0.25 – / –
ESV04195 (0.008%) 1.1 0.38 – / –
ESV04895 (0.01%) 0.3 0.75 – / –
ESV04928 (0.003%) 1.2 0.36 – / –
ESV05242 (0.01%) 0.6 0.59 – / –
ESV05269 (0.004%) 0.0 0.98 N / –
ESV05913 (0.002%) 0.9 0.44 – / –
ESV06059 (0.003%) 0.6 0.56 – / –
ESV06379 (0.005%) 1.2 0.38 – / –
ESV06467 (0.004%) 0.6 0.58 – / –
ESV07067 (0.003%) 1.0 0.42 N / –
f:Simkaniaceae (0.15%) 5.1 0.05 . – / –
f:Simkaniaceae_uncl (0.15%) 5.1 0.05 . – / –
f:Simkaniaceae_uncl (0.15%) 5.1 0.05 . – / –
ESV00403 (0.04%) 10.1 0.01 * – / –
ESV01483 (0.01%) 5.0 0.05 . – / –
ESV01768 (0.02%) 1.0 0.43 – / –
ESV01925 (0.02%) 0.7 0.53 – / –
ESV02496 (0.008%) 5.6 0.04 * – / –
ESV03127 (0.005%) 2.0 0.21 – / –
ESV03242 (0.007%) 1.3 0.33 – / –
ESV04874 (0.01%) 0.0 0.98 – / –
ESV04898 (0.01%) 0.2 0.83 – / –
ESV05092 (0.005%) 2.9 0.13 – / –
ESV05316 (0.003%) 0.6 0.56 – / –
ESV05664 (0.004%) 3.5 0.10 . – / –
ESV05991 (0.01%) 1.5 0.30 – / –
ESV06064 (0.002%) 2.5 0.16 N / –
ESV06202 (0.005%) 1.6 0.27 – / –
ESV07543 (0.003%) 0.6 0.58 – / –
o:Chlamydiales_uncl (0.09%) 2.3 0.19 – / –
o:Chlamydiales_uncl (0.09%) 2.3 0.19 – / –
o:Chlamydiales_uncl (0.09%) 2.3 0.19 – / –
ESV02705 (0.01%) 2.3 0.18 – / –
ESV03673 (0.004%) 1.0 0.42 N / –
ESV04506 (0.01%) 0.3 0.72 – / –
ESV04598 (0.01%) 0.9 0.45 – / –
ESV05104 (0.004%) 4.2 0.07 . – / –
ESV05134 (0.003%) 0.4 0.69 – / –
ESV05498 (0.02%) 0.1 0.87 – / –
ESV05613 (0.003%) 10.7 0.01 * N / –
ESV06041 (0.003%) 2.6 0.15 – / –
ESV06629 (0.009%) 1.1 0.40 – / –
ESV07282 (0.009%) 5.2 0.05 * – / –
ESV07413 (0.004%) 1.3 0.34 – / –
p:Chlorobi (0.08%) 6.8 0.03 * – / –
c:Chlorobia (0.08%) 6.8 0.03 * – / –
o:Chlorobiales (0.08%) 6.8 0.03 * – / –
f:OPB56 (0.05%) 0.3 0.77 – / –
f:OPB56_uncl (0.05%) 0.3 0.77 – / –
f:OPB56_uncl (0.05%) 0.3 0.77 – / –
ESV04423 (0.007%) 0.1 0.86 – / –
ESV04589 (0.006%) 0.8 0.50 – / –
ESV04885 (0.003%) 2.5 0.16 – / –
ESV07025 (0.01%) 0.0 0.96 – / –

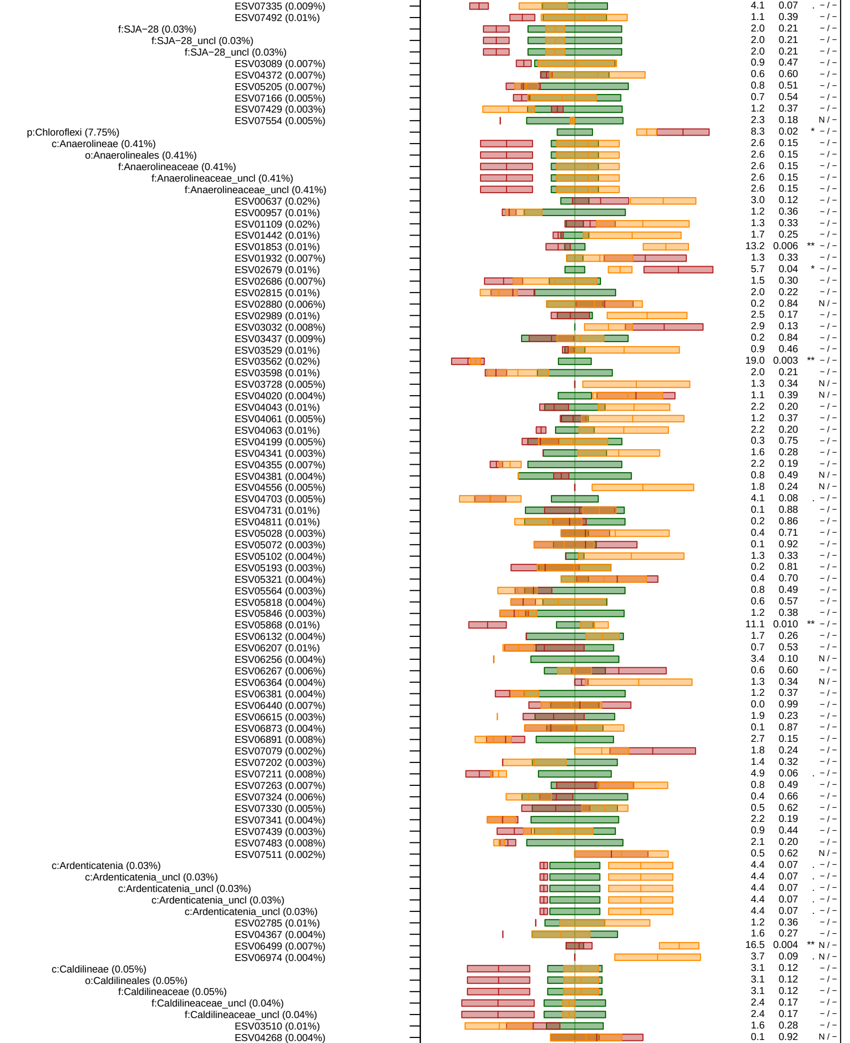

ESV07335 (0.009%) 4.1 0.07 . - / -
ESV07492 (0.01%) 1.1 0.39 - / -
f:SJA−28 (0.03%) 2.0 0.21 - / -
f:SJA−28_uncl (0.03%) 2.0 0.21 - / -
f:SJA−28_uncl (0.03%) 2.0 0.21 - / -
ESV03089 (0.007%) 0.9 0.47 - / -
ESV04372 (0.007%) 0.6 0.60 - / -
ESV05205 (0.007%) 0.8 0.51 - / -
ESV07166 (0.005%) 0.7 0.54 - / -
ESV07429 (0.003%) 1.2 0.37 - / -
ESV07554 (0.005%) 2.3 0.18 N / -
p:Chloroflexi (7.75%) 8.3 0.02 * - / -
c:Anaerolineae (0.41%) 2.6 0.15 - / -
o:Anaerolineales (0.41%) 2.6 0.15 - / -
f:Anaerolineaceae (0.41%) 2.6 0.15 - / -
f:Anaerolineaceae_uncl (0.41%) 2.6 0.15 - / -
f:Anaerolineaceae_uncl (0.41%) 2.6 0.15 - / -
ESV00637 (0.02%) 3.0 0.12 - / -
ESV00957 (0.01%) 1.2 0.36 - / -
ESV01109 (0.02%) 1.3 0.33 - / -
ESV01442 (0.01%) 1.7 0.25 - / -
ESV01853 (0.01%) 13.2 0.006 ** - / -
ESV01932 (0.007%) 1.3 0.33 - / -
ESV02679 (0.01%) 5.7 0.04 * - / -
ESV02686 (0.007%) 1.5 0.30 - / -
ESV02815 (0.01%) 2.0 0.22 - / -
ESV02880 (0.006%) 0.2 0.84 N / -
ESV02989 (0.01%) 2.5 0.17 - / -
ESV03032 (0.008%) 2.9 0.13 - / -
ESV03437 (0.009%) 0.2 0.84 - / -
ESV03529 (0.01%) 0.9 0.46 - / -
ESV03562 (0.02%) 19.0 0.003 ** - / -
ESV03598 (0.01%) 2.0 0.21 - / -
ESV03728 (0.005%) 1.3 0.34 N / -
ESV04020 (0.004%) 1.1 0.39 N / -
ESV04043 (0.01%) 2.2 0.20 - / -
ESV04061 (0.005%) 1.2 0.37 - / -
ESV04063 (0.01%) 2.2 0.20 - / -
ESV04199 (0.005%) 0.3 0.75 - / -
ESV04341 (0.003%) 1.6 0.28 - / -
ESV04355 (0.007%) 2.2 0.19 - / -
ESV04381 (0.004%) 0.8 0.49 N / -
ESV04556 (0.005%) 1.8 0.24 N / -
ESV04703 (0.005%) 4.1 0.08 . - / -
ESV04731 (0.01%) 0.1 0.88 - / -
ESV04811 (0.01%) 0.2 0.86 - / -
ESV05028 (0.003%) 0.4 0.71 - / -
ESV05072 (0.003%) 0.1 0.92 - / -
ESV05102 (0.004%) 1.3 0.33 - / -
ESV05193 (0.003%) 0.2 0.81 - / -
ESV05321 (0.004%) 0.4 0.70 - / -
ESV05564 (0.003%) 0.8 0.49 - / -
ESV05818 (0.004%) 0.6 0.57 - / -
ESV05846 (0.003%) 1.2 0.38 - / -
ESV05868 (0.01%) 11.1 0.010 ** - / -
ESV06132 (0.004%) 1.7 0.26 - / -
ESV06207 (0.01%) 0.7 0.53 - / -
ESV06256 (0.004%) 3.4 0.10 N / -
ESV06267 (0.006%) 0.6 0.60 - / -
ESV06364 (0.004%) 1.3 0.34 N / -
ESV06381 (0.004%) 1.2 0.37 - / -
ESV06440 (0.007%) 0.0 0.99 - / -
ESV06615 (0.003%) 1.9 0.23 - / -
ESV06873 (0.004%) 0.1 0.87 - / -
ESV06891 (0.008%) 2.7 0.15 - / -
ESV07079 (0.002%) 1.8 0.24 - / -
ESV07202 (0.003%) 1.4 0.32 - / -
ESV07211 (0.008%) 4.9 0.06 . - / -
ESV07263 (0.007%) 0.8 0.49 - / -
ESV07324 (0.006%) 0.4 0.66 - / -
ESV07330 (0.005%) 0.5 0.62 - / -
ESV07341 (0.004%) 2.2 0.19 - / -
ESV07439 (0.003%) 0.9 0.44 - / -
ESV07483 (0.008%) 2.1 0.20 - / -
ESV07511 (0.002%) 0.5 0.62 N / -
c:Ardenticatenia (0.03%) 4.4 0.07 . - / -
c:Ardenticatenia_uncl (0.03%) 4.4 0.07 . - / -
c:Ardenticatenia_uncl (0.03%) 4.4 0.07 . - / -
c:Ardenticatenia_uncl (0.03%) 4.4 0.07 . - / -
c:Ardenticatenia_uncl (0.03%) 4.4 0.07 . - / -
ESV02785 (0.01%) 1.2 0.36 - / -
ESV04367 (0.004%) 1.6 0.27 - / -
ESV06499 (0.007%) 16.5 0.004 ** N / -
ESV06974 (0.004%) 3.7 0.09 . N / -
c:Caldilineae (0.05%) 3.1 0.12 - / -
o:Caldilineales (0.05%) 3.1 0.12 - / -
f:Caldilineaceae (0.05%) 3.1 0.12 - / -
f:Caldilineaceae_uncl (0.04%) 2.4 0.17 - / -
f:Caldilineaceae_uncl (0.04%) 2.4 0.17 - / -
ESV03510 (0.01%) 1.6 0.28 - / -
ESV04268 (0.004%) 0.1 0.92 N / -

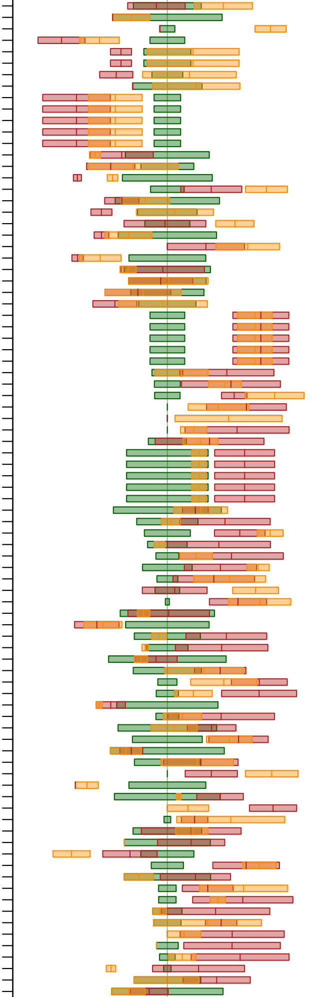

ESV04772 (0.003%)	1.4	0.32		− / −
ESV06752 (0.002%)	0.7	0.53		− / −
ESV06755 (0.008%)	38.4	< 0.001	***	− / −
ESV07499 (0.01%)	6.9	0.03	*	− / −
g:Litorilinea (0.01%)	1.4	0.31		− / −
g:Litorilinea_uncl (0.01%)	1.4	0.31		− / −
ESV02975 (0.008%)	1.6	0.28		− / −
ESV05800 (0.003%)	1.0	0.43		− / −
c:Chloroflexia (0.13%)	3.1	0.12		− / −
o:Chloroflexales (0.13%)	3.1	0.12		− / −
f:Roseiflexaceae (0.13%)	3.1	0.12		− / −
g:Roseiflexus (0.13%)	3.1	0.12		− / −
g:Roseiflexus_uncl (0.13%)	3.1	0.12		− / −
ESV00894 (0.02%)	1.4	0.31		− / −
ESV02611 (0.02%)	0.7	0.52		− / −
ESV02618 (0.01%)	3.0	0.13		− / −
ESV03730 (0.005%)	4.5	0.06	.	− / −
ESV03780 (0.008%)	0.4	0.67		− / −
ESV04028 (0.01%)	2.0	0.22		− / −
ESV04041 (0.005%)	1.9	0.24		− / −
ESV04554 (0.006%)	1.1	0.39		− / −
ESV04920 (0.005%)	1.9	0.22		− / −
ESV05078 (0.01%)	3.4	0.10		− / −
ESV05491 (0.01%)	0.4	0.70		− / −
ESV06114 (0.003%)	0.0	0.99		N / −
ESV06215 (0.003%)	0.2	0.83		N / −
ESV06943 (0.01%)	0.8	0.50		− / −
c:JG30−KF−CM66 (0.07%)	5.9	0.04	*	− / −
c:JG30−KF−CM66_uncl (0.07%)	5.9	0.04	*	− / −
c:JG30−KF−CM66_uncl (0.07%)	5.9	0.04	*	− / −
c:JG30−KF−CM66_uncl (0.07%)	5.9	0.04	*	− / −
c:JG30−KF−CM66_uncl (0.07%)	5.9	0.04	*	− / −
ESV00520 (0.02%)	0.9	0.45		− / −
ESV01893 (0.01%)	1.3	0.34		− / −
ESV02566 (0.02%)	7.4	0.02	*	− / −
ESV04526 (0.003%)	1.9	0.23		− / −
ESV04641 (0.005%)	1.3	0.34		N / −
ESV06239 (0.003%)	1.3	0.34		− / −
ESV06370 (0.005%)	0.4	0.68		− / −
c:JG37−AG−4 (0.75%)	1.7	0.25		− / −
c:JG37−AG−4_uncl (0.75%)	1.7	0.25		− / −
c:JG37−AG−4_uncl (0.75%)	1.7	0.25		− / −
c:JG37−AG−4_uncl (0.75%)	1.7	0.25		− / −
c:JG37−AG−4_uncl (0.75%)	1.7	0.25		− / −
ESV00074 (0.08%)	0.3	0.78		− / −
ESV00108 (0.06%)	1.0	0.42		− / −
ESV00199 (0.04%)	6.3	0.03	*	− / −
ESV00415 (0.03%)	1.0	0.42		− / −
ESV00446 (0.03%)	1.0	0.42		− / −
ESV00488 (0.02%)	3.1	0.12		− / −
ESV00491 (0.02%)	1.0	0.41		− / −
ESV00639 (0.02%)	4.2	0.07	.	− / −
ESV00660 (0.02%)	3.9	0.08	.	− / −
ESV00728 (0.02%)	0.2	0.86		− / −
ESV00869 (0.02%)	1.8	0.25		− / −
ESV00880 (0.01%)	1.5	0.31		− / −
ESV00938 (0.02%)	1.8	0.24		− / −
ESV00953 (0.02%)	0.1	0.88		− / −
ESV00973 (0.02%)	0.7	0.56		− / −
ESV01019 (0.01%)	3.3	0.11		− / −
ESV01062 (0.01%)	3.6	0.10	.	− / −
ESV01212 (0.02%)	1.4	0.31		− / −
ESV01269 (0.01%)	0.7	0.54		− / −
ESV01573 (0.01%)	0.5	0.64		− / −
ESV01578 (0.01%)	1.7	0.26		− / −
ESV01617 (0.01%)	0.4	0.67		− / −
ESV01882 (0.007%)	0.3	0.77		− / −
ESV01894 (0.01%)	6.0	0.04	*	− / −
ESV01940 (0.009%)	4.7	0.06	.	− / −
ESV02330 (0.009%)	0.7	0.54		− / −
ESV02458 (0.008%)	9.6	0.01	*	− / −
ESV02594 (0.01%)	1.0	0.43		− / −
ESV02647 (0.008%)	0.1	0.86		− / −
ESV02745 (0.005%)	1.2	0.38		− / −
ESV02793 (0.01%)	3.9	0.08	.	− / −
ESV03228 (0.006%)	4.5	0.06	.	− / −
ESV03359 (0.004%)	0.7	0.52		N / −
ESV03375 (0.007%)	1.6	0.27		− / −
ESV03378 (0.008%)	1.7	0.26		− / −
ESV03518 (0.005%)	0.9	0.47		− / −
ESV03555 (0.004%)	0.7	0.53		− / −
ESV03578 (0.006%)	1.1	0.38		− / −
ESV03911 (0.004%)	2.0	0.21		− / −
ESV03927 (0.006%)	1.7	0.26		− / −
ESV04473 (0.02%)	2.7	0.14		− / −
ESV04682 (0.004%)	0.8	0.51		N / −
ESV04745 (0.003%)	0.3	0.75		− / −

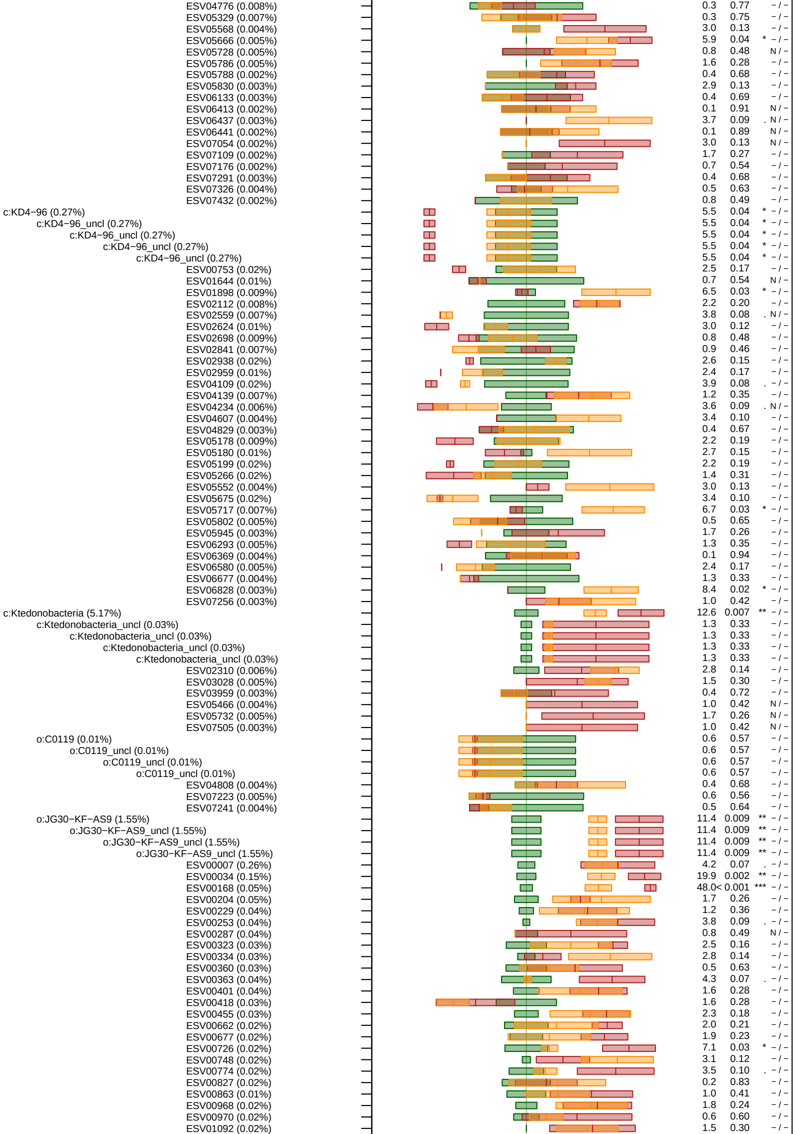

ESV04776 (0.008%) 0.3 0.77 - / -
ESV05329 (0.007%) 0.3 0.75 - / -
ESV05568 (0.004%) 3.0 0.13 - / -
ESV05666 (0.005%) 5.9 0.04 * - / -
ESV05728 (0.005%) 0.8 0.48 N / -
ESV05786 (0.005%) 1.6 0.28 - / -
ESV05788 (0.002%) 0.4 0.68 - / -
ESV05830 (0.003%) 2.9 0.13 - / -
ESV06133 (0.003%) 0.4 0.69 - / -
ESV06413 (0.002%) 0.1 0.91 N / -
ESV06437 (0.003%) 3.7 0.09 . N / -
ESV06441 (0.002%) 0.1 0.89 N / -
ESV07054 (0.002%) 3.0 0.13 N / -
ESV07109 (0.002%) 1.7 0.27 - / -
ESV07176 (0.002%) 0.7 0.54 - / -
ESV07291 (0.003%) 0.4 0.68 - / -
ESV07326 (0.004%) 0.5 0.63 - / -
ESV07432 (0.002%) 0.8 0.49 - / -
c:KD4−96 (0.27%) 5.5 0.04 * - / -
c:KD4−96_uncl (0.27%) 5.5 0.04 * - / -
c:KD4−96_uncl (0.27%) 5.5 0.04 * - / -
c:KD4−96_uncl (0.27%) 5.5 0.04 * - / -
c:KD4−96_uncl (0.27%) 5.5 0.04 * - / -
ESV00753 (0.02%) 2.5 0.17 - / -
ESV01644 (0.01%) 0.7 0.54 N / -
ESV01898 (0.009%) 6.5 0.03 * - / -
ESV02112 (0.008%) 2.2 0.20 - / -
ESV02559 (0.007%) 3.8 0.08 . N / -
ESV02624 (0.01%) 3.0 0.12 - / -
ESV02698 (0.009%) 0.8 0.48 - / -
ESV02841 (0.007%) 0.9 0.46 - / -
ESV02938 (0.02%) 2.6 0.15 - / -
ESV02959 (0.01%) 2.4 0.17 - / -
ESV04109 (0.02%) 3.9 0.08 . - / -
ESV04139 (0.007%) 1.2 0.35 - / -
ESV04234 (0.006%) 3.6 0.09 . N / -
ESV04607 (0.004%) 3.4 0.10 - / -
ESV04829 (0.003%) 0.4 0.67 - / -
ESV05178 (0.009%) 2.2 0.19 - / -
ESV05180 (0.01%) 2.7 0.15 - / -
ESV05199 (0.02%) 2.2 0.19 - / -
ESV05266 (0.02%) 1.4 0.31 - / -
ESV05552 (0.004%) 3.0 0.13 - / -
ESV05675 (0.02%) 3.4 0.10 - / -
ESV05717 (0.007%) 6.7 0.03 * - / -
ESV05802 (0.005%) 0.5 0.65 - / -
ESV05945 (0.003%) 1.7 0.26 - / -
ESV06293 (0.005%) 1.3 0.35 - / -
ESV06369 (0.004%) 0.1 0.94 - / -
ESV06580 (0.005%) 2.4 0.17 - / -
ESV06677 (0.004%) 1.3 0.33 - / -
ESV06828 (0.003%) 8.4 0.02 * - / -
ESV07256 (0.003%) 1.0 0.42 - / -
c:Ktedonobacteria (5.17%) 12.6 0.007 ** - / -
c:Ktedonobacteria_uncl (0.03%) 1.3 0.33 - / -
c:Ktedonobacteria_uncl (0.03%) 1.3 0.33 - / -
c:Ktedonobacteria_uncl (0.03%) 1.3 0.33 - / -
c:Ktedonobacteria_uncl (0.03%) 1.3 0.33 - / -
ESV02310 (0.006%) 2.8 0.14 - / -
ESV03028 (0.005%) 1.5 0.30 - / -
ESV03959 (0.003%) 0.4 0.72 - / -
ESV05466 (0.004%) 1.0 0.42 N / -
ESV05732 (0.005%) 1.7 0.26 N / -
ESV07505 (0.003%) 1.0 0.42 N / -
o:C0119 (0.01%) 0.6 0.57 - / -
o:C0119_uncl (0.01%) 0.6 0.57 - / -
o:C0119_uncl (0.01%) 0.6 0.57 - / -
o:C0119_uncl (0.01%) 0.6 0.57 - / -
ESV04808 (0.004%) 0.4 0.68 - / -
ESV07223 (0.005%) 0.6 0.56 - / -
ESV07241 (0.004%) 0.5 0.64 - / -
o:JG30−KF−AS9 (1.55%) 11.4 0.009 ** - / -
o:JG30−KF−AS9_uncl (1.55%) 11.4 0.009 ** - / -
o:JG30−KF−AS9_uncl (1.55%) 11.4 0.009 ** - / -
o:JG30−KF−AS9_uncl (1.55%) 11.4 0.009 ** - / -
ESV00007 (0.26%) 4.2 0.07 . - / -
ESV00034 (0.15%) 19.9 0.002 ** - / -
ESV00168 (0.05%) 48.0 < 0.001 *** - / -
ESV00204 (0.05%) 1.7 0.26 - / -
ESV00229 (0.04%) 1.2 0.36 - / -
ESV00253 (0.04%) 3.8 0.09 . - / -
ESV00287 (0.04%) 0.8 0.49 N / -
ESV00323 (0.03%) 2.5 0.16 - / -
ESV00334 (0.03%) 2.8 0.14 - / -
ESV00360 (0.03%) 0.5 0.63 - / -
ESV00363 (0.04%) 4.3 0.07 . - / -
ESV00401 (0.04%) 1.6 0.28 - / -
ESV00418 (0.03%) 1.6 0.28 - / -
ESV00455 (0.03%) 2.3 0.18 - / -
ESV00662 (0.02%) 2.0 0.21 - / -
ESV00677 (0.02%) 1.9 0.23 - / -
ESV00726 (0.02%) 7.1 0.03 * - / -
ESV00748 (0.02%) 3.1 0.12 - / -
ESV00774 (0.02%) 3.5 0.10 . - / -
ESV00827 (0.02%) 0.2 0.83 - / -
ESV00863 (0.01%) 1.0 0.41 - / -
ESV00968 (0.02%) 1.8 0.24 - / -
ESV00970 (0.02%) 0.6 0.60 - / -
ESV01092 (0.02%) 1.5 0.30 - / -

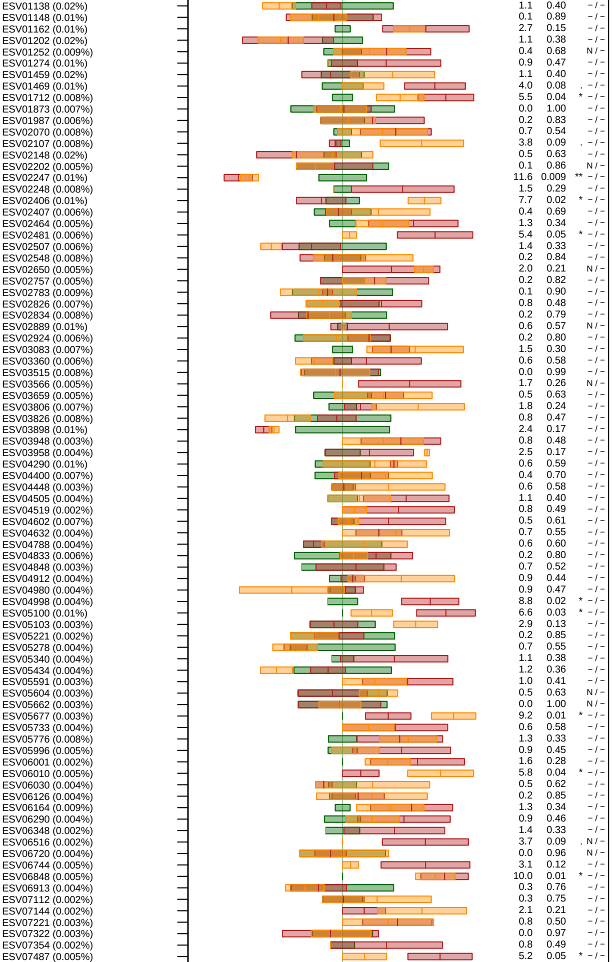
ESV01138 (0.02%) 1.1 0.40 – / –
ESV01148 (0.01%) 0.1 0.89 – / –
ESV01162 (0.01%) 2.7 0.15 – / –
ESV01202 (0.02%) 1.1 0.38 – / –
ESV01252 (0.009%) 0.4 0.68 N / –
ESV01274 (0.01%) 0.9 0.47 – / –
ESV01459 (0.02%) 1.1 0.40 – / –
ESV01469 (0.01%) 4.0 0.08 . – / –
ESV01712 (0.008%) 5.5 0.04 * – / –
ESV01873 (0.007%) 0.0 1.00 – / –
ESV01987 (0.006%) 0.2 0.83 – / –
ESV02070 (0.008%) 0.7 0.54 – / –
ESV02107 (0.008%) 3.8 0.09 . – / –
ESV02148 (0.02%) 0.5 0.63 – / –
ESV02202 (0.005%) 0.1 0.86 N / –
ESV02247 (0.01%) 11.6 0.009 ** – / –
ESV02248 (0.008%) 1.5 0.29 – / –
ESV02406 (0.01%) 7.7 0.02 * – / –
ESV02407 (0.006%) 0.4 0.69 – / –
ESV02464 (0.005%) 1.3 0.34 – / –
ESV02481 (0.006%) 5.4 0.05 * – / –
ESV02507 (0.006%) 1.4 0.33 – / –
ESV02548 (0.008%) 0.2 0.84 – / –
ESV02650 (0.005%) 2.0 0.21 N / –
ESV02757 (0.005%) 0.2 0.82 – / –
ESV02783 (0.009%) 0.1 0.90 – / –
ESV02826 (0.007%) 0.8 0.48 – / –
ESV02834 (0.008%) 0.2 0.79 – / –
ESV02889 (0.01%) 0.6 0.57 N / –
ESV02924 (0.006%) 0.2 0.80 – / –
ESV03083 (0.007%) 1.5 0.30 – / –
ESV03360 (0.006%) 0.6 0.58 – / –
ESV03515 (0.008%) 0.0 0.99 – / –
ESV03566 (0.005%) 1.7 0.26 N / –
ESV03659 (0.005%) 0.5 0.63 – / –
ESV03806 (0.007%) 1.8 0.24 – / –
ESV03826 (0.008%) 0.8 0.47 – / –
ESV03898 (0.01%) 2.4 0.17 – / –
ESV03948 (0.003%) 0.8 0.48 – / –
ESV03958 (0.004%) 2.5 0.17 – / –
ESV04290 (0.01%) 0.6 0.59 – / –
ESV04400 (0.007%) 0.4 0.70 – / –
ESV04448 (0.003%) 0.6 0.58 – / –
ESV04505 (0.004%) 1.1 0.40 – / –
ESV04519 (0.002%) 0.8 0.49 – / –
ESV04602 (0.007%) 0.5 0.61 – / –
ESV04632 (0.004%) 0.7 0.55 – / –
ESV04788 (0.004%) 0.6 0.60 – / –
ESV04833 (0.006%) 0.2 0.80 – / –
ESV04848 (0.003%) 0.7 0.52 – / –
ESV04912 (0.004%) 0.9 0.44 – / –
ESV04980 (0.004%) 0.9 0.47 – / –
ESV04998 (0.004%) 8.8 0.02 * – / –
ESV05100 (0.01%) 6.6 0.03 * – / –
ESV05103 (0.003%) 2.9 0.13 – / –
ESV05221 (0.002%) 0.2 0.85 – / –
ESV05278 (0.004%) 0.7 0.55 – / –
ESV05340 (0.004%) 1.1 0.38 – / –
ESV05434 (0.004%) 1.2 0.36 – / –
ESV05591 (0.003%) 1.0 0.41 – / –
ESV05604 (0.003%) 0.5 0.63 N / –
ESV05662 (0.003%) 0.0 1.00 N / –
ESV05677 (0.003%) 9.2 0.01 * – / –
ESV05733 (0.004%) 0.6 0.58 – / –
ESV05776 (0.008%) 1.3 0.33 – / –
ESV05996 (0.005%) 0.9 0.45 – / –
ESV06001 (0.002%) 1.6 0.28 – / –
ESV06010 (0.005%) 5.8 0.04 * – / –
ESV06030 (0.004%) 0.5 0.62 – / –
ESV06126 (0.004%) 0.2 0.85 – / –
ESV06164 (0.009%) 1.3 0.34 – / –
ESV06290 (0.004%) 0.9 0.46 – / –
ESV06348 (0.002%) 1.4 0.33 – / –
ESV06516 (0.002%) 3.7 0.09 . N / –
ESV06720 (0.004%) 0.0 0.96 N / –
ESV06744 (0.005%) 3.1 0.12 – / –
ESV06848 (0.005%) 10.0 0.01 * – / –
ESV06913 (0.004%) 0.3 0.76 – / –
ESV07112 (0.002%) 0.3 0.75 – / –
ESV07144 (0.002%) 2.1 0.21 – / –
ESV07221 (0.003%) 0.8 0.50 – / –
ESV07322 (0.003%) 0.0 0.97 – / –
ESV07354 (0.002%) 0.8 0.49 – / –
ESV07487 (0.005%) 5.2 0.05 * – / –

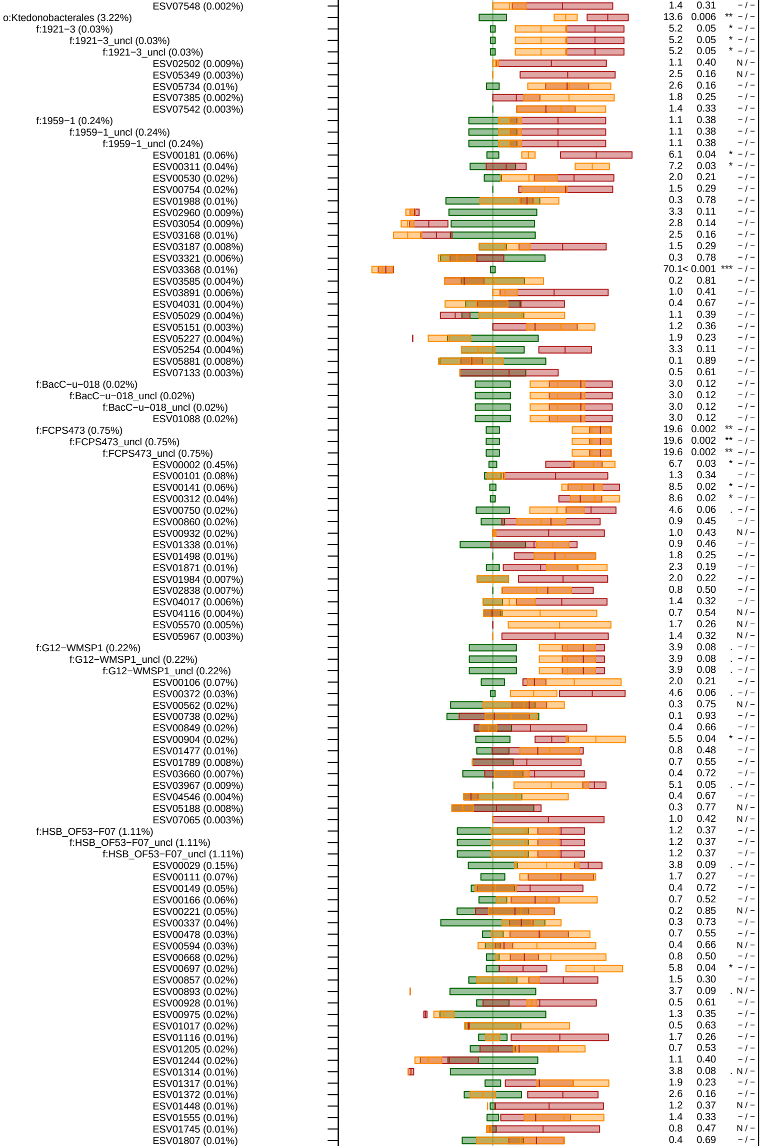
ESV07548 (0.002%) 1.4 0.31 −/−
o:Ktedonobacterales (3.22%) 13.6 0.006 ** −/−
f:1921−3 (0.03%) 5.2 0.05 * −/−
f:1921−3_uncl (0.03%) 5.2 0.05 * −/−
f:1921−3_uncl (0.03%) 5.2 0.05 * −/−
ESV02502 (0.009%) 1.1 0.40 N/−
ESV05349 (0.003%) 2.5 0.16 N/−
ESV05734 (0.01%) 2.6 0.16 −/−
ESV07385 (0.002%) 1.8 0.25 −/−
ESV07542 (0.003%) 1.4 0.33 −/−
f:1959−1 (0.24%) 1.1 0.38 −/−
f:1959−1_uncl (0.24%) 1.1 0.38 −/−
f:1959−1_uncl (0.24%) 1.1 0.38 −/−
ESV00181 (0.06%) 6.1 0.04 * −/−
ESV00311 (0.04%) 7.2 0.03 * −/−
ESV00530 (0.02%) 2.0 0.21 −/−
ESV00754 (0.02%) 1.5 0.29 −/−
ESV01988 (0.01%) 0.3 0.78 −/−
ESV02960 (0.009%) 3.3 0.11 −/−
ESV03054 (0.009%) 2.8 0.14 −/−
ESV03168 (0.01%) 2.5 0.16 −/−
ESV03187 (0.008%) 1.5 0.29 −/−
ESV03321 (0.006%) 0.3 0.78 −/−
ESV03368 (0.01%) 70.1 < 0.001 *** −/−
ESV03585 (0.004%) 0.2 0.81 −/−
ESV03891 (0.006%) 1.0 0.41 −/−
ESV04031 (0.004%) 0.4 0.67 −/−
ESV05029 (0.004%) 1.1 0.39 −/−
ESV05151 (0.003%) 1.2 0.36 −/−
ESV05227 (0.004%) 1.9 0.23 −/−
ESV05254 (0.004%) 3.3 0.11 −/−
ESV05881 (0.008%) 0.1 0.89 −/−
ESV07133 (0.003%) 0.5 0.61 −/−
f:BacC−u−018 (0.02%) 3.0 0.12 −/−
f:BacC−u−018_uncl (0.02%) 3.0 0.12 −/−
f:BacC−u−018_uncl (0.02%) 3.0 0.12 −/−
ESV01088 (0.02%) 3.0 0.12 −/−
f:FCPS473 (0.75%) 19.6 0.002 ** −/−
f:FCPS473_uncl (0.75%) 19.6 0.002 ** −/−
f:FCPS473_uncl (0.75%) 19.6 0.002 ** −/−
ESV00002 (0.45%) 6.7 0.03 * −/−
ESV00101 (0.08%) 1.3 0.34 −/−
ESV00141 (0.06%) 8.5 0.02 * −/−
ESV00312 (0.04%) 8.6 0.02 * −/−
ESV00750 (0.02%) 4.6 0.06 . −/−
ESV00860 (0.02%) 0.9 0.45 −/−
ESV00932 (0.02%) 1.0 0.43 N/−
ESV01338 (0.01%) 0.9 0.46 −/−
ESV01498 (0.01%) 1.8 0.25 −/−
ESV01871 (0.01%) 2.3 0.19 −/−
ESV01984 (0.007%) 2.0 0.22 −/−
ESV02838 (0.007%) 0.8 0.50 −/−
ESV04017 (0.006%) 1.4 0.32 −/−
ESV04116 (0.004%) 0.7 0.54 N/−
ESV05570 (0.005%) 1.7 0.26 N/−
ESV05967 (0.003%) 1.4 0.32 N/−
f:G12−WMSP1 (0.22%) 3.9 0.08 . −/−
f:G12−WMSP1_uncl (0.22%) 3.9 0.08 . −/−
f:G12−WMSP1_uncl (0.22%) 3.9 0.08 . −/−
ESV00106 (0.07%) 2.0 0.21 −/−
ESV00372 (0.03%) 4.6 0.06 . −/−
ESV00562 (0.02%) 0.3 0.75 N/−
ESV00738 (0.02%) 0.1 0.93 −/−
ESV00849 (0.02%) 0.4 0.66 −/−
ESV00904 (0.02%) 5.5 0.04 * −/−
ESV01477 (0.01%) 0.8 0.48 −/−
ESV01789 (0.008%) 0.7 0.55 −/−
ESV03660 (0.007%) 0.4 0.72 −/−
ESV03967 (0.009%) 5.1 0.05 . −/−
ESV04546 (0.004%) 0.4 0.67 −/−
ESV05188 (0.008%) 0.3 0.77 N/−
ESV07065 (0.003%) 1.0 0.42 N/−
f:HSB_OF53−F07 (1.11%) 1.2 0.37 −/−
f:HSB_OF53−F07_uncl (1.11%) 1.2 0.37 −/−
f:HSB_OF53−F07_uncl (1.11%) 1.2 0.37 −/−
ESV00029 (0.15%) 3.8 0.09 . −/−
ESV00111 (0.07%) 1.7 0.27 −/−
ESV00149 (0.05%) 0.4 0.72 −/−
ESV00166 (0.06%) 0.7 0.52 −/−
ESV00221 (0.05%) 0.2 0.85 N/−
ESV00337 (0.04%) 0.3 0.73 −/−
ESV00478 (0.03%) 0.7 0.55 −/−
ESV00594 (0.03%) 0.4 0.66 N/−
ESV00668 (0.02%) 0.8 0.50 −/−
ESV00697 (0.02%) 5.8 0.04 * −/−
ESV00857 (0.02%) 1.5 0.30 −/−
ESV00893 (0.02%) 3.7 0.09 . N/−
ESV00928 (0.01%) 0.5 0.61 −/−
ESV00975 (0.02%) 1.3 0.35 −/−
ESV01017 (0.02%) 0.5 0.63 −/−
ESV01116 (0.01%) 1.7 0.26 −/−
ESV01205 (0.02%) 0.7 0.53 −/−
ESV01244 (0.02%) 1.1 0.40 −/−
ESV01314 (0.01%) 3.8 0.08 . N/−
ESV01317 (0.01%) 1.9 0.23 −/−
ESV01372 (0.01%) 2.6 0.16 −/−
ESV01448 (0.01%) 1.2 0.37 N/−
ESV01555 (0.01%) 1.4 0.33 −/−
ESV01745 (0.01%) 0.8 0.47 N/−
ESV01807 (0.01%) 0.4 0.69 −/−

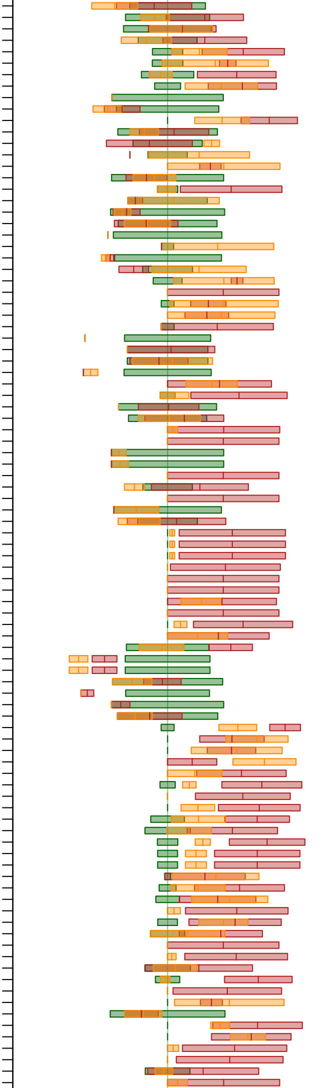

ESV01824 (0.02%)	0.7	0.55		- / -
ESV01826 (0.01%)	0.6	0.59		- / -
ESV02007 (0.01%)	0.0	0.95		- / -
ESV02116 (0.008%)	0.8	0.49		- / -
ESV02295 (0.01%)	1.6	0.28		- / -
ESV02296 (0.009%)	1.0	0.43		- / -
ESV02324 (0.01%)	2.2	0.19		- / -
ESV02359 (0.006%)	1.7	0.26		- / -
ESV02370 (0.008%)	1.0	0.42		N / -
ESV02385 (0.02%)	0.9	0.45		- / -
ESV02428 (0.005%)	5.0	0.05	.	N / -
ESV02534 (0.01%)	0.2	0.83		- / -
ESV02628 (0.01%)	1.0	0.42		- / -
ESV02669 (0.01%)	1.2	0.36		- / -
ESV02867 (0.007%)	0.8	0.50		N / -
ESV03138 (0.005%)	0.1	0.92		- / -
ESV03303 (0.004%)	1.5	0.30		- / -
ESV03489 (0.009%)	0.3	0.72		N / -
ESV03624 (0.02%)	0.5	0.61		- / -
ESV03644 (0.006%)	0.1	0.90		- / -
ESV03669 (0.008%)	1.2	0.36		N / -
ESV03698 (0.005%)	0.9	0.46		N / -
ESV03700 (0.008%)	1.2	0.36		N / -
ESV03987 (0.005%)	1.0	0.41		- / -
ESV04204 (0.005%)	1.5	0.30		- / -
ESV04461 (0.003%)	1.0	0.42		N / -
ESV04492 (0.009%)	0.8	0.50		- / -
ESV04542 (0.003%)	0.7	0.54		- / -
ESV04681 (0.004%)	0.9	0.46		N / -
ESV04853 (0.003%)	3.7	0.09	.	N / -
ESV05177 (0.003%)	0.5	0.63		N / -
ESV05303 (0.01%)	0.0	0.97		- / -
ESV05354 (0.005%)	3.3	0.11		- / -
ESV05477 (0.003%)	0.7	0.53		- / -
ESV06103 (0.006%)	1.8	0.24		- / -
ESV06134 (0.003%)	0.7	0.52		- / -
ESV06424 (0.008%)	0.1	0.94		- / -
ESV06577 (0.005%)	0.9	0.45		N / -
ESV06592 (0.003%)	1.0	0.42		N / -
ESV06597 (0.004%)	0.9	0.47		N / -
ESV06795 (0.003%)	0.9	0.47		N / -
ESV06804 (0.003%)	1.0	0.42		N / -
ESV06856 (0.006%)	1.0	0.41		- / -
ESV07416 (0.002%)	1.0	0.42		N / -
ESV07417 (0.003%)	0.6	0.56		- / -
ESV07577 (0.008%)	0.3	0.75		- / -
f:JG30a−KF−32 (0.05%)	1.4	0.32		N / -
f:JG30a−KF−32_uncl (0.05%)	1.4	0.32		N / -
f:JG30a−KF−32_uncl (0.05%)	1.4	0.32		N / -
ESV01018 (0.01%)	1.1	0.39		N / -
ESV01294 (0.01%)	1.0	0.42		N / -
ESV02853 (0.007%)	1.0	0.42		N / -
ESV03268 (0.004%)	0.7	0.55		- / -
ESV03707 (0.005%)	1.0	0.42		N / -
ESV04855 (0.006%)	2.0	0.22		N / -
ESV07423 (0.003%)	0.6	0.60		- / -
f:Ktedonobacteraceae (0.05%)	1.7	0.27		- / -
f:Ktedonobacteraceae_uncl (0.02%)	3.1	0.12		- / -
f:Ktedonobacteraceae_uncl (0.02%)	3.1	0.12		- / -
ESV02195 (0.008%)	0.3	0.76		- / -
ESV06789 (0.006%)	3.9	0.08	.	N / -
ESV06888 (0.003%)	0.8	0.48		N / -
ESV07356 (0.003%)	0.2	0.82		- / -
g:Ktedonobacter (0.03%)	16.5	0.004	**	- / -
g:Ktedonobacter_uncl (0.01%)	3.2	0.11		- / -
ESV02891 (0.006%)	1.7	0.26		- / -
ESV03394 (0.004%)	4.8	0.06	.	- / -
ESV07124 (0.002%)	1.5	0.29		- / -
s_Ktedonobacter_racemifer (0.02%)	4.3	0.07	.	- / -
ESV04202 (0.004%)	2.5	0.16		N / -
ESV04430 (0.005%)	3.4	0.11		- / -
ESV04841 (0.005%)	3.0	0.12		- / -
ESV06503 (0.003%)	1.1	0.40		- / -
f:Thermosporotrichaceae (0.29%)	4.8	0.06	.	- / -
f:Thermosporotrichaceae_uncl (0.2%)	3.1	0.12		- / -
f:Thermosporotrichaceae_uncl (0.2%)	3.1	0.12		- / -
ESV01036 (0.01%)	0.5	0.60		N / -
ESV02427 (0.008%)	1.4	0.32		- / -
ESV02498 (0.006%)	1.1	0.40		- / -
ESV02658 (0.005%)	1.6	0.27		- / -
ESV02731 (0.007%)	1.4	0.32		- / -
ESV02776 (0.006%)	0.6	0.56		- / -
ESV03186 (0.005%)	1.0	0.42		N / -
ESV03217 (0.007%)	1.7	0.27		N / -
ESV03274 (0.007%)	0.2	0.82		- / -
ESV03398 (0.02%)	6.4	0.03	*	- / -
ESV03570 (0.007%)	1.2	0.36		N / -
ESV03849 (0.009%)	1.0	0.43		- / -
ESV03906 (0.005%)	0.2	0.85		- / -
ESV03942 (0.006%)	2.9	0.13		- / -
ESV04032 (0.004%)	3.6	0.10	.	- / -
ESV04137 (0.006%)	1.5	0.29		N / -
ESV04194 (0.004%)	1.4	0.33		N / -
ESV04235 (0.02%)	0.4	0.70		- / -
ESV04354 (0.006%)	0.8	0.48		N / -

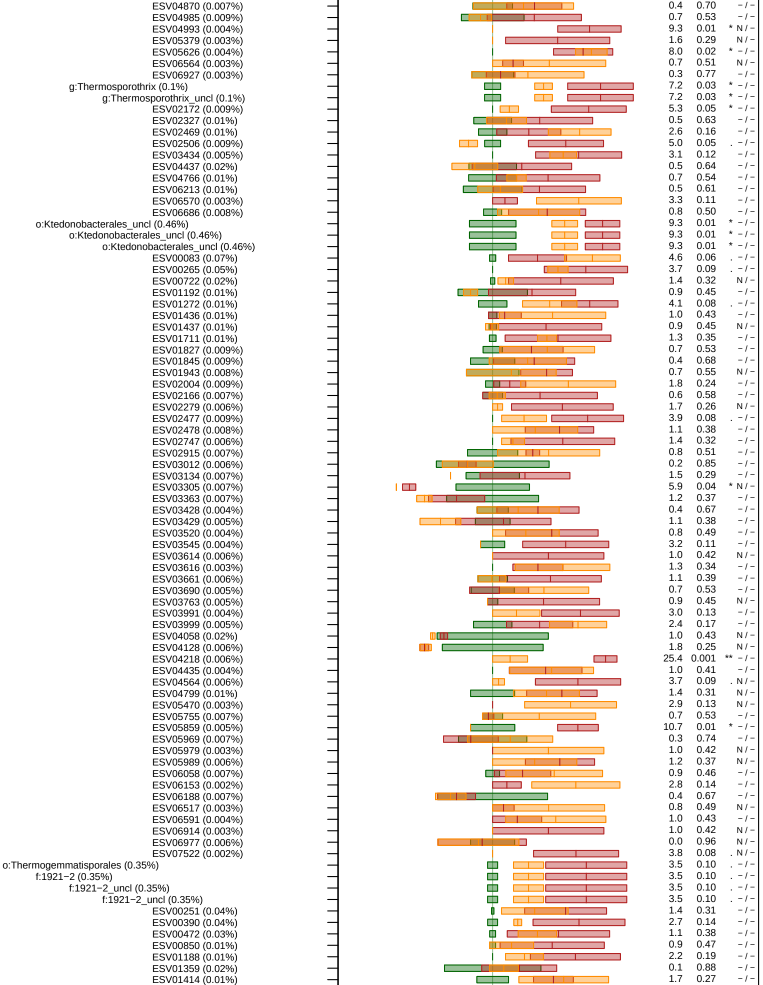
ESV04870 (0.007%) 0.4 0.70 – / –
ESV04985 (0.009%) 0.7 0.53 – / –
ESV04993 (0.004%) 9.3 0.01 * N / –
ESV05379 (0.003%) 1.6 0.29 N / –
ESV05626 (0.004%) 8.0 0.02 * – / –
ESV06564 (0.003%) 0.7 0.51 N / –
ESV06927 (0.003%) 0.3 0.77 – / –
g:Thermosporothrix (0.1%) 7.2 0.03 * – / –
g:Thermosporothrix_uncl (0.1%) 7.2 0.03 * – / –
ESV02172 (0.009%) 5.3 0.05 * – / –
ESV02327 (0.01%) 0.5 0.63 – / –
ESV02469 (0.01%) 2.6 0.16 – / –
ESV02506 (0.009%) 5.0 0.05 . – / –
ESV03434 (0.005%) 3.1 0.12 – / –
ESV04437 (0.02%) 0.5 0.64 – / –
ESV04766 (0.01%) 0.7 0.54 – / –
ESV06213 (0.01%) 0.5 0.61 – / –
ESV06570 (0.003%) 3.3 0.11 – / –
ESV06686 (0.008%) 0.8 0.50 – / –
o:Ktedonobacterales_uncl (0.46%) 9.3 0.01 * – / –
o:Ktedonobacterales_uncl (0.46%) 9.3 0.01 * – / –
o:Ktedonobacterales_uncl (0.46%) 9.3 0.01 * – / –
ESV00083 (0.07%) 4.6 0.06 . – / –
ESV00265 (0.05%) 3.7 0.09 . – / –
ESV00722 (0.02%) 1.4 0.32 N / –
ESV01192 (0.01%) 0.9 0.45 – / –
ESV01272 (0.01%) 4.1 0.08 . – / –
ESV01436 (0.01%) 1.0 0.43 – / –
ESV01437 (0.01%) 0.9 0.45 N / –
ESV01711 (0.01%) 1.3 0.35 – / –
ESV01827 (0.009%) 0.7 0.53 – / –
ESV01845 (0.009%) 0.4 0.68 – / –
ESV01943 (0.008%) 0.7 0.55 N / –
ESV02004 (0.009%) 1.8 0.24 – / –
ESV02166 (0.007%) 0.6 0.58 – / –
ESV02279 (0.006%) 1.7 0.26 N / –
ESV02477 (0.009%) 3.9 0.08 . – / –
ESV02478 (0.008%) 1.1 0.38 – / –
ESV02747 (0.006%) 1.4 0.32 – / –
ESV02915 (0.007%) 0.8 0.51 – / –
ESV03012 (0.006%) 0.2 0.85 – / –
ESV03134 (0.007%) 1.5 0.29 – / –
ESV03305 (0.007%) 5.9 0.04 * N / –
ESV03363 (0.007%) 1.2 0.37 – / –
ESV03428 (0.004%) 0.4 0.67 – / –
ESV03429 (0.005%) 1.1 0.38 – / –
ESV03520 (0.004%) 0.8 0.49 – / –
ESV03545 (0.004%) 3.2 0.11 – / –
ESV03614 (0.006%) 1.0 0.42 N / –
ESV03616 (0.003%) 1.3 0.34 – / –
ESV03661 (0.006%) 1.1 0.39 – / –
ESV03690 (0.005%) 0.7 0.53 – / –
ESV03763 (0.005%) 0.9 0.45 N / –
ESV03991 (0.004%) 3.0 0.13 – / –
ESV03999 (0.005%) 2.4 0.17 – / –
ESV04058 (0.02%) 1.0 0.43 N / –
ESV04128 (0.006%) 1.8 0.25 N / –
ESV04218 (0.006%) 25.4 0.001 ** – / –
ESV04435 (0.004%) 1.0 0.41 – / –
ESV04564 (0.006%) 3.7 0.09 . N / –
ESV04799 (0.01%) 1.4 0.31 N / –
ESV05470 (0.003%) 2.9 0.13 N / –
ESV05755 (0.007%) 0.7 0.53 – / –
ESV05859 (0.005%) 10.7 0.01 * – / –
ESV05969 (0.007%) 0.3 0.74 – / –
ESV05979 (0.003%) 1.0 0.42 N / –
ESV05989 (0.006%) 1.2 0.37 N / –
ESV06058 (0.007%) 0.9 0.46 – / –
ESV06153 (0.002%) 2.8 0.14 – / –
ESV06188 (0.007%) 0.4 0.67 – / –
ESV06517 (0.003%) 0.8 0.49 N / –
ESV06591 (0.004%) 1.0 0.43 – / –
ESV06914 (0.003%) 1.0 0.42 N / –
ESV06977 (0.006%) 0.0 0.96 N / –
ESV07522 (0.002%) 3.8 0.08 . N / –
o:Thermogemmatisporales (0.35%) 3.5 0.10 . – / –
f:1921−2 (0.35%) 3.5 0.10 . – / –
f:1921−2_uncl (0.35%) 3.5 0.10 . – / –
f:1921−2_uncl (0.35%) 3.5 0.10 . – / –
ESV00251 (0.04%) 1.4 0.31 – / –
ESV00390 (0.04%) 2.7 0.14 – / –
ESV00472 (0.03%) 1.1 0.38 – / –
ESV00850 (0.01%) 0.9 0.47 – / –
ESV01188 (0.01%) 2.2 0.19 – / –
ESV01359 (0.02%) 0.1 0.88 – / –
ESV01414 (0.01%) 1.7 0.27 – / –

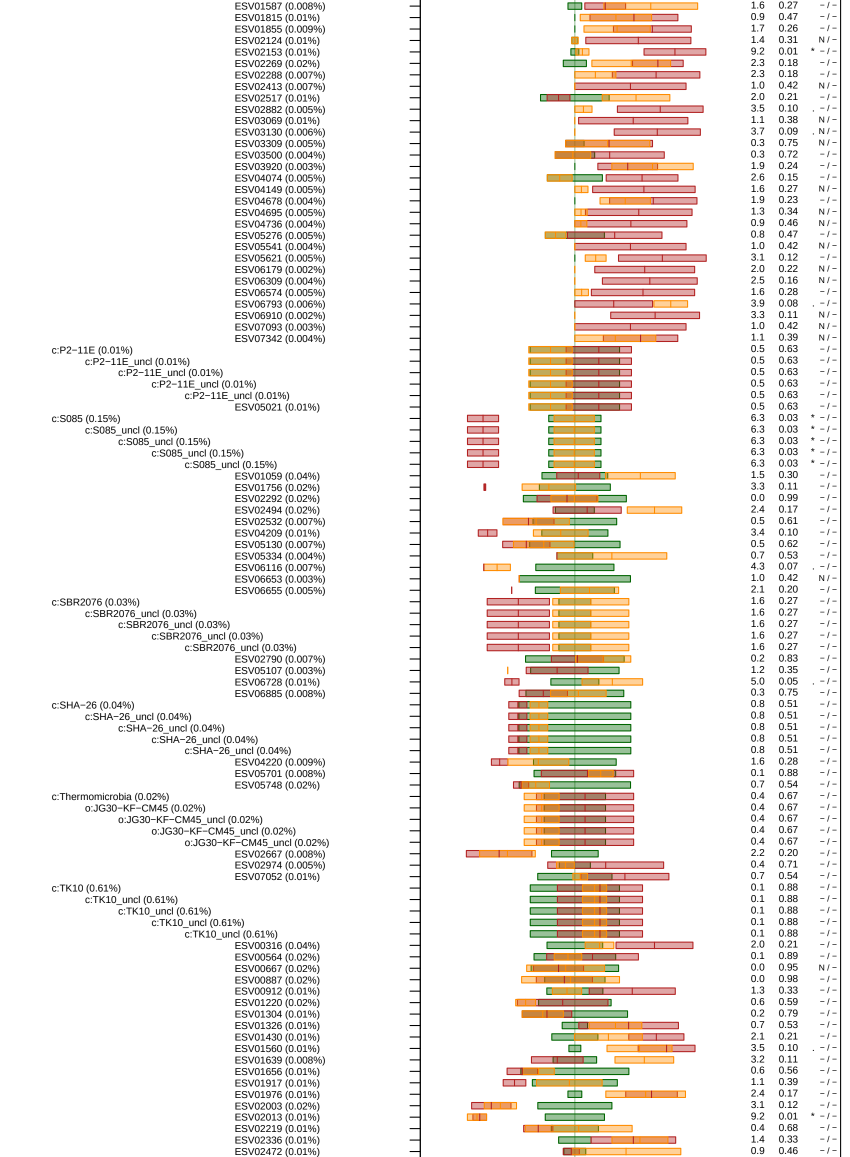
ESV01587 (0.008%) 1.6 0.27 - / -
ESV01815 (0.01%) 0.9 0.47 - / -
ESV01855 (0.009%) 1.7 0.26 - / -
ESV02124 (0.01%) 1.4 0.31 N / -
ESV02153 (0.01%) 9.2 0.01 * - / -
ESV02269 (0.02%) 2.3 0.18 - / -
ESV02288 (0.007%) 2.3 0.18 - / -
ESV02413 (0.007%) 1.0 0.42 N / -
ESV02517 (0.01%) 2.0 0.21 - / -
ESV02882 (0.005%) 3.5 0.10 . - / -
ESV03069 (0.01%) 1.1 0.38 N / -
ESV03130 (0.006%) 3.7 0.09 . N / -
ESV03309 (0.005%) 0.3 0.75 N / -
ESV03500 (0.004%) 0.3 0.72 - / -
ESV03920 (0.003%) 1.9 0.24 - / -
ESV04074 (0.005%) 2.6 0.15 - / -
ESV04149 (0.005%) 1.6 0.27 N / -
ESV04678 (0.004%) 1.9 0.23 - / -
ESV04695 (0.005%) 1.3 0.34 N / -
ESV04736 (0.004%) 0.9 0.46 N / -
ESV05276 (0.005%) 0.8 0.47 - / -
ESV05541 (0.004%) 1.0 0.42 N / -
ESV05621 (0.005%) 3.1 0.12 - / -
ESV06179 (0.002%) 2.0 0.22 N / -
ESV06309 (0.004%) 2.5 0.16 N / -
ESV06574 (0.005%) 1.6 0.28 - / -
ESV06793 (0.006%) 3.9 0.08 . - / -
ESV06910 (0.002%) 3.3 0.11 N / -
ESV07093 (0.003%) 1.0 0.42 N / -
ESV07342 (0.004%) 1.1 0.39 N / -
c:P2−11E (0.01%) 0.5 0.63 - / -
c:P2−11E_uncl (0.01%) 0.5 0.63 - / -
c:P2−11E_uncl (0.01%) 0.5 0.63 - / -
c:P2−11E_uncl (0.01%) 0.5 0.63 - / -
c:P2−11E_uncl (0.01%) 0.5 0.63 - / -
ESV05021 (0.01%) 0.5 0.63 - / -
c:S085 (0.15%) 6.3 0.03 * - / -
c:S085_uncl (0.15%) 6.3 0.03 * - / -
c:S085_uncl (0.15%) 6.3 0.03 * - / -
c:S085_uncl (0.15%) 6.3 0.03 * - / -
c:S085_uncl (0.15%) 6.3 0.03 * - / -
ESV01059 (0.04%) 1.5 0.30 - / -
ESV01756 (0.02%) 3.3 0.11 - / -
ESV02292 (0.02%) 0.0 0.99 - / -
ESV02494 (0.02%) 2.4 0.17 - / -
ESV02532 (0.007%) 0.5 0.61 - / -
ESV04209 (0.01%) 3.4 0.10 - / -
ESV05130 (0.007%) 0.5 0.62 - / -
ESV05334 (0.004%) 0.7 0.53 - / -
ESV06116 (0.007%) 4.3 0.07 . - / -
ESV06653 (0.003%) 1.0 0.42 N / -
ESV06655 (0.005%) 2.1 0.20 - / -
c:SBR2076 (0.03%) 1.6 0.27 - / -
c:SBR2076_uncl (0.03%) 1.6 0.27 - / -
c:SBR2076_uncl (0.03%) 1.6 0.27 - / -
c:SBR2076_uncl (0.03%) 1.6 0.27 - / -
c:SBR2076_uncl (0.03%) 1.6 0.27 - / -
ESV02790 (0.007%) 0.2 0.83 - / -
ESV05107 (0.003%) 1.2 0.35 - / -
ESV06728 (0.01%) 5.0 0.05 . - / -
ESV06885 (0.008%) 0.3 0.75 - / -
c:SHA−26 (0.04%) 0.8 0.51 - / -
c:SHA−26_uncl (0.04%) 0.8 0.51 - / -
c:SHA−26_uncl (0.04%) 0.8 0.51 - / -
c:SHA−26_uncl (0.04%) 0.8 0.51 - / -
c:SHA−26_uncl (0.04%) 0.8 0.51 - / -
ESV04220 (0.009%) 1.6 0.28 - / -
ESV05701 (0.008%) 0.1 0.88 - / -
ESV05748 (0.02%) 0.7 0.54 - / -
c:Thermomicrobia (0.02%) 0.4 0.67 - / -
o:JG30−KF−CM45 (0.02%) 0.4 0.67 - / -
o:JG30−KF−CM45_uncl (0.02%) 0.4 0.67 - / -
o:JG30−KF−CM45_uncl (0.02%) 0.4 0.67 - / -
o:JG30−KF−CM45_uncl (0.02%) 0.4 0.67 - / -
ESV02667 (0.008%) 2.2 0.20 - / -
ESV02974 (0.005%) 0.4 0.71 - / -
ESV07052 (0.01%) 0.7 0.54 - / -
c:TK10 (0.61%) 0.1 0.88 - / -
c:TK10_uncl (0.61%) 0.1 0.88 - / -
c:TK10_uncl (0.61%) 0.1 0.88 - / -
c:TK10_uncl (0.61%) 0.1 0.88 - / -
c:TK10_uncl (0.61%) 0.1 0.88 - / -
ESV00316 (0.04%) 2.0 0.21 - / -
ESV00564 (0.02%) 0.1 0.89 - / -
ESV00667 (0.02%) 0.0 0.95 N / -
ESV00887 (0.02%) 0.0 0.98 - / -
ESV00912 (0.01%) 1.3 0.33 - / -
ESV01220 (0.02%) 0.6 0.59 - / -
ESV01304 (0.01%) 0.2 0.79 - / -
ESV01326 (0.01%) 0.7 0.53 - / -
ESV01430 (0.01%) 2.1 0.21 - / -
ESV01560 (0.01%) 3.5 0.10 . - / -
ESV01639 (0.008%) 3.2 0.11 - / -
ESV01656 (0.01%) 0.6 0.56 - / -
ESV01917 (0.01%) 1.1 0.39 - / -
ESV01976 (0.01%) 2.4 0.17 - / -
ESV02003 (0.02%) 3.1 0.12 - / -
ESV02013 (0.01%) 9.2 0.01 * - / -
ESV02219 (0.01%) 0.4 0.68 - / -
ESV02336 (0.01%) 1.4 0.33 - / -
ESV02472 (0.01%) 0.9 0.46 - / -

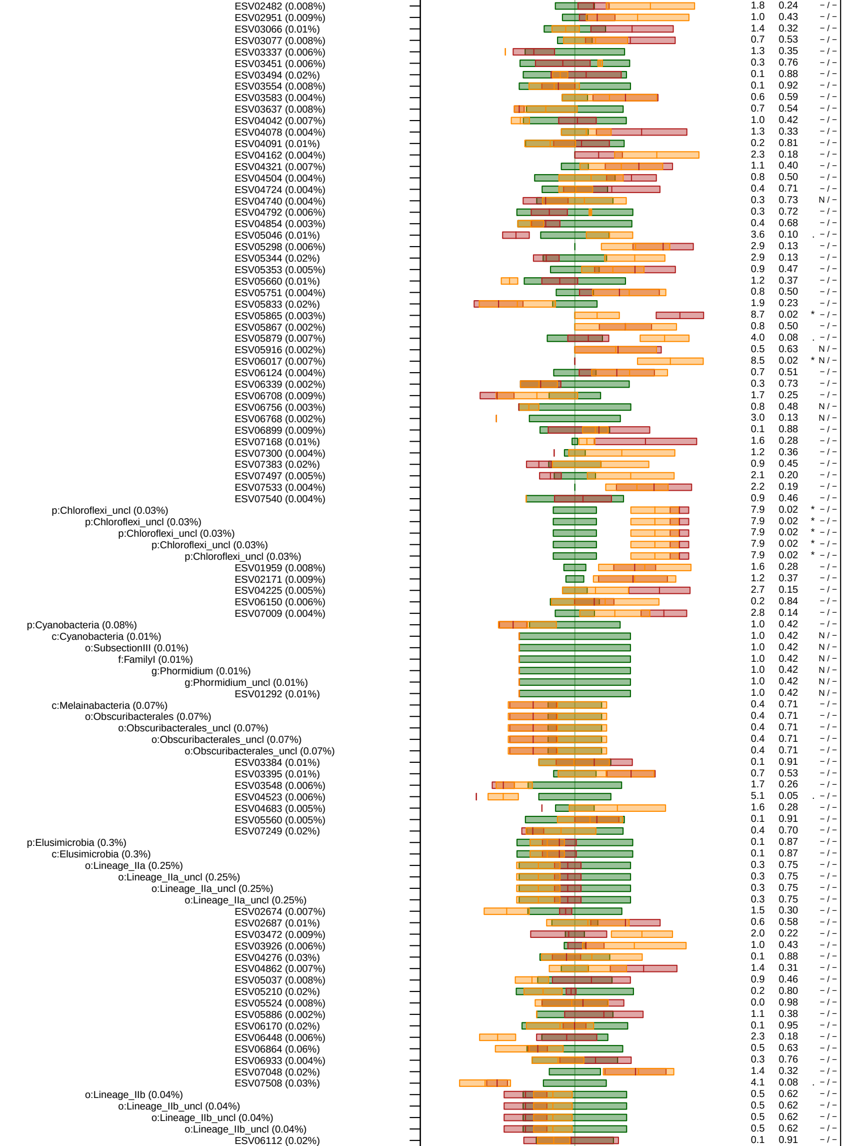
ESV02482 (0.008%) 1.8 0.24 - / -
ESV02951 (0.009%) 1.0 0.43 - / -
ESV03066 (0.01%) 1.4 0.32 - / -
ESV03077 (0.008%) 0.7 0.53 - / -
ESV03337 (0.006%) 1.3 0.35 - / -
ESV03451 (0.006%) 0.3 0.76 - / -
ESV03494 (0.02%) 0.1 0.88 - / -
ESV03554 (0.008%) 0.1 0.92 - / -
ESV03583 (0.004%) 0.6 0.59 - / -
ESV03637 (0.008%) 0.7 0.54 - / -
ESV04042 (0.007%) 1.0 0.42 - / -
ESV04078 (0.004%) 1.3 0.33 - / -
ESV04091 (0.01%) 0.2 0.81 - / -
ESV04162 (0.004%) 2.3 0.18 - / -
ESV04321 (0.007%) 1.1 0.40 - / -
ESV04504 (0.004%) 0.8 0.50 - / -
ESV04724 (0.004%) 0.4 0.71 - / -
ESV04740 (0.004%) 0.3 0.73 N / -
ESV04792 (0.006%) 0.3 0.72 - / -
ESV04854 (0.003%) 0.4 0.68 - / -
ESV05046 (0.01%) 3.6 0.10 . - / -
ESV05298 (0.006%) 2.9 0.13 - / -
ESV05344 (0.02%) 2.9 0.13 - / -
ESV05353 (0.005%) 0.9 0.47 - / -
ESV05660 (0.01%) 1.2 0.37 - / -
ESV05751 (0.004%) 0.8 0.50 - / -
ESV05833 (0.02%) 1.9 0.23 - / -
ESV05865 (0.003%) 8.7 0.02 * - / -
ESV05867 (0.002%) 0.8 0.50 - / -
ESV05879 (0.007%) 4.0 0.08 . - / -
ESV05916 (0.002%) 0.5 0.63 N / -
ESV06017 (0.007%) 8.5 0.02 * N / -
ESV06124 (0.004%) 0.7 0.51 - / -
ESV06339 (0.002%) 0.3 0.73 - / -
ESV06708 (0.009%) 1.7 0.25 - / -
ESV06756 (0.003%) 0.8 0.48 N / -
ESV06768 (0.002%) 3.0 0.13 N / -
ESV06899 (0.009%) 0.1 0.88 - / -
ESV07168 (0.01%) 1.6 0.28 - / -
ESV07300 (0.004%) 1.2 0.36 - / -
ESV07383 (0.02%) 0.9 0.45 - / -
ESV07497 (0.005%) 2.1 0.20 - / -
ESV07533 (0.004%) 2.2 0.19 - / -
ESV07540 (0.004%) 0.9 0.46 - / -
p:Chloroflexi_uncl (0.03%) 7.9 0.02 * - / -
p:Chloroflexi_uncl (0.03%) 7.9 0.02 * - / -
p:Chloroflexi_uncl (0.03%) 7.9 0.02 * - / -
p:Chloroflexi_uncl (0.03%) 7.9 0.02 * - / -
p:Chloroflexi_uncl (0.03%) 7.9 0.02 * - / -
ESV01959 (0.008%) 1.6 0.28 - / -
ESV02171 (0.009%) 1.2 0.37 - / -
ESV04225 (0.005%) 2.7 0.15 - / -
ESV06150 (0.006%) 0.2 0.84 - / -
ESV07009 (0.004%) 2.8 0.14 - / -
p:Cyanobacteria (0.08%) 1.0 0.42 - / -
c:Cyanobacteria (0.01%) 1.0 0.42 N / -
o:SubsectionIII (0.01%) 1.0 0.42 N / -
f:FamilyI (0.01%) 1.0 0.42 N / -
g:Phormidium (0.01%) 1.0 0.42 N / -
g:Phormidium_uncl (0.01%) 1.0 0.42 N / -
ESV01292 (0.01%) 1.0 0.42 N / -
c:Melainabacteria (0.07%) 0.4 0.71 - / -
o:Obscuribacterales (0.07%) 0.4 0.71 - / -
o:Obscuribacterales_uncl (0.07%) 0.4 0.71 - / -
o:Obscuribacterales_uncl (0.07%) 0.4 0.71 - / -
o:Obscuribacterales_uncl (0.07%) 0.4 0.71 - / -
ESV03384 (0.01%) 0.1 0.91 - / -
ESV03395 (0.01%) 0.7 0.53 - / -
ESV03548 (0.006%) 1.7 0.26 - / -
ESV04523 (0.006%) 5.1 0.05 . - / -
ESV04683 (0.005%) 1.6 0.28 - / -
ESV05560 (0.005%) 0.1 0.91 - / -
ESV07249 (0.02%) 0.4 0.70 - / -
p:Elusimicrobia (0.3%) 0.1 0.87 - / -
c:Elusimicrobia (0.3%) 0.1 0.87 - / -
o:Lineage_IIa (0.25%) 0.3 0.75 - / -
o:Lineage_IIa_uncl (0.25%) 0.3 0.75 - / -
o:Lineage_IIa_uncl (0.25%) 0.3 0.75 - / -
o:Lineage_IIa_uncl (0.25%) 0.3 0.75 - / -
ESV02674 (0.007%) 1.5 0.30 - / -
ESV02687 (0.01%) 0.6 0.58 - / -
ESV03472 (0.009%) 2.0 0.22 - / -
ESV03926 (0.006%) 1.0 0.43 - / -
ESV04276 (0.03%) 0.1 0.88 - / -
ESV04862 (0.007%) 1.4 0.31 - / -
ESV05037 (0.008%) 0.9 0.46 - / -
ESV05210 (0.02%) 0.2 0.80 - / -
ESV05524 (0.008%) 0.0 0.98 - / -
ESV05886 (0.002%) 1.1 0.38 - / -
ESV06170 (0.02%) 0.1 0.95 - / -
ESV06448 (0.006%) 2.3 0.18 - / -
ESV06864 (0.06%) 0.5 0.63 - / -
ESV06933 (0.004%) 0.3 0.76 - / -
ESV07048 (0.02%) 1.4 0.32 - / -
ESV07508 (0.03%) 4.1 0.08 . - / -
o:Lineage_IIb (0.04%) 0.5 0.62 - / -
o:Lineage_IIb_uncl (0.04%) 0.5 0.62 - / -
o:Lineage_IIb_uncl (0.04%) 0.5 0.62 - / -
o:Lineage_IIb_uncl (0.04%) 0.5 0.62 - / -
ESV06112 (0.02%) 0.1 0.91 - / -

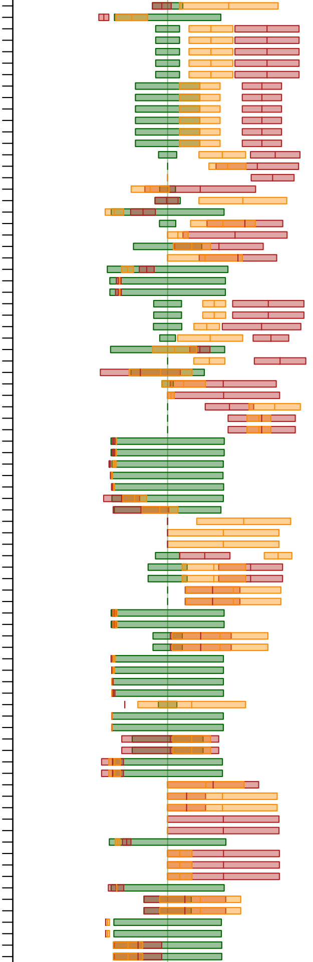

ESV07235 (0.005%)	1.5	0.30		- / -
ESV07344 (0.02%)	1.0	0.43		- / -
o:Lineage_IV (0.01%)	4.5	0.06	.	- / -
o:Lineage_IV_uncl (0.01%)	4.5	0.06	.	- / -
o:Lineage_IV_uncl (0.01%)	4.5	0.06	.	- / -
o:Lineage_IV_uncl (0.01%)	4.5	0.06	.	- / -
ESV04036 (0.01%)	4.5	0.06	.	- / -
p:FCPU426 (0.1%)	3.8	0.09	.	- / -
p:FCPU426_uncl (0.1%)	3.8	0.09	.	- / -
p:FCPU426_uncl (0.1%)	3.8	0.09	.	- / -
p:FCPU426_uncl (0.1%)	3.8	0.09	.	- / -
p:FCPU426_uncl (0.1%)	3.8	0.09	.	- / -
p:FCPU426_uncl (0.1%)	3.8	0.09	.	- / -
ESV03692 (0.009%)	7.0	0.03	*	- / -
ESV04270 (0.009%)	3.1	0.12		- / -
ESV04289 (0.003%)	24.0	0.001	**	N / -
ESV04828 (0.01%)	0.6	0.60		- / -
ESV04953 (0.004%)	2.6	0.16		- / -
ESV05822 (0.03%)	0.6	0.57		- / -
ESV06115 (0.01%)	1.9	0.23		- / -
ESV06435 (0.003%)	1.4	0.32		- / -
ESV07036 (0.01%)	0.6	0.59		- / -
ESV07178 (0.004%)	1.3	0.34		- / -
p:Firmicutes (1.54%)	0.3	0.73		- / -
c:Bacilli (0.91%)	0.7	0.53		N / -
o:Bacillales (0.9%)	0.7	0.53		N / -
f:Alicyclobacillaceae (0.09%)	4.8	0.06	.	- / -
g:Tumebacillus (0.09%)	4.8	0.06	.	- / -
g:Tumebacillus_uncl (0.08%)	3.5	0.10	.	- / -
ESV01248 (0.02%)	5.6	0.04	*	- / -
ESV01265 (0.02%)	0.2	0.81		- / -
ESV02121 (0.02%)	10.7	0.01	*	- / -
ESV02700 (0.01%)	0.2	0.86		- / -
ESV03832 (0.007%)	0.7	0.52		- / -
ESV05292 (0.007%)	0.9	0.44		N / -
ESV05934 (0.008%)	6.9	0.03	*	- / -
s_Tumebacillus_permanentifrigoris (0.007%)	6.8	0.03	*	- / -
ESV05070 (0.007%)	6.8	0.03	*	- / -
f:Bacillaceae (0.74%)	0.9	0.47		N / -
g:Bacillus (0.73%)	0.9	0.46		N / -
g:Bacillus_uncl (0.25%)	1.0	0.42		N / -
ESV00033 (0.18%)	1.0	0.41		N / -
ESV00483 (0.03%)	1.0	0.44		N / -
ESV03609 (0.01%)	0.5	0.65		- / -
ESV04732 (0.005%)	0.1	0.87		- / -
ESV05230 (0.003%)	2.6	0.15		N / -
ESV05924 (0.002%)	1.0	0.42		N / -
ESV06525 (0.003%)	1.0	0.42		N / -
ESV07329 (0.01%)	9.7	0.01	*	- / -
s_Bacillus_anthracis (0.03%)	2.2	0.20		- / -
ESV00473 (0.03%)	2.2	0.20		- / -
s_Bacillus_aryabhattai (0.01%)	1.1	0.38		- / -
ESV02487 (0.01%)	1.1	0.38		- / -
s_Bacillus_drentensis (0.29%)	0.9	0.46		N / -
ESV00021 (0.29%)	0.9	0.46		N / -
s_Bacillus_litoralis (0.004%)	0.6	0.57		- / -
ESV04050 (0.004%)	0.6	0.57		- / -
s_Bacillus_luciferensis (0.12%)	1.0	0.43		N / -
ESV00249 (0.04%)	1.0	0.43		N / -
ESV00346 (0.04%)	1.0	0.43		N / -
ESV01521 (0.02%)	1.0	0.43		N / -
ESV02293 (0.005%)	1.2	0.38		- / -
ESV03155 (0.007%)	1.0	0.42		N / -
ESV07183 (0.003%)	1.0	0.42		N / -
s_Bacillus_niacini (0.02%)	0.1	0.88		- / -
ESV00972 (0.02%)	0.1	0.88		- / -
s_Bacillus_simplex (0.01%)	0.9	0.45		- / -
ESV01507 (0.01%)	0.9	0.45		- / -
g:Fictibacillus (0.006%)	0.5	0.63		N / -
g:Fictibacillus_uncl (0.003%)	0.7	0.54		- / -
ESV07006 (0.003%)	0.7	0.54		- / -
s_Fictibacillus_arsenicus (0.002%)	1.0	0.42		N / -
ESV07370 (0.002%)	1.0	0.42		N / -
f:Paenibacillaceae (0.04%)	0.6	0.57		- / -
g:Brevibacillus (0.005%)	0.8	0.50		- / -
g:Brevibacillus_uncl (0.005%)	0.8	0.50		- / -
ESV06834 (0.005%)	0.8	0.50		- / -
g:Paenibacillus (0.03%)	0.8	0.49		N / -
g:Paenibacillus_uncl (0.005%)	0.2	0.82		N / -
ESV07399 (0.005%)	0.2	0.82		N / -
s_Paenibacillus_alginolyticus (0.02%)	1.3	0.34		N / -
ESV01621 (0.02%)	1.3	0.34		N / -
s_Paenibacillus_taihuensis (0.01%)	0.3	0.72		- / -
ESV07057 (0.01%)	0.3	0.72		- / -

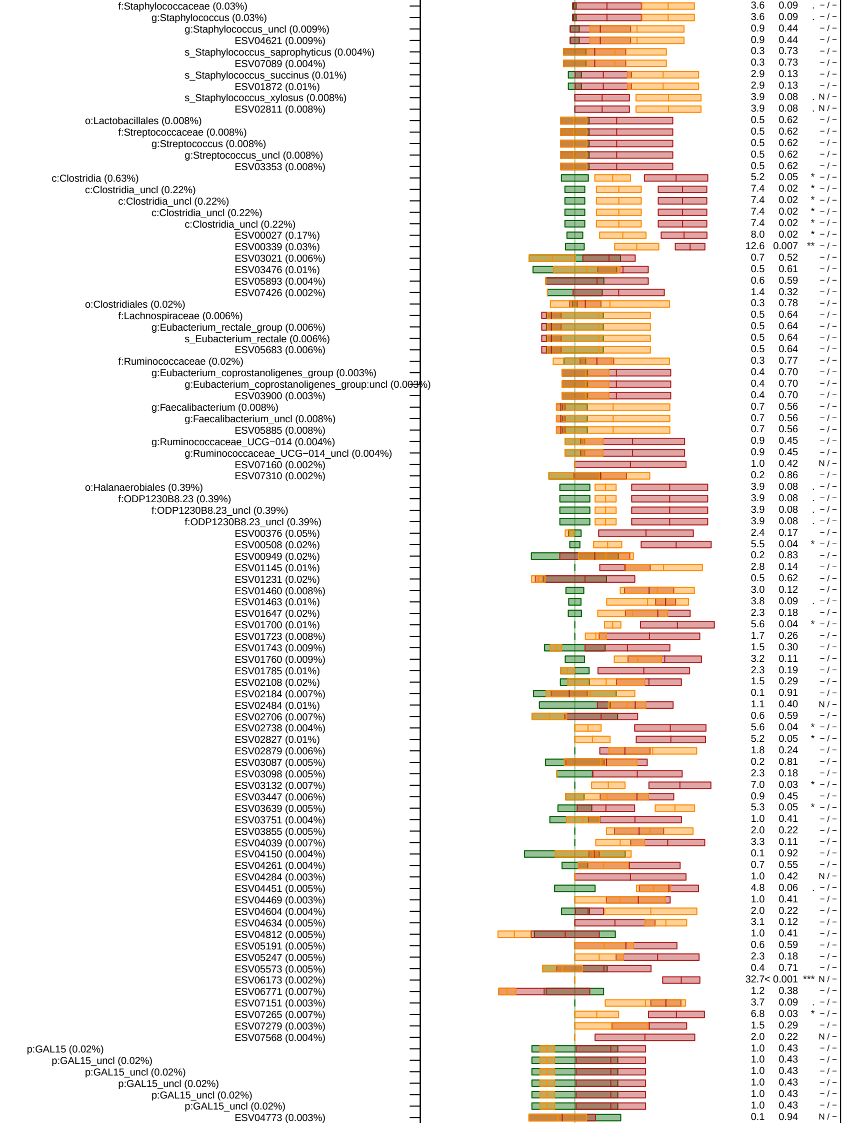

f:Staphylococcaceae (0.03%) 3.6 0.09 . – / –
g:Staphylococcus (0.03%) 3.6 0.09 . – / –
g:Staphylococcus_uncl (0.009%) 0.9 0.44 – / –
ESV04621 (0.009%) 0.9 0.44 – / –
s_Staphylococcus_saprophyticus (0.004%) 0.3 0.73 – / –
ESV07089 (0.004%) 0.3 0.73 – / –
s_Staphylococcus_succinus (0.01%) 2.9 0.13 – / –
ESV01872 (0.01%) 2.9 0.13 – / –
s_Staphylococcus_xylosus (0.008%) 3.9 0.08 . N / –
ESV02811 (0.008%) 3.9 0.08 . N / –
o:Lactobacillales (0.008%) 0.5 0.62 – / –
f:Streptococcaceae (0.008%) 0.5 0.62 – / –
g:Streptococcus (0.008%) 0.5 0.62 – / –
g:Streptococcus_uncl (0.008%) 0.5 0.62 – / –
ESV03353 (0.008%) 0.5 0.62 – / –
c:Clostridia (0.63%) 5.2 0.05 * – / –
c:Clostridia_uncl (0.22%) 7.4 0.02 * – / –
c:Clostridia_uncl (0.22%) 7.4 0.02 * – / –
c:Clostridia_uncl (0.22%) 7.4 0.02 * – / –
c:Clostridia_uncl (0.22%) 7.4 0.02 * – / –
ESV00027 (0.17%) 8.0 0.02 * – / –
ESV00339 (0.03%) 12.6 0.007 ** – / –
ESV03021 (0.006%) 0.7 0.52 – / –
ESV03476 (0.01%) 0.5 0.61 – / –
ESV05893 (0.004%) 0.6 0.59 – / –
ESV07426 (0.002%) 1.4 0.32 – / –
o:Clostridiales (0.02%) 0.3 0.78 – / –
f:Lachnospiraceae (0.006%) 0.5 0.64 – / –
g:Eubacterium_rectale_group (0.006%) 0.5 0.64 – / –
s_Eubacterium_rectale (0.006%) 0.5 0.64 – / –
ESV05683 (0.006%) 0.5 0.64 – / –
f:Ruminococcaceae (0.02%) 0.3 0.77 – / –
g:Eubacterium_coprostanoligenes_group (0.003%) 0.4 0.70 – / –
g:Eubacterium_coprostanoligenes_group:uncl (0.003%) 0.4 0.70 – / –
ESV03900 (0.003%) 0.4 0.70 – / –
g:Faecalibacterium (0.008%) 0.7 0.56 – / –
g:Faecalibacterium_uncl (0.008%) 0.7 0.56 – / –
ESV05885 (0.008%) 0.7 0.56 – / –
g:Ruminococcaceae_UCG–014 (0.004%) 0.9 0.45 – / –
g:Ruminococcaceae_UCG–014_uncl (0.004%) 0.9 0.45 – / –
ESV07160 (0.002%) 1.0 0.42 N / –
ESV07310 (0.002%) 0.2 0.86 – / –
o:Halanaerobiales (0.39%) 3.9 0.08 . – / –
f:ODP1230B8.23 (0.39%) 3.9 0.08 . – / –
f:ODP1230B8.23_uncl (0.39%) 3.9 0.08 . – / –
f:ODP1230B8.23_uncl (0.39%) 3.9 0.08 . – / –
ESV00376 (0.05%) 2.4 0.17 – / –
ESV00508 (0.02%) 5.5 0.04 * – / –
ESV00949 (0.02%) 0.2 0.83 – / –
ESV01145 (0.01%) 2.8 0.14 – / –
ESV01231 (0.02%) 0.5 0.62 – / –
ESV01460 (0.008%) 3.0 0.12 – / –
ESV01463 (0.01%) 3.8 0.09 . – / –
ESV01647 (0.02%) 2.3 0.18 – / –
ESV01700 (0.01%) 5.6 0.04 * – / –
ESV01723 (0.008%) 1.7 0.26 – / –
ESV01743 (0.009%) 1.5 0.30 – / –
ESV01760 (0.009%) 3.2 0.11 – / –
ESV01785 (0.01%) 2.3 0.19 – / –
ESV02108 (0.02%) 1.5 0.29 – / –
ESV02184 (0.007%) 0.1 0.91 – / –
ESV02484 (0.01%) 1.1 0.40 N / –
ESV02706 (0.007%) 0.6 0.59 – / –
ESV02738 (0.004%) 5.6 0.04 * – / –
ESV02827 (0.01%) 5.2 0.05 * – / –
ESV02879 (0.006%) 1.8 0.24 – / –
ESV03087 (0.005%) 0.2 0.81 – / –
ESV03098 (0.005%) 2.3 0.18 – / –
ESV03132 (0.007%) 7.0 0.03 * – / –
ESV03447 (0.006%) 0.9 0.45 – / –
ESV03639 (0.005%) 5.3 0.05 * – / –
ESV03751 (0.004%) 1.0 0.41 – / –
ESV03855 (0.005%) 2.0 0.22 – / –
ESV04039 (0.007%) 3.3 0.11 – / –
ESV04150 (0.004%) 0.1 0.92 – / –
ESV04261 (0.004%) 0.7 0.55 – / –
ESV04284 (0.003%) 1.0 0.42 N / –
ESV04451 (0.005%) 4.8 0.06 . – / –
ESV04469 (0.003%) 1.0 0.41 – / –
ESV04604 (0.004%) 2.0 0.22 – / –
ESV04634 (0.005%) 3.1 0.12 – / –
ESV04812 (0.005%) 1.0 0.41 – / –
ESV05191 (0.005%) 0.6 0.59 – / –
ESV05247 (0.005%) 2.3 0.18 – / –
ESV05573 (0.005%) 0.4 0.71 – / –
ESV06173 (0.002%) 32.7 < 0.001 *** N / –
ESV06771 (0.007%) 1.2 0.38 – / –
ESV07151 (0.003%) 3.7 0.09 . – / –
ESV07265 (0.007%) 6.8 0.03 * – / –
ESV07279 (0.003%) 1.5 0.29 – / –
ESV07568 (0.004%) 2.0 0.22 N / –
p:GAL15 (0.02%) 1.0 0.43 – / –
p:GAL15_uncl (0.02%) 1.0 0.43 – / –
p:GAL15_uncl (0.02%) 1.0 0.43 – / –
p:GAL15_uncl (0.02%) 1.0 0.43 – / –
p:GAL15_uncl (0.02%) 1.0 0.43 – / –
p:GAL15_uncl (0.02%) 1.0 0.43 – / –
ESV04773 (0.003%) 0.1 0.94 N / –

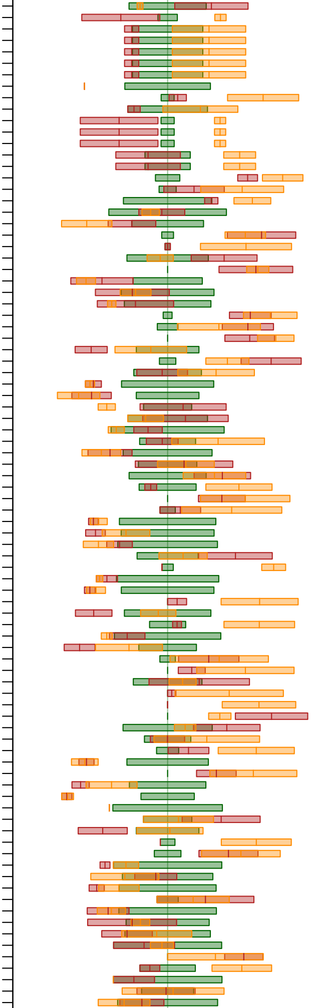

ESV06388 (0.02%)	1.4	0.32		− / −
p:Gemmatimonadetes (2.34%)	4.5	0.06	.	− / −
c:BD2−11_terrestrial_group (0.02%)	1.7	0.26		− / −
c:BD2−11_terrestrial_group:uncl (0.02%)	1.7	0.26		− / −
c:BD2−11_terrestrial_group:uncl (0.02%)	1.7	0.26		− / −
c:BD2−11_terrestrial_group:uncl (0.02%)	1.7	0.26		− / −
c:BD2−11_terrestrial_group:uncl (0.02%)	1.7	0.26		− / −
ESV05499 (0.003%)	3.8	0.09	.	N / −
ESV05562 (0.009%)	6.0	0.04	*	− / −
ESV06989 (0.007%)	1.1	0.39		− / −
c:Gemmatimonadetes (2.28%)	4.8	0.06	.	− / −
o:Gemmatimonadales (2.28%)	4.8	0.06	.	− / −
f:Gemmatimonadaceae (2.28%)	4.8	0.06	.	− / −
f:Gemmatimonadaceae_uncl (1.63%)	3.9	0.08	.	− / −
f:Gemmatimonadaceae_uncl (1.63%)	3.9	0.08	.	− / −
ESV00056 (0.12%)	15.9	0.004	**	− / −
ESV00173 (0.05%)	1.6	0.28		− / −
ESV00182 (0.05%)	2.3	0.18		− / −
ESV00188 (0.06%)	0.1	0.95		− / −
ESV00227 (0.05%)	2.0	0.22		− / −
ESV00260 (0.04%)	4.7	0.06	.	− / −
ESV00293 (0.05%)	2.9	0.13		N / −
ESV00301 (0.03%)	1.3	0.35		− / −
ESV00366 (0.04%)	5.4	0.05	*	− / −
ESV00386 (0.03%)	2.5	0.16		− / −
ESV00397 (0.04%)	0.3	0.75		− / −
ESV00450 (0.03%)	0.7	0.53		− / −
ESV00569 (0.02%)	7.6	0.02	*	− / −
ESV00659 (0.02%)	2.0	0.22		− / −
ESV00747 (0.02%)	10.1	0.01	*	− / −
ESV00779 (0.03%)	1.9	0.23		− / −
ESV00784 (0.02%)	5.6	0.04	*	− / −
ESV00888 (0.01%)	0.8	0.49		− / −
ESV00985 (0.02%)	2.6	0.15		− / −
ESV01002 (0.02%)	3.8	0.09	.	− / −
ESV01076 (0.02%)	2.0	0.22		− / −
ESV01117 (0.009%)	0.3	0.74		− / −
ESV01150 (0.02%)	0.6	0.59		− / −
ESV01152 (0.01%)	0.9	0.46		− / −
ESV01158 (0.01%)	1.4	0.32		− / −
ESV01284 (0.01%)	0.1	0.93		− / −
ESV01332 (0.01%)	0.8	0.49		− / −
ESV01376 (0.009%)	3.4	0.10		− / −
ESV01476 (0.009%)	1.9	0.23		− / −
ESV01636 (0.01%)	1.2	0.38		− / −
ESV01659 (0.009%)	2.1	0.20		− / −
ESV01775 (0.02%)	1.4	0.32		− / −
ESV01828 (0.01%)	1.3	0.34		− / −
ESV01905 (0.008%)	1.3	0.33		− / −
ESV01907 (0.01%)	68.0	< 0.001	***	− / −
ESV01911 (0.01%)	1.5	0.29		− / −
ESV01924 (0.009%)	2.5	0.16		− / −
ESV01931 (0.01%)	4.9	0.06	.	− / −
ESV02097 (0.02%)	1.9	0.22		− / −
ESV02102 (0.01%)	4.8	0.06	.	− / −
ESV02176 (0.007%)	0.9	0.45		− / −
ESV02189 (0.01%)	2.7	0.15		− / −
ESV02211 (0.007%)	0.7	0.55		− / −
ESV02227 (0.006%)	1.9	0.23		− / −
ESV02309 (0.01%)	0.2	0.83		− / −
ESV02369 (0.006%)	1.2	0.36		N / −
ESV02564 (0.01%)	6.2	0.03	*	N / −
ESV02653 (0.007%)	5.7	0.04	*	− / −
ESV02746 (0.01%)	0.9	0.47		− / −
ESV02754 (0.01%)	0.4	0.67		− / −
ESV02978 (0.01%)	3.2	0.11		− / −
ESV03005 (0.008%)	3.6	0.10	.	− / −
ESV03027 (0.006%)	2.4	0.17		− / −
ESV03068 (0.006%)	2.7	0.15		− / −
ESV03076 (0.009%)	13.1	0.007	**	− / −
ESV03120 (0.01%)	1.1	0.38		N / −
ESV03191 (0.005%)	0.7	0.52		− / −
ESV03215 (0.009%)	1.6	0.27		− / −
ESV03251 (0.006%)	6.7	0.03	*	− / −
ESV03362 (0.01%)	1.8	0.25		− / −
ESV03381 (0.01%)	1.0	0.43		− / −
ESV03413 (0.005%)	0.4	0.69		− / −
ESV03435 (0.007%)	1.4	0.32		− / −
ESV03467 (0.004%)	0.3	0.75		− / −
ESV03480 (0.01%)	1.1	0.39		− / −
ESV03483 (0.01%)	0.3	0.77		− / −
ESV03568 (0.008%)	0.4	0.71		− / −
ESV03580 (0.005%)	0.1	0.89		− / −
ESV03600 (0.004%)	1.7	0.26		− / −
ESV03611 (0.007%)	4.1	0.08	.	− / −
ESV03620 (0.004%)	0.7	0.55		− / −
ESV03630 (0.006%)	0.0	0.99		− / −
ESV03719 (0.007%)	0.4	0.69		− / −

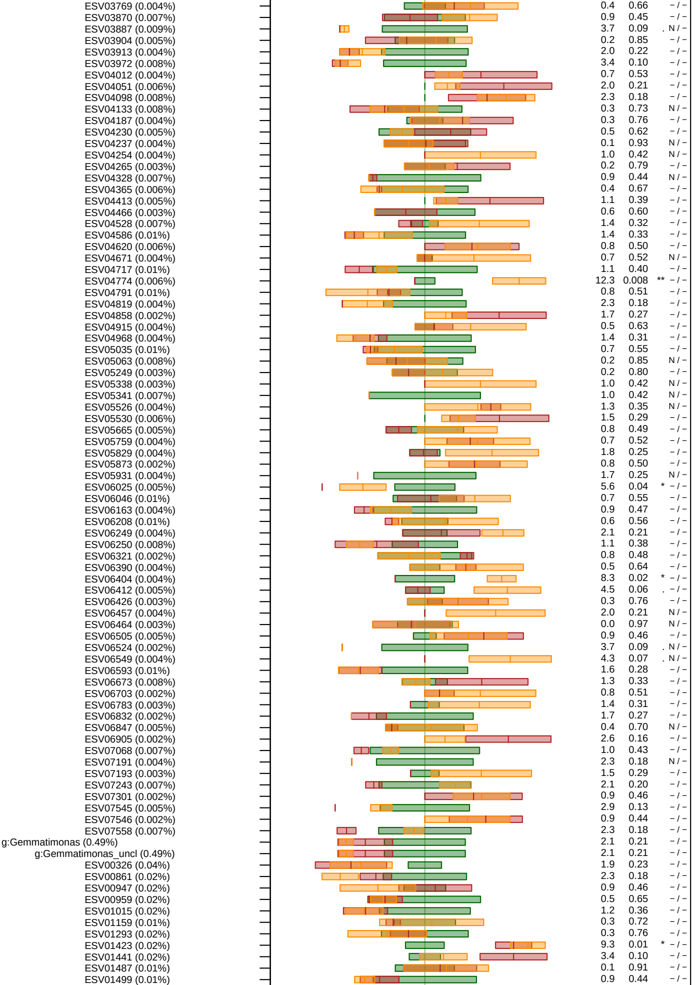
ESV03769 (0.004%) 0.4 0.66 – / –
ESV03870 (0.007%) 0.9 0.45 – / –
ESV03887 (0.009%) 3.7 0.09 . N / –
ESV03904 (0.005%) 0.2 0.85 – / –
ESV03913 (0.004%) 2.0 0.22 – / –
ESV03972 (0.008%) 3.4 0.10 – / –
ESV04012 (0.004%) 0.7 0.53 – / –
ESV04051 (0.006%) 2.0 0.21 – / –
ESV04098 (0.008%) 2.3 0.18 – / –
ESV04133 (0.008%) 0.3 0.73 N / –
ESV04187 (0.004%) 0.3 0.76 – / –
ESV04230 (0.005%) 0.5 0.62 – / –
ESV04237 (0.004%) 0.1 0.93 N / –
ESV04254 (0.004%) 1.0 0.42 N / –
ESV04265 (0.003%) 0.2 0.79 – / –
ESV04328 (0.007%) 0.9 0.44 N / –
ESV04365 (0.006%) 0.4 0.67 – / –
ESV04413 (0.005%) 1.1 0.39 – / –
ESV04466 (0.003%) 0.6 0.60 – / –
ESV04528 (0.007%) 1.4 0.32 – / –
ESV04586 (0.01%) 1.4 0.33 – / –
ESV04620 (0.006%) 0.8 0.50 – / –
ESV04671 (0.004%) 0.7 0.52 N / –
ESV04717 (0.01%) 1.1 0.40 – / –
ESV04774 (0.006%) 12.3 0.008 ** – / –
ESV04791 (0.01%) 0.8 0.51 – / –
ESV04819 (0.004%) 2.3 0.18 – / –
ESV04858 (0.002%) 1.7 0.27 – / –
ESV04915 (0.004%) 0.5 0.63 – / –
ESV04968 (0.004%) 1.4 0.31 – / –
ESV05035 (0.01%) 0.7 0.55 – / –
ESV05063 (0.008%) 0.2 0.85 N / –
ESV05249 (0.003%) 0.2 0.80 – / –
ESV05338 (0.003%) 1.0 0.42 N / –
ESV05341 (0.007%) 1.0 0.42 N / –
ESV05526 (0.004%) 1.3 0.35 N / –
ESV05530 (0.006%) 1.5 0.29 – / –
ESV05665 (0.005%) 0.8 0.49 – / –
ESV05759 (0.004%) 0.7 0.52 – / –
ESV05829 (0.004%) 1.8 0.25 – / –
ESV05873 (0.002%) 0.8 0.50 – / –
ESV05931 (0.004%) 1.7 0.25 N / –
ESV06025 (0.005%) 5.6 0.04 * – / –
ESV06046 (0.01%) 0.7 0.55 – / –
ESV06163 (0.004%) 0.9 0.47 – / –
ESV06208 (0.01%) 0.6 0.56 – / –
ESV06249 (0.004%) 2.1 0.21 – / –
ESV06250 (0.008%) 1.1 0.38 – / –
ESV06321 (0.002%) 0.8 0.48 – / –
ESV06390 (0.004%) 0.5 0.64 – / –
ESV06404 (0.004%) 8.3 0.02 * – / –
ESV06412 (0.005%) 4.5 0.06 . – / –
ESV06426 (0.003%) 0.3 0.76 – / –
ESV06457 (0.004%) 2.0 0.21 N / –
ESV06464 (0.003%) 0.0 0.97 N / –
ESV06505 (0.005%) 0.9 0.46 – / –
ESV06524 (0.002%) 3.7 0.09 . N / –
ESV06549 (0.004%) 4.3 0.07 . N / –
ESV06593 (0.01%) 1.6 0.28 – / –
ESV06673 (0.008%) 1.3 0.33 – / –
ESV06703 (0.002%) 0.8 0.51 – / –
ESV06783 (0.003%) 1.4 0.31 – / –
ESV06832 (0.002%) 1.7 0.27 – / –
ESV06847 (0.005%) 0.4 0.70 N / –
ESV06905 (0.002%) 2.6 0.16 – / –
ESV07068 (0.007%) 1.0 0.43 – / –
ESV07191 (0.004%) 2.3 0.18 N / –
ESV07193 (0.003%) 1.5 0.29 – / –
ESV07243 (0.007%) 2.1 0.20 – / –
ESV07301 (0.002%) 0.9 0.46 – / –
ESV07545 (0.005%) 2.9 0.13 – / –
ESV07546 (0.002%) 0.9 0.44 – / –
ESV07558 (0.007%) 2.3 0.18 – / –
g:Gemmatimonas (0.49%) 2.1 0.21 – / –
g:Gemmatimonas_uncl (0.49%) 2.1 0.21 – / –
ESV00326 (0.04%) 1.9 0.23 – / –
ESV00861 (0.02%) 2.3 0.18 – / –
ESV00947 (0.02%) 0.9 0.46 – / –
ESV00959 (0.02%) 0.5 0.65 – / –
ESV01015 (0.02%) 1.2 0.36 – / –
ESV01159 (0.01%) 0.3 0.72 – / –
ESV01293 (0.02%) 0.3 0.76 – / –
ESV01423 (0.02%) 9.3 0.01 * – / –
ESV01441 (0.02%) 3.4 0.10 – / –
ESV01487 (0.01%) 0.1 0.91 – / –
ESV01499 (0.01%) 0.9 0.44 – / –

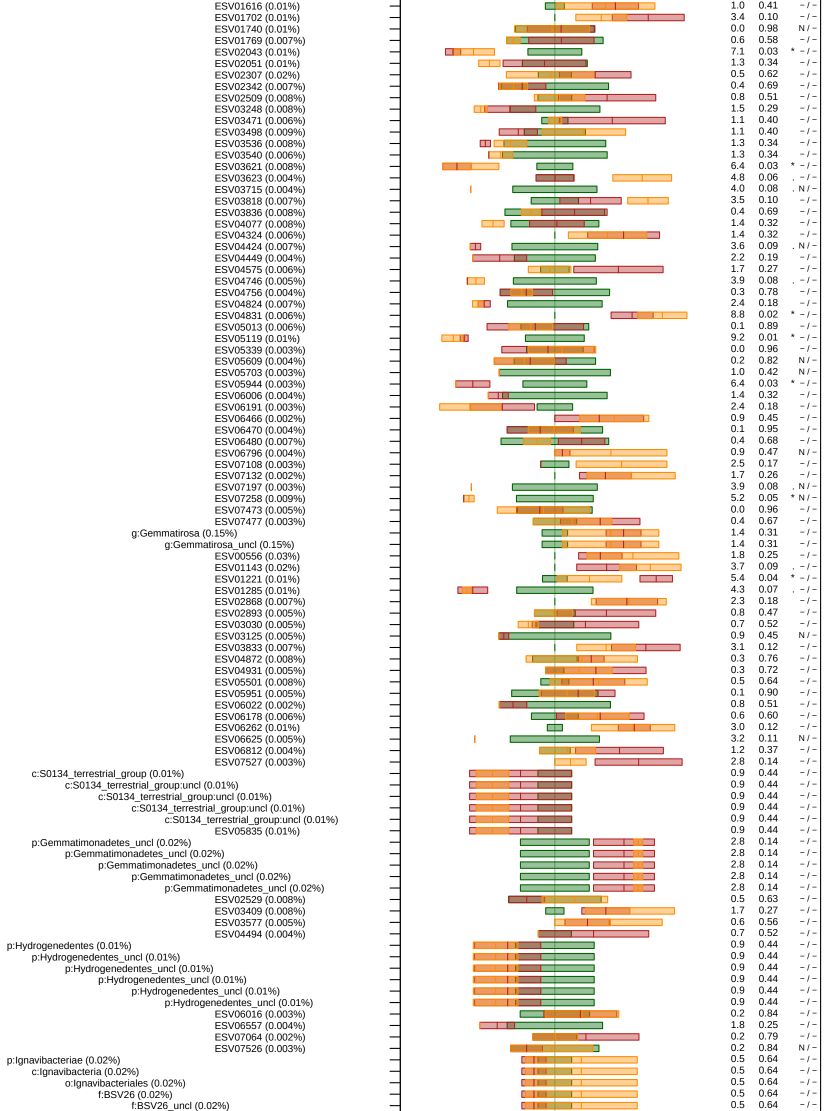
ESV01616 (0.01%) 1.0 0.41 - / -
ESV01702 (0.01%) 3.4 0.10 - / -
ESV01740 (0.01%) 0.0 0.98 N / -
ESV01769 (0.007%) 0.6 0.58 - / -
ESV02043 (0.01%) 7.1 0.03 * - / -
ESV02051 (0.01%) 1.3 0.34 - / -
ESV02307 (0.02%) 0.5 0.62 - / -
ESV02342 (0.007%) 0.4 0.69 - / -
ESV02509 (0.008%) 0.8 0.51 - / -
ESV03248 (0.008%) 1.5 0.29 - / -
ESV03471 (0.006%) 1.1 0.40 - / -
ESV03498 (0.009%) 1.1 0.40 - / -
ESV03536 (0.008%) 1.3 0.34 - / -
ESV03540 (0.006%) 1.3 0.34 - / -
ESV03621 (0.008%) 6.4 0.03 * - / -
ESV03623 (0.004%) 4.8 0.06 . - / -
ESV03715 (0.004%) 4.0 0.08 . N / -
ESV03818 (0.007%) 3.5 0.10 - / -
ESV03836 (0.008%) 0.4 0.69 - / -
ESV04077 (0.008%) 1.4 0.32 - / -
ESV04324 (0.006%) 1.4 0.32 - / -
ESV04424 (0.007%) 3.6 0.09 . N / -
ESV04449 (0.004%) 2.2 0.19 - / -
ESV04575 (0.006%) 1.7 0.27 - / -
ESV04746 (0.005%) 3.9 0.08 . - / -
ESV04756 (0.004%) 0.3 0.78 - / -
ESV04824 (0.007%) 2.4 0.18 - / -
ESV04831 (0.006%) 8.8 0.02 * - / -
ESV05013 (0.006%) 0.1 0.89 - / -
ESV05119 (0.01%) 9.2 0.01 * - / -
ESV05339 (0.003%) 0.0 0.96 - / -
ESV05609 (0.004%) 0.2 0.82 N / -
ESV05703 (0.003%) 1.0 0.42 N / -
ESV05944 (0.003%) 6.4 0.03 * - / -
ESV06006 (0.004%) 1.4 0.32 - / -
ESV06191 (0.003%) 2.4 0.18 - / -
ESV06466 (0.002%) 0.9 0.45 - / -
ESV06470 (0.004%) 0.1 0.95 - / -
ESV06480 (0.007%) 0.4 0.68 - / -
ESV06796 (0.004%) 0.9 0.47 N / -
ESV07108 (0.003%) 2.5 0.17 - / -
ESV07132 (0.002%) 1.7 0.26 - / -
ESV07197 (0.003%) 3.9 0.08 . N / -
ESV07258 (0.009%) 5.2 0.05 * N / -
ESV07473 (0.005%) 0.0 0.96 - / -
ESV07477 (0.003%) 0.4 0.67 - / -
g:Gemmatirosa (0.15%) 1.4 0.31 - / -
g:Gemmatirosa_uncl (0.15%) 1.4 0.31 - / -
ESV00556 (0.03%) 1.8 0.25 - / -
ESV01143 (0.02%) 3.7 0.09 . - / -
ESV01221 (0.01%) 5.4 0.04 * - / -
ESV01285 (0.01%) 4.3 0.07 . - / -
ESV02868 (0.007%) 2.3 0.18 - / -
ESV02893 (0.005%) 0.8 0.47 - / -
ESV03030 (0.005%) 0.7 0.52 - / -
ESV03125 (0.005%) 0.9 0.45 N / -
ESV03833 (0.007%) 3.1 0.12 - / -
ESV04872 (0.008%) 0.3 0.76 - / -
ESV04931 (0.005%) 0.3 0.72 - / -
ESV05501 (0.008%) 0.5 0.64 - / -
ESV05951 (0.005%) 0.1 0.90 - / -
ESV06022 (0.002%) 0.8 0.51 - / -
ESV06178 (0.006%) 0.6 0.60 - / -
ESV06262 (0.01%) 3.0 0.12 - / -
ESV06625 (0.005%) 3.2 0.11 N / -
ESV06812 (0.004%) 1.2 0.37 - / -
ESV07527 (0.003%) 2.8 0.14 - / -
c:S0134_terrestrial_group (0.01%) 0.9 0.44 - / -
c:S0134_terrestrial_group:uncl (0.01%) 0.9 0.44 - / -
c:S0134_terrestrial_group:uncl (0.01%) 0.9 0.44 - / -
c:S0134_terrestrial_group:uncl (0.01%) 0.9 0.44 - / -
c:S0134_terrestrial_group:uncl (0.01%) 0.9 0.44 - / -
ESV05835 (0.01%) 0.9 0.44 - / -
p:Gemmatimonadetes_uncl (0.02%) 2.8 0.14 - / -
p:Gemmatimonadetes_uncl (0.02%) 2.8 0.14 - / -
p:Gemmatimonadetes_uncl (0.02%) 2.8 0.14 - / -
p:Gemmatimonadetes_uncl (0.02%) 2.8 0.14 - / -
p:Gemmatimonadetes_uncl (0.02%) 2.8 0.14 - / -
ESV02529 (0.008%) 0.5 0.63 - / -
ESV03409 (0.008%) 1.7 0.27 - / -
ESV03577 (0.005%) 0.6 0.56 - / -
ESV04494 (0.004%) 0.7 0.52 - / -
p:Hydrogenedentes (0.01%) 0.9 0.44 - / -
p:Hydrogenedentes_uncl (0.01%) 0.9 0.44 - / -
p:Hydrogenedentes_uncl (0.01%) 0.9 0.44 - / -
p:Hydrogenedentes_uncl (0.01%) 0.9 0.44 - / -
p:Hydrogenedentes_uncl (0.01%) 0.9 0.44 - / -
p:Hydrogenedentes_uncl (0.01%) 0.9 0.44 - / -
ESV06016 (0.003%) 0.2 0.84 - / -
ESV06557 (0.004%) 1.8 0.25 - / -
ESV07064 (0.002%) 0.2 0.79 - / -
ESV07526 (0.003%) 0.2 0.84 N / -
p:Ignavibacteriae (0.02%) 0.5 0.64 - / -
c:Ignavibacteria (0.02%) 0.5 0.64 - / -
o:Ignavibacteriales (0.02%) 0.5 0.64 - / -
f:BSV26 (0.02%) 0.5 0.64 - / -
f:BSV26_uncl (0.02%) 0.5 0.64 - / -

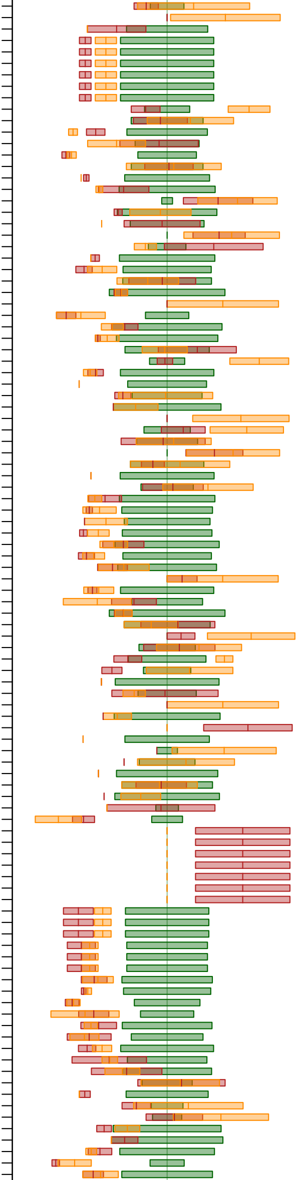

f:BSV26_uncl (0.02%)	0.5	0.64	– / –
ESV02163 (0.01%)	1.1	0.38	N / –
ESV05777 (0.008%)	2.0	0.22	– / –
p:Latescibacteria (0.59%)	2.3	0.18	– / –
p:Latescibacteria_uncl (0.59%)	2.3	0.18	– / –
p:Latescibacteria_uncl (0.59%)	2.3	0.18	– / –
p:Latescibacteria_uncl (0.59%)	2.3	0.18	– / –
p:Latescibacteria_uncl (0.59%)	2.3	0.18	– / –
p:Latescibacteria_uncl (0.59%)	2.3	0.18	– / –
ESV00899 (0.03%)	7.7	0.02	* – / –
ESV00918 (0.02%)	0.2	0.83	– / –
ESV01283 (0.01%)	4.2	0.07	. – / –
ESV01632 (0.02%)	0.7	0.55	– / –
ESV02041 (0.01%)	10.9	0.01	* – / –
ESV02236 (0.01%)	0.0	0.99	– / –
ESV02697 (0.02%)	3.9	0.08	. N / –
ESV02749 (0.02%)	1.2	0.37	– / –
ESV03090 (0.01%)	1.4	0.31	– / –
ESV03117 (0.01%)	0.6	0.57	– / –
ESV03407 (0.01%)	1.4	0.31	– / –
ESV03543 (0.005%)	1.2	0.37	– / –
ESV03601 (0.005%)	1.3	0.35	– / –
ESV03667 (0.007%)	2.4	0.17	N / –
ESV03680 (0.03%)	2.6	0.16	– / –
ESV03694 (0.01%)	0.1	0.93	N / –
ESV03716 (0.008%)	0.6	0.57	– / –
ESV03834 (0.005%)	1.0	0.42	N / –
ESV03871 (0.005%)	7.7	0.02	* – / –
ESV03966 (0.008%)	0.7	0.51	– / –
ESV03969 (0.01%)	1.6	0.28	– / –
ESV03997 (0.005%)	0.3	0.78	– / –
ESV04090 (0.008%)	7.0	0.03	* – / –
ESV04143 (0.01%)	2.4	0.17	– / –
ESV04192 (0.01%)	5.0	0.05	. N / –
ESV04306 (0.007%)	0.5	0.61	– / –
ESV04348 (0.005%)	0.6	0.56	– / –
ESV04414 (0.005%)	2.3	0.18	N / –
ESV04495 (0.005%)	2.3	0.19	– / –
ESV04512 (0.006%)	0.0	0.98	N / –
ESV04570 (0.004%)	1.2	0.37	– / –
ESV04572 (0.004%)	0.1	0.87	– / –
ESV04605 (0.005%)	2.6	0.15	N / –
ESV04786 (0.009%)	0.4	0.69	– / –
ESV04965 (0.02%)	1.8	0.25	– / –
ESV05018 (0.01%)	2.3	0.18	– / –
ESV05183 (0.004%)	2.4	0.17	– / –
ESV05481 (0.01%)	2.8	0.14	– / –
ESV05496 (0.005%)	0.7	0.52	– / –
ESV05527 (0.02%)	2.8	0.14	– / –
ESV05556 (0.02%)	0.8	0.51	– / –
ESV05940 (0.002%)	0.7	0.51	– / –
ESV06011 (0.01%)	2.1	0.20	– / –
ESV06122 (0.005%)	1.3	0.35	N / –
ESV06288 (0.008%)	0.5	0.61	– / –
ESV06320 (0.004%)	0.1	0.87	N / –
ESV06407 (0.003%)	2.9	0.13	– / –
ESV06455 (0.004%)	0.2	0.82	– / –
ESV06672 (0.01%)	4.0	0.08	. – / –
ESV06693 (0.01%)	1.9	0.23	– / –
ESV06764 (0.003%)	1.6	0.28	N / –
ESV06809 (0.005%)	0.3	0.77	– / –
ESV06850 (0.003%)	1.0	0.42	N / –
ESV06883 (0.005%)	1.1	0.38	– / –
ESV06929 (0.002%)	3.3	0.11	N / –
ESV06966 (0.004%)	4.0	0.08	. N / –
ESV07044 (0.004%)	1.4	0.32	– / –
ESV07062 (0.005%)	1.0	0.43	– / –
ESV07111 (0.005%)	1.8	0.24	N / –
ESV07181 (0.006%)	0.0	0.99	N / –
ESV07352 (0.006%)	1.0	0.43	– / –
ESV07512 (0.004%)	1.1	0.40	N / –
ESV07576 (0.02%)	10.9	0.01	* – / –
p:Microgenomates (0.001%)	2.6	0.16	N / –
c:Candidatus_Levybacteria (0.001%)	2.6	0.16	N / –
c:Candidatus_Levybacteria_uncl (0.001%)	2.6	0.16	N / –
c:Candidatus_Levybacteria_uncl (0.001%)	2.6	0.16	N / –
c:Candidatus_Levybacteria_uncl (0.001%)	2.6	0.16	N / –
c:Candidatus_Levybacteria_uncl (0.001%)	2.6	0.16	N / –
ESV07205 (0.001%)	2.6	0.16	N / –
p:Nitrospirae (1.95%)	3.1	0.12	– / –
c:Nitrospira (1.95%)	3.1	0.12	– / –
o:Nitrospirales (1.95%)	3.1	0.12	– / –
f:0319−6A21 (0.83%)	3.5	0.10	. – / –
f:0319−6A21_uncl (0.83%)	3.5	0.10	. – / –
f:0319−6A21_uncl (0.83%)	3.5	0.10	. – / –
ESV00126 (0.09%)	2.1	0.21	– / –
ESV00218 (0.06%)	3.5	0.10	N / –
ESV00241 (0.05%)	7.6	0.02	* – / –
ESV00275 (0.04%)	2.9	0.13	– / –
ESV00416 (0.05%)	2.2	0.19	– / –
ESV00598 (0.03%)	3.3	0.11	– / –
ESV00675 (0.02%)	2.3	0.18	– / –
ESV00758 (0.02%)	1.1	0.39	– / –
ESV01016 (0.01%)	0.5	0.62	– / –
ESV01204 (0.01%)	0.0	0.95	– / –
ESV01401 (0.01%)	4.2	0.07	. – / –
ESV01619 (0.009%)	0.6	0.58	– / –
ESV01626 (0.02%)	0.8	0.50	– / –
ESV01688 (0.01%)	1.0	0.43	– / –
ESV01751 (0.008%)	0.8	0.50	– / –
ESV01772 (0.02%)	2.1	0.20	– / –
ESV01981 (0.01%)	18.3	0.003	** – / –
ESV02029 (0.02%)	2.0	0.21	– / –

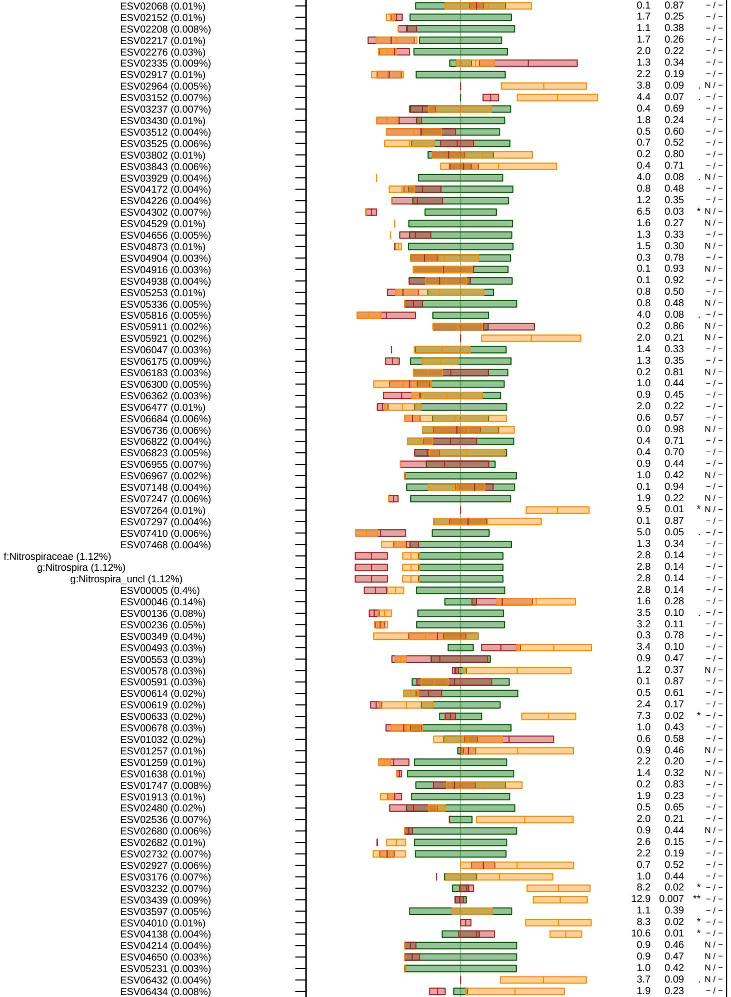

ESV02068 (0.01%) 0.1 0.87 - / -
ESV02152 (0.01%) 1.7 0.25 - / -
ESV02208 (0.008%) 1.1 0.38 - / -
ESV02217 (0.01%) 1.7 0.26 - / -
ESV02276 (0.03%) 2.0 0.22 - / -
ESV02335 (0.009%) 1.3 0.34 - / -
ESV02917 (0.01%) 2.2 0.19 - / -
ESV02964 (0.005%) 3.8 0.09 . N / -
ESV03152 (0.007%) 4.4 0.07 . - / -
ESV03237 (0.007%) 0.4 0.69 - / -
ESV03430 (0.01%) 1.8 0.24 - / -
ESV03512 (0.004%) 0.5 0.60 - / -
ESV03525 (0.006%) 0.7 0.52 - / -
ESV03802 (0.01%) 0.2 0.80 - / -
ESV03843 (0.006%) 0.4 0.71 - / -
ESV03929 (0.004%) 4.0 0.08 . N / -
ESV04172 (0.004%) 0.8 0.48 - / -
ESV04226 (0.004%) 1.2 0.35 - / -
ESV04302 (0.007%) 6.5 0.03 * N / -
ESV04529 (0.01%) 1.6 0.27 N / -
ESV04656 (0.005%) 1.3 0.33 - / -
ESV04873 (0.01%) 1.5 0.30 N / -
ESV04904 (0.003%) 0.3 0.78 - / -
ESV04916 (0.003%) 0.1 0.93 N / -
ESV04938 (0.004%) 0.1 0.92 - / -
ESV05253 (0.01%) 0.8 0.50 - / -
ESV05336 (0.005%) 0.8 0.48 N / -
ESV05816 (0.005%) 4.0 0.08 . - / -
ESV05911 (0.002%) 0.2 0.86 N / -
ESV05921 (0.002%) 2.0 0.21 N / -
ESV06047 (0.003%) 1.4 0.33 - / -
ESV06175 (0.009%) 1.3 0.35 - / -
ESV06183 (0.003%) 0.2 0.81 N / -
ESV06300 (0.005%) 1.0 0.44 - / -
ESV06362 (0.003%) 0.9 0.45 - / -
ESV06477 (0.01%) 2.0 0.22 - / -
ESV06684 (0.006%) 0.6 0.57 - / -
ESV06736 (0.006%) 0.0 0.98 N / -
ESV06822 (0.004%) 0.4 0.71 - / -
ESV06823 (0.005%) 0.4 0.70 - / -
ESV06955 (0.007%) 0.9 0.44 - / -
ESV06967 (0.002%) 1.0 0.42 N / -
ESV07148 (0.004%) 0.1 0.94 - / -
ESV07247 (0.006%) 1.9 0.22 N / -
ESV07264 (0.01%) 9.5 0.01 * N / -
ESV07297 (0.004%) 0.1 0.87 - / -
ESV07410 (0.006%) 5.0 0.05 . - / -
ESV07468 (0.004%) 1.3 0.34 - / -
f:Nitrospiraceae (1.12%) 2.8 0.14 - / -
g:Nitrospira (1.12%) 2.8 0.14 - / -
g:Nitrospira_uncl (1.12%) 2.8 0.14 - / -
ESV00005 (0.4%) 2.8 0.14 - / -
ESV00046 (0.14%) 1.6 0.28 - / -
ESV00136 (0.08%) 3.5 0.10 . - / -
ESV00236 (0.05%) 3.2 0.11 - / -
ESV00349 (0.04%) 0.3 0.78 - / -
ESV00493 (0.03%) 3.4 0.10 - / -
ESV00553 (0.03%) 0.9 0.47 - / -
ESV00578 (0.03%) 1.2 0.37 N / -
ESV00591 (0.03%) 0.1 0.87 - / -
ESV00614 (0.02%) 0.5 0.61 - / -
ESV00619 (0.02%) 2.4 0.17 - / -
ESV00633 (0.02%) 7.3 0.02 * - / -
ESV00678 (0.03%) 1.0 0.43 - / -
ESV01032 (0.02%) 0.6 0.58 - / -
ESV01257 (0.01%) 0.9 0.46 N / -
ESV01259 (0.01%) 2.2 0.20 - / -
ESV01638 (0.01%) 1.4 0.32 N / -
ESV01747 (0.008%) 0.2 0.83 - / -
ESV01913 (0.01%) 1.9 0.23 - / -
ESV02480 (0.02%) 0.5 0.65 - / -
ESV02536 (0.007%) 2.0 0.21 - / -
ESV02680 (0.006%) 0.9 0.44 N / -
ESV02682 (0.01%) 2.6 0.15 - / -
ESV02732 (0.007%) 2.2 0.19 - / -
ESV02927 (0.006%) 0.7 0.52 - / -
ESV03176 (0.007%) 1.0 0.44 - / -
ESV03232 (0.007%) 8.2 0.02 * - / -
ESV03439 (0.009%) 12.9 0.007 ** - / -
ESV03597 (0.005%) 1.1 0.39 - / -
ESV04010 (0.01%) 8.3 0.02 * - / -
ESV04138 (0.004%) 10.6 0.01 * - / -
ESV04214 (0.004%) 0.9 0.46 N / -
ESV04650 (0.003%) 0.9 0.47 N / -
ESV05231 (0.003%) 1.0 0.42 N / -
ESV06432 (0.004%) 3.7 0.09 . N / -
ESV06434 (0.008%) 1.9 0.23 - / -

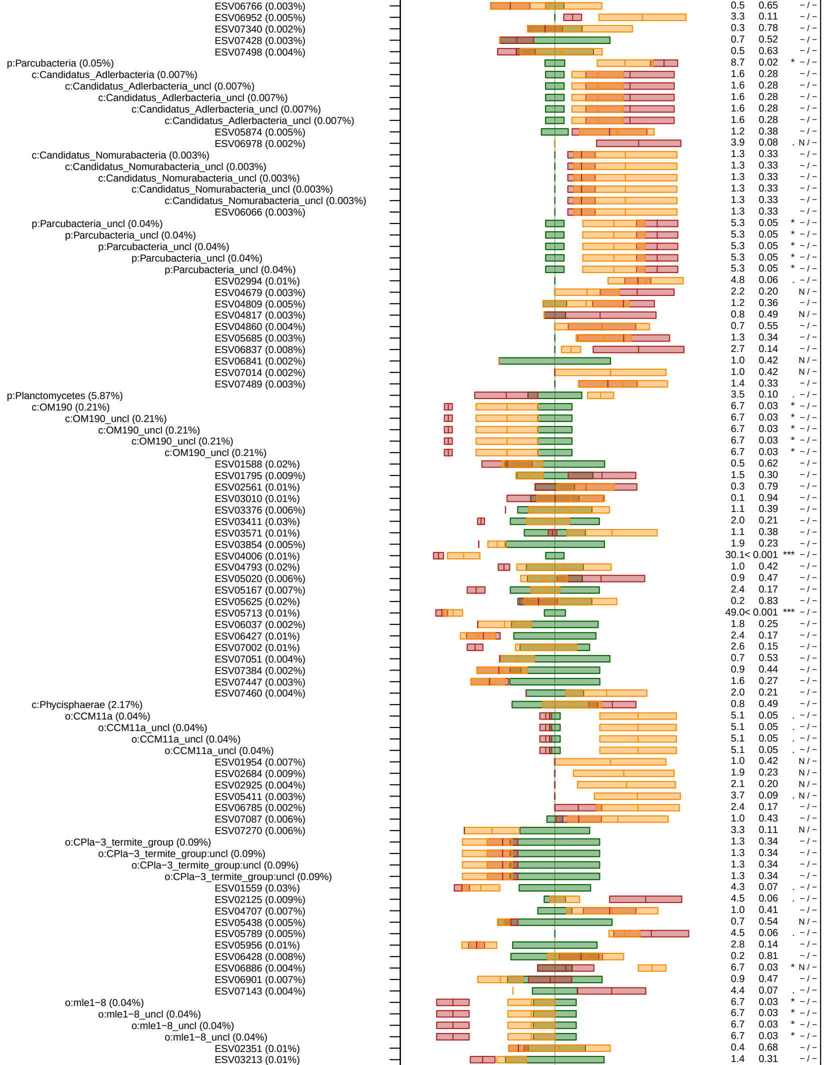

ESV06766 (0.003%) 0.5 0.65 − / −
ESV06952 (0.005%) 3.3 0.11 − / −
ESV07340 (0.002%) 0.3 0.78 − / −
ESV07428 (0.003%) 0.7 0.52 − / −
ESV07498 (0.004%) 0.5 0.63 − / −
p:Parcubacteria (0.05%) 8.7 0.02 * − / −
c:Candidatus_Adlerbacteria (0.007%) 1.6 0.28 − / −
c:Candidatus_Adlerbacteria_uncl (0.007%) 1.6 0.28 − / −
c:Candidatus_Adlerbacteria_uncl (0.007%) 1.6 0.28 − / −
c:Candidatus_Adlerbacteria_uncl (0.007%) 1.6 0.28 − / −
c:Candidatus_Adlerbacteria_uncl (0.007%) 1.6 0.28 − / −
ESV05874 (0.005%) 1.2 0.38 − / −
ESV06978 (0.002%) 3.9 0.08 . N / −
c:Candidatus_Nomurabacteria (0.003%) 1.3 0.33 − / −
c:Candidatus_Nomurabacteria_uncl (0.003%) 1.3 0.33 − / −
c:Candidatus_Nomurabacteria_uncl (0.003%) 1.3 0.33 − / −
c:Candidatus_Nomurabacteria_uncl (0.003%) 1.3 0.33 − / −
c:Candidatus_Nomurabacteria_uncl (0.003%) 1.3 0.33 − / −
ESV06066 (0.003%) 1.3 0.33 − / −
p:Parcubacteria_uncl (0.04%) 5.3 0.05 * − / −
p:Parcubacteria_uncl (0.04%) 5.3 0.05 * − / −
p:Parcubacteria_uncl (0.04%) 5.3 0.05 * − / −
p:Parcubacteria_uncl (0.04%) 5.3 0.05 * − / −
p:Parcubacteria_uncl (0.04%) 5.3 0.05 * − / −
ESV02994 (0.01%) 4.8 0.06 . − / −
ESV04679 (0.003%) 2.2 0.20 N / −
ESV04809 (0.005%) 1.2 0.36 − / −
ESV04817 (0.003%) 0.8 0.49 N / −
ESV04860 (0.004%) 0.7 0.55 − / −
ESV05685 (0.003%) 1.3 0.34 − / −
ESV06837 (0.008%) 2.7 0.14 − / −
ESV06841 (0.002%) 1.0 0.42 N / −
ESV07014 (0.002%) 1.0 0.42 N / −
ESV07489 (0.003%) 1.4 0.33 − / −
p:Planctomycetes (5.87%) 3.5 0.10 . − / −
c:OM190 (0.21%) 6.7 0.03 * − / −
c:OM190_uncl (0.21%) 6.7 0.03 * − / −
c:OM190_uncl (0.21%) 6.7 0.03 * − / −
c:OM190_uncl (0.21%) 6.7 0.03 * − / −
c:OM190_uncl (0.21%) 6.7 0.03 * − / −
ESV01588 (0.02%) 0.5 0.62 − / −
ESV01795 (0.009%) 1.5 0.30 − / −
ESV02561 (0.01%) 0.3 0.79 − / −
ESV03010 (0.01%) 0.1 0.94 − / −
ESV03376 (0.006%) 1.1 0.39 − / −
ESV03411 (0.03%) 2.0 0.21 − / −
ESV03571 (0.01%) 1.1 0.38 − / −
ESV03854 (0.005%) 1.9 0.23 − / −
ESV04006 (0.01%) 30.1< 0.001 *** − / −
ESV04793 (0.02%) 1.0 0.42 − / −
ESV05020 (0.006%) 0.9 0.47 − / −
ESV05167 (0.007%) 2.4 0.17 − / −
ESV05625 (0.02%) 0.2 0.83 − / −
ESV05713 (0.01%) 49.0< 0.001 *** − / −
ESV06037 (0.002%) 1.8 0.25 − / −
ESV06427 (0.01%) 2.4 0.17 − / −
ESV07002 (0.01%) 2.6 0.15 − / −
ESV07051 (0.004%) 0.7 0.53 − / −
ESV07384 (0.002%) 0.9 0.44 − / −
ESV07447 (0.003%) 1.6 0.27 − / −
ESV07460 (0.004%) 2.0 0.21 − / −
c:Phycisphaerae (2.17%) 0.8 0.49 − / −
o:CCM11a (0.04%) 5.1 0.05 . − / −
o:CCM11a_uncl (0.04%) 5.1 0.05 . − / −
o:CCM11a_uncl (0.04%) 5.1 0.05 . − / −
o:CCM11a_uncl (0.04%) 5.1 0.05 . − / −
ESV01954 (0.007%) 1.0 0.42 N / −
ESV02684 (0.009%) 1.9 0.23 N / −
ESV02925 (0.004%) 2.1 0.20 N / −
ESV05411 (0.003%) 3.7 0.09 . N / −
ESV06785 (0.002%) 2.4 0.17 − / −
ESV07087 (0.006%) 1.0 0.43 − / −
ESV07270 (0.006%) 3.3 0.11 N / −
o:CPla−3_termite_group (0.09%) 1.3 0.34 − / −
o:CPla−3_termite_group:uncl (0.09%) 1.3 0.34 − / −
o:CPla−3_termite_group:uncl (0.09%) 1.3 0.34 − / −
o:CPla−3_termite_group:uncl (0.09%) 1.3 0.34 − / −
ESV01559 (0.03%) 4.3 0.07 . − / −
ESV02125 (0.009%) 4.5 0.06 . − / −
ESV04707 (0.007%) 1.0 0.41 − / −
ESV05438 (0.005%) 0.7 0.54 N / −
ESV05789 (0.005%) 4.5 0.06 . − / −
ESV05956 (0.01%) 2.8 0.14 − / −
ESV06428 (0.008%) 0.2 0.81 − / −
ESV06886 (0.004%) 6.7 0.03 * N / −
ESV06901 (0.007%) 0.9 0.47 − / −
ESV07143 (0.004%) 4.4 0.07 . − / −
o:mle1−8 (0.04%) 6.7 0.03 * − / −
o:mle1−8_uncl (0.04%) 6.7 0.03 * − / −
o:mle1−8_uncl (0.04%) 6.7 0.03 * − / −
o:mle1−8_uncl (0.04%) 6.7 0.03 * − / −
ESV02351 (0.01%) 0.4 0.68 − / −
ESV03213 (0.01%) 1.4 0.31 − / −

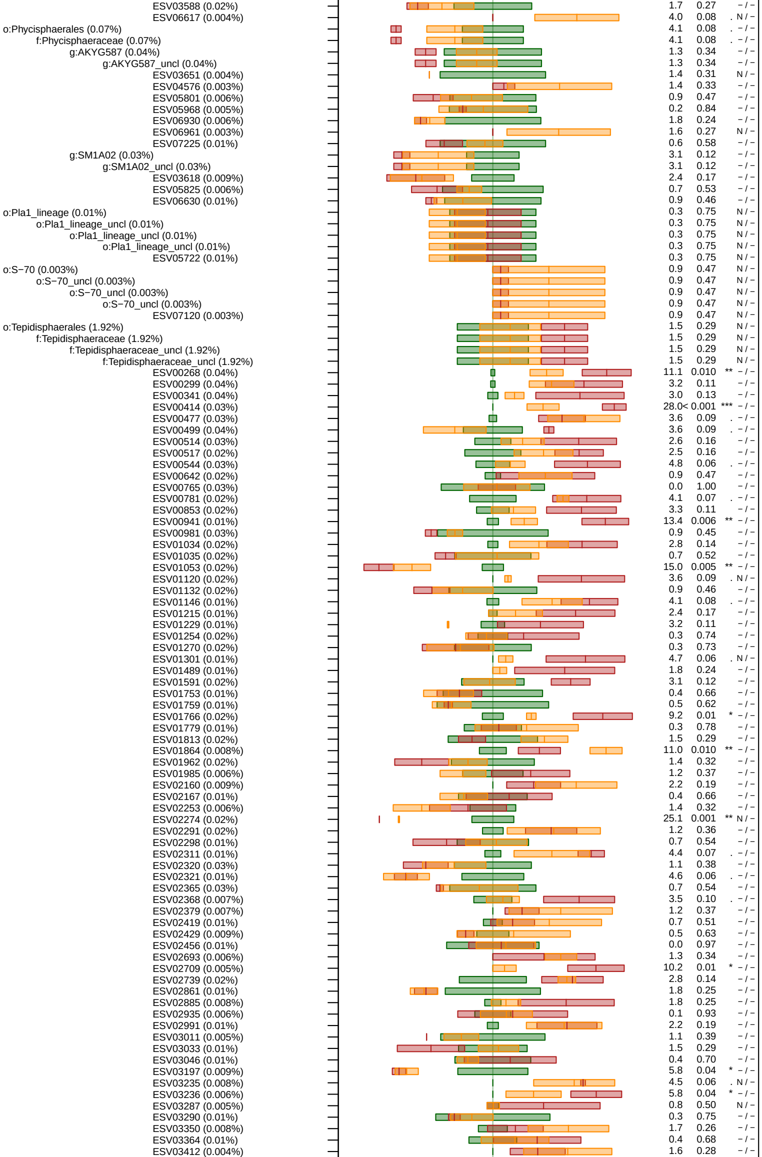

ESV03588 (0.02%) 1.7 0.27 - / -
ESV06617 (0.004%) 4.0 0.08 . N / -
o:Phycisphaerales (0.07%) 4.1 0.08 . - / -
f:Phycisphaeraceae (0.07%) 4.1 0.08 . - / -
g:AKYG587 (0.04%) 1.3 0.34 - / -
g:AKYG587_uncl (0.04%) 1.3 0.34 - / -
ESV03651 (0.004%) 1.4 0.31 N / -
ESV04576 (0.003%) 1.4 0.33 - / -
ESV05801 (0.006%) 0.9 0.47 - / -
ESV05968 (0.005%) 0.2 0.84 - / -
ESV06930 (0.006%) 1.8 0.24 - / -
ESV06961 (0.003%) 1.6 0.27 N / -
ESV07225 (0.01%) 0.6 0.58 - / -
g:SM1A02 (0.03%) 3.1 0.12 - / -
g:SM1A02_uncl (0.03%) 3.1 0.12 - / -
ESV03618 (0.009%) 2.4 0.17 - / -
ESV05825 (0.006%) 0.7 0.53 - / -
ESV06630 (0.01%) 0.9 0.46 - / -
o:Pla1_lineage (0.01%) 0.3 0.75 N / -
o:Pla1_lineage_uncl (0.01%) 0.3 0.75 N / -
o:Pla1_lineage_uncl (0.01%) 0.3 0.75 N / -
o:Pla1_lineage_uncl (0.01%) 0.3 0.75 N / -
ESV05722 (0.01%) 0.3 0.75 N / -
o:S−70 (0.003%) 0.9 0.47 N / -
o:S−70_uncl (0.003%) 0.9 0.47 N / -
o:S−70_uncl (0.003%) 0.9 0.47 N / -
o:S−70_uncl (0.003%) 0.9 0.47 N / -
ESV07120 (0.003%) 0.9 0.47 N / -
o:Tepidisphaerales (1.92%) 1.5 0.29 N / -
f:Tepidisphaeraceae (1.92%) 1.5 0.29 N / -
f:Tepidisphaeraceae_uncl (1.92%) 1.5 0.29 N / -
f:Tepidisphaeraceae_uncl (1.92%) 1.5 0.29 N / -
ESV00268 (0.04%) 11.1 0.010 ** - / -
ESV00299 (0.04%) 3.2 0.11 - / -
ESV00341 (0.04%) 3.0 0.13 - / -
ESV00414 (0.03%) 28.0 < 0.001 *** - / -
ESV00477 (0.03%) 3.6 0.09 . - / -
ESV00499 (0.04%) 3.6 0.09 . - / -
ESV00514 (0.03%) 2.6 0.16 - / -
ESV00517 (0.02%) 2.5 0.16 - / -
ESV00544 (0.03%) 4.8 0.06 . - / -
ESV00642 (0.02%) 0.9 0.47 - / -
ESV00765 (0.03%) 0.0 1.00 - / -
ESV00781 (0.02%) 4.1 0.07 . - / -
ESV00853 (0.02%) 3.3 0.11 - / -
ESV00941 (0.01%) 13.4 0.006 ** - / -
ESV00981 (0.03%) 0.9 0.45 - / -
ESV01034 (0.02%) 2.8 0.14 - / -
ESV01035 (0.02%) 0.7 0.52 - / -
ESV01053 (0.02%) 15.0 0.005 ** - / -
ESV01120 (0.02%) 3.6 0.09 . N / -
ESV01132 (0.02%) 0.9 0.46 - / -
ESV01146 (0.01%) 4.1 0.08 . - / -
ESV01215 (0.01%) 2.4 0.17 - / -
ESV01229 (0.01%) 3.2 0.11 - / -
ESV01254 (0.02%) 0.3 0.74 - / -
ESV01270 (0.02%) 0.3 0.73 - / -
ESV01301 (0.01%) 4.7 0.06 . N / -
ESV01489 (0.01%) 1.8 0.24 - / -
ESV01591 (0.02%) 3.1 0.12 - / -
ESV01753 (0.01%) 0.4 0.66 - / -
ESV01759 (0.01%) 0.5 0.62 - / -
ESV01766 (0.02%) 9.2 0.01 * - / -
ESV01779 (0.01%) 0.3 0.78 - / -
ESV01813 (0.02%) 1.5 0.29 - / -
ESV01864 (0.008%) 11.0 0.010 ** - / -
ESV01962 (0.02%) 1.4 0.32 - / -
ESV01985 (0.006%) 1.2 0.37 - / -
ESV02160 (0.009%) 2.2 0.19 - / -
ESV02167 (0.01%) 0.4 0.66 - / -
ESV02253 (0.006%) 1.4 0.32 - / -
ESV02274 (0.02%) 25.1 0.001 ** N / -
ESV02291 (0.02%) 1.2 0.36 - / -
ESV02298 (0.01%) 0.7 0.54 - / -
ESV02311 (0.01%) 4.4 0.07 . - / -
ESV02320 (0.03%) 1.1 0.38 - / -
ESV02321 (0.01%) 4.6 0.06 . - / -
ESV02365 (0.03%) 0.7 0.54 - / -
ESV02368 (0.007%) 3.5 0.10 . - / -
ESV02379 (0.007%) 1.2 0.37 - / -
ESV02419 (0.01%) 0.7 0.51 - / -
ESV02429 (0.009%) 0.5 0.63 - / -
ESV02456 (0.01%) 0.0 0.97 - / -
ESV02693 (0.006%) 1.3 0.34 - / -
ESV02709 (0.005%) 10.2 0.01 * - / -
ESV02739 (0.02%) 2.8 0.14 - / -
ESV02861 (0.01%) 1.8 0.25 - / -
ESV02885 (0.008%) 1.8 0.25 - / -
ESV02935 (0.006%) 0.1 0.93 - / -
ESV02991 (0.01%) 2.2 0.19 - / -
ESV03011 (0.005%) 1.1 0.39 - / -
ESV03033 (0.01%) 1.5 0.29 - / -
ESV03046 (0.01%) 0.4 0.70 - / -
ESV03197 (0.009%) 5.8 0.04 * - / -
ESV03235 (0.008%) 4.5 0.06 . N / -
ESV03236 (0.006%) 5.8 0.04 * - / -
ESV03287 (0.005%) 0.8 0.50 N / -
ESV03290 (0.01%) 0.3 0.75 - / -
ESV03350 (0.008%) 1.7 0.26 - / -
ESV03364 (0.01%) 0.4 0.68 - / -
ESV03412 (0.004%) 1.6 0.28 - / -

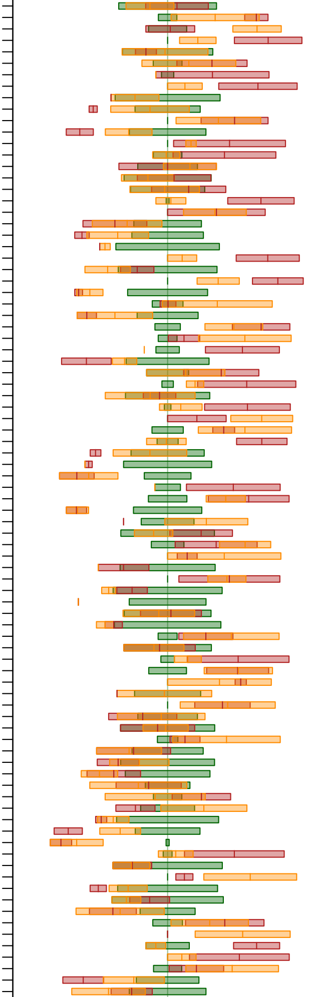

ESV03563 (0.009%)	0.1	0.87		– / –
ESV03608 (0.009%)	2.7	0.14		– / –
ESV03732 (0.006%)	5.0	0.05	.	– / –
ESV03734 (0.006%)	5.4	0.05	*	– / –
ESV03796 (0.007%)	0.1	0.90		– / –
ESV03816 (0.008%)	0.4	0.68		– / –
ESV03828 (0.007%)	0.8	0.48		N / –
ESV03845 (0.005%)	3.8	0.09	.	– / –
ESV03857 (0.01%)	0.7	0.52		– / –
ESV03861 (0.01%)	1.7	0.25		– / –
ESV03892 (0.009%)	1.3	0.35		– / –
ESV03914 (0.02%)	2.6	0.15		– / –
ESV03924 (0.008%)	0.9	0.45		N / –
ESV03950 (0.005%)	1.1	0.39		– / –
ESV03951 (0.009%)	0.1	0.88		– / –
ESV04030 (0.005%)	0.1	0.87		– / –
ESV04060 (0.008%)	0.1	0.88		– / –
ESV04083 (0.02%)	6.3	0.03	*	– / –
ESV04103 (0.007%)	0.7	0.54		– / –
ESV04104 (0.01%)	0.7	0.54		N / –
ESV04124 (0.007%)	2.4	0.17		– / –
ESV04158 (0.006%)	1.6	0.28		N / –
ESV04206 (0.003%)	7.3	0.02	*	– / –
ESV04256 (0.009%)	0.8	0.48		– / –
ESV04269 (0.01%)	8.4	0.02	*	– / –
ESV04294 (0.02%)	3.9	0.08	.	– / –
ESV04334 (0.006%)	0.7	0.52		– / –
ESV04377 (0.007%)	2.1	0.21		– / –
ESV04458 (0.008%)	3.8	0.09	.	– / –
ESV04480 (0.006%)	2.0	0.22		– / –
ESV04491 (0.005%)	5.2	0.05	*	– / –
ESV04582 (0.03%)	2.0	0.22		– / –
ESV04587 (0.003%)	0.7	0.55		– / –
ESV04593 (0.01%)	1.9	0.23		– / –
ESV04623 (0.008%)	0.1	0.95		– / –
ESV04635 (0.01%)	2.4	0.17		– / –
ESV04685 (0.005%)	3.8	0.08	.	– / –
ESV04804 (0.005%)	1.9	0.23		– / –
ESV04816 (0.007%)	7.9	0.02	*	– / –
ESV04850 (0.01%)	1.6	0.29		– / –
ESV04863 (0.009%)	3.3	0.11		N / –
ESV04886 (0.007%)	4.3	0.07	.	– / –
ESV04948 (0.003%)	2.3	0.19		– / –
ESV05019 (0.004%)	2.2	0.19		– / –
ESV05146 (0.004%)	5.9	0.04	*	– / –
ESV05206 (0.006%)	2.1	0.20		– / –
ESV05301 (0.006%)	0.5	0.63		– / –
ESV05369 (0.005%)	1.8	0.25		– / –
ESV05385 (0.003%)	0.8	0.51		N / –
ESV05393 (0.004%)	1.3	0.35		– / –
ESV05395 (0.008%)	1.3	0.35		– / –
ESV05424 (0.01%)	0.8	0.49		– / –
ESV05446 (0.006%)	5.4	0.05	*	N / –
ESV05514 (0.007%)	0.0	0.97		N / –
ESV05519 (0.005%)	1.2	0.38		– / –
ESV05605 (0.006%)	1.0	0.42		– / –
ESV05711 (0.003%)	0.0	0.97		N / –
ESV05712 (0.007%)	1.4	0.32		– / –
ESV05793 (0.008%)	2.0	0.22		– / –
ESV05866 (0.004%)	1.6	0.28		N / –
ESV05869 (0.004%)	0.7	0.52		– / –
ESV05907 (0.006%)	1.1	0.39		– / –
ESV05917 (0.01%)	0.1	0.88		– / –
ESV05955 (0.003%)	0.0	0.98		N / –
ESV06039 (0.005%)	0.9	0.47		– / –
ESV06044 (0.002%)	0.4	0.71		– / –
ESV06048 (0.007%)	0.5	0.62		– / –
ESV06068 (0.01%)	1.4	0.32		– / –
ESV06102 (0.007%)	0.2	0.85		– / –
ESV06137 (0.005%)	0.5	0.64		– / –
ESV06180 (0.007%)	1.2	0.37		– / –
ESV06238 (0.009%)	1.3	0.34		– / –
ESV06274 (0.02%)	4.5	0.07	.	– / –
ESV06280 (0.005%)	12.2	0.008	**	– / –
ESV06285 (0.01%)	1.4	0.31		– / –
ESV06307 (0.002%)	0.3	0.72		– / –
ESV06308 (0.005%)	2.6	0.16		N / –
ESV06319 (0.006%)	1.2	0.35		– / –
ESV06327 (0.003%)	0.3	0.72		– / –
ESV06350 (0.01%)	0.8	0.49		– / –
ESV06351 (0.005%)	0.8	0.49		– / –
ESV06395 (0.003%)	2.5	0.16		N / –
ESV06446 (0.006%)	8.3	0.02	*	– / –
ESV06468 (0.003%)	1.6	0.28		– / –
ESV06481 (0.004%)	1.0	0.42		– / –
ESV06496 (0.01%)	2.3	0.18		– / –
ESV06541 (0.01%)	0.8	0.48		– / –

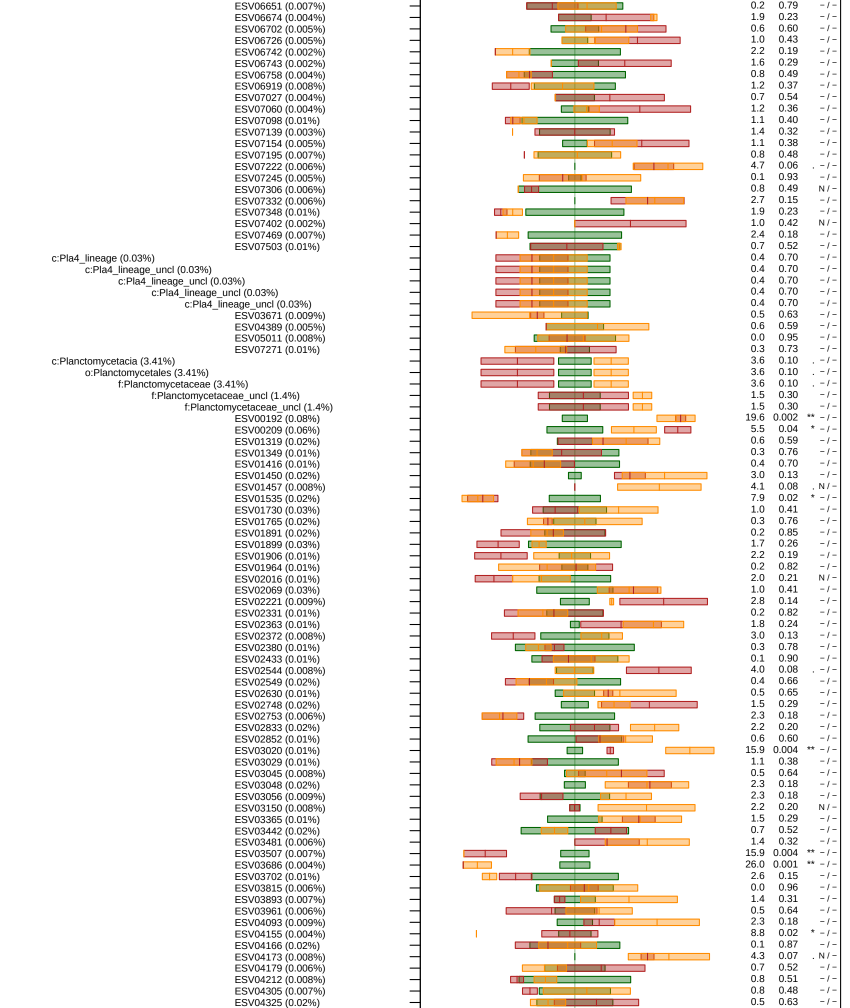
ESV06651 (0.007%) 0.2 0.79 - / -
ESV06674 (0.004%) 1.9 0.23 - / -
ESV06702 (0.005%) 0.6 0.60 - / -
ESV06726 (0.005%) 1.0 0.43 - / -
ESV06742 (0.002%) 2.2 0.19 - / -
ESV06743 (0.002%) 1.6 0.29 - / -
ESV06758 (0.004%) 0.8 0.49 - / -
ESV06919 (0.008%) 1.2 0.37 - / -
ESV07027 (0.004%) 0.7 0.54 - / -
ESV07060 (0.004%) 1.2 0.36 - / -
ESV07098 (0.01%) 1.1 0.40 - / -
ESV07139 (0.003%) 1.4 0.32 - / -
ESV07154 (0.005%) 1.1 0.38 - / -
ESV07195 (0.007%) 0.8 0.48 - / -
ESV07222 (0.006%) 4.7 0.06 . - / -
ESV07245 (0.005%) 0.1 0.93 - / -
ESV07306 (0.006%) 0.8 0.49 N / -
ESV07332 (0.006%) 2.7 0.15 - / -
ESV07348 (0.01%) 1.9 0.23 - / -
ESV07402 (0.002%) 1.0 0.42 N / -
ESV07469 (0.007%) 2.4 0.18 - / -
ESV07503 (0.01%) 0.7 0.52 - / -
c:Pla4_lineage (0.03%) 0.4 0.70 - / -
c:Pla4_lineage_uncl (0.03%) 0.4 0.70 - / -
c:Pla4_lineage_uncl (0.03%) 0.4 0.70 - / -
c:Pla4_lineage_uncl (0.03%) 0.4 0.70 - / -
c:Pla4_lineage_uncl (0.03%) 0.4 0.70 - / -
ESV03671 (0.009%) 0.5 0.63 - / -
ESV04389 (0.005%) 0.6 0.59 - / -
ESV05011 (0.008%) 0.0 0.95 - / -
ESV07271 (0.01%) 0.3 0.73 - / -
c:Planctomycetacia (3.41%) 3.6 0.10 . - / -
o:Planctomycetales (3.41%) 3.6 0.10 . - / -
f:Planctomycetaceae (3.41%) 3.6 0.10 . - / -
f:Planctomycetaceae_uncl (1.4%) 1.5 0.30 - / -
f:Planctomycetaceae_uncl (1.4%) 1.5 0.30 - / -
ESV00192 (0.08%) 19.6 0.002 ** - / -
ESV00209 (0.06%) 5.5 0.04 * - / -
ESV01319 (0.02%) 0.6 0.59 - / -
ESV01349 (0.01%) 0.3 0.76 - / -
ESV01416 (0.01%) 0.4 0.70 - / -
ESV01450 (0.02%) 3.0 0.13 . N / -
ESV01457 (0.008%) 4.1 0.08 . N / -
ESV01535 (0.02%) 7.9 0.02 * - / -
ESV01730 (0.03%) 1.0 0.41 - / -
ESV01765 (0.02%) 0.3 0.76 - / -
ESV01891 (0.02%) 0.2 0.85 - / -
ESV01899 (0.03%) 1.7 0.26 - / -
ESV01906 (0.01%) 2.2 0.19 - / -
ESV01964 (0.01%) 0.2 0.82 - / -
ESV02016 (0.01%) 2.0 0.21 N / -
ESV02069 (0.03%) 1.0 0.41 - / -
ESV02221 (0.009%) 2.8 0.14 - / -
ESV02331 (0.01%) 0.2 0.82 - / -
ESV02363 (0.01%) 1.8 0.24 - / -
ESV02372 (0.008%) 3.0 0.13 - / -
ESV02380 (0.01%) 0.3 0.78 - / -
ESV02433 (0.01%) 0.1 0.90 - / -
ESV02544 (0.008%) 4.0 0.08 . - / -
ESV02549 (0.02%) 0.4 0.66 - / -
ESV02630 (0.01%) 0.5 0.65 - / -
ESV02748 (0.02%) 1.5 0.29 - / -
ESV02753 (0.006%) 2.3 0.18 - / -
ESV02833 (0.02%) 2.2 0.20 - / -
ESV02852 (0.01%) 0.6 0.60 - / -
ESV03020 (0.01%) 15.9 0.004 ** - / -
ESV03029 (0.01%) 1.1 0.38 - / -
ESV03045 (0.008%) 0.5 0.64 - / -
ESV03048 (0.02%) 2.3 0.18 - / -
ESV03056 (0.009%) 2.3 0.18 - / -
ESV03150 (0.008%) 2.2 0.20 N / -
ESV03365 (0.01%) 1.5 0.29 - / -
ESV03442 (0.02%) 0.7 0.52 - / -
ESV03481 (0.006%) 1.4 0.32 - / -
ESV03507 (0.007%) 15.9 0.004 ** - / -
ESV03686 (0.004%) 26.0 0.001 ** - / -
ESV03702 (0.01%) 2.6 0.15 - / -
ESV03815 (0.006%) 0.0 0.96 - / -
ESV03893 (0.007%) 1.4 0.31 - / -
ESV03961 (0.006%) 0.5 0.64 - / -
ESV04093 (0.009%) 2.3 0.18 - / -
ESV04155 (0.004%) 8.8 0.02 * - / -
ESV04166 (0.02%) 0.1 0.87 - / -
ESV04173 (0.008%) 4.3 0.07 . N / -
ESV04179 (0.006%) 0.7 0.52 - / -
ESV04212 (0.008%) 0.8 0.51 - / -
ESV04305 (0.007%) 0.8 0.48 - / -
ESV04325 (0.02%) 0.5 0.63 - / -

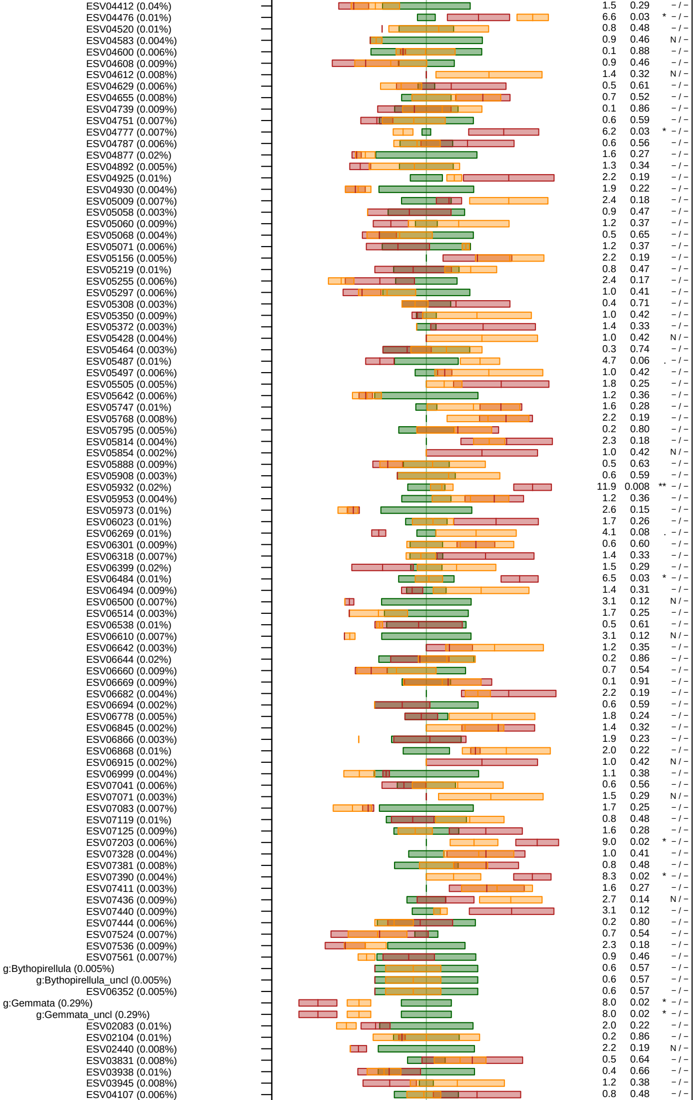
ESV04412 (0.04%) 1.5 0.29 – / –
ESV04476 (0.01%) 6.6 0.03 * – / –
ESV04520 (0.01%) 0.8 0.48 – / –
ESV04583 (0.004%) 0.9 0.46 N / –
ESV04600 (0.006%) 0.1 0.88 – / –
ESV04608 (0.009%) 0.9 0.46 – / –
ESV04612 (0.008%) 1.4 0.32 N / –
ESV04629 (0.006%) 0.5 0.61 – / –
ESV04655 (0.008%) 0.7 0.52 – / –
ESV04739 (0.009%) 0.1 0.86 – / –
ESV04751 (0.007%) 0.6 0.59 – / –
ESV04777 (0.007%) 6.2 0.03 * – / –
ESV04787 (0.006%) 0.6 0.56 – / –
ESV04877 (0.02%) 1.6 0.27 – / –
ESV04892 (0.005%) 1.3 0.34 – / –
ESV04925 (0.01%) 2.2 0.19 – / –
ESV04930 (0.004%) 1.9 0.22 – / –
ESV05009 (0.007%) 2.4 0.18 – / –
ESV05058 (0.003%) 0.9 0.47 – / –
ESV05060 (0.009%) 1.2 0.37 – / –
ESV05068 (0.004%) 0.5 0.65 – / –
ESV05071 (0.006%) 1.2 0.37 – / –
ESV05156 (0.005%) 2.2 0.19 – / –
ESV05219 (0.01%) 0.8 0.47 – / –
ESV05255 (0.006%) 2.4 0.17 – / –
ESV05297 (0.006%) 1.0 0.41 – / –
ESV05308 (0.003%) 0.4 0.71 – / –
ESV05350 (0.009%) 1.0 0.42 – / –
ESV05372 (0.003%) 1.4 0.33 – / –
ESV05428 (0.004%) 1.0 0.42 N / –
ESV05464 (0.003%) 0.3 0.74 – / –
ESV05487 (0.01%) 4.7 0.06 . – / –
ESV05497 (0.006%) 1.0 0.42 – / –
ESV05505 (0.005%) 1.8 0.25 – / –
ESV05642 (0.006%) 1.2 0.36 – / –
ESV05747 (0.01%) 1.6 0.28 – / –
ESV05768 (0.008%) 2.2 0.19 – / –
ESV05795 (0.005%) 0.2 0.80 – / –
ESV05814 (0.004%) 2.3 0.18 – / –
ESV05854 (0.002%) 1.0 0.42 N / –
ESV05888 (0.009%) 0.5 0.63 – / –
ESV05908 (0.003%) 0.6 0.59 – / –
ESV05932 (0.02%) 11.9 0.008 ** – / –
ESV05953 (0.004%) 1.2 0.36 – / –
ESV05973 (0.01%) 2.6 0.15 – / –
ESV06023 (0.01%) 1.7 0.26 – / –
ESV06269 (0.01%) 4.1 0.08 . – / –
ESV06301 (0.009%) 0.6 0.60 – / –
ESV06318 (0.007%) 1.4 0.33 – / –
ESV06399 (0.02%) 1.5 0.29 – / –
ESV06484 (0.01%) 6.5 0.03 * – / –
ESV06494 (0.009%) 1.4 0.31 – / –
ESV06500 (0.007%) 3.1 0.12 N / –
ESV06514 (0.003%) 1.7 0.25 – / –
ESV06538 (0.01%) 0.5 0.61 – / –
ESV06610 (0.007%) 3.1 0.12 N / –
ESV06642 (0.003%) 1.2 0.35 – / –
ESV06644 (0.02%) 0.2 0.86 – / –
ESV06660 (0.009%) 0.7 0.54 – / –
ESV06669 (0.009%) 0.1 0.91 – / –
ESV06682 (0.004%) 2.2 0.19 – / –
ESV06694 (0.002%) 0.6 0.59 – / –
ESV06778 (0.005%) 1.8 0.24 – / –
ESV06845 (0.002%) 1.4 0.32 – / –
ESV06866 (0.003%) 1.9 0.23 – / –
ESV06868 (0.01%) 2.0 0.22 – / –
ESV06915 (0.002%) 1.0 0.42 N / –
ESV06999 (0.004%) 1.1 0.38 – / –
ESV07041 (0.006%) 0.6 0.56 – / –
ESV07071 (0.003%) 1.5 0.29 N / –
ESV07083 (0.007%) 1.7 0.25 – / –
ESV07119 (0.01%) 0.8 0.48 – / –
ESV07125 (0.009%) 1.6 0.28 – / –
ESV07203 (0.006%) 9.0 0.02 * – / –
ESV07328 (0.004%) 1.0 0.41 – / –
ESV07381 (0.008%) 0.8 0.48 – / –
ESV07390 (0.004%) 8.3 0.02 * – / –
ESV07411 (0.003%) 1.6 0.27 – / –
ESV07436 (0.009%) 2.7 0.14 N / –
ESV07440 (0.009%) 3.1 0.12 – / –
ESV07444 (0.006%) 0.2 0.80 – / –
ESV07524 (0.007%) 0.7 0.54 – / –
ESV07536 (0.009%) 2.3 0.18 – / –
ESV07561 (0.007%) 0.9 0.46 – / –
g:Bythopirellula (0.005%) 0.6 0.57 – / –
g:Bythopirellula_uncl (0.005%) 0.6 0.57 – / –
ESV06352 (0.005%) 0.6 0.57 – / –
g:Gemmata (0.29%) 8.0 0.02 * – / –
g:Gemmata_uncl (0.29%) 8.0 0.02 * – / –
ESV02083 (0.01%) 2.0 0.22 – / –
ESV02104 (0.01%) 0.2 0.86 – / –
ESV02440 (0.008%) 2.2 0.19 N / –
ESV03831 (0.008%) 0.5 0.64 – / –
ESV03938 (0.01%) 0.4 0.66 – / –
ESV03945 (0.008%) 1.2 0.38 – / –
ESV04107 (0.006%) 0.8 0.48 – / –

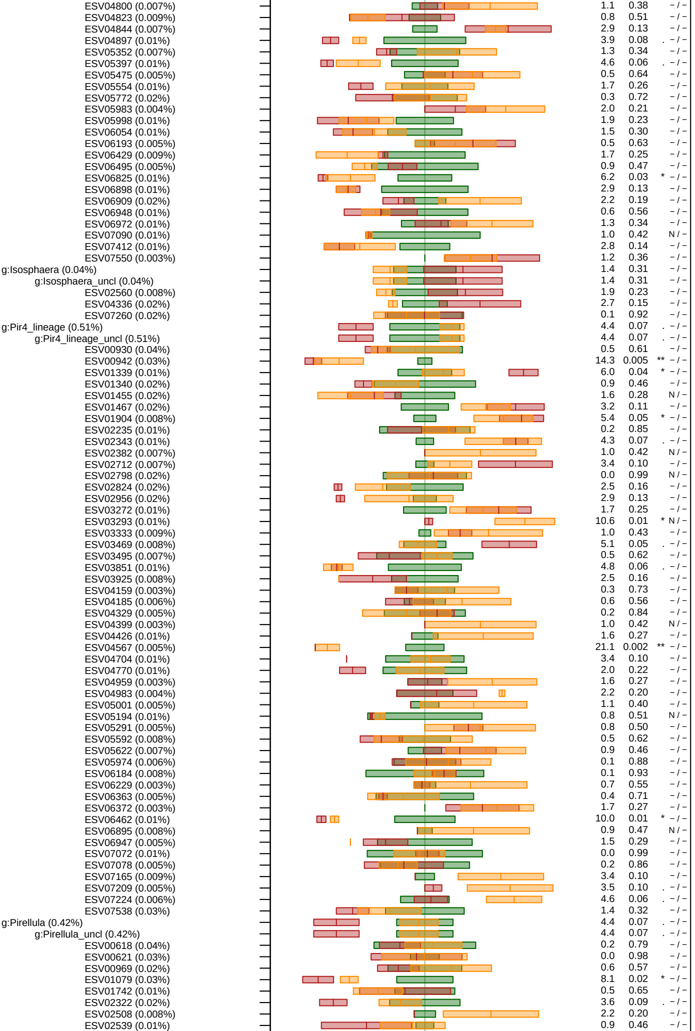
ESV04800 (0.007%) 1.1 0.38 - / -
ESV04823 (0.009%) 0.8 0.51 - / -
ESV04844 (0.007%) 2.9 0.13 - / -
ESV04897 (0.01%) 3.9 0.08 . - / -
ESV05352 (0.007%) 1.3 0.34 - / -
ESV05397 (0.01%) 4.6 0.06 . - / -
ESV05475 (0.005%) 0.5 0.64 - / -
ESV05554 (0.01%) 1.7 0.26 - / -
ESV05772 (0.02%) 0.3 0.72 - / -
ESV05983 (0.004%) 2.0 0.21 - / -
ESV05998 (0.01%) 1.9 0.23 - / -
ESV06054 (0.01%) 1.5 0.30 - / -
ESV06193 (0.005%) 0.5 0.63 - / -
ESV06429 (0.009%) 1.7 0.25 - / -
ESV06495 (0.005%) 0.9 0.47 - / -
ESV06825 (0.01%) 6.2 0.03 * - / -
ESV06898 (0.01%) 2.9 0.13 - / -
ESV06909 (0.02%) 2.2 0.19 - / -
ESV06948 (0.01%) 0.6 0.56 - / -
ESV06972 (0.01%) 1.3 0.34 - / -
ESV07090 (0.01%) 1.0 0.42 N / -
ESV07412 (0.01%) 2.8 0.14 - / -
ESV07550 (0.003%) 1.2 0.36 - / -
g:Isosphaera (0.04%) 1.4 0.31 - / -
g:Isosphaera_uncl (0.04%) 1.4 0.31 - / -
ESV02560 (0.008%) 1.9 0.23 - / -
ESV04336 (0.02%) 2.7 0.15 - / -
ESV07260 (0.02%) 0.1 0.92 - / -
g:Pir4_lineage (0.51%) 4.4 0.07 . - / -
g:Pir4_lineage_uncl (0.51%) 4.4 0.07 . - / -
ESV00930 (0.04%) 0.5 0.61 - / -
ESV00942 (0.03%) 14.3 0.005 ** - / -
ESV01339 (0.01%) 6.0 0.04 * - / -
ESV01340 (0.02%) 0.9 0.46 - / -
ESV01455 (0.02%) 1.6 0.28 N / -
ESV01467 (0.02%) 3.2 0.11 - / -
ESV01904 (0.008%) 5.4 0.05 * - / -
ESV02235 (0.01%) 0.2 0.85 - / -
ESV02343 (0.01%) 4.3 0.07 . - / -
ESV02382 (0.007%) 1.0 0.42 N / -
ESV02712 (0.007%) 3.4 0.10 - / -
ESV02798 (0.02%) 0.0 0.99 N / -
ESV02824 (0.02%) 2.5 0.16 - / -
ESV02956 (0.02%) 2.9 0.13 - / -
ESV03272 (0.01%) 1.7 0.25 - / -
ESV03293 (0.01%) 10.6 0.01 * N / -
ESV03333 (0.009%) 1.0 0.43 - / -
ESV03469 (0.008%) 5.1 0.05 . - / -
ESV03495 (0.007%) 0.5 0.62 - / -
ESV03851 (0.01%) 4.8 0.06 . - / -
ESV03925 (0.008%) 2.5 0.16 - / -
ESV04159 (0.003%) 0.3 0.73 - / -
ESV04185 (0.006%) 0.6 0.56 - / -
ESV04329 (0.005%) 0.2 0.84 - / -
ESV04399 (0.003%) 1.0 0.42 N / -
ESV04426 (0.01%) 1.6 0.27 - / -
ESV04567 (0.005%) 21.1 0.002 ** - / -
ESV04704 (0.01%) 3.4 0.10 - / -
ESV04770 (0.01%) 2.0 0.22 - / -
ESV04959 (0.003%) 1.6 0.27 - / -
ESV04983 (0.004%) 2.2 0.20 - / -
ESV05001 (0.005%) 1.1 0.40 - / -
ESV05194 (0.01%) 0.8 0.51 N / -
ESV05291 (0.005%) 0.8 0.50 - / -
ESV05592 (0.008%) 0.5 0.62 - / -
ESV05622 (0.007%) 0.9 0.46 - / -
ESV05974 (0.006%) 0.1 0.88 - / -
ESV06184 (0.008%) 0.1 0.93 - / -
ESV06229 (0.003%) 0.7 0.55 - / -
ESV06363 (0.005%) 0.4 0.71 - / -
ESV06372 (0.003%) 1.7 0.27 - / -
ESV06462 (0.01%) 10.0 0.01 * - / -
ESV06895 (0.008%) 0.9 0.47 N / -
ESV06947 (0.005%) 1.5 0.29 - / -
ESV07072 (0.01%) 0.0 0.99 - / -
ESV07078 (0.005%) 0.2 0.86 - / -
ESV07165 (0.009%) 3.4 0.10 - / -
ESV07209 (0.005%) 3.5 0.10 . - / -
ESV07224 (0.006%) 4.6 0.06 . - / -
ESV07538 (0.03%) 1.4 0.32 - / -
g:Pirellula (0.42%) 4.4 0.07 . - / -
g:Pirellula_uncl (0.42%) 4.4 0.07 . - / -
ESV00618 (0.04%) 0.2 0.79 - / -
ESV00621 (0.03%) 0.0 0.98 - / -
ESV00969 (0.02%) 0.6 0.57 - / -
ESV01079 (0.03%) 8.1 0.02 * - / -
ESV01742 (0.01%) 0.5 0.65 - / -
ESV02322 (0.02%) 3.6 0.09 . - / -
ESV02508 (0.008%) 2.2 0.20 - / -
ESV02539 (0.01%) 0.9 0.46 - / -

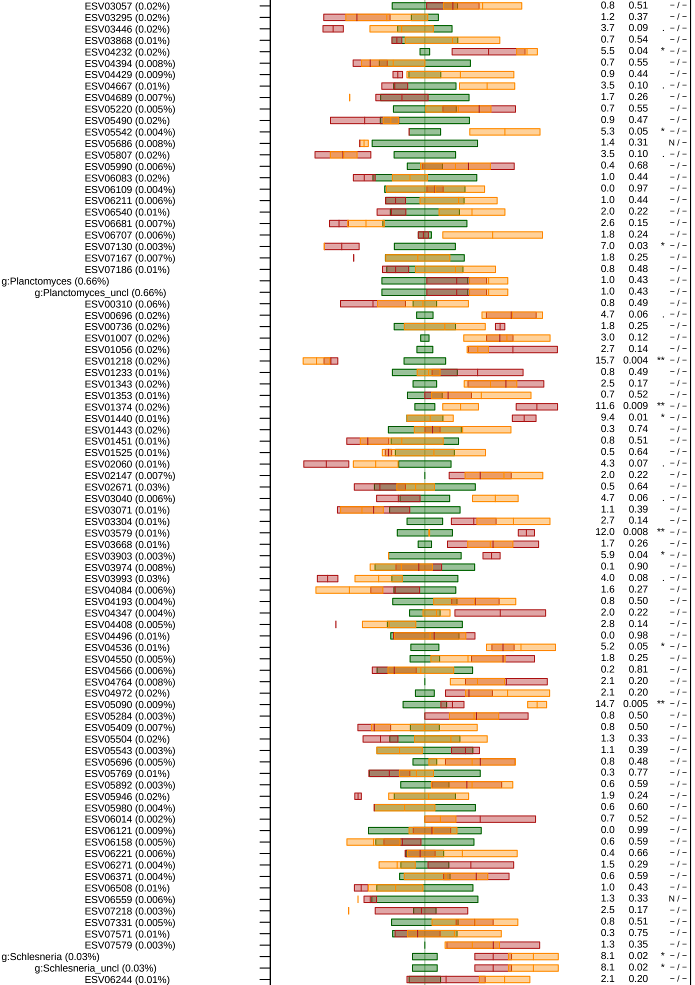
ESV03057 (0.02%) 0.8 0.51 - / -
ESV03295 (0.02%) 1.2 0.37 - / -
ESV03446 (0.02%) 3.7 0.09 . - / -
ESV03868 (0.01%) 0.7 0.54 - / -
ESV04232 (0.02%) 5.5 0.04 * - / -
ESV04394 (0.008%) 0.7 0.55 - / -
ESV04429 (0.009%) 0.9 0.44 - / -
ESV04667 (0.01%) 3.5 0.10 . - / -
ESV04689 (0.007%) 1.7 0.26 - / -
ESV05220 (0.005%) 0.7 0.55 - / -
ESV05490 (0.02%) 0.9 0.47 - / -
ESV05542 (0.004%) 5.3 0.05 * - / -
ESV05686 (0.008%) 1.4 0.31 N / -
ESV05807 (0.02%) 3.5 0.10 . - / -
ESV05990 (0.006%) 0.4 0.68 - / -
ESV06083 (0.02%) 1.0 0.44 - / -
ESV06109 (0.004%) 0.0 0.97 - / -
ESV06211 (0.006%) 1.0 0.44 - / -
ESV06540 (0.01%) 2.0 0.22 - / -
ESV06681 (0.007%) 2.6 0.15 - / -
ESV06707 (0.006%) 1.8 0.24 - / -
ESV07130 (0.003%) 7.0 0.03 * - / -
ESV07167 (0.007%) 1.8 0.25 - / -
ESV07186 (0.01%) 0.8 0.48 - / -
g:Planctomyces (0.66%) 1.0 0.43 - / -
g:Planctomyces_uncl (0.66%) 1.0 0.43 - / -
ESV00310 (0.06%) 0.8 0.49 - / -
ESV00696 (0.02%) 4.7 0.06 . - / -
ESV00736 (0.02%) 1.8 0.25 - / -
ESV01007 (0.02%) 3.0 0.12 - / -
ESV01056 (0.02%) 2.7 0.14 - / -
ESV01218 (0.02%) 15.7 0.004 ** - / -
ESV01233 (0.01%) 0.8 0.49 - / -
ESV01343 (0.02%) 2.5 0.17 - / -
ESV01353 (0.01%) 0.7 0.52 - / -
ESV01374 (0.02%) 11.6 0.009 ** - / -
ESV01440 (0.01%) 9.4 0.01 * - / -
ESV01443 (0.02%) 0.3 0.74 - / -
ESV01451 (0.01%) 0.8 0.51 - / -
ESV01525 (0.01%) 0.5 0.64 - / -
ESV02060 (0.01%) 4.3 0.07 . - / -
ESV02147 (0.007%) 2.0 0.22 - / -
ESV02671 (0.03%) 0.5 0.64 - / -
ESV03040 (0.006%) 4.7 0.06 . - / -
ESV03071 (0.01%) 1.1 0.39 - / -
ESV03304 (0.01%) 2.7 0.14 - / -
ESV03579 (0.01%) 12.0 0.008 ** - / -
ESV03668 (0.01%) 1.7 0.26 - / -
ESV03903 (0.003%) 5.9 0.04 * - / -
ESV03974 (0.008%) 0.1 0.90 - / -
ESV03993 (0.03%) 4.0 0.08 . - / -
ESV04084 (0.006%) 1.6 0.27 - / -
ESV04193 (0.004%) 0.8 0.50 - / -
ESV04347 (0.004%) 2.0 0.22 - / -
ESV04408 (0.005%) 2.8 0.14 - / -
ESV04496 (0.01%) 0.0 0.98 - / -
ESV04536 (0.01%) 5.2 0.05 * - / -
ESV04550 (0.005%) 1.8 0.25 - / -
ESV04566 (0.006%) 0.2 0.81 - / -
ESV04764 (0.008%) 2.1 0.20 - / -
ESV04972 (0.02%) 2.1 0.20 - / -
ESV05090 (0.009%) 14.7 0.005 ** - / -
ESV05284 (0.003%) 0.8 0.50 - / -
ESV05409 (0.007%) 0.8 0.50 - / -
ESV05504 (0.02%) 1.3 0.33 - / -
ESV05543 (0.003%) 1.1 0.39 - / -
ESV05696 (0.005%) 0.8 0.48 - / -
ESV05769 (0.01%) 0.3 0.77 - / -
ESV05892 (0.003%) 0.6 0.59 - / -
ESV05946 (0.02%) 1.9 0.24 - / -
ESV05980 (0.004%) 0.6 0.60 - / -
ESV06014 (0.002%) 0.7 0.52 - / -
ESV06121 (0.009%) 0.0 0.99 - / -
ESV06158 (0.005%) 0.6 0.59 - / -
ESV06221 (0.006%) 0.4 0.66 - / -
ESV06271 (0.004%) 1.5 0.29 - / -
ESV06371 (0.004%) 0.6 0.59 - / -
ESV06508 (0.01%) 1.0 0.43 - / -
ESV06559 (0.006%) 1.3 0.33 N / -
ESV07218 (0.003%) 2.5 0.17 - / -
ESV07331 (0.005%) 0.8 0.51 - / -
ESV07571 (0.01%) 0.3 0.75 - / -
ESV07579 (0.003%) 1.3 0.35 - / -
g:Schlesneria (0.03%) 8.1 0.02 * - / -
g:Schlesneria_uncl (0.03%) 8.1 0.02 * - / -
ESV06244 (0.01%) 2.1 0.20 - / -

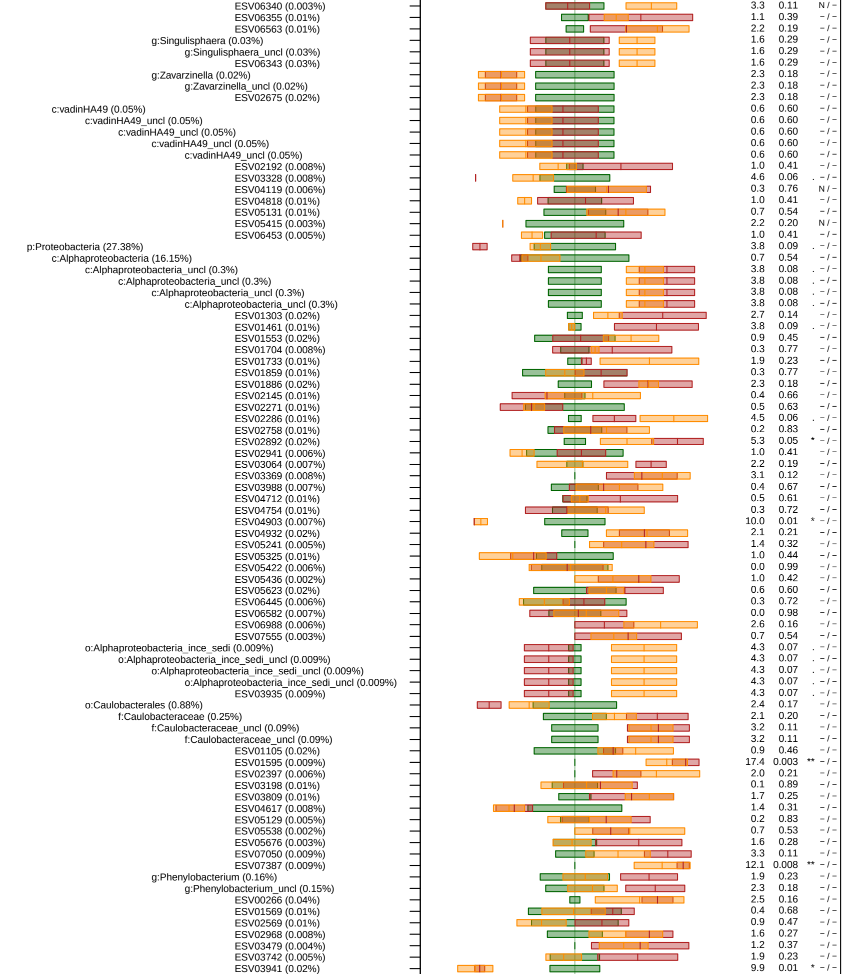
ESV06340 (0.003%) 3.3 0.11 N / –
ESV06355 (0.01%) 1.1 0.39 – / –
ESV06563 (0.01%) 2.2 0.19 – / –
g:Singulisphaera (0.03%) 1.6 0.29 – / –
g:Singulisphaera_uncl (0.03%) 1.6 0.29 – / –
ESV06343 (0.03%) 1.6 0.29 – / –
g:Zavarzinella (0.02%) 2.3 0.18 – / –
g:Zavarzinella_uncl (0.02%) 2.3 0.18 – / –
ESV02675 (0.02%) 2.3 0.18 – / –
c:vadinHA49 (0.05%) 0.6 0.60 – / –
c:vadinHA49_uncl (0.05%) 0.6 0.60 – / –
c:vadinHA49_uncl (0.05%) 0.6 0.60 – / –
c:vadinHA49_uncl (0.05%) 0.6 0.60 – / –
c:vadinHA49_uncl (0.05%) 0.6 0.60 – / –
ESV02192 (0.008%) 1.0 0.41 – / –
ESV03328 (0.008%) 4.6 0.06 . – / –
ESV04119 (0.006%) 0.3 0.76 N / –
ESV04818 (0.01%) 1.0 0.41 – / –
ESV05131 (0.01%) 0.7 0.54 – / –
ESV05415 (0.003%) 2.2 0.20 N / –
ESV06453 (0.005%) 1.0 0.41 – / –
p:Proteobacteria (27.38%) 3.8 0.09 . – / –
c:Alphaproteobacteria (16.15%) 0.7 0.54 – / –
c:Alphaproteobacteria_uncl (0.3%) 3.8 0.08 . – / –
c:Alphaproteobacteria_uncl (0.3%) 3.8 0.08 . – / –
c:Alphaproteobacteria_uncl (0.3%) 3.8 0.08 . – / –
c:Alphaproteobacteria_uncl (0.3%) 3.8 0.08 . – / –
ESV01303 (0.02%) 2.7 0.14 – / –
ESV01461 (0.01%) 3.8 0.09 . – / –
ESV01553 (0.02%) 0.9 0.45 – / –
ESV01704 (0.008%) 0.3 0.77 – / –
ESV01733 (0.01%) 1.9 0.23 – / –
ESV01859 (0.01%) 0.3 0.77 – / –
ESV01886 (0.02%) 2.3 0.18 – / –
ESV02145 (0.01%) 0.4 0.66 – / –
ESV02271 (0.01%) 0.5 0.63 – / –
ESV02286 (0.01%) 4.5 0.06 . – / –
ESV02758 (0.01%) 0.2 0.83 – / –
ESV02892 (0.02%) 5.3 0.05 * – / –
ESV02941 (0.006%) 1.0 0.41 – / –
ESV03064 (0.007%) 2.2 0.19 – / –
ESV03369 (0.008%) 3.1 0.12 – / –
ESV03988 (0.007%) 0.4 0.67 – / –
ESV04712 (0.01%) 0.5 0.61 – / –
ESV04754 (0.01%) 0.3 0.72 – / –
ESV04903 (0.007%) 10.0 0.01 * – / –
ESV04932 (0.02%) 2.1 0.21 – / –
ESV05241 (0.005%) 1.4 0.32 – / –
ESV05325 (0.01%) 1.0 0.44 – / –
ESV05422 (0.006%) 0.0 0.99 – / –
ESV05436 (0.002%) 1.0 0.42 – / –
ESV05623 (0.02%) 0.6 0.60 – / –
ESV06445 (0.006%) 0.3 0.72 – / –
ESV06582 (0.007%) 0.0 0.98 – / –
ESV06988 (0.006%) 2.6 0.16 – / –
ESV07555 (0.003%) 0.7 0.54 – / –
o:Alphaproteobacteria_ince_sedi (0.009%) 4.3 0.07 . – / –
o:Alphaproteobacteria_ince_sedi_uncl (0.009%) 4.3 0.07 . – / –
o:Alphaproteobacteria_ince_sedi_uncl (0.009%) 4.3 0.07 . – / –
o:Alphaproteobacteria_ince_sedi_uncl (0.009%) 4.3 0.07 . – / –
ESV03935 (0.009%) 4.3 0.07 . – / –
o:Caulobacterales (0.88%) 2.4 0.17 – / –
f:Caulobacteraceae (0.25%) 2.1 0.20 – / –
f:Caulobacteraceae_uncl (0.09%) 3.2 0.11 – / –
f:Caulobacteraceae_uncl (0.09%) 3.2 0.11 – / –
ESV01105 (0.02%) 0.9 0.46 – / –
ESV01595 (0.009%) 17.4 0.003 ** – / –
ESV02397 (0.006%) 2.0 0.21 – / –
ESV03198 (0.01%) 0.1 0.89 – / –
ESV03809 (0.01%) 1.7 0.25 – / –
ESV04617 (0.008%) 1.4 0.31 – / –
ESV05129 (0.005%) 0.2 0.83 – / –
ESV05538 (0.002%) 0.7 0.53 – / –
ESV05676 (0.003%) 1.6 0.28 – / –
ESV07050 (0.009%) 3.3 0.11 – / –
ESV07387 (0.009%) 12.1 0.008 ** – / –
g:Phenylobacterium (0.16%) 1.9 0.23 – / –
g:Phenylobacterium_uncl (0.15%) 2.3 0.18 – / –
ESV00266 (0.04%) 2.5 0.16 – / –
ESV01569 (0.01%) 0.4 0.68 – / –
ESV02569 (0.01%) 0.9 0.47 – / –
ESV02968 (0.008%) 1.6 0.27 – / –
ESV03479 (0.004%) 1.2 0.37 – / –
ESV03742 (0.005%) 1.9 0.23 – / –
ESV03941 (0.02%) 9.9 0.01 * – / –

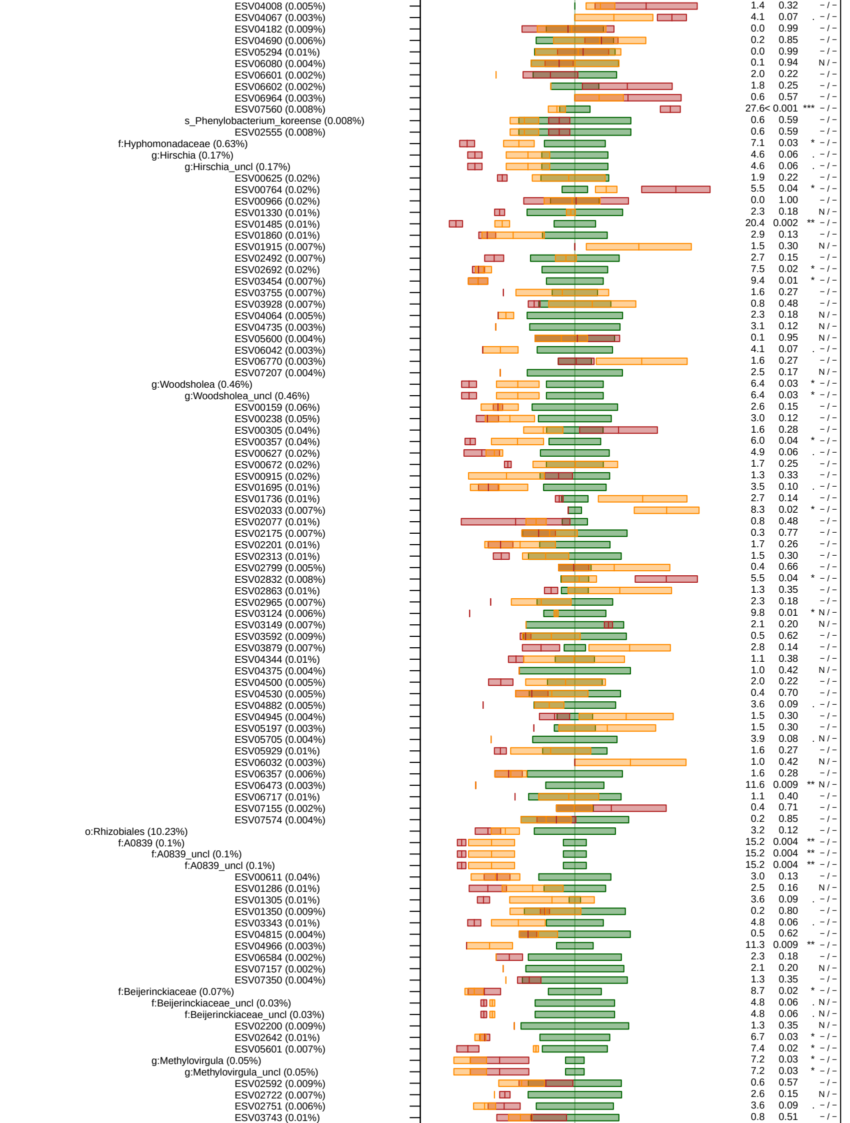

ESV04008 (0.005%) 1.4 0.32 – / –
ESV04067 (0.003%) 4.1 0.07 . – / –
ESV04182 (0.009%) 0.0 0.99 – / –
ESV04690 (0.006%) 0.2 0.85 – / –
ESV05294 (0.01%) 0.0 0.99 – / –
ESV06080 (0.004%) 0.1 0.94 N / –
ESV06601 (0.002%) 2.0 0.22 – / –
ESV06602 (0.002%) 1.8 0.25 – / –
ESV06964 (0.003%) 0.6 0.57 – / –
ESV07560 (0.008%) 27.6 < 0.001 *** – / –
s_Phenylobacterium_koreense (0.008%) 0.6 0.59 – / –
ESV02555 (0.008%) 0.6 0.59 – / –
f:Hyphomonadaceae (0.63%) 7.1 0.03 * – / –
g:Hirschia (0.17%) 4.6 0.06 . – / –
g:Hirschia_uncl (0.17%) 4.6 0.06 . – / –
ESV00625 (0.02%) 1.9 0.22 – / –
ESV00764 (0.02%) 5.5 0.04 * – / –
ESV00966 (0.02%) 0.0 1.00 – / –
ESV01330 (0.01%) 2.3 0.18 N / –
ESV01485 (0.01%) 20.4 0.002 ** – / –
ESV01860 (0.01%) 2.9 0.13 – / –
ESV01915 (0.007%) 1.5 0.30 N / –
ESV02492 (0.007%) 2.7 0.15 – / –
ESV02692 (0.02%) 7.5 0.02 * – / –
ESV03454 (0.007%) 9.4 0.01 * – / –
ESV03755 (0.007%) 1.6 0.27 – / –
ESV03928 (0.007%) 0.8 0.48 – / –
ESV04064 (0.005%) 2.3 0.18 N / –
ESV04735 (0.003%) 3.1 0.12 N / –
ESV05600 (0.004%) 0.1 0.95 N / –
ESV06042 (0.003%) 4.1 0.07 . – / –
ESV06770 (0.003%) 1.6 0.27 – / –
ESV07207 (0.004%) 2.5 0.17 N / –
g:Woodsholea (0.46%) 6.4 0.03 * – / –
g:Woodsholea_uncl (0.46%) 6.4 0.03 * – / –
ESV00159 (0.06%) 2.6 0.15 – / –
ESV00238 (0.05%) 3.0 0.12 – / –
ESV00305 (0.04%) 1.6 0.28 – / –
ESV00357 (0.04%) 6.0 0.04 * – / –
ESV00627 (0.02%) 4.9 0.06 . – / –
ESV00672 (0.02%) 1.7 0.25 – / –
ESV00915 (0.02%) 1.3 0.33 – / –
ESV01695 (0.01%) 3.5 0.10 . – / –
ESV01736 (0.01%) 2.7 0.14 – / –
ESV02033 (0.007%) 8.3 0.02 * – / –
ESV02077 (0.01%) 0.8 0.48 – / –
ESV02175 (0.007%) 0.3 0.77 – / –
ESV02201 (0.01%) 1.7 0.26 – / –
ESV02313 (0.01%) 1.5 0.30 – / –
ESV02799 (0.005%) 0.4 0.66 – / –
ESV02832 (0.008%) 5.5 0.04 * – / –
ESV02863 (0.01%) 1.3 0.35 – / –
ESV02965 (0.007%) 2.3 0.18 – / –
ESV03124 (0.006%) 9.8 0.01 * N / –
ESV03149 (0.007%) 2.1 0.20 N / –
ESV03592 (0.009%) 0.5 0.62 – / –
ESV03879 (0.007%) 2.8 0.14 – / –
ESV04344 (0.01%) 1.1 0.38 – / –
ESV04375 (0.004%) 1.0 0.42 N / –
ESV04500 (0.005%) 2.0 0.22 – / –
ESV04530 (0.005%) 0.4 0.70 – / –
ESV04882 (0.005%) 3.6 0.09 . – / –
ESV04945 (0.004%) 1.5 0.30 – / –
ESV05197 (0.003%) 1.5 0.30 – / –
ESV05705 (0.004%) 3.9 0.08 . N / –
ESV05929 (0.01%) 1.6 0.27 – / –
ESV06032 (0.003%) 1.0 0.42 N / –
ESV06357 (0.006%) 1.6 0.28 – / –
ESV06473 (0.003%) 11.6 0.009 ** N / –
ESV06717 (0.01%) 1.1 0.40 – / –
ESV07155 (0.002%) 0.4 0.71 – / –
ESV07574 (0.004%) 0.2 0.85 – / –
o:Rhizobiales (10.23%) 3.2 0.12 – / –
f:A0839 (0.1%) 15.2 0.004 ** – / –
f:A0839_uncl (0.1%) 15.2 0.004 ** – / –
f:A0839_uncl (0.1%) 15.2 0.004 ** – / –
ESV00611 (0.04%) 3.0 0.13 – / –
ESV01286 (0.01%) 2.5 0.16 N / –
ESV01305 (0.01%) 3.6 0.09 . – / –
ESV01350 (0.009%) 0.2 0.80 – / –
ESV03343 (0.01%) 4.8 0.06 . – / –
ESV04815 (0.004%) 0.5 0.62 – / –
ESV04966 (0.003%) 11.3 0.009 ** – / –
ESV06584 (0.002%) 2.3 0.18 – / –
ESV07157 (0.002%) 2.1 0.20 N / –
ESV07350 (0.004%) 1.3 0.35 – / –
f:Beijerinckiaceae (0.07%) 8.7 0.02 * – / –
f:Beijerinckiaceae_uncl (0.03%) 4.8 0.06 . N / –
f:Beijerinckiaceae_uncl (0.03%) 4.8 0.06 . N / –
ESV02200 (0.009%) 1.3 0.35 N / –
ESV02642 (0.01%) 6.7 0.03 * – / –
ESV05601 (0.007%) 7.4 0.02 * – / –
g:Methylovirgula (0.05%) 7.2 0.03 * – / –
g:Methylovirgula_uncl (0.05%) 7.2 0.03 * – / –
ESV02592 (0.009%) 0.6 0.57 – / –
ESV02722 (0.007%) 2.6 0.15 N / –
ESV02751 (0.006%) 3.6 0.09 . – / –
ESV03743 (0.01%) 0.8 0.51 – / –

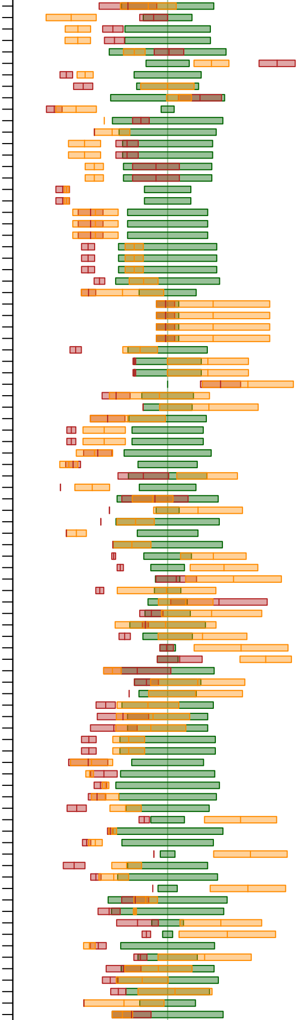

ESV05938 (0.004%)	0.3	0.74		- / -
ESV07277 (0.009%)	5.8	0.04	*	- / -
f:Bradyrhizobiaceae (1.07%)	2.9	0.13		- / -
g:Bradyrhizobium (1.06%)	2.9	0.13		- / -
g:Bradyrhizobium_uncl (0.33%)	0.3	0.75		- / -
ESV00031 (0.17%)	8.3	0.02	*	- / -
ESV00089 (0.08%)	7.1	0.03	*	- / -
ESV00466 (0.03%)	4.0	0.08	.	- / -
ESV00519 (0.03%)	0.2	0.82		- / -
ESV01291 (0.01%)	21.0	0.002	**	- / -
ESV01839 (0.008%)	1.0	0.43		- / -
ESV05451 (0.002%)	1.6	0.27		- / -
s_Bradyrhizobium_elkanii (0.64%)	2.2	0.20		- / -
ESV00001 (0.64%)	2.2	0.20		- / -
s_Bradyrhizobium_japonicum (0.007%)	1.8	0.24		- / -
ESV02371 (0.007%)	1.8	0.24		- / -
s_Bradyrhizobium_yuanmingense (0.07%)	18.0	0.003	**	- / -
ESV00127 (0.07%)	18.0	0.003	**	- / -
s_Afipia_genosp. (0.01%)	2.5	0.17		- / -
s_Afipia_genosp._uncl (0.01%)	2.5	0.17		- / -
ESV01136 (0.01%)	2.5	0.17		- / -
f:DUNssu044 (0.02%)	1.9	0.23		- / -
f:DUNssu044_uncl (0.02%)	1.9	0.23		- / -
f:DUNssu044_uncl (0.02%)	1.9	0.23		- / -
ESV01798 (0.02%)	1.2	0.36		- / -
ESV02384 (0.007%)	1.8	0.24		- / -
f:FFCH5858 (0.003%)	0.6	0.56		- / -
f:FFCH5858_uncl (0.003%)	0.6	0.56		- / -
f:FFCH5858_uncl (0.003%)	0.6	0.56		- / -
ESV04151 (0.003%)	0.6	0.56		- / -
f:Hyphomicrobiaceae (0.92%)	3.5	0.10	.	- / -
f:Hyphomicrobiaceae_uncl (0.03%)	1.5	0.30		- / -
f:Hyphomicrobiaceae_uncl (0.03%)	1.5	0.30		- / -
ESV01357 (0.008%)	2.0	0.21		- / -
ESV01583 (0.01%)	0.7	0.54		- / -
ESV03097 (0.003%)	1.1	0.39		- / -
ESV06078 (0.003%)	0.9	0.46		- / -
g:Devosia (0.08%)	4.1	0.07	.	- / -
g:Devosia_uncl (0.08%)	4.1	0.07	.	- / -
ESV01173 (0.02%)	2.1	0.20		- / -
ESV01298 (0.01%)	9.2	0.01	*	- / -
ESV02449 (0.01%)	1.0	0.42		- / -
ESV03547 (0.006%)	8.2	0.02	*	- / -
ESV03983 (0.009%)	0.0	0.95		- / -
ESV04184 (0.009%)	2.9	0.13		- / -
ESV05077 (0.006%)	1.1	0.39		- / -
ESV05238 (0.005%)	9.1	0.02	*	- / -
ESV06036 (0.002%)	0.7	0.54		- / -
g:Hyphomicrobium (0.09%)	4.5	0.06	.	- / -
g:Hyphomicrobium_uncl (0.07%)	5.6	0.04	*	- / -
ESV03299 (0.006%)	1.3	0.33		- / -
ESV03387 (0.01%)	1.7	0.26		- / -
ESV03534 (0.006%)	0.6	0.58		- / -
ESV03655 (0.005%)	0.9	0.45		- / -
ESV04154 (0.006%)	0.1	0.89		- / -
ESV04493 (0.009%)	1.7	0.25		- / -
ESV04501 (0.006%)	2.4	0.17		- / -
ESV04929 (0.006%)	6.7	0.03	*	- / -
ESV05646 (0.006%)	0.7	0.54		- / -
ESV06223 (0.004%)	0.6	0.59		- / -
ESV07466 (0.007%)	1.1	0.39		- / -
s_Hyphomicrobium_facile (0.02%)	1.0	0.44		- / -
ESV02663 (0.01%)	0.4	0.67		- / -
ESV03273 (0.007%)	0.7	0.54		- / -
g:Pedomicrobium (0.48%)	1.8	0.24		- / -
g:Pedomicrobium_uncl (0.48%)	1.8	0.24		- / -
ESV00095 (0.08%)	2.9	0.13		- / -
ESV00219 (0.04%)	2.1	0.20		- / -
ESV00224 (0.04%)	1.5	0.29		- / -
ESV00453 (0.03%)	1.7	0.26		- / -
ESV00486 (0.03%)	3.0	0.12		- / -
ESV00703 (0.02%)	4.7	0.06	.	- / -
ESV00859 (0.02%)	1.0	0.41		N / -
ESV01071 (0.02%)	2.8	0.14		- / -
ESV01168 (0.02%)	5.8	0.04	*	- / -
ESV01275 (0.02%)	3.4	0.10		- / -
ESV01542 (0.02%)	1.5	0.29		- / -
ESV01568 (0.01%)	5.2	0.05	*	- / -
ESV01657 (0.01%)	0.2	0.81		- / -
ESV01717 (0.008%)	0.8	0.50		- / -
ESV01741 (0.01%)	2.3	0.18		- / -
ESV01770 (0.01%)	2.2	0.19		- / -
ESV01928 (0.008%)	2.4	0.17		- / -
ESV02045 (0.01%)	1.0	0.42		- / -
ESV02446 (0.009%)	0.5	0.66		- / -
ESV02750 (0.005%)	0.8	0.51		- / -
ESV03443 (0.008%)	0.9	0.46		- / -
ESV04851 (0.004%)	2.2	0.19		- / -
ESV05159 (0.004%)	0.5	0.64		- / -

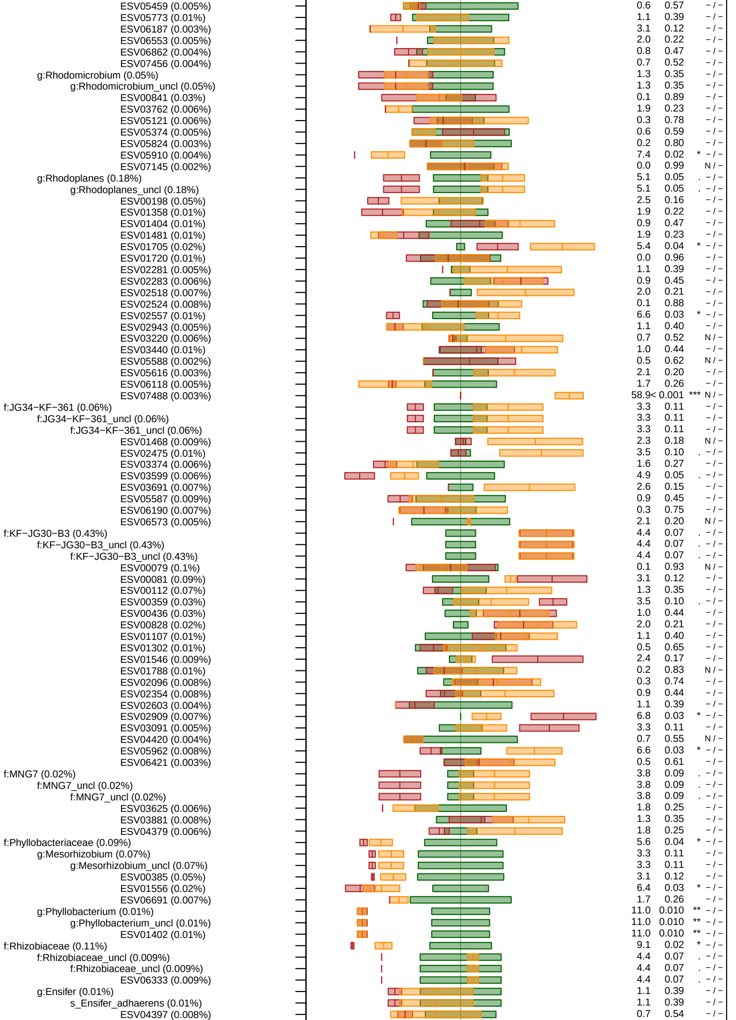
ESV05459 (0.005%) 0.6 0.57 − / −
ESV05773 (0.01%) 1.1 0.39 − / −
ESV06187 (0.003%) 3.1 0.12 − / −
ESV06553 (0.005%) 2.0 0.22 − / −
ESV06862 (0.004%) 0.8 0.47 − / −
ESV07456 (0.004%) 0.7 0.52 − / −
g:Rhodomicrobium (0.05%) 1.3 0.35 − / −
g:Rhodomicrobium_uncl (0.05%) 1.3 0.35 − / −
ESV00841 (0.03%) 0.1 0.89 − / −
ESV03762 (0.006%) 1.9 0.23 − / −
ESV05121 (0.006%) 0.3 0.78 − / −
ESV05374 (0.005%) 0.6 0.59 − / −
ESV05824 (0.003%) 0.2 0.80 − / −
ESV05910 (0.004%) 7.4 0.02 * − / −
ESV07145 (0.002%) 0.0 0.99 N / −
g:Rhodoplanes (0.18%) 5.1 0.05 . − / −
g:Rhodoplanes_uncl (0.18%) 5.1 0.05 . − / −
ESV00198 (0.05%) 2.5 0.16 − / −
ESV01358 (0.01%) 1.9 0.22 − / −
ESV01404 (0.01%) 0.9 0.47 − / −
ESV01481 (0.01%) 1.9 0.23 − / −
ESV01705 (0.02%) 5.4 0.04 * − / −
ESV01720 (0.01%) 0.0 0.96 − / −
ESV02281 (0.005%) 1.1 0.39 − / −
ESV02283 (0.006%) 0.9 0.45 − / −
ESV02518 (0.007%) 2.0 0.21 − / −
ESV02524 (0.008%) 0.1 0.88 − / −
ESV02557 (0.01%) 6.6 0.03 * − / −
ESV02943 (0.005%) 1.1 0.40 − / −
ESV03220 (0.006%) 0.7 0.52 N / −
ESV03440 (0.01%) 1.0 0.44 − / −
ESV05588 (0.002%) 0.5 0.62 N / −
ESV05616 (0.003%) 2.1 0.20 − / −
ESV06118 (0.005%) 1.7 0.26 − / −
ESV07488 (0.003%) 58.9 < 0.001 *** N / −
f:JG34−KF−361 (0.06%) 3.3 0.11 − / −
f:JG34−KF−361_uncl (0.06%) 3.3 0.11 − / −
f:JG34−KF−361_uncl (0.06%) 3.3 0.11 − / −
ESV01468 (0.009%) 2.3 0.18 N / −
ESV02475 (0.01%) 3.5 0.10 . − / −
ESV03374 (0.006%) 1.6 0.27 − / −
ESV03599 (0.006%) 4.9 0.05 . − / −
ESV03691 (0.007%) 2.6 0.15 − / −
ESV05587 (0.009%) 0.9 0.45 − / −
ESV06190 (0.007%) 0.3 0.75 − / −
ESV06573 (0.005%) 2.1 0.20 N / −
f:KF−JG30−B3 (0.43%) 4.4 0.07 . − / −
f:KF−JG30−B3_uncl (0.43%) 4.4 0.07 . − / −
f:KF−JG30−B3_uncl (0.43%) 4.4 0.07 . − / −
ESV00079 (0.1%) 0.1 0.93 N / −
ESV00081 (0.09%) 3.1 0.12 − / −
ESV00112 (0.07%) 1.3 0.35 − / −
ESV00359 (0.03%) 3.5 0.10 . − / −
ESV00436 (0.03%) 1.0 0.44 − / −
ESV00828 (0.02%) 2.0 0.21 − / −
ESV01107 (0.01%) 1.1 0.40 − / −
ESV01302 (0.01%) 0.5 0.65 − / −
ESV01546 (0.009%) 2.4 0.17 − / −
ESV01788 (0.01%) 0.2 0.83 N / −
ESV02096 (0.008%) 0.3 0.74 − / −
ESV02354 (0.008%) 0.9 0.44 − / −
ESV02603 (0.004%) 1.1 0.39 − / −
ESV02909 (0.007%) 6.8 0.03 * − / −
ESV03091 (0.005%) 3.3 0.11 − / −
ESV04420 (0.004%) 0.7 0.55 N / −
ESV05962 (0.008%) 6.6 0.03 * − / −
ESV06421 (0.003%) 0.5 0.61 − / −
f:MNG7 (0.02%) 3.8 0.09 . − / −
f:MNG7_uncl (0.02%) 3.8 0.09 . − / −
f:MNG7_uncl (0.02%) 3.8 0.09 . − / −
ESV03625 (0.006%) 1.8 0.25 − / −
ESV03881 (0.008%) 1.3 0.35 − / −
ESV04379 (0.006%) 1.8 0.25 − / −
f:Phyllobacteriaceae (0.09%) 5.6 0.04 * − / −
g:Mesorhizobium (0.07%) 3.3 0.11 − / −
g:Mesorhizobium_uncl (0.07%) 3.3 0.11 − / −
ESV00385 (0.05%) 3.1 0.12 − / −
ESV01556 (0.02%) 6.4 0.03 * − / −
ESV06691 (0.007%) 1.7 0.26 − / −
g:Phyllobacterium (0.01%) 11.0 0.010 ** − / −
g:Phyllobacterium_uncl (0.01%) 11.0 0.010 ** − / −
ESV01402 (0.01%) 11.0 0.010 ** − / −
f:Rhizobiaceae (0.11%) 9.1 0.02 * − / −
f:Rhizobiaceae_uncl (0.009%) 4.4 0.07 . − / −
f:Rhizobiaceae_uncl (0.009%) 4.4 0.07 . − / −
ESV06333 (0.009%) 4.4 0.07 . − / −
g:Ensifer (0.01%) 1.1 0.39 − / −
s_Ensifer_adhaerens (0.01%) 1.1 0.39 − / −
ESV04397 (0.008%) 0.7 0.54 − / −

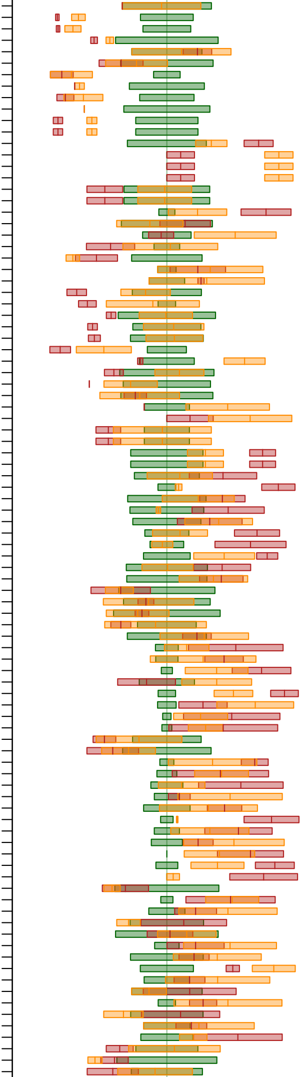

ESV06556 (0.002%)	0.5	0.63		N / –
g:Rhizobium (0.09%)	13.6	0.006	**	– / –
g:Rhizobium_uncl (0.07%)	16.3	0.004	**	– / –
ESV00982 (0.01%)	1.7	0.27		N / –
ESV02105 (0.009%)	0.2	0.84		– / –
ESV03108 (0.007%)	0.5	0.65		– / –
ESV03754 (0.006%)	13.5	0.006	**	N / –
ESV04291 (0.008%)	5.6	0.04	*	N / –
ESV05323 (0.02%)	7.4	0.02	*	– / –
ESV06461 (0.003%)	3.7	0.09	.	N / –
s_Rhizobium_leguminosarum (0.02%)	9.2	0.02	*	– / –
ESV00513 (0.02%)	9.2	0.02	*	– / –
f:Rhizobiales_ince_sedi (1.92%)	3.1	0.12		– / –
f:Rhizobiales_ince_sedi_uncl (0.003%)	28.6	< 0.001	***	N / –
f:Rhizobiales_ince_sedi_uncl (0.003%)	28.6	< 0.001	***	N / –
ESV06740 (0.003%)	28.6	< 0.001	***	N / –
g:Bauldia (0.1%)	1.3	0.34		– / –
g:Bauldia_uncl (0.1%)	1.3	0.34		– / –
ESV00937 (0.01%)	5.0	0.05	.	– / –
ESV01721 (0.02%)	0.2	0.79		– / –
ESV02091 (0.009%)	2.1	0.20		– / –
ESV02652 (0.01%)	1.1	0.38		– / –
ESV02727 (0.009%)	4.1	0.07	.	– / –
ESV03523 (0.005%)	0.4	0.68		– / –
ESV05305 (0.003%)	0.4	0.70		– / –
ESV06138 (0.008%)	3.5	0.10	.	– / –
ESV06843 (0.009%)	2.3	0.18		– / –
ESV07126 (0.01%)	1.0	0.43		– / –
g:Nordella (0.09%)	2.9	0.13		– / –
g:Nordella_uncl (0.09%)	2.7	0.15		– / –
ESV00467 (0.03%)	6.9	0.03	*	– / –
ESV00760 (0.02%)	7.5	0.02	*	– / –
ESV01694 (0.01%)	1.2	0.35		– / –
ESV01819 (0.01%)	1.7	0.26		– / –
ESV02394 (0.01%)	0.2	0.80		– / –
ESV04849 (0.002%)	2.5	0.16		– / –
ESV06405 (0.002%)	2.4	0.17		– / –
s_Nordella_oligomobilis (0.005%)	1.0	0.41		– / –
ESV02955 (0.005%)	1.0	0.41		– / –
g:Rhizomicrobium (1.73%)	3.9	0.08	.	– / –
g:Rhizomicrobium_uncl (1.73%)	3.9	0.08	.	– / –
ESV00009 (0.25%)	0.8	0.49		– / –
ESV00041 (0.15%)	30.7	< 0.001	***	– / –
ESV00057 (0.1%)	0.7	0.54		– / –
ESV00094 (0.08%)	1.6	0.27		– / –
ESV00176 (0.06%)	0.7	0.54		N / –
ESV00190 (0.06%)	4.1	0.08	.	– / –
ESV00214 (0.04%)	4.5	0.06	.	– / –
ESV00225 (0.06%)	4.8	0.06	.	– / –
ESV00348 (0.04%)	1.0	0.43		– / –
ESV00377 (0.03%)	0.8	0.49		– / –
ESV00424 (0.03%)	0.6	0.56		– / –
ESV00538 (0.03%)	0.1	0.91		– / –
ESV00612 (0.02%)	0.2	0.83		– / –
ESV00689 (0.02%)	0.5	0.64		– / –
ESV00692 (0.02%)	0.3	0.74		– / –
ESV00730 (0.02%)	1.3	0.33		– / –
ESV00737 (0.02%)	0.8	0.51		– / –
ESV00739 (0.02%)	3.6	0.09	.	– / –
ESV00743 (0.02%)	1.4	0.32		– / –
ESV00751 (0.02%)	16.3	0.004	**	– / –
ESV00831 (0.01%)	2.8	0.14		– / –
ESV00871 (0.01%)	1.0	0.41		– / –
ESV00913 (0.01%)	0.8	0.51		– / –
ESV01011 (0.01%)	0.9	0.44		– / –
ESV01068 (0.02%)	0.7	0.54		– / –
ESV01077 (0.02%)	2.6	0.15		– / –
ESV01147 (0.01%)	0.9	0.44		– / –
ESV01222 (0.01%)	1.8	0.25		– / –
ESV01287 (0.02%)	1.1	0.39		– / –
ESV01341 (0.01%)	0.8	0.48		– / –
ESV01406 (0.02%)	12.6	0.007	**	– / –
ESV01474 (0.01%)	1.4	0.32		– / –
ESV01549 (0.01%)	1.1	0.38		– / –
ESV01576 (0.009%)	2.3	0.18		– / –
ESV01628 (0.009%)	7.3	0.02	*	– / –
ESV01803 (0.007%)	7.4	0.02	*	– / –
ESV01818 (0.01%)	0.7	0.52		– / –
ESV01832 (0.01%)	1.5	0.29		– / –
ESV01878 (0.01%)	0.9	0.46		– / –
ESV01951 (0.01%)	0.7	0.52		– / –
ESV02233 (0.009%)	0.1	0.92		– / –
ESV02240 (0.01%)	1.0	0.43		– / –
ESV02294 (0.01%)	0.5	0.60		– / –
ESV02595 (0.007%)	7.2	0.03	*	– / –
ESV02606 (0.008%)	0.6	0.60		– / –
ESV02676 (0.006%)	0.3	0.73		– / –
ESV02805 (0.008%)	0.9	0.46		– / –
ESV02862 (0.007%)	0.8	0.48		– / –
ESV02932 (0.005%)	0.2	0.81		– / –
ESV02979 (0.01%)	1.4	0.32		– / –
ESV03039 (0.004%)	0.8	0.49		– / –
ESV03142 (0.007%)	1.5	0.29		– / –
ESV03207 (0.01%)	0.7	0.52		– / –

ESV03230 (0.008%)	0.7	0.52		- / -
ESV03344 (0.005%)	1.1	0.39		- / -
ESV03477 (0.007%)	1.1	0.40		- / -
ESV03629 (0.01%)	0.2	0.83		- / -
ESV03649 (0.005%)	1.2	0.36		- / -
ESV03662 (0.009%)	1.3	0.34		- / -
ESV03699 (0.007%)	0.9	0.46		- / -
ESV03733 (0.004%)	0.3	0.73		- / -
ESV03781 (0.01%)	0.8	0.49		- / -
ESV03805 (0.007%)	1.9	0.23		- / -
ESV03907 (0.005%)	3.9	0.08	.	- / -
ESV04026 (0.005%)	1.4	0.32		- / -
ESV04123 (0.02%)	0.1	0.91		- / -
ESV04145 (0.01%)	1.2	0.36		- / -
ESV04358 (0.01%)	1.3	0.34		- / -
ESV04431 (0.007%)	1.3	0.34		- / -
ESV04784 (0.002%)	0.8	0.47		- / -
ESV04859 (0.007%)	1.1	0.40		- / -
ESV04899 (0.003%)	2.0	0.21		- / -
ESV04960 (0.01%)	1.0	0.42		- / -
ESV04994 (0.004%)	0.4	0.71		- / -
ESV05076 (0.005%)	1.3	0.34		- / -
ESV05170 (0.007%)	0.8	0.51		- / -
ESV05248 (0.005%)	1.0	0.43		- / -
ESV05299 (0.003%)	0.9	0.47		- / -
ESV05335 (0.009%)	1.0	0.42		N / -
ESV05380 (0.003%)	1.8	0.25		- / -
ESV05391 (0.002%)	1.6	0.27		- / -
ESV05533 (0.004%)	2.2	0.20		- / -
ESV05575 (0.004%)	1.0	0.42		N / -
ESV05612 (0.006%)	0.9	0.46		- / -
ESV05615 (0.007%)	1.3	0.35		- / -
ESV05942 (0.005%)	0.8	0.50		- / -
ESV05952 (0.004%)	3.7	0.09	.	- / -
ESV05982 (0.004%)	0.8	0.49		- / -
ESV06275 (0.004%)	0.4	0.71		- / -
ESV06305 (0.002%)	0.8	0.49		- / -
ESV06334 (0.003%)	1.7	0.26		- / -
ESV06515 (0.005%)	1.6	0.28		- / -
ESV06670 (0.007%)	0.6	0.58		- / -
ESV06714 (0.002%)	0.5	0.63		N / -
ESV06941 (0.003%)	2.6	0.16		N / -
ESV06965 (0.003%)	2.0	0.22		- / -
ESV07272 (0.004%)	5.1	0.05	.	- / -
ESV07459 (0.005%)	4.6	0.06	.	- / -
ESV07481 (0.003%)	1.0	0.42		N / -
f:Rhodobiaceae (0.13%)	4.5	0.06	.	- / -
f:Rhodobiaceae_uncl (0.13%)	4.5	0.06	.	- / -
f:Rhodobiaceae_uncl (0.13%)	4.5	0.06	.	- / -
ESV00179 (0.06%)	1.3	0.33		- / -
ESV00382 (0.04%)	0.8	0.49		- / -
ESV00620 (0.02%)	3.6	0.09	.	- / -
ESV02962 (0.004%)	0.9	0.46		- / -
ESV04406 (0.006%)	2.3	0.18		- / -
ESV05198 (0.004%)	1.6	0.28		- / -
f:Roseiarcaceae (0.06%)	3.5	0.10	.	- / -
g:Roseiarcus (0.06%)	3.5	0.10	.	- / -
g:Roseiarcus_uncl (0.06%)	3.5	0.10	.	- / -
ESV01009 (0.01%)	1.2	0.37		- / -
ESV01458 (0.02%)	0.4	0.68		- / -
ESV02243 (0.01%)	4.5	0.06	.	- / -
ESV05655 (0.02%)	2.8	0.14		- / -
ESV06604 (0.003%)	1.2	0.38		- / -
f:Xanthobacteraceae (4.43%)	7.3	0.02	*	- / -
f:Xanthobacteraceae_uncl (1.16%)	3.5	0.10	.	- / -
f:Xanthobacteraceae_uncl (1.16%)	3.5	0.10	.	- / -
ESV00003 (0.45%)	19.1	0.002	**	- / -
ESV00019 (0.19%)	1.9	0.23		- / -
ESV00072 (0.1%)	2.0	0.21		- / -
ESV00102 (0.07%)	0.6	0.57		- / -
ESV00130 (0.06%)	1.5	0.29		- / -
ESV00145 (0.05%)	1.1	0.39		- / -
ESV00294 (0.05%)	0.4	0.66		- / -
ESV00410 (0.03%)	0.7	0.52		- / -
ESV00484 (0.02%)	1.1	0.39		N / -
ESV00500 (0.03%)	1.1	0.38		- / -
ESV00791 (0.02%)	0.3	0.75		- / -
ESV00826 (0.02%)	0.9	0.44		- / -
ESV00964 (0.02%)	8.6	0.02	*	- / -
ESV01005 (0.02%)	3.0	0.12		- / -
ESV02111 (0.01%)	0.2	0.81		- / -
ESV02463 (0.007%)	0.4	0.70		- / -
ESV04286 (0.003%)	0.7	0.54		- / -
ESV05518 (0.004%)	1.2	0.37		- / -
ESV07022 (0.004%)	0.9	0.46		- / -
g:Labrys (0.03%)	7.0	0.03	*	- / -
g:Labrys_uncl (0.02%)	7.8	0.02	*	- / -
ESV05049 (0.004%)	2.6	0.15		N / -
ESV06159 (0.003%)	6.2	0.03	*	- / -
ESV07199 (0.003%)	1.8	0.25		- / -
ESV07230 (0.005%)	4.1	0.07	.	- / -
s_Labrys_miyagiensis (0.008%)	0.5	0.63		- / -
ESV05171 (0.008%)	0.5	0.63		- / -
s_Labrys_wisconsinensis (0.01%)	3.1	0.12		- / -
ESV02455 (0.01%)	3.1	0.12		- / -
g:Pseudolabrys (0.17%)	3.7	0.09	.	- / -
g:Pseudolabrys_uncl (0.17%)	3.7	0.09	.	- / -
ESV00203 (0.06%)	3.1	0.12		N / -
ESV00228 (0.04%)	11.6	0.009	**	- / -
ESV00950 (0.02%)	3.9	0.08	.	- / -

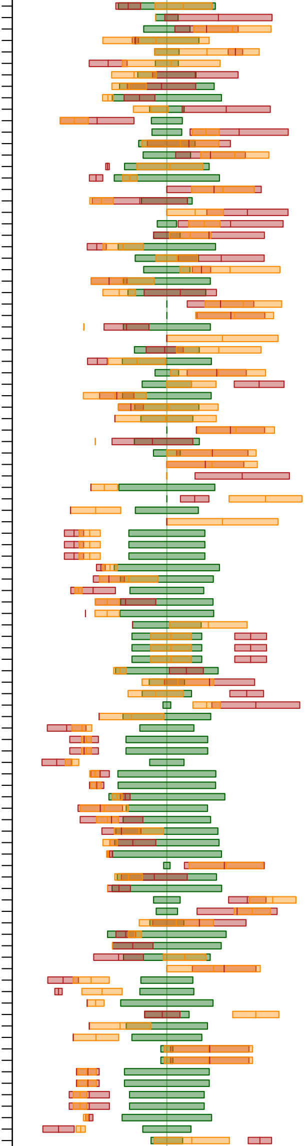

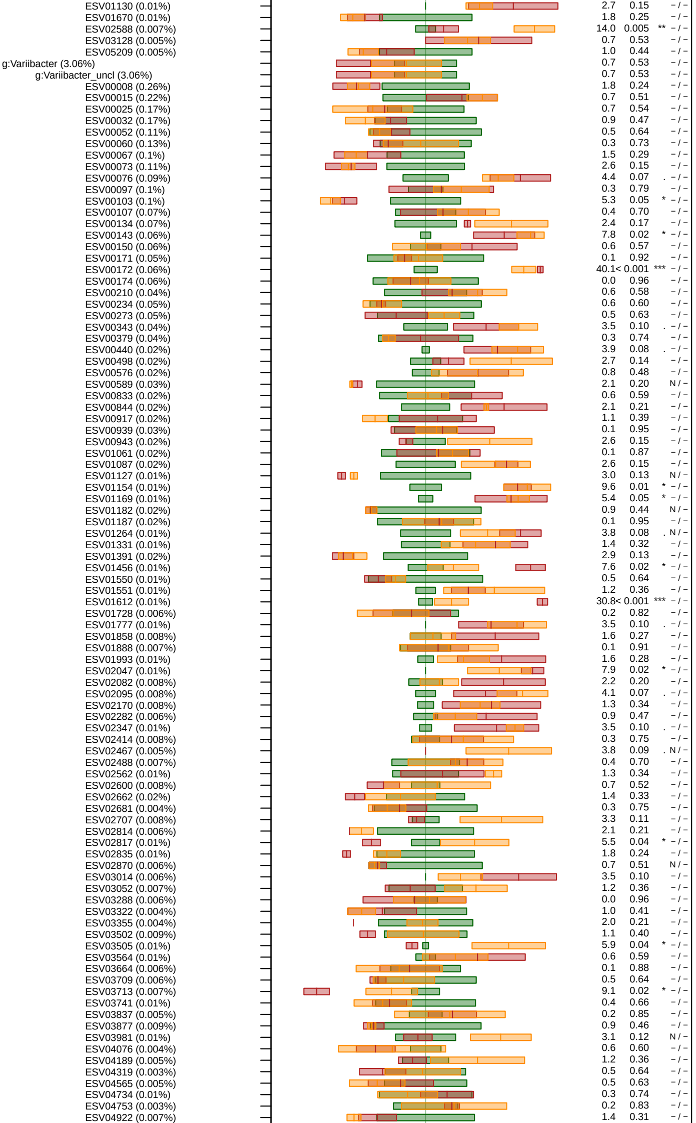

ESV01130 (0.01%) 2.7 0.15 – / –
ESV01670 (0.01%) 1.8 0.25 – / –
ESV02588 (0.007%) 14.0 0.005 ** – / –
ESV03128 (0.005%) 0.7 0.53 – / –
ESV05209 (0.005%) 1.0 0.44 – / –
g:Variibacter (3.06%) 0.7 0.53 – / –
g:Variibacter_uncl (3.06%) 0.7 0.53 – / –
ESV00008 (0.26%) 1.8 0.24 – / –
ESV00015 (0.22%) 0.7 0.51 – / –
ESV00025 (0.17%) 0.7 0.54 – / –
ESV00032 (0.17%) 0.9 0.47 – / –
ESV00052 (0.11%) 0.5 0.64 – / –
ESV00060 (0.13%) 0.3 0.73 – / –
ESV00067 (0.1%) 1.5 0.29 – / –
ESV00073 (0.11%) 2.6 0.15 – / –
ESV00076 (0.09%) 4.4 0.07 . – / –
ESV00097 (0.1%) 0.3 0.79 – / –
ESV00103 (0.1%) 5.3 0.05 * – / –
ESV00107 (0.07%) 0.4 0.70 – / –
ESV00134 (0.07%) 2.4 0.17 – / –
ESV00143 (0.06%) 7.8 0.02 * – / –
ESV00150 (0.06%) 0.6 0.57 – / –
ESV00171 (0.05%) 0.1 0.92 – / –
ESV00172 (0.06%) 40.1 < 0.001 *** – / –
ESV00174 (0.06%) 0.0 0.96 – / –
ESV00210 (0.04%) 0.6 0.58 – / –
ESV00234 (0.05%) 0.6 0.60 – / –
ESV00273 (0.05%) 0.5 0.63 – / –
ESV00343 (0.04%) 3.5 0.10 . – / –
ESV00379 (0.04%) 0.3 0.74 – / –
ESV00440 (0.02%) 3.9 0.08 . – / –
ESV00498 (0.02%) 2.7 0.14 – / –
ESV00576 (0.02%) 0.8 0.48 – / –
ESV00589 (0.03%) 2.1 0.20 N / –
ESV00833 (0.02%) 0.6 0.59 – / –
ESV00844 (0.02%) 2.1 0.21 – / –
ESV00917 (0.02%) 1.1 0.39 – / –
ESV00939 (0.03%) 0.1 0.95 – / –
ESV00943 (0.02%) 2.6 0.15 – / –
ESV01061 (0.02%) 0.1 0.87 – / –
ESV01087 (0.02%) 2.6 0.15 – / –
ESV01127 (0.01%) 3.0 0.13 N / –
ESV01154 (0.01%) 9.6 0.01 * – / –
ESV01169 (0.01%) 5.4 0.05 * – / –
ESV01182 (0.02%) 0.9 0.44 N / –
ESV01187 (0.02%) 0.1 0.95 – / –
ESV01264 (0.01%) 3.8 0.08 . N / –
ESV01331 (0.01%) 1.4 0.32 – / –
ESV01391 (0.02%) 2.9 0.13 – / –
ESV01456 (0.01%) 7.6 0.02 * – / –
ESV01550 (0.01%) 0.5 0.64 – / –
ESV01551 (0.01%) 1.2 0.36 – / –
ESV01612 (0.01%) 30.8 < 0.001 *** – / –
ESV01728 (0.006%) 0.2 0.82 – / –
ESV01777 (0.01%) 3.5 0.10 . – / –
ESV01858 (0.008%) 1.6 0.27 – / –
ESV01888 (0.007%) 0.1 0.91 – / –
ESV01993 (0.01%) 1.6 0.28 – / –
ESV02047 (0.01%) 7.9 0.02 * – / –
ESV02082 (0.008%) 2.2 0.20 – / –
ESV02095 (0.008%) 4.1 0.07 . – / –
ESV02170 (0.008%) 1.3 0.34 – / –
ESV02282 (0.006%) 0.9 0.47 – / –
ESV02347 (0.01%) 3.5 0.10 . – / –
ESV02414 (0.008%) 0.3 0.75 – / –
ESV02467 (0.005%) 3.8 0.09 . N / –
ESV02488 (0.007%) 0.4 0.70 – / –
ESV02562 (0.01%) 1.3 0.34 – / –
ESV02600 (0.008%) 0.7 0.52 – / –
ESV02662 (0.02%) 1.4 0.33 – / –
ESV02681 (0.004%) 0.3 0.75 – / –
ESV02707 (0.008%) 3.3 0.11 – / –
ESV02814 (0.006%) 2.1 0.21 – / –
ESV02817 (0.01%) 5.5 0.04 * – / –
ESV02835 (0.01%) 1.8 0.24 – / –
ESV02870 (0.006%) 0.7 0.51 N / –
ESV03014 (0.006%) 3.5 0.10 – / –
ESV03052 (0.007%) 1.2 0.36 – / –
ESV03288 (0.006%) 0.0 0.96 – / –
ESV03322 (0.004%) 1.0 0.41 – / –
ESV03355 (0.004%) 2.0 0.21 – / –
ESV03502 (0.009%) 1.1 0.40 – / –
ESV03505 (0.01%) 5.9 0.04 * – / –
ESV03564 (0.01%) 0.6 0.59 – / –
ESV03664 (0.006%) 0.1 0.88 – / –
ESV03709 (0.006%) 0.5 0.64 – / –
ESV03713 (0.007%) 9.1 0.02 * – / –
ESV03741 (0.01%) 0.4 0.66 – / –
ESV03837 (0.005%) 0.2 0.85 – / –
ESV03877 (0.009%) 0.9 0.46 – / –
ESV03981 (0.01%) 3.1 0.12 N / –
ESV04076 (0.004%) 0.6 0.60 – / –
ESV04189 (0.005%) 1.2 0.36 – / –
ESV04319 (0.003%) 0.5 0.64 – / –
ESV04565 (0.005%) 0.5 0.63 – / –
ESV04734 (0.01%) 0.3 0.74 – / –
ESV04753 (0.003%) 0.2 0.83 – / –
ESV04922 (0.007%) 1.4 0.31 – / –

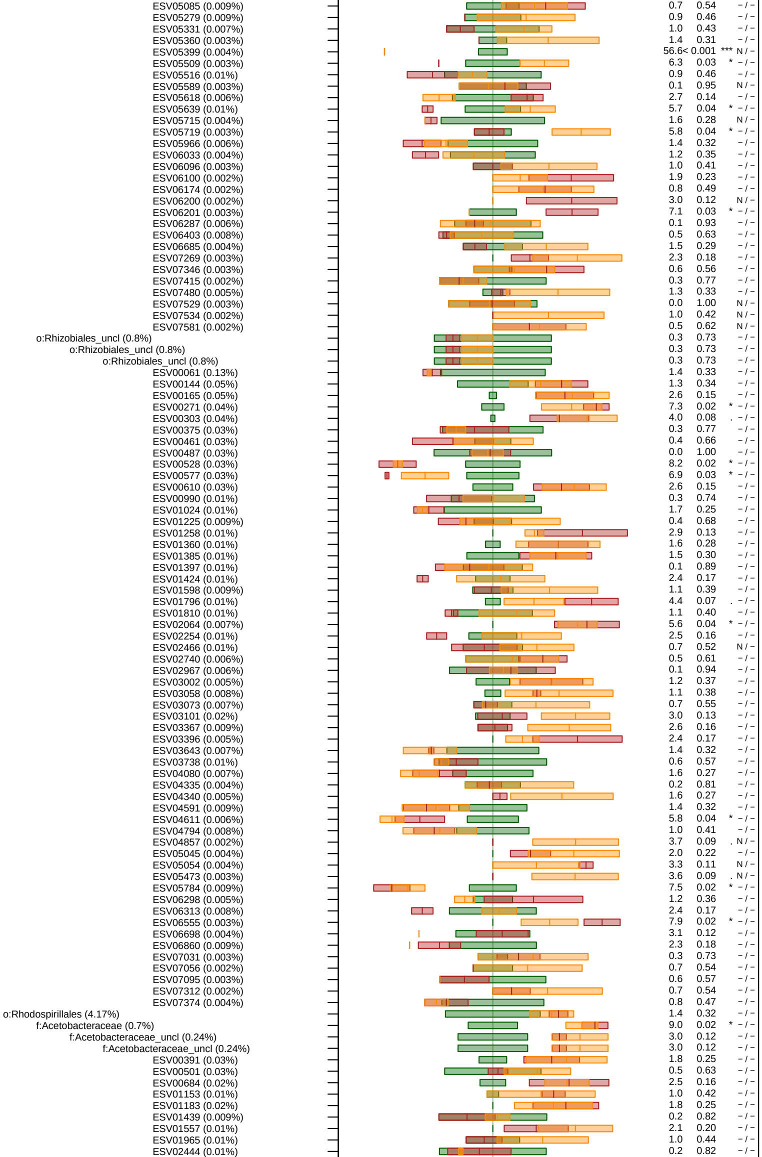

ESV05085 (0.009%) 0.7 0.54 – / –
ESV05279 (0.009%) 0.9 0.46 – / –
ESV05331 (0.007%) 1.0 0.43 – / –
ESV05360 (0.003%) 1.4 0.31 – / –
ESV05399 (0.004%) 56.6 < 0.001 *** N / –
ESV05509 (0.003%) 6.3 0.03 * – / –
ESV05516 (0.01%) 0.9 0.46 – / –
ESV05589 (0.003%) 0.1 0.95 N / –
ESV05618 (0.006%) 2.7 0.14 – / –
ESV05639 (0.01%) 5.7 0.04 * – / –
ESV05715 (0.004%) 1.6 0.28 N / –
ESV05719 (0.003%) 5.8 0.04 * – / –
ESV05966 (0.006%) 1.4 0.32 – / –
ESV06033 (0.004%) 1.2 0.35 – / –
ESV06096 (0.003%) 1.0 0.41 – / –
ESV06100 (0.002%) 1.9 0.23 – / –
ESV06174 (0.002%) 0.8 0.49 – / –
ESV06200 (0.002%) 3.0 0.12 N / –
ESV06201 (0.003%) 7.1 0.03 * – / –
ESV06287 (0.006%) 0.1 0.93 – / –
ESV06403 (0.008%) 0.5 0.63 – / –
ESV06685 (0.004%) 1.5 0.29 – / –
ESV07269 (0.003%) 2.3 0.18 – / –
ESV07346 (0.003%) 0.6 0.56 – / –
ESV07415 (0.002%) 0.3 0.77 – / –
ESV07480 (0.005%) 1.3 0.33 – / –
ESV07529 (0.003%) 0.0 1.00 N / –
ESV07534 (0.002%) 1.0 0.42 N / –
ESV07581 (0.002%) 0.5 0.62 N / –
o:Rhizobiales_uncl (0.8%) 0.3 0.73 – / –
o:Rhizobiales_uncl (0.8%) 0.3 0.73 – / –
o:Rhizobiales_uncl (0.8%) 0.3 0.73 – / –
ESV00061 (0.13%) 1.4 0.33 – / –
ESV00144 (0.05%) 1.3 0.34 – / –
ESV00165 (0.05%) 2.6 0.15 – / –
ESV00271 (0.04%) 7.3 0.02 * – / –
ESV00303 (0.04%) 4.0 0.08 . – / –
ESV00375 (0.03%) 0.3 0.77 – / –
ESV00461 (0.03%) 0.4 0.66 – / –
ESV00487 (0.03%) 0.0 1.00 – / –
ESV00528 (0.03%) 8.2 0.02 * – / –
ESV00577 (0.03%) 6.9 0.03 * – / –
ESV00610 (0.03%) 2.6 0.15 – / –
ESV00990 (0.01%) 0.3 0.74 – / –
ESV01024 (0.01%) 1.7 0.25 – / –
ESV01225 (0.009%) 0.4 0.68 – / –
ESV01258 (0.01%) 2.9 0.13 – / –
ESV01360 (0.01%) 1.6 0.28 – / –
ESV01385 (0.01%) 1.5 0.30 – / –
ESV01397 (0.01%) 0.1 0.89 – / –
ESV01424 (0.01%) 2.4 0.17 – / –
ESV01598 (0.009%) 1.1 0.39 – / –
ESV01796 (0.01%) 4.4 0.07 . – / –
ESV01810 (0.01%) 1.1 0.40 – / –
ESV02064 (0.007%) 5.6 0.04 * – / –
ESV02254 (0.01%) 2.5 0.16 – / –
ESV02466 (0.01%) 0.7 0.52 N / –
ESV02740 (0.006%) 0.5 0.61 – / –
ESV02967 (0.006%) 0.1 0.94 – / –
ESV03002 (0.005%) 1.2 0.37 – / –
ESV03058 (0.008%) 1.1 0.38 – / –
ESV03073 (0.007%) 0.7 0.55 – / –
ESV03101 (0.02%) 3.0 0.13 – / –
ESV03367 (0.009%) 2.6 0.16 – / –
ESV03396 (0.005%) 2.4 0.17 – / –
ESV03643 (0.007%) 1.4 0.32 – / –
ESV03738 (0.01%) 0.6 0.57 – / –
ESV04080 (0.007%) 1.6 0.27 – / –
ESV04335 (0.004%) 0.2 0.81 – / –
ESV04340 (0.005%) 1.6 0.27 – / –
ESV04591 (0.009%) 1.4 0.32 – / –
ESV04611 (0.006%) 5.8 0.04 * – / –
ESV04794 (0.008%) 1.0 0.41 – / –
ESV04857 (0.002%) 3.7 0.09 . N / –
ESV05045 (0.004%) 2.0 0.22 – / –
ESV05054 (0.004%) 3.3 0.11 N / –
ESV05473 (0.003%) 3.6 0.09 . N / –
ESV05784 (0.009%) 7.5 0.02 * – / –
ESV06298 (0.005%) 1.2 0.36 – / –
ESV06313 (0.008%) 2.4 0.17 – / –
ESV06555 (0.003%) 7.9 0.02 * – / –
ESV06698 (0.004%) 3.1 0.12 – / –
ESV06860 (0.009%) 2.3 0.18 – / –
ESV07031 (0.003%) 0.3 0.73 – / –
ESV07056 (0.002%) 0.7 0.54 – / –
ESV07095 (0.003%) 0.6 0.57 – / –
ESV07312 (0.002%) 0.7 0.54 – / –
ESV07374 (0.004%) 0.8 0.47 – / –
o:Rhodospirillales (4.17%) 1.4 0.32 – / –
f:Acetobacteraceae (0.7%) 9.0 0.02 * – / –
f:Acetobacteraceae_uncl (0.24%) 3.0 0.12 – / –
f:Acetobacteraceae_uncl (0.24%) 3.0 0.12 – / –
ESV00391 (0.03%) 1.8 0.25 – / –
ESV00501 (0.03%) 0.5 0.63 – / –
ESV00684 (0.02%) 2.5 0.16 – / –
ESV01153 (0.01%) 1.0 0.42 – / –
ESV01183 (0.02%) 1.8 0.25 – / –
ESV01439 (0.009%) 0.2 0.82 – / –
ESV01557 (0.01%) 2.1 0.20 – / –
ESV01965 (0.01%) 1.0 0.44 – / –
ESV02444 (0.01%) 0.2 0.82 – / –

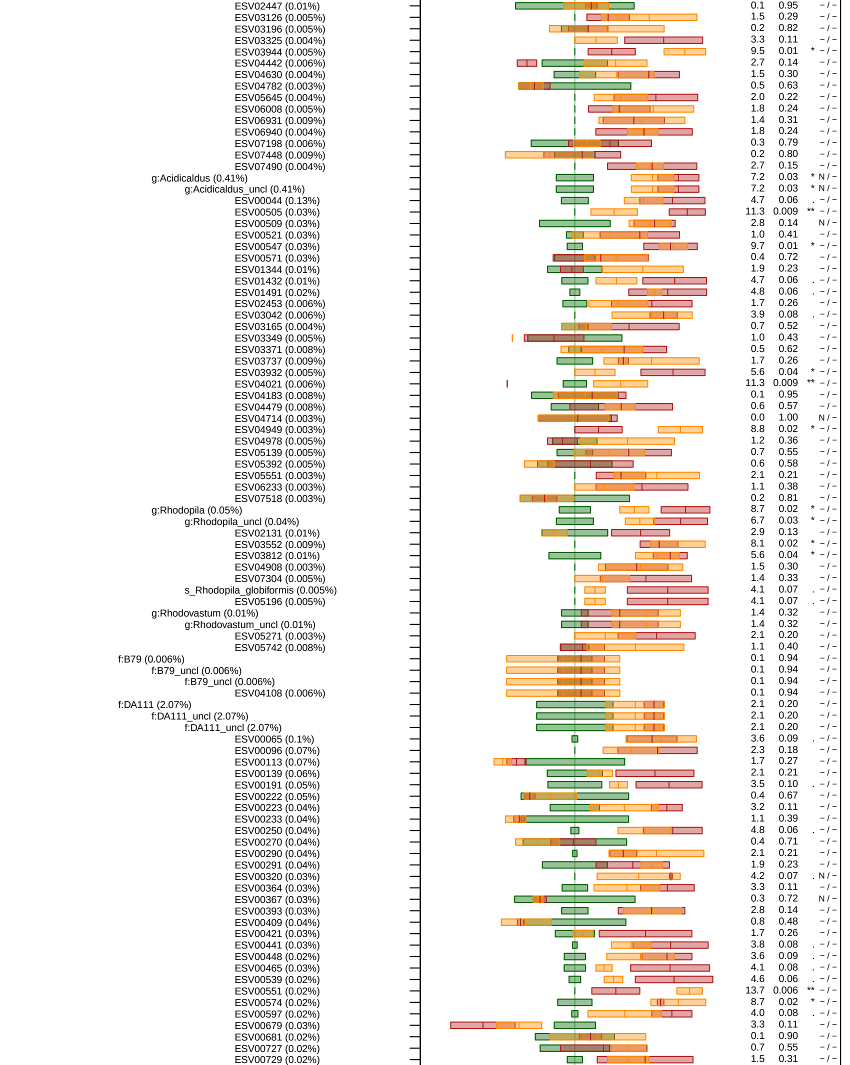
ESV02447 (0.01%) 0.1 0.95 - / -
ESV03126 (0.005%) 1.5 0.29 - / -
ESV03196 (0.005%) 0.2 0.82 - / -
ESV03325 (0.004%) 3.3 0.11 - / -
ESV03944 (0.005%) 9.5 0.01 * - / -
ESV04442 (0.006%) 2.7 0.14 - / -
ESV04630 (0.004%) 1.5 0.30 - / -
ESV04782 (0.003%) 0.5 0.63 - / -
ESV05645 (0.004%) 2.0 0.22 - / -
ESV06008 (0.005%) 1.8 0.24 - / -
ESV06931 (0.009%) 1.4 0.31 - / -
ESV06940 (0.004%) 1.8 0.24 - / -
ESV07198 (0.006%) 0.3 0.79 - / -
ESV07448 (0.009%) 0.2 0.80 - / -
ESV07490 (0.004%) 2.7 0.15 - / -
g:Acidicaldus (0.41%) 7.2 0.03 * N / -
g:Acidicaldus_uncl (0.41%) 7.2 0.03 * N / -
ESV00044 (0.13%) 4.7 0.06 . - / -
ESV00505 (0.03%) 11.3 0.009 ** - / -
ESV00509 (0.03%) 2.8 0.14 N / -
ESV00521 (0.03%) 1.0 0.41 - / -
ESV00547 (0.03%) 9.7 0.01 * - / -
ESV00571 (0.03%) 0.4 0.72 - / -
ESV01344 (0.01%) 1.9 0.23 - / -
ESV01432 (0.01%) 4.7 0.06 . - / -
ESV01491 (0.02%) 4.8 0.06 . - / -
ESV02453 (0.006%) 1.7 0.26 - / -
ESV03042 (0.006%) 3.9 0.08 . - / -
ESV03165 (0.004%) 0.7 0.52 - / -
ESV03349 (0.005%) 1.0 0.43 - / -
ESV03371 (0.008%) 0.5 0.62 - / -
ESV03737 (0.009%) 1.7 0.26 - / -
ESV03932 (0.005%) 5.6 0.04 * - / -
ESV04021 (0.006%) 11.3 0.009 ** - / -
ESV04183 (0.008%) 0.1 0.95 - / -
ESV04479 (0.008%) 0.6 0.57 - / -
ESV04714 (0.003%) 0.0 1.00 N / -
ESV04949 (0.003%) 8.8 0.02 * - / -
ESV04978 (0.005%) 1.2 0.36 - / -
ESV05139 (0.005%) 0.7 0.55 - / -
ESV05392 (0.005%) 0.6 0.58 - / -
ESV05551 (0.003%) 2.1 0.21 - / -
ESV06233 (0.003%) 1.1 0.38 - / -
ESV07518 (0.003%) 0.2 0.81 - / -
g:Rhodopila (0.05%) 8.7 0.02 * - / -
g:Rhodopila_uncl (0.04%) 6.7 0.03 * - / -
ESV02131 (0.01%) 2.9 0.13 - / -
ESV03552 (0.009%) 8.1 0.02 * - / -
ESV03812 (0.01%) 5.6 0.04 * - / -
ESV04908 (0.003%) 1.5 0.30 - / -
ESV07304 (0.005%) 1.4 0.33 - / -
s_Rhodopila_globiformis (0.005%) 4.1 0.07 . - / -
ESV05196 (0.005%) 4.1 0.07 . - / -
g:Rhodovastum (0.01%) 1.4 0.32 - / -
g:Rhodovastum_uncl (0.01%) 1.4 0.32 - / -
ESV05271 (0.003%) 2.1 0.20 - / -
ESV05742 (0.008%) 1.1 0.40 - / -
f:B79 (0.006%) 0.1 0.94 - / -
f:B79_uncl (0.006%) 0.1 0.94 - / -
f:B79_uncl (0.006%) 0.1 0.94 - / -
ESV04108 (0.006%) 0.1 0.94 - / -
f:DA111 (2.07%) 2.1 0.20 - / -
f:DA111_uncl (2.07%) 2.1 0.20 - / -
f:DA111_uncl (2.07%) 2.1 0.20 - / -
ESV00065 (0.1%) 3.6 0.09 . - / -
ESV00096 (0.07%) 2.3 0.18 - / -
ESV00113 (0.07%) 1.7 0.27 - / -
ESV00139 (0.06%) 2.1 0.21 - / -
ESV00191 (0.05%) 3.5 0.10 . - / -
ESV00222 (0.05%) 0.4 0.67 - / -
ESV00223 (0.04%) 3.2 0.11 - / -
ESV00233 (0.04%) 1.1 0.39 - / -
ESV00250 (0.04%) 4.8 0.06 . - / -
ESV00270 (0.04%) 0.4 0.71 - / -
ESV00290 (0.04%) 2.1 0.21 - / -
ESV00291 (0.04%) 1.9 0.23 - / -
ESV00320 (0.03%) 4.2 0.07 . N / -
ESV00364 (0.03%) 3.3 0.11 - / -
ESV00367 (0.03%) 0.3 0.72 N / -
ESV00393 (0.03%) 2.8 0.14 - / -
ESV00409 (0.04%) 0.8 0.48 - / -
ESV00421 (0.03%) 1.7 0.26 - / -
ESV00441 (0.03%) 3.8 0.08 . - / -
ESV00448 (0.02%) 3.6 0.09 . - / -
ESV00465 (0.03%) 4.1 0.08 . - / -
ESV00539 (0.02%) 4.6 0.06 . - / -
ESV00551 (0.02%) 13.7 0.006 ** - / -
ESV00574 (0.02%) 8.7 0.02 * - / -
ESV00597 (0.02%) 4.0 0.08 . - / -
ESV00679 (0.03%) 3.3 0.11 - / -
ESV00681 (0.02%) 0.1 0.90 - / -
ESV00727 (0.02%) 0.7 0.55 - / -
ESV00729 (0.02%) 1.5 0.31 - / -

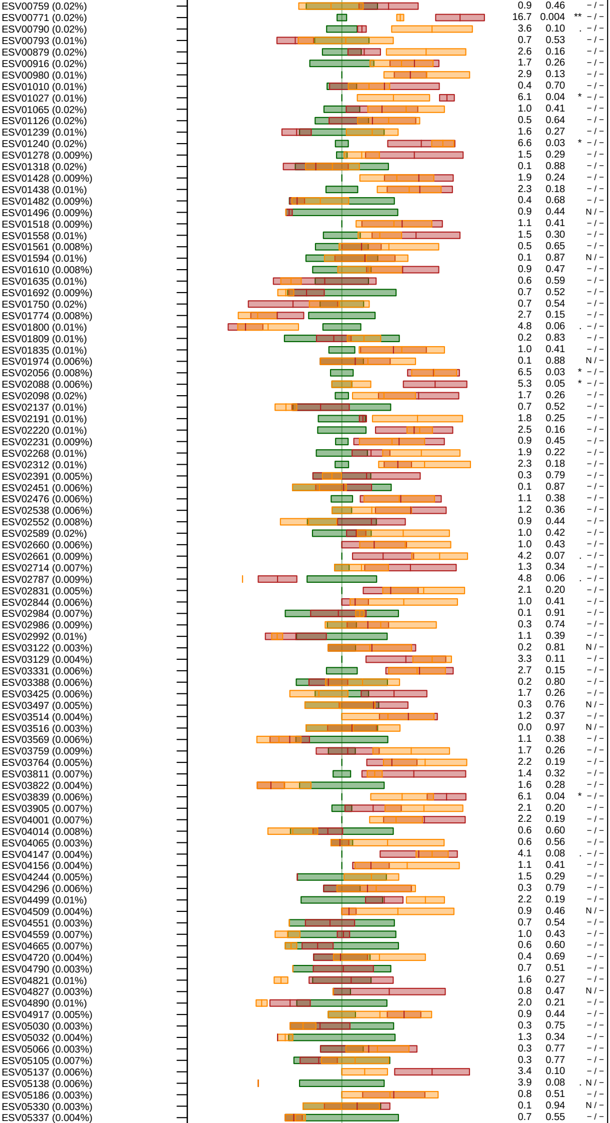
ESV00759 (0.02%) 0.9 0.46 – / –
ESV00771 (0.02%) 16.7 0.004 ** – / –
ESV00790 (0.02%) 3.6 0.10 . – / –
ESV00793 (0.01%) 0.7 0.53 – / –
ESV00879 (0.02%) 2.6 0.16 – / –
ESV00916 (0.02%) 1.7 0.26 – / –
ESV00980 (0.01%) 2.9 0.13 – / –
ESV01010 (0.01%) 0.4 0.70 – / –
ESV01027 (0.01%) 6.1 0.04 * – / –
ESV01065 (0.02%) 1.0 0.41 – / –
ESV01126 (0.02%) 0.5 0.64 – / –
ESV01239 (0.01%) 1.6 0.27 – / –
ESV01240 (0.02%) 6.6 0.03 * – / –
ESV01278 (0.009%) 1.5 0.29 – / –
ESV01318 (0.02%) 0.1 0.88 – / –
ESV01428 (0.009%) 1.9 0.24 – / –
ESV01438 (0.01%) 2.3 0.18 – / –
ESV01482 (0.009%) 0.4 0.68 – / –
ESV01496 (0.009%) 0.9 0.44 N / –
ESV01518 (0.009%) 1.1 0.41 – / –
ESV01558 (0.01%) 1.5 0.30 – / –
ESV01561 (0.008%) 0.5 0.65 – / –
ESV01594 (0.01%) 0.1 0.87 N / –
ESV01610 (0.008%) 0.9 0.47 – / –
ESV01635 (0.01%) 0.6 0.59 – / –
ESV01692 (0.009%) 0.7 0.52 – / –
ESV01750 (0.02%) 0.7 0.54 – / –
ESV01774 (0.008%) 2.7 0.15 – / –
ESV01800 (0.01%) 4.8 0.06 . – / –
ESV01809 (0.01%) 0.2 0.83 – / –
ESV01835 (0.01%) 1.0 0.41 – / –
ESV01974 (0.006%) 0.1 0.88 N / –
ESV02056 (0.008%) 6.5 0.03 * – / –
ESV02088 (0.006%) 5.3 0.05 * – / –
ESV02098 (0.02%) 1.7 0.26 – / –
ESV02137 (0.01%) 0.7 0.52 – / –
ESV02191 (0.01%) 1.8 0.25 – / –
ESV02220 (0.01%) 2.5 0.16 – / –
ESV02231 (0.009%) 0.9 0.45 – / –
ESV02268 (0.01%) 1.9 0.22 – / –
ESV02312 (0.01%) 2.3 0.18 – / –
ESV02391 (0.005%) 0.3 0.79 – / –
ESV02451 (0.006%) 0.1 0.87 – / –
ESV02476 (0.006%) 1.1 0.38 – / –
ESV02538 (0.006%) 1.2 0.36 – / –
ESV02552 (0.008%) 0.9 0.44 – / –
ESV02589 (0.02%) 1.0 0.42 – / –
ESV02660 (0.006%) 1.0 0.43 – / –
ESV02661 (0.009%) 4.2 0.07 . – / –
ESV02714 (0.007%) 1.3 0.34 – / –
ESV02787 (0.009%) 4.8 0.06 . – / –
ESV02831 (0.005%) 2.1 0.20 – / –
ESV02844 (0.006%) 1.0 0.41 – / –
ESV02984 (0.007%) 0.1 0.91 – / –
ESV02986 (0.009%) 0.3 0.74 – / –
ESV02992 (0.01%) 1.1 0.39 – / –
ESV03122 (0.003%) 0.2 0.81 N / –
ESV03129 (0.004%) 3.3 0.11 – / –
ESV03331 (0.006%) 2.7 0.15 – / –
ESV03388 (0.006%) 0.2 0.80 – / –
ESV03425 (0.006%) 1.7 0.26 – / –
ESV03497 (0.005%) 0.3 0.76 N / –
ESV03514 (0.004%) 1.2 0.37 – / –
ESV03516 (0.003%) 0.0 0.97 N / –
ESV03569 (0.006%) 1.1 0.38 – / –
ESV03759 (0.009%) 1.7 0.26 – / –
ESV03764 (0.005%) 2.2 0.19 – / –
ESV03811 (0.007%) 1.4 0.32 – / –
ESV03822 (0.004%) 1.6 0.28 – / –
ESV03839 (0.006%) 6.1 0.04 * – / –
ESV03905 (0.007%) 2.1 0.20 – / –
ESV04001 (0.007%) 2.2 0.19 – / –
ESV04014 (0.008%) 0.6 0.60 – / –
ESV04065 (0.003%) 0.6 0.56 – / –
ESV04147 (0.004%) 4.1 0.08 . – / –
ESV04156 (0.004%) 1.1 0.41 – / –
ESV04244 (0.005%) 1.5 0.29 – / –
ESV04296 (0.006%) 0.3 0.79 – / –
ESV04499 (0.01%) 2.2 0.19 – / –
ESV04509 (0.004%) 0.9 0.46 N / –
ESV04551 (0.003%) 0.7 0.54 – / –
ESV04559 (0.007%) 1.0 0.43 – / –
ESV04665 (0.007%) 0.6 0.60 – / –
ESV04720 (0.004%) 0.4 0.69 – / –
ESV04790 (0.003%) 0.7 0.51 – / –
ESV04821 (0.01%) 1.6 0.27 – / –
ESV04827 (0.003%) 0.8 0.47 N / –
ESV04890 (0.01%) 2.0 0.21 – / –
ESV04917 (0.005%) 0.9 0.44 – / –
ESV05030 (0.003%) 0.3 0.75 – / –
ESV05032 (0.004%) 1.3 0.34 – / –
ESV05066 (0.003%) 0.3 0.77 – / –
ESV05105 (0.007%) 0.3 0.77 – / –
ESV05137 (0.006%) 3.4 0.10 – / –
ESV05138 (0.006%) 3.9 0.08 . N / –
ESV05186 (0.003%) 0.8 0.51 – / –
ESV05330 (0.003%) 0.1 0.94 N / –
ESV05337 (0.004%) 0.7 0.55 – / –

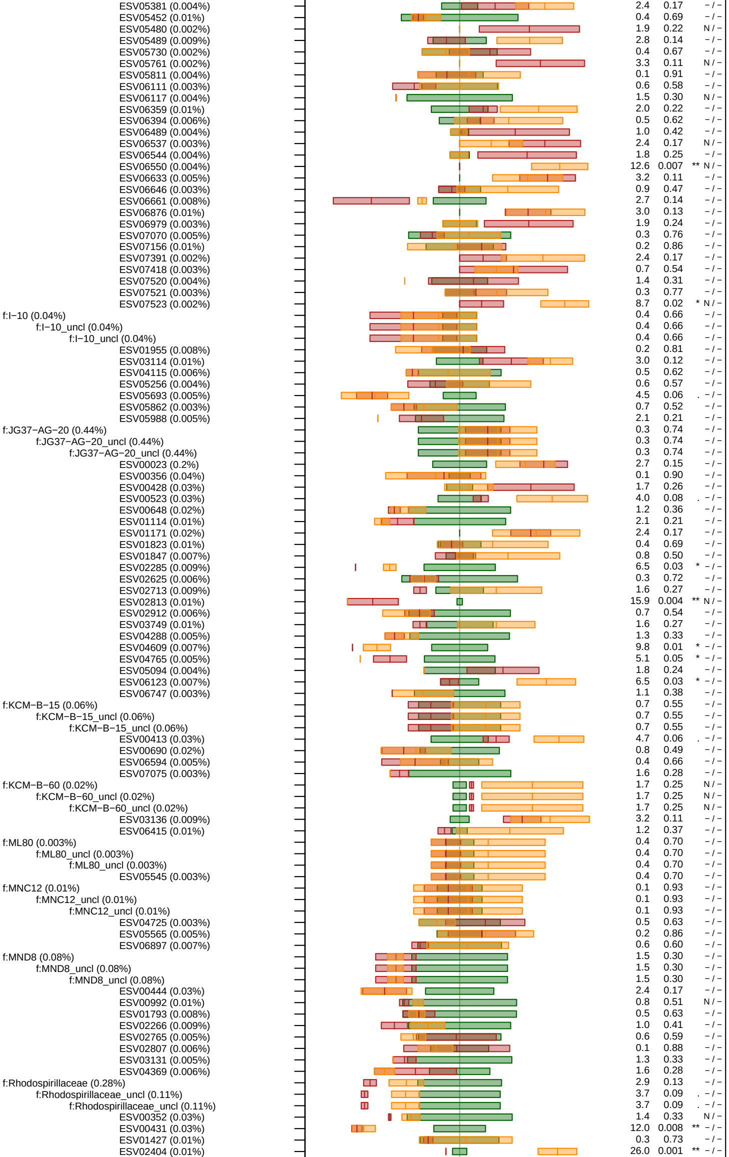
ESV05381 (0.004%) 2.4 0.17 − / −
ESV05452 (0.01%) 0.4 0.69 − / −
ESV05480 (0.002%) 1.9 0.22 N / −
ESV05489 (0.009%) 2.8 0.14 − / −
ESV05730 (0.002%) 0.4 0.67 − / −
ESV05761 (0.002%) 3.3 0.11 N / −
ESV05811 (0.004%) 0.1 0.91 − / −
ESV06111 (0.003%) 0.6 0.58 − / −
ESV06117 (0.004%) 1.5 0.30 N / −
ESV06359 (0.01%) 2.0 0.22 − / −
ESV06394 (0.006%) 0.5 0.62 − / −
ESV06489 (0.004%) 1.0 0.42 − / −
ESV06537 (0.003%) 2.4 0.17 N / −
ESV06544 (0.004%) 1.8 0.25 − / −
ESV06550 (0.004%) 12.6 0.007 ** N / −
ESV06633 (0.005%) 3.2 0.11 − / −
ESV06646 (0.003%) 0.9 0.47 − / −
ESV06661 (0.008%) 2.7 0.14 − / −
ESV06876 (0.01%) 3.0 0.13 − / −
ESV06979 (0.003%) 1.9 0.24 − / −
ESV07070 (0.005%) 0.3 0.76 − / −
ESV07156 (0.01%) 0.2 0.86 − / −
ESV07391 (0.002%) 2.4 0.17 − / −
ESV07418 (0.003%) 0.7 0.54 − / −
ESV07520 (0.004%) 1.4 0.31 − / −
ESV07521 (0.003%) 0.3 0.77 − / −
ESV07523 (0.002%) 8.7 0.02 * N / −
f:I−10 (0.04%) 0.4 0.66 − / −
f:I−10_uncl (0.04%) 0.4 0.66 − / −
f:I−10_uncl (0.04%) 0.4 0.66 − / −
ESV01955 (0.008%) 0.2 0.81 − / −
ESV03114 (0.01%) 3.0 0.12 − / −
ESV04115 (0.006%) 0.5 0.62 − / −
ESV05256 (0.004%) 0.6 0.57 − / −
ESV05693 (0.005%) 4.5 0.06 . − / −
ESV05862 (0.003%) 0.7 0.52 − / −
ESV05988 (0.005%) 2.1 0.21 − / −
f:JG37−AG−20 (0.44%) 0.3 0.74 − / −
f:JG37−AG−20_uncl (0.44%) 0.3 0.74 − / −
f:JG37−AG−20_uncl (0.44%) 0.3 0.74 − / −
ESV00023 (0.2%) 2.7 0.15 − / −
ESV00356 (0.04%) 0.1 0.90 − / −
ESV00428 (0.03%) 1.7 0.26 − / −
ESV00523 (0.03%) 4.0 0.08 . − / −
ESV00648 (0.02%) 1.2 0.36 − / −
ESV01114 (0.01%) 2.1 0.21 − / −
ESV01171 (0.02%) 2.4 0.17 − / −
ESV01823 (0.01%) 0.4 0.69 − / −
ESV01847 (0.007%) 0.8 0.50 − / −
ESV02285 (0.009%) 6.5 0.03 * − / −
ESV02625 (0.006%) 0.3 0.72 − / −
ESV02713 (0.009%) 1.6 0.27 − / −
ESV02813 (0.01%) 15.9 0.004 ** N / −
ESV02912 (0.006%) 0.7 0.54 − / −
ESV03749 (0.01%) 1.6 0.27 − / −
ESV04288 (0.005%) 1.3 0.33 − / −
ESV04609 (0.007%) 9.8 0.01 * − / −
ESV04765 (0.005%) 5.1 0.05 * − / −
ESV05094 (0.004%) 1.8 0.24 − / −
ESV06123 (0.007%) 6.5 0.03 * − / −
ESV06747 (0.003%) 1.1 0.38 − / −
f:KCM−B−15 (0.06%) 0.7 0.55 − / −
f:KCM−B−15_uncl (0.06%) 0.7 0.55 − / −
f:KCM−B−15_uncl (0.06%) 0.7 0.55 − / −
ESV00413 (0.03%) 4.7 0.06 . − / −
ESV00690 (0.02%) 0.8 0.49 − / −
ESV06594 (0.005%) 0.4 0.66 − / −
ESV07075 (0.003%) 1.6 0.28 − / −
f:KCM−B−60 (0.02%) 1.7 0.25 N / −
f:KCM−B−60_uncl (0.02%) 1.7 0.25 N / −
f:KCM−B−60_uncl (0.02%) 1.7 0.25 N / −
ESV03136 (0.009%) 3.2 0.11 − / −
ESV06415 (0.01%) 1.2 0.37 − / −
f:ML80 (0.003%) 0.4 0.70 − / −
f:ML80_uncl (0.003%) 0.4 0.70 − / −
f:ML80_uncl (0.003%) 0.4 0.70 − / −
ESV05545 (0.003%) 0.4 0.70 − / −
f:MNC12 (0.01%) 0.1 0.93 − / −
f:MNC12_uncl (0.01%) 0.1 0.93 − / −
f:MNC12_uncl (0.01%) 0.1 0.93 − / −
ESV04725 (0.003%) 0.5 0.63 − / −
ESV05565 (0.005%) 0.2 0.86 − / −
ESV06897 (0.007%) 0.6 0.60 − / −
f:MND8 (0.08%) 1.5 0.30 − / −
f:MND8_uncl (0.08%) 1.5 0.30 − / −
f:MND8_uncl (0.08%) 1.5 0.30 − / −
ESV00444 (0.03%) 2.4 0.17 − / −
ESV00992 (0.01%) 0.8 0.51 N / −
ESV01793 (0.008%) 0.5 0.63 − / −
ESV02266 (0.009%) 1.0 0.41 − / −
ESV02765 (0.005%) 0.6 0.59 − / −
ESV02807 (0.006%) 0.1 0.88 − / −
ESV03131 (0.005%) 1.3 0.33 − / −
ESV04369 (0.006%) 1.6 0.28 − / −
f:Rhodospirillaceae (0.28%) 2.9 0.13 − / −
f:Rhodospirillaceae_uncl (0.11%) 3.7 0.09 . − / −
f:Rhodospirillaceae_uncl (0.11%) 3.7 0.09 . − / −
ESV00352 (0.03%) 1.4 0.33 N / −
ESV00431 (0.03%) 12.0 0.008 ** − / −
ESV01427 (0.01%) 0.3 0.73 − / −
ESV02404 (0.01%) 26.0 0.001 ** − / −

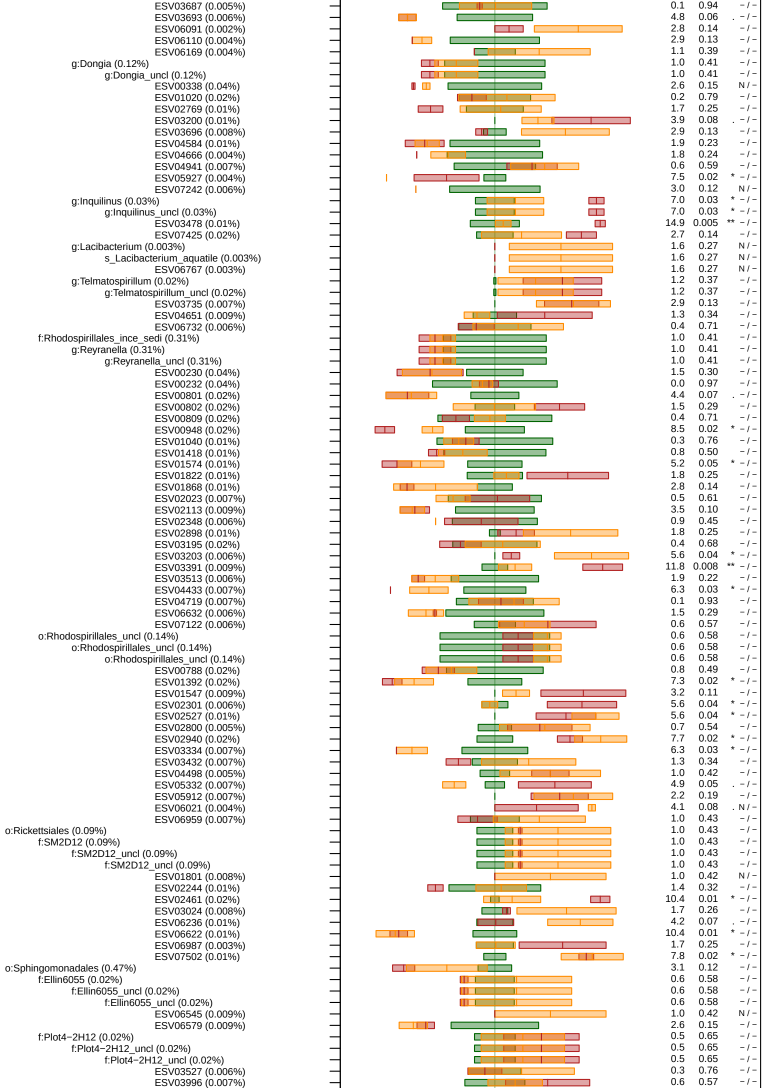
ESV03687 (0.005%) 0.1 0.94 − / −
ESV03693 (0.006%) 4.8 0.06 . − / −
ESV06091 (0.002%) 2.8 0.14 − / −
ESV06110 (0.004%) 2.9 0.13 − / −
ESV06169 (0.004%) 1.1 0.39 − / −
g:Dongia (0.12%) 1.0 0.41 − / −
g:Dongia_uncl (0.12%) 1.0 0.41 − / −
ESV00338 (0.04%) 2.6 0.15 N / −
ESV01020 (0.02%) 0.2 0.79 − / −
ESV02769 (0.01%) 1.7 0.25 − / −
ESV03200 (0.01%) 3.9 0.08 . − / −
ESV03696 (0.008%) 2.9 0.13 − / −
ESV04584 (0.01%) 1.9 0.23 − / −
ESV04666 (0.004%) 1.8 0.24 − / −
ESV04941 (0.007%) 0.6 0.59 − / −
ESV05927 (0.004%) 7.5 0.02 * − / −
ESV07242 (0.006%) 3.0 0.12 N / −
g:Inquilinus (0.03%) 7.0 0.03 * − / −
g:Inquilinus_uncl (0.03%) 7.0 0.03 * − / −
ESV03478 (0.01%) 14.9 0.005 ** − / −
ESV07425 (0.02%) 2.7 0.14 − / −
g:Lacibacterium (0.003%) 1.6 0.27 N / −
s_Lacibacterium_aquatile (0.003%) 1.6 0.27 N / −
ESV06767 (0.003%) 1.6 0.27 N / −
g:Telmatospirillum (0.02%) 1.2 0.37 − / −
g:Telmatospirillum_uncl (0.02%) 1.2 0.37 − / −
ESV03735 (0.007%) 2.9 0.13 − / −
ESV04651 (0.009%) 1.3 0.34 − / −
ESV06732 (0.006%) 0.4 0.71 − / −
f:Rhodospirillales_ince_sedi (0.31%) 1.0 0.41 − / −
g:Reyranella (0.31%) 1.0 0.41 − / −
g:Reyranella_uncl (0.31%) 1.0 0.41 − / −
ESV00230 (0.04%) 1.5 0.30 − / −
ESV00232 (0.04%) 0.0 0.97 − / −
ESV00801 (0.02%) 4.4 0.07 . − / −
ESV00802 (0.02%) 1.5 0.29 − / −
ESV00809 (0.02%) 0.4 0.71 − / −
ESV00948 (0.02%) 8.5 0.02 * − / −
ESV01040 (0.01%) 0.3 0.76 − / −
ESV01418 (0.01%) 0.8 0.50 − / −
ESV01574 (0.01%) 5.2 0.05 * − / −
ESV01822 (0.01%) 1.8 0.25 − / −
ESV01868 (0.01%) 2.8 0.14 − / −
ESV02023 (0.007%) 0.5 0.61 − / −
ESV02113 (0.009%) 3.5 0.10 − / −
ESV02348 (0.006%) 0.9 0.45 − / −
ESV02898 (0.01%) 1.8 0.25 − / −
ESV03195 (0.02%) 0.4 0.68 − / −
ESV03203 (0.006%) 5.6 0.04 * − / −
ESV03391 (0.009%) 11.8 0.008 ** − / −
ESV03513 (0.006%) 1.9 0.22 − / −
ESV04433 (0.007%) 6.3 0.03 * − / −
ESV04719 (0.007%) 0.1 0.93 − / −
ESV06632 (0.006%) 1.5 0.29 − / −
ESV07122 (0.006%) 0.6 0.57 − / −
o:Rhodospirillales_uncl (0.14%) 0.6 0.58 − / −
o:Rhodospirillales_uncl (0.14%) 0.6 0.58 − / −
o:Rhodospirillales_uncl (0.14%) 0.6 0.58 − / −
ESV00788 (0.02%) 0.8 0.49 − / −
ESV01392 (0.02%) 7.3 0.02 * − / −
ESV01547 (0.009%) 3.2 0.11 − / −
ESV02301 (0.006%) 5.6 0.04 * − / −
ESV02527 (0.01%) 5.6 0.04 * − / −
ESV02800 (0.005%) 0.7 0.54 − / −
ESV02940 (0.02%) 7.7 0.02 * − / −
ESV03334 (0.007%) 6.3 0.03 * − / −
ESV03432 (0.007%) 1.3 0.34 − / −
ESV04498 (0.005%) 1.0 0.42 − / −
ESV05332 (0.007%) 4.9 0.05 . − / −
ESV05912 (0.007%) 2.2 0.19 − / −
ESV06021 (0.004%) 4.1 0.08 . N / −
ESV06959 (0.007%) 1.0 0.43 − / −
o:Rickettsiales (0.09%) 1.0 0.43 − / −
f:SM2D12 (0.09%) 1.0 0.43 − / −
f:SM2D12_uncl (0.09%) 1.0 0.43 − / −
f:SM2D12_uncl (0.09%) 1.0 0.43 − / −
ESV01801 (0.008%) 1.0 0.42 N / −
ESV02244 (0.01%) 1.4 0.32 − / −
ESV02461 (0.02%) 10.4 0.01 * − / −
ESV03024 (0.008%) 1.7 0.26 − / −
ESV06236 (0.01%) 4.2 0.07 . − / −
ESV06622 (0.01%) 10.4 0.01 * − / −
ESV06987 (0.003%) 1.7 0.25 − / −
ESV07502 (0.01%) 7.8 0.02 * − / −
o:Sphingomonadales (0.47%) 3.1 0.12 − / −
f:Ellin6055 (0.02%) 0.6 0.58 − / −
f:Ellin6055_uncl (0.02%) 0.6 0.58 − / −
f:Ellin6055_uncl (0.02%) 0.6 0.58 − / −
ESV06545 (0.009%) 1.0 0.42 N / −
ESV06579 (0.009%) 2.6 0.15 − / −
f:Plot4−2H12 (0.02%) 0.5 0.65 − / −
f:Plot4−2H12_uncl (0.02%) 0.5 0.65 − / −
f:Plot4−2H12_uncl (0.02%) 0.5 0.65 − / −
ESV03527 (0.006%) 0.3 0.76 − / −
ESV03996 (0.007%) 0.6 0.57 − / −

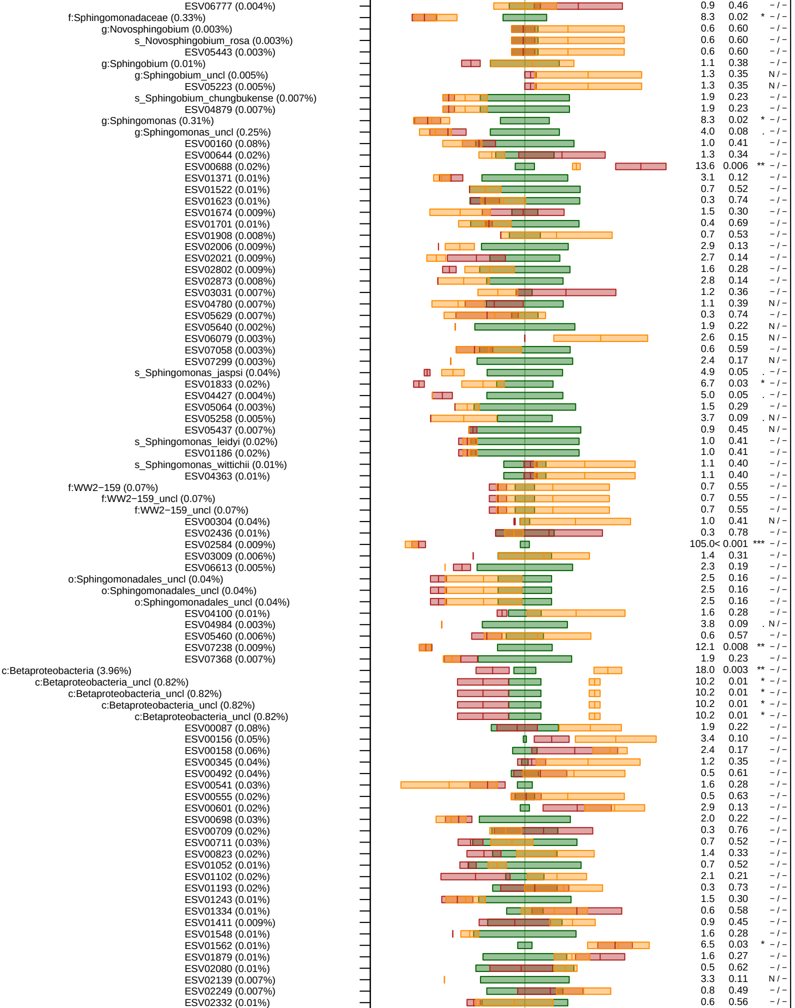
ESV06777 (0.004%) 0.9 0.46 - / -
f:Sphingomonadaceae (0.33%) 8.3 0.02 * - / -
g:Novosphingobium (0.003%) 0.6 0.60 - / -
s_Novosphingobium_rosa (0.003%) 0.6 0.60 - / -
ESV05443 (0.003%) 0.6 0.60 - / -
g:Sphingobium (0.01%) 1.1 0.38 - / -
g:Sphingobium_uncl (0.005%) 1.3 0.35 N / -
ESV05223 (0.005%) 1.3 0.35 N / -
s_Sphingobium_chungbukense (0.007%) 1.9 0.23 - / -
ESV04879 (0.007%) 1.9 0.23 - / -
g:Sphingomonas (0.31%) 8.3 0.02 * - / -
g:Sphingomonas_uncl (0.25%) 4.0 0.08 . - / -
ESV00160 (0.08%) 1.0 0.41 - / -
ESV00644 (0.02%) 1.3 0.34 - / -
ESV00688 (0.02%) 13.6 0.006 ** - / -
ESV01371 (0.01%) 3.1 0.12 - / -
ESV01522 (0.01%) 0.7 0.52 - / -
ESV01623 (0.01%) 0.3 0.74 - / -
ESV01674 (0.009%) 1.5 0.30 - / -
ESV01701 (0.01%) 0.4 0.69 - / -
ESV01908 (0.008%) 0.7 0.53 - / -
ESV02006 (0.009%) 2.9 0.13 - / -
ESV02021 (0.009%) 2.7 0.14 - / -
ESV02802 (0.009%) 1.6 0.28 - / -
ESV02873 (0.008%) 2.8 0.14 - / -
ESV03031 (0.007%) 1.2 0.36 - / -
ESV04780 (0.007%) 1.1 0.39 N / -
ESV05629 (0.007%) 0.3 0.74 - / -
ESV05640 (0.002%) 1.9 0.22 N / -
ESV06079 (0.003%) 2.6 0.15 N / -
ESV07058 (0.003%) 0.6 0.59 - / -
ESV07299 (0.003%) 2.4 0.17 N / -
s_Sphingomonas_jaspsi (0.04%) 4.9 0.05 . - / -
ESV01833 (0.02%) 6.7 0.03 * - / -
ESV04427 (0.004%) 5.0 0.05 . - / -
ESV05064 (0.003%) 1.5 0.29 - / -
ESV05258 (0.005%) 3.7 0.09 . N / -
ESV05437 (0.007%) 0.9 0.45 N / -
s_Sphingomonas_leidyi (0.02%) 1.0 0.41 - / -
ESV01186 (0.02%) 1.0 0.41 - / -
s_Sphingomonas_wittichii (0.01%) 1.1 0.40 - / -
ESV04363 (0.01%) 1.1 0.40 - / -
f:WW2−159 (0.07%) 0.7 0.55 - / -
f:WW2−159_uncl (0.07%) 0.7 0.55 - / -
f:WW2−159_uncl (0.07%) 0.7 0.55 - / -
ESV00304 (0.04%) 1.0 0.41 N / -
ESV02436 (0.01%) 0.3 0.78 - / -
ESV02584 (0.009%) 105.0< 0.001 *** - / -
ESV03009 (0.006%) 1.4 0.31 - / -
ESV06613 (0.005%) 2.3 0.19 - / -
o:Sphingomonadales_uncl (0.04%) 2.5 0.16 - / -
o:Sphingomonadales_uncl (0.04%) 2.5 0.16 - / -
o:Sphingomonadales_uncl (0.04%) 2.5 0.16 - / -
ESV04100 (0.01%) 1.6 0.28 - / -
ESV04984 (0.003%) 3.8 0.09 . N / -
ESV05460 (0.006%) 0.6 0.57 - / -
ESV07238 (0.009%) 12.1 0.008 ** - / -
ESV07368 (0.007%) 1.9 0.23 - / -
c:Betaproteobacteria (3.96%) 18.0 0.003 ** - / -
c:Betaproteobacteria_uncl (0.82%) 10.2 0.01 * - / -
c:Betaproteobacteria_uncl (0.82%) 10.2 0.01 * - / -
c:Betaproteobacteria_uncl (0.82%) 10.2 0.01 * - / -
c:Betaproteobacteria_uncl (0.82%) 10.2 0.01 * - / -
ESV00087 (0.08%) 1.9 0.22 - / -
ESV00156 (0.05%) 3.4 0.10 - / -
ESV00158 (0.06%) 2.4 0.17 - / -
ESV00345 (0.04%) 1.2 0.35 - / -
ESV00492 (0.04%) 0.5 0.61 - / -
ESV00541 (0.03%) 1.6 0.28 - / -
ESV00555 (0.02%) 0.5 0.63 - / -
ESV00601 (0.02%) 2.9 0.13 - / -
ESV00698 (0.03%) 2.0 0.22 - / -
ESV00709 (0.02%) 0.3 0.76 - / -
ESV00711 (0.03%) 0.7 0.52 - / -
ESV00823 (0.02%) 1.4 0.33 - / -
ESV01052 (0.01%) 0.7 0.52 - / -
ESV01102 (0.02%) 2.1 0.21 - / -
ESV01193 (0.02%) 0.3 0.73 - / -
ESV01243 (0.01%) 1.5 0.30 - / -
ESV01334 (0.01%) 0.6 0.58 - / -
ESV01411 (0.009%) 0.9 0.45 - / -
ESV01548 (0.01%) 1.6 0.28 - / -
ESV01562 (0.01%) 6.5 0.03 * - / -
ESV01879 (0.01%) 1.6 0.27 - / -
ESV02080 (0.01%) 0.5 0.62 - / -
ESV02139 (0.007%) 3.3 0.11 N / -
ESV02249 (0.007%) 0.8 0.49 - / -
ESV02332 (0.01%) 0.6 0.56 - / -

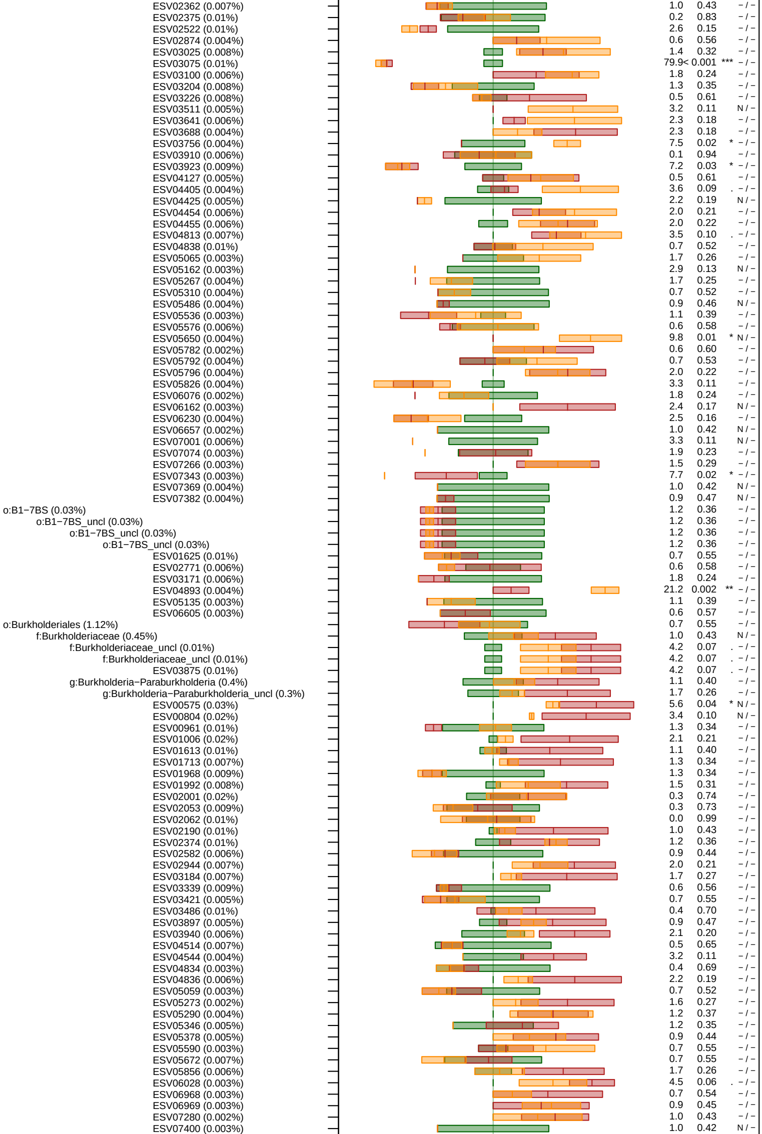
ESV02362 (0.007%) 1.0 0.43 − / −
ESV02375 (0.01%) 0.2 0.83 − / −
ESV02522 (0.01%) 2.6 0.15 − / −
ESV02874 (0.004%) 0.6 0.56 − / −
ESV03025 (0.008%) 1.4 0.32 − / −
ESV03075 (0.01%) 79.9 < 0.001 *** − / −
ESV03100 (0.006%) 1.8 0.24 − / −
ESV03204 (0.008%) 1.3 0.35 − / −
ESV03226 (0.008%) 0.5 0.61 − / −
ESV03511 (0.005%) 3.2 0.11 N / −
ESV03641 (0.006%) 2.3 0.18 − / −
ESV03688 (0.004%) 2.3 0.18 − / −
ESV03756 (0.004%) 7.5 0.02 * − / −
ESV03910 (0.006%) 0.1 0.94 − / −
ESV03923 (0.009%) 7.2 0.03 * − / −
ESV04127 (0.005%) 0.5 0.61 − / −
ESV04405 (0.004%) 3.6 0.09 . − / −
ESV04425 (0.005%) 2.2 0.19 N / −
ESV04454 (0.006%) 2.0 0.21 − / −
ESV04455 (0.006%) 2.0 0.22 − / −
ESV04813 (0.007%) 3.5 0.10 . − / −
ESV04838 (0.01%) 0.7 0.52 − / −
ESV05065 (0.003%) 1.7 0.26 − / −
ESV05162 (0.003%) 2.9 0.13 N / −
ESV05267 (0.004%) 1.7 0.25 − / −
ESV05310 (0.004%) 0.7 0.52 − / −
ESV05486 (0.004%) 0.9 0.46 N / −
ESV05536 (0.003%) 1.1 0.39 − / −
ESV05576 (0.006%) 0.6 0.58 − / −
ESV05650 (0.004%) 9.8 0.01 * N / −
ESV05782 (0.002%) 0.6 0.60 − / −
ESV05792 (0.004%) 0.7 0.53 − / −
ESV05796 (0.004%) 2.0 0.22 − / −
ESV05826 (0.004%) 3.3 0.11 − / −
ESV06076 (0.002%) 1.8 0.24 − / −
ESV06162 (0.003%) 2.4 0.17 N / −
ESV06230 (0.004%) 2.5 0.16 − / −
ESV06657 (0.002%) 1.0 0.42 N / −
ESV07001 (0.006%) 3.3 0.11 N / −
ESV07074 (0.003%) 1.9 0.23 − / −
ESV07266 (0.003%) 1.5 0.29 − / −
ESV07343 (0.003%) 7.7 0.02 * − / −
ESV07369 (0.004%) 1.0 0.42 N / −
ESV07382 (0.004%) 0.9 0.47 N / −
o:B1−7BS (0.03%) 1.2 0.36 − / −
o:B1−7BS_uncl (0.03%) 1.2 0.36 − / −
o:B1−7BS_uncl (0.03%) 1.2 0.36 − / −
o:B1−7BS_uncl (0.03%) 1.2 0.36 − / −
ESV01625 (0.01%) 0.7 0.55 − / −
ESV02771 (0.006%) 0.6 0.58 − / −
ESV03171 (0.006%) 1.8 0.24 − / −
ESV04893 (0.004%) 21.2 0.002 ** − / −
ESV05135 (0.003%) 1.1 0.39 − / −
ESV06605 (0.003%) 0.6 0.57 − / −
o:Burkholderiales (1.12%) 0.7 0.55 − / −
f:Burkholderiaceae (0.45%) 1.0 0.43 N / −
f:Burkholderiaceae_uncl (0.01%) 4.2 0.07 . − / −
f:Burkholderiaceae_uncl (0.01%) 4.2 0.07 . − / −
ESV03875 (0.01%) 4.2 0.07 . − / −
g:Burkholderia−Paraburkholderia (0.4%) 1.1 0.40 − / −
g:Burkholderia−Paraburkholderia_uncl (0.3%) 1.7 0.26 − / −
ESV00575 (0.03%) 5.6 0.04 * N / −
ESV00804 (0.02%) 3.4 0.10 N / −
ESV00961 (0.01%) 1.3 0.34 − / −
ESV01006 (0.02%) 2.1 0.21 − / −
ESV01613 (0.01%) 1.1 0.40 − / −
ESV01713 (0.007%) 1.3 0.34 − / −
ESV01968 (0.009%) 1.3 0.34 − / −
ESV01992 (0.008%) 1.5 0.31 − / −
ESV02001 (0.02%) 0.3 0.74 − / −
ESV02053 (0.009%) 0.3 0.73 − / −
ESV02062 (0.01%) 0.0 0.99 − / −
ESV02190 (0.01%) 1.0 0.43 − / −
ESV02374 (0.01%) 1.2 0.36 − / −
ESV02582 (0.006%) 0.9 0.44 − / −
ESV02944 (0.007%) 2.0 0.21 − / −
ESV03184 (0.007%) 1.7 0.27 − / −
ESV03339 (0.009%) 0.6 0.56 − / −
ESV03421 (0.005%) 0.7 0.55 − / −
ESV03486 (0.01%) 0.4 0.70 − / −
ESV03897 (0.005%) 0.9 0.47 − / −
ESV03940 (0.006%) 2.1 0.20 − / −
ESV04514 (0.007%) 0.5 0.65 − / −
ESV04544 (0.004%) 3.2 0.11 − / −
ESV04834 (0.003%) 0.4 0.69 − / −
ESV04836 (0.006%) 2.2 0.19 − / −
ESV05059 (0.003%) 0.7 0.52 − / −
ESV05273 (0.002%) 1.6 0.27 − / −
ESV05290 (0.004%) 1.2 0.37 − / −
ESV05346 (0.005%) 1.2 0.35 − / −
ESV05378 (0.005%) 0.9 0.44 − / −
ESV05590 (0.003%) 0.7 0.55 − / −
ESV05672 (0.007%) 0.7 0.55 − / −
ESV05856 (0.006%) 1.7 0.26 − / −
ESV06028 (0.003%) 4.5 0.06 . − / −
ESV06968 (0.003%) 0.7 0.54 − / −
ESV06969 (0.003%) 0.9 0.45 − / −
ESV07280 (0.002%) 1.0 0.43 − / −
ESV07400 (0.003%) 1.0 0.42 N / −

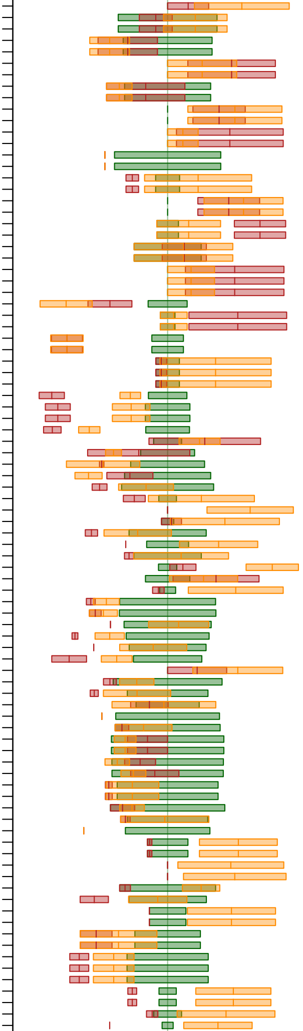

ESV07454 (0.002%)	1.7	0.27		− / −
s_Burkholderia_ambifaria (0.01%)	0.3	0.73		− / −
ESV02012 (0.01%)	0.3	0.73		− / −
s_Burkholderia_cenocepacia (0.01%)	0.8	0.49		− / −
ESV01472 (0.01%)	0.8	0.49		− / −
s_Burkholderia_monticola (0.003%)	1.0	0.43		− / −
ESV06135 (0.003%)	1.0	0.43		− / −
s_Burkholderia_susongensis (0.004%)	0.5	0.63		− / −
ESV06140 (0.004%)	0.5	0.63		− / −
s_Paraburkholderia_nodosa (0.008%)	1.3	0.34		− / −
ESV02174 (0.008%)	1.3	0.34		− / −
s_Paraburkholderia_silvatlantica (0.005%)	1.0	0.41		− / −
ESV04664 (0.005%)	1.0	0.41		− / −
s_Paraburkholderia_soli (0.005%)	1.4	0.32		N / −
ESV06711 (0.005%)	1.4	0.32		N / −
s_Paraburkholderia_terrae (0.02%)	1.1	0.40		− / −
ESV01322 (0.02%)	1.1	0.40		− / −
s_Paraburkholderia_unamae (0.008%)	1.9	0.23		− / −
ESV07032 (0.008%)	1.9	0.23		− / −
s_Paraburkholderia_xenovorans (0.006%)	3.9	0.08	.	− / −
ESV07476 (0.006%)	3.9	0.08	.	− / −
s_Paraburkholderia_zhejiangensis (0.02%)	0.1	0.94		− / −
ESV02132 (0.02%)	0.1	0.94		− / −
g:Chitinimonas (0.003%)	1.2	0.37		− / −
g:Chitinimonas_uncl (0.003%)	1.2	0.37		− / −
ESV04646 (0.003%)	1.2	0.37		− / −
g:Cupriavidus (0.02%)	5.0	0.05	.	− / −
g:Cupriavidus_uncl (0.007%)	1.7	0.26		− / −
ESV03329 (0.007%)	1.7	0.26		− / −
s_Cupriavidus_necator (0.02%)	13.4	0.006	**	− / −
ESV07281 (0.02%)	13.4	0.006	**	− / −
g:Ralstonia (0.01%)	0.8	0.49		− / −
s_Ralstonia_pickettii (0.01%)	0.8	0.49		− / −
ESV01672 (0.01%)	0.8	0.49		− / −
f:Comamonadaceae (0.46%)	16.5	0.004	**	− / −
f:Comamonadaceae_uncl (0.27%)	9.3	0.01	*	− / −
f:Comamonadaceae_uncl (0.27%)	9.3	0.01	*	− / −
ESV01511 (0.01%)	15.6	0.004	**	− / −
ESV01791 (0.01%)	0.3	0.73		− / −
ESV01887 (0.02%)	0.6	0.56		− / −
ESV02072 (0.02%)	1.6	0.28		− / −
ESV02123 (0.01%)	1.8	0.24		− / −
ESV02130 (0.02%)	1.2	0.35		− / −
ESV02261 (0.006%)	1.1	0.39		− / −
ESV02390 (0.006%)	3.7	0.09	.	N / −
ESV02416 (0.006%)	1.0	0.43		− / −
ESV03357 (0.008%)	1.7	0.27		− / −
ESV03885 (0.009%)	3.3	0.11		− / −
ESV04022 (0.01%)	0.6	0.56		− / −
ESV04070 (0.007%)	10.2	0.01	*	− / −
ESV04911 (0.003%)	0.5	0.61		− / −
ESV05544 (0.008%)	2.3	0.18		− / −
ESV05630 (0.02%)	2.0	0.22		− / −
ESV05753 (0.006%)	1.8	0.24		− / −
ESV05803 (0.009%)	1.4	0.31		− / −
ESV06231 (0.02%)	3.4	0.10		− / −
ESV06296 (0.003%)	1.8	0.25		− / −
ESV06482 (0.02%)	4.3	0.07	.	− / −
ESV06569 (0.002%)	1.3	0.34		− / −
ESV06668 (0.007%)	0.8	0.51		− / −
ESV06757 (0.01%)	1.5	0.30		− / −
ESV07047 (0.004%)	0.1	0.93		− / −
ESV07274 (0.003%)	1.6	0.28		N / −
ESV07553 (0.006%)	0.4	0.67		− / −
g:Aquincola (0.02%)	0.4	0.71		− / −
g:Aquincola_uncl (0.02%)	0.4	0.71		− / −
ESV04578 (0.008%)	0.5	0.61		− / −
ESV05319 (0.008%)	0.2	0.80		− / −
g:Ideonella (0.02%)	0.8	0.49		− / −
g:Ideonella_uncl (0.02%)	0.8	0.49		− / −
ESV04961 (0.005%)	0.5	0.64		− / −
ESV05176 (0.009%)	0.5	0.65		N / −
ESV07357 (0.004%)	3.9	0.08	.	N / −
g:Inhella (0.02%)	3.4	0.10		− / −
g:Inhella_uncl (0.02%)	3.4	0.10		− / −
ESV02936 (0.004%)	1.4	0.31		N / −
ESV04370 (0.005%)	1.7	0.26		N / −
ESV06198 (0.009%)	1.6	0.27		− / −
g:Pelomonas (0.02%)	1.9	0.23		− / −
s_Pelomonas_puraquae (0.005%)	2.4	0.17		− / −
ESV03354 (0.005%)	2.4	0.17		− / −
s_Pelomonas_saccharophila (0.01%)	1.4	0.31		− / −
ESV03003 (0.01%)	1.4	0.31		− / −
g:Piscinibacter (0.02%)	2.8	0.14		− / −
g:Piscinibacter_uncl (0.02%)	2.8	0.14		− / −
ESV03726 (0.02%)	2.8	0.14		− / −
g:Ramlibacter (0.02%)	5.2	0.05	*	− / −
g:Ramlibacter_uncl (0.02%)	5.2	0.05	*	− / −
ESV05239 (0.009%)	1.7	0.25		− / −
ESV07213 (0.007%)	7.3	0.02	*	− / −

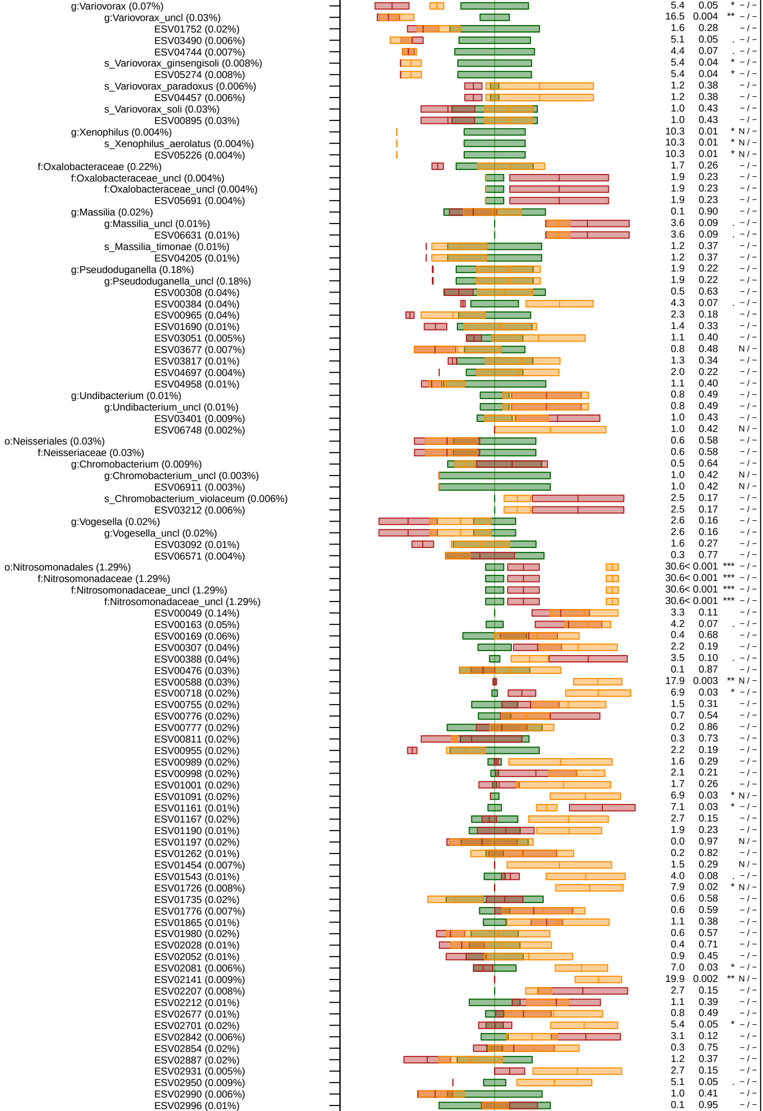
g:Variovorax (0.07%) 5.4 0.05 * – / –
g:Variovorax_uncl (0.03%) 16.5 0.004 ** – / –
ESV01752 (0.02%) 1.6 0.28 – / –
ESV03490 (0.006%) 5.1 0.05 . – / –
ESV04744 (0.007%) 4.4 0.07 . – / –
s_Variovorax_ginsengisoli (0.008%) 5.4 0.04 * – / –
ESV05274 (0.008%) 5.4 0.04 * – / –
s_Variovorax_paradoxus (0.006%) 1.2 0.38 – / –
ESV04457 (0.006%) 1.2 0.38 – / –
s_Variovorax_soli (0.03%) 1.0 0.43 – / –
ESV00895 (0.03%) 1.0 0.43 – / –
g:Xenophilus (0.004%) 10.3 0.01 * N / –
s_Xenophilus_aerolatus (0.004%) 10.3 0.01 * N / –
ESV05226 (0.004%) 10.3 0.01 * N / –
f:Oxalobacteraceae (0.22%) 1.7 0.26 – / –
f:Oxalobacteraceae_uncl (0.004%) 1.9 0.23 – / –
f:Oxalobacteraceae_uncl (0.004%) 1.9 0.23 – / –
ESV05691 (0.004%) 1.9 0.23 – / –
g:Massilia (0.02%) 0.1 0.90 – / –
g:Massilia_uncl (0.01%) 3.6 0.09 . – / –
ESV06631 (0.01%) 3.6 0.09 . – / –
s_Massilia_timonae (0.01%) 1.2 0.37 – / –
ESV04205 (0.01%) 1.2 0.37 – / –
g:Pseudoduganella (0.18%) 1.9 0.22 – / –
g:Pseudoduganella_uncl (0.18%) 1.9 0.22 – / –
ESV00308 (0.04%) 0.5 0.63 – / –
ESV00384 (0.04%) 4.3 0.07 . – / –
ESV00965 (0.04%) 2.3 0.18 – / –
ESV01690 (0.01%) 1.4 0.33 – / –
ESV03051 (0.005%) 1.1 0.40 – / –
ESV03677 (0.007%) 0.8 0.48 N / –
ESV03817 (0.01%) 1.3 0.34 – / –
ESV04697 (0.004%) 2.0 0.22 – / –
ESV04958 (0.01%) 1.1 0.40 – / –
g:Undibacterium (0.01%) 0.8 0.49 – / –
g:Undibacterium_uncl (0.01%) 0.8 0.49 – / –
ESV03401 (0.009%) 1.0 0.43 – / –
ESV06748 (0.002%) 1.0 0.42 N / –
o:Neisseriales (0.03%) 0.6 0.58 – / –
f:Neisseriaceae (0.03%) 0.6 0.58 – / –
g:Chromobacterium (0.009%) 0.5 0.64 – / –
g:Chromobacterium_uncl (0.003%) 1.0 0.42 N / –
ESV06911 (0.003%) 1.0 0.42 N / –
s_Chromobacterium_violaceum (0.006%) 2.5 0.17 – / –
ESV03212 (0.006%) 2.5 0.17 – / –
g:Vogesella (0.02%) 2.6 0.16 – / –
g:Vogesella_uncl (0.02%) 2.6 0.16 – / –
ESV03092 (0.01%) 1.6 0.27 – / –
ESV06571 (0.004%) 0.3 0.77 – / –
o:Nitrosomonadales (1.29%) 30.6 < 0.001 *** – / –
f:Nitrosomonadaceae (1.29%) 30.6 < 0.001 *** – / –
f:Nitrosomonadaceae_uncl (1.29%) 30.6 < 0.001 *** – / –
f:Nitrosomonadaceae_uncl (1.29%) 30.6 < 0.001 *** – / –
ESV00049 (0.14%) 3.3 0.11 – / –
ESV00163 (0.05%) 4.2 0.07 . – / –
ESV00169 (0.06%) 0.4 0.68 – / –
ESV00307 (0.04%) 2.2 0.19 – / –
ESV00388 (0.04%) 3.5 0.10 . – / –
ESV00476 (0.03%) 0.1 0.87 – / –
ESV00588 (0.03%) 17.9 0.003 ** N / –
ESV00718 (0.02%) 6.9 0.03 * – / –
ESV00755 (0.02%) 1.5 0.31 – / –
ESV00776 (0.02%) 0.7 0.54 – / –
ESV00777 (0.02%) 0.2 0.86 – / –
ESV00811 (0.02%) 0.3 0.73 – / –
ESV00955 (0.02%) 2.2 0.19 – / –
ESV00989 (0.02%) 1.6 0.29 – / –
ESV00998 (0.02%) 2.1 0.21 – / –
ESV01001 (0.02%) 1.7 0.26 – / –
ESV01091 (0.02%) 6.9 0.03 * N / –
ESV01161 (0.01%) 7.1 0.03 * – / –
ESV01167 (0.02%) 2.7 0.15 – / –
ESV01190 (0.01%) 1.9 0.23 – / –
ESV01197 (0.02%) 0.0 0.97 N / –
ESV01262 (0.01%) 0.2 0.82 – / –
ESV01454 (0.007%) 1.5 0.29 N / –
ESV01543 (0.01%) 4.0 0.08 . – / –
ESV01726 (0.008%) 7.9 0.02 * N / –
ESV01735 (0.02%) 0.6 0.58 – / –
ESV01776 (0.007%) 0.6 0.59 – / –
ESV01865 (0.01%) 1.1 0.38 – / –
ESV01980 (0.02%) 0.6 0.57 – / –
ESV02028 (0.01%) 0.4 0.71 – / –
ESV02052 (0.01%) 0.9 0.45 – / –
ESV02081 (0.006%) 7.0 0.03 * – / –
ESV02141 (0.009%) 19.9 0.002 ** N / –
ESV02207 (0.008%) 2.7 0.15 – / –
ESV02212 (0.01%) 1.1 0.39 – / –
ESV02677 (0.01%) 0.8 0.49 – / –
ESV02701 (0.02%) 5.4 0.05 * – / –
ESV02842 (0.006%) 3.1 0.12 – / –
ESV02854 (0.02%) 0.3 0.75 – / –
ESV02887 (0.02%) 1.2 0.37 – / –
ESV02931 (0.005%) 2.7 0.15 – / –
ESV02950 (0.009%) 5.1 0.05 . – / –
ESV02990 (0.006%) 1.0 0.41 – / –
ESV02996 (0.01%) 0.1 0.95 – / –

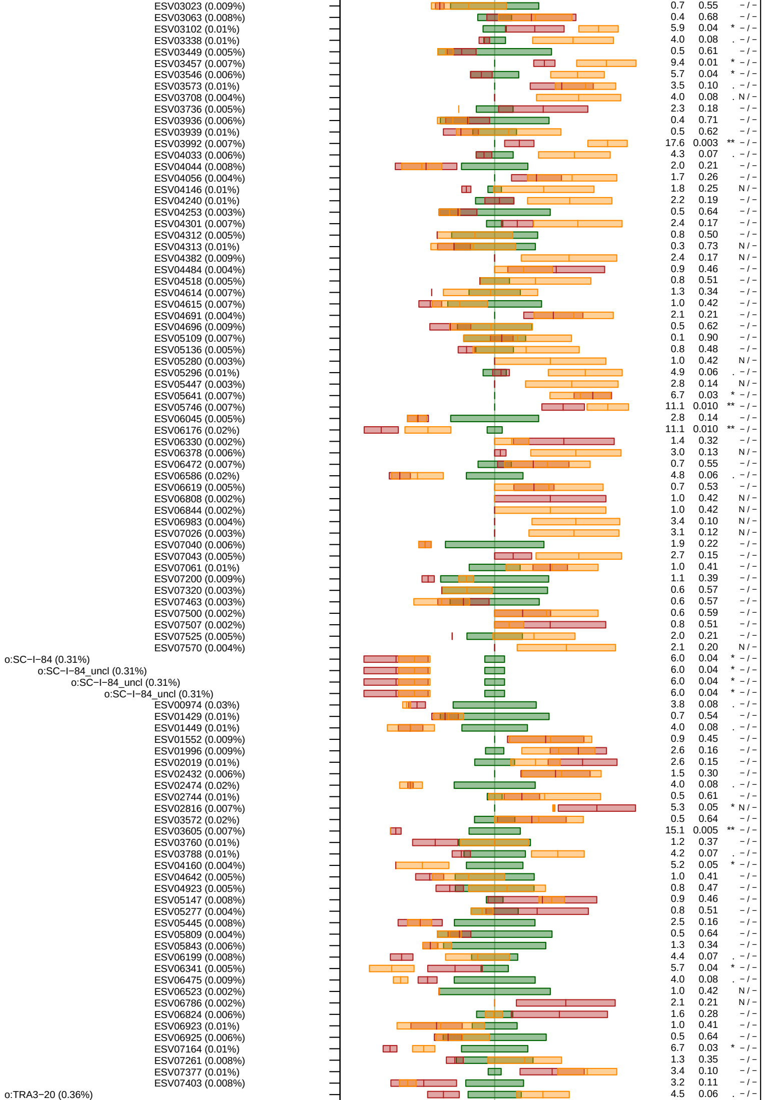
ESV03023 (0.009%) 0.7 0.55 – / –
ESV03063 (0.008%) 0.4 0.68 – / –
ESV03102 (0.01%) 5.9 0.04 * – / –
ESV03338 (0.01%) 4.0 0.08 . – / –
ESV03449 (0.005%) 0.5 0.61 – / –
ESV03457 (0.007%) 9.4 0.01 * – / –
ESV03546 (0.006%) 5.7 0.04 * – / –
ESV03573 (0.01%) 3.5 0.10 . – / –
ESV03708 (0.004%) 4.0 0.08 . N / –
ESV03736 (0.005%) 2.3 0.18 – / –
ESV03936 (0.006%) 0.4 0.71 – / –
ESV03939 (0.01%) 0.5 0.62 – / –
ESV03992 (0.007%) 17.6 0.003 ** – / –
ESV04033 (0.006%) 4.3 0.07 . – / –
ESV04044 (0.008%) 2.0 0.21 – / –
ESV04056 (0.004%) 1.7 0.26 – / –
ESV04146 (0.01%) 1.8 0.25 N / –
ESV04240 (0.01%) 2.2 0.19 – / –
ESV04253 (0.003%) 0.5 0.64 – / –
ESV04301 (0.007%) 2.4 0.17 – / –
ESV04312 (0.005%) 0.8 0.50 – / –
ESV04313 (0.01%) 0.3 0.73 N / –
ESV04382 (0.009%) 2.4 0.17 N / –
ESV04484 (0.004%) 0.9 0.46 – / –
ESV04518 (0.005%) 0.8 0.51 – / –
ESV04614 (0.007%) 1.3 0.34 – / –
ESV04615 (0.007%) 1.0 0.42 – / –
ESV04691 (0.004%) 2.1 0.21 – / –
ESV04696 (0.009%) 0.5 0.62 – / –
ESV05109 (0.007%) 0.1 0.90 – / –
ESV05136 (0.005%) 0.8 0.48 – / –
ESV05280 (0.003%) 1.0 0.42 N / –
ESV05296 (0.01%) 4.9 0.06 . – / –
ESV05447 (0.003%) 2.8 0.14 N / –
ESV05641 (0.007%) 6.7 0.03 * – / –
ESV05746 (0.007%) 11.1 0.010 ** – / –
ESV06045 (0.005%) 2.8 0.14 – / –
ESV06176 (0.02%) 11.1 0.010 ** – / –
ESV06330 (0.002%) 1.4 0.32 – / –
ESV06378 (0.006%) 3.0 0.13 N / –
ESV06472 (0.007%) 0.7 0.55 – / –
ESV06586 (0.02%) 4.8 0.06 . – / –
ESV06619 (0.005%) 0.7 0.53 – / –
ESV06808 (0.002%) 1.0 0.42 N / –
ESV06844 (0.002%) 1.0 0.42 N / –
ESV06983 (0.004%) 3.4 0.10 N / –
ESV07026 (0.003%) 3.1 0.12 N / –
ESV07040 (0.006%) 1.9 0.22 – / –
ESV07043 (0.005%) 2.7 0.15 – / –
ESV07061 (0.01%) 1.0 0.41 – / –
ESV07200 (0.009%) 1.1 0.39 – / –
ESV07320 (0.003%) 0.6 0.57 – / –
ESV07463 (0.003%) 0.6 0.57 – / –
ESV07500 (0.002%) 0.6 0.59 – / –
ESV07507 (0.002%) 0.8 0.51 – / –
ESV07525 (0.005%) 2.0 0.21 – / –
ESV07570 (0.004%) 2.1 0.20 N / –
o:SC−I−84 (0.31%) 6.0 0.04 * – / –
o:SC−I−84_uncl (0.31%) 6.0 0.04 * – / –
o:SC−I−84_uncl (0.31%) 6.0 0.04 * – / –
o:SC−I−84_uncl (0.31%) 6.0 0.04 * – / –
ESV00974 (0.03%) 3.8 0.08 . – / –
ESV01429 (0.01%) 0.7 0.54 – / –
ESV01449 (0.01%) 4.0 0.08 . – / –
ESV01552 (0.009%) 0.9 0.45 – / –
ESV01996 (0.009%) 2.6 0.16 – / –
ESV02019 (0.01%) 2.6 0.15 – / –
ESV02432 (0.006%) 1.5 0.30 – / –
ESV02474 (0.02%) 4.0 0.08 . – / –
ESV02744 (0.01%) 0.5 0.61 – / –
ESV02816 (0.007%) 5.3 0.05 * N / –
ESV03572 (0.02%) 0.5 0.64 – / –
ESV03605 (0.007%) 15.1 0.005 ** – / –
ESV03760 (0.01%) 1.2 0.37 – / –
ESV03788 (0.01%) 4.2 0.07 . – / –
ESV04160 (0.004%) 5.2 0.05 * – / –
ESV04642 (0.005%) 1.0 0.41 – / –
ESV04923 (0.005%) 0.8 0.47 – / –
ESV05147 (0.008%) 0.9 0.46 – / –
ESV05277 (0.004%) 0.8 0.51 – / –
ESV05445 (0.008%) 2.5 0.16 – / –
ESV05809 (0.004%) 0.5 0.64 – / –
ESV05843 (0.006%) 1.3 0.34 – / –
ESV06199 (0.008%) 4.4 0.07 . – / –
ESV06341 (0.005%) 5.7 0.04 * – / –
ESV06475 (0.009%) 4.0 0.08 . – / –
ESV06523 (0.002%) 1.0 0.42 N / –
ESV06786 (0.002%) 2.1 0.21 N / –
ESV06824 (0.006%) 1.6 0.28 – / –
ESV06923 (0.01%) 1.0 0.41 – / –
ESV06925 (0.006%) 0.5 0.64 – / –
ESV07164 (0.01%) 6.7 0.03 * – / –
ESV07261 (0.008%) 1.3 0.35 – / –
ESV07377 (0.01%) 3.4 0.10 – / –
ESV07403 (0.008%) 3.2 0.11 – / –
o:TRA3−20 (0.36%) 4.5 0.06 . – / –

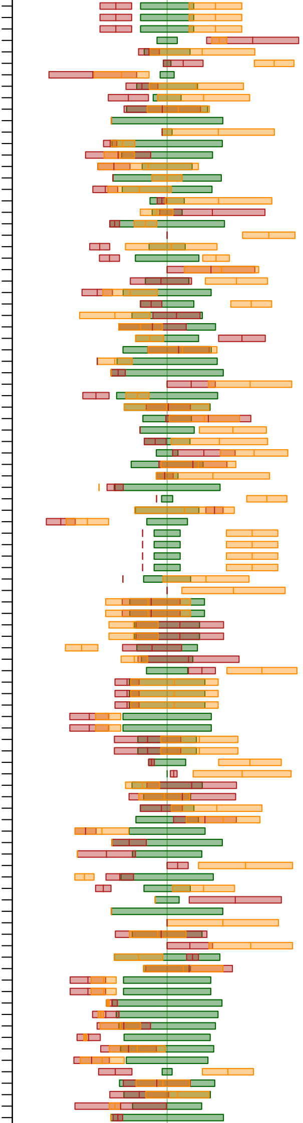

o:TRA3−20_uncl (0.36%)	4.5	0.06	.	− / −
o:TRA3−20_uncl (0.36%)	4.5	0.06	.	− / −
o:TRA3−20_uncl (0.36%)	4.5	0.06	.	− / −
ESV00475 (0.03%)	2.4	0.17		− / −
ESV00732 (0.02%)	0.6	0.56		− / −
ESV00862 (0.02%)	12.5	0.007	**	− / −
ESV00898 (0.02%)	1.5	0.29		− / −
ESV00914 (0.03%)	0.7	0.55		− / −
ESV01465 (0.02%)	1.6	0.28		− / −
ESV01761 (0.01%)	0.0	0.95		− / −
ESV01804 (0.01%)	1.0	0.42		N / −
ESV02085 (0.006%)	0.9	0.45		N / −
ESV02622 (0.01%)	0.8	0.48		− / −
ESV02623 (0.006%)	0.7	0.53		− / −
ESV02673 (0.01%)	0.6	0.56		− / −
ESV02778 (0.01%)	0.9	0.45		− / −
ESV02947 (0.006%)	0.9	0.45		− / −
ESV03088 (0.009%)	0.9	0.44		− / −
ESV03147 (0.006%)	0.8	0.49		− / −
ESV03167 (0.007%)	0.6	0.58		− / −
ESV03294 (0.004%)	15.0	0.005	**	N / −
ESV03342 (0.008%)	1.9	0.23		− / −
ESV03373 (0.01%)	6.6	0.03	*	− / −
ESV04233 (0.003%)	0.8	0.51		− / −
ESV04356 (0.01%)	2.2	0.19		− / −
ESV04472 (0.004%)	1.3	0.35		− / −
ESV04662 (0.005%)	7.0	0.03	*	− / −
ESV04670 (0.006%)	1.1	0.39		N / −
ESV04706 (0.004%)	0.1	0.88		N / −
ESV04738 (0.004%)	4.2	0.07	.	N / −
ESV04995 (0.007%)	0.0	0.95		− / −
ESV05408 (0.004%)	1.4	0.32		− / −
ESV05525 (0.003%)	0.9	0.46		N / −
ESV05707 (0.003%)	2.4	0.17		− / −
ESV05727 (0.009%)	1.3	0.33		− / −
ESV05744 (0.003%)	0.0	1.00		N / −
ESV05834 (0.003%)	0.4	0.67		− / −
ESV05838 (0.004%)	3.7	0.09	.	− / −
ESV06000 (0.005%)	1.2	0.37		− / −
ESV06226 (0.006%)	2.6	0.16		− / −
ESV06314 (0.003%)	0.2	0.81		− / −
ESV06530 (0.003%)	0.7	0.55		− / −
ESV07013 (0.004%)	1.3	0.33		− / −
ESV07248 (0.01%)	25.5	0.001	**	− / −
ESV07510 (0.006%)	0.5	0.64		− / −
c:Deltaproteobacteria (3.35%)	8.6	0.02	*	− / −
c:Deltaproteobacteria_uncl (0.006%)	11.9	0.008	**	− / −
c:Deltaproteobacteria_uncl (0.006%)	11.9	0.008	**	− / −
c:Deltaproteobacteria_uncl (0.006%)	11.9	0.008	**	− / −
c:Deltaproteobacteria_uncl (0.006%)	11.9	0.008	**	− / −
ESV05930 (0.003%)	2.2	0.20		− / −
ESV06818 (0.003%)	1.7	0.27		N / −
o:Bdellovibrionales (0.05%)	0.1	0.93		− / −
f:Bdellovibrionaceae (0.05%)	0.1	0.93		− / −
g:Bdellovibrio (0.04%)	0.5	0.64		− / −
g:Bdellovibrio:uncl (0.04%)	0.5	0.64		− / −
ESV04729 (0.02%)	3.0	0.12		− / −
ESV06855 (0.008%)	0.7	0.55		− / −
ESV06981 (0.02%)	3.8	0.09	.	− / −
g:OM27_clade (0.01%)	0.6	0.60		− / −
g:OM27_clade_uncl (0.01%)	0.6	0.60		− / −
ESV05574 (0.01%)	0.6	0.60		− / −
o:Desulfurellales (1.46%)	2.0	0.22		− / −
f:Desulfurellaceae (1.46%)	2.0	0.22		− / −
g:G55 (0.13%)	0.6	0.58		− / −
g:G55_uncl (0.13%)	0.6	0.58		− / −
ESV01049 (0.02%)	6.3	0.03	*	− / −
ESV01816 (0.01%)	2.1	0.20		N / −
ESV02094 (0.02%)	0.5	0.62		− / −
ESV02711 (0.02%)	0.1	0.93		− / −
ESV02942 (0.004%)	0.9	0.47		− / −
ESV03946 (0.008%)	0.7	0.52		− / −
ESV04003 (0.009%)	2.5	0.17		− / −
ESV04307 (0.003%)	0.7	0.53		− / −
ESV04721 (0.003%)	3.1	0.12		N / −
ESV04990 (0.003%)	2.3	0.18		− / −
ESV05214 (0.007%)	2.1	0.20		− / −
ESV06087 (0.009%)	5.0	0.05	.	− / −
ESV06400 (0.004%)	2.5	0.16		− / −
ESV06512 (0.003%)	1.0	0.42		N / −
ESV06810 (0.002%)	1.0	0.42		N / −
ESV06904 (0.008%)	0.0	0.98		N / −
ESV07055 (0.003%)	2.5	0.16		− / −
ESV07163 (0.003%)	0.6	0.56		− / −
ESV07434 (0.008%)	0.1	0.91		− / −
g:H16 (1.33%)	2.2	0.19		− / −
g:H16_uncl (1.33%)	2.2	0.19		− / −
ESV00085 (0.1%)	1.1	0.40		N / −
ESV00129 (0.08%)	1.5	0.30		− / −
ESV00202 (0.05%)	0.6	0.58		− / −
ESV00226 (0.05%)	3.2	0.11		− / −
ESV00245 (0.04%)	0.3	0.73		− / −
ESV00257 (0.06%)	2.0	0.21		− / −
ESV00471 (0.04%)	10.0	0.01	*	− / −
ESV00502 (0.03%)	0.0	0.98		− / −
ESV00504 (0.03%)	0.3	0.74		− / −
ESV00876 (0.02%)	0.7	0.52		− / −
ESV00896 (0.02%)	0.9	0.46		N / −

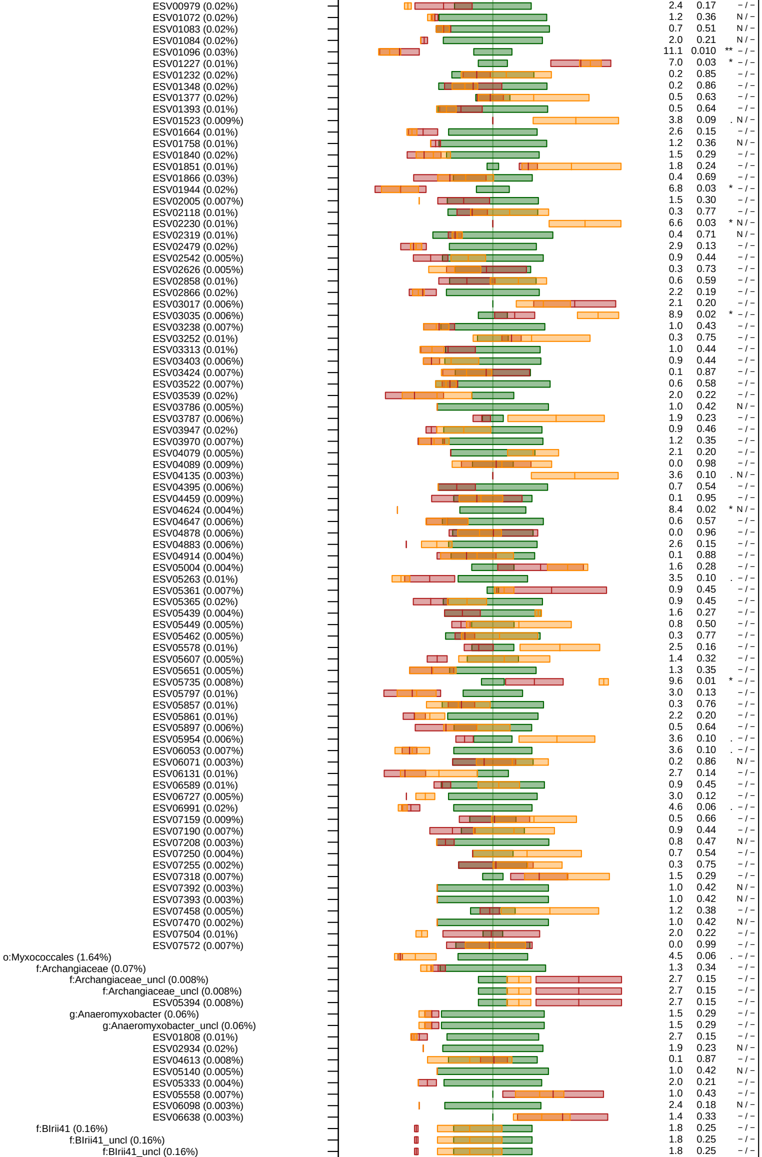

ESV00979 (0.02%) 2.4 0.17 – / –
ESV01072 (0.02%) 1.2 0.36 N / –
ESV01083 (0.02%) 0.7 0.51 N / –
ESV01084 (0.02%) 2.0 0.21 N / –
ESV01096 (0.03%) 11.1 0.010 ** – / –
ESV01227 (0.01%) 7.0 0.03 * – / –
ESV01232 (0.02%) 0.2 0.85 – / –
ESV01348 (0.02%) 0.2 0.86 – / –
ESV01377 (0.02%) 0.5 0.63 – / –
ESV01393 (0.01%) 0.5 0.64 – / –
ESV01523 (0.009%) 3.8 0.09 . N / –
ESV01664 (0.01%) 2.6 0.15 – / –
ESV01758 (0.01%) 1.2 0.36 N / –
ESV01840 (0.02%) 1.5 0.29 – / –
ESV01851 (0.01%) 1.8 0.24 – / –
ESV01866 (0.03%) 0.4 0.69 – / –
ESV01944 (0.02%) 6.8 0.03 * – / –
ESV02005 (0.007%) 1.5 0.30 – / –
ESV02118 (0.01%) 0.3 0.77 – / –
ESV02230 (0.01%) 6.6 0.03 * N / –
ESV02319 (0.01%) 0.4 0.71 N / –
ESV02479 (0.02%) 2.9 0.13 – / –
ESV02542 (0.005%) 0.9 0.44 – / –
ESV02626 (0.005%) 0.3 0.73 – / –
ESV02858 (0.01%) 0.6 0.59 – / –
ESV02866 (0.02%) 2.2 0.19 – / –
ESV03017 (0.006%) 2.1 0.20 – / –
ESV03035 (0.006%) 8.9 0.02 * – / –
ESV03238 (0.007%) 1.0 0.43 – / –
ESV03252 (0.01%) 0.3 0.75 – / –
ESV03313 (0.01%) 1.0 0.44 – / –
ESV03403 (0.006%) 0.9 0.44 – / –
ESV03424 (0.007%) 0.1 0.87 – / –
ESV03522 (0.007%) 0.6 0.58 – / –
ESV03539 (0.02%) 2.0 0.22 – / –
ESV03786 (0.005%) 1.0 0.42 N / –
ESV03787 (0.006%) 1.9 0.23 – / –
ESV03947 (0.02%) 0.9 0.46 – / –
ESV03970 (0.007%) 1.2 0.35 – / –
ESV04079 (0.005%) 2.1 0.20 – / –
ESV04089 (0.009%) 0.0 0.98 – / –
ESV04135 (0.003%) 3.6 0.10 . N / –
ESV04395 (0.006%) 0.7 0.54 – / –
ESV04459 (0.009%) 0.1 0.95 – / –
ESV04624 (0.004%) 8.4 0.02 * N / –
ESV04647 (0.006%) 0.6 0.57 – / –
ESV04878 (0.006%) 0.0 0.96 – / –
ESV04883 (0.006%) 2.6 0.15 – / –
ESV04914 (0.004%) 0.1 0.88 – / –
ESV05004 (0.004%) 1.6 0.28 – / –
ESV05263 (0.01%) 3.5 0.10 . – / –
ESV05361 (0.007%) 0.9 0.45 – / –
ESV05365 (0.02%) 0.9 0.45 – / –
ESV05439 (0.004%) 1.6 0.27 – / –
ESV05449 (0.005%) 0.8 0.50 – / –
ESV05462 (0.005%) 0.3 0.77 – / –
ESV05578 (0.01%) 2.5 0.16 – / –
ESV05607 (0.005%) 1.4 0.32 – / –
ESV05651 (0.005%) 1.3 0.35 – / –
ESV05735 (0.008%) 9.6 0.01 * – / –
ESV05797 (0.01%) 3.0 0.13 – / –
ESV05857 (0.01%) 0.3 0.76 – / –
ESV05861 (0.01%) 2.2 0.20 – / –
ESV05897 (0.006%) 0.5 0.64 – / –
ESV05954 (0.006%) 3.6 0.10 . – / –
ESV06053 (0.007%) 3.6 0.10 . – / –
ESV06071 (0.003%) 0.2 0.86 N / –
ESV06131 (0.01%) 2.7 0.14 – / –
ESV06589 (0.01%) 0.9 0.45 – / –
ESV06727 (0.005%) 3.0 0.12 – / –
ESV06991 (0.02%) 4.6 0.06 . – / –
ESV07159 (0.009%) 0.5 0.66 – / –
ESV07190 (0.007%) 0.9 0.44 – / –
ESV07208 (0.003%) 0.8 0.47 N / –
ESV07250 (0.004%) 0.7 0.54 – / –
ESV07255 (0.002%) 0.3 0.75 – / –
ESV07318 (0.007%) 1.5 0.29 – / –
ESV07392 (0.003%) 1.0 0.42 N / –
ESV07393 (0.003%) 1.0 0.42 N / –
ESV07458 (0.005%) 1.2 0.38 – / –
ESV07470 (0.002%) 1.0 0.42 N / –
ESV07504 (0.01%) 2.0 0.22 – / –
ESV07572 (0.007%) 0.0 0.99 – / –
o:Myxococcales (1.64%) 4.5 0.06 . – / –
f:Archangiaceae (0.07%) 1.3 0.34 – / –
f:Archangiaceae_uncl (0.008%) 2.7 0.15 – / –
f:Archangiaceae_uncl (0.008%) 2.7 0.15 – / –
ESV05394 (0.008%) 2.7 0.15 – / –
g:Anaeromyxobacter (0.06%) 1.5 0.29 – / –
g:Anaeromyxobacter_uncl (0.06%) 1.5 0.29 – / –
ESV01808 (0.01%) 2.7 0.15 – / –
ESV02934 (0.02%) 1.9 0.23 N / –
ESV04613 (0.008%) 0.1 0.87 – / –
ESV05140 (0.005%) 1.0 0.42 N / –
ESV05333 (0.004%) 2.0 0.21 – / –
ESV05558 (0.007%) 1.0 0.43 – / –
ESV06098 (0.003%) 2.4 0.18 N / –
ESV06638 (0.003%) 1.4 0.33 – / –
f:BIrii41 (0.16%) 1.8 0.25 – / –
f:BIrii41_uncl (0.16%) 1.8 0.25 – / –
f:BIrii41_uncl (0.16%) 1.8 0.25 – / –

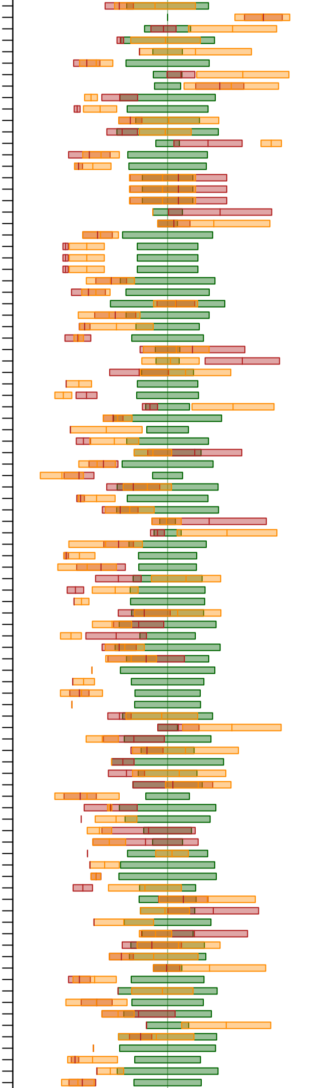

ESV00837 (0.02%)	0.5	0.61	- / -
ESV01582 (0.01%)	8.2	0.02	* - / -
ESV02820 (0.01%)	1.7	0.26	- / -
ESV03312 (0.02%)	0.6	0.57	- / -
ESV03821 (0.003%)	0.7	0.53	- / -
ESV03858 (0.01%)	2.7	0.15	- / -
ESV04073 (0.004%)	1.9	0.23	- / -
ESV04190 (0.009%)	1.2	0.38	- / -
ESV04293 (0.009%)	1.7	0.26	- / -
ESV06248 (0.02%)	3.5	0.10	- / -
ESV06663 (0.004%)	0.4	0.69	- / -
ESV07141 (0.007%)	0.6	0.60	- / -
ESV07303 (0.007%)	5.8	0.04	* - / -
ESV07309 (0.006%)	2.3	0.18	- / -
ESV07427 (0.01%)	3.7	0.09	. - / -
f:Blfdi19 (0.01%)	0.0	0.95	- / -
f:Blfdi19_uncl (0.01%)	0.0	0.95	- / -
f:Blfdi19_uncl (0.01%)	0.0	0.95	- / -
ESV05657 (0.004%)	1.3	0.34	- / -
ESV06228 (0.004%)	0.5	0.61	- / -
ESV06745 (0.003%)	1.8	0.24	- / -
f:Haliangiaceae (0.79%)	7.0	0.03	* - / -
g:Haliangium (0.79%)	7.0	0.03	* - / -
g:Haliangium_uncl (0.79%)	7.0	0.03	* - / -
ESV00272 (0.06%)	1.1	0.40	- / -
ESV00708 (0.03%)	2.6	0.16	- / -
ESV00873 (0.02%)	0.0	0.98	- / -
ESV00971 (0.02%)	1.0	0.42	- / -
ESV01003 (0.02%)	2.2	0.19	- / -
ESV01025 (0.03%)	5.4	0.05	* - / -
ESV01210 (0.02%)	0.1	0.89	- / -
ESV01253 (0.02%)	2.3	0.18	- / -
ESV01373 (0.02%)	0.5	0.65	- / -
ESV01727 (0.01%)	8.5	0.02	* - / -
ESV01969 (0.009%)	7.9	0.02	* - / -
ESV01973 (0.008%)	2.6	0.15	- / -
ESV02272 (0.01%)	0.9	0.47	- / -
ESV02513 (0.01%)	4.2	0.07	. - / -
ESV02585 (0.02%)	2.3	0.18	- / -
ESV02645 (0.005%)	0.4	0.71	- / -
ESV02777 (0.02%)	1.7	0.25	- / -
ESV02823 (0.02%)	10.6	0.01	* - / -
ESV02865 (0.009%)	0.2	0.80	- / -
ESV02908 (0.01%)	3.2	0.11	- / -
ESV02988 (0.02%)	0.5	0.61	- / -
ESV02997 (0.005%)	0.5	0.63	- / -
ESV03170 (0.01%)	1.6	0.29	- / -
ESV03286 (0.009%)	1.0	0.41	- / -
ESV03330 (0.03%)	8.3	0.02	* - / -
ESV03386 (0.01%)	2.4	0.17	N / -
ESV03778 (0.008%)	1.3	0.35	- / -
ESV03883 (0.009%)	3.2	0.11	- / -
ESV03980 (0.006%)	5.7	0.04	* - / -
ESV03995 (0.005%)	0.2	0.80	- / -
ESV04046 (0.003%)	0.7	0.54	- / -
ESV04068 (0.007%)	3.9	0.08	. - / -
ESV04176 (0.01%)	0.6	0.57	- / -
ESV04178 (0.02%)	0.3	0.78	- / -
ESV04228 (0.007%)	2.6	0.16	N / -
ESV04318 (0.005%)	5.6	0.04	* - / -
ESV04346 (0.007%)	4.8	0.06	. - / -
ESV04625 (0.004%)	8.4	0.02	* N / -
ESV04676 (0.006%)	0.6	0.58	- / -
ESV04700 (0.005%)	1.2	0.36	- / -
ESV04845 (0.004%)	1.0	0.41	- / -
ESV04906 (0.008%)	0.3	0.75	- / -
ESV05089 (0.002%)	0.8	0.49	N / -
ESV05110 (0.009%)	0.7	0.54	- / -
ESV05116 (0.004%)	0.2	0.82	- / -
ESV05289 (0.009%)	4.0	0.08	. - / -
ESV05363 (0.01%)	1.1	0.40	- / -
ESV05373 (0.005%)	2.5	0.16	- / -
ESV05389 (0.005%)	1.3	0.34	- / -
ESV05413 (0.004%)	0.8	0.49	- / -
ESV05454 (0.02%)	3.6	0.09	. - / -
ESV05493 (0.005%)	2.1	0.20	- / -
ESV05507 (0.007%)	2.1	0.20	N / -
ESV05752 (0.01%)	2.6	0.15	- / -
ESV05778 (0.004%)	0.3	0.74	- / -
ESV06165 (0.002%)	0.7	0.55	- / -
ESV06220 (0.006%)	1.5	0.30	- / -
ESV06286 (0.006%)	0.3	0.74	- / -
ESV06329 (0.005%)	0.1	0.87	N / -
ESV06345 (0.004%)	0.5	0.64	- / -
ESV06366 (0.003%)	0.5	0.62	- / -
ESV06476 (0.03%)	3.5	0.10	. - / -
ESV06501 (0.004%)	0.7	0.55	- / -
ESV06635 (0.01%)	2.1	0.21	- / -
ESV06637 (0.003%)	0.5	0.61	- / -
ESV06792 (0.004%)	2.1	0.20	- / -
ESV06902 (0.01%)	0.1	0.87	- / -
ESV06958 (0.003%)	2.4	0.17	N / -
ESV06994 (0.01%)	4.2	0.07	. - / -
ESV07042 (0.003%)	1.6	0.29	- / -
ESV07088 (0.02%)	4.9	0.06	. - / -

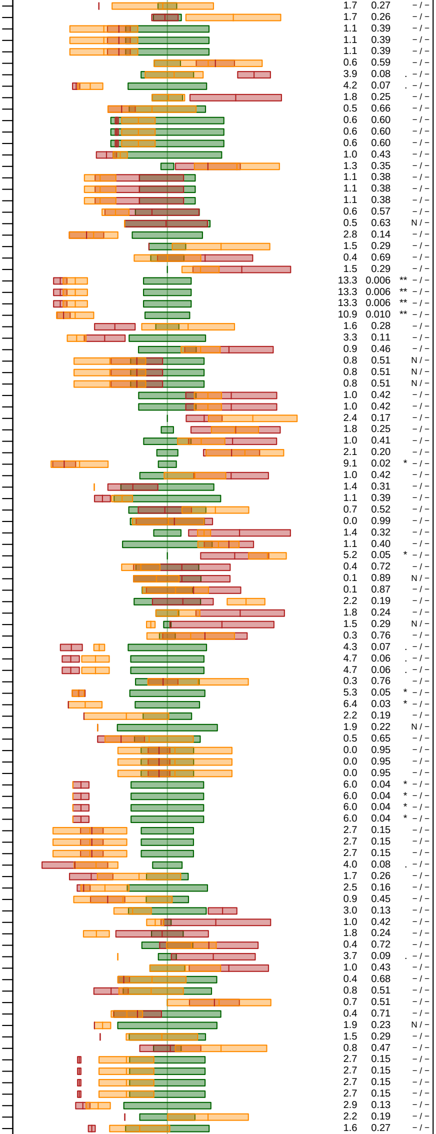

ESV07313 (0.006%)	1.7	0.27		– / –
ESV07449 (0.005%)	1.7	0.26		– / –
f:mle1−27 (0.03%)	1.1	0.39		– / –
f:mle1−27_uncl (0.03%)	1.1	0.39		– / –
f:mle1−27_uncl (0.03%)	1.1	0.39		– / –
ESV03530 (0.004%)	0.6	0.59		– / –
ESV04686 (0.008%)	3.9	0.08	.	– / –
ESV05406 (0.01%)	4.2	0.07	.	– / –
ESV06627 (0.003%)	1.8	0.25		– / –
ESV06861 (0.004%)	0.5	0.66		– / –
f:Myxococcaceae (0.03%)	0.6	0.60		– / –
g:Aggregicoccus (0.03%)	0.6	0.60		– / –
s_Aggregicoccus_edonensis (0.03%)	0.6	0.60		– / –
ESV01155 (0.02%)	1.0	0.43		– / –
ESV01849 (0.009%)	1.3	0.35		– / –
f:P3OB−42 (0.04%)	1.1	0.38		– / –
f:P3OB−42_uncl (0.04%)	1.1	0.38		– / –
f:P3OB−42_uncl (0.04%)	1.1	0.38		– / –
ESV03519 (0.009%)	0.6	0.57		– / –
ESV04616 (0.008%)	0.5	0.63		N / –
ESV04733 (0.01%)	2.8	0.14		– / –
ESV06061 (0.002%)	1.5	0.29		– / –
ESV06746 (0.004%)	0.4	0.69		– / –
ESV07513 (0.004%)	1.5	0.29		– / –
f:Phaselicystidaceae (0.05%)	13.3	0.006	**	– / –
g:Phaselicystis (0.05%)	13.3	0.006	**	– / –
g:Phaselicystis_uncl (0.05%)	13.3	0.006	**	– / –
ESV01763 (0.02%)	10.9	0.010	**	– / –
ESV02079 (0.01%)	1.6	0.28		– / –
ESV04045 (0.01%)	3.3	0.11		– / –
f:Polyangiaceae (0.25%)	0.9	0.46		– / –
f:Polyangiaceae_uncl (0.03%)	0.8	0.51		N / –
f:Polyangiaceae_uncl (0.03%)	0.8	0.51		N / –
ESV04035 (0.03%)	0.8	0.51		N / –
g:Sorangium (0.21%)	1.0	0.42		– / –
g:Sorangium_uncl (0.21%)	1.0	0.42		– / –
ESV01901 (0.009%)	2.4	0.17		– / –
ESV02305 (0.01%)	1.8	0.25		– / –
ESV02341 (0.02%)	1.0	0.41		– / –
ESV03156 (0.009%)	2.1	0.20		– / –
ESV03468 (0.03%)	9.1	0.02	*	– / –
ESV03524 (0.01%)	1.0	0.42		– / –
ESV03917 (0.004%)	1.4	0.31		– / –
ESV04672 (0.01%)	1.1	0.39		– / –
ESV05039 (0.01%)	0.7	0.52		– / –
ESV05251 (0.01%)	0.0	0.99		– / –
ESV05485 (0.02%)	1.4	0.32		– / –
ESV05653 (0.02%)	1.1	0.40		– / –
ESV05668 (0.007%)	5.2	0.05	*	– / –
ESV05900 (0.006%)	0.4	0.72		– / –
ESV06005 (0.002%)	0.1	0.89		N / –
ESV06397 (0.004%)	0.1	0.87		– / –
ESV06409 (0.005%)	2.2	0.19		– / –
ESV06506 (0.005%)	1.8	0.24		– / –
ESV06690 (0.01%)	1.5	0.29		N / –
ESV07364 (0.009%)	0.3	0.76		– / –
f:Sandaracinaceae (0.05%)	4.3	0.07	.	– / –
f:Sandaracinaceae_uncl (0.04%)	4.7	0.06	.	– / –
f:Sandaracinaceae_uncl (0.04%)	4.7	0.06	.	– / –
ESV02612 (0.009%)	0.3	0.76		– / –
ESV03587 (0.008%)	5.3	0.05	*	– / –
ESV04002 (0.006%)	6.4	0.03	*	– / –
ESV04555 (0.005%)	2.2	0.19		– / –
ESV04797 (0.003%)	1.9	0.22		N / –
ESV04992 (0.01%)	0.5	0.65		– / –
g:Sandaracinus (0.01%)	0.0	0.95		– / –
g:Sandaracinus_uncl (0.01%)	0.0	0.95		– / –
ESV02437 (0.01%)	0.0	0.95		– / –
f:Vulgatibacteraceae (0.006%)	6.0	0.04	*	– / –
g:Vulgatibacter (0.006%)	6.0	0.04	*	– / –
g:Vulgatibacter_uncl (0.006%)	6.0	0.04	*	– / –
ESV05849 (0.006%)	6.0	0.04	*	– / –
o:Myxococcales_uncl (0.16%)	2.7	0.15		– / –
o:Myxococcales_uncl (0.16%)	2.7	0.15		– / –
o:Myxococcales_uncl (0.16%)	2.7	0.15		– / –
ESV00536 (0.04%)	4.0	0.08	.	– / –
ESV01111 (0.02%)	1.7	0.26		– / –
ESV02441 (0.009%)	2.5	0.16		– / –
ESV02828 (0.008%)	0.9	0.45		– / –
ESV03711 (0.01%)	3.0	0.13		– / –
ESV03884 (0.01%)	1.0	0.42		– / –
ESV04200 (0.006%)	1.8	0.24		– / –
ESV04796 (0.005%)	0.4	0.72		– / –
ESV04956 (0.006%)	3.7	0.09	.	– / –
ESV05244 (0.003%)	1.0	0.43		– / –
ESV05678 (0.006%)	0.4	0.68		– / –
ESV05726 (0.02%)	0.8	0.51		– / –
ESV05941 (0.002%)	0.7	0.51		– / –
ESV06192 (0.006%)	0.4	0.71		– / –
ESV06986 (0.005%)	1.9	0.23		N / –
ESV07486 (0.003%)	1.5	0.29		– / –
ESV07519 (0.005%)	0.8	0.47		– / –
o:NB1−j (0.07%)	2.7	0.15		– / –
o:NB1−j_uncl (0.07%)	2.7	0.15		– / –
o:NB1−j_uncl (0.07%)	2.7	0.15		– / –
o:NB1−j_uncl (0.07%)	2.7	0.15		– / –
ESV04631 (0.01%)	2.9	0.13		– / –
ESV04806 (0.005%)	2.2	0.19		– / –
ESV04944 (0.04%)	1.6	0.27		– / –

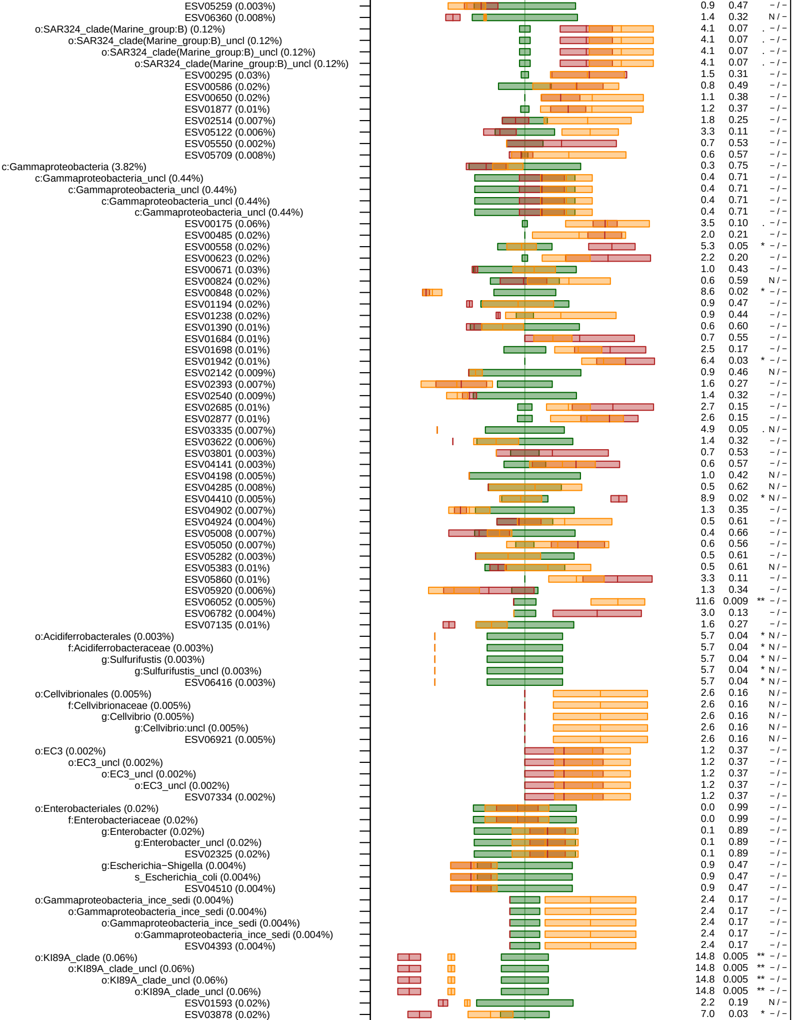

ESV05259 (0.003%) 0.9 0.47 - / -
ESV06360 (0.008%) 1.4 0.32 N / -
o:SAR324_clade(Marine_group:B) (0.12%) 4.1 0.07 . - / -
o:SAR324_clade(Marine_group:B)_uncl (0.12%) 4.1 0.07 . - / -
o:SAR324_clade(Marine_group:B)_uncl (0.12%) 4.1 0.07 . - / -
o:SAR324_clade(Marine_group:B)_uncl (0.12%) 4.1 0.07 . - / -
ESV00295 (0.03%) 1.5 0.31 - / -
ESV00586 (0.02%) 0.8 0.49 - / -
ESV00650 (0.02%) 1.1 0.38 - / -
ESV01877 (0.01%) 1.2 0.37 - / -
ESV02514 (0.007%) 1.8 0.25 - / -
ESV05122 (0.006%) 3.3 0.11 - / -
ESV05550 (0.002%) 0.7 0.53 - / -
ESV05709 (0.008%) 0.6 0.57 - / -
c:Gammaproteobacteria (3.82%) 0.3 0.75 - / -
c:Gammaproteobacteria_uncl (0.44%) 0.4 0.71 - / -
c:Gammaproteobacteria_uncl (0.44%) 0.4 0.71 - / -
c:Gammaproteobacteria_uncl (0.44%) 0.4 0.71 - / -
c:Gammaproteobacteria_uncl (0.44%) 0.4 0.71 - / -
ESV00175 (0.06%) 3.5 0.10 . - / -
ESV00485 (0.02%) 2.0 0.21 - / -
ESV00558 (0.02%) 5.3 0.05 * - / -
ESV00623 (0.02%) 2.2 0.20 - / -
ESV00671 (0.03%) 1.0 0.43 - / -
ESV00824 (0.02%) 0.6 0.59 N / -
ESV00848 (0.02%) 8.6 0.02 * - / -
ESV01194 (0.02%) 0.9 0.47 - / -
ESV01238 (0.02%) 0.9 0.44 - / -
ESV01390 (0.01%) 0.6 0.60 - / -
ESV01684 (0.01%) 0.7 0.55 - / -
ESV01698 (0.01%) 2.5 0.17 - / -
ESV01942 (0.01%) 6.4 0.03 * - / -
ESV02142 (0.009%) 0.9 0.46 N / -
ESV02393 (0.007%) 1.6 0.27 - / -
ESV02540 (0.009%) 1.4 0.32 - / -
ESV02685 (0.01%) 2.7 0.15 - / -
ESV02877 (0.01%) 2.6 0.15 - / -
ESV03335 (0.007%) 4.9 0.05 . N / -
ESV03622 (0.006%) 1.4 0.32 - / -
ESV03801 (0.003%) 0.7 0.53 - / -
ESV04141 (0.003%) 0.6 0.57 - / -
ESV04198 (0.005%) 1.0 0.42 N / -
ESV04285 (0.008%) 0.5 0.62 N / -
ESV04410 (0.005%) 8.9 0.02 * N / -
ESV04902 (0.007%) 1.3 0.35 - / -
ESV04924 (0.004%) 0.5 0.61 - / -
ESV05008 (0.007%) 0.4 0.66 - / -
ESV05050 (0.007%) 0.6 0.56 - / -
ESV05282 (0.003%) 0.5 0.61 - / -
ESV05383 (0.01%) 0.5 0.61 N / -
ESV05860 (0.01%) 3.3 0.11 - / -
ESV05920 (0.006%) 1.3 0.34 - / -
ESV06052 (0.005%) 11.6 0.009 ** - / -
ESV06782 (0.004%) 3.0 0.13 - / -
ESV07135 (0.01%) 1.6 0.27 - / -
o:Acidiferrobacterales (0.003%) 5.7 0.04 * N / -
f:Acidiferrobacteraceae (0.003%) 5.7 0.04 * N / -
g:Sulfurifustis (0.003%) 5.7 0.04 * N / -
g:Sulfurifustis_uncl (0.003%) 5.7 0.04 * N / -
ESV06416 (0.003%) 5.7 0.04 * N / -
o:Cellvibrionales (0.005%) 2.6 0.16 N / -
f:Cellvibrionaceae (0.005%) 2.6 0.16 N / -
g:Cellvibrio (0.005%) 2.6 0.16 N / -
g:Cellvibrio:uncl (0.005%) 2.6 0.16 N / -
ESV06921 (0.005%) 2.6 0.16 N / -
o:EC3 (0.002%) 1.2 0.37 - / -
o:EC3_uncl (0.002%) 1.2 0.37 - / -
o:EC3_uncl (0.002%) 1.2 0.37 - / -
o:EC3_uncl (0.002%) 1.2 0.37 - / -
ESV07334 (0.002%) 1.2 0.37 - / -
o:Enterobacteriales (0.02%) 0.0 0.99 - / -
f:Enterobacteriaceae (0.02%) 0.0 0.99 - / -
g:Enterobacter (0.02%) 0.1 0.89 - / -
g:Enterobacter_uncl (0.02%) 0.1 0.89 - / -
ESV02325 (0.02%) 0.1 0.89 - / -
g:Escherichia−Shigella (0.004%) 0.9 0.47 - / -
s_Escherichia_coli (0.004%) 0.9 0.47 - / -
ESV04510 (0.004%) 0.9 0.47 - / -
o:Gammaproteobacteria_ince_sedi (0.004%) 2.4 0.17 - / -
o:Gammaproteobacteria_ince_sedi (0.004%) 2.4 0.17 - / -
o:Gammaproteobacteria_ince_sedi (0.004%) 2.4 0.17 - / -
o:Gammaproteobacteria_ince_sedi (0.004%) 2.4 0.17 - / -
ESV04393 (0.004%) 2.4 0.17 - / -
o:KI89A_clade (0.06%) 14.8 0.005 ** - / -
o:KI89A_clade_uncl (0.06%) 14.8 0.005 ** - / -
o:KI89A_clade_uncl (0.06%) 14.8 0.005 ** - / -
o:KI89A_clade_uncl (0.06%) 14.8 0.005 ** - / -
ESV01593 (0.02%) 2.2 0.19 N / -
ESV03878 (0.02%) 7.0 0.03 * - / -

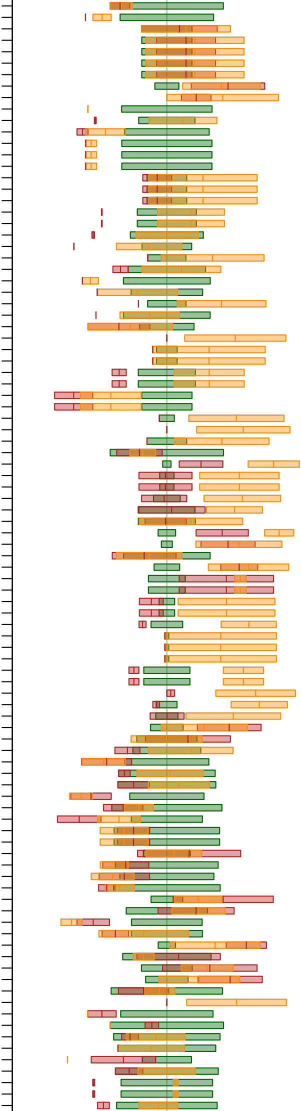

ESV05161 (0.006%)	0.6	0.57		– / –
ESV05343 (0.008%)	2.4	0.17		– / –
ESV06227 (0.002%)	0.1	0.93		N / –
o:Legionellales (0.01%)	0.2	0.86		– / –
f:Coxiellaceae (0.01%)	0.2	0.86		– / –
g:Aquicella (0.01%)	0.2	0.86		– / –
g:Aquicella_uncl (0.01%)	0.2	0.86		– / –
ESV04227 (0.006%)	1.1	0.38		– / –
ESV07349 (0.003%)	0.7	0.53		– / –
ESV07371 (0.002%)	3.0	0.12		N / –
o:Pseudomonadales (0.12%)	3.4	0.10		– / –
f:Moraxellaceae (0.01%)	2.6	0.16		– / –
g:Acinetobacter (0.007%)	3.0	0.13		N / –
s_Acinetobacter_lwoffii (0.007%)	3.0	0.13		N / –
ESV01771 (0.007%)	3.0	0.13		N / –
g:Perlucidibaca (0.004%)	0.5	0.63		– / –
g:Perlucidibaca_uncl (0.004%)	0.5	0.63		– / –
ESV07405 (0.004%)	0.5	0.63		– / –
f:Pseudomonadaceae (0.11%)	3.2	0.11		– / –
g:Pseudomonas (0.11%)	3.2	0.11		– / –
g:Pseudomonas_uncl (0.07%)	2.4	0.17		– / –
ESV00715 (0.02%)	4.3	0.07	.	– / –
ESV01975 (0.008%)	1.1	0.39		– / –
ESV03535 (0.008%)	1.0	0.43		– / –
ESV03676 (0.005%)	3.3	0.11		– / –
ESV03740 (0.005%)	1.3	0.35		– / –
ESV04359 (0.007%)	2.3	0.18		– / –
ESV04881 (0.01%)	1.5	0.30		– / –
ESV04952 (0.005%)	0.5	0.61		N / –
ESV06060 (0.002%)	1.8	0.24		N / –
s_Pseudomonas_alcaligenes (0.01%)	0.8	0.50		N / –
ESV01251 (0.01%)	0.8	0.50		N / –
s_Pseudomonas_jessenii (0.009%)	2.9	0.13		– / –
ESV01874 (0.009%)	2.9	0.13		– / –
s_Pseudomonas_koreensis (0.007%)	3.4	0.10		– / –
ESV05919 (0.007%)	3.4	0.10		– / –
s_Pseudomonas_resinovorans (0.01%)	2.3	0.18		N / –
ESV03455 (0.006%)	2.7	0.15		N / –
ESV05981 (0.004%)	1.7	0.26		– / –
o:Xanthomonadales (3.15%)	0.2	0.85		– / –
f:Nevskiaceae (0.06%)	7.8	0.02	*	– / –
g:Alkanibacter (0.02%)	2.3	0.18		– / –
g:Alkanibacter_uncl (0.02%)	2.3	0.18		– / –
ESV02346 (0.007%)	2.7	0.14		– / –
ESV03061 (0.007%)	1.6	0.28		– / –
ESV07137 (0.007%)	0.2	0.86		– / –
g:Nevskia (0.04%)	9.8	0.01	*	– / –
g:Nevskia_uncl (0.01%)	1.7	0.26		– / –
ESV04869 (0.003%)	0.1	0.90		– / –
ESV07394 (0.007%)	2.8	0.14		– / –
s_Nevskia_terrae (0.02%)	1.8	0.25		– / –
ESV02086 (0.02%)	1.8	0.25		– / –
s_Persicaria_minor (0.009%)	1.9	0.24		– / –
ESV04552 (0.009%)	1.9	0.24		– / –
f:Solimonadaceae (0.11%)	9.1	0.02	*	– / –
f:Solimonadaceae_uncl (0.01%)	1.0	0.43		N / –
f:Solimonadaceae_uncl (0.01%)	1.0	0.43		N / –
ESV01923 (0.01%)	1.0	0.43		N / –
g:Polycyclovorans (0.1%)	9.8	0.01	*	– / –
g:Polycyclovorans_uncl (0.1%)	9.8	0.01	*	– / –
ESV01536 (0.01%)	4.7	0.06	.	N / –
ESV01972 (0.02%)	10.6	0.01	*	– / –
ESV02520 (0.01%)	1.7	0.27		– / –
ESV02845 (0.008%)	0.9	0.44		– / –
ESV02930 (0.009%)	0.1	0.89		– / –
ESV03803 (0.005%)	1.0	0.43		– / –
ESV04052 (0.006%)	1.3	0.33		– / –
ESV05521 (0.007%)	0.6	0.60		– / –
ESV05620 (0.004%)	0.5	0.65		– / –
ESV07267 (0.006%)	3.4	0.10		– / –
ESV07323 (0.01%)	0.4	0.68		– / –
f:Xanthomonadaceae (0.71%)	2.6	0.15		– / –
f:Xanthomonadaceae_uncl (0.22%)	0.4	0.68		– / –
f:Xanthomonadaceae_uncl (0.22%)	0.4	0.68		– / –
ESV00607 (0.03%)	0.1	0.91		– / –
ESV00721 (0.03%)	0.7	0.54		– / –
ESV00921 (0.02%)	0.7	0.52		– / –
ESV01179 (0.02%)	1.0	0.43		– / –
ESV01673 (0.01%)	0.7	0.52		– / –
ESV01998 (0.02%)	0.2	0.79		– / –
ESV02025 (0.01%)	4.6	0.06	.	– / –
ESV02530 (0.02%)	0.6	0.59		– / –
ESV02782 (0.008%)	1.9	0.23		– / –
ESV02795 (0.005%)	0.2	0.79		– / –
ESV03201 (0.007%)	0.5	0.64		– / –
ESV03372 (0.008%)	1.0	0.43		– / –
ESV04913 (0.01%)	0.1	0.90		– / –
ESV04926 (0.004%)	1.9	0.22		N / –
ESV05483 (0.002%)	2.3	0.18		– / –
ESV05547 (0.004%)	0.8	0.49		N / –
ESV05566 (0.006%)	0.3	0.76		– / –
ESV06497 (0.002%)	0.5	0.61		– / –
ESV06575 (0.006%)	4.5	0.06	.	– / –
ESV06806 (0.003%)	0.4	0.70		– / –
g:Arenimonas (0.05%)	2.9	0.13		N / –
g:Arenimonas_uncl (0.05%)	2.9	0.13		N / –
ESV01124 (0.03%)	1.2	0.36		– / –

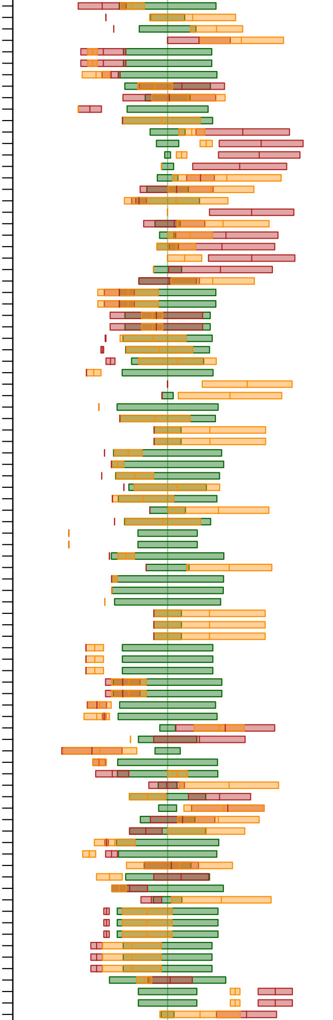

ESV03827 (0.007%)	1.0	0.41		− / −
ESV04905 (0.007%)	2.8	0.14		− / −
ESV06700 (0.007%)	5.3	0.05	*	− / −
ESV07551 (0.004%)	1.5	0.30		− / −
g:Dokdonella (0.03%)	2.0	0.22		− / −
g:Dokdonella_uncl (0.03%)	2.0	0.22		− / −
ESV02605 (0.009%)	1.6	0.28		− / −
ESV03850 (0.006%)	0.1	0.88		− / −
ESV04444 (0.003%)	0.1	0.91		− / −
ESV04779 (0.01%)	4.0	0.08	.	− / −
ESV06725 (0.002%)	0.5	0.63		N / −
g:Dyella (0.09%)	1.7	0.27		− / −
g:Dyella_uncl (0.05%)	3.4	0.10		− / −
ESV00769 (0.02%)	4.3	0.07	.	N / −
ESV02849 (0.006%)	2.5	0.16		N / −
ESV03121 (0.005%)	0.8	0.49		− / −
ESV05157 (0.008%)	0.4	0.67		− / −
ESV05652 (0.006%)	0.3	0.75		− / −
ESV05839 (0.002%)	4.0	0.08	.	N / −
ESV06284 (0.006%)	0.9	0.47		− / −
s_Dyella_japonica (0.02%)	0.8	0.49		− / −
ESV04901 (0.004%)	0.8	0.50		− / −
ESV04962 (0.003%)	2.7	0.14		− / −
ESV06522 (0.003%)	1.3	0.34		− / −
ESV06993 (0.006%)	0.6	0.60		− / −
s_Dyella_koreensis (0.01%)	0.6	0.59		− / −
ESV01277 (0.01%)	0.6	0.59		− / −
s_Dyella_marensis (0.007%)	0.0	0.95		− / −
ESV05586 (0.007%)	0.0	0.95		− / −
g:Lysobacter (0.13%)	1.0	0.41		− / −
g:Lysobacter_uncl (0.04%)	1.3	0.33		− / −
ESV01622 (0.01%)	1.4	0.32		− / −
ESV03072 (0.01%)	2.8	0.13		N / −
ESV03214 (0.004%)	3.1	0.12		N / −
ESV03420 (0.006%)	1.6	0.28		N / −
ESV04856 (0.004%)	1.9	0.24		N / −
ESV05407 (0.003%)	0.5	0.62		− / −
s_Lysobacter_brunescens (0.01%)	0.8	0.50		− / −
ESV01075 (0.01%)	0.8	0.50		− / −
s_Lysobacter_dokdonensis (0.02%)	1.0	0.43		− / −
ESV03852 (0.004%)	0.9	0.46		N / −
ESV05671 (0.01%)	1.1	0.40		− / −
s_Lysobacter_soli (0.03%)	0.7	0.52		− / −
ESV01902 (0.01%)	0.7	0.54		− / −
ESV02439 (0.009%)	1.3	0.33		− / −
ESV05142 (0.006%)	0.8	0.50		− / −
s_Lysobacter_ximonensis (0.003%)	11.1	0.010	**	N / −
ESV07169 (0.003%)	11.1	0.010	**	N / −
s_Lysobacter_yangpyeongensis (0.03%)	0.8	0.48		N / −
ESV02232 (0.007%)	2.5	0.17		− / −
ESV02412 (0.01%)	1.0	0.44		N / −
ESV02424 (0.005%)	1.0	0.42		N / −
ESV04349 (0.004%)	1.4	0.32		N / −
g:Mizugakiibacter (0.005%)	0.8	0.51		− / −
g:Mizugakiibacter_uncl (0.005%)	0.8	0.51		− / −
ESV05224 (0.005%)	0.8	0.51		− / −
g:Pseudoxanthomonas (0.004%)	2.8	0.14		− / −
s_Pseudoxanthomonas_mexicana (0.004%)	2.8	0.14		− / −
ESV04876 (0.004%)	2.8	0.14		− / −
g:Rhodanobacter (0.16%)	0.5	0.63		− / −
g:Rhodanobacter_uncl (0.16%)	0.5	0.63		− / −
ESV00846 (0.02%)	1.9	0.23		− / −
ESV00910 (0.02%)	1.7	0.25		− / −
ESV01288 (0.01%)	1.0	0.43		− / −
ESV02360 (0.009%)	1.2	0.36		− / −
ESV02574 (0.01%)	2.1	0.20		− / −
ESV02637 (0.006%)	1.8	0.24		− / −
ESV02728 (0.01%)	1.3	0.34		− / −
ESV03183 (0.01%)	1.3	0.33		− / −
ESV03193 (0.007%)	1.5	0.30		− / −
ESV03385 (0.008%)	1.1	0.38		− / −
ESV03874 (0.01%)	0.6	0.58		− / −
ESV05270 (0.003%)	0.9	0.46		− / −
ESV05356 (0.007%)	1.1	0.40		− / −
ESV05500 (0.009%)	2.0	0.22		− / −
ESV05762 (0.004%)	0.0	0.97		− / −
ESV06367 (0.007%)	1.6	0.27		− / −
ESV06520 (0.005%)	0.6	0.60		− / −
ESV06652 (0.005%)	1.4	0.31		− / −
g:Rudaea (0.008%)	0.9	0.45		− / −
s_Rudaea_cellulosilytica (0.008%)	0.9	0.45		− / −
ESV05694 (0.008%)	0.9	0.45		− / −
g:Stenotrophomonas (0.01%)	1.3	0.34		− / −
s_Stenotrophomonas_maltophilia (0.01%)	1.3	0.34		− / −
ESV03055 (0.01%)	1.3	0.34		− / −
f:Xanthomonadales_ince_sedi (2.25%)	0.2	0.86		− / −
f:Xanthomonadales_ince_sedi (0.09%)	7.6	0.02	*	− / −
f:Xanthomonadales_ince_sedi (0.09%)	7.6	0.02	*	− / −
ESV00685 (0.02%)	1.8	0.24		− / −

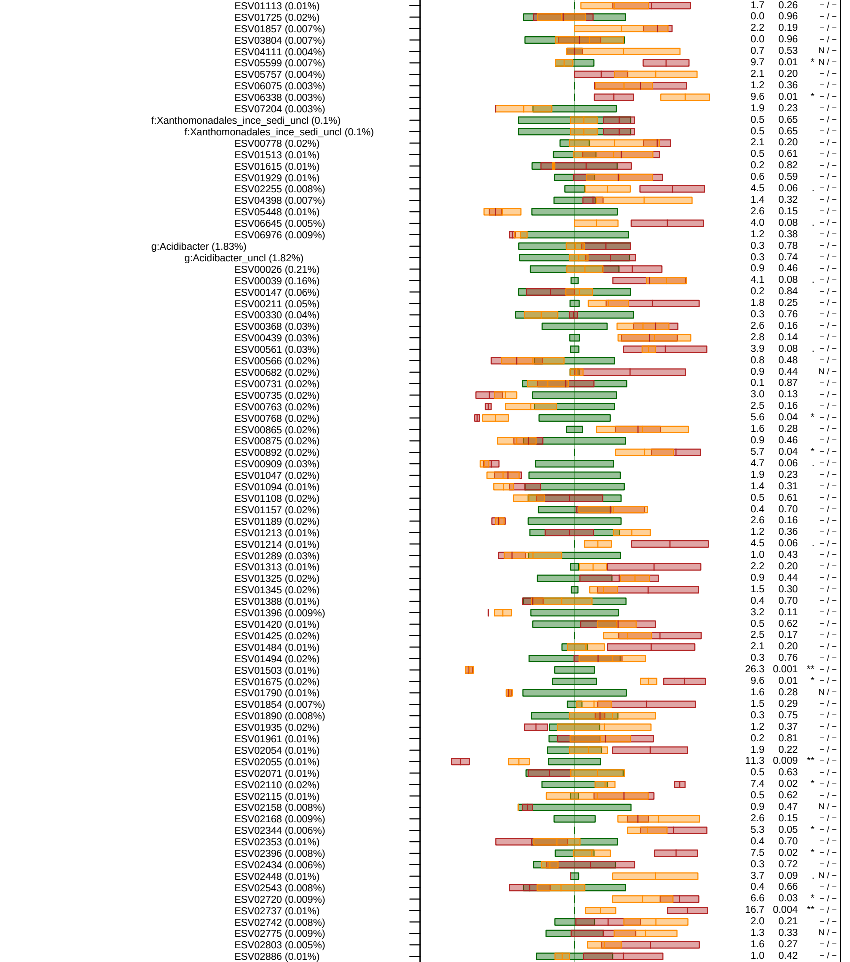
ESV01113 (0.01%) 1.7 0.26 - / -
ESV01725 (0.02%) 0.0 0.96 - / -
ESV01857 (0.007%) 2.2 0.19 - / -
ESV03804 (0.007%) 0.0 0.96 - / -
ESV04111 (0.004%) 0.7 0.53 N / -
ESV05599 (0.007%) 9.7 0.01 * N / -
ESV05757 (0.004%) 2.1 0.20 - / -
ESV06075 (0.003%) 1.2 0.36 - / -
ESV06338 (0.003%) 9.6 0.01 * - / -
ESV07204 (0.003%) 1.9 0.23 - / -
f:Xanthomonadales_ince_sedi_uncl (0.1%) 0.5 0.65 - / -
f:Xanthomonadales_ince_sedi_uncl (0.1%) 0.5 0.65 - / -
ESV00778 (0.02%) 2.1 0.20 - / -
ESV01513 (0.01%) 0.5 0.61 - / -
ESV01615 (0.01%) 0.2 0.82 - / -
ESV01929 (0.01%) 0.6 0.59 - / -
ESV02255 (0.008%) 4.5 0.06 . - / -
ESV04398 (0.007%) 1.4 0.32 - / -
ESV05448 (0.01%) 2.6 0.15 - / -
ESV06645 (0.005%) 4.0 0.08 . - / -
ESV06976 (0.009%) 1.2 0.38 - / -
g:Acidibacter (1.83%) 0.3 0.78 - / -
g:Acidibacter_uncl (1.82%) 0.3 0.74 - / -
ESV00026 (0.21%) 0.9 0.46 - / -
ESV00039 (0.16%) 4.1 0.08 . - / -
ESV00147 (0.06%) 0.2 0.84 - / -
ESV00211 (0.05%) 1.8 0.25 - / -
ESV00330 (0.04%) 0.3 0.76 - / -
ESV00368 (0.03%) 2.6 0.16 - / -
ESV00439 (0.03%) 2.8 0.14 - / -
ESV00561 (0.03%) 3.9 0.08 . - / -
ESV00566 (0.02%) 0.8 0.48 - / -
ESV00682 (0.02%) 0.9 0.44 N / -
ESV00731 (0.02%) 0.1 0.87 - / -
ESV00735 (0.02%) 3.0 0.13 - / -
ESV00763 (0.02%) 2.5 0.16 - / -
ESV00768 (0.02%) 5.6 0.04 * - / -
ESV00865 (0.02%) 1.6 0.28 - / -
ESV00875 (0.02%) 0.9 0.46 - / -
ESV00892 (0.02%) 5.7 0.04 * - / -
ESV00909 (0.03%) 4.7 0.06 . - / -
ESV01047 (0.02%) 1.9 0.23 - / -
ESV01094 (0.01%) 1.4 0.31 - / -
ESV01108 (0.02%) 0.5 0.61 - / -
ESV01157 (0.02%) 0.4 0.70 - / -
ESV01189 (0.02%) 2.6 0.16 - / -
ESV01213 (0.01%) 1.2 0.36 - / -
ESV01214 (0.01%) 4.5 0.06 . - / -
ESV01289 (0.03%) 1.0 0.43 - / -
ESV01313 (0.01%) 2.2 0.20 - / -
ESV01325 (0.02%) 0.9 0.44 - / -
ESV01345 (0.02%) 1.5 0.30 - / -
ESV01388 (0.01%) 0.4 0.70 - / -
ESV01396 (0.009%) 3.2 0.11 - / -
ESV01420 (0.01%) 0.5 0.62 - / -
ESV01425 (0.02%) 2.5 0.17 - / -
ESV01484 (0.01%) 2.1 0.20 - / -
ESV01494 (0.02%) 0.3 0.76 - / -
ESV01503 (0.01%) 26.3 0.001 ** - / -
ESV01675 (0.02%) 9.6 0.01 * - / -
ESV01790 (0.01%) 1.6 0.28 N / -
ESV01854 (0.007%) 1.5 0.29 - / -
ESV01890 (0.008%) 0.3 0.75 - / -
ESV01935 (0.02%) 1.2 0.37 - / -
ESV01961 (0.01%) 0.2 0.81 - / -
ESV02054 (0.01%) 1.9 0.22 - / -
ESV02055 (0.01%) 11.3 0.009 ** - / -
ESV02071 (0.01%) 0.5 0.63 - / -
ESV02110 (0.02%) 7.4 0.02 * - / -
ESV02115 (0.01%) 0.5 0.62 - / -
ESV02158 (0.008%) 0.9 0.47 N / -
ESV02168 (0.009%) 2.6 0.15 - / -
ESV02344 (0.006%) 5.3 0.05 * - / -
ESV02353 (0.01%) 0.4 0.70 - / -
ESV02396 (0.008%) 7.5 0.02 * - / -
ESV02434 (0.006%) 0.3 0.72 - / -
ESV02448 (0.01%) 3.7 0.09 . N / -
ESV02543 (0.008%) 0.4 0.66 - / -
ESV02720 (0.009%) 6.6 0.03 * - / -
ESV02737 (0.01%) 16.7 0.004 ** - / -
ESV02742 (0.008%) 2.0 0.21 - / -
ESV02775 (0.009%) 1.3 0.33 N / -
ESV02803 (0.005%) 1.6 0.27 - / -
ESV02886 (0.01%) 1.0 0.42 - / -

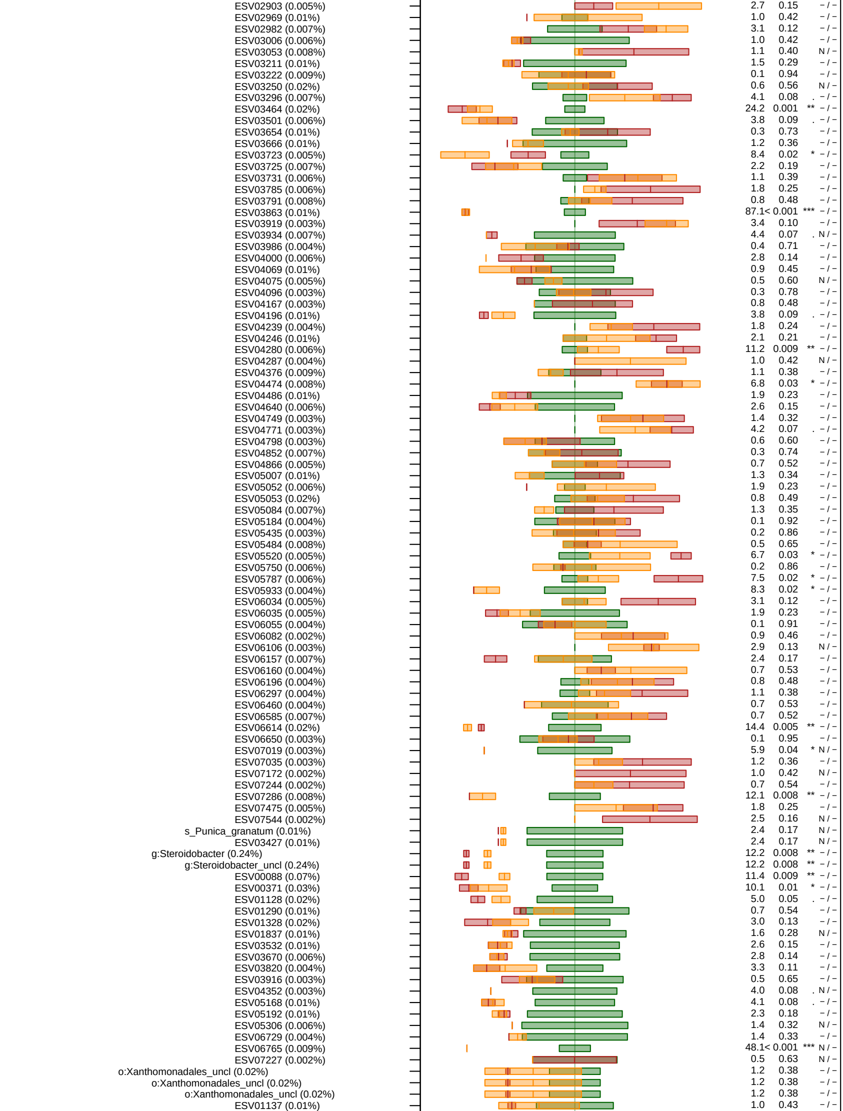

ESV02903 (0.005%) 2.7 0.15 – / –
ESV02969 (0.01%) 1.0 0.42 – / –
ESV02982 (0.007%) 3.1 0.12 – / –
ESV03006 (0.006%) 1.0 0.42 – / –
ESV03053 (0.008%) 1.1 0.40 N / –
ESV03211 (0.01%) 1.5 0.29 – / –
ESV03222 (0.009%) 0.1 0.94 – / –
ESV03250 (0.02%) 0.6 0.56 N / –
ESV03296 (0.007%) 4.1 0.08 . – / –
ESV03464 (0.02%) 24.2 0.001 ** – / –
ESV03501 (0.006%) 3.8 0.09 . – / –
ESV03654 (0.01%) 0.3 0.73 – / –
ESV03666 (0.01%) 1.2 0.36 – / –
ESV03723 (0.005%) 8.4 0.02 * – / –
ESV03725 (0.007%) 2.2 0.19 – / –
ESV03731 (0.006%) 1.1 0.39 – / –
ESV03785 (0.006%) 1.8 0.25 – / –
ESV03791 (0.008%) 0.8 0.48 – / –
ESV03863 (0.01%) 87.1 < 0.001 *** – / –
ESV03919 (0.003%) 3.4 0.10 – / –
ESV03934 (0.007%) 4.4 0.07 . N / –
ESV03986 (0.004%) 0.4 0.71 – / –
ESV04000 (0.006%) 2.8 0.14 – / –
ESV04069 (0.01%) 0.9 0.45 – / –
ESV04075 (0.005%) 0.5 0.60 N / –
ESV04096 (0.003%) 0.3 0.78 – / –
ESV04167 (0.003%) 0.8 0.48 – / –
ESV04196 (0.01%) 3.8 0.09 . – / –
ESV04239 (0.004%) 1.8 0.24 – / –
ESV04246 (0.01%) 2.1 0.21 – / –
ESV04280 (0.006%) 11.2 0.009 ** – / –
ESV04287 (0.004%) 1.0 0.42 N / –
ESV04376 (0.009%) 1.1 0.38 – / –
ESV04474 (0.008%) 6.8 0.03 * – / –
ESV04486 (0.01%) 1.9 0.23 – / –
ESV04640 (0.006%) 2.6 0.15 – / –
ESV04749 (0.003%) 1.4 0.32 – / –
ESV04771 (0.003%) 4.2 0.07 . – / –
ESV04798 (0.003%) 0.6 0.60 – / –
ESV04852 (0.007%) 0.3 0.74 – / –
ESV04866 (0.005%) 0.7 0.52 – / –
ESV05007 (0.01%) 1.3 0.34 – / –
ESV05052 (0.006%) 1.9 0.23 – / –
ESV05053 (0.02%) 0.8 0.49 – / –
ESV05084 (0.007%) 1.3 0.35 – / –
ESV05184 (0.004%) 0.1 0.92 – / –
ESV05435 (0.003%) 0.2 0.86 – / –
ESV05484 (0.008%) 0.5 0.65 – / –
ESV05520 (0.005%) 6.7 0.03 * – / –
ESV05750 (0.006%) 0.2 0.86 – / –
ESV05787 (0.006%) 7.5 0.02 * – / –
ESV05933 (0.004%) 8.3 0.02 * – / –
ESV06034 (0.005%) 3.1 0.12 – / –
ESV06035 (0.005%) 1.9 0.23 – / –
ESV06055 (0.004%) 0.1 0.91 – / –
ESV06082 (0.002%) 0.9 0.46 – / –
ESV06106 (0.003%) 2.9 0.13 N / –
ESV06157 (0.007%) 2.4 0.17 – / –
ESV06160 (0.004%) 0.7 0.53 – / –
ESV06196 (0.004%) 0.8 0.48 – / –
ESV06297 (0.004%) 1.1 0.38 – / –
ESV06460 (0.004%) 0.7 0.53 – / –
ESV06585 (0.007%) 0.7 0.52 – / –
ESV06614 (0.02%) 14.4 0.005 ** – / –
ESV06650 (0.003%) 0.1 0.95 – / –
ESV07019 (0.003%) 5.9 0.04 * N / –
ESV07035 (0.003%) 1.2 0.36 – / –
ESV07172 (0.002%) 1.0 0.42 N / –
ESV07244 (0.002%) 0.7 0.54 – / –
ESV07286 (0.008%) 12.1 0.008 ** – / –
ESV07475 (0.005%) 1.8 0.25 – / –
ESV07544 (0.002%) 2.5 0.16 N / –
s_Punica_granatum (0.01%) 2.4 0.17 N / –
ESV03427 (0.01%) 2.4 0.17 N / –
g:Steroidobacter (0.24%) 12.2 0.008 ** – / –
g:Steroidobacter_uncl (0.24%) 12.2 0.008 ** – / –
ESV00088 (0.07%) 11.4 0.009 ** – / –
ESV00371 (0.03%) 10.1 0.01 * – / –
ESV01128 (0.02%) 5.0 0.05 . – / –
ESV01290 (0.01%) 0.7 0.54 – / –
ESV01328 (0.02%) 3.0 0.13 – / –
ESV01837 (0.01%) 1.6 0.28 N / –
ESV03532 (0.01%) 2.6 0.15 – / –
ESV03670 (0.006%) 2.8 0.14 – / –
ESV03820 (0.004%) 3.3 0.11 – / –
ESV03916 (0.003%) 0.5 0.65 – / –
ESV04352 (0.003%) 4.0 0.08 . N / –
ESV05168 (0.01%) 4.1 0.08 . – / –
ESV05192 (0.01%) 2.3 0.18 – / –
ESV05306 (0.006%) 1.4 0.32 N / –
ESV06729 (0.004%) 1.4 0.33 – / –
ESV06765 (0.009%) 48.1 < 0.001 *** N / –
ESV07227 (0.002%) 0.5 0.63 N / –
o:Xanthomonadales_uncl (0.02%) 1.2 0.38 – / –
o:Xanthomonadales_uncl (0.02%) 1.2 0.38 – / –
o:Xanthomonadales_uncl (0.02%) 1.2 0.38 – / –
ESV01137 (0.01%) 1.0 0.43 – / –

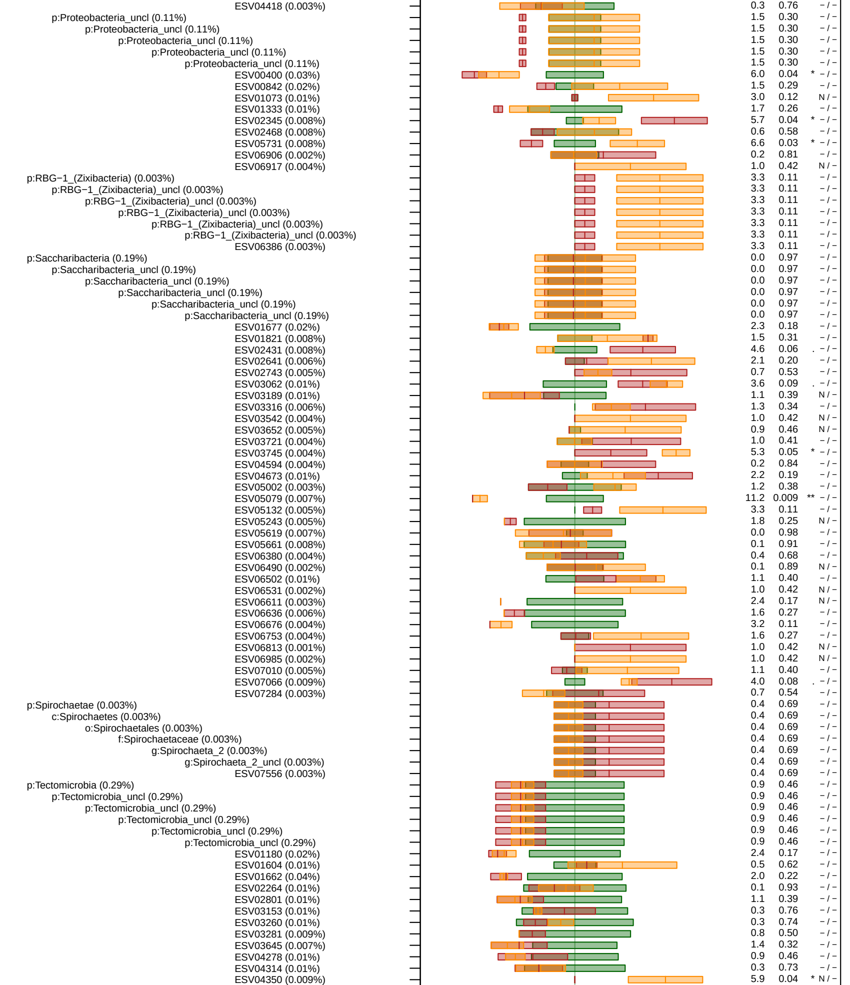
ESV04418 (0.003%) 0.3 0.76 - / -
p:Proteobacteria_uncl (0.11%) 1.5 0.30 - / -
p:Proteobacteria_uncl (0.11%) 1.5 0.30 - / -
p:Proteobacteria_uncl (0.11%) 1.5 0.30 - / -
p:Proteobacteria_uncl (0.11%) 1.5 0.30 - / -
p:Proteobacteria_uncl (0.11%) 1.5 0.30 - / -
ESV00400 (0.03%) 6.0 0.04 * - / -
ESV00842 (0.02%) 1.5 0.29 - / -
ESV01073 (0.01%) 3.0 0.12 N / -
ESV01333 (0.01%) 1.7 0.26 - / -
ESV02345 (0.008%) 5.7 0.04 * - / -
ESV02468 (0.008%) 0.6 0.58 - / -
ESV05731 (0.008%) 6.6 0.03 * - / -
ESV06906 (0.002%) 0.2 0.81 - / -
ESV06917 (0.004%) 1.0 0.42 N / -
p:RBG−1_(Zixibacteria) (0.003%) 3.3 0.11 - / -
p:RBG−1_(Zixibacteria)_uncl (0.003%) 3.3 0.11 - / -
p:RBG−1_(Zixibacteria)_uncl (0.003%) 3.3 0.11 - / -
p:RBG−1_(Zixibacteria)_uncl (0.003%) 3.3 0.11 - / -
p:RBG−1_(Zixibacteria)_uncl (0.003%) 3.3 0.11 - / -
p:RBG−1_(Zixibacteria)_uncl (0.003%) 3.3 0.11 - / -
ESV06386 (0.003%) 3.3 0.11 - / -
p:Saccharibacteria (0.19%) 0.0 0.97 - / -
p:Saccharibacteria_uncl (0.19%) 0.0 0.97 - / -
p:Saccharibacteria_uncl (0.19%) 0.0 0.97 - / -
p:Saccharibacteria_uncl (0.19%) 0.0 0.97 - / -
p:Saccharibacteria_uncl (0.19%) 0.0 0.97 - / -
p:Saccharibacteria_uncl (0.19%) 0.0 0.97 - / -
ESV01677 (0.02%) 2.3 0.18 - / -
ESV01821 (0.008%) 1.5 0.31 - / -
ESV02431 (0.008%) 4.6 0.06 . - / -
ESV02641 (0.006%) 2.1 0.20 - / -
ESV02743 (0.005%) 0.7 0.53 - / -
ESV03062 (0.01%) 3.6 0.09 . - / -
ESV03189 (0.01%) 1.1 0.39 N / -
ESV03316 (0.006%) 1.3 0.34 - / -
ESV03542 (0.004%) 1.0 0.42 N / -
ESV03652 (0.005%) 0.9 0.46 N / -
ESV03721 (0.004%) 1.0 0.41 - / -
ESV03745 (0.004%) 5.3 0.05 * - / -
ESV04594 (0.004%) 0.2 0.84 - / -
ESV04673 (0.01%) 2.2 0.19 - / -
ESV05002 (0.003%) 1.2 0.38 - / -
ESV05079 (0.007%) 11.2 0.009 ** - / -
ESV05132 (0.005%) 3.3 0.11 - / -
ESV05243 (0.005%) 1.8 0.25 N / -
ESV05619 (0.007%) 0.0 0.98 - / -
ESV05661 (0.008%) 0.1 0.91 - / -
ESV06380 (0.004%) 0.4 0.68 - / -
ESV06490 (0.002%) 0.1 0.89 N / -
ESV06502 (0.01%) 1.1 0.40 - / -
ESV06531 (0.002%) 1.0 0.42 N / -
ESV06611 (0.003%) 2.4 0.17 N / -
ESV06636 (0.006%) 1.6 0.27 - / -
ESV06676 (0.004%) 3.2 0.11 - / -
ESV06753 (0.004%) 1.6 0.27 - / -
ESV06813 (0.001%) 1.0 0.42 N / -
ESV06985 (0.002%) 1.0 0.42 N / -
ESV07010 (0.005%) 1.1 0.40 - / -
ESV07066 (0.009%) 4.0 0.08 . - / -
ESV07284 (0.003%) 0.7 0.54 - / -
p:Spirochaetae (0.003%) 0.4 0.69 - / -
c:Spirochaetes (0.003%) 0.4 0.69 - / -
o:Spirochaetales (0.003%) 0.4 0.69 - / -
f:Spirochaetaceae (0.003%) 0.4 0.69 - / -
g:Spirochaeta_2 (0.003%) 0.4 0.69 - / -
g:Spirochaeta_2_uncl (0.003%) 0.4 0.69 - / -
ESV07556 (0.003%) 0.4 0.69 - / -
p:Tectomicrobia (0.29%) 0.9 0.46 - / -
p:Tectomicrobia_uncl (0.29%) 0.9 0.46 - / -
p:Tectomicrobia_uncl (0.29%) 0.9 0.46 - / -
p:Tectomicrobia_uncl (0.29%) 0.9 0.46 - / -
p:Tectomicrobia_uncl (0.29%) 0.9 0.46 - / -
p:Tectomicrobia_uncl (0.29%) 0.9 0.46 - / -
ESV01180 (0.02%) 2.4 0.17 - / -
ESV01604 (0.01%) 0.5 0.62 - / -
ESV01662 (0.04%) 2.0 0.22 - / -
ESV02264 (0.01%) 0.1 0.93 - / -
ESV02801 (0.01%) 1.1 0.39 - / -
ESV03153 (0.01%) 0.3 0.76 - / -
ESV03260 (0.01%) 0.3 0.74 - / -
ESV03281 (0.009%) 0.8 0.50 - / -
ESV03645 (0.007%) 1.4 0.32 - / -
ESV04278 (0.01%) 0.9 0.46 - / -
ESV04314 (0.01%) 0.3 0.73 - / -
ESV04350 (0.009%) 5.9 0.04 * N / -

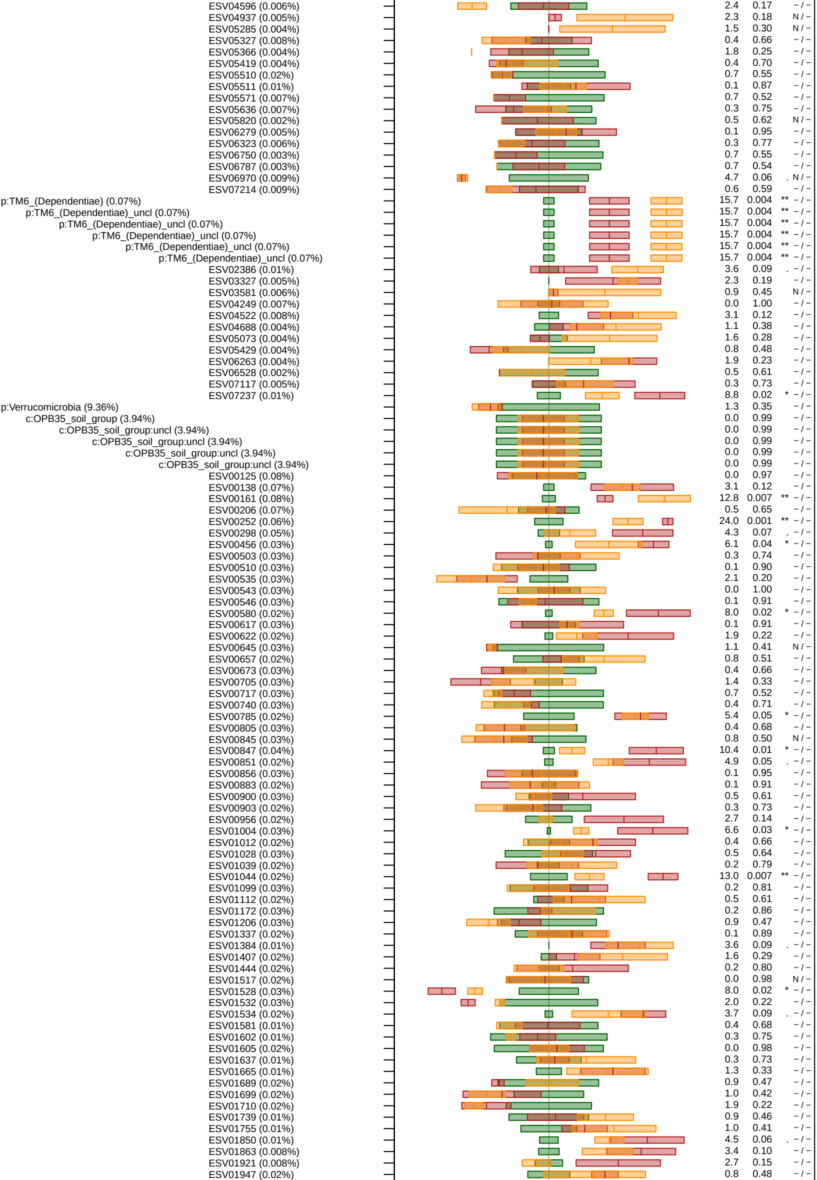

ESV04596 (0.006%) 2.4 0.17 – / –
ESV04937 (0.005%) 2.3 0.18 N / –
ESV05285 (0.004%) 1.5 0.30 N / –
ESV05327 (0.008%) 0.4 0.66 – / –
ESV05366 (0.004%) 1.8 0.25 – / –
ESV05419 (0.004%) 0.4 0.70 – / –
ESV05510 (0.02%) 0.7 0.55 – / –
ESV05511 (0.01%) 0.1 0.87 – / –
ESV05571 (0.007%) 0.7 0.52 – / –
ESV05636 (0.007%) 0.3 0.75 – / –
ESV05820 (0.002%) 0.5 0.62 N / –
ESV06279 (0.005%) 0.1 0.95 – / –
ESV06323 (0.006%) 0.3 0.77 – / –
ESV06750 (0.003%) 0.7 0.55 – / –
ESV06787 (0.003%) 0.7 0.54 – / –
ESV06970 (0.009%) 4.7 0.06 . N / –
ESV07214 (0.009%) 0.6 0.59 – / –
p:TM6_(Dependentiae) (0.07%) 15.7 0.004 ** – / –
p:TM6_(Dependentiae)_uncl (0.07%) 15.7 0.004 ** – / –
p:TM6_(Dependentiae)_uncl (0.07%) 15.7 0.004 ** – / –
p:TM6_(Dependentiae)_uncl (0.07%) 15.7 0.004 ** – / –
p:TM6_(Dependentiae)_uncl (0.07%) 15.7 0.004 ** – / –
p:TM6_(Dependentiae)_uncl (0.07%) 15.7 0.004 ** – / –
ESV02386 (0.01%) 3.6 0.09 . – / –
ESV03327 (0.005%) 2.3 0.19 – / –
ESV03581 (0.006%) 0.9 0.45 N / –
ESV04249 (0.007%) 0.0 1.00 – / –
ESV04522 (0.008%) 3.1 0.12 – / –
ESV04688 (0.004%) 1.1 0.38 – / –
ESV05073 (0.004%) 1.6 0.28 – / –
ESV05429 (0.004%) 0.8 0.48 – / –
ESV06263 (0.004%) 1.9 0.23 – / –
ESV06528 (0.002%) 0.5 0.61 – / –
ESV07117 (0.005%) 0.3 0.73 – / –
ESV07237 (0.01%) 8.8 0.02 * – / –
p:Verrucomicrobia (9.36%) 1.3 0.35 – / –
c:OPB35_soil_group (3.94%) 0.0 0.99 – / –
c:OPB35_soil_group:uncl (3.94%) 0.0 0.99 – / –
c:OPB35_soil_group:uncl (3.94%) 0.0 0.99 – / –
c:OPB35_soil_group:uncl (3.94%) 0.0 0.99 – / –
c:OPB35_soil_group:uncl (3.94%) 0.0 0.99 – / –
ESV00125 (0.08%) 0.0 0.97 – / –
ESV00138 (0.07%) 3.1 0.12 – / –
ESV00161 (0.08%) 12.8 0.007 ** – / –
ESV00206 (0.07%) 0.5 0.65 – / –
ESV00252 (0.06%) 24.0 0.001 ** – / –
ESV00298 (0.05%) 4.3 0.07 . – / –
ESV00456 (0.03%) 6.1 0.04 * – / –
ESV00503 (0.03%) 0.3 0.74 – / –
ESV00510 (0.03%) 0.1 0.90 – / –
ESV00535 (0.03%) 2.1 0.20 – / –
ESV00543 (0.03%) 0.0 1.00 – / –
ESV00546 (0.03%) 0.1 0.91 – / –
ESV00580 (0.02%) 8.0 0.02 * – / –
ESV00617 (0.03%) 0.1 0.91 – / –
ESV00622 (0.02%) 1.9 0.22 – / –
ESV00645 (0.03%) 1.1 0.41 N / –
ESV00657 (0.02%) 0.8 0.51 – / –
ESV00673 (0.03%) 0.4 0.66 – / –
ESV00705 (0.03%) 1.4 0.33 – / –
ESV00717 (0.03%) 0.7 0.52 – / –
ESV00740 (0.03%) 0.4 0.71 – / –
ESV00785 (0.02%) 5.4 0.05 * – / –
ESV00805 (0.03%) 0.4 0.68 – / –
ESV00845 (0.03%) 0.8 0.50 N / –
ESV00847 (0.04%) 10.4 0.01 * – / –
ESV00851 (0.02%) 4.9 0.05 . – / –
ESV00856 (0.03%) 0.1 0.95 – / –
ESV00883 (0.02%) 0.1 0.91 – / –
ESV00900 (0.03%) 0.5 0.61 – / –
ESV00903 (0.02%) 0.3 0.73 – / –
ESV00956 (0.02%) 2.7 0.14 – / –
ESV01004 (0.03%) 6.6 0.03 * – / –
ESV01012 (0.02%) 0.4 0.66 – / –
ESV01028 (0.03%) 0.5 0.64 – / –
ESV01039 (0.02%) 0.2 0.79 – / –
ESV01044 (0.02%) 13.0 0.007 ** – / –
ESV01099 (0.03%) 0.2 0.81 – / –
ESV01112 (0.02%) 0.5 0.61 – / –
ESV01172 (0.03%) 0.2 0.86 – / –
ESV01206 (0.03%) 0.9 0.47 – / –
ESV01337 (0.02%) 0.1 0.89 – / –
ESV01384 (0.01%) 3.6 0.09 . – / –
ESV01407 (0.02%) 1.6 0.29 – / –
ESV01444 (0.02%) 0.2 0.80 – / –
ESV01517 (0.02%) 0.0 0.98 N / –
ESV01528 (0.03%) 8.0 0.02 * – / –
ESV01532 (0.03%) 2.0 0.22 – / –
ESV01534 (0.02%) 3.7 0.09 . – / –
ESV01581 (0.01%) 0.4 0.68 – / –
ESV01602 (0.01%) 0.3 0.75 – / –
ESV01605 (0.02%) 0.0 0.98 – / –
ESV01637 (0.01%) 0.3 0.73 – / –
ESV01665 (0.01%) 1.3 0.33 – / –
ESV01689 (0.02%) 0.9 0.47 – / –
ESV01699 (0.02%) 1.0 0.42 – / –
ESV01710 (0.02%) 1.9 0.22 – / –
ESV01739 (0.01%) 0.9 0.46 – / –
ESV01755 (0.01%) 1.0 0.41 – / –
ESV01850 (0.01%) 4.5 0.06 . – / –
ESV01863 (0.008%) 3.4 0.10 – / –
ESV01921 (0.008%) 2.7 0.15 – / –
ESV01947 (0.02%) 0.8 0.48 – / –

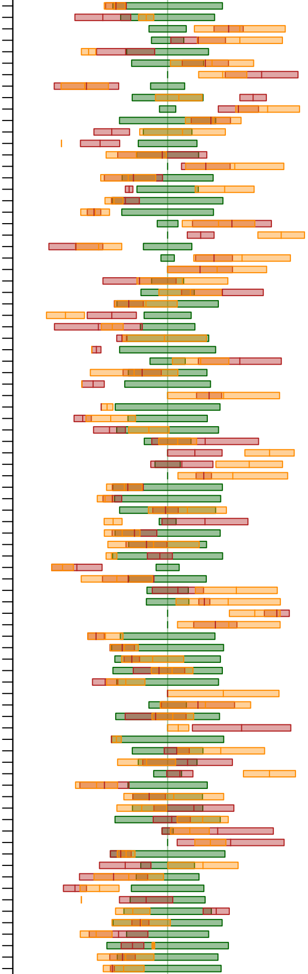

ESV01956 (0.02%)	0.8	0.47		– / –
ESV01979 (0.01%)	1.1	0.40		– / –
ESV02034 (0.01%)	1.7	0.26		– / –
ESV02036 (0.02%)	1.4	0.32		– / –
ESV02038 (0.02%)	1.8	0.24		– / –
ESV02042 (0.01%)	0.5	0.64		– / –
ESV02126 (0.02%)	3.5	0.10	.	– / –
ESV02133 (0.01%)	3.6	0.10	.	– / –
ESV02178 (0.008%)	3.3	0.11		– / –
ESV02215 (0.01%)	5.3	0.05	*	– / –
ESV02228 (0.03%)	0.6	0.59		– / –
ESV02258 (0.04%)	1.5	0.29		– / –
ESV02306 (0.009%)	6.9	0.03	*	– / –
ESV02383 (0.009%)	0.2	0.81		– / –
ESV02409 (0.01%)	1.1	0.38		– / –
ESV02489 (0.01%)	0.4	0.70		– / –
ESV02511 (0.02%)	3.8	0.09	.	– / –
ESV02521 (0.009%)	0.7	0.53		– / –
ESV02537 (0.007%)	2.3	0.18		– / –
ESV02617 (0.006%)	1.2	0.38		– / –
ESV02638 (0.01%)	14.2	0.005	**	– / –
ESV02668 (0.01%)	3.7	0.09	.	– / –
ESV02690 (0.01%)	1.6	0.29		– / –
ESV02735 (0.006%)	0.5	0.61		– / –
ESV02770 (0.01%)	0.4	0.69		– / –
ESV02810 (0.005%)	0.7	0.55		– / –
ESV02843 (0.01%)	0.3	0.75		– / –
ESV02855 (0.01%)	5.2	0.05	*	– / –
ESV02871 (0.01%)	1.4	0.31		– / –
ESV02895 (0.01%)	0.6	0.60		– / –
ESV02913 (0.01%)	2.3	0.18		N / –
ESV02920 (0.004%)	1.4	0.32		– / –
ESV02933 (0.01%)	0.2	0.79		– / –
ESV02939 (0.006%)	3.3	0.11		– / –
ESV02946 (0.005%)	0.8	0.50		– / –
ESV02949 (0.01%)	1.5	0.30		N / –
ESV02952 (0.02%)	2.5	0.16		– / –
ESV03022 (0.008%)	0.8	0.49		– / –
ESV03047 (0.01%)	0.3	0.75		– / –
ESV03096 (0.009%)	6.2	0.03	*	– / –
ESV03106 (0.02%)	2.5	0.16		– / –
ESV03116 (0.006%)	1.0	0.41		N / –
ESV03118 (0.01%)	0.5	0.64		– / –
ESV03159 (0.02%)	1.2	0.37		– / –
ESV03164 (0.006%)	0.1	0.90		– / –
ESV03208 (0.01%)	3.2	0.11		– / –
ESV03266 (0.009%)	0.5	0.65		– / –
ESV03267 (0.004%)	0.1	0.92		– / –
ESV03275 (0.02%)	0.9	0.47		– / –
ESV03297 (0.007%)	11.0	0.010	**	– / –
ESV03315 (0.01%)	0.6	0.57		– / –
ESV03346 (0.008%)	1.5	0.30		– / –
ESV03361 (0.01%)	0.9	0.47		– / –
ESV03382 (0.02%)	12.5	0.007	**	– / –
ESV03392 (0.008%)	1.0	0.42		– / –
ESV03400 (0.006%)	1.7	0.25		– / –
ESV03491 (0.006%)	0.6	0.59		– / –
ESV03503 (0.009%)	0.3	0.78		– / –
ESV03521 (0.01%)	0.0	0.97		– / –
ESV03526 (0.005%)	1.0	0.43		– / –
ESV03553 (0.006%)	1.0	0.42		N / –
ESV03565 (0.006%)	0.3	0.74		– / –
ESV03626 (0.008%)	0.1	0.94		– / –
ESV03635 (0.003%)	1.9	0.23		– / –
ESV03658 (0.01%)	0.9	0.45		N / –
ESV03675 (0.02%)	0.7	0.52		– / –
ESV03682 (0.01%)	0.3	0.73		– / –
ESV03739 (0.005%)	8.9	0.02	*	– / –
ESV03779 (0.02%)	1.7	0.25		– / –
ESV03813 (0.009%)	0.1	0.89		– / –
ESV03825 (0.009%)	0.6	0.58		– / –
ESV03860 (0.005%)	0.3	0.76		– / –
ESV03864 (0.007%)	0.5	0.61		– / –
ESV03901 (0.008%)	1.0	0.42		– / –
ESV03909 (0.007%)	0.6	0.59		– / –
ESV03922 (0.007%)	1.7	0.25		N / –
ESV03943 (0.008%)	0.7	0.54		– / –
ESV03949 (0.009%)	3.8	0.09	.	– / –
ESV03953 (0.005%)	2.9	0.13		– / –
ESV03990 (0.009%)	2.2	0.19		– / –
ESV04013 (0.008%)	0.2	0.84		– / –
ESV04082 (0.02%)	1.4	0.31		– / –
ESV04086 (0.007%)	0.2	0.79		– / –
ESV04095 (0.004%)	0.5	0.61		– / –
ESV04114 (0.007%)	0.8	0.50		– / –

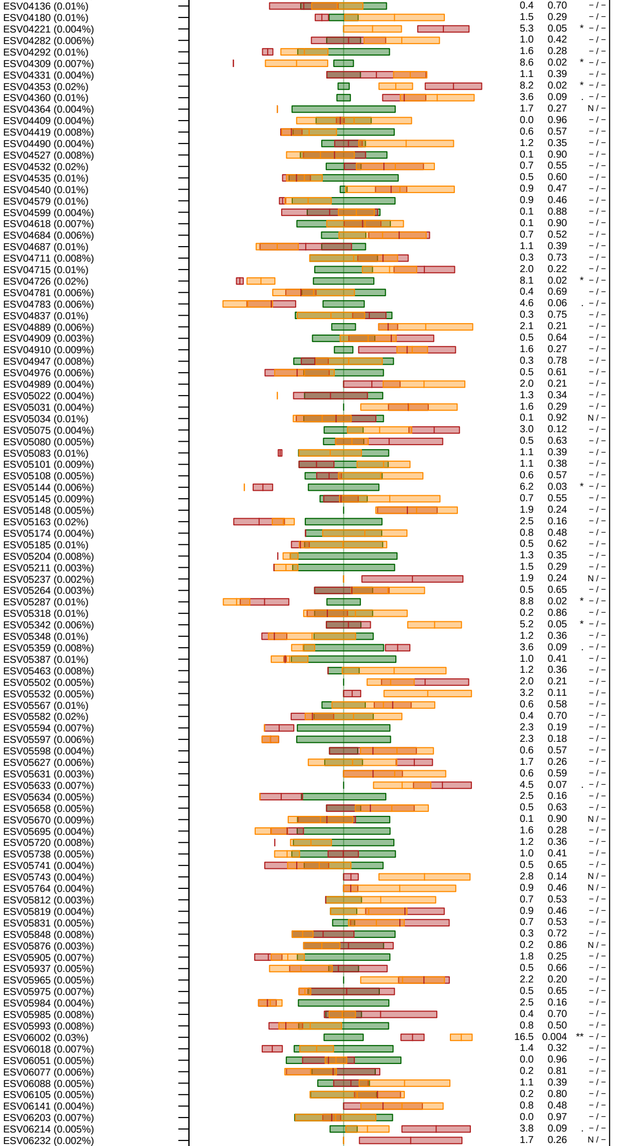

ESV04136 (0.01%) 0.4 0.70 – / –
ESV04180 (0.01%) 1.5 0.29 – / –
ESV04221 (0.004%) 5.3 0.05 * – / –
ESV04282 (0.006%) 1.0 0.42 – / –
ESV04292 (0.01%) 1.6 0.28 – / –
ESV04309 (0.007%) 8.6 0.02 * – / –
ESV04331 (0.004%) 1.1 0.39 – / –
ESV04353 (0.02%) 8.2 0.02 * – / –
ESV04360 (0.01%) 3.6 0.09 . – / –
ESV04364 (0.004%) 1.7 0.27 N / –
ESV04409 (0.004%) 0.0 0.96 – / –
ESV04419 (0.008%) 0.6 0.57 – / –
ESV04490 (0.004%) 1.2 0.35 – / –
ESV04527 (0.008%) 0.1 0.90 – / –
ESV04532 (0.02%) 0.7 0.55 – / –
ESV04535 (0.01%) 0.5 0.60 – / –
ESV04540 (0.01%) 0.9 0.47 – / –
ESV04579 (0.01%) 0.9 0.46 – / –
ESV04599 (0.004%) 0.1 0.88 – / –
ESV04618 (0.007%) 0.1 0.90 – / –
ESV04684 (0.006%) 0.7 0.52 – / –
ESV04687 (0.01%) 1.1 0.39 – / –
ESV04711 (0.008%) 0.3 0.73 – / –
ESV04715 (0.01%) 2.0 0.22 – / –
ESV04726 (0.02%) 8.1 0.02 * – / –
ESV04781 (0.006%) 0.4 0.69 – / –
ESV04783 (0.006%) 4.6 0.06 . – / –
ESV04837 (0.01%) 0.3 0.75 – / –
ESV04889 (0.006%) 2.1 0.21 – / –
ESV04909 (0.003%) 0.5 0.64 – / –
ESV04910 (0.009%) 1.6 0.27 – / –
ESV04947 (0.008%) 0.3 0.78 – / –
ESV04976 (0.006%) 0.5 0.61 – / –
ESV04989 (0.004%) 2.0 0.21 – / –
ESV05022 (0.004%) 1.3 0.34 – / –
ESV05031 (0.004%) 1.6 0.29 – / –
ESV05034 (0.01%) 0.1 0.92 N / –
ESV05075 (0.004%) 3.0 0.12 – / –
ESV05080 (0.005%) 0.5 0.63 – / –
ESV05083 (0.01%) 1.1 0.39 – / –
ESV05101 (0.009%) 1.1 0.38 – / –
ESV05108 (0.005%) 0.6 0.57 – / –
ESV05144 (0.006%) 6.2 0.03 * – / –
ESV05145 (0.009%) 0.7 0.55 – / –
ESV05148 (0.005%) 1.9 0.24 – / –
ESV05163 (0.02%) 2.5 0.16 – / –
ESV05174 (0.004%) 0.8 0.48 – / –
ESV05185 (0.01%) 0.5 0.62 – / –
ESV05204 (0.008%) 1.3 0.35 – / –
ESV05211 (0.003%) 1.5 0.29 – / –
ESV05237 (0.002%) 1.9 0.24 N / –
ESV05264 (0.003%) 0.5 0.65 – / –
ESV05287 (0.01%) 8.8 0.02 * – / –
ESV05318 (0.01%) 0.2 0.86 – / –
ESV05342 (0.006%) 5.2 0.05 * – / –
ESV05348 (0.01%) 1.2 0.36 – / –
ESV05359 (0.008%) 3.6 0.09 . – / –
ESV05387 (0.01%) 1.0 0.41 – / –
ESV05463 (0.008%) 1.2 0.36 – / –
ESV05502 (0.005%) 2.0 0.21 – / –
ESV05532 (0.005%) 3.2 0.11 – / –
ESV05567 (0.01%) 0.6 0.58 – / –
ESV05582 (0.02%) 0.4 0.70 – / –
ESV05594 (0.007%) 2.3 0.19 – / –
ESV05597 (0.006%) 2.3 0.18 – / –
ESV05598 (0.004%) 0.6 0.57 – / –
ESV05627 (0.006%) 1.7 0.26 – / –
ESV05631 (0.003%) 0.6 0.59 – / –
ESV05633 (0.007%) 4.5 0.07 . – / –
ESV05634 (0.005%) 2.5 0.16 – / –
ESV05658 (0.005%) 0.5 0.63 – / –
ESV05670 (0.009%) 0.1 0.90 N / –
ESV05695 (0.004%) 1.6 0.28 – / –
ESV05720 (0.008%) 1.2 0.36 – / –
ESV05738 (0.005%) 1.0 0.41 – / –
ESV05741 (0.004%) 0.5 0.65 – / –
ESV05743 (0.004%) 2.8 0.14 N / –
ESV05764 (0.004%) 0.9 0.46 N / –
ESV05812 (0.003%) 0.7 0.53 – / –
ESV05819 (0.004%) 0.9 0.46 – / –
ESV05831 (0.005%) 0.7 0.53 – / –
ESV05848 (0.008%) 0.3 0.72 – / –
ESV05876 (0.003%) 0.2 0.86 N / –
ESV05905 (0.007%) 1.8 0.25 – / –
ESV05937 (0.005%) 0.5 0.66 – / –
ESV05965 (0.005%) 2.2 0.20 – / –
ESV05975 (0.007%) 0.5 0.65 – / –
ESV05984 (0.004%) 2.5 0.16 – / –
ESV05985 (0.008%) 0.4 0.70 – / –
ESV05993 (0.008%) 0.8 0.50 – / –
ESV06002 (0.03%) 16.5 0.004 ** – / –
ESV06018 (0.007%) 1.4 0.32 – / –
ESV06051 (0.005%) 0.0 0.96 – / –
ESV06077 (0.006%) 0.2 0.81 – / –
ESV06088 (0.005%) 1.1 0.39 – / –
ESV06105 (0.005%) 0.2 0.80 – / –
ESV06141 (0.004%) 0.8 0.48 – / –
ESV06203 (0.007%) 0.0 0.97 – / –
ESV06214 (0.005%) 3.8 0.09 . – / –
ESV06232 (0.002%) 1.7 0.26 N / –

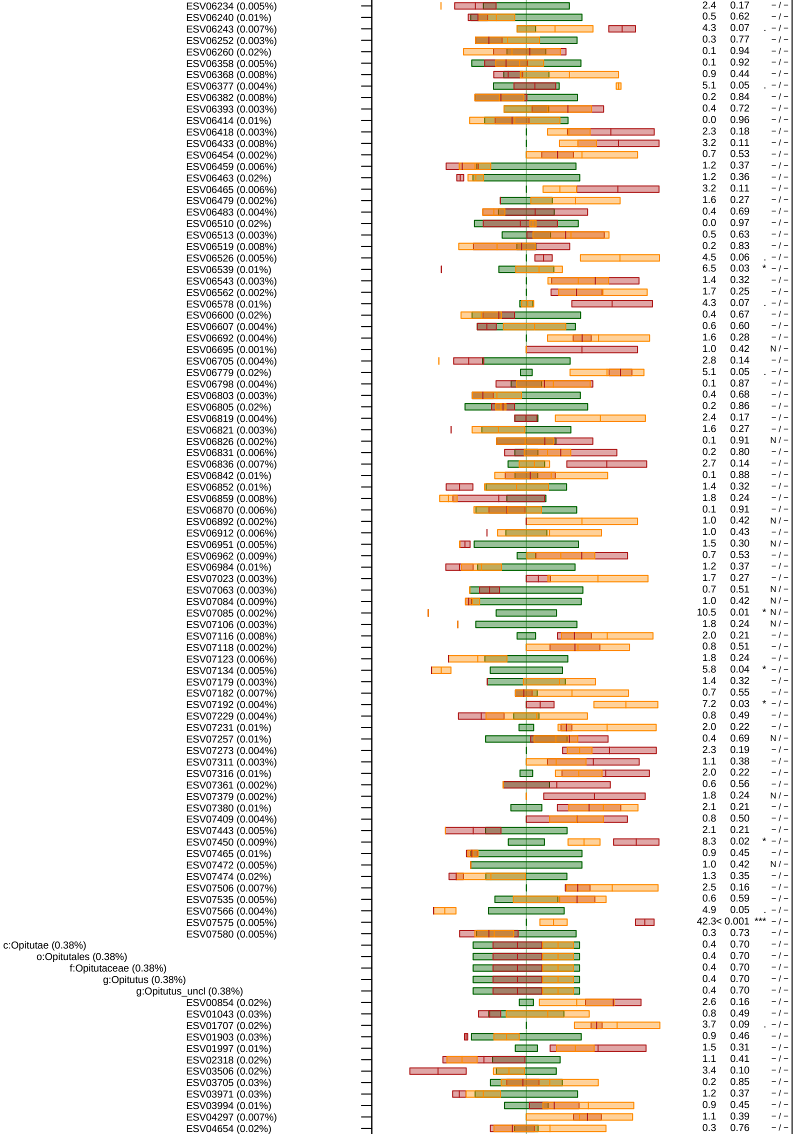

ESV06234 (0.005%) 2.4 0.17 – / –
ESV06240 (0.01%) 0.5 0.62 – / –
ESV06243 (0.007%) 4.3 0.07 . – / –
ESV06252 (0.003%) 0.3 0.77 – / –
ESV06260 (0.02%) 0.1 0.94 – / –
ESV06358 (0.005%) 0.1 0.92 – / –
ESV06368 (0.008%) 0.9 0.44 – / –
ESV06377 (0.004%) 5.1 0.05 . – / –
ESV06382 (0.008%) 0.2 0.84 – / –
ESV06393 (0.003%) 0.4 0.72 – / –
ESV06414 (0.01%) 0.0 0.96 – / –
ESV06418 (0.003%) 2.3 0.18 – / –
ESV06433 (0.008%) 3.2 0.11 – / –
ESV06454 (0.002%) 0.7 0.53 – / –
ESV06459 (0.006%) 1.2 0.37 – / –
ESV06463 (0.02%) 1.2 0.36 – / –
ESV06465 (0.006%) 3.2 0.11 – / –
ESV06479 (0.002%) 1.6 0.27 – / –
ESV06483 (0.004%) 0.4 0.69 – / –
ESV06510 (0.02%) 0.0 0.97 – / –
ESV06513 (0.003%) 0.5 0.63 – / –
ESV06519 (0.008%) 0.2 0.83 – / –
ESV06526 (0.005%) 4.5 0.06 . – / –
ESV06539 (0.01%) 6.5 0.03 * – / –
ESV06543 (0.003%) 1.4 0.32 – / –
ESV06562 (0.002%) 1.7 0.25 – / –
ESV06578 (0.01%) 4.3 0.07 . – / –
ESV06600 (0.02%) 0.4 0.67 – / –
ESV06607 (0.004%) 0.6 0.60 – / –
ESV06692 (0.004%) 1.6 0.28 – / –
ESV06695 (0.001%) 1.0 0.42 N / –
ESV06705 (0.004%) 2.8 0.14 – / –
ESV06779 (0.02%) 5.1 0.05 . – / –
ESV06798 (0.004%) 0.1 0.87 – / –
ESV06803 (0.003%) 0.4 0.68 – / –
ESV06805 (0.02%) 0.2 0.86 – / –
ESV06819 (0.004%) 2.4 0.17 – / –
ESV06821 (0.003%) 1.6 0.27 – / –
ESV06826 (0.002%) 0.1 0.91 N / –
ESV06831 (0.006%) 0.2 0.80 – / –
ESV06836 (0.007%) 2.7 0.14 – / –
ESV06842 (0.01%) 0.1 0.88 – / –
ESV06852 (0.01%) 1.4 0.32 – / –
ESV06859 (0.008%) 1.8 0.24 – / –
ESV06870 (0.006%) 0.1 0.91 – / –
ESV06892 (0.002%) 1.0 0.42 N / –
ESV06912 (0.006%) 1.0 0.43 – / –
ESV06951 (0.005%) 1.5 0.30 N / –
ESV06962 (0.009%) 0.7 0.53 – / –
ESV06984 (0.01%) 1.2 0.37 – / –
ESV07023 (0.003%) 1.7 0.27 – / –
ESV07063 (0.003%) 0.7 0.51 N / –
ESV07084 (0.009%) 1.0 0.42 N / –
ESV07085 (0.002%) 10.5 0.01 * N / –
ESV07106 (0.003%) 1.8 0.24 N / –
ESV07116 (0.008%) 2.0 0.21 – / –
ESV07118 (0.002%) 0.8 0.51 – / –
ESV07123 (0.006%) 1.8 0.24 – / –
ESV07134 (0.005%) 5.8 0.04 * – / –
ESV07179 (0.003%) 1.4 0.32 – / –
ESV07182 (0.007%) 0.7 0.55 – / –
ESV07192 (0.004%) 7.2 0.03 * – / –
ESV07229 (0.004%) 0.8 0.49 – / –
ESV07231 (0.01%) 2.0 0.22 – / –
ESV07257 (0.01%) 0.4 0.69 N / –
ESV07273 (0.004%) 2.3 0.19 – / –
ESV07311 (0.003%) 1.1 0.38 – / –
ESV07316 (0.01%) 2.0 0.22 – / –
ESV07361 (0.002%) 0.6 0.56 – / –
ESV07379 (0.002%) 1.8 0.24 N / –
ESV07380 (0.01%) 2.1 0.21 – / –
ESV07409 (0.004%) 0.8 0.50 – / –
ESV07443 (0.005%) 2.1 0.21 – / –
ESV07450 (0.009%) 8.3 0.02 * – / –
ESV07465 (0.01%) 0.9 0.45 – / –
ESV07472 (0.005%) 1.0 0.42 N / –
ESV07474 (0.02%) 1.3 0.35 – / –
ESV07506 (0.007%) 2.5 0.16 – / –
ESV07535 (0.005%) 0.6 0.59 – / –
ESV07566 (0.004%) 4.9 0.05 . – / –
ESV07575 (0.005%) 42.3 < 0.001 *** – / –
ESV07580 (0.005%) 0.3 0.73 – / –
c:Opitutae (0.38%) 0.4 0.70 – / –
o:Opitutales (0.38%) 0.4 0.70 – / –
f:Opitutaceae (0.38%) 0.4 0.70 – / –
g:Opitutus (0.38%) 0.4 0.70 – / –
g:Opitutus_uncl (0.38%) 0.4 0.70 – / –
ESV00854 (0.02%) 2.6 0.16 – / –
ESV01043 (0.03%) 0.8 0.49 – / –
ESV01707 (0.02%) 3.7 0.09 . – / –
ESV01903 (0.03%) 0.9 0.46 – / –
ESV01997 (0.01%) 1.5 0.31 – / –
ESV02318 (0.02%) 1.1 0.41 – / –
ESV03506 (0.02%) 3.4 0.10 – / –
ESV03705 (0.03%) 0.2 0.85 – / –
ESV03971 (0.03%) 1.2 0.37 – / –
ESV03994 (0.01%) 0.9 0.45 – / –
ESV04297 (0.007%) 1.1 0.39 – / –
ESV04654 (0.02%) 0.3 0.76 – / –

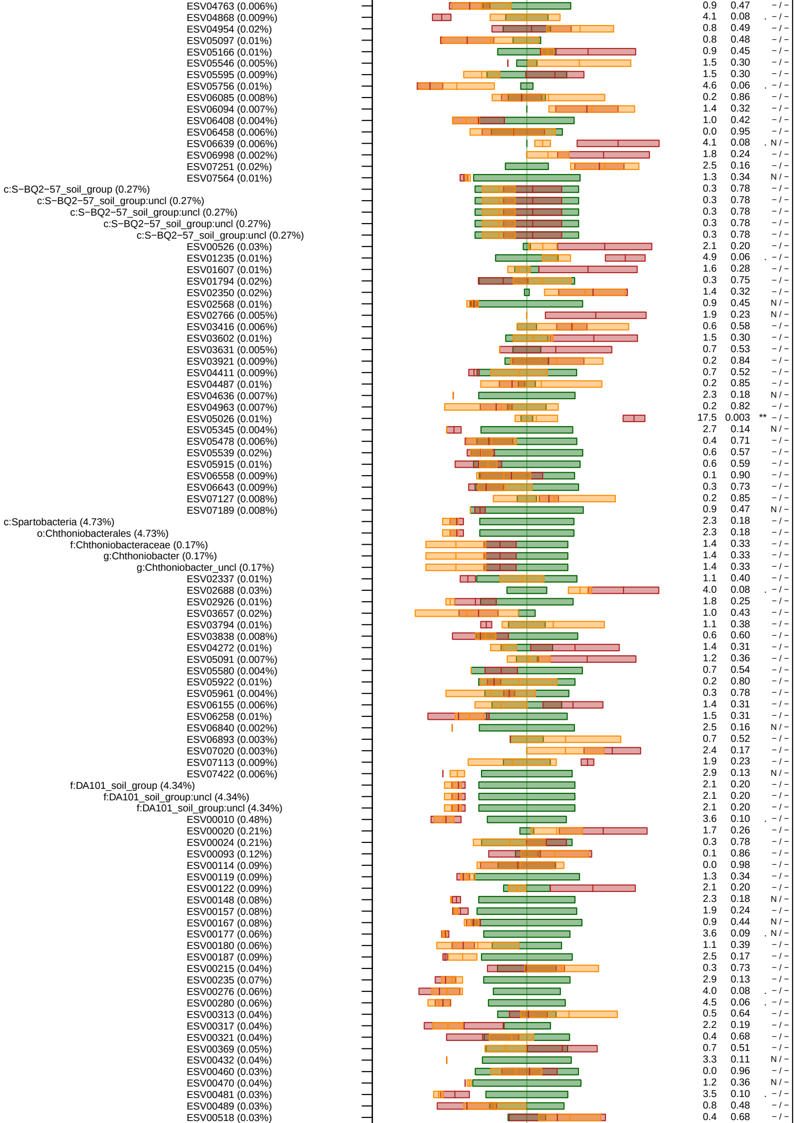
ESV04763 (0.006%) 0.9 0.47 - / -
ESV04868 (0.009%) 4.1 0.08 . - / -
ESV04954 (0.02%) 0.8 0.49 - / -
ESV05097 (0.01%) 0.8 0.48 - / -
ESV05166 (0.01%) 0.9 0.45 - / -
ESV05546 (0.005%) 1.5 0.30 - / -
ESV05595 (0.009%) 1.5 0.30 - / -
ESV05756 (0.01%) 4.6 0.06 . - / -
ESV06085 (0.008%) 0.2 0.86 - / -
ESV06094 (0.007%) 1.4 0.32 - / -
ESV06408 (0.004%) 1.0 0.42 - / -
ESV06458 (0.006%) 0.0 0.95 - / -
ESV06639 (0.006%) 4.1 0.08 . N / -
ESV06998 (0.002%) 1.8 0.24 - / -
ESV07251 (0.02%) 2.5 0.16 - / -
ESV07564 (0.01%) 1.3 0.34 N / -
c:S−BQ2−57_soil_group (0.27%) 0.3 0.78 - / -
c:S−BQ2−57_soil_group:uncl (0.27%) 0.3 0.78 - / -
c:S−BQ2−57_soil_group:uncl (0.27%) 0.3 0.78 - / -
c:S−BQ2−57_soil_group:uncl (0.27%) 0.3 0.78 - / -
c:S−BQ2−57_soil_group:uncl (0.27%) 0.3 0.78 - / -
ESV00526 (0.03%) 2.1 0.20 - / -
ESV01235 (0.01%) 4.9 0.06 . - / -
ESV01607 (0.01%) 1.6 0.28 - / -
ESV01794 (0.02%) 0.3 0.75 - / -
ESV02350 (0.02%) 1.4 0.32 - / -
ESV02568 (0.01%) 0.9 0.45 N / -
ESV02766 (0.005%) 1.9 0.23 N / -
ESV03416 (0.006%) 0.6 0.58 - / -
ESV03602 (0.01%) 1.5 0.30 - / -
ESV03631 (0.005%) 0.7 0.53 - / -
ESV03921 (0.009%) 0.2 0.84 - / -
ESV04411 (0.009%) 0.7 0.52 - / -
ESV04487 (0.01%) 0.2 0.85 - / -
ESV04636 (0.007%) 2.3 0.18 N / -
ESV04963 (0.007%) 0.2 0.82 - / -
ESV05026 (0.01%) 17.5 0.003 ** - / -
ESV05345 (0.004%) 2.7 0.14 N / -
ESV05478 (0.006%) 0.4 0.71 - / -
ESV05539 (0.02%) 0.6 0.57 - / -
ESV05915 (0.01%) 0.6 0.59 - / -
ESV06558 (0.009%) 0.1 0.90 - / -
ESV06643 (0.009%) 0.3 0.73 - / -
ESV07127 (0.008%) 0.2 0.85 - / -
ESV07189 (0.008%) 0.9 0.47 N / -
c:Spartobacteria (4.73%) 2.3 0.18 - / -
o:Chthoniobacterales (4.73%) 2.3 0.18 - / -
f:Chthoniobacteraceae (0.17%) 1.4 0.33 - / -
g:Chthoniobacter (0.17%) 1.4 0.33 - / -
g:Chthoniobacter_uncl (0.17%) 1.4 0.33 - / -
ESV02337 (0.01%) 1.1 0.40 - / -
ESV02688 (0.03%) 4.0 0.08 . - / -
ESV02926 (0.01%) 1.8 0.25 - / -
ESV03657 (0.02%) 1.0 0.43 - / -
ESV03794 (0.01%) 1.1 0.38 - / -
ESV03838 (0.008%) 0.6 0.60 - / -
ESV04272 (0.01%) 1.4 0.31 - / -
ESV05091 (0.007%) 1.2 0.36 - / -
ESV05580 (0.004%) 0.7 0.54 - / -
ESV05922 (0.01%) 0.2 0.80 - / -
ESV05961 (0.004%) 0.3 0.78 - / -
ESV06155 (0.006%) 1.4 0.31 - / -
ESV06258 (0.01%) 1.5 0.31 - / -
ESV06840 (0.002%) 2.5 0.16 N / -
ESV06893 (0.003%) 0.7 0.52 - / -
ESV07020 (0.003%) 2.4 0.17 - / -
ESV07113 (0.009%) 1.9 0.23 - / -
ESV07422 (0.006%) 2.9 0.13 N / -
f:DA101_soil_group (4.34%) 2.1 0.20 - / -
f:DA101_soil_group:uncl (4.34%) 2.1 0.20 - / -
f:DA101_soil_group:uncl (4.34%) 2.1 0.20 - / -
ESV00010 (0.48%) 3.6 0.10 . - / -
ESV00020 (0.21%) 1.7 0.26 - / -
ESV00024 (0.21%) 0.3 0.78 - / -
ESV00093 (0.12%) 0.1 0.86 - / -
ESV00114 (0.09%) 0.0 0.98 - / -
ESV00119 (0.09%) 1.3 0.34 - / -
ESV00122 (0.09%) 2.1 0.20 - / -
ESV00148 (0.08%) 2.3 0.18 N / -
ESV00157 (0.08%) 1.9 0.24 - / -
ESV00167 (0.08%) 0.9 0.44 N / -
ESV00177 (0.06%) 3.6 0.09 . N / -
ESV00180 (0.06%) 1.1 0.39 - / -
ESV00187 (0.09%) 2.5 0.17 - / -
ESV00215 (0.04%) 0.3 0.73 - / -
ESV00235 (0.07%) 2.9 0.13 - / -
ESV00276 (0.06%) 4.0 0.08 . - / -
ESV00280 (0.06%) 4.5 0.06 . - / -
ESV00313 (0.04%) 0.5 0.64 - / -
ESV00317 (0.04%) 2.2 0.19 - / -
ESV00321 (0.04%) 0.4 0.68 - / -
ESV00369 (0.05%) 0.7 0.51 - / -
ESV00432 (0.04%) 3.3 0.11 N / -
ESV00460 (0.03%) 0.0 0.96 - / -
ESV00470 (0.04%) 1.2 0.36 N / -
ESV00481 (0.03%) 3.5 0.10 . - / -
ESV00489 (0.03%) 0.8 0.48 - / -
ESV00518 (0.03%) 0.4 0.68 - / -

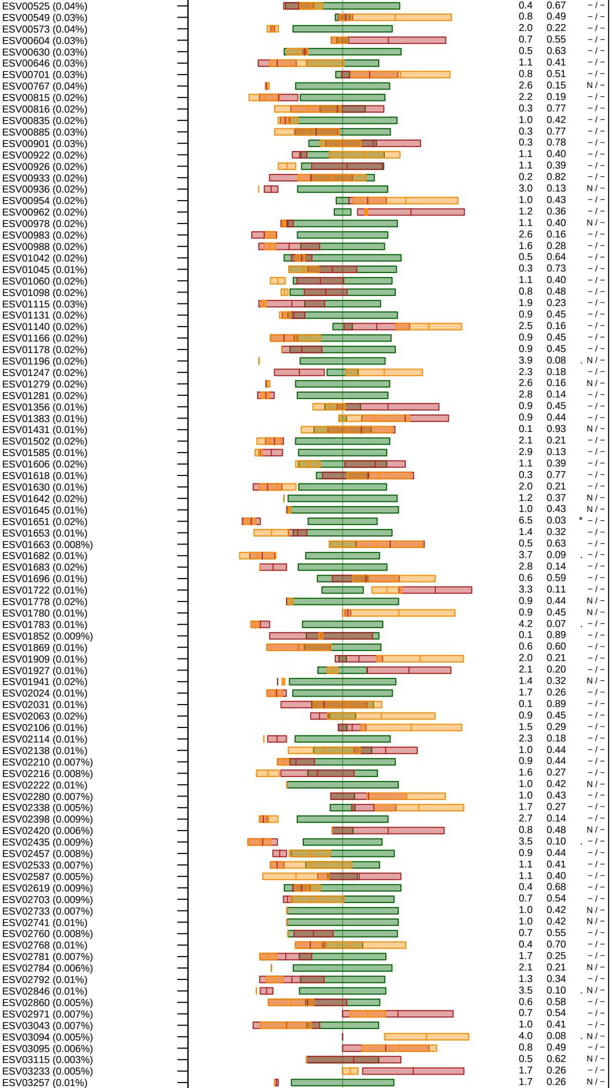
ESV00525 (0.04%) 0.4 0.67 − / −
ESV00549 (0.03%) 0.8 0.49 − / −
ESV00573 (0.04%) 2.0 0.22 − / −
ESV00604 (0.03%) 0.7 0.55 − / −
ESV00630 (0.03%) 0.5 0.63 − / −
ESV00646 (0.03%) 1.1 0.41 − / −
ESV00701 (0.03%) 0.8 0.51 − / −
ESV00767 (0.04%) 2.6 0.15 N / −
ESV00815 (0.02%) 2.2 0.19 − / −
ESV00816 (0.02%) 0.3 0.77 − / −
ESV00835 (0.02%) 1.0 0.42 − / −
ESV00885 (0.03%) 0.3 0.77 − / −
ESV00901 (0.03%) 0.3 0.78 − / −
ESV00922 (0.02%) 1.1 0.40 − / −
ESV00926 (0.02%) 1.1 0.39 − / −
ESV00933 (0.02%) 0.2 0.82 − / −
ESV00936 (0.02%) 3.0 0.13 N / −
ESV00954 (0.02%) 1.0 0.43 − / −
ESV00962 (0.02%) 1.2 0.36 − / −
ESV00978 (0.02%) 1.1 0.40 N / −
ESV00983 (0.02%) 2.6 0.16 − / −
ESV00988 (0.02%) 1.6 0.28 − / −
ESV01042 (0.02%) 0.5 0.64 − / −
ESV01045 (0.01%) 0.3 0.73 − / −
ESV01060 (0.02%) 1.1 0.40 − / −
ESV01098 (0.02%) 0.8 0.48 − / −
ESV01115 (0.03%) 1.9 0.23 − / −
ESV01131 (0.02%) 0.9 0.45 − / −
ESV01140 (0.02%) 2.5 0.16 − / −
ESV01166 (0.02%) 0.9 0.45 − / −
ESV01178 (0.02%) 0.9 0.45 − / −
ESV01196 (0.02%) 3.9 0.08 . N / −
ESV01247 (0.02%) 2.3 0.18 − / −
ESV01279 (0.02%) 2.6 0.16 N / −
ESV01281 (0.02%) 2.8 0.14 − / −
ESV01356 (0.01%) 0.9 0.45 − / −
ESV01383 (0.01%) 0.9 0.44 − / −
ESV01431 (0.01%) 0.1 0.93 N / −
ESV01502 (0.02%) 2.1 0.21 − / −
ESV01585 (0.01%) 2.9 0.13 − / −
ESV01606 (0.02%) 1.1 0.39 − / −
ESV01618 (0.01%) 0.3 0.77 − / −
ESV01630 (0.01%) 2.0 0.21 − / −
ESV01642 (0.02%) 1.2 0.37 N / −
ESV01645 (0.01%) 1.0 0.43 N / −
ESV01651 (0.02%) 6.5 0.03 * − / −
ESV01653 (0.01%) 1.4 0.32 − / −
ESV01663 (0.008%) 0.5 0.63 − / −
ESV01682 (0.01%) 3.7 0.09 . − / −
ESV01683 (0.02%) 2.8 0.14 − / −
ESV01696 (0.01%) 0.6 0.59 − / −
ESV01722 (0.01%) 3.3 0.11 − / −
ESV01778 (0.02%) 0.9 0.44 N / −
ESV01780 (0.01%) 0.9 0.45 N / −
ESV01783 (0.01%) 4.2 0.07 . − / −
ESV01852 (0.009%) 0.1 0.89 − / −
ESV01869 (0.01%) 0.6 0.60 − / −
ESV01909 (0.01%) 2.0 0.21 − / −
ESV01927 (0.01%) 2.1 0.20 − / −
ESV01941 (0.02%) 1.4 0.32 N / −
ESV02024 (0.01%) 1.7 0.26 − / −
ESV02031 (0.01%) 0.1 0.89 − / −
ESV02063 (0.02%) 0.9 0.45 − / −
ESV02106 (0.01%) 1.5 0.29 − / −
ESV02114 (0.01%) 2.3 0.18 − / −
ESV02138 (0.01%) 1.0 0.44 − / −
ESV02210 (0.007%) 0.9 0.44 − / −
ESV02216 (0.008%) 1.6 0.27 − / −
ESV02222 (0.01%) 1.0 0.42 N / −
ESV02280 (0.007%) 1.0 0.43 − / −
ESV02338 (0.005%) 1.7 0.27 − / −
ESV02398 (0.009%) 2.7 0.14 − / −
ESV02420 (0.006%) 0.8 0.48 N / −
ESV02435 (0.009%) 3.5 0.10 . − / −
ESV02457 (0.008%) 0.9 0.44 − / −
ESV02533 (0.007%) 1.1 0.41 − / −
ESV02587 (0.005%) 1.1 0.40 − / −
ESV02619 (0.009%) 0.4 0.68 − / −
ESV02703 (0.009%) 0.7 0.54 − / −
ESV02733 (0.007%) 1.0 0.42 N / −
ESV02741 (0.01%) 1.0 0.42 N / −
ESV02760 (0.008%) 0.7 0.55 − / −
ESV02768 (0.01%) 0.4 0.70 − / −
ESV02781 (0.007%) 1.7 0.25 − / −
ESV02784 (0.006%) 2.1 0.21 N / −
ESV02792 (0.01%) 1.3 0.34 − / −
ESV02846 (0.01%) 3.5 0.10 . N / −
ESV02860 (0.005%) 0.6 0.58 − / −
ESV02971 (0.007%) 0.7 0.54 − / −
ESV03043 (0.007%) 1.0 0.41 − / −
ESV03094 (0.005%) 4.0 0.08 . N / −
ESV03095 (0.006%) 0.8 0.49 − / −
ESV03115 (0.003%) 0.5 0.62 N / −
ESV03233 (0.005%) 1.7 0.26 − / −
ESV03257 (0.01%) 1.7 0.26 N / −

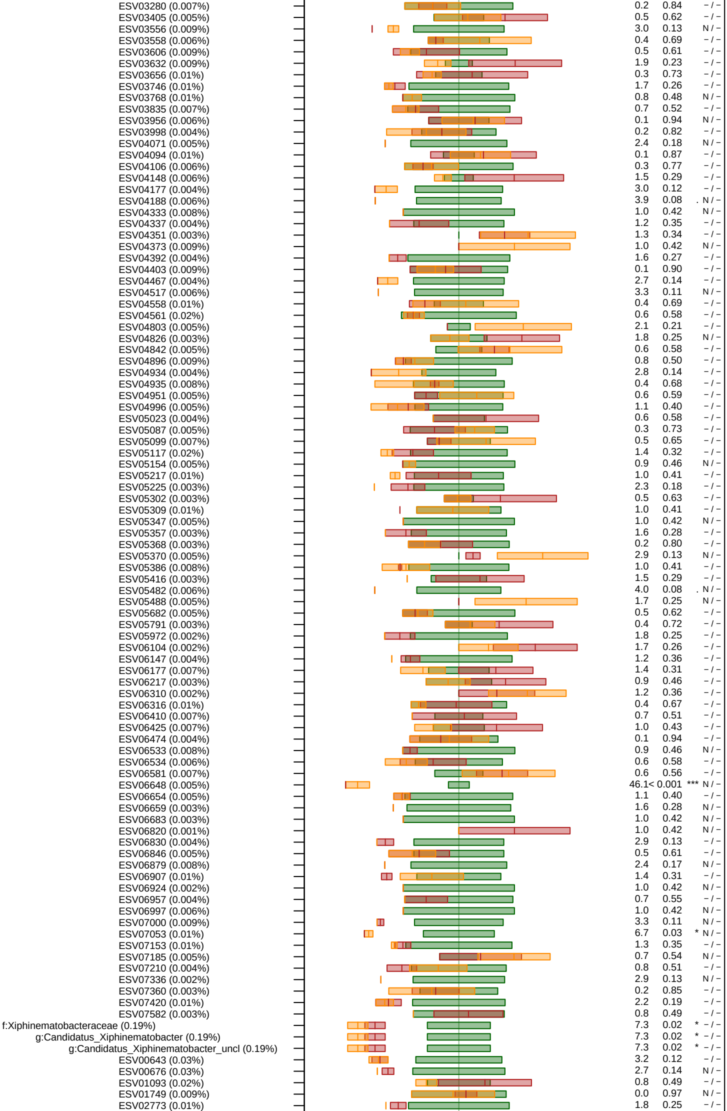
ESV03280 (0.007%) 0.2 0.84 − / −
ESV03405 (0.005%) 0.5 0.62 − / −
ESV03556 (0.009%) 3.0 0.13 N / −
ESV03558 (0.006%) 0.4 0.69 − / −
ESV03606 (0.009%) 0.5 0.61 − / −
ESV03632 (0.009%) 1.9 0.23 − / −
ESV03656 (0.01%) 0.3 0.73 − / −
ESV03746 (0.01%) 1.7 0.26 − / −
ESV03768 (0.01%) 0.8 0.48 N / −
ESV03835 (0.007%) 0.7 0.52 − / −
ESV03956 (0.006%) 0.1 0.94 N / −
ESV03998 (0.004%) 0.2 0.82 − / −
ESV04071 (0.005%) 2.4 0.18 N / −
ESV04094 (0.01%) 0.1 0.87 − / −
ESV04106 (0.006%) 0.3 0.77 − / −
ESV04148 (0.006%) 1.5 0.29 − / −
ESV04177 (0.004%) 3.0 0.12 − / −
ESV04188 (0.006%) 3.9 0.08 . N / −
ESV04333 (0.008%) 1.0 0.42 N / −
ESV04337 (0.004%) 1.2 0.35 − / −
ESV04351 (0.003%) 1.3 0.34 − / −
ESV04373 (0.009%) 1.0 0.42 N / −
ESV04392 (0.004%) 1.6 0.27 − / −
ESV04403 (0.009%) 0.1 0.90 − / −
ESV04467 (0.004%) 2.7 0.14 − / −
ESV04517 (0.006%) 3.3 0.11 N / −
ESV04558 (0.01%) 0.4 0.69 − / −
ESV04561 (0.02%) 0.6 0.58 − / −
ESV04803 (0.005%) 2.1 0.21 − / −
ESV04826 (0.003%) 1.8 0.25 N / −
ESV04842 (0.005%) 0.6 0.58 − / −
ESV04896 (0.009%) 0.8 0.50 − / −
ESV04934 (0.004%) 2.8 0.14 − / −
ESV04935 (0.008%) 0.4 0.68 − / −
ESV04951 (0.005%) 0.6 0.59 − / −
ESV04996 (0.005%) 1.1 0.40 − / −
ESV05023 (0.004%) 0.6 0.58 − / −
ESV05087 (0.005%) 0.3 0.73 − / −
ESV05099 (0.007%) 0.5 0.65 − / −
ESV05117 (0.02%) 1.4 0.32 − / −
ESV05154 (0.005%) 0.9 0.46 N / −
ESV05217 (0.01%) 1.0 0.41 − / −
ESV05225 (0.003%) 2.3 0.18 − / −
ESV05302 (0.003%) 0.5 0.63 − / −
ESV05309 (0.01%) 1.0 0.41 − / −
ESV05347 (0.005%) 1.0 0.42 N / −
ESV05357 (0.003%) 1.6 0.28 − / −
ESV05368 (0.003%) 0.2 0.80 − / −
ESV05370 (0.005%) 2.9 0.13 N / −
ESV05386 (0.008%) 1.0 0.41 − / −
ESV05416 (0.003%) 1.5 0.29 − / −
ESV05482 (0.006%) 4.0 0.08 . N / −
ESV05488 (0.005%) 1.7 0.25 N / −
ESV05682 (0.005%) 0.5 0.62 − / −
ESV05791 (0.003%) 0.4 0.72 − / −
ESV05972 (0.002%) 1.8 0.25 − / −
ESV06104 (0.002%) 1.7 0.26 − / −
ESV06147 (0.004%) 1.2 0.36 − / −
ESV06177 (0.007%) 1.4 0.31 − / −
ESV06217 (0.003%) 0.9 0.46 − / −
ESV06310 (0.002%) 1.2 0.36 − / −
ESV06316 (0.01%) 0.4 0.67 − / −
ESV06410 (0.007%) 0.7 0.51 − / −
ESV06425 (0.007%) 1.0 0.43 − / −
ESV06474 (0.004%) 0.1 0.94 − / −
ESV06533 (0.008%) 0.9 0.46 N / −
ESV06534 (0.006%) 0.6 0.58 − / −
ESV06581 (0.007%) 0.6 0.56 − / −
ESV06648 (0.005%) 46.1< 0.001 *** N / −
ESV06654 (0.005%) 1.1 0.40 − / −
ESV06659 (0.003%) 1.6 0.28 N / −
ESV06683 (0.003%) 1.0 0.42 N / −
ESV06820 (0.001%) 1.0 0.42 N / −
ESV06830 (0.004%) 2.9 0.13 − / −
ESV06846 (0.005%) 0.5 0.61 − / −
ESV06879 (0.008%) 2.4 0.17 N / −
ESV06907 (0.01%) 1.4 0.31 − / −
ESV06924 (0.002%) 1.0 0.42 N / −
ESV06957 (0.004%) 0.7 0.55 − / −
ESV06997 (0.006%) 1.0 0.42 N / −
ESV07000 (0.009%) 3.3 0.11 N / −
ESV07053 (0.01%) 6.7 0.03 * N / −
ESV07153 (0.01%) 1.3 0.35 − / −
ESV07185 (0.005%) 0.7 0.54 N / −
ESV07210 (0.004%) 0.8 0.51 − / −
ESV07336 (0.002%) 2.9 0.13 N / −
ESV07360 (0.003%) 0.2 0.85 − / −
ESV07420 (0.01%) 2.2 0.19 − / −
ESV07582 (0.003%) 0.8 0.49 − / −
f:Xiphinematobacteraceae (0.19%) 7.3 0.02 * − / −
g:Candidatus_Xiphinematobacter (0.19%) 7.3 0.02 * − / −
g:Candidatus_Xiphinematobacter_uncl (0.19%) 7.3 0.02 * − / −
ESV00643 (0.03%) 3.2 0.12 − / −
ESV00676 (0.03%) 2.7 0.14 N / −
ESV01093 (0.02%) 0.8 0.49 − / −
ESV01749 (0.009%) 0.0 0.97 N / −
ESV02773 (0.01%) 1.8 0.25 − / −

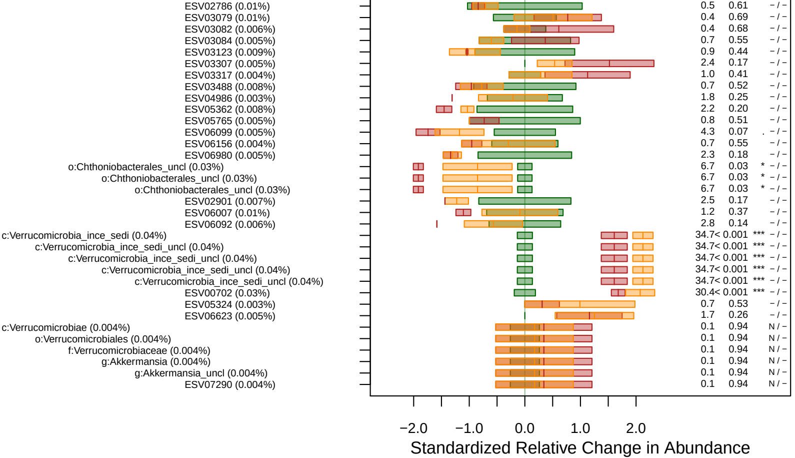
ESV02786 (0.01%) 0.5 0.61 – / –
ESV03079 (0.01%) 0.4 0.69 – / –
ESV03082 (0.006%) 0.4 0.68 – / –
ESV03084 (0.005%) 0.7 0.55 – / –
ESV03123 (0.009%) 0.9 0.44 – / –
ESV03307 (0.005%) 2.4 0.17 – / –
ESV03317 (0.004%) 1.0 0.41 – / –
ESV03488 (0.008%) 0.7 0.52 – / –
ESV04986 (0.003%) 1.8 0.25 – / –
ESV05362 (0.008%) 2.2 0.20 – / –
ESV05765 (0.005%) 0.8 0.51 – / –
ESV06099 (0.005%) 4.3 0.07 . – / –
ESV06156 (0.004%) 0.7 0.55 – / –
ESV06980 (0.005%) 2.3 0.18 – / –
o:Chthoniobacterales_uncl (0.03%) 6.7 0.03 * – / –
o:Chthoniobacterales_uncl (0.03%) 6.7 0.03 * – / –
o:Chthoniobacterales_uncl (0.03%) 6.7 0.03 * – / –
ESV02901 (0.007%) 2.5 0.17 – / –
ESV06007 (0.01%) 1.2 0.37 – / –
ESV06092 (0.006%) 2.8 0.14 – / –
c:Verrucomicrobia_ince_sedi (0.04%) 34.7 < 0.001 *** – / –
c:Verrucomicrobia_ince_sedi_uncl (0.04%) 34.7 < 0.001 *** – / –
c:Verrucomicrobia_ince_sedi_uncl (0.04%) 34.7 < 0.001 *** – / –
c:Verrucomicrobia_ince_sedi_uncl (0.04%) 34.7 < 0.001 *** – / –
c:Verrucomicrobia_ince_sedi_uncl (0.04%) 34.7 < 0.001 *** – / –
ESV00702 (0.03%) 30.4 < 0.001 *** – / –
ESV05324 (0.003%) 0.7 0.53 – / –
ESV06623 (0.005%) 1.7 0.26 – / –
c:Verrucomicrobiae (0.004%) 0.1 0.94 N / –
o:Verrucomicrobiales (0.004%) 0.1 0.94 N / –
f:Verrucomicrobiaceae (0.004%) 0.1 0.94 N / –
g:Akkermansia (0.004%) 0.1 0.94 N / –
g:Akkermansia_uncl (0.004%) 0.1 0.94 N / –
ESV07290 (0.004%) 0.1 0.94 N / –
−2.0 −1.0 0.0 1.0 2.0
Standardized Relative Change in Abundance
