# Supplementary material for: Thinning Partially Mitigates the Impact of Atlantic Forest Replacement by Pine Monocultures on the Soil Microbiome
Source: Front Microbiol. 2020 Jul 3;11:1491. doi: 10.3389/fmicb.2020.01491 (PMC7350009; doi:10.3389/fmicb.2020.01491)
Supplement: Supplementary file 2 [file Image_2.PDF]

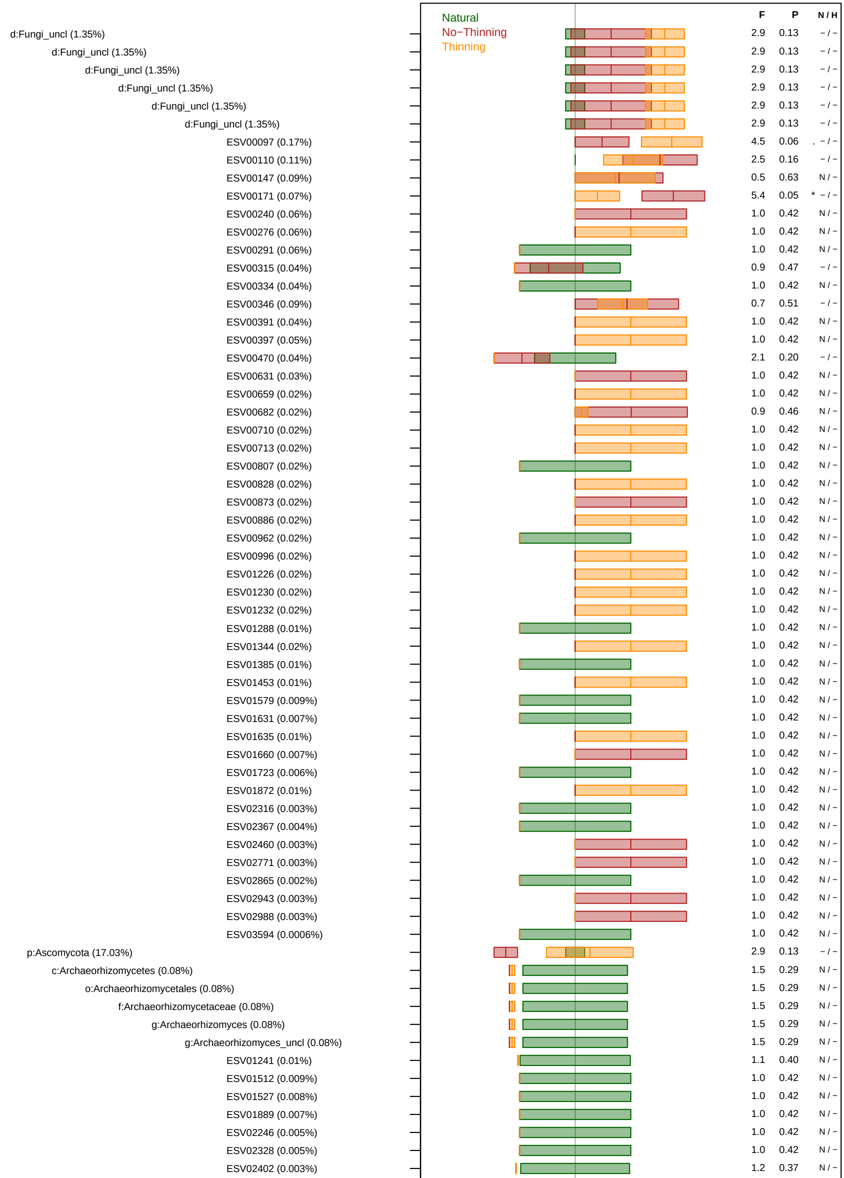

Natural
No−Thinning
Thinning
F
P
N / H
d:Fungi_uncl (1.35%) 2.9 0.13 – / –
d:Fungi_uncl (1.35%) 2.9 0.13 – / –
d:Fungi_uncl (1.35%) 2.9 0.13 – / –
d:Fungi_uncl (1.35%) 2.9 0.13 – / –
d:Fungi_uncl (1.35%) 2.9 0.13 – / –
d:Fungi_uncl (1.35%) 2.9 0.13 – / –
ESV00097 (0.17%) 4.5 0.06 . – / –
ESV00110 (0.11%) 2.5 0.16 – / –
ESV00147 (0.09%) 0.5 0.63 N / –
ESV00171 (0.07%) 5.4 0.05 * – / –
ESV00240 (0.06%) 1.0 0.42 N / –
ESV00276 (0.06%) 1.0 0.42 N / –
ESV00291 (0.06%) 1.0 0.42 N / –
ESV00315 (0.04%) 0.9 0.47 – / –
ESV00334 (0.04%) 1.0 0.42 N / –
ESV00346 (0.09%) 0.7 0.51 – / –
ESV00391 (0.04%) 1.0 0.42 N / –
ESV00397 (0.05%) 1.0 0.42 N / –
ESV00470 (0.04%) 2.1 0.20 – / –
ESV00631 (0.03%) 1.0 0.42 N / –
ESV00659 (0.02%) 1.0 0.42 N / –
ESV00682 (0.02%) 0.9 0.46 N / –
ESV00710 (0.02%) 1.0 0.42 N / –
ESV00713 (0.02%) 1.0 0.42 N / –
ESV00807 (0.02%) 1.0 0.42 N / –
ESV00828 (0.02%) 1.0 0.42 N / –
ESV00873 (0.02%) 1.0 0.42 N / –
ESV00886 (0.02%) 1.0 0.42 N / –
ESV00962 (0.02%) 1.0 0.42 N / –
ESV00996 (0.02%) 1.0 0.42 N / –
ESV01226 (0.02%) 1.0 0.42 N / –
ESV01230 (0.02%) 1.0 0.42 N / –
ESV01232 (0.02%) 1.0 0.42 N / –
ESV01288 (0.01%) 1.0 0.42 N / –
ESV01344 (0.02%) 1.0 0.42 N / –
ESV01385 (0.01%) 1.0 0.42 N / –
ESV01453 (0.01%) 1.0 0.42 N / –
ESV01579 (0.009%) 1.0 0.42 N / –
ESV01631 (0.007%) 1.0 0.42 N / –
ESV01635 (0.01%) 1.0 0.42 N / –
ESV01660 (0.007%) 1.0 0.42 N / –
ESV01723 (0.006%) 1.0 0.42 N / –
ESV01872 (0.01%) 1.0 0.42 N / –
ESV02316 (0.003%) 1.0 0.42 N / –
ESV02367 (0.004%) 1.0 0.42 N / –
ESV02460 (0.003%) 1.0 0.42 N / –
ESV02771 (0.003%) 1.0 0.42 N / –
ESV02865 (0.002%) 1.0 0.42 N / –
ESV02943 (0.003%) 1.0 0.42 N / –
ESV02988 (0.003%) 1.0 0.42 N / –
ESV03594 (0.0006%) 1.0 0.42 N / –
p:Ascomycota (17.03%) 2.9 0.13 – / –
c:Archaeorhizomycetes (0.08%) 1.5 0.29 N / –
o:Archaeorhizomycetales (0.08%) 1.5 0.29 N / –
f:Archaeorhizomycetaceae (0.08%) 1.5 0.29 N / –
g:Archaeorhizomyces (0.08%) 1.5 0.29 N / –
g:Archaeorhizomyces_uncl (0.08%) 1.5 0.29 N / –
ESV01241 (0.01%) 1.1 0.40 N / –
ESV01512 (0.009%) 1.0 0.42 N / –
ESV01527 (0.008%) 1.0 0.42 N / –
ESV01889 (0.007%) 1.0 0.42 N / –
ESV02246 (0.005%) 1.0 0.42 N / –
ESV02328 (0.005%) 1.0 0.42 N / –
ESV02402 (0.003%) 1.2 0.37 N / –

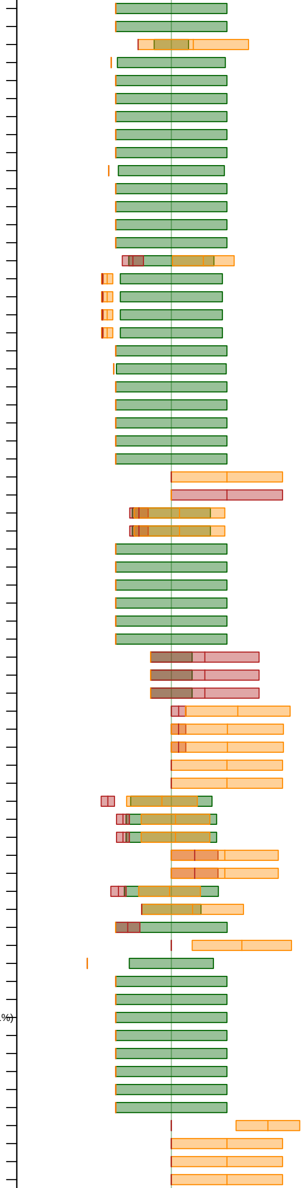

ESV02429 (0.004%)	1.0	0.42	N / –
ESV02508 (0.004%)	1.0	0.42	N / –
ESV02527 (0.005%)	0.7	0.54	– / –
ESV02560 (0.004%)	1.2	0.35	N / –
ESV02628 (0.004%)	1.0	0.42	N / –
ESV03058 (0.002%)	1.0	0.42	N / –
ESV03203 (0.001%)	1.0	0.42	N / –
ESV03354 (0.001%)	1.0	0.42	N / –
ESV03428 (0.001%)	1.0	0.42	N / –
ESV03451 (0.001%)	1.4	0.32	N / –
ESV03480 (0.001%)	1.0	0.42	N / –
ESV03504 (0.001%)	1.0	0.42	N / –
ESV03548 (0.001%)	1.0	0.42	N / –
ESV03583 (0.001%)	1.0	0.42	N / –
c:Dothideomycetes (3.09%)	1.3	0.34	– / –
c:Dothideomycetes_uncl (0.07%)	1.7	0.26	N / –
c:Dothideomycetes_uncl (0.07%)	1.7	0.26	N / –
c:Dothideomycetes_uncl (0.07%)	1.7	0.26	N / –
c:Dothideomycetes_uncl (0.07%)	1.7	0.26	N / –
ESV01084 (0.02%)	1.0	0.42	N / –
ESV01589 (0.01%)	1.1	0.39	N / –
ESV01607 (0.007%)	1.0	0.42	N / –
ESV01804 (0.01%)	1.0	0.42	N / –
ESV02050 (0.005%)	1.0	0.42	N / –
ESV02348 (0.006%)	1.0	0.42	N / –
ESV02750 (0.004%)	1.0	0.42	N / –
ESV02837 (0.005%)	1.0	0.42	N / –
ESV03612 (0.0007%)	1.0	0.42	N / –
o:Botryosphaeriales (0.15%)	0.4	0.70	– / –
f:Botryosphaeriaceae (0.15%)	0.4	0.70	– / –
g:Barriopsis (0.004%)	1.0	0.42	N / –
s:Barriopsis_fusca (0.004%)	1.0	0.42	N / –
ESV02413 (0.004%)	1.0	0.42	N / –
g:Botryosphaeria (0.05%)	1.0	0.42	N / –
g:Botryosphaeria_uncl (0.05%)	1.0	0.42	N / –
ESV00325 (0.05%)	1.0	0.42	N / –
g:Dothiorella (0.02%)	0.7	0.55	– / –
g:Dothiorella_uncl (0.02%)	0.7	0.55	– / –
ESV00815 (0.02%)	0.7	0.55	– / –
g:Lasiodiplodia (0.08%)	1.4	0.31	– / –
g:Lasiodiplodia_uncl (0.07%)	0.9	0.46	N / –
ESV00190 (0.07%)	0.9	0.46	N / –
s:Lasiodiplodia_gonubiensis (0.01%)	1.0	0.42	N / –
ESV01569 (0.01%)	1.0	0.42	N / –
o:Capnodiales (0.14%)	1.2	0.37	– / –
f:Cladosporiaceae (0.13%)	0.8	0.50	– / –
g:Cladosporium (0.13%)	0.8	0.50	– / –
g:Cladosporium_uncl (0.008%)	0.6	0.56	– / –
ESV01479 (0.008%)	0.6	0.56	– / –
s:Cladosporium_exasperatum (0.12%)	0.8	0.48	– / –
ESV00335 (0.04%)	0.6	0.60	– / –
ESV00464 (0.04%)	0.8	0.50	– / –
ESV00818 (0.03%)	2.0	0.21	N / –
ESV01404 (0.009%)	4.0	0.08	. N / –
f:Mycosphaerellaceae (0.01%)	1.0	0.42	N / –
g:Pseudocercospora (0.01%)	1.0	0.42	N / –
s:Pseudocercospora_sphaerellae−eugeniae (0.01%)	1.0	0.42	N / –
ESV01228 (0.01%)	1.0	0.42	N / –
o:Capnodiales_uncl (0.002%)	1.0	0.42	N / –
o:Capnodiales_uncl (0.002%)	1.0	0.42	N / –
o:Capnodiales_uncl (0.002%)	1.0	0.42	N / –
ESV02958 (0.002%)	1.0	0.42	N / –
o:Dothideales (0.07%)	9.2	0.01	* N / –
f:Aureobasidiaceae (0.04%)	1.0	0.42	N / –
g:Aureobasidium (0.04%)	1.0	0.42	N / –
g:Aureobasidium_uncl (0.04%)	1.0	0.42	N / –

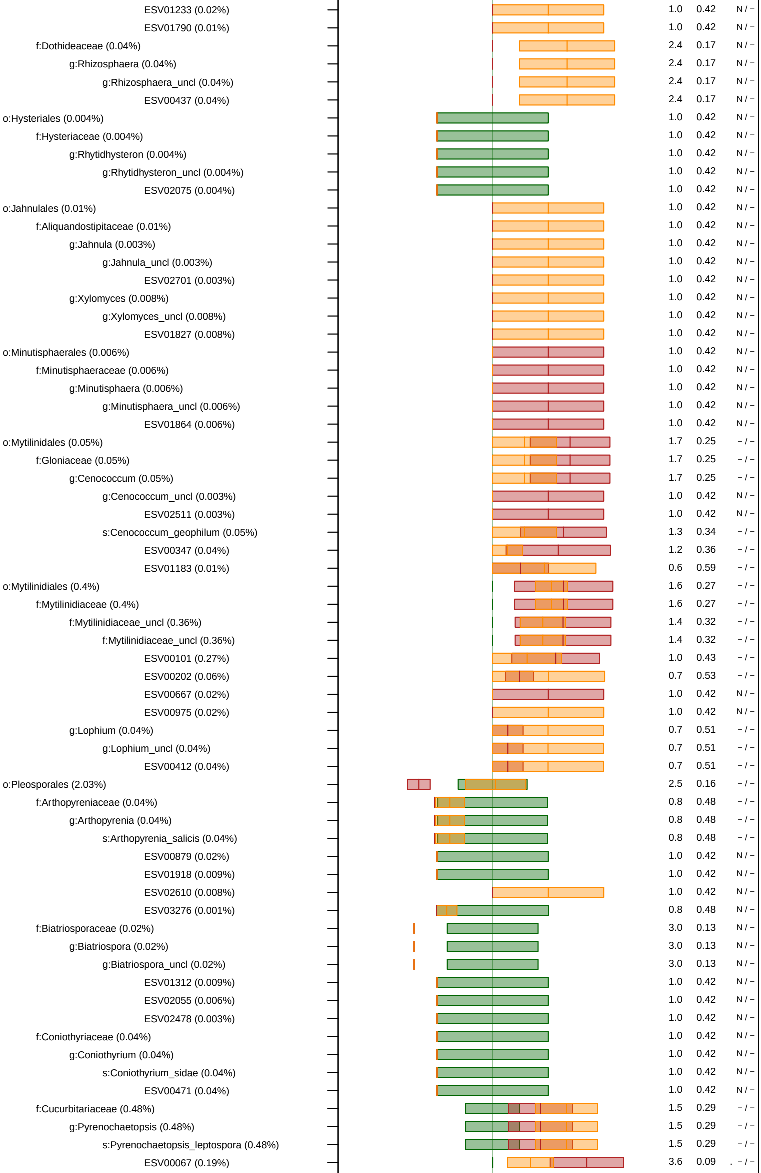
ESV01233 (0.02%) 1.0 0.42 N / –
ESV01790 (0.01%) 1.0 0.42 N / –
f:Dothideaceae (0.04%) 2.4 0.17 N / –
g:Rhizosphaera (0.04%) 2.4 0.17 N / –
g:Rhizosphaera_uncl (0.04%) 2.4 0.17 N / –
ESV00437 (0.04%) 2.4 0.17 N / –
o:Hysteriales (0.004%) 1.0 0.42 N / –
f:Hysteriaceae (0.004%) 1.0 0.42 N / –
g:Rhytidhysteron (0.004%) 1.0 0.42 N / –
g:Rhytidhysteron_uncl (0.004%) 1.0 0.42 N / –
ESV02075 (0.004%) 1.0 0.42 N / –
o:Jahnulales (0.01%) 1.0 0.42 N / –
f:Aliquandostipitaceae (0.01%) 1.0 0.42 N / –
g:Jahnula (0.003%) 1.0 0.42 N / –
g:Jahnula_uncl (0.003%) 1.0 0.42 N / –
ESV02701 (0.003%) 1.0 0.42 N / –
g:Xylomyces (0.008%) 1.0 0.42 N / –
g:Xylomyces_uncl (0.008%) 1.0 0.42 N / –
ESV01827 (0.008%) 1.0 0.42 N / –
o:Minutisphaerales (0.006%) 1.0 0.42 N / –
f:Minutisphaeraceae (0.006%) 1.0 0.42 N / –
g:Minutisphaera (0.006%) 1.0 0.42 N / –
g:Minutisphaera_uncl (0.006%) 1.0 0.42 N / –
ESV01864 (0.006%) 1.0 0.42 N / –
o:Mytilinidales (0.05%) 1.7 0.25 – / –
f:Gloniaceae (0.05%) 1.7 0.25 – / –
g:Cenococcum (0.05%) 1.7 0.25 – / –
g:Cenococcum_uncl (0.003%) 1.0 0.42 N / –
ESV02511 (0.003%) 1.0 0.42 N / –
s:Cenococcum_geophilum (0.05%) 1.3 0.34 – / –
ESV00347 (0.04%) 1.2 0.36 – / –
ESV01183 (0.01%) 0.6 0.59 – / –
o:Mytilinidiales (0.4%) 1.6 0.27 – / –
f:Mytilinidiaceae (0.4%) 1.6 0.27 – / –
f:Mytilinidiaceae_uncl (0.36%) 1.4 0.32 – / –
f:Mytilinidiaceae_uncl (0.36%) 1.4 0.32 – / –
ESV00101 (0.27%) 1.0 0.43 – / –
ESV00202 (0.06%) 0.7 0.53 – / –
ESV00667 (0.02%) 1.0 0.42 N / –
ESV00975 (0.02%) 1.0 0.42 N / –
g:Lophium (0.04%) 0.7 0.51 – / –
g:Lophium_uncl (0.04%) 0.7 0.51 – / –
ESV00412 (0.04%) 0.7 0.51 – / –
o:Pleosporales (2.03%) 2.5 0.16 – / –
f:Arthopyreniaceae (0.04%) 0.8 0.48 – / –
g:Arthopyrenia (0.04%) 0.8 0.48 – / –
s:Arthopyrenia_salicis (0.04%) 0.8 0.48 – / –
ESV00879 (0.02%) 1.0 0.42 N / –
ESV01918 (0.009%) 1.0 0.42 N / –
ESV02610 (0.008%) 1.0 0.42 N / –
ESV03276 (0.001%) 0.8 0.48 N / –
f:Biatriosporaceae (0.02%) 3.0 0.13 N / –
g:Biatriospora (0.02%) 3.0 0.13 N / –
g:Biatriospora_uncl (0.02%) 3.0 0.13 N / –
ESV01312 (0.009%) 1.0 0.42 N / –
ESV02055 (0.006%) 1.0 0.42 N / –
ESV02478 (0.003%) 1.0 0.42 N / –
f:Coniothyriaceae (0.04%) 1.0 0.42 N / –
g:Coniothyrium (0.04%) 1.0 0.42 N / –
s:Coniothyrium_sidae (0.04%) 1.0 0.42 N / –
ESV00471 (0.04%) 1.0 0.42 N / –
f:Cucurbitariaceae (0.48%) 1.5 0.29 – / –
g:Pyrenochaetopsis (0.48%) 1.5 0.29 – / –
s:Pyrenochaetopsis_leptospora (0.48%) 1.5 0.29 – / –
ESV00067 (0.19%) 3.6 0.09 – / –

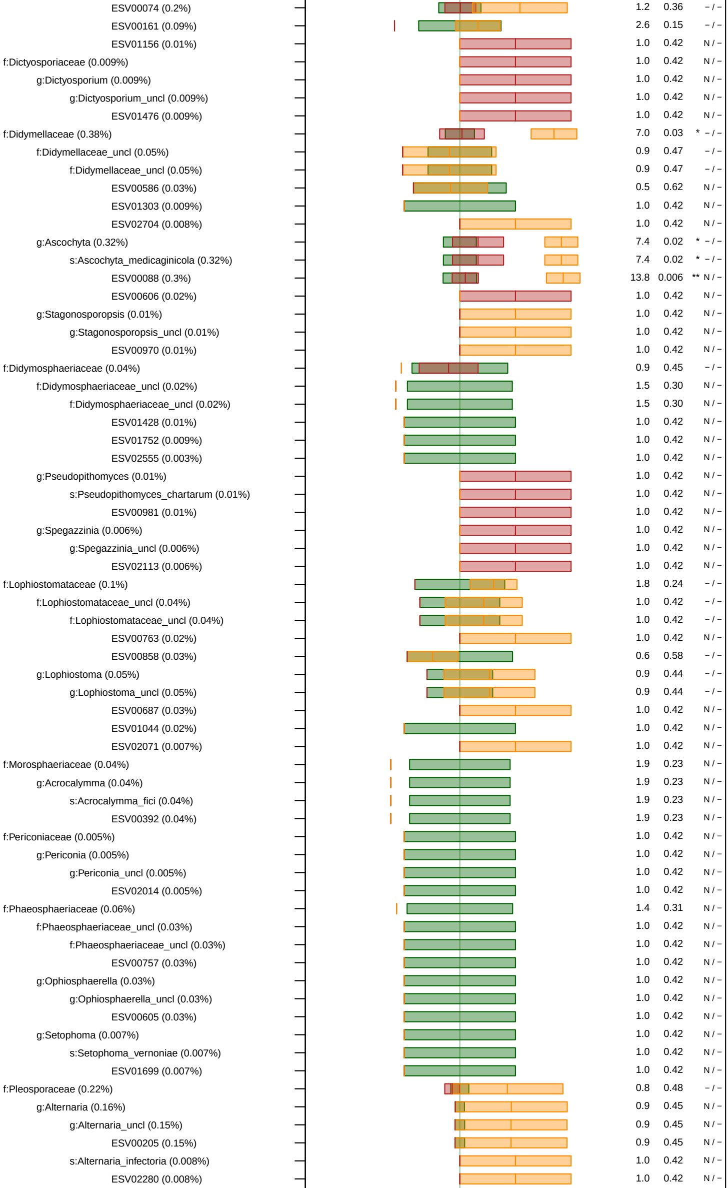
ESV00074 (0.2%) 1.2 0.36 – / –
ESV00161 (0.09%) 2.6 0.15 – / –
ESV01156 (0.01%) 1.0 0.42 N / –
f:Dictyosporiaceae (0.009%) 1.0 0.42 N / –
g:Dictyosporium (0.009%) 1.0 0.42 N / –
g:Dictyosporium_uncl (0.009%) 1.0 0.42 N / –
ESV01476 (0.009%) 1.0 0.42 N / –
f:Didymellaceae (0.38%) 7.0 0.03 * – / –
f:Didymellaceae_uncl (0.05%) 0.9 0.47 – / –
f:Didymellaceae_uncl (0.05%) 0.9 0.47 – / –
ESV00586 (0.03%) 0.5 0.62 N / –
ESV01303 (0.009%) 1.0 0.42 N / –
ESV02704 (0.008%) 1.0 0.42 N / –
g:Ascochyta (0.32%) 7.4 0.02 * – / –
s:Ascochyta_medicaginicola (0.32%) 7.4 0.02 * – / –
ESV00088 (0.3%) 13.8 0.006 ** N / –
ESV00606 (0.02%) 1.0 0.42 N / –
g:Stagonosporopsis (0.01%) 1.0 0.42 N / –
g:Stagonosporopsis_uncl (0.01%) 1.0 0.42 N / –
ESV00970 (0.01%) 1.0 0.42 N / –
f:Didymosphaeriaceae (0.04%) 0.9 0.45 – / –
f:Didymosphaeriaceae_uncl (0.02%) 1.5 0.30 N / –
f:Didymosphaeriaceae_uncl (0.02%) 1.5 0.30 N / –
ESV01428 (0.01%) 1.0 0.42 N / –
ESV01752 (0.009%) 1.0 0.42 N / –
ESV02555 (0.003%) 1.0 0.42 N / –
g:Pseudopithomyces (0.01%) 1.0 0.42 N / –
s:Pseudopithomyces_chartarum (0.01%) 1.0 0.42 N / –
ESV00981 (0.01%) 1.0 0.42 N / –
g:Spegazzinia (0.006%) 1.0 0.42 N / –
g:Spegazzinia_uncl (0.006%) 1.0 0.42 N / –
ESV02113 (0.006%) 1.0 0.42 N / –
f:Lophiostomataceae (0.1%) 1.8 0.24 – / –
f:Lophiostomataceae_uncl (0.04%) 1.0 0.42 – / –
f:Lophiostomataceae_uncl (0.04%) 1.0 0.42 – / –
ESV00763 (0.02%) 1.0 0.42 N / –
ESV00858 (0.03%) 0.6 0.58 – / –
g:Lophiostoma (0.05%) 0.9 0.44 – / –
g:Lophiostoma_uncl (0.05%) 0.9 0.44 – / –
ESV00687 (0.03%) 1.0 0.42 N / –
ESV01044 (0.02%) 1.0 0.42 N / –
ESV02071 (0.007%) 1.0 0.42 N / –
f:Morosphaeriaceae (0.04%) 1.9 0.23 N / –
g:Acrocalymma (0.04%) 1.9 0.23 N / –
s:Acrocalymma_fici (0.04%) 1.9 0.23 N / –
ESV00392 (0.04%) 1.9 0.23 N / –
f:Periconiaceae (0.005%) 1.0 0.42 N / –
g:Periconia (0.005%) 1.0 0.42 N / –
g:Periconia_uncl (0.005%) 1.0 0.42 N / –
ESV02014 (0.005%) 1.0 0.42 N / –
f:Phaeosphaeriaceae (0.06%) 1.4 0.31 N / –
f:Phaeosphaeriaceae_uncl (0.03%) 1.0 0.42 N / –
f:Phaeosphaeriaceae_uncl (0.03%) 1.0 0.42 N / –
ESV00757 (0.03%) 1.0 0.42 N / –
g:Ophiosphaerella (0.03%) 1.0 0.42 N / –
g:Ophiosphaerella_uncl (0.03%) 1.0 0.42 N / –
ESV00605 (0.03%) 1.0 0.42 N / –
g:Setophoma (0.007%) 1.0 0.42 N / –
s:Setophoma_vernoniae (0.007%) 1.0 0.42 N / –
ESV01699 (0.007%) 1.0 0.42 N / –
f:Pleosporaceae (0.22%) 0.8 0.48 – / –
g:Alternaria (0.16%) 0.9 0.45 N / –
g:Alternaria_uncl (0.15%) 0.9 0.45 N / –
ESV00205 (0.15%) 0.9 0.45 N / –
s:Alternaria_infectoria (0.008%) 1.0 0.42 N / –
ESV02280 (0.008%) 1.0 0.42 N / –

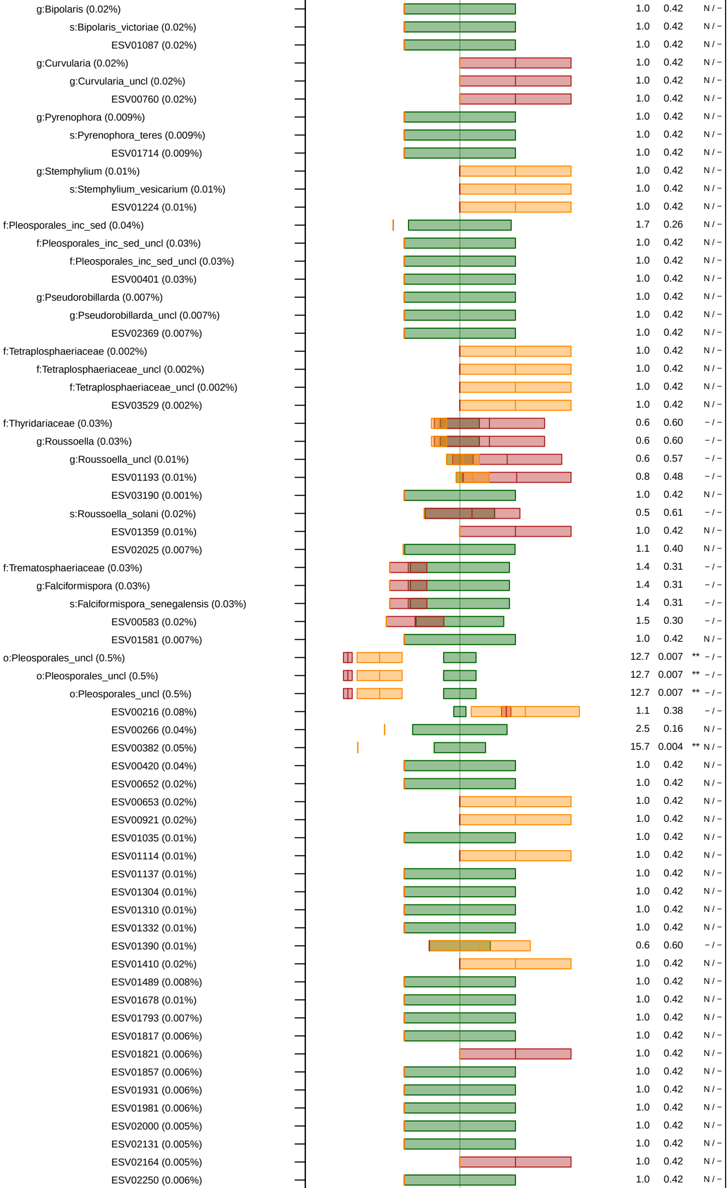
g:Bipolaris (0.02%) 1.0 0.42 N / –
s:Bipolaris_victoriae (0.02%) 1.0 0.42 N / –
ESV01087 (0.02%) 1.0 0.42 N / –
g:Curvularia (0.02%) 1.0 0.42 N / –
g:Curvularia_uncl (0.02%) 1.0 0.42 N / –
ESV00760 (0.02%) 1.0 0.42 N / –
g:Pyrenophora (0.009%) 1.0 0.42 N / –
s:Pyrenophora_teres (0.009%) 1.0 0.42 N / –
ESV01714 (0.009%) 1.0 0.42 N / –
g:Stemphylium (0.01%) 1.0 0.42 N / –
s:Stemphylium_vesicarium (0.01%) 1.0 0.42 N / –
ESV01224 (0.01%) 1.0 0.42 N / –
f:Pleosporales_inc_sed (0.04%) 1.7 0.26 N / –
f:Pleosporales_inc_sed_uncl (0.03%) 1.0 0.42 N / –
f:Pleosporales_inc_sed_uncl (0.03%) 1.0 0.42 N / –
ESV00401 (0.03%) 1.0 0.42 N / –
g:Pseudorobillarda (0.007%) 1.0 0.42 N / –
g:Pseudorobillarda_uncl (0.007%) 1.0 0.42 N / –
ESV02369 (0.007%) 1.0 0.42 N / –
f:Tetraplosphaeriaceae (0.002%) 1.0 0.42 N / –
f:Tetraplosphaeriaceae_uncl (0.002%) 1.0 0.42 N / –
f:Tetraplosphaeriaceae_uncl (0.002%) 1.0 0.42 N / –
ESV03529 (0.002%) 1.0 0.42 N / –
f:Thyridariaceae (0.03%) 0.6 0.60 – / –
g:Roussoella (0.03%) 0.6 0.60 – / –
g:Roussoella_uncl (0.01%) 0.6 0.57 – / –
ESV01193 (0.01%) 0.8 0.48 – / –
ESV03190 (0.001%) 1.0 0.42 N / –
s:Roussoella_solani (0.02%) 0.5 0.61 – / –
ESV01359 (0.01%) 1.0 0.42 N / –
ESV02025 (0.007%) 1.1 0.40 N / –
f:Trematosphaeriaceae (0.03%) 1.4 0.31 – / –
g:Falciformispora (0.03%) 1.4 0.31 – / –
s:Falciformispora_senegalensis (0.03%) 1.4 0.31 – / –
ESV00583 (0.02%) 1.5 0.30 – / –
ESV01581 (0.007%) 1.0 0.42 N / –
o:Pleosporales_uncl (0.5%) 12.7 0.007 ** – / –
o:Pleosporales_uncl (0.5%) 12.7 0.007 ** – / –
o:Pleosporales_uncl (0.5%) 12.7 0.007 ** – / –
ESV00216 (0.08%) 1.1 0.38 – / –
ESV00266 (0.04%) 2.5 0.16 N / –
ESV00382 (0.05%) 15.7 0.004 ** N / –
ESV00420 (0.04%) 1.0 0.42 N / –
ESV00652 (0.02%) 1.0 0.42 N / –
ESV00653 (0.02%) 1.0 0.42 N / –
ESV00921 (0.02%) 1.0 0.42 N / –
ESV01035 (0.01%) 1.0 0.42 N / –
ESV01114 (0.01%) 1.0 0.42 N / –
ESV01137 (0.01%) 1.0 0.42 N / –
ESV01304 (0.01%) 1.0 0.42 N / –
ESV01310 (0.01%) 1.0 0.42 N / –
ESV01332 (0.01%) 1.0 0.42 N / –
ESV01390 (0.01%) 0.6 0.60 – / –
ESV01410 (0.02%) 1.0 0.42 N / –
ESV01489 (0.008%) 1.0 0.42 N / –
ESV01678 (0.01%) 1.0 0.42 N / –
ESV01793 (0.007%) 1.0 0.42 N / –
ESV01817 (0.006%) 1.0 0.42 N / –
ESV01821 (0.006%) 1.0 0.42 N / –
ESV01857 (0.006%) 1.0 0.42 N / –
ESV01931 (0.006%) 1.0 0.42 N / –
ESV01981 (0.006%) 1.0 0.42 N / –
ESV02000 (0.005%) 1.0 0.42 N / –
ESV02131 (0.005%) 1.0 0.42 N / –
ESV02164 (0.005%) 1.0 0.42 N / –
ESV02250 (0.006%) 1.0 0.42 N / –

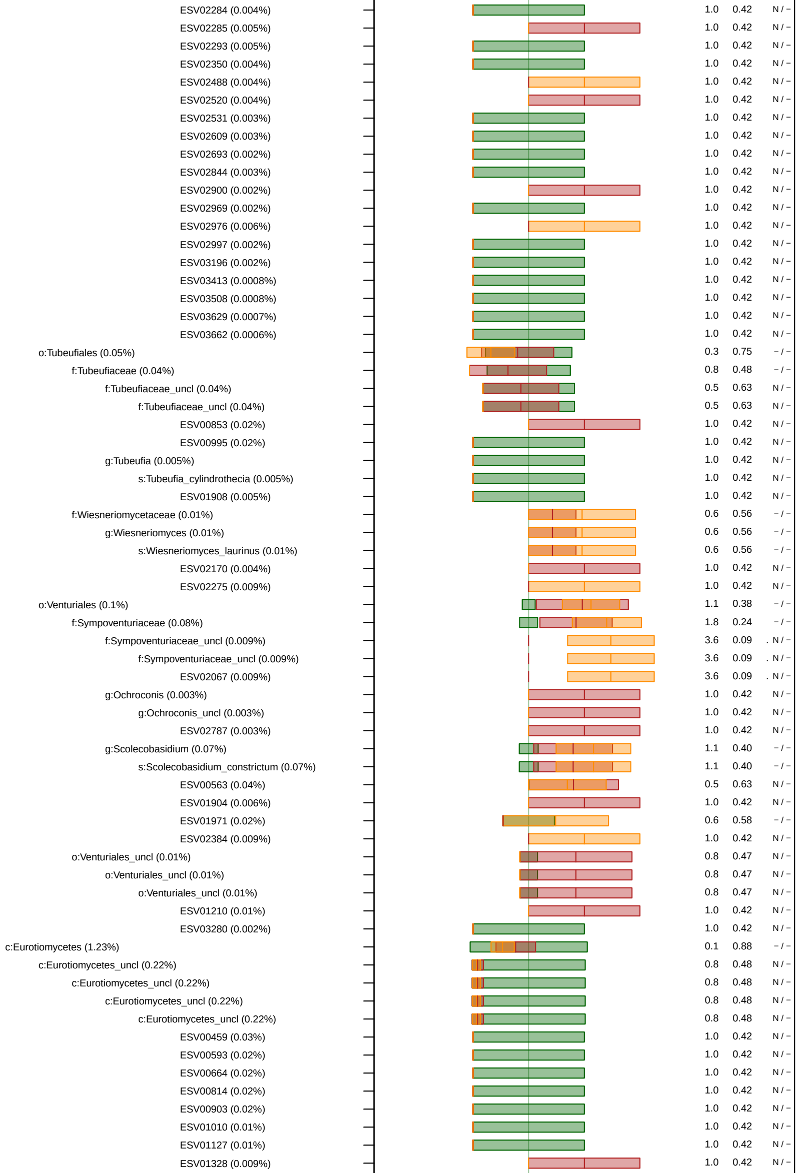

ESV02284 (0.004%) 1.0 0.42 N / –
ESV02285 (0.005%) 1.0 0.42 N / –
ESV02293 (0.005%) 1.0 0.42 N / –
ESV02350 (0.004%) 1.0 0.42 N / –
ESV02488 (0.004%) 1.0 0.42 N / –
ESV02520 (0.004%) 1.0 0.42 N / –
ESV02531 (0.003%) 1.0 0.42 N / –
ESV02609 (0.003%) 1.0 0.42 N / –
ESV02693 (0.002%) 1.0 0.42 N / –
ESV02844 (0.003%) 1.0 0.42 N / –
ESV02900 (0.002%) 1.0 0.42 N / –
ESV02969 (0.002%) 1.0 0.42 N / –
ESV02976 (0.006%) 1.0 0.42 N / –
ESV02997 (0.002%) 1.0 0.42 N / –
ESV03196 (0.002%) 1.0 0.42 N / –
ESV03413 (0.0008%) 1.0 0.42 N / –
ESV03508 (0.0008%) 1.0 0.42 N / –
ESV03629 (0.0007%) 1.0 0.42 N / –
ESV03662 (0.0006%) 1.0 0.42 N / –
o:Tubeufiales (0.05%) 0.3 0.75 – / –
f:Tubeufiaceae (0.04%) 0.8 0.48 – / –
f:Tubeufiaceae_uncl (0.04%) 0.5 0.63 N / –
f:Tubeufiaceae_uncl (0.04%) 0.5 0.63 N / –
ESV00853 (0.02%) 1.0 0.42 N / –
ESV00995 (0.02%) 1.0 0.42 N / –
g:Tubeufia (0.005%) 1.0 0.42 N / –
s:Tubeufia_cylindrothecia (0.005%) 1.0 0.42 N / –
ESV01908 (0.005%) 1.0 0.42 N / –
f:Wiesneriomycetaceae (0.01%) 0.6 0.56 – / –
g:Wiesneriomyces (0.01%) 0.6 0.56 – / –
s:Wiesneriomyces_laurinus (0.01%) 0.6 0.56 – / –
ESV02170 (0.004%) 1.0 0.42 N / –
ESV02275 (0.009%) 1.0 0.42 N / –
o:Venturiales (0.1%) 1.1 0.38 – / –
f:Sympoventuriaceae (0.08%) 1.8 0.24 – / –
f:Sympoventuriaceae_uncl (0.009%) 3.6 0.09 . N / –
f:Sympoventuriaceae_uncl (0.009%) 3.6 0.09 . N / –
ESV02067 (0.009%) 3.6 0.09 . N / –
g:Ochroconis (0.003%) 1.0 0.42 N / –
g:Ochroconis_uncl (0.003%) 1.0 0.42 N / –
ESV02787 (0.003%) 1.0 0.42 N / –
g:Scolecobasidium (0.07%) 1.1 0.40 – / –
s:Scolecobasidium_constrictum (0.07%) 1.1 0.40 – / –
ESV00563 (0.04%) 0.5 0.63 N / –
ESV01904 (0.006%) 1.0 0.42 N / –
ESV01971 (0.02%) 0.6 0.58 – / –
ESV02384 (0.009%) 1.0 0.42 N / –
o:Venturiales_uncl (0.01%) 0.8 0.47 N / –
o:Venturiales_uncl (0.01%) 0.8 0.47 N / –
o:Venturiales_uncl (0.01%) 0.8 0.47 N / –
ESV01210 (0.01%) 1.0 0.42 N / –
ESV03280 (0.002%) 1.0 0.42 N / –
c:Eurotiomycetes (1.23%) 0.1 0.88 – / –
c:Eurotiomycetes_uncl (0.22%) 0.8 0.48 N / –
c:Eurotiomycetes_uncl (0.22%) 0.8 0.48 N / –
c:Eurotiomycetes_uncl (0.22%) 0.8 0.48 N / –
c:Eurotiomycetes_uncl (0.22%) 0.8 0.48 N / –
ESV00459 (0.03%) 1.0 0.42 N / –
ESV00593 (0.02%) 1.0 0.42 N / –
ESV00664 (0.02%) 1.0 0.42 N / –
ESV00814 (0.02%) 1.0 0.42 N / –
ESV00903 (0.02%) 1.0 0.42 N / –
ESV01010 (0.01%) 1.0 0.42 N / –
ESV01127 (0.01%) 1.0 0.42 N / –
ESV01328 (0.009%) 1.0 0.42 N / –

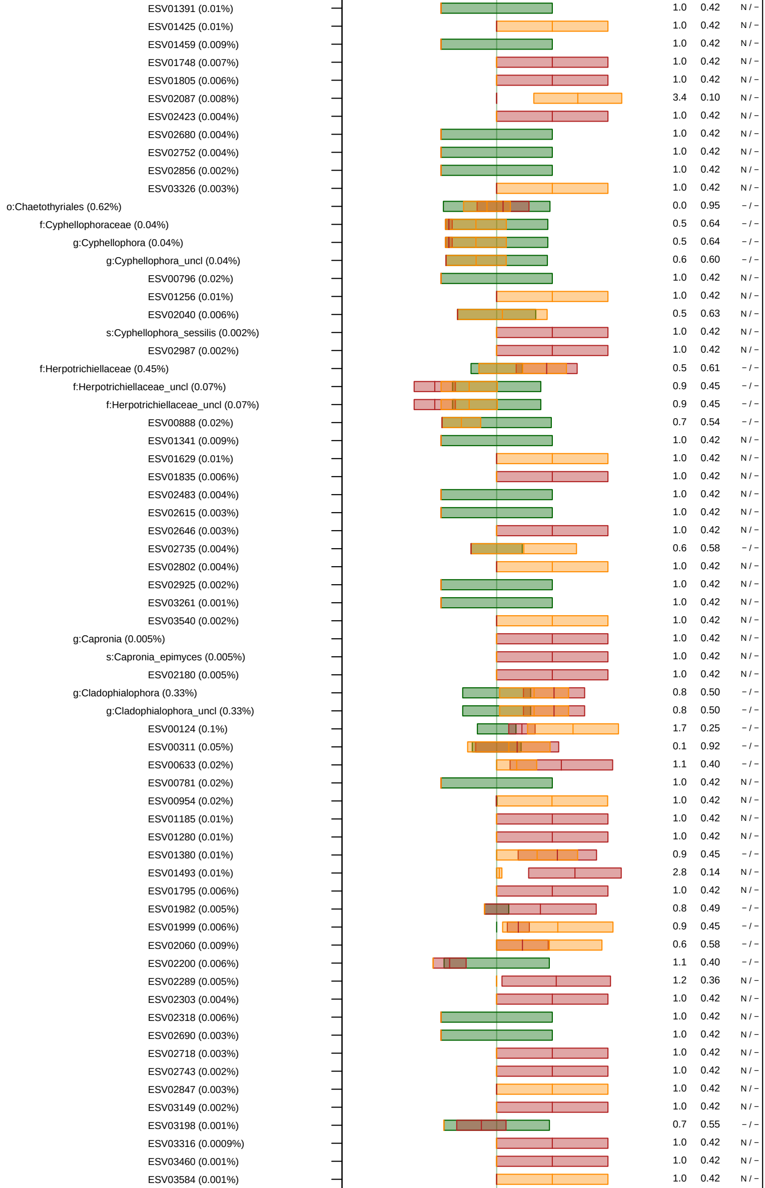
ESV01391 (0.01%) 1.0 0.42 N / –
ESV01425 (0.01%) 1.0 0.42 N / –
ESV01459 (0.009%) 1.0 0.42 N / –
ESV01748 (0.007%) 1.0 0.42 N / –
ESV01805 (0.006%) 1.0 0.42 N / –
ESV02087 (0.008%) 3.4 0.10 N / –
ESV02423 (0.004%) 1.0 0.42 N / –
ESV02680 (0.004%) 1.0 0.42 N / –
ESV02752 (0.004%) 1.0 0.42 N / –
ESV02856 (0.002%) 1.0 0.42 N / –
ESV03326 (0.003%) 1.0 0.42 N / –
o:Chaetothyriales (0.62%) 0.0 0.95 – / –
f:Cyphellophoraceae (0.04%) 0.5 0.64 – / –
g:Cyphellophora (0.04%) 0.5 0.64 – / –
g:Cyphellophora_uncl (0.04%) 0.6 0.60 – / –
ESV00796 (0.02%) 1.0 0.42 N / –
ESV01256 (0.01%) 1.0 0.42 N / –
ESV02040 (0.006%) 0.5 0.63 N / –
s:Cyphellophora_sessilis (0.002%) 1.0 0.42 N / –
ESV02987 (0.002%) 1.0 0.42 N / –
f:Herpotrichiellaceae (0.45%) 0.5 0.61 – / –
f:Herpotrichiellaceae_uncl (0.07%) 0.9 0.45 – / –
f:Herpotrichiellaceae_uncl (0.07%) 0.9 0.45 – / –
ESV00888 (0.02%) 0.7 0.54 – / –
ESV01341 (0.009%) 1.0 0.42 N / –
ESV01629 (0.01%) 1.0 0.42 N / –
ESV01835 (0.006%) 1.0 0.42 N / –
ESV02483 (0.004%) 1.0 0.42 N / –
ESV02615 (0.003%) 1.0 0.42 N / –
ESV02646 (0.003%) 1.0 0.42 N / –
ESV02735 (0.004%) 0.6 0.58 – / –
ESV02802 (0.004%) 1.0 0.42 N / –
ESV02925 (0.002%) 1.0 0.42 N / –
ESV03261 (0.001%) 1.0 0.42 N / –
ESV03540 (0.002%) 1.0 0.42 N / –
g:Capronia (0.005%) 1.0 0.42 N / –
s:Capronia_epimyces (0.005%) 1.0 0.42 N / –
ESV02180 (0.005%) 1.0 0.42 N / –
g:Cladophialophora (0.33%) 0.8 0.50 – / –
g:Cladophialophora_uncl (0.33%) 0.8 0.50 – / –
ESV00124 (0.1%) 1.7 0.25 – / –
ESV00311 (0.05%) 0.1 0.92 – / –
ESV00633 (0.02%) 1.1 0.40 – / –
ESV00781 (0.02%) 1.0 0.42 N / –
ESV00954 (0.02%) 1.0 0.42 N / –
ESV01185 (0.01%) 1.0 0.42 N / –
ESV01280 (0.01%) 1.0 0.42 N / –
ESV01380 (0.01%) 0.9 0.45 – / –
ESV01493 (0.01%) 2.8 0.14 N / –
ESV01795 (0.006%) 1.0 0.42 N / –
ESV01982 (0.005%) 0.8 0.49 – / –
ESV01999 (0.006%) 0.9 0.45 – / –
ESV02060 (0.009%) 0.6 0.58 – / –
ESV02200 (0.006%) 1.1 0.40 – / –
ESV02289 (0.005%) 1.2 0.36 N / –
ESV02303 (0.004%) 1.0 0.42 N / –
ESV02318 (0.006%) 1.0 0.42 N / –
ESV02690 (0.003%) 1.0 0.42 N / –
ESV02718 (0.003%) 1.0 0.42 N / –
ESV02743 (0.002%) 1.0 0.42 N / –
ESV02847 (0.003%) 1.0 0.42 N / –
ESV03149 (0.002%) 1.0 0.42 N / –
ESV03198 (0.001%) 0.7 0.55 – / –
ESV03316 (0.0009%) 1.0 0.42 N / –
ESV03460 (0.001%) 1.0 0.42 N / –
ESV03584 (0.001%) 1.0 0.42 N / –

g:Exophiala (0.03%)	0.8	0.51		– / –
s:Exophiala_cancerae (0.002%)	1.0	0.42		N / –
ESV03313 (0.002%)	1.0	0.42		N / –
s:Exophiala_dermatitidis (0.006%)	1.0	0.42		N / –
ESV02213 (0.006%)	1.0	0.42		N / –
s:Exophiala_pisciphila (0.02%)	1.2	0.37		– / –
ESV00717 (0.02%)	1.2	0.37		– / –
g:Fonsecaea (0.009%)	0.7	0.52		– / –
g:Fonsecaea_uncl (0.001%)	1.0	0.42		N / –
ESV03253 (0.001%)	1.0	0.42		N / –
s:Fonsecaea_pedrosoi (0.008%)	0.8	0.49		N / –
ESV02751 (0.006%)	1.0	0.42		N / –
ESV03379 (0.001%)	1.0	0.42		N / –
g:Veronaea (0.004%)	1.0	0.42		N / –
g:Veronaea_uncl (0.004%)	1.0	0.42		N / –
ESV02989 (0.004%)	1.0	0.42		N / –
f:Trichomeriaceae (0.09%)	0.8	0.50		– / –
f:Trichomeriaceae_uncl (0.05%)	1.0	0.41		– / –
f:Trichomeriaceae_uncl (0.05%)	1.0	0.41		– / –
ESV00529 (0.03%)	1.1	0.39		N / –
ESV01199 (0.01%)	0.7	0.53		– / –
g:Arthrocladium (0.04%)	0.1	0.88		– / –
g:Arthrocladium_uncl (0.04%)	0.1	0.88		– / –
ESV00536 (0.03%)	0.6	0.58		– / –
ESV01726 (0.01%)	1.0	0.42		N / –
ESV02247 (0.004%)	1.0	0.42		N / –
ESV03205 (0.002%)	1.0	0.42		N / –
o:Chaetothyriales_uncl (0.04%)	0.5	0.65		– / –
o:Chaetothyriales_uncl (0.04%)	0.5	0.65		– / –
o:Chaetothyriales_uncl (0.04%)	0.5	0.65		– / –
ESV01502 (0.008%)	1.0	0.42		N / –
ESV01525 (0.02%)	0.6	0.59		– / –
ESV02141 (0.004%)	1.0	0.42		N / –
ESV02895 (0.004%)	1.0	0.42		N / –
ESV02960 (0.005%)	1.0	0.42		N / –
o:Eurotiales (0.38%)	3.0	0.12		– / –
f:Aspergillaceae (0.2%)	4.8	0.06	.	– / –
f:Aspergillaceae_uncl (0.005%)	0.2	0.83		– / –
f:Aspergillaceae_uncl (0.005%)	0.2	0.83		– / –
ESV03359 (0.002%)	1.0	0.42		N / –
ESV03385 (0.001%)	1.5	0.30		– / –
ESV03640 (0.0008%)	1.0	0.42		N / –
ESV03675 (0.0008%)	1.0	0.42		N / –
g:Aspergillus (0.03%)	3.3	0.11		– / –
g:Aspergillus_uncl (0.02%)	2.9	0.13		– / –
ESV01992 (0.01%)	1.9	0.22		– / –
ESV02564 (0.005%)	0.7	0.55		– / –
ESV02687 (0.005%)	1.5	0.30		N / –
ESV03112 (0.003%)	0.6	0.58		– / –
ESV03469 (0.001%)	1.0	0.42		N / –
s:Aspergillus_auratus (0.002%)	0.5	0.63		N / –
ESV03035 (0.002%)	0.5	0.63		N / –
s:Aspergillus_otanii (0.003%)	1.5	0.29		– / –
ESV02916 (0.003%)	1.5	0.29		– / –
s:Aspergillus_penicillioides (0.0007%)	1.0	0.42		N / –
ESV03643 (0.0007%)	1.0	0.42		N / –
g:Penicillium (0.16%)	4.2	0.07	.	– / –
g:Penicillium_uncl (0.1%)	6.5	0.03	*	– / –
ESV00492 (0.03%)	2.9	0.13		– / –
ESV00648 (0.03%)	2.6	0.15		N / –
ESV01491 (0.01%)	5.2	0.05	*	– / –
ESV01991 (0.006%)	2.3	0.18		– / –
ESV02814 (0.003%)	5.7	0.04	*	N / –
ESV02871 (0.002%)	0.6	0.58		– / –
ESV02933 (0.002%)	1.8	0.24		N / –
ESV03030 (0.002%)	1.0	0.42		N / –

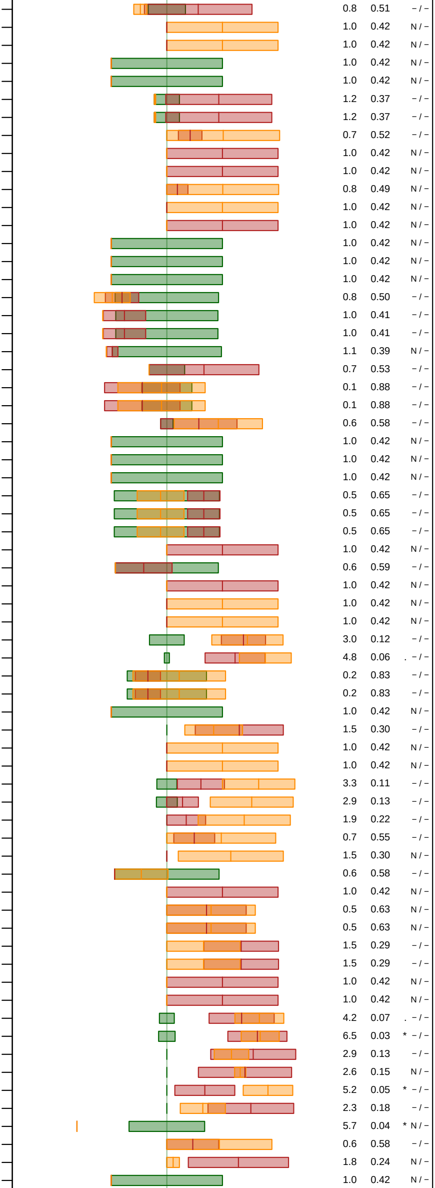

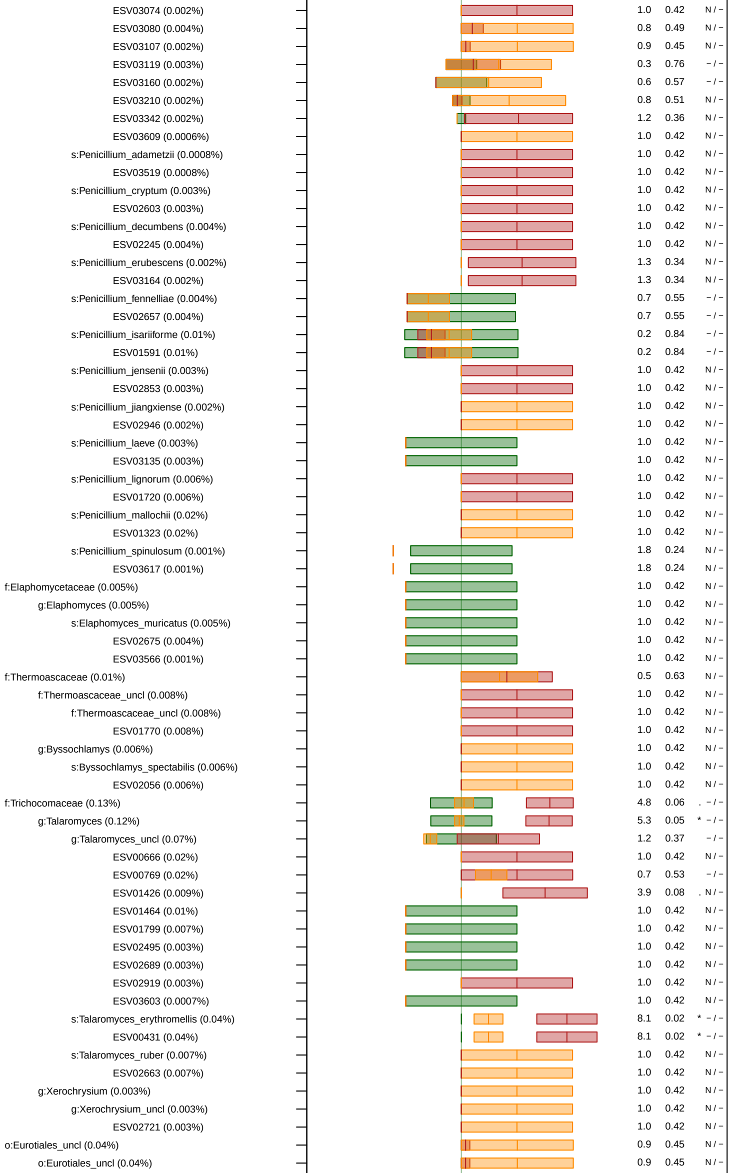
ESV03074 (0.002%) 1.0 0.42 N / –
ESV03080 (0.004%) 0.8 0.49 N / –
ESV03107 (0.002%) 0.9 0.45 N / –
ESV03119 (0.003%) 0.3 0.76 – / –
ESV03160 (0.002%) 0.6 0.57 – / –
ESV03210 (0.002%) 0.8 0.51 N / –
ESV03342 (0.002%) 1.2 0.36 N / –
ESV03609 (0.0006%) 1.0 0.42 N / –
s:Penicillium_adametzii (0.0008%) 1.0 0.42 N / –
ESV03519 (0.0008%) 1.0 0.42 N / –
s:Penicillium_cryptum (0.003%) 1.0 0.42 N / –
ESV02603 (0.003%) 1.0 0.42 N / –
s:Penicillium_decumbens (0.004%) 1.0 0.42 N / –
ESV02245 (0.004%) 1.0 0.42 N / –
s:Penicillium_erubescens (0.002%) 1.3 0.34 N / –
ESV03164 (0.002%) 1.3 0.34 N / –
s:Penicillium_fennelliae (0.004%) 0.7 0.55 – / –
ESV02657 (0.004%) 0.7 0.55 – / –
s:Penicillium_isariiforme (0.01%) 0.2 0.84 – / –
ESV01591 (0.01%) 0.2 0.84 – / –
s:Penicillium_jensenii (0.003%) 1.0 0.42 N / –
ESV02853 (0.003%) 1.0 0.42 N / –
s:Penicillium_jiangxiense (0.002%) 1.0 0.42 N / –
ESV02946 (0.002%) 1.0 0.42 N / –
s:Penicillium_laeve (0.003%) 1.0 0.42 N / –
ESV03135 (0.003%) 1.0 0.42 N / –
s:Penicillium_lignorum (0.006%) 1.0 0.42 N / –
ESV01720 (0.006%) 1.0 0.42 N / –
s:Penicillium_mallochii (0.02%) 1.0 0.42 N / –
ESV01323 (0.02%) 1.0 0.42 N / –
s:Penicillium_spinulosum (0.001%) 1.8 0.24 N / –
ESV03617 (0.001%) 1.8 0.24 N / –
f:Elaphomycetaceae (0.005%) 1.0 0.42 N / –
g:Elaphomyces (0.005%) 1.0 0.42 N / –
s:Elaphomyces_muricatus (0.005%) 1.0 0.42 N / –
ESV02675 (0.004%) 1.0 0.42 N / –
ESV03566 (0.001%) 1.0 0.42 N / –
f:Thermoascaceae (0.01%) 0.5 0.63 N / –
f:Thermoascaceae_uncl (0.008%) 1.0 0.42 N / –
f:Thermoascaceae_uncl (0.008%) 1.0 0.42 N / –
ESV01770 (0.008%) 1.0 0.42 N / –
g:Byssochlamys (0.006%) 1.0 0.42 N / –
s:Byssochlamys_spectabilis (0.006%) 1.0 0.42 N / –
ESV02056 (0.006%) 1.0 0.42 N / –
f:Trichocomaceae (0.13%) 4.8 0.06 . – / –
g:Talaromyces (0.12%) 5.3 0.05 * – / –
g:Talaromyces_uncl (0.07%) 1.2 0.37 – / –
ESV00666 (0.02%) 1.0 0.42 N / –
ESV00769 (0.02%) 0.7 0.53 – / –
ESV01426 (0.009%) 3.9 0.08 . N / –
ESV01464 (0.01%) 1.0 0.42 N / –
ESV01799 (0.007%) 1.0 0.42 N / –
ESV02495 (0.003%) 1.0 0.42 N / –
ESV02689 (0.003%) 1.0 0.42 N / –
ESV02919 (0.003%) 1.0 0.42 N / –
ESV03603 (0.0007%) 1.0 0.42 N / –
s:Talaromyces_erythromellis (0.04%) 8.1 0.02 * – / –
ESV00431 (0.04%) 8.1 0.02 * – / –
s:Talaromyces_ruber (0.007%) 1.0 0.42 N / –
ESV02663 (0.007%) 1.0 0.42 N / –
g:Xerochrysium (0.003%) 1.0 0.42 N / –
g:Xerochrysium_uncl (0.003%) 1.0 0.42 N / –
ESV02721 (0.003%) 1.0 0.42 N / –
o:Eurotiales_uncl (0.04%) 0.9 0.45 N / –
o:Eurotiales_uncl (0.04%) 0.9 0.45 N / –

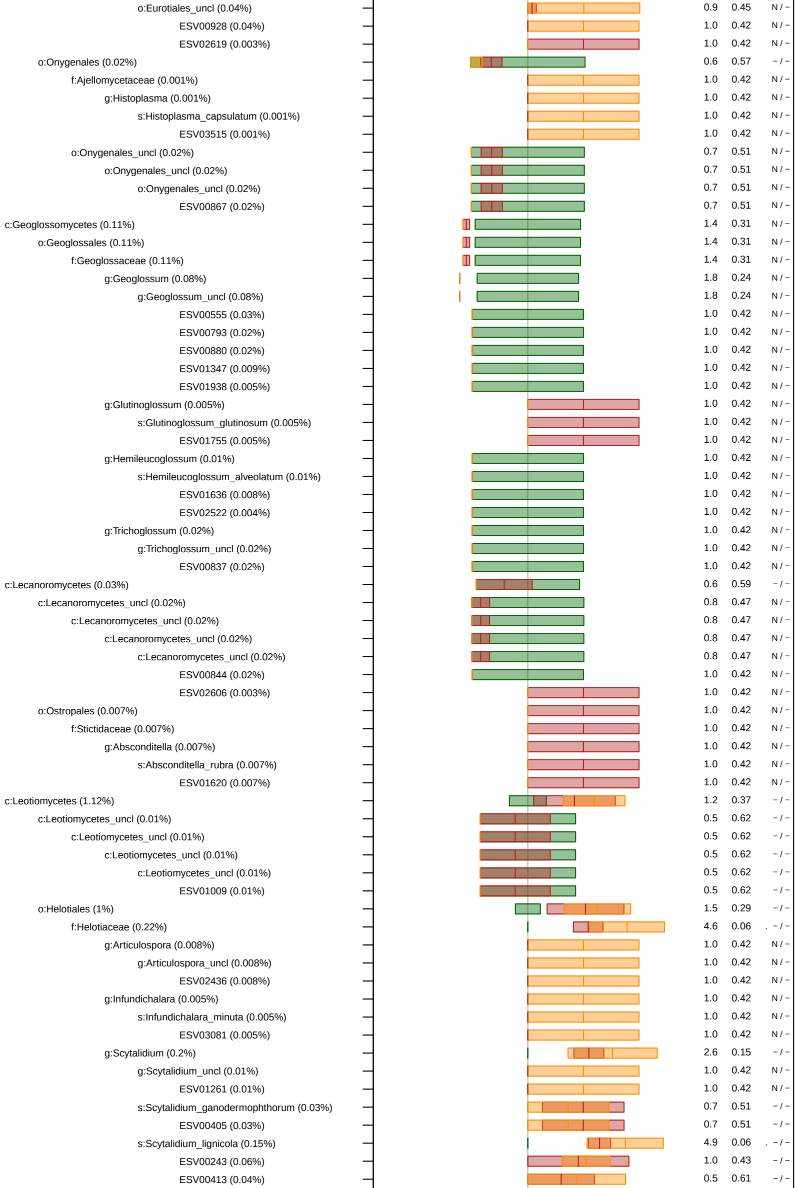
o:Eurotiales_uncl (0.04%) 0.9 0.45 N / –
ESV00928 (0.04%) 1.0 0.42 N / –
ESV02619 (0.003%) 1.0 0.42 N / –
o:Onygenales (0.02%) 0.6 0.57 – / –
f:Ajellomycetaceae (0.001%) 1.0 0.42 N / –
g:Histoplasma (0.001%) 1.0 0.42 N / –
s:Histoplasma_capsulatum (0.001%) 1.0 0.42 N / –
ESV03515 (0.001%) 1.0 0.42 N / –
o:Onygenales_uncl (0.02%) 0.7 0.51 N / –
o:Onygenales_uncl (0.02%) 0.7 0.51 N / –
o:Onygenales_uncl (0.02%) 0.7 0.51 N / –
ESV00867 (0.02%) 0.7 0.51 N / –
c:Geoglossomycetes (0.11%) 1.4 0.31 N / –
o:Geoglossales (0.11%) 1.4 0.31 N / –
f:Geoglossaceae (0.11%) 1.4 0.31 N / –
g:Geoglossum (0.08%) 1.8 0.24 N / –
g:Geoglossum_uncl (0.08%) 1.8 0.24 N / –
ESV00555 (0.03%) 1.0 0.42 N / –
ESV00793 (0.02%) 1.0 0.42 N / –
ESV00880 (0.02%) 1.0 0.42 N / –
ESV01347 (0.009%) 1.0 0.42 N / –
ESV01938 (0.005%) 1.0 0.42 N / –
g:Glutinoglossum (0.005%) 1.0 0.42 N / –
s:Glutinoglossum_glutinosum (0.005%) 1.0 0.42 N / –
ESV01755 (0.005%) 1.0 0.42 N / –
g:Hemileucoglossum (0.01%) 1.0 0.42 N / –
s:Hemileucoglossum_alveolatum (0.01%) 1.0 0.42 N / –
ESV01636 (0.008%) 1.0 0.42 N / –
ESV02522 (0.004%) 1.0 0.42 N / –
g:Trichoglossum (0.02%) 1.0 0.42 N / –
g:Trichoglossum_uncl (0.02%) 1.0 0.42 N / –
ESV00837 (0.02%) 1.0 0.42 N / –
c:Lecanoromycetes (0.03%) 0.6 0.59 – / –
c:Lecanoromycetes_uncl (0.02%) 0.8 0.47 N / –
c:Lecanoromycetes_uncl (0.02%) 0.8 0.47 N / –
c:Lecanoromycetes_uncl (0.02%) 0.8 0.47 N / –
c:Lecanoromycetes_uncl (0.02%) 0.8 0.47 N / –
ESV00844 (0.02%) 1.0 0.42 N / –
ESV02606 (0.003%) 1.0 0.42 N / –
o:Ostropales (0.007%) 1.0 0.42 N / –
f:Stictidaceae (0.007%) 1.0 0.42 N / –
g:Absconditella (0.007%) 1.0 0.42 N / –
s:Absconditella_rubra (0.007%) 1.0 0.42 N / –
ESV01620 (0.007%) 1.0 0.42 N / –
c:Leotiomycetes (1.12%) 1.2 0.37 – / –
c:Leotiomycetes_uncl (0.01%) 0.5 0.62 – / –
c:Leotiomycetes_uncl (0.01%) 0.5 0.62 – / –
c:Leotiomycetes_uncl (0.01%) 0.5 0.62 – / –
c:Leotiomycetes_uncl (0.01%) 0.5 0.62 – / –
ESV01009 (0.01%) 0.5 0.62 – / –
o:Helotiales (1%) 1.5 0.29 – / –
f:Helotiaceae (0.22%) 4.6 0.06 . – / –
g:Articulospora (0.008%) 1.0 0.42 N / –
g:Articulospora_uncl (0.008%) 1.0 0.42 N / –
ESV02436 (0.008%) 1.0 0.42 N / –
g:Infundichalara (0.005%) 1.0 0.42 N / –
s:Infundichalara_minuta (0.005%) 1.0 0.42 N / –
ESV03081 (0.005%) 1.0 0.42 N / –
g:Scytalidium (0.2%) 2.6 0.15 – / –
g:Scytalidium_uncl (0.01%) 1.0 0.42 N / –
ESV01261 (0.01%) 1.0 0.42 N / –
s:Scytalidium_ganodermophthorum (0.03%) 0.7 0.51 – / –
ESV00405 (0.03%) 0.7 0.51 – / –
s:Scytalidium_lignicola (0.15%) 4.9 0.06 . – / –
ESV00243 (0.06%) 1.0 0.43 – / –
ESV00413 (0.04%) 0.5 0.61 – / –

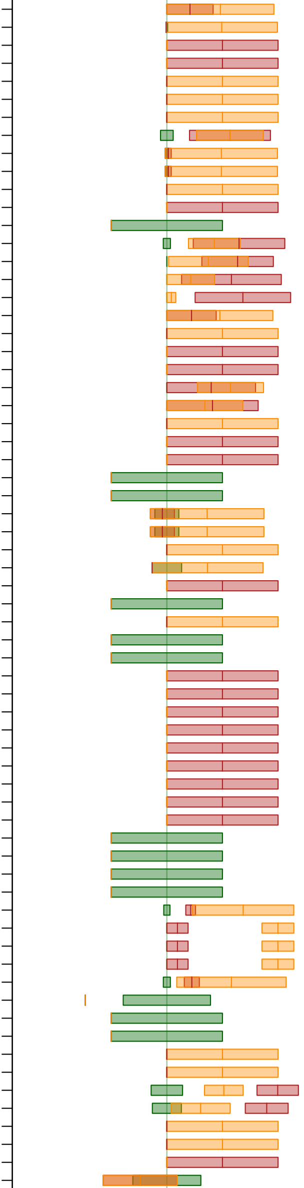

ESV00456 (0.03%)	0.6	0.56		– / –
ESV01824 (0.009%)	1.0	0.43		N / –
ESV01829 (0.005%)	1.0	0.42		N / –
ESV02034 (0.006%)	1.0	0.42		N / –
g:Tetracladium (0.009%)	1.0	0.42		N / –
g:Tetracladium_uncl (0.009%)	1.0	0.42		N / –
ESV01580 (0.009%)	1.0	0.42		N / –
f:Helotiales_inc_sed (0.4%)	1.4	0.31		– / –
f:Helotiales_inc_sed_uncl (0.06%)	0.9	0.45		N / –
f:Helotiales_inc_sed_uncl (0.06%)	0.9	0.45		N / –
ESV00556 (0.05%)	1.0	0.42		N / –
ESV02768 (0.003%)	1.0	0.42		N / –
ESV03123 (0.001%)	1.0	0.42		N / –
g:Chalara (0.24%)	1.5	0.31		– / –
g:Chalara_uncl (0.18%)	1.3	0.33		– / –
ESV00168 (0.08%)	1.0	0.41		– / –
ESV00380 (0.03%)	2.4	0.17		N / –
ESV00991 (0.02%)	0.6	0.57		– / –
ESV01132 (0.02%)	1.0	0.42		N / –
ESV01842 (0.006%)	1.0	0.42		N / –
ESV01969 (0.006%)	1.0	0.42		N / –
s:Chalara_clidemiae (0.05%)	1.0	0.41		– / –
ESV00364 (0.04%)	0.5	0.63		N / –
ESV01121 (0.01%)	1.0	0.42		N / –
s:Chalara_holubovae (0.009%)	1.0	0.42		N / –
ESV01334 (0.009%)	1.0	0.42		N / –
s:Chalara_pseudoaffinis (0.007%)	1.0	0.42		N / –
ESV01669 (0.007%)	1.0	0.42		N / –
g:Leohumicola (0.07%)	0.5	0.62		– / –
g:Leohumicola_uncl (0.07%)	0.5	0.62		– / –
ESV00708 (0.02%)	1.0	0.42		N / –
ESV00915 (0.02%)	0.8	0.51		– / –
ESV01282 (0.009%)	1.0	0.42		N / –
ESV02331 (0.004%)	1.0	0.42		N / –
ESV02780 (0.004%)	1.0	0.42		N / –
ESV02822 (0.002%)	1.0	0.42		N / –
ESV02954 (0.002%)	1.0	0.42		N / –
g:Leptodontidium (0.008%)	1.0	0.42		N / –
g:Leptodontidium_uncl (0.008%)	1.0	0.42		N / –
ESV01611 (0.008%)	1.0	0.42		N / –
g:Xenochalara (0.003%)	1.0	0.42		N / –
g:Xenochalara_uncl (0.003%)	1.0	0.42		N / –
ESV02746 (0.003%)	1.0	0.42		N / –
g:Xylogone (0.03%)	1.0	0.42		N / –
g:Xylogone_uncl (0.03%)	1.0	0.42		N / –
ESV00463 (0.03%)	1.0	0.42		N / –
f:Hyaloscyphaceae (0.007%)	1.0	0.42		N / –
g:Bactrodesmium (0.007%)	1.0	0.42		N / –
s:Bactrodesmium_longisporum (0.007%)	1.0	0.42		N / –
ESV01584 (0.007%)	1.0	0.42		N / –
f:Myxotrichaceae (0.12%)	1.8	0.25		N / –
f:Myxotrichaceae_uncl (0.02%)	30.7	< 0.001	***	– / –
f:Myxotrichaceae_uncl (0.02%)	30.7	< 0.001	***	– / –
ESV01164 (0.02%)	30.7	< 0.001	***	– / –
g:Oidiodendron (0.1%)	1.0	0.41		– / –
g:Oidiodendron_uncl (0.01%)	3.5	0.10	.	N / –
ESV01951 (0.006%)	1.0	0.42		N / –
ESV02635 (0.004%)	1.0	0.42		N / –
s:Oidiodendron_griseum (0.04%)	1.0	0.42		N / –
ESV00734 (0.04%)	1.0	0.42		N / –
s:Oidiodendron_maius (0.05%)	8.8	0.02	*	– / –
ESV00304 (0.05%)	5.0	0.05	.	– / –
ESV02364 (0.004%)	1.0	0.42		N / –
ESV03543 (0.0008%)	1.0	0.42		N / –
ESV03596 (0.0006%)	1.0	0.42		N / –
f:Vibrisseaceae (0.009%)	0.2	0.84		N / –

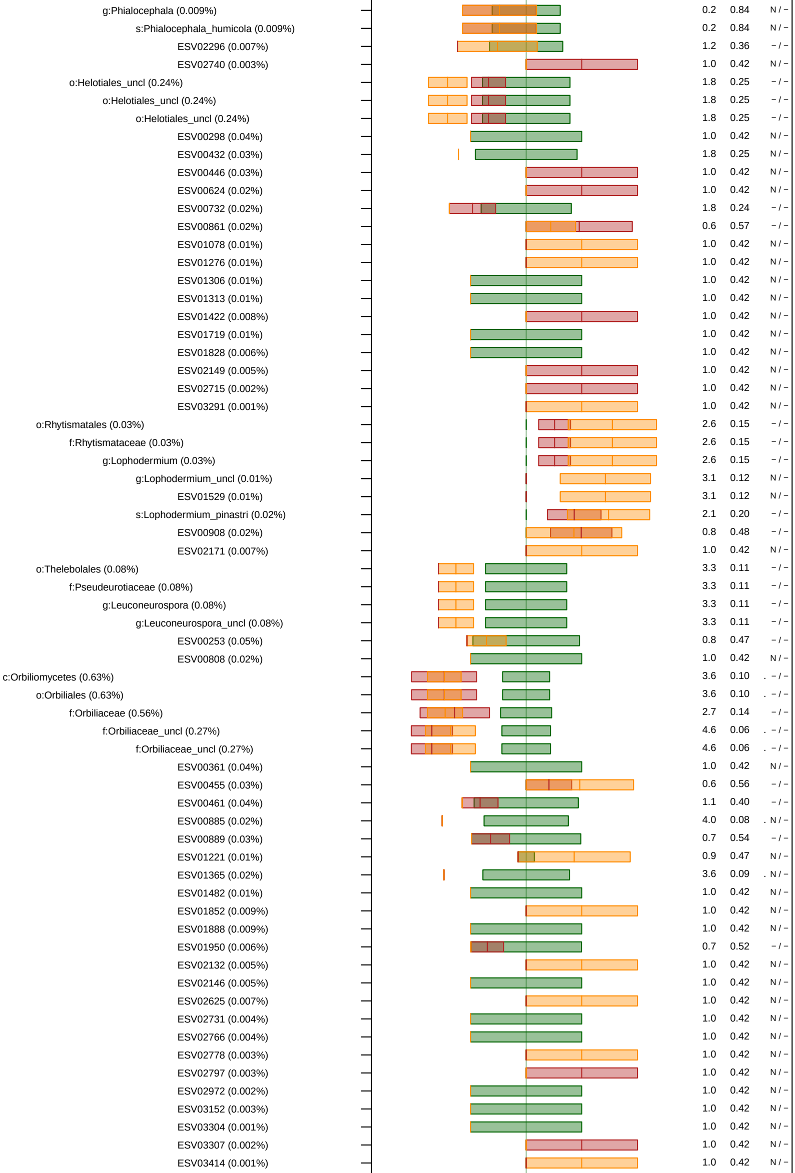

g:Phialocephala (0.009%) 0.2 0.84 N / –
s:Phialocephala_humicola (0.009%) 0.2 0.84 N / –
ESV02296 (0.007%) 1.2 0.36 – / –
ESV02740 (0.003%) 1.0 0.42 N / –
o:Helotiales_uncl (0.24%) 1.8 0.25 – / –
o:Helotiales_uncl (0.24%) 1.8 0.25 – / –
o:Helotiales_uncl (0.24%) 1.8 0.25 – / –
ESV00298 (0.04%) 1.0 0.42 N / –
ESV00432 (0.03%) 1.8 0.25 N / –
ESV00446 (0.03%) 1.0 0.42 N / –
ESV00624 (0.02%) 1.0 0.42 N / –
ESV00732 (0.02%) 1.8 0.24 – / –
ESV00861 (0.02%) 0.6 0.57 – / –
ESV01078 (0.01%) 1.0 0.42 N / –
ESV01276 (0.01%) 1.0 0.42 N / –
ESV01306 (0.01%) 1.0 0.42 N / –
ESV01313 (0.01%) 1.0 0.42 N / –
ESV01422 (0.008%) 1.0 0.42 N / –
ESV01719 (0.01%) 1.0 0.42 N / –
ESV01828 (0.006%) 1.0 0.42 N / –
ESV02149 (0.005%) 1.0 0.42 N / –
ESV02715 (0.002%) 1.0 0.42 N / –
ESV03291 (0.001%) 1.0 0.42 N / –
o:Rhytismatales (0.03%) 2.6 0.15 – / –
f:Rhytismataceae (0.03%) 2.6 0.15 – / –
g:Lophodermium (0.03%) 2.6 0.15 – / –
g:Lophodermium_uncl (0.01%) 3.1 0.12 N / –
ESV01529 (0.01%) 3.1 0.12 N / –
s:Lophodermium_pinastri (0.02%) 2.1 0.20 – / –
ESV00908 (0.02%) 0.8 0.48 – / –
ESV02171 (0.007%) 1.0 0.42 N / –
o:Thelebolales (0.08%) 3.3 0.11 – / –
f:Pseudeurotiaceae (0.08%) 3.3 0.11 – / –
g:Leuconeurospora (0.08%) 3.3 0.11 – / –
g:Leuconeurospora_uncl (0.08%) 3.3 0.11 – / –
ESV00253 (0.05%) 0.8 0.47 – / –
ESV00808 (0.02%) 1.0 0.42 N / –
c:Orbiliomycetes (0.63%) 3.6 0.10 . – / –
o:Orbiliales (0.63%) 3.6 0.10 . – / –
f:Orbiliaceae (0.56%) 2.7 0.14 – / –
f:Orbiliaceae_uncl (0.27%) 4.6 0.06 . – / –
f:Orbiliaceae_uncl (0.27%) 4.6 0.06 . – / –
ESV00361 (0.04%) 1.0 0.42 N / –
ESV00455 (0.03%) 0.6 0.56 – / –
ESV00461 (0.04%) 1.1 0.40 – / –
ESV00885 (0.02%) 4.0 0.08 . N / –
ESV00889 (0.03%) 0.7 0.54 – / –
ESV01221 (0.01%) 0.9 0.47 N / –
ESV01365 (0.02%) 3.6 0.09 . N / –
ESV01482 (0.01%) 1.0 0.42 N / –
ESV01852 (0.009%) 1.0 0.42 N / –
ESV01888 (0.009%) 1.0 0.42 N / –
ESV01950 (0.006%) 0.7 0.52 – / –
ESV02132 (0.005%) 1.0 0.42 N / –
ESV02146 (0.005%) 1.0 0.42 N / –
ESV02625 (0.007%) 1.0 0.42 N / –
ESV02731 (0.004%) 1.0 0.42 N / –
ESV02766 (0.004%) 1.0 0.42 N / –
ESV02778 (0.003%) 1.0 0.42 N / –
ESV02797 (0.003%) 1.0 0.42 N / –
ESV02972 (0.002%) 1.0 0.42 N / –
ESV03152 (0.003%) 1.0 0.42 N / –
ESV03304 (0.001%) 1.0 0.42 N / –
ESV03307 (0.002%) 1.0 0.42 N / –
ESV03414 (0.001%) 1.0 0.42 N / –

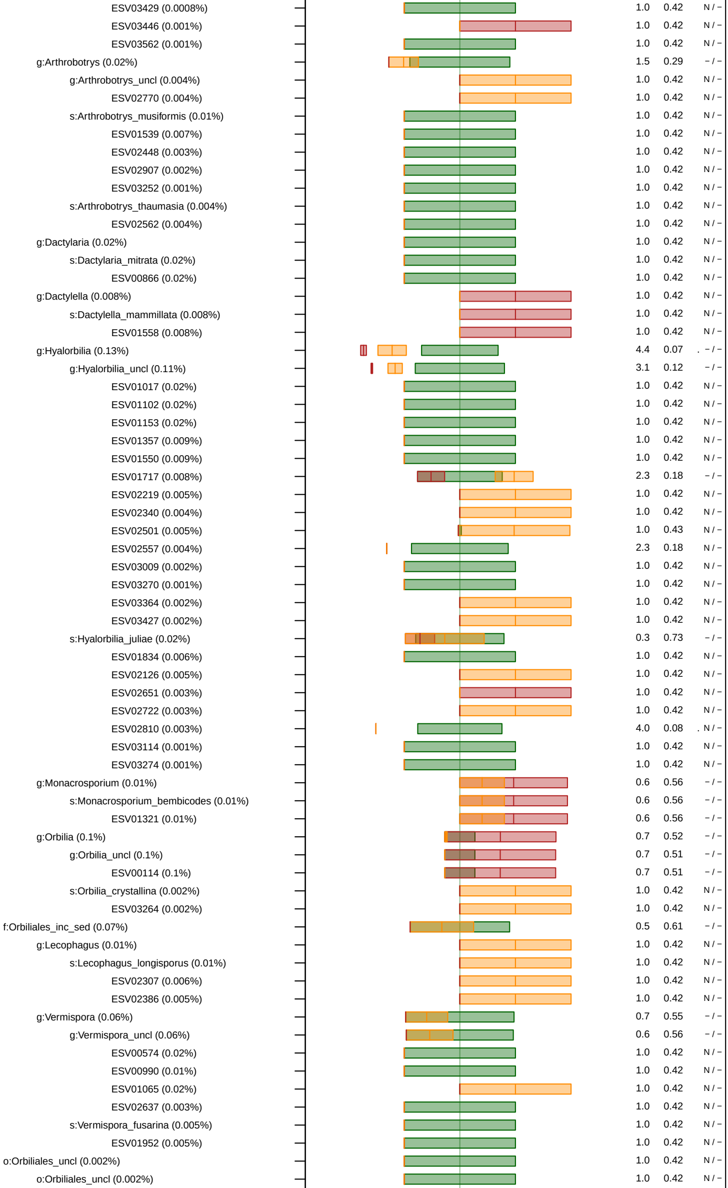

ESV03429 (0.0008%) 1.0 0.42 N / –
ESV03446 (0.001%) 1.0 0.42 N / –
ESV03562 (0.001%) 1.0 0.42 N / –
g:Arthrobotrys (0.02%) 1.5 0.29 – / –
g:Arthrobotrys_uncl (0.004%) 1.0 0.42 N / –
ESV02770 (0.004%) 1.0 0.42 N / –
s:Arthrobotrys_musiformis (0.01%) 1.0 0.42 N / –
ESV01539 (0.007%) 1.0 0.42 N / –
ESV02448 (0.003%) 1.0 0.42 N / –
ESV02907 (0.002%) 1.0 0.42 N / –
ESV03252 (0.001%) 1.0 0.42 N / –
s:Arthrobotrys_thaumasia (0.004%) 1.0 0.42 N / –
ESV02562 (0.004%) 1.0 0.42 N / –
g:Dactylaria (0.02%) 1.0 0.42 N / –
s:Dactylaria_mitrata (0.02%) 1.0 0.42 N / –
ESV00866 (0.02%) 1.0 0.42 N / –
g:Dactylella (0.008%) 1.0 0.42 N / –
s:Dactylella_mammillata (0.008%) 1.0 0.42 N / –
ESV01558 (0.008%) 1.0 0.42 N / –
g:Hyalorbilia (0.13%) 4.4 0.07 . – / –
g:Hyalorbilia_uncl (0.11%) 3.1 0.12 – / –
ESV01017 (0.02%) 1.0 0.42 N / –
ESV01102 (0.02%) 1.0 0.42 N / –
ESV01153 (0.02%) 1.0 0.42 N / –
ESV01357 (0.009%) 1.0 0.42 N / –
ESV01550 (0.009%) 1.0 0.42 N / –
ESV01717 (0.008%) 2.3 0.18 – / –
ESV02219 (0.005%) 1.0 0.42 N / –
ESV02340 (0.004%) 1.0 0.42 N / –
ESV02501 (0.005%) 1.0 0.43 N / –
ESV02557 (0.004%) 2.3 0.18 N / –
ESV03009 (0.002%) 1.0 0.42 N / –
ESV03270 (0.001%) 1.0 0.42 N / –
ESV03364 (0.002%) 1.0 0.42 N / –
ESV03427 (0.002%) 1.0 0.42 N / –
s:Hyalorbilia_juliae (0.02%) 0.3 0.73 – / –
ESV01834 (0.006%) 1.0 0.42 N / –
ESV02126 (0.005%) 1.0 0.42 N / –
ESV02651 (0.003%) 1.0 0.42 N / –
ESV02722 (0.003%) 1.0 0.42 N / –
ESV02810 (0.003%) 4.0 0.08 . N / –
ESV03114 (0.001%) 1.0 0.42 N / –
ESV03274 (0.001%) 1.0 0.42 N / –
g:Monacrosporium (0.01%) 0.6 0.56 – / –
s:Monacrosporium_bembicodes (0.01%) 0.6 0.56 – / –
ESV01321 (0.01%) 0.6 0.56 – / –
g:Orbilia (0.1%) 0.7 0.52 – / –
g:Orbilia_uncl (0.1%) 0.7 0.51 – / –
ESV00114 (0.1%) 0.7 0.51 – / –
s:Orbilia_crystallina (0.002%) 1.0 0.42 N / –
ESV03264 (0.002%) 1.0 0.42 N / –
f:Orbiliales_inc_sed (0.07%) 0.5 0.61 – / –
g:Lecophagus (0.01%) 1.0 0.42 N / –
s:Lecophagus_longisporus (0.01%) 1.0 0.42 N / –
ESV02307 (0.006%) 1.0 0.42 N / –
ESV02386 (0.005%) 1.0 0.42 N / –
g:Vermispora (0.06%) 0.7 0.55 – / –
g:Vermispora_uncl (0.06%) 0.6 0.56 – / –
ESV00574 (0.02%) 1.0 0.42 N / –
ESV00990 (0.01%) 1.0 0.42 N / –
ESV01065 (0.02%) 1.0 0.42 N / –
ESV02637 (0.003%) 1.0 0.42 N / –
s:Vermispora_fusarina (0.005%) 1.0 0.42 N / –
ESV01952 (0.005%) 1.0 0.42 N / –
o:Orbiliales_uncl (0.002%) 1.0 0.42 N / –
o:Orbiliales_uncl (0.002%) 1.0 0.42 N / –

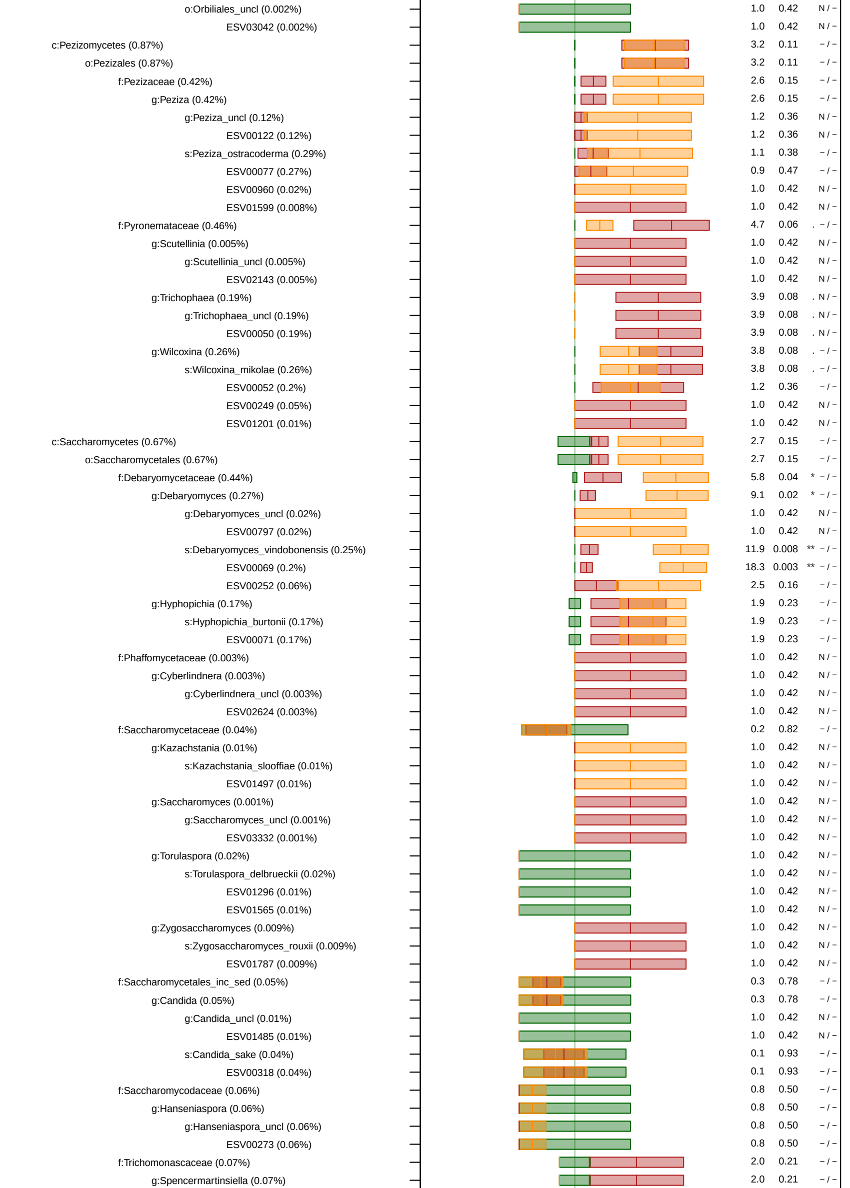
o:Orbiliales_uncl (0.002%) 1.0 0.42 N / –
ESV03042 (0.002%) 1.0 0.42 N / –
c:Pezizomycetes (0.87%) 3.2 0.11 – / –
o:Pezizales (0.87%) 3.2 0.11 – / –
f:Pezizaceae (0.42%) 2.6 0.15 – / –
g:Peziza (0.42%) 2.6 0.15 – / –
g:Peziza_uncl (0.12%) 1.2 0.36 N / –
ESV00122 (0.12%) 1.2 0.36 N / –
s:Peziza_ostracoderma (0.29%) 1.1 0.38 – / –
ESV00077 (0.27%) 0.9 0.47 – / –
ESV00960 (0.02%) 1.0 0.42 N / –
ESV01599 (0.008%) 1.0 0.42 N / –
f:Pyronemataceae (0.46%) 4.7 0.06 . – / –
g:Scutellinia (0.005%) 1.0 0.42 N / –
g:Scutellinia_uncl (0.005%) 1.0 0.42 N / –
ESV02143 (0.005%) 1.0 0.42 N / –
g:Trichophaea (0.19%) 3.9 0.08 . N / –
g:Trichophaea_uncl (0.19%) 3.9 0.08 . N / –
ESV00050 (0.19%) 3.9 0.08 . N / –
g:Wilcoxina (0.26%) 3.8 0.08 . – / –
s:Wilcoxina_mikolae (0.26%) 3.8 0.08 . – / –
ESV00052 (0.2%) 1.2 0.36 – / –
ESV00249 (0.05%) 1.0 0.42 N / –
ESV01201 (0.01%) 1.0 0.42 N / –
c:Saccharomycetes (0.67%) 2.7 0.15 – / –
o:Saccharomycetales (0.67%) 2.7 0.15 – / –
f:Debaryomycetaceae (0.44%) 5.8 0.04 * – / –
g:Debaryomyces (0.27%) 9.1 0.02 * – / –
g:Debaryomyces_uncl (0.02%) 1.0 0.42 N / –
ESV00797 (0.02%) 1.0 0.42 N / –
s:Debaryomyces_vindobonensis (0.25%) 11.9 0.008 ** – / –
ESV00069 (0.2%) 18.3 0.003 ** – / –
ESV00252 (0.06%) 2.5 0.16 – / –
g:Hyphopichia (0.17%) 1.9 0.23 – / –
s:Hyphopichia_burtonii (0.17%) 1.9 0.23 – / –
ESV00071 (0.17%) 1.9 0.23 – / –
f:Phaffomycetaceae (0.003%) 1.0 0.42 N / –
g:Cyberlindnera (0.003%) 1.0 0.42 N / –
g:Cyberlindnera_uncl (0.003%) 1.0 0.42 N / –
ESV02624 (0.003%) 1.0 0.42 N / –
f:Saccharomycetaceae (0.04%) 0.2 0.82 – / –
g:Kazachstania (0.01%) 1.0 0.42 N / –
s:Kazachstania_slooffiae (0.01%) 1.0 0.42 N / –
ESV01497 (0.01%) 1.0 0.42 N / –
g:Saccharomyces (0.001%) 1.0 0.42 N / –
g:Saccharomyces_uncl (0.001%) 1.0 0.42 N / –
ESV03332 (0.001%) 1.0 0.42 N / –
g:Torulaspora (0.02%) 1.0 0.42 N / –
s:Torulaspora_delbrueckii (0.02%) 1.0 0.42 N / –
ESV01296 (0.01%) 1.0 0.42 N / –
ESV01565 (0.01%) 1.0 0.42 N / –
g:Zygosaccharomyces (0.009%) 1.0 0.42 N / –
s:Zygosaccharomyces_rouxii (0.009%) 1.0 0.42 N / –
ESV01787 (0.009%) 1.0 0.42 N / –
f:Saccharomycetales_inc_sed (0.05%) 0.3 0.78 – / –
g:Candida (0.05%) 0.3 0.78 – / –
g:Candida_uncl (0.01%) 1.0 0.42 N / –
ESV01485 (0.01%) 1.0 0.42 N / –
s:Candida_sake (0.04%) 0.1 0.93 – / –
ESV00318 (0.04%) 0.1 0.93 – / –
f:Saccharomycodaceae (0.06%) 0.8 0.50 – / –
g:Hanseniaspora (0.06%) 0.8 0.50 – / –
g:Hanseniaspora_uncl (0.06%) 0.8 0.50 – / –
ESV00273 (0.06%) 0.8 0.50 – / –
f:Trichomonascaceae (0.07%) 2.0 0.21 – / –
g:Spencermartinsiella (0.07%) 2.0 0.21 – / –

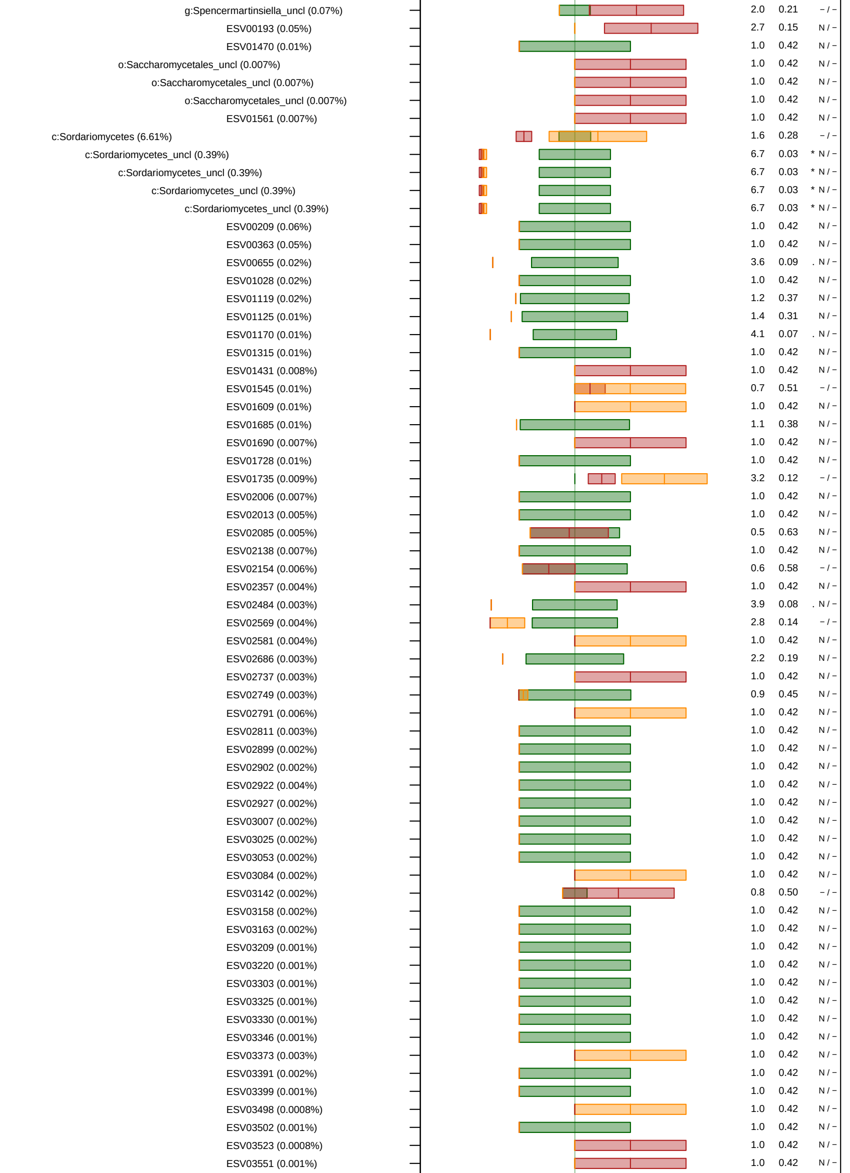
g:Spencermartinsiella_uncl (0.07%) 2.0 0.21 – / –
ESV00193 (0.05%) 2.7 0.15 N / –
ESV01470 (0.01%) 1.0 0.42 N / –
o:Saccharomycetales_uncl (0.007%) 1.0 0.42 N / –
o:Saccharomycetales_uncl (0.007%) 1.0 0.42 N / –
o:Saccharomycetales_uncl (0.007%) 1.0 0.42 N / –
ESV01561 (0.007%) 1.0 0.42 N / –
c:Sordariomycetes (6.61%) 1.6 0.28 – / –
c:Sordariomycetes_uncl (0.39%) 6.7 0.03 * N / –
c:Sordariomycetes_uncl (0.39%) 6.7 0.03 * N / –
c:Sordariomycetes_uncl (0.39%) 6.7 0.03 * N / –
c:Sordariomycetes_uncl (0.39%) 6.7 0.03 * N / –
ESV00209 (0.06%) 1.0 0.42 N / –
ESV00363 (0.05%) 1.0 0.42 N / –
ESV00655 (0.02%) 3.6 0.09 . N / –
ESV01028 (0.02%) 1.0 0.42 N / –
ESV01119 (0.02%) 1.2 0.37 N / –
ESV01125 (0.01%) 1.4 0.31 N / –
ESV01170 (0.01%) 4.1 0.07 . N / –
ESV01315 (0.01%) 1.0 0.42 N / –
ESV01431 (0.008%) 1.0 0.42 N / –
ESV01545 (0.01%) 0.7 0.51 – / –
ESV01609 (0.01%) 1.0 0.42 N / –
ESV01685 (0.01%) 1.1 0.38 N / –
ESV01690 (0.007%) 1.0 0.42 N / –
ESV01728 (0.01%) 1.0 0.42 N / –
ESV01735 (0.009%) 3.2 0.12 – / –
ESV02006 (0.007%) 1.0 0.42 N / –
ESV02013 (0.005%) 1.0 0.42 N / –
ESV02085 (0.005%) 0.5 0.63 N / –
ESV02138 (0.007%) 1.0 0.42 N / –
ESV02154 (0.006%) 0.6 0.58 – / –
ESV02357 (0.004%) 1.0 0.42 N / –
ESV02484 (0.003%) 3.9 0.08 . N / –
ESV02569 (0.004%) 2.8 0.14 – / –
ESV02581 (0.004%) 1.0 0.42 N / –
ESV02686 (0.003%) 2.2 0.19 N / –
ESV02737 (0.003%) 1.0 0.42 N / –
ESV02749 (0.003%) 0.9 0.45 N / –
ESV02791 (0.006%) 1.0 0.42 N / –
ESV02811 (0.003%) 1.0 0.42 N / –
ESV02899 (0.002%) 1.0 0.42 N / –
ESV02902 (0.002%) 1.0 0.42 N / –
ESV02922 (0.004%) 1.0 0.42 N / –
ESV02927 (0.002%) 1.0 0.42 N / –
ESV03007 (0.002%) 1.0 0.42 N / –
ESV03025 (0.002%) 1.0 0.42 N / –
ESV03053 (0.002%) 1.0 0.42 N / –
ESV03084 (0.002%) 1.0 0.42 N / –
ESV03142 (0.002%) 0.8 0.50 – / –
ESV03158 (0.002%) 1.0 0.42 N / –
ESV03163 (0.002%) 1.0 0.42 N / –
ESV03209 (0.001%) 1.0 0.42 N / –
ESV03220 (0.001%) 1.0 0.42 N / –
ESV03303 (0.001%) 1.0 0.42 N / –
ESV03325 (0.001%) 1.0 0.42 N / –
ESV03330 (0.001%) 1.0 0.42 N / –
ESV03346 (0.001%) 1.0 0.42 N / –
ESV03373 (0.003%) 1.0 0.42 N / –
ESV03391 (0.002%) 1.0 0.42 N / –
ESV03399 (0.001%) 1.0 0.42 N / –
ESV03498 (0.0008%) 1.0 0.42 N / –
ESV03502 (0.001%) 1.0 0.42 N / –
ESV03523 (0.0008%) 1.0 0.42 N / –
ESV03551 (0.001%) 1.0 0.42 N / –

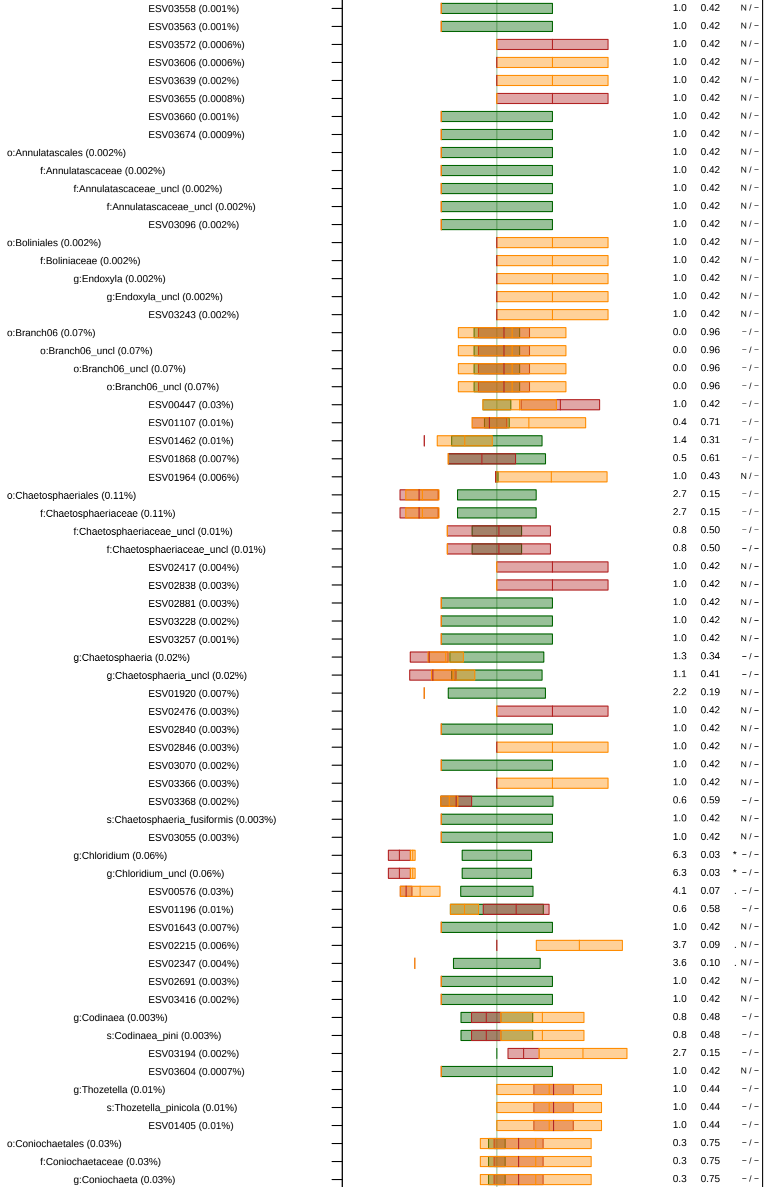
ESV03558 (0.001%) 1.0 0.42 N / –
ESV03563 (0.001%) 1.0 0.42 N / –
ESV03572 (0.0006%) 1.0 0.42 N / –
ESV03606 (0.0006%) 1.0 0.42 N / –
ESV03639 (0.002%) 1.0 0.42 N / –
ESV03655 (0.0008%) 1.0 0.42 N / –
ESV03660 (0.001%) 1.0 0.42 N / –
ESV03674 (0.0009%) 1.0 0.42 N / –
o:Annulatascales (0.002%) 1.0 0.42 N / –
f:Annulatascaceae (0.002%) 1.0 0.42 N / –
f:Annulatascaceae_uncl (0.002%) 1.0 0.42 N / –
f:Annulatascaceae_uncl (0.002%) 1.0 0.42 N / –
ESV03096 (0.002%) 1.0 0.42 N / –
o:Boliniales (0.002%) 1.0 0.42 N / –
f:Boliniaceae (0.002%) 1.0 0.42 N / –
g:Endoxyla (0.002%) 1.0 0.42 N / –
g:Endoxyla_uncl (0.002%) 1.0 0.42 N / –
ESV03243 (0.002%) 1.0 0.42 N / –
o:Branch06 (0.07%) 0.0 0.96 – / –
o:Branch06_uncl (0.07%) 0.0 0.96 – / –
o:Branch06_uncl (0.07%) 0.0 0.96 – / –
o:Branch06_uncl (0.07%) 0.0 0.96 – / –
ESV00447 (0.03%) 1.0 0.42 – / –
ESV01107 (0.01%) 0.4 0.71 – / –
ESV01462 (0.01%) 1.4 0.31 – / –
ESV01868 (0.007%) 0.5 0.61 – / –
ESV01964 (0.006%) 1.0 0.43 N / –
o:Chaetosphaeriales (0.11%) 2.7 0.15 – / –
f:Chaetosphaeriaceae (0.11%) 2.7 0.15 – / –
f:Chaetosphaeriaceae_uncl (0.01%) 0.8 0.50 – / –
f:Chaetosphaeriaceae_uncl (0.01%) 0.8 0.50 – / –
ESV02417 (0.004%) 1.0 0.42 N / –
ESV02838 (0.003%) 1.0 0.42 N / –
ESV02881 (0.003%) 1.0 0.42 N / –
ESV03228 (0.002%) 1.0 0.42 N / –
ESV03257 (0.001%) 1.0 0.42 N / –
g:Chaetosphaeria (0.02%) 1.3 0.34 – / –
g:Chaetosphaeria_uncl (0.02%) 1.1 0.41 – / –
ESV01920 (0.007%) 2.2 0.19 N / –
ESV02476 (0.003%) 1.0 0.42 N / –
ESV02840 (0.003%) 1.0 0.42 N / –
ESV02846 (0.003%) 1.0 0.42 N / –
ESV03070 (0.002%) 1.0 0.42 N / –
ESV03366 (0.003%) 1.0 0.42 N / –
ESV03368 (0.002%) 0.6 0.59 – / –
s:Chaetosphaeria_fusiformis (0.003%) 1.0 0.42 N / –
ESV03055 (0.003%) 1.0 0.42 N / –
g:Chloridium (0.06%) 6.3 0.03 * – / –
g:Chloridium_uncl (0.06%) 6.3 0.03 * – / –
ESV00576 (0.03%) 4.1 0.07 . – / –
ESV01196 (0.01%) 0.6 0.58 – / –
ESV01643 (0.007%) 1.0 0.42 N / –
ESV02215 (0.006%) 3.7 0.09 . N / –
ESV02347 (0.004%) 3.6 0.10 . N / –
ESV02691 (0.003%) 1.0 0.42 N / –
ESV03416 (0.002%) 1.0 0.42 N / –
g:Codinaea (0.003%) 0.8 0.48 – / –
s:Codinaea_pini (0.003%) 0.8 0.48 – / –
ESV03194 (0.002%) 2.7 0.15 – / –
ESV03604 (0.0007%) 1.0 0.42 N / –
g:Thozetella (0.01%) 1.0 0.44 – / –
s:Thozetella_pinicola (0.01%) 1.0 0.44 – / –
ESV01405 (0.01%) 1.0 0.44 – / –
o:Coniochaetales (0.03%) 0.3 0.75 – / –
f:Coniochaetaceae (0.03%) 0.3 0.75 – / –
g:Coniochaeta (0.03%) 0.3 0.75 – / –

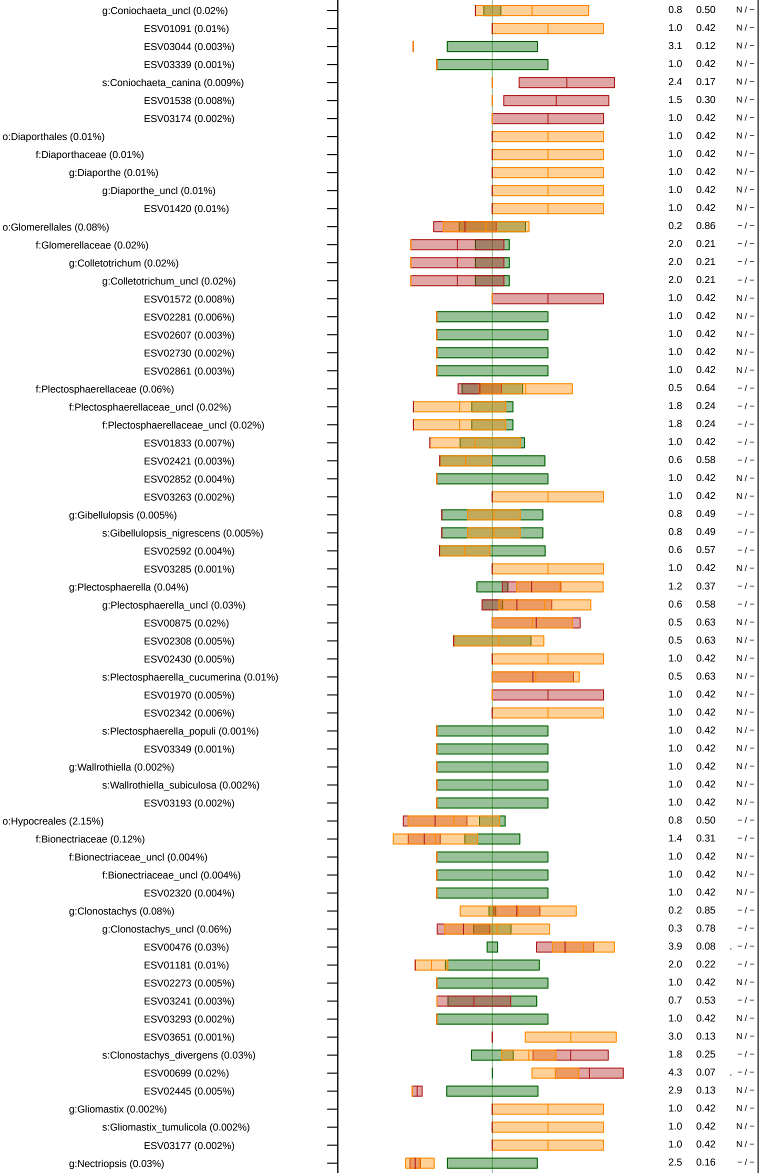
g:Coniochaeta_uncl (0.02%) 0.8 0.50 N / –
ESV01091 (0.01%) 1.0 0.42 N / –
ESV03044 (0.003%) 3.1 0.12 N / –
ESV03339 (0.001%) 1.0 0.42 N / –
s:Coniochaeta_canina (0.009%) 2.4 0.17 N / –
ESV01538 (0.008%) 1.5 0.30 N / –
ESV03174 (0.002%) 1.0 0.42 N / –
o:Diaporthales (0.01%) 1.0 0.42 N / –
f:Diaporthaceae (0.01%) 1.0 0.42 N / –
g:Diaporthe (0.01%) 1.0 0.42 N / –
g:Diaporthe_uncl (0.01%) 1.0 0.42 N / –
ESV01420 (0.01%) 1.0 0.42 N / –
o:Glomerellales (0.08%) 0.2 0.86 – / –
f:Glomerellaceae (0.02%) 2.0 0.21 – / –
g:Colletotrichum (0.02%) 2.0 0.21 – / –
g:Colletotrichum_uncl (0.02%) 2.0 0.21 – / –
ESV01572 (0.008%) 1.0 0.42 N / –
ESV02281 (0.006%) 1.0 0.42 N / –
ESV02607 (0.003%) 1.0 0.42 N / –
ESV02730 (0.002%) 1.0 0.42 N / –
ESV02861 (0.003%) 1.0 0.42 N / –
f:Plectosphaerellaceae (0.06%) 0.5 0.64 – / –
f:Plectosphaerellaceae_uncl (0.02%) 1.8 0.24 – / –
f:Plectosphaerellaceae_uncl (0.02%) 1.8 0.24 – / –
ESV01833 (0.007%) 1.0 0.42 – / –
ESV02421 (0.003%) 0.6 0.58 – / –
ESV02852 (0.004%) 1.0 0.42 N / –
ESV03263 (0.002%) 1.0 0.42 N / –
g:Gibellulopsis (0.005%) 0.8 0.49 – / –
s:Gibellulopsis_nigrescens (0.005%) 0.8 0.49 – / –
ESV02592 (0.004%) 0.6 0.57 – / –
ESV03285 (0.001%) 1.0 0.42 N / –
g:Plectosphaerella (0.04%) 1.2 0.37 – / –
g:Plectosphaerella_uncl (0.03%) 0.6 0.58 – / –
ESV00875 (0.02%) 0.5 0.63 N / –
ESV02308 (0.005%) 0.5 0.63 N / –
ESV02430 (0.005%) 1.0 0.42 N / –
s:Plectosphaerella_cucumerina (0.01%) 0.5 0.63 N / –
ESV01970 (0.005%) 1.0 0.42 N / –
ESV02342 (0.006%) 1.0 0.42 N / –
s:Plectosphaerella_populi (0.001%) 1.0 0.42 N / –
ESV03349 (0.001%) 1.0 0.42 N / –
g:Wallrothiella (0.002%) 1.0 0.42 N / –
s:Wallrothiella_subiculosa (0.002%) 1.0 0.42 N / –
ESV03193 (0.002%) 1.0 0.42 N / –
o:Hypocreales (2.15%) 0.8 0.50 – / –
f:Bionectriaceae (0.12%) 1.4 0.31 – / –
f:Bionectriaceae_uncl (0.004%) 1.0 0.42 N / –
f:Bionectriaceae_uncl (0.004%) 1.0 0.42 N / –
ESV02320 (0.004%) 1.0 0.42 N / –
g:Clonostachys (0.08%) 0.2 0.85 – / –
g:Clonostachys_uncl (0.06%) 0.3 0.78 – / –
ESV00476 (0.03%) 3.9 0.08 . – / –
ESV01181 (0.01%) 2.0 0.22 – / –
ESV02273 (0.005%) 1.0 0.42 N / –
ESV03241 (0.003%) 0.7 0.53 – / –
ESV03293 (0.002%) 1.0 0.42 N / –
ESV03651 (0.001%) 3.0 0.13 N / –
s:Clonostachys_divergens (0.03%) 1.8 0.25 – / –
ESV00699 (0.02%) 4.3 0.07 . – / –
ESV02445 (0.005%) 2.9 0.13 N / –
g:Gliomastix (0.002%) 1.0 0.42 N / –
s:Gliomastix_tumulicola (0.002%) 1.0 0.42 N / –
ESV03177 (0.002%) 1.0 0.42 N / –
g:Nectriopsis (0.03%) 2.5 0.16 – / –

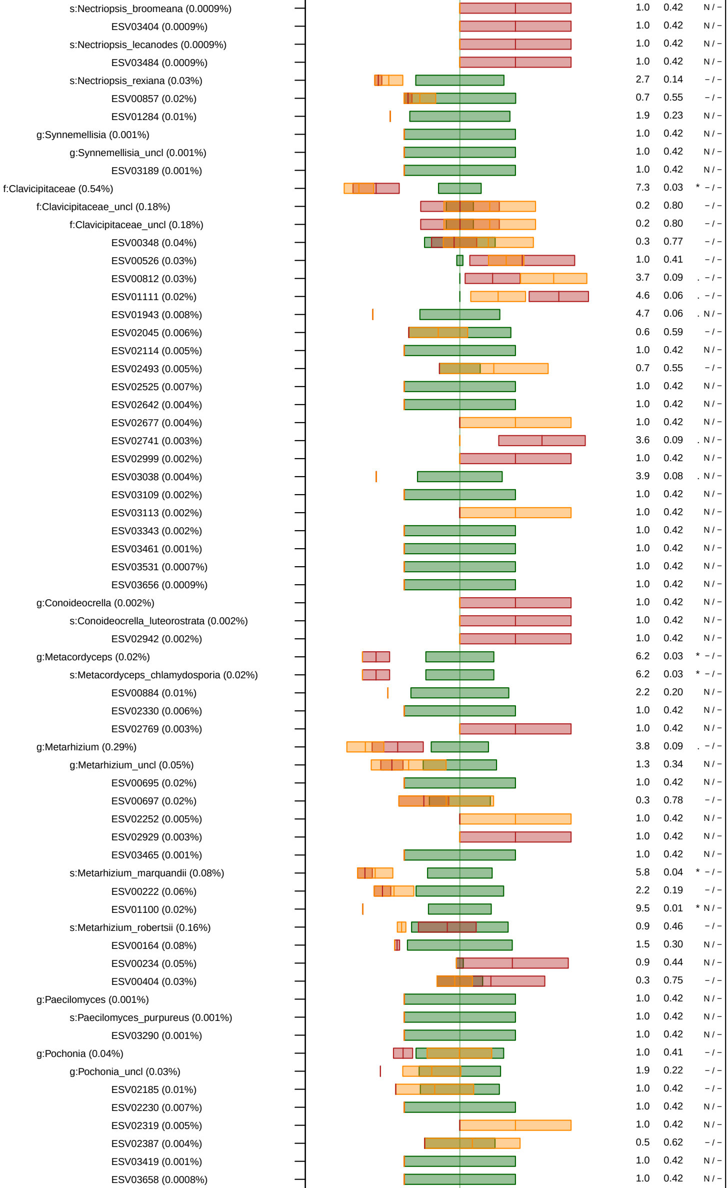
s:Nectriopsis_broomeana (0.0009%) 1.0 0.42 N / –
ESV03404 (0.0009%) 1.0 0.42 N / –
s:Nectriopsis_lecanodes (0.0009%) 1.0 0.42 N / –
ESV03484 (0.0009%) 1.0 0.42 N / –
s:Nectriopsis_rexiana (0.03%) 2.7 0.14 – / –
ESV00857 (0.02%) 0.7 0.55 – / –
ESV01284 (0.01%) 1.9 0.23 N / –
g:Synnemellisia (0.001%) 1.0 0.42 N / –
g:Synnemellisia_uncl (0.001%) 1.0 0.42 N / –
ESV03189 (0.001%) 1.0 0.42 N / –
f:Clavicipitaceae (0.54%) 7.3 0.03 * – / –
f:Clavicipitaceae_uncl (0.18%) 0.2 0.80 – / –
f:Clavicipitaceae_uncl (0.18%) 0.2 0.80 – / –
ESV00348 (0.04%) 0.3 0.77 – / –
ESV00526 (0.03%) 1.0 0.41 – / –
ESV00812 (0.03%) 3.7 0.09 . – / –
ESV01111 (0.02%) 4.6 0.06 . – / –
ESV01943 (0.008%) 4.7 0.06 . N / –
ESV02045 (0.006%) 0.6 0.59 – / –
ESV02114 (0.005%) 1.0 0.42 N / –
ESV02493 (0.005%) 0.7 0.55 – / –
ESV02525 (0.007%) 1.0 0.42 N / –
ESV02642 (0.004%) 1.0 0.42 N / –
ESV02677 (0.004%) 1.0 0.42 N / –
ESV02741 (0.003%) 3.6 0.09 . N / –
ESV02999 (0.002%) 1.0 0.42 N / –
ESV03038 (0.004%) 3.9 0.08 . N / –
ESV03109 (0.002%) 1.0 0.42 N / –
ESV03113 (0.002%) 1.0 0.42 N / –
ESV03343 (0.002%) 1.0 0.42 N / –
ESV03461 (0.001%) 1.0 0.42 N / –
ESV03531 (0.0007%) 1.0 0.42 N / –
ESV03656 (0.0009%) 1.0 0.42 N / –
g:Conoideocrella (0.002%) 1.0 0.42 N / –
s:Conoideocrella_luteorostrata (0.002%) 1.0 0.42 N / –
ESV02942 (0.002%) 1.0 0.42 N / –
g:Metacordyceps (0.02%) 6.2 0.03 * – / –
s:Metacordyceps_chlamydosporia (0.02%) 6.2 0.03 * – / –
ESV00884 (0.01%) 2.2 0.20 N / –
ESV02330 (0.006%) 1.0 0.42 N / –
ESV02769 (0.003%) 1.0 0.42 N / –
g:Metarhizium (0.29%) 3.8 0.09 . – / –
g:Metarhizium_uncl (0.05%) 1.3 0.34 N / –
ESV00695 (0.02%) 1.0 0.42 N / –
ESV00697 (0.02%) 0.3 0.78 – / –
ESV02252 (0.005%) 1.0 0.42 N / –
ESV02929 (0.003%) 1.0 0.42 N / –
ESV03465 (0.001%) 1.0 0.42 N / –
s:Metarhizium_marquandii (0.08%) 5.8 0.04 * – / –
ESV00222 (0.06%) 2.2 0.19 – / –
ESV01100 (0.02%) 9.5 0.01 * N / –
s:Metarhizium_robertsii (0.16%) 0.9 0.46 – / –
ESV00164 (0.08%) 1.5 0.30 N / –
ESV00234 (0.05%) 0.9 0.44 N / –
ESV00404 (0.03%) 0.3 0.75 – / –
g:Paecilomyces (0.001%) 1.0 0.42 N / –
s:Paecilomyces_purpureus (0.001%) 1.0 0.42 N / –
ESV03290 (0.001%) 1.0 0.42 N / –
g:Pochonia (0.04%) 1.0 0.41 – / –
g:Pochonia_uncl (0.03%) 1.9 0.22 – / –
ESV02185 (0.01%) 1.0 0.42 – / –
ESV02230 (0.007%) 1.0 0.42 N / –
ESV02319 (0.005%) 1.0 0.42 N / –
ESV02387 (0.004%) 0.5 0.62 – / –
ESV03419 (0.001%) 1.0 0.42 N / –
ESV03658 (0.0008%) 1.0 0.42 N / –

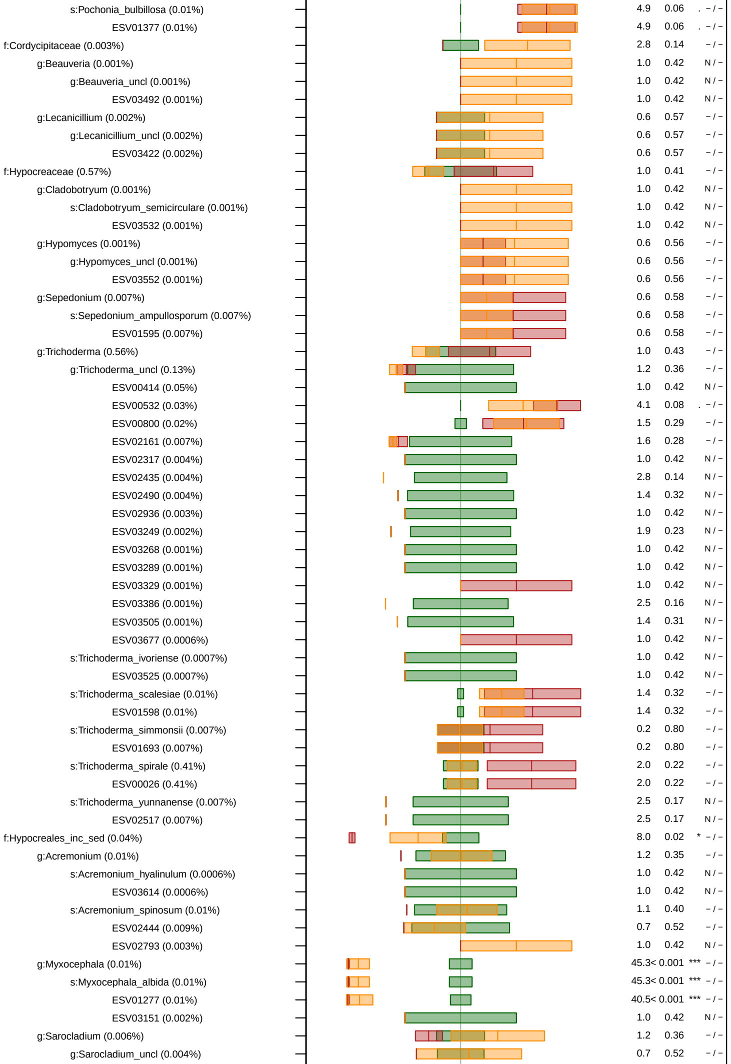
s:Pochonia_bulbillosa (0.01%) 4.9 0.06 . – / –
ESV01377 (0.01%) 4.9 0.06 . – / –
f:Cordycipitaceae (0.003%) 2.8 0.14 – / –
g:Beauveria (0.001%) 1.0 0.42 N / –
g:Beauveria_uncl (0.001%) 1.0 0.42 N / –
ESV03492 (0.001%) 1.0 0.42 N / –
g:Lecanicillium (0.002%) 0.6 0.57 – / –
g:Lecanicillium_uncl (0.002%) 0.6 0.57 – / –
ESV03422 (0.002%) 0.6 0.57 – / –
f:Hypocreaceae (0.57%) 1.0 0.41 – / –
g:Cladobotryum (0.001%) 1.0 0.42 N / –
s:Cladobotryum_semicirculare (0.001%) 1.0 0.42 N / –
ESV03532 (0.001%) 1.0 0.42 N / –
g:Hypomyces (0.001%) 0.6 0.56 – / –
g:Hypomyces_uncl (0.001%) 0.6 0.56 – / –
ESV03552 (0.001%) 0.6 0.56 – / –
g:Sepedonium (0.007%) 0.6 0.58 – / –
s:Sepedonium_ampullosporum (0.007%) 0.6 0.58 – / –
ESV01595 (0.007%) 0.6 0.58 – / –
g:Trichoderma (0.56%) 1.0 0.43 – / –
g:Trichoderma_uncl (0.13%) 1.2 0.36 – / –
ESV00414 (0.05%) 1.0 0.42 N / –
ESV00532 (0.03%) 4.1 0.08 . – / –
ESV00800 (0.02%) 1.5 0.29 – / –
ESV02161 (0.007%) 1.6 0.28 – / –
ESV02317 (0.004%) 1.0 0.42 N / –
ESV02435 (0.004%) 2.8 0.14 N / –
ESV02490 (0.004%) 1.4 0.32 N / –
ESV02936 (0.003%) 1.0 0.42 N / –
ESV03249 (0.002%) 1.9 0.23 N / –
ESV03268 (0.001%) 1.0 0.42 N / –
ESV03289 (0.001%) 1.0 0.42 N / –
ESV03329 (0.001%) 1.0 0.42 N / –
ESV03386 (0.001%) 2.5 0.16 N / –
ESV03505 (0.001%) 1.4 0.31 N / –
ESV03677 (0.0006%) 1.0 0.42 N / –
s:Trichoderma_ivoriense (0.0007%) 1.0 0.42 N / –
ESV03525 (0.0007%) 1.0 0.42 N / –
s:Trichoderma_scalesiae (0.01%) 1.4 0.32 – / –
ESV01598 (0.01%) 1.4 0.32 – / –
s:Trichoderma_simmonsii (0.007%) 0.2 0.80 – / –
ESV01693 (0.007%) 0.2 0.80 – / –
s:Trichoderma_spirale (0.41%) 2.0 0.22 – / –
ESV00026 (0.41%) 2.0 0.22 – / –
s:Trichoderma_yunnanense (0.007%) 2.5 0.17 N / –
ESV02517 (0.007%) 2.5 0.17 N / –
f:Hypocreales_inc_sed (0.04%) 8.0 0.02 * – / –
g:Acremonium (0.01%) 1.2 0.35 – / –
s:Acremonium_hyalinulum (0.0006%) 1.0 0.42 N / –
ESV03614 (0.0006%) 1.0 0.42 N / –
s:Acremonium_spinosum (0.01%) 1.1 0.40 – / –
ESV02444 (0.009%) 0.7 0.52 – / –
ESV02793 (0.003%) 1.0 0.42 N / –
g:Myxocephala (0.01%) 45.3 < 0.001 *** – / –
s:Myxocephala_albida (0.01%) 45.3 < 0.001 *** – / –
ESV01277 (0.01%) 40.5 < 0.001 *** – / –
ESV03151 (0.002%) 1.0 0.42 N / –
g:Sarocladium (0.006%) 1.2 0.36 – / –
g:Sarocladium_uncl (0.004%) 0.7 0.52 – / –

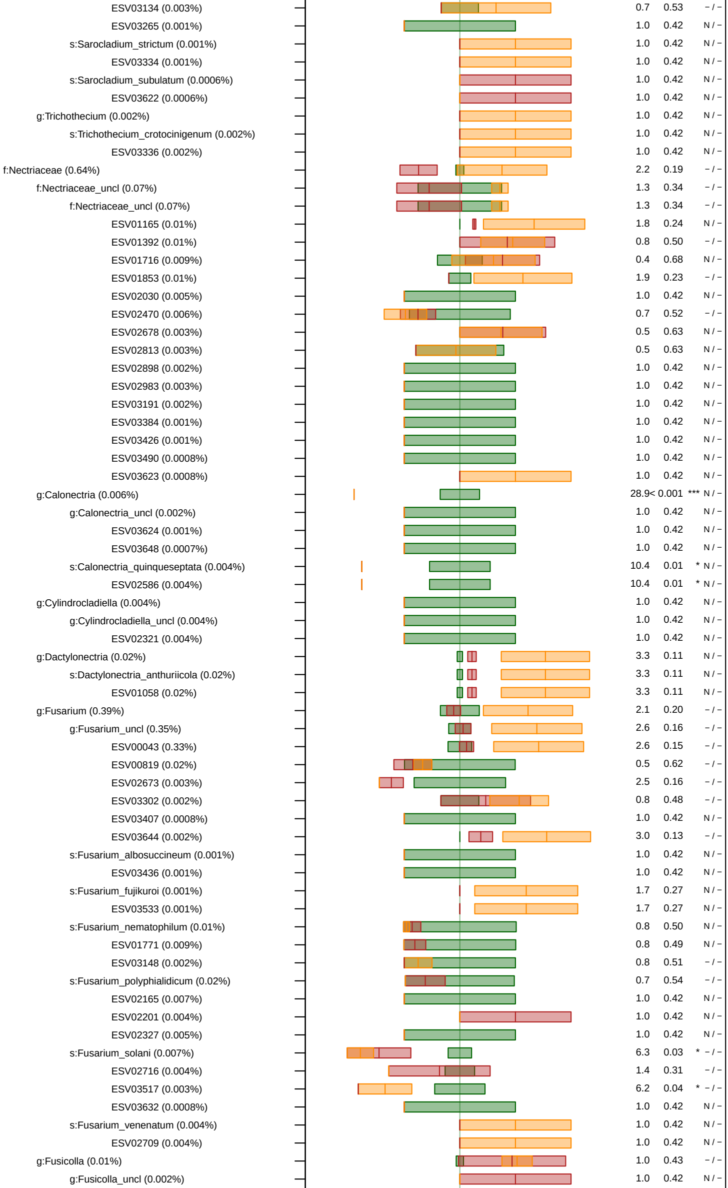

ESV03134 (0.003%) 0.7 0.53 – / –
ESV03265 (0.001%) 1.0 0.42 N / –
s:Sarocladium_strictum (0.001%) 1.0 0.42 N / –
ESV03334 (0.001%) 1.0 0.42 N / –
s:Sarocladium_subulatum (0.0006%) 1.0 0.42 N / –
ESV03622 (0.0006%) 1.0 0.42 N / –
g:Trichothecium (0.002%) 1.0 0.42 N / –
s:Trichothecium_crotocinigenum (0.002%) 1.0 0.42 N / –
ESV03336 (0.002%) 1.0 0.42 N / –
f:Nectriaceae (0.64%) 2.2 0.19 – / –
f:Nectriaceae_uncl (0.07%) 1.3 0.34 – / –
f:Nectriaceae_uncl (0.07%) 1.3 0.34 – / –
ESV01165 (0.01%) 1.8 0.24 N / –
ESV01392 (0.01%) 0.8 0.50 – / –
ESV01716 (0.009%) 0.4 0.68 N / –
ESV01853 (0.01%) 1.9 0.23 – / –
ESV02030 (0.005%) 1.0 0.42 N / –
ESV02470 (0.006%) 0.7 0.52 – / –
ESV02678 (0.003%) 0.5 0.63 N / –
ESV02813 (0.003%) 0.5 0.63 N / –
ESV02898 (0.002%) 1.0 0.42 N / –
ESV02983 (0.003%) 1.0 0.42 N / –
ESV03191 (0.002%) 1.0 0.42 N / –
ESV03384 (0.001%) 1.0 0.42 N / –
ESV03426 (0.001%) 1.0 0.42 N / –
ESV03490 (0.0008%) 1.0 0.42 N / –
ESV03623 (0.0008%) 1.0 0.42 N / –
g:Calonectria (0.006%) 28.9 < 0.001 *** N / –
g:Calonectria_uncl (0.002%) 1.0 0.42 N / –
ESV03624 (0.001%) 1.0 0.42 N / –
ESV03648 (0.0007%) 1.0 0.42 N / –
s:Calonectria_quinqueseptata (0.004%) 10.4 0.01 * N / –
ESV02586 (0.004%) 10.4 0.01 * N / –
g:Cylindrocladiella (0.004%) 1.0 0.42 N / –
g:Cylindrocladiella_uncl (0.004%) 1.0 0.42 N / –
ESV02321 (0.004%) 1.0 0.42 N / –
g:Dactylonectria (0.02%) 3.3 0.11 N / –
s:Dactylonectria_anthuriicola (0.02%) 3.3 0.11 N / –
ESV01058 (0.02%) 3.3 0.11 N / –
g:Fusarium (0.39%) 2.1 0.20 – / –
g:Fusarium_uncl (0.35%) 2.6 0.16 – / –
ESV00043 (0.33%) 2.6 0.15 – / –
ESV00819 (0.02%) 0.5 0.62 – / –
ESV02673 (0.003%) 2.5 0.16 – / –
ESV03302 (0.002%) 0.8 0.48 – / –
ESV03407 (0.0008%) 1.0 0.42 N / –
ESV03644 (0.002%) 3.0 0.13 – / –
s:Fusarium_albosuccineum (0.001%) 1.0 0.42 N / –
ESV03436 (0.001%) 1.0 0.42 N / –
s:Fusarium_fujikuroi (0.001%) 1.7 0.27 N / –
ESV03533 (0.001%) 1.7 0.27 N / –
s:Fusarium_nematophilum (0.01%) 0.8 0.50 N / –
ESV01771 (0.009%) 0.8 0.49 N / –
ESV03148 (0.002%) 0.8 0.51 – / –
s:Fusarium_polyphialidicum (0.02%) 0.7 0.54 – / –
ESV02165 (0.007%) 1.0 0.42 N / –
ESV02201 (0.004%) 1.0 0.42 N / –
ESV02327 (0.005%) 1.0 0.42 N / –
s:Fusarium_solani (0.007%) 6.3 0.03 * – / –
ESV02716 (0.004%) 1.4 0.31 – / –
ESV03517 (0.003%) 6.2 0.04 * – / –
ESV03632 (0.0008%) 1.0 0.42 N / –
s:Fusarium_venenatum (0.004%) 1.0 0.42 N / –
ESV02709 (0.004%) 1.0 0.42 N / –
g:Fusicolla (0.01%) 1.0 0.43 – / –
g:Fusicolla_uncl (0.002%) 1.0 0.42 N / –

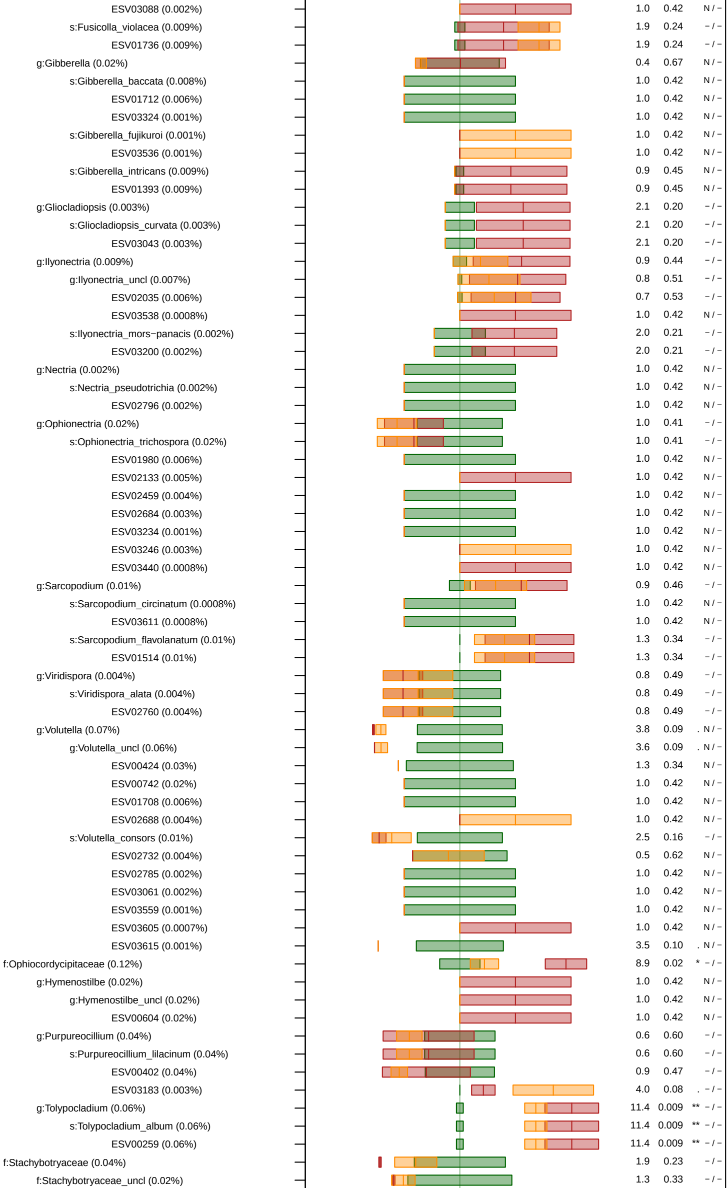

ESV03088 (0.002%) 1.0 0.42 N / –
s:Fusicolla_violacea (0.009%) 1.9 0.24 – / –
ESV01736 (0.009%) 1.9 0.24 – / –
g:Gibberella (0.02%) 0.4 0.67 N / –
s:Gibberella_baccata (0.008%) 1.0 0.42 N / –
ESV01712 (0.006%) 1.0 0.42 N / –
ESV03324 (0.001%) 1.0 0.42 N / –
s:Gibberella_fujikuroi (0.001%) 1.0 0.42 N / –
ESV03536 (0.001%) 1.0 0.42 N / –
s:Gibberella_intricans (0.009%) 0.9 0.45 N / –
ESV01393 (0.009%) 0.9 0.45 N / –
g:Gliocladiopsis (0.003%) 2.1 0.20 – / –
s:Gliocladiopsis_curvata (0.003%) 2.1 0.20 – / –
ESV03043 (0.003%) 2.1 0.20 – / –
g:Ilyonectria (0.009%) 0.9 0.44 – / –
g:Ilyonectria_uncl (0.007%) 0.8 0.51 – / –
ESV02035 (0.006%) 0.7 0.53 – / –
ESV03538 (0.0008%) 1.0 0.42 N / –
s:Ilyonectria_mors−panacis (0.002%) 2.0 0.21 – / –
ESV03200 (0.002%) 2.0 0.21 – / –
g:Nectria (0.002%) 1.0 0.42 N / –
s:Nectria_pseudotrichia (0.002%) 1.0 0.42 N / –
ESV02796 (0.002%) 1.0 0.42 N / –
g:Ophionectria (0.02%) 1.0 0.41 – / –
s:Ophionectria_trichospora (0.02%) 1.0 0.41 – / –
ESV01980 (0.006%) 1.0 0.42 N / –
ESV02133 (0.005%) 1.0 0.42 N / –
ESV02459 (0.004%) 1.0 0.42 N / –
ESV02684 (0.003%) 1.0 0.42 N / –
ESV03234 (0.001%) 1.0 0.42 N / –
ESV03246 (0.003%) 1.0 0.42 N / –
ESV03440 (0.0008%) 1.0 0.42 N / –
g:Sarcopodium (0.01%) 0.9 0.46 – / –
s:Sarcopodium_circinatum (0.0008%) 1.0 0.42 N / –
ESV03611 (0.0008%) 1.0 0.42 N / –
s:Sarcopodium_flavolanatum (0.01%) 1.3 0.34 – / –
ESV01514 (0.01%) 1.3 0.34 – / –
g:Viridispora (0.004%) 0.8 0.49 – / –
s:Viridispora_alata (0.004%) 0.8 0.49 – / –
ESV02760 (0.004%) 0.8 0.49 – / –
g:Volutella (0.07%) 3.8 0.09 . N / –
g:Volutella_uncl (0.06%) 3.6 0.09 . N / –
ESV00424 (0.03%) 1.3 0.34 N / –
ESV00742 (0.02%) 1.0 0.42 N / –
ESV01708 (0.006%) 1.0 0.42 N / –
ESV02688 (0.004%) 1.0 0.42 N / –
s:Volutella_consors (0.01%) 2.5 0.16 – / –
ESV02732 (0.004%) 0.5 0.62 N / –
ESV02785 (0.002%) 1.0 0.42 N / –
ESV03061 (0.002%) 1.0 0.42 N / –
ESV03559 (0.001%) 1.0 0.42 N / –
ESV03605 (0.0007%) 1.0 0.42 N / –
ESV03615 (0.001%) 3.5 0.10 . N / –
f:Ophiocordycipitaceae (0.12%) 8.9 0.02 * – / –
g:Hymenostilbe (0.02%) 1.0 0.42 N / –
g:Hymenostilbe_uncl (0.02%) 1.0 0.42 N / –
ESV00604 (0.02%) 1.0 0.42 N / –
g:Purpureocillium (0.04%) 0.6 0.60 – / –
s:Purpureocillium_lilacinum (0.04%) 0.6 0.60 – / –
ESV00402 (0.04%) 0.9 0.47 – / –
ESV03183 (0.003%) 4.0 0.08 . – / –
g:Tolypocladium (0.06%) 11.4 0.009 ** – / –
s:Tolypocladium_album (0.06%) 11.4 0.009 ** – / –
ESV00259 (0.06%) 11.4 0.009 ** – / –
f:Stachybotryaceae (0.04%) 1.9 0.23 – / –
f:Stachybotryaceae_uncl (0.02%) 1.3 0.33 – / –

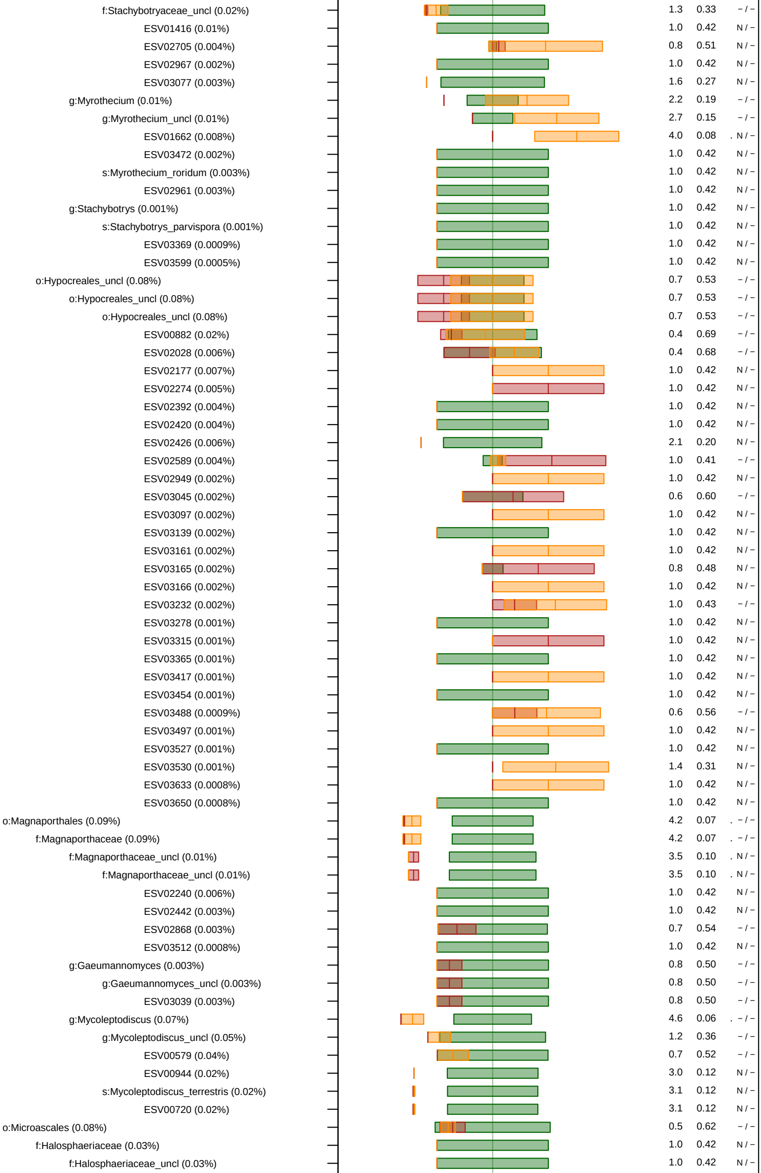

f:Stachybotryaceae_uncl (0.02%) 1.3 0.33 – / –
ESV01416 (0.01%) 1.0 0.42 N / –
ESV02705 (0.004%) 0.8 0.51 N / –
ESV02967 (0.002%) 1.0 0.42 N / –
ESV03077 (0.003%) 1.6 0.27 N / –
g:Myrothecium (0.01%) 2.2 0.19 – / –
g:Myrothecium_uncl (0.01%) 2.7 0.15 – / –
ESV01662 (0.008%) 4.0 0.08 . N / –
ESV03472 (0.002%) 1.0 0.42 N / –
s:Myrothecium_roridum (0.003%) 1.0 0.42 N / –
ESV02961 (0.003%) 1.0 0.42 N / –
g:Stachybotrys (0.001%) 1.0 0.42 N / –
s:Stachybotrys_parvispora (0.001%) 1.0 0.42 N / –
ESV03369 (0.0009%) 1.0 0.42 N / –
ESV03599 (0.0005%) 1.0 0.42 N / –
o:Hypocreales_uncl (0.08%) 0.7 0.53 – / –
o:Hypocreales_uncl (0.08%) 0.7 0.53 – / –
o:Hypocreales_uncl (0.08%) 0.7 0.53 – / –
ESV00882 (0.02%) 0.4 0.69 – / –
ESV02028 (0.006%) 0.4 0.68 – / –
ESV02177 (0.007%) 1.0 0.42 N / –
ESV02274 (0.005%) 1.0 0.42 N / –
ESV02392 (0.004%) 1.0 0.42 N / –
ESV02420 (0.004%) 1.0 0.42 N / –
ESV02426 (0.006%) 2.1 0.20 N / –
ESV02589 (0.004%) 1.0 0.41 – / –
ESV02949 (0.002%) 1.0 0.42 N / –
ESV03045 (0.002%) 0.6 0.60 – / –
ESV03097 (0.002%) 1.0 0.42 N / –
ESV03139 (0.002%) 1.0 0.42 N / –
ESV03161 (0.002%) 1.0 0.42 N / –
ESV03165 (0.002%) 0.8 0.48 N / –
ESV03166 (0.002%) 1.0 0.42 N / –
ESV03232 (0.002%) 1.0 0.43 – / –
ESV03278 (0.001%) 1.0 0.42 N / –
ESV03315 (0.001%) 1.0 0.42 N / –
ESV03365 (0.001%) 1.0 0.42 N / –
ESV03417 (0.001%) 1.0 0.42 N / –
ESV03454 (0.001%) 1.0 0.42 N / –
ESV03488 (0.0009%) 0.6 0.56 – / –
ESV03497 (0.001%) 1.0 0.42 N / –
ESV03527 (0.001%) 1.0 0.42 N / –
ESV03530 (0.001%) 1.4 0.31 N / –
ESV03633 (0.0008%) 1.0 0.42 N / –
ESV03650 (0.0008%) 1.0 0.42 N / –
o:Magnaporthales (0.09%) 4.2 0.07 . – / –
f:Magnaporthaceae (0.09%) 4.2 0.07 . – / –
f:Magnaporthaceae_uncl (0.01%) 3.5 0.10 . N / –
f:Magnaporthaceae_uncl (0.01%) 3.5 0.10 . N / –
ESV02240 (0.006%) 1.0 0.42 N / –
ESV02442 (0.003%) 1.0 0.42 N / –
ESV02868 (0.003%) 0.7 0.54 – / –
ESV03512 (0.0008%) 1.0 0.42 N / –
g:Gaeumannomyces (0.003%) 0.8 0.50 – / –
g:Gaeumannomyces_uncl (0.003%) 0.8 0.50 – / –
ESV03039 (0.003%) 0.8 0.50 – / –
g:Mycoleptodiscus (0.07%) 4.6 0.06 . – / –
g:Mycoleptodiscus_uncl (0.05%) 1.2 0.36 – / –
ESV00579 (0.04%) 0.7 0.52 – / –
ESV00944 (0.02%) 3.0 0.12 N / –
s:Mycoleptodiscus_terrestris (0.02%) 3.1 0.12 N / –
ESV00720 (0.02%) 3.1 0.12 N / –
o:Microascales (0.08%) 0.5 0.62 – / –
f:Halosphaeriaceae (0.03%) 1.0 0.42 N / –
f:Halosphaeriaceae_uncl (0.03%) 1.0 0.42 N / –

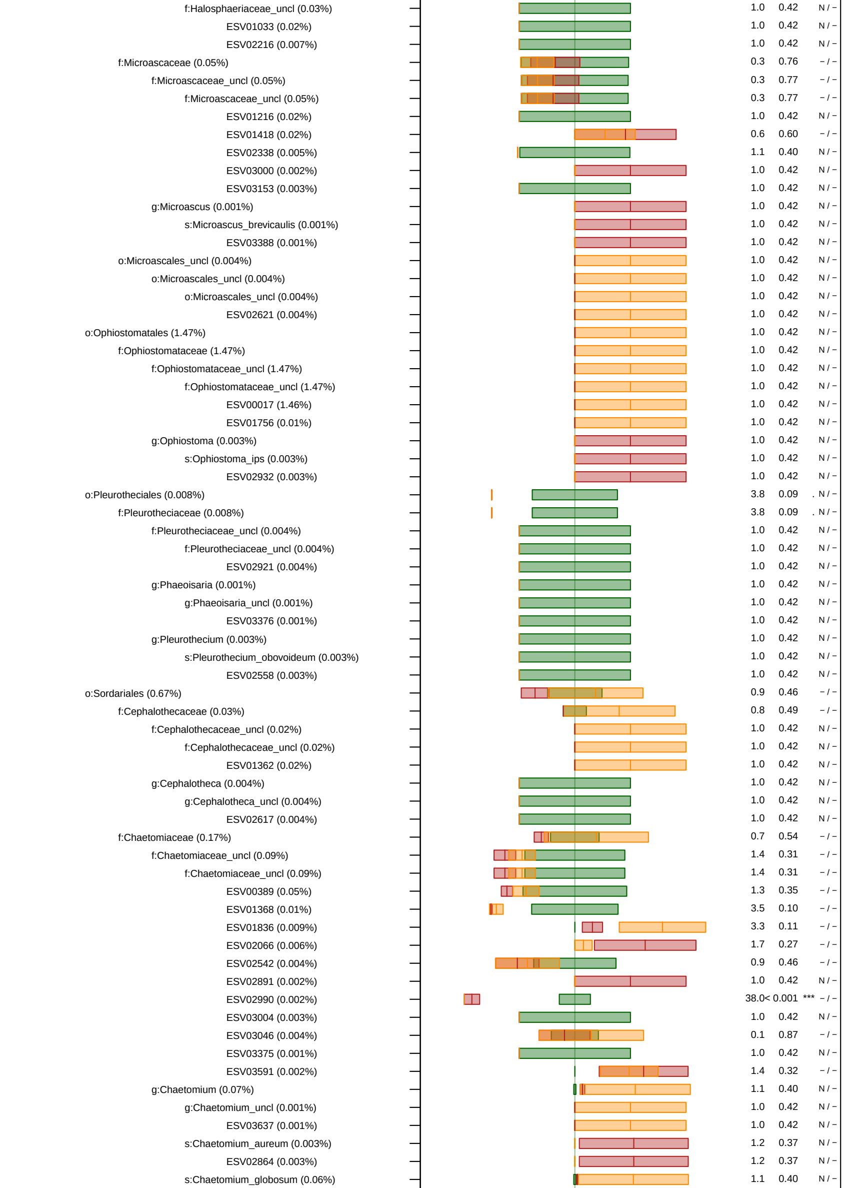
f:Halosphaeriaceae_uncl (0.03%) 1.0 0.42 N / –
ESV01033 (0.02%) 1.0 0.42 N / –
ESV02216 (0.007%) 1.0 0.42 N / –
f:Microascaceae (0.05%) 0.3 0.76 – / –
f:Microascaceae_uncl (0.05%) 0.3 0.77 – / –
f:Microascaceae_uncl (0.05%) 0.3 0.77 – / –
ESV01216 (0.02%) 1.0 0.42 N / –
ESV01418 (0.02%) 0.6 0.60 – / –
ESV02338 (0.005%) 1.1 0.40 N / –
ESV03000 (0.002%) 1.0 0.42 N / –
ESV03153 (0.003%) 1.0 0.42 N / –
g:Microascus (0.001%) 1.0 0.42 N / –
s:Microascus_brevicaulis (0.001%) 1.0 0.42 N / –
ESV03388 (0.001%) 1.0 0.42 N / –
o:Microascales_uncl (0.004%) 1.0 0.42 N / –
o:Microascales_uncl (0.004%) 1.0 0.42 N / –
o:Microascales_uncl (0.004%) 1.0 0.42 N / –
ESV02621 (0.004%) 1.0 0.42 N / –
o:Ophiostomatales (1.47%) 1.0 0.42 N / –
f:Ophiostomataceae (1.47%) 1.0 0.42 N / –
f:Ophiostomataceae_uncl (1.47%) 1.0 0.42 N / –
f:Ophiostomataceae_uncl (1.47%) 1.0 0.42 N / –
ESV00017 (1.46%) 1.0 0.42 N / –
ESV01756 (0.01%) 1.0 0.42 N / –
g:Ophiostoma (0.003%) 1.0 0.42 N / –
s:Ophiostoma_ips (0.003%) 1.0 0.42 N / –
ESV02932 (0.003%) 1.0 0.42 N / –
o:Pleurotheciales (0.008%) 3.8 0.09 . N / –
f:Pleurotheciaceae (0.008%) 3.8 0.09 . N / –
f:Pleurotheciaceae_uncl (0.004%) 1.0 0.42 N / –
f:Pleurotheciaceae_uncl (0.004%) 1.0 0.42 N / –
ESV02921 (0.004%) 1.0 0.42 N / –
g:Phaeoisaria (0.001%) 1.0 0.42 N / –
g:Phaeoisaria_uncl (0.001%) 1.0 0.42 N / –
ESV03376 (0.001%) 1.0 0.42 N / –
g:Pleurothecium (0.003%) 1.0 0.42 N / –
s:Pleurothecium_obovoideum (0.003%) 1.0 0.42 N / –
ESV02558 (0.003%) 1.0 0.42 N / –
o:Sordariales (0.67%) 0.9 0.46 – / –
f:Cephalothecaceae (0.03%) 0.8 0.49 – / –
f:Cephalothecaceae_uncl (0.02%) 1.0 0.42 N / –
f:Cephalothecaceae_uncl (0.02%) 1.0 0.42 N / –
ESV01362 (0.02%) 1.0 0.42 N / –
g:Cephalotheca (0.004%) 1.0 0.42 N / –
g:Cephalotheca_uncl (0.004%) 1.0 0.42 N / –
ESV02617 (0.004%) 1.0 0.42 N / –
f:Chaetomiaceae (0.17%) 0.7 0.54 – / –
f:Chaetomiaceae_uncl (0.09%) 1.4 0.31 – / –
f:Chaetomiaceae_uncl (0.09%) 1.4 0.31 – / –
ESV00389 (0.05%) 1.3 0.35 – / –
ESV01368 (0.01%) 3.5 0.10 – / –
ESV01836 (0.009%) 3.3 0.11 – / –
ESV02066 (0.006%) 1.7 0.27 – / –
ESV02542 (0.004%) 0.9 0.46 – / –
ESV02891 (0.002%) 1.0 0.42 N / –
ESV02990 (0.002%) 38.0 < 0.001 *** – / –
ESV03004 (0.003%) 1.0 0.42 N / –
ESV03046 (0.004%) 0.1 0.87 – / –
ESV03375 (0.001%) 1.0 0.42 N / –
ESV03591 (0.002%) 1.4 0.32 – / –
g:Chaetomium (0.07%) 1.1 0.40 N / –
g:Chaetomium_uncl (0.001%) 1.0 0.42 N / –
ESV03637 (0.001%) 1.0 0.42 N / –
s:Chaetomium_aureum (0.003%) 1.2 0.37 N / –
ESV02864 (0.003%) 1.2 0.37 N / –
s:Chaetomium_globosum (0.06%) 1.1 0.40 N / –

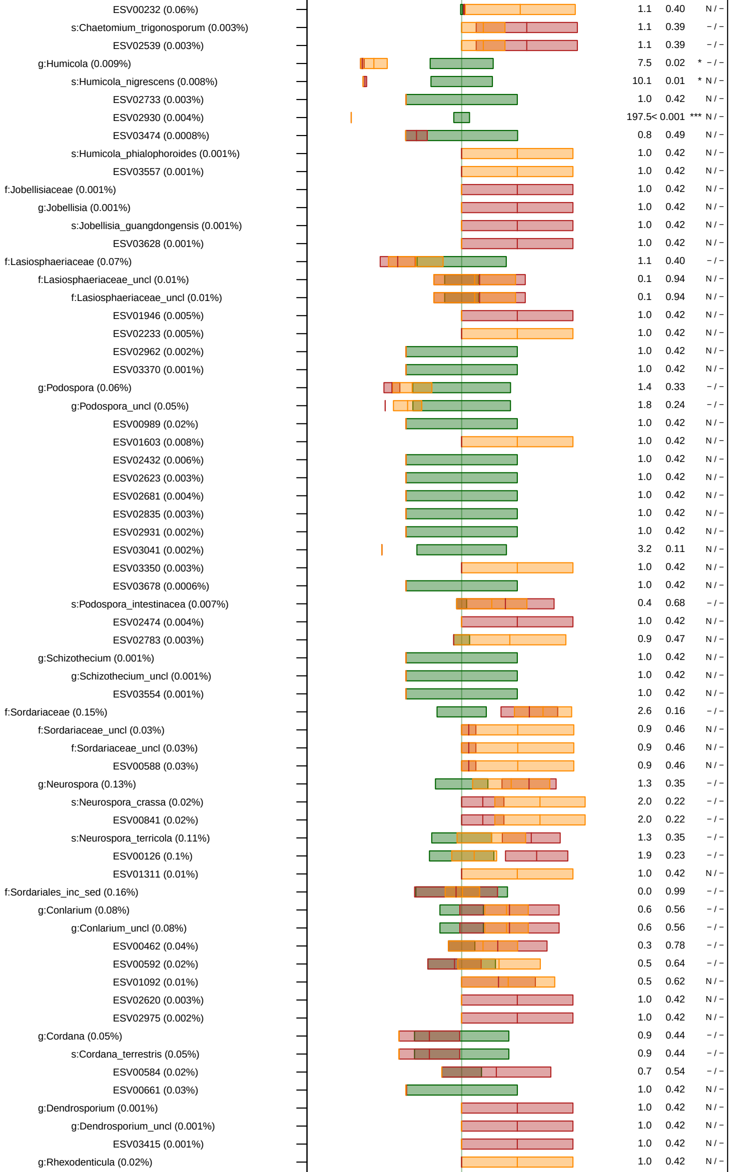
ESV00232 (0.06%) 1.1 0.40 N / –
s:Chaetomium_trigonosporum (0.003%) 1.1 0.39 – / –
ESV02539 (0.003%) 1.1 0.39 – / –
g:Humicola (0.009%) 7.5 0.02 * – / –
s:Humicola_nigrescens (0.008%) 10.1 0.01 * N / –
ESV02733 (0.003%) 1.0 0.42 N / –
ESV02930 (0.004%) 197.5< 0.001 *** N / –
ESV03474 (0.0008%) 0.8 0.49 N / –
s:Humicola_phialophoroides (0.001%) 1.0 0.42 N / –
ESV03557 (0.001%) 1.0 0.42 N / –
f:Jobellisiaceae (0.001%) 1.0 0.42 N / –
g:Jobellisia (0.001%) 1.0 0.42 N / –
s:Jobellisia_guangdongensis (0.001%) 1.0 0.42 N / –
ESV03628 (0.001%) 1.0 0.42 N / –
f:Lasiosphaeriaceae (0.07%) 1.1 0.40 – / –
f:Lasiosphaeriaceae_uncl (0.01%) 0.1 0.94 N / –
f:Lasiosphaeriaceae_uncl (0.01%) 0.1 0.94 N / –
ESV01946 (0.005%) 1.0 0.42 N / –
ESV02233 (0.005%) 1.0 0.42 N / –
ESV02962 (0.002%) 1.0 0.42 N / –
ESV03370 (0.001%) 1.0 0.42 N / –
g:Podospora (0.06%) 1.4 0.33 – / –
g:Podospora_uncl (0.05%) 1.8 0.24 – / –
ESV00989 (0.02%) 1.0 0.42 N / –
ESV01603 (0.008%) 1.0 0.42 N / –
ESV02432 (0.006%) 1.0 0.42 N / –
ESV02623 (0.003%) 1.0 0.42 N / –
ESV02681 (0.004%) 1.0 0.42 N / –
ESV02835 (0.003%) 1.0 0.42 N / –
ESV02931 (0.002%) 1.0 0.42 N / –
ESV03041 (0.002%) 3.2 0.11 N / –
ESV03350 (0.003%) 1.0 0.42 N / –
ESV03678 (0.0006%) 1.0 0.42 N / –
s:Podospora_intestinacea (0.007%) 0.4 0.68 – / –
ESV02474 (0.004%) 1.0 0.42 N / –
ESV02783 (0.003%) 0.9 0.47 N / –
g:Schizothecium (0.001%) 1.0 0.42 N / –
g:Schizothecium_uncl (0.001%) 1.0 0.42 N / –
ESV03554 (0.001%) 1.0 0.42 N / –
f:Sordariaceae (0.15%) 2.6 0.16 – / –
f:Sordariaceae_uncl (0.03%) 0.9 0.46 N / –
f:Sordariaceae_uncl (0.03%) 0.9 0.46 N / –
ESV00588 (0.03%) 0.9 0.46 N / –
g:Neurospora (0.13%) 1.3 0.35 – / –
s:Neurospora_crassa (0.02%) 2.0 0.22 – / –
ESV00841 (0.02%) 2.0 0.22 – / –
s:Neurospora_terricola (0.11%) 1.3 0.35 – / –
ESV00126 (0.1%) 1.9 0.23 – / –
ESV01311 (0.01%) 1.0 0.42 N / –
f:Sordariales_inc_sed (0.16%) 0.0 0.99 – / –
g:Conlarium (0.08%) 0.6 0.56 – / –
g:Conlarium_uncl (0.08%) 0.6 0.56 – / –
ESV00462 (0.04%) 0.3 0.78 – / –
ESV00592 (0.02%) 0.5 0.64 – / –
ESV01092 (0.01%) 0.5 0.62 N / –
ESV02620 (0.003%) 1.0 0.42 N / –
ESV02975 (0.002%) 1.0 0.42 N / –
g:Cordana (0.05%) 0.9 0.44 – / –
s:Cordana_terrestris (0.05%) 0.9 0.44 – / –
ESV00584 (0.02%) 0.7 0.54 – / –
ESV00661 (0.03%) 1.0 0.42 N / –
g:Dendrosporium (0.001%) 1.0 0.42 N / –
g:Dendrosporium_uncl (0.001%) 1.0 0.42 N / –
ESV03415 (0.001%) 1.0 0.42 N / –
g:Rhexodenticula (0.02%) 1.0 0.42 N / –

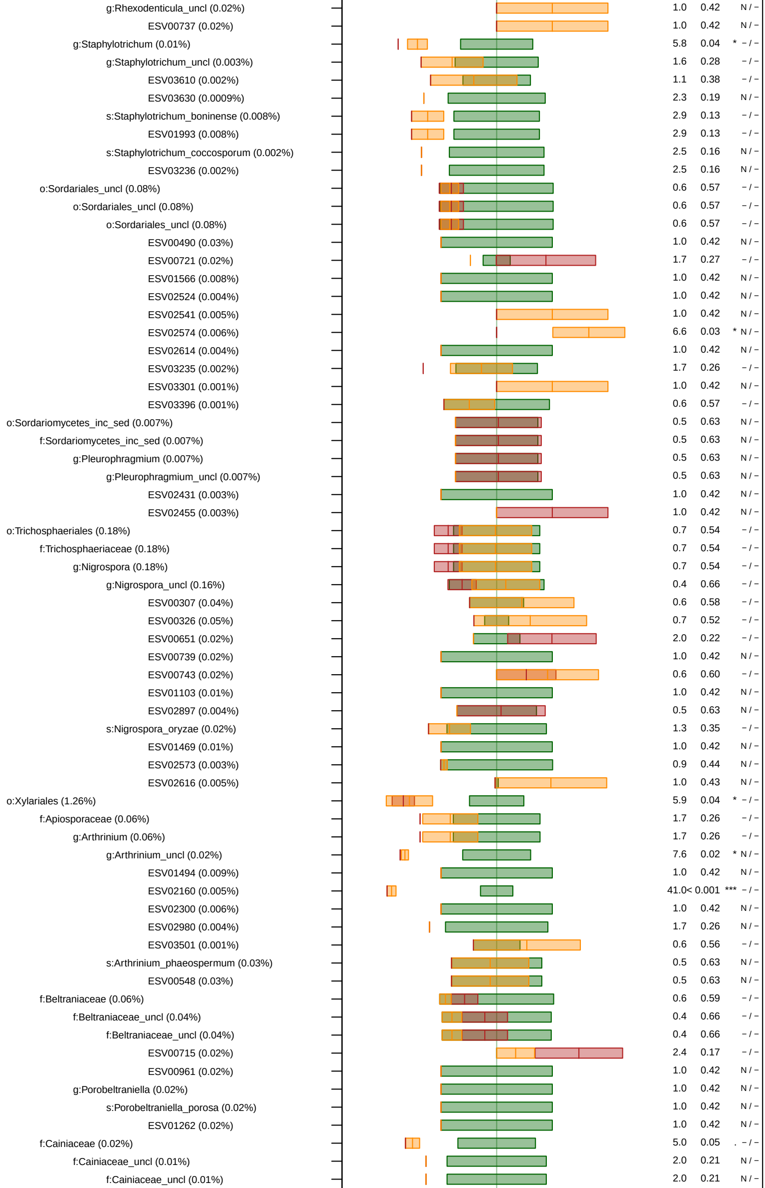

g:Rhexodenticula_uncl (0.02%) 1.0 0.42 N / –
ESV00737 (0.02%) 1.0 0.42 N / –
g:Staphylotrichum (0.01%) 5.8 0.04 * – / –
g:Staphylotrichum_uncl (0.003%) 1.6 0.28 – / –
ESV03610 (0.002%) 1.1 0.38 – / –
ESV03630 (0.0009%) 2.3 0.19 N / –
s:Staphylotrichum_boninense (0.008%) 2.9 0.13 – / –
ESV01993 (0.008%) 2.9 0.13 – / –
s:Staphylotrichum_coccosporum (0.002%) 2.5 0.16 N / –
ESV03236 (0.002%) 2.5 0.16 N / –
o:Sordariales_uncl (0.08%) 0.6 0.57 – / –
o:Sordariales_uncl (0.08%) 0.6 0.57 – / –
o:Sordariales_uncl (0.08%) 0.6 0.57 – / –
ESV00490 (0.03%) 1.0 0.42 N / –
ESV00721 (0.02%) 1.7 0.27 – / –
ESV01566 (0.008%) 1.0 0.42 N / –
ESV02524 (0.004%) 1.0 0.42 N / –
ESV02541 (0.005%) 1.0 0.42 N / –
ESV02574 (0.006%) 6.6 0.03 * N / –
ESV02614 (0.004%) 1.0 0.42 N / –
ESV03235 (0.002%) 1.7 0.26 – / –
ESV03301 (0.001%) 1.0 0.42 N / –
ESV03396 (0.001%) 0.6 0.57 – / –
o:Sordariomycetes_inc_sed (0.007%) 0.5 0.63 N / –
f:Sordariomycetes_inc_sed (0.007%) 0.5 0.63 N / –
g:Pleurophragmium (0.007%) 0.5 0.63 N / –
g:Pleurophragmium_uncl (0.007%) 0.5 0.63 N / –
ESV02431 (0.003%) 1.0 0.42 N / –
ESV02455 (0.003%) 1.0 0.42 N / –
o:Trichosphaeriales (0.18%) 0.7 0.54 – / –
f:Trichosphaeriaceae (0.18%) 0.7 0.54 – / –
g:Nigrospora (0.18%) 0.7 0.54 – / –
g:Nigrospora_uncl (0.16%) 0.4 0.66 – / –
ESV00307 (0.04%) 0.6 0.58 – / –
ESV00326 (0.05%) 0.7 0.52 – / –
ESV00651 (0.02%) 2.0 0.22 – / –
ESV00739 (0.02%) 1.0 0.42 N / –
ESV00743 (0.02%) 0.6 0.60 – / –
ESV01103 (0.01%) 1.0 0.42 N / –
ESV02897 (0.004%) 0.5 0.63 N / –
s:Nigrospora_oryzae (0.02%) 1.3 0.35 – / –
ESV01469 (0.01%) 1.0 0.42 N / –
ESV02573 (0.003%) 0.9 0.44 N / –
ESV02616 (0.005%) 1.0 0.43 N / –
o:Xylariales (1.26%) 5.9 0.04 * – / –
f:Apiosporaceae (0.06%) 1.7 0.26 – / –
g:Arthrinium (0.06%) 1.7 0.26 – / –
g:Arthrinium_uncl (0.02%) 7.6 0.02 * N / –
ESV01494 (0.009%) 1.0 0.42 N / –
ESV02160 (0.005%) 41.0 < 0.001 *** – / –
ESV02300 (0.006%) 1.0 0.42 N / –
ESV02980 (0.004%) 1.7 0.26 N / –
ESV03501 (0.001%) 0.6 0.56 – / –
s:Arthrinium_phaeospermum (0.03%) 0.5 0.63 N / –
ESV00548 (0.03%) 0.5 0.63 N / –
f:Beltraniaceae (0.06%) 0.6 0.59 – / –
f:Beltraniaceae_uncl (0.04%) 0.4 0.66 – / –
f:Beltraniaceae_uncl (0.04%) 0.4 0.66 – / –
ESV00715 (0.02%) 2.4 0.17 – / –
ESV00961 (0.02%) 1.0 0.42 N / –
g:Porobeltraniella (0.02%) 1.0 0.42 N / –
s:Porobeltraniella_porosa (0.02%) 1.0 0.42 N / –
ESV01262 (0.02%) 1.0 0.42 N / –
f:Cainiaceae (0.02%) 5.0 0.05 . – / –
f:Cainiaceae_uncl (0.01%) 2.0 0.21 N / –
f:Cainiaceae_uncl (0.01%) 2.0 0.21 N / –

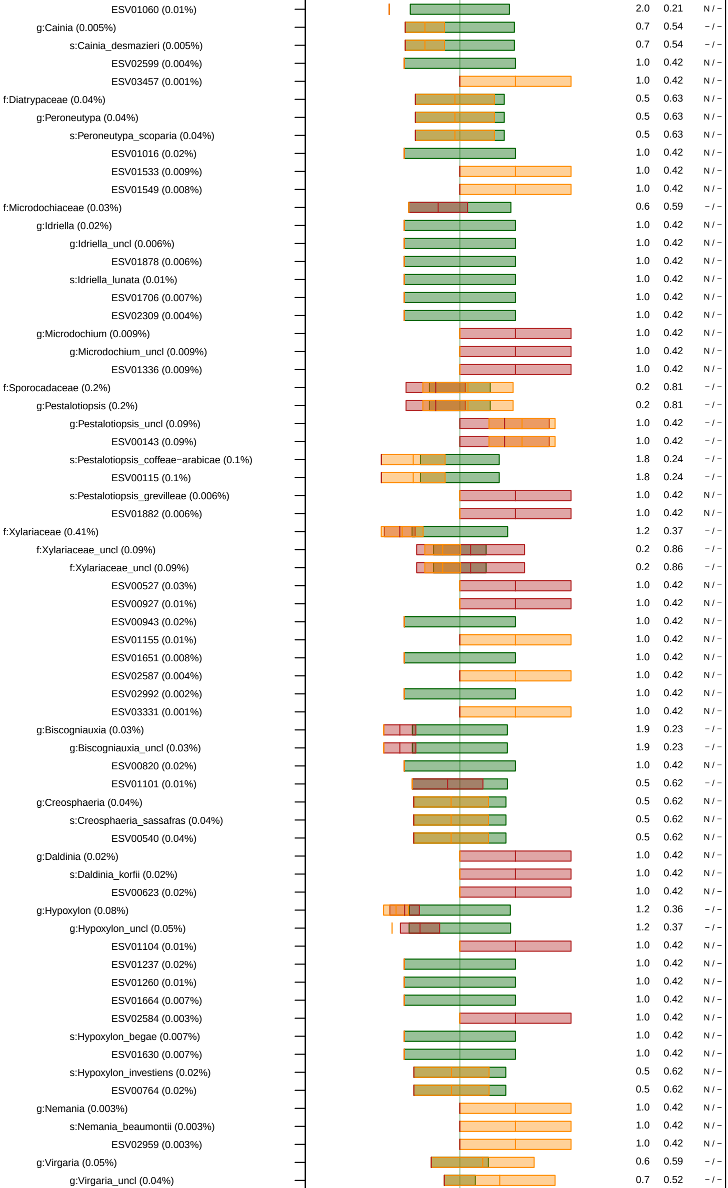
ESV01060 (0.01%) 2.0 0.21 N / −
g:Cainia (0.005%) 0.7 0.54 − / −
s:Cainia_desmazieri (0.005%) 0.7 0.54 − / −
ESV02599 (0.004%) 1.0 0.42 N / −
ESV03457 (0.001%) 1.0 0.42 N / −
f:Diatrypaceae (0.04%) 0.5 0.63 N / −
g:Peroneutypa (0.04%) 0.5 0.63 N / −
s:Peroneutypa_scoparia (0.04%) 0.5 0.63 N / −
ESV01016 (0.02%) 1.0 0.42 N / −
ESV01533 (0.009%) 1.0 0.42 N / −
ESV01549 (0.008%) 1.0 0.42 N / −
f:Microdochiaceae (0.03%) 0.6 0.59 − / −
g:Idriella (0.02%) 1.0 0.42 N / −
g:Idriella_uncl (0.006%) 1.0 0.42 N / −
ESV01878 (0.006%) 1.0 0.42 N / −
s:Idriella_lunata (0.01%) 1.0 0.42 N / −
ESV01706 (0.007%) 1.0 0.42 N / −
ESV02309 (0.004%) 1.0 0.42 N / −
g:Microdochium (0.009%) 1.0 0.42 N / −
g:Microdochium_uncl (0.009%) 1.0 0.42 N / −
ESV01336 (0.009%) 1.0 0.42 N / −
f:Sporocadaceae (0.2%) 0.2 0.81 − / −
g:Pestalotiopsis (0.2%) 0.2 0.81 − / −
g:Pestalotiopsis_uncl (0.09%) 1.0 0.42 − / −
ESV00143 (0.09%) 1.0 0.42 − / −
s:Pestalotiopsis_coffeae−arabicae (0.1%) 1.8 0.24 − / −
ESV00115 (0.1%) 1.8 0.24 − / −
s:Pestalotiopsis_grevilleae (0.006%) 1.0 0.42 N / −
ESV01882 (0.006%) 1.0 0.42 N / −
f:Xylariaceae (0.41%) 1.2 0.37 − / −
f:Xylariaceae_uncl (0.09%) 0.2 0.86 − / −
f:Xylariaceae_uncl (0.09%) 0.2 0.86 − / −
ESV00527 (0.03%) 1.0 0.42 N / −
ESV00927 (0.01%) 1.0 0.42 N / −
ESV00943 (0.02%) 1.0 0.42 N / −
ESV01155 (0.01%) 1.0 0.42 N / −
ESV01651 (0.008%) 1.0 0.42 N / −
ESV02587 (0.004%) 1.0 0.42 N / −
ESV02992 (0.002%) 1.0 0.42 N / −
ESV03331 (0.001%) 1.0 0.42 N / −
g:Biscogniauxia (0.03%) 1.9 0.23 − / −
g:Biscogniauxia_uncl (0.03%) 1.9 0.23 − / −
ESV00820 (0.02%) 1.0 0.42 N / −
ESV01101 (0.01%) 0.5 0.62 − / −
g:Creosphaeria (0.04%) 0.5 0.62 N / −
s:Creosphaeria_sassafras (0.04%) 0.5 0.62 N / −
ESV00540 (0.04%) 0.5 0.62 N / −
g:Daldinia (0.02%) 1.0 0.42 N / −
s:Daldinia_korfii (0.02%) 1.0 0.42 N / −
ESV00623 (0.02%) 1.0 0.42 N / −
g:Hypoxylon (0.08%) 1.2 0.36 − / −
g:Hypoxylon_uncl (0.05%) 1.2 0.37 − / −
ESV01104 (0.01%) 1.0 0.42 N / −
ESV01237 (0.02%) 1.0 0.42 N / −
ESV01260 (0.01%) 1.0 0.42 N / −
ESV01664 (0.007%) 1.0 0.42 N / −
ESV02584 (0.003%) 1.0 0.42 N / −
s:Hypoxylon_begae (0.007%) 1.0 0.42 N / −
ESV01630 (0.007%) 1.0 0.42 N / −
s:Hypoxylon_investiens (0.02%) 0.5 0.62 N / −
ESV00764 (0.02%) 0.5 0.62 N / −
g:Nemania (0.003%) 1.0 0.42 N / −
s:Nemania_beaumontii (0.003%) 1.0 0.42 N / −
ESV02959 (0.003%) 1.0 0.42 N / −
g:Virgaria (0.05%) 0.6 0.59 − / −
g:Virgaria_uncl (0.04%) 0.7 0.52 − / −

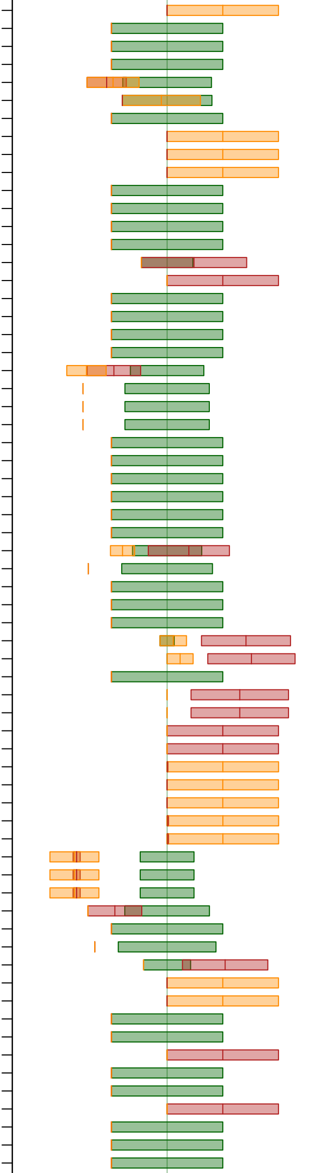

ESV00544 (0.03%)	1.0	0.42	N / –
ESV01399 (0.009%)	1.0	0.42	N / –
s:Virgaria_nigra (0.009%)	1.0	0.42	N / –
ESV01372 (0.009%)	1.0	0.42	N / –
g:Xylaria (0.09%)	1.1	0.39	– / –
g:Xylaria_uncl (0.04%)	0.5	0.63	N / –
ESV00932 (0.02%)	1.0	0.42	N / –
ESV01694 (0.01%)	1.0	0.42	N / –
ESV02480 (0.005%)	1.0	0.42	N / –
ESV02654 (0.004%)	1.0	0.42	N / –
s:Xylaria_curta (0.004%)	1.0	0.42	N / –
ESV02234 (0.004%)	1.0	0.42	N / –
s:Xylaria_hypoxylon (0.006%)	1.0	0.42	N / –
ESV01632 (0.006%)	1.0	0.42	N / –
s:Xylaria_multiplex (0.02%)	0.6	0.58	– / –
ESV00868 (0.01%)	1.0	0.42	N / –
ESV01830 (0.007%)	1.0	0.42	N / –
s:Xylaria_venosula (0.02%)	1.0	0.42	N / –
ESV01544 (0.01%)	1.0	0.42	N / –
ESV01989 (0.008%)	1.0	0.42	N / –
f:Xylariales_inc_sed (0.18%)	2.1	0.21	– / –
f:Xylariales_inc_sed_uncl (0.02%)	4.0	0.08	. N / –
f:Xylariales_inc_sed_uncl (0.02%)	4.0	0.08	. N / –
ESV00663 (0.02%)	4.0	0.08	. N / –
g:Beltraniopsis (0.004%)	1.0	0.42	N / –
s:Beltraniopsis_neolitseae (0.004%)	1.0	0.42	N / –
ESV02363 (0.004%)	1.0	0.42	N / –
g:Castanediella (0.04%)	1.0	0.42	N / –
s:Castanediella_couratarii (0.04%)	1.0	0.42	N / –
ESV00355 (0.04%)	1.0	0.42	N / –
g:Fusidium (0.08%)	1.1	0.38	– / –
g:Fusidium_uncl (0.03%)	3.0	0.12	N / –
ESV01180 (0.01%)	1.0	0.42	N / –
ESV01423 (0.01%)	1.0	0.42	N / –
ESV01585 (0.008%)	1.0	0.42	N / –
s:Fusidium_griseum (0.06%)	2.6	0.15	– / –
ESV00256 (0.05%)	3.0	0.13	– / –
ESV02343 (0.004%)	1.0	0.42	N / –
g:Phialemoniopsis (0.006%)	2.2	0.19	N / –
g:Phialemoniopsis_uncl (0.006%)	2.2	0.19	N / –
ESV02375 (0.004%)	1.0	0.42	N / –
ESV03201 (0.001%)	1.0	0.42	N / –
g:Robillarda (0.02%)	1.0	0.42	N / –
g:Robillarda_uncl (0.009%)	1.0	0.42	N / –
ESV01528 (0.009%)	1.0	0.42	N / –
s:Robillarda_terrae (0.01%)	1.0	0.43	N / –
ESV01222 (0.01%)	1.0	0.43	N / –
o:Xylariales_uncl (0.28%)	6.3	0.03	* – / –
o:Xylariales_uncl (0.28%)	6.3	0.03	* – / –
o:Xylariales_uncl (0.28%)	6.3	0.03	* – / –
ESV00221 (0.05%)	1.9	0.23	– / –
ESV00299 (0.06%)	1.0	0.42	N / –
ESV00390 (0.03%)	2.2	0.19	N / –
ESV00444 (0.03%)	2.2	0.19	– / –
ESV00475 (0.03%)	1.0	0.42	N / –
ESV01195 (0.01%)	1.0	0.42	N / –
ESV01301 (0.01%)	1.0	0.42	N / –
ESV01433 (0.01%)	1.0	0.42	N / –
ESV01530 (0.007%)	1.0	0.42	N / –
ESV01729 (0.01%)	1.0	0.42	N / –
ESV01803 (0.007%)	1.0	0.42	N / –
ESV02027 (0.005%)	1.0	0.42	N / –
ESV02053 (0.005%)	1.0	0.42	N / –
ESV02418 (0.005%)	1.0	0.42	N / –
ESV02819 (0.005%)	1.0	0.42	N / –

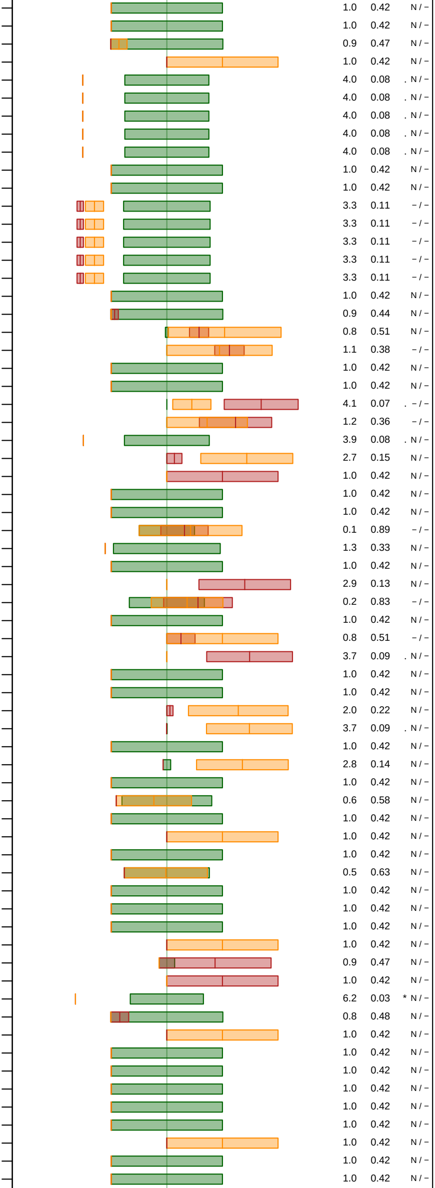

ESV03005 (0.003%)	1.0	0.42		N / –
ESV03207 (0.001%)	1.0	0.42		N / –
ESV03239 (0.002%)	0.9	0.47		N / –
ESV03266 (0.002%)	1.0	0.42		N / –
c:Xylonomycetes (0.04%)	4.0	0.08	.	N / –
o:GS34 (0.04%)	4.0	0.08	.	N / –
o:GS34_uncl (0.04%)	4.0	0.08	.	N / –
o:GS34_uncl (0.04%)	4.0	0.08	.	N / –
o:GS34_uncl (0.04%)	4.0	0.08	.	N / –
ESV00711 (0.02%)	1.0	0.42		N / –
ESV00956 (0.02%)	1.0	0.42		N / –
p:Ascomycota_uncl (2.54%)	3.3	0.11		– / –
p:Ascomycota_uncl (2.54%)	3.3	0.11		– / –
p:Ascomycota_uncl (2.54%)	3.3	0.11		– / –
p:Ascomycota_uncl (2.54%)	3.3	0.11		– / –
p:Ascomycota_uncl (2.54%)	3.3	0.11		– / –
ESV00068 (0.15%)	1.0	0.42		N / –
ESV00078 (0.13%)	0.9	0.44		N / –
ESV00082 (0.17%)	0.8	0.51		N / –
ESV00113 (0.12%)	1.1	0.38		– / –
ESV00181 (0.07%)	1.0	0.42		N / –
ESV00212 (0.06%)	1.0	0.42		N / –
ESV00214 (0.07%)	4.1	0.07	.	– / –
ESV00223 (0.06%)	1.2	0.36		– / –
ESV00272 (0.05%)	3.9	0.08	.	N / –
ESV00277 (0.05%)	2.7	0.15		N / –
ESV00305 (0.04%)	1.0	0.42		N / –
ESV00322 (0.03%)	1.0	0.42		N / –
ESV00324 (0.04%)	1.0	0.42		N / –
ESV00331 (0.04%)	0.1	0.89		– / –
ESV00394 (0.04%)	1.3	0.33		N / –
ESV00395 (0.04%)	1.0	0.42		N / –
ESV00398 (0.03%)	2.9	0.13		N / –
ESV00399 (0.03%)	0.2	0.83		– / –
ESV00427 (0.03%)	1.0	0.42		N / –
ESV00429 (0.03%)	0.8	0.51		– / –
ESV00457 (0.03%)	3.7	0.09	.	N / –
ESV00515 (0.03%)	1.0	0.42		N / –
ESV00534 (0.03%)	1.0	0.42		N / –
ESV00535 (0.03%)	2.0	0.22		N / –
ESV00546 (0.04%)	3.7	0.09	.	N / –
ESV00585 (0.02%)	1.0	0.42		N / –
ESV00601 (0.04%)	2.8	0.14		N / –
ESV00618 (0.02%)	1.0	0.42		N / –
ESV00629 (0.02%)	0.6	0.58		N / –
ESV00640 (0.02%)	1.0	0.42		N / –
ESV00642 (0.02%)	1.0	0.42		N / –
ESV00658 (0.02%)	1.0	0.42		N / –
ESV00675 (0.03%)	0.5	0.63		N / –
ESV00676 (0.03%)	1.0	0.42		N / –
ESV00677 (0.02%)	1.0	0.42		N / –
ESV00686 (0.02%)	1.0	0.42		N / –
ESV00702 (0.03%)	1.0	0.42		N / –
ESV00707 (0.02%)	0.9	0.47		N / –
ESV00745 (0.02%)	1.0	0.42		N / –
ESV00795 (0.02%)	6.2	0.03	*	N / –
ESV00836 (0.01%)	0.8	0.48		N / –
ESV00847 (0.02%)	1.0	0.42		N / –
ESV00872 (0.02%)	1.0	0.42		N / –
ESV00892 (0.02%)	1.0	0.42		N / –
ESV00913 (0.01%)	1.0	0.42		N / –
ESV00929 (0.01%)	1.0	0.42		N / –
ESV00949 (0.02%)	1.0	0.42		N / –
ESV00959 (0.02%)	1.0	0.42		N / –
ESV01027 (0.01%)	1.0	0.42		N / –
ESV01031 (0.02%)	1.0	0.42		N / –

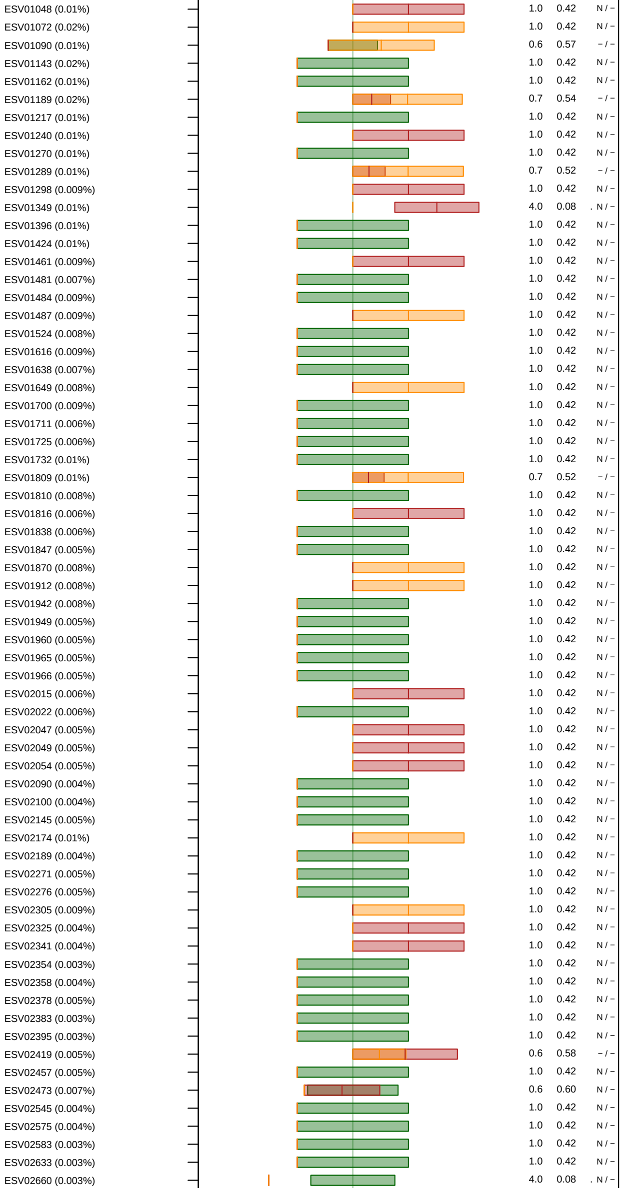
ESV01048 (0.01%) 1.0 0.42 N / –
ESV01072 (0.02%) 1.0 0.42 N / –
ESV01090 (0.01%) 0.6 0.57 – / –
ESV01143 (0.02%) 1.0 0.42 N / –
ESV01162 (0.01%) 1.0 0.42 N / –
ESV01189 (0.02%) 0.7 0.54 – / –
ESV01217 (0.01%) 1.0 0.42 N / –
ESV01240 (0.01%) 1.0 0.42 N / –
ESV01270 (0.01%) 1.0 0.42 N / –
ESV01289 (0.01%) 0.7 0.52 – / –
ESV01298 (0.009%) 1.0 0.42 N / –
ESV01349 (0.01%) 4.0 0.08 . N / –
ESV01396 (0.01%) 1.0 0.42 N / –
ESV01424 (0.01%) 1.0 0.42 N / –
ESV01461 (0.009%) 1.0 0.42 N / –
ESV01481 (0.007%) 1.0 0.42 N / –
ESV01484 (0.009%) 1.0 0.42 N / –
ESV01487 (0.009%) 1.0 0.42 N / –
ESV01524 (0.008%) 1.0 0.42 N / –
ESV01616 (0.009%) 1.0 0.42 N / –
ESV01638 (0.007%) 1.0 0.42 N / –
ESV01649 (0.008%) 1.0 0.42 N / –
ESV01700 (0.009%) 1.0 0.42 N / –
ESV01711 (0.006%) 1.0 0.42 N / –
ESV01725 (0.006%) 1.0 0.42 N / –
ESV01732 (0.01%) 1.0 0.42 N / –
ESV01809 (0.01%) 0.7 0.52 – / –
ESV01810 (0.008%) 1.0 0.42 N / –
ESV01816 (0.006%) 1.0 0.42 N / –
ESV01838 (0.006%) 1.0 0.42 N / –
ESV01847 (0.005%) 1.0 0.42 N / –
ESV01870 (0.008%) 1.0 0.42 N / –
ESV01912 (0.008%) 1.0 0.42 N / –
ESV01942 (0.008%) 1.0 0.42 N / –
ESV01949 (0.005%) 1.0 0.42 N / –
ESV01960 (0.005%) 1.0 0.42 N / –
ESV01965 (0.005%) 1.0 0.42 N / –
ESV01966 (0.005%) 1.0 0.42 N / –
ESV02015 (0.006%) 1.0 0.42 N / –
ESV02022 (0.006%) 1.0 0.42 N / –
ESV02047 (0.005%) 1.0 0.42 N / –
ESV02049 (0.005%) 1.0 0.42 N / –
ESV02054 (0.005%) 1.0 0.42 N / –
ESV02090 (0.004%) 1.0 0.42 N / –
ESV02100 (0.004%) 1.0 0.42 N / –
ESV02145 (0.005%) 1.0 0.42 N / –
ESV02174 (0.01%) 1.0 0.42 N / –
ESV02189 (0.004%) 1.0 0.42 N / –
ESV02271 (0.005%) 1.0 0.42 N / –
ESV02276 (0.005%) 1.0 0.42 N / –
ESV02305 (0.009%) 1.0 0.42 N / –
ESV02325 (0.004%) 1.0 0.42 N / –
ESV02341 (0.004%) 1.0 0.42 N / –
ESV02354 (0.003%) 1.0 0.42 N / –
ESV02358 (0.004%) 1.0 0.42 N / –
ESV02378 (0.005%) 1.0 0.42 N / –
ESV02383 (0.003%) 1.0 0.42 N / –
ESV02395 (0.003%) 1.0 0.42 N / –
ESV02419 (0.005%) 0.6 0.58 – / –
ESV02457 (0.005%) 1.0 0.42 N / –
ESV02473 (0.007%) 0.6 0.60 N / –
ESV02545 (0.004%) 1.0 0.42 N / –
ESV02575 (0.004%) 1.0 0.42 N / –
ESV02583 (0.003%) 1.0 0.42 N / –
ESV02633 (0.003%) 1.0 0.42 N / –
ESV02660 (0.003%) 4.0 0.08 . N / –

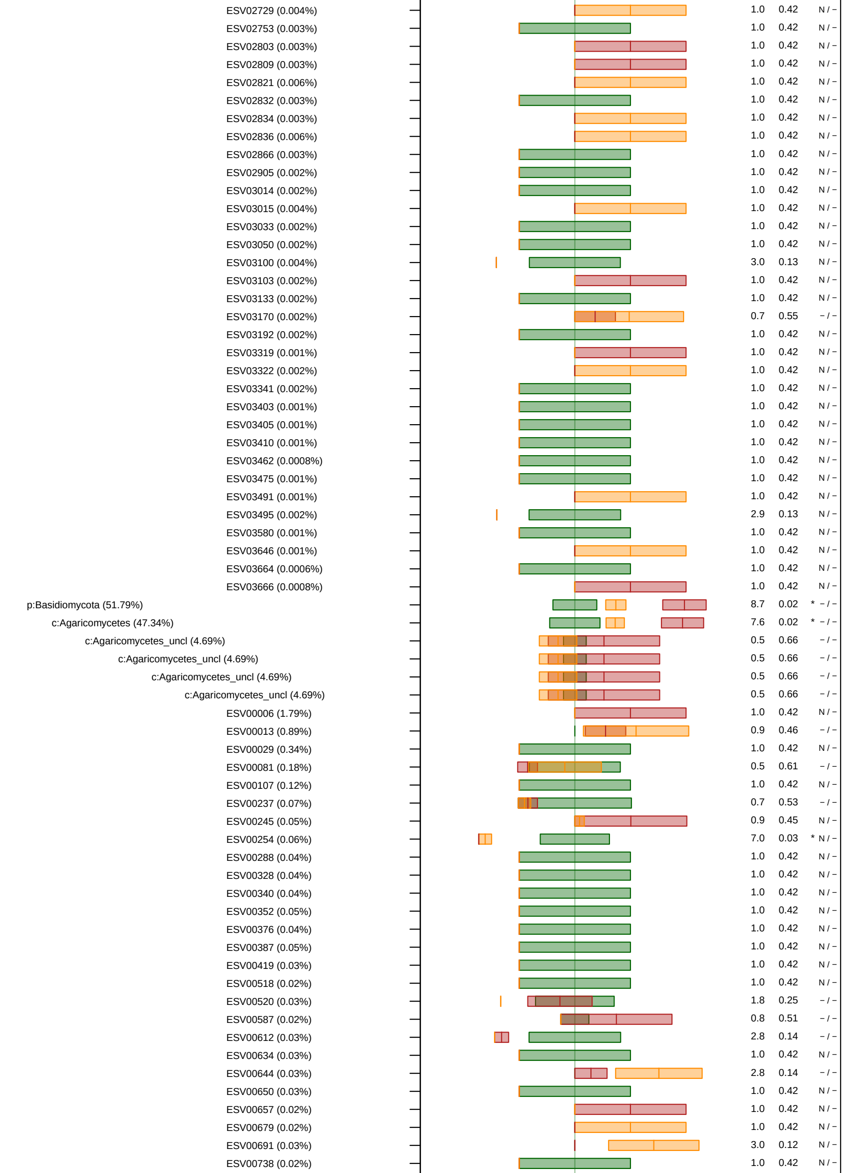

ESV02729 (0.004%) 1.0 0.42 N / –
ESV02753 (0.003%) 1.0 0.42 N / –
ESV02803 (0.003%) 1.0 0.42 N / –
ESV02809 (0.003%) 1.0 0.42 N / –
ESV02821 (0.006%) 1.0 0.42 N / –
ESV02832 (0.003%) 1.0 0.42 N / –
ESV02834 (0.003%) 1.0 0.42 N / –
ESV02836 (0.006%) 1.0 0.42 N / –
ESV02866 (0.003%) 1.0 0.42 N / –
ESV02905 (0.002%) 1.0 0.42 N / –
ESV03014 (0.002%) 1.0 0.42 N / –
ESV03015 (0.004%) 1.0 0.42 N / –
ESV03033 (0.002%) 1.0 0.42 N / –
ESV03050 (0.002%) 1.0 0.42 N / –
ESV03100 (0.004%) 3.0 0.13 N / –
ESV03103 (0.002%) 1.0 0.42 N / –
ESV03133 (0.002%) 1.0 0.42 N / –
ESV03170 (0.002%) 0.7 0.55 – / –
ESV03192 (0.002%) 1.0 0.42 N / –
ESV03319 (0.001%) 1.0 0.42 N / –
ESV03322 (0.002%) 1.0 0.42 N / –
ESV03341 (0.002%) 1.0 0.42 N / –
ESV03403 (0.001%) 1.0 0.42 N / –
ESV03405 (0.001%) 1.0 0.42 N / –
ESV03410 (0.001%) 1.0 0.42 N / –
ESV03462 (0.0008%) 1.0 0.42 N / –
ESV03475 (0.001%) 1.0 0.42 N / –
ESV03491 (0.001%) 1.0 0.42 N / –
ESV03495 (0.002%) 2.9 0.13 N / –
ESV03580 (0.001%) 1.0 0.42 N / –
ESV03646 (0.001%) 1.0 0.42 N / –
ESV03664 (0.0006%) 1.0 0.42 N / –
ESV03666 (0.0008%) 1.0 0.42 N / –
p:Basidiomycota (51.79%) 8.7 0.02 * – / –
c:Agaricomycetes (47.34%) 7.6 0.02 * – / –
c:Agaricomycetes_uncl (4.69%) 0.5 0.66 – / –
c:Agaricomycetes_uncl (4.69%) 0.5 0.66 – / –
c:Agaricomycetes_uncl (4.69%) 0.5 0.66 – / –
c:Agaricomycetes_uncl (4.69%) 0.5 0.66 – / –
ESV00006 (1.79%) 1.0 0.42 N / –
ESV00013 (0.89%) 0.9 0.46 – / –
ESV00029 (0.34%) 1.0 0.42 N / –
ESV00081 (0.18%) 0.5 0.61 – / –
ESV00107 (0.12%) 1.0 0.42 N / –
ESV00237 (0.07%) 0.7 0.53 – / –
ESV00245 (0.05%) 0.9 0.45 N / –
ESV00254 (0.06%) 7.0 0.03 * N / –
ESV00288 (0.04%) 1.0 0.42 N / –
ESV00328 (0.04%) 1.0 0.42 N / –
ESV00340 (0.04%) 1.0 0.42 N / –
ESV00352 (0.05%) 1.0 0.42 N / –
ESV00376 (0.04%) 1.0 0.42 N / –
ESV00387 (0.05%) 1.0 0.42 N / –
ESV00419 (0.03%) 1.0 0.42 N / –
ESV00518 (0.02%) 1.0 0.42 N / –
ESV00520 (0.03%) 1.8 0.25 – / –
ESV00587 (0.02%) 0.8 0.51 – / –
ESV00612 (0.03%) 2.8 0.14 – / –
ESV00634 (0.03%) 1.0 0.42 N / –
ESV00644 (0.03%) 2.8 0.14 – / –
ESV00650 (0.03%) 1.0 0.42 N / –
ESV00657 (0.02%) 1.0 0.42 N / –
ESV00679 (0.02%) 1.0 0.42 N / –
ESV00691 (0.03%) 3.0 0.12 N / –
ESV00738 (0.02%) 1.0 0.42 N / –

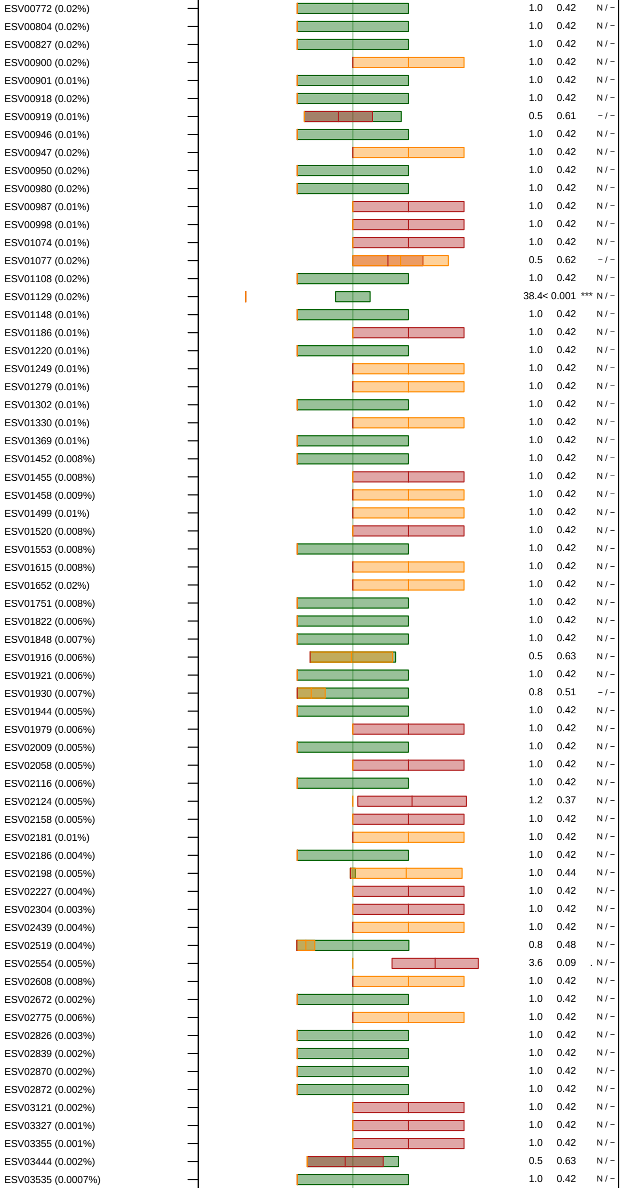
ESV00772 (0.02%) 1.0 0.42 N / –
ESV00804 (0.02%) 1.0 0.42 N / –
ESV00827 (0.02%) 1.0 0.42 N / –
ESV00900 (0.02%) 1.0 0.42 N / –
ESV00901 (0.01%) 1.0 0.42 N / –
ESV00918 (0.02%) 1.0 0.42 N / –
ESV00919 (0.01%) 0.5 0.61 – / –
ESV00946 (0.01%) 1.0 0.42 N / –
ESV00947 (0.02%) 1.0 0.42 N / –
ESV00950 (0.02%) 1.0 0.42 N / –
ESV00980 (0.02%) 1.0 0.42 N / –
ESV00987 (0.01%) 1.0 0.42 N / –
ESV00998 (0.01%) 1.0 0.42 N / –
ESV01074 (0.01%) 1.0 0.42 N / –
ESV01077 (0.02%) 0.5 0.62 – / –
ESV01108 (0.02%) 1.0 0.42 N / –
ESV01129 (0.02%) 38.4 < 0.001 *** N / –
ESV01148 (0.01%) 1.0 0.42 N / –
ESV01186 (0.01%) 1.0 0.42 N / –
ESV01220 (0.01%) 1.0 0.42 N / –
ESV01249 (0.01%) 1.0 0.42 N / –
ESV01279 (0.01%) 1.0 0.42 N / –
ESV01302 (0.01%) 1.0 0.42 N / –
ESV01330 (0.01%) 1.0 0.42 N / –
ESV01369 (0.01%) 1.0 0.42 N / –
ESV01452 (0.008%) 1.0 0.42 N / –
ESV01455 (0.008%) 1.0 0.42 N / –
ESV01458 (0.009%) 1.0 0.42 N / –
ESV01499 (0.01%) 1.0 0.42 N / –
ESV01520 (0.008%) 1.0 0.42 N / –
ESV01553 (0.008%) 1.0 0.42 N / –
ESV01615 (0.008%) 1.0 0.42 N / –
ESV01652 (0.02%) 1.0 0.42 N / –
ESV01751 (0.008%) 1.0 0.42 N / –
ESV01822 (0.006%) 1.0 0.42 N / –
ESV01848 (0.007%) 1.0 0.42 N / –
ESV01916 (0.006%) 0.5 0.63 N / –
ESV01921 (0.006%) 1.0 0.42 N / –
ESV01930 (0.007%) 0.8 0.51 – / –
ESV01944 (0.005%) 1.0 0.42 N / –
ESV01979 (0.006%) 1.0 0.42 N / –
ESV02009 (0.005%) 1.0 0.42 N / –
ESV02058 (0.005%) 1.0 0.42 N / –
ESV02116 (0.006%) 1.0 0.42 N / –
ESV02124 (0.005%) 1.2 0.37 N / –
ESV02158 (0.005%) 1.0 0.42 N / –
ESV02181 (0.01%) 1.0 0.42 N / –
ESV02186 (0.004%) 1.0 0.42 N / –
ESV02198 (0.005%) 1.0 0.44 N / –
ESV02227 (0.004%) 1.0 0.42 N / –
ESV02304 (0.003%) 1.0 0.42 N / –
ESV02439 (0.004%) 1.0 0.42 N / –
ESV02519 (0.004%) 0.8 0.48 N / –
ESV02554 (0.005%) 3.6 0.09 . N / –
ESV02608 (0.008%) 1.0 0.42 N / –
ESV02672 (0.002%) 1.0 0.42 N / –
ESV02775 (0.006%) 1.0 0.42 N / –
ESV02826 (0.003%) 1.0 0.42 N / –
ESV02839 (0.002%) 1.0 0.42 N / –
ESV02870 (0.002%) 1.0 0.42 N / –
ESV02872 (0.002%) 1.0 0.42 N / –
ESV03121 (0.002%) 1.0 0.42 N / –
ESV03327 (0.001%) 1.0 0.42 N / –
ESV03355 (0.001%) 1.0 0.42 N / –
ESV03444 (0.002%) 0.5 0.63 N / –
ESV03535 (0.0007%) 1.0 0.42 N / –

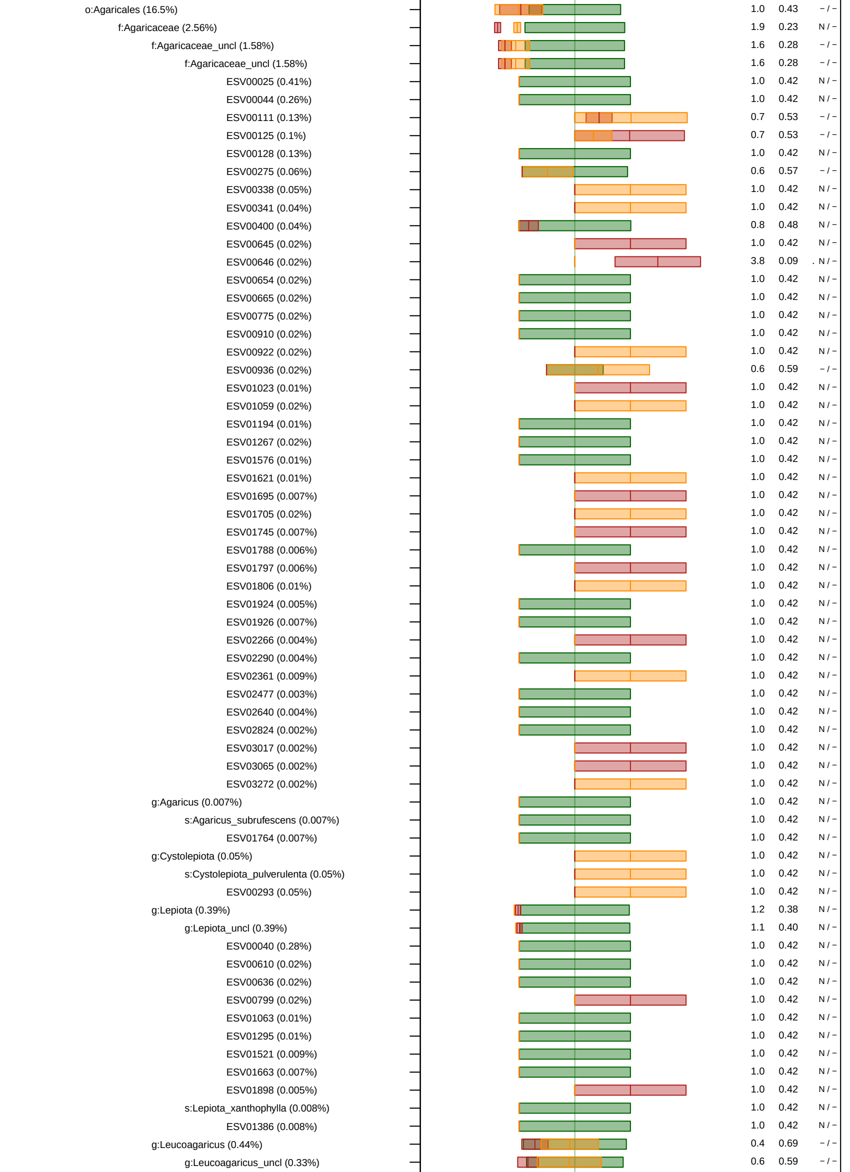
o:Agaricales (16.5%) 1.0 0.43 − / −
f:Agaricaceae (2.56%) 1.9 0.23 N / −
f:Agaricaceae_uncl (1.58%) 1.6 0.28 − / −
f:Agaricaceae_uncl (1.58%) 1.6 0.28 − / −
ESV00025 (0.41%) 1.0 0.42 N / −
ESV00044 (0.26%) 1.0 0.42 N / −
ESV00111 (0.13%) 0.7 0.53 − / −
ESV00125 (0.1%) 0.7 0.53 − / −
ESV00128 (0.13%) 1.0 0.42 N / −
ESV00275 (0.06%) 0.6 0.57 − / −
ESV00338 (0.05%) 1.0 0.42 N / −
ESV00341 (0.04%) 1.0 0.42 N / −
ESV00400 (0.04%) 0.8 0.48 N / −
ESV00645 (0.02%) 1.0 0.42 N / −
ESV00646 (0.02%) 3.8 0.09 . N / −
ESV00654 (0.02%) 1.0 0.42 N / −
ESV00665 (0.02%) 1.0 0.42 N / −
ESV00775 (0.02%) 1.0 0.42 N / −
ESV00910 (0.02%) 1.0 0.42 N / −
ESV00922 (0.02%) 1.0 0.42 N / −
ESV00936 (0.02%) 0.6 0.59 − / −
ESV01023 (0.01%) 1.0 0.42 N / −
ESV01059 (0.02%) 1.0 0.42 N / −
ESV01194 (0.01%) 1.0 0.42 N / −
ESV01267 (0.02%) 1.0 0.42 N / −
ESV01576 (0.01%) 1.0 0.42 N / −
ESV01621 (0.01%) 1.0 0.42 N / −
ESV01695 (0.007%) 1.0 0.42 N / −
ESV01705 (0.02%) 1.0 0.42 N / −
ESV01745 (0.007%) 1.0 0.42 N / −
ESV01788 (0.006%) 1.0 0.42 N / −
ESV01797 (0.006%) 1.0 0.42 N / −
ESV01806 (0.01%) 1.0 0.42 N / −
ESV01924 (0.005%) 1.0 0.42 N / −
ESV01926 (0.007%) 1.0 0.42 N / −
ESV02266 (0.004%) 1.0 0.42 N / −
ESV02290 (0.004%) 1.0 0.42 N / −
ESV02361 (0.009%) 1.0 0.42 N / −
ESV02477 (0.003%) 1.0 0.42 N / −
ESV02640 (0.004%) 1.0 0.42 N / −
ESV02824 (0.002%) 1.0 0.42 N / −
ESV03017 (0.002%) 1.0 0.42 N / −
ESV03065 (0.002%) 1.0 0.42 N / −
ESV03272 (0.002%) 1.0 0.42 N / −
g:Agaricus (0.007%) 1.0 0.42 N / −
s:Agaricus_subrufescens (0.007%) 1.0 0.42 N / −
ESV01764 (0.007%) 1.0 0.42 N / −
g:Cystolepiota (0.05%) 1.0 0.42 N / −
s:Cystolepiota_pulverulenta (0.05%) 1.0 0.42 N / −
ESV00293 (0.05%) 1.0 0.42 N / −
g:Lepiota (0.39%) 1.2 0.38 N / −
g:Lepiota_uncl (0.39%) 1.1 0.40 N / −
ESV00040 (0.28%) 1.0 0.42 N / −
ESV00610 (0.02%) 1.0 0.42 N / −
ESV00636 (0.02%) 1.0 0.42 N / −
ESV00799 (0.02%) 1.0 0.42 N / −
ESV01063 (0.01%) 1.0 0.42 N / −
ESV01295 (0.01%) 1.0 0.42 N / −
ESV01521 (0.009%) 1.0 0.42 N / −
ESV01663 (0.007%) 1.0 0.42 N / −
ESV01898 (0.005%) 1.0 0.42 N / −
s:Lepiota_xanthophylla (0.008%) 1.0 0.42 N / −
ESV01386 (0.008%) 1.0 0.42 N / −
g:Leucoagaricus (0.44%) 0.4 0.69 − / −
g:Leucoagaricus_uncl (0.33%) 0.6 0.59 − / −

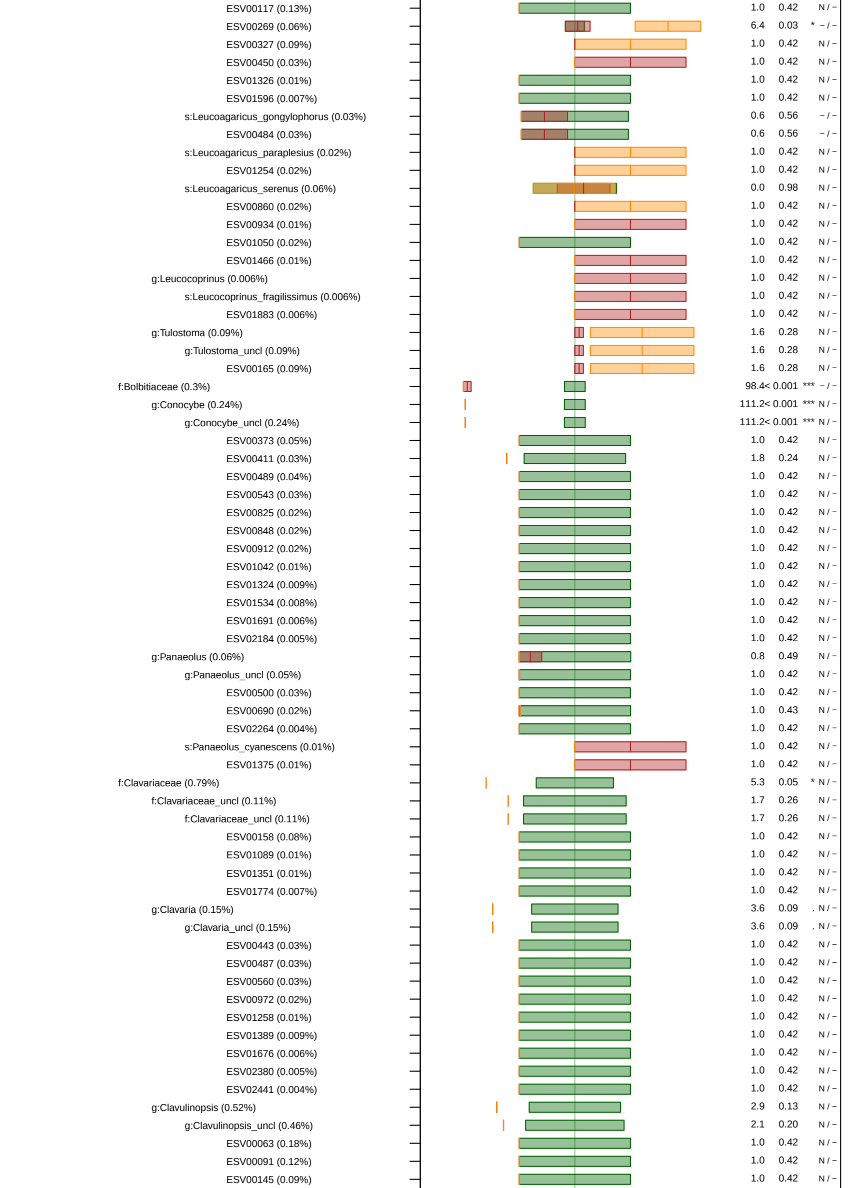
ESV00117 (0.13%) 1.0 0.42 N / –
ESV00269 (0.06%) 6.4 0.03 * – / –
ESV00327 (0.09%) 1.0 0.42 N / –
ESV00450 (0.03%) 1.0 0.42 N / –
ESV01326 (0.01%) 1.0 0.42 N / –
ESV01596 (0.007%) 1.0 0.42 N / –
s:Leucoagaricus_gongylophorus (0.03%) 0.6 0.56 – / –
ESV00484 (0.03%) 0.6 0.56 – / –
s:Leucoagaricus_paraplesius (0.02%) 1.0 0.42 N / –
ESV01254 (0.02%) 1.0 0.42 N / –
s:Leucoagaricus_serenus (0.06%) 0.0 0.98 N / –
ESV00860 (0.02%) 1.0 0.42 N / –
ESV00934 (0.01%) 1.0 0.42 N / –
ESV01050 (0.02%) 1.0 0.42 N / –
ESV01466 (0.01%) 1.0 0.42 N / –
g:Leucocoprinus (0.006%) 1.0 0.42 N / –
s:Leucocoprinus_fragilissimus (0.006%) 1.0 0.42 N / –
ESV01883 (0.006%) 1.0 0.42 N / –
g:Tulostoma (0.09%) 1.6 0.28 N / –
g:Tulostoma_uncl (0.09%) 1.6 0.28 N / –
ESV00165 (0.09%) 1.6 0.28 N / –
f:Bolbitiaceae (0.3%) 98.4 < 0.001 *** – / –
g:Conocybe (0.24%) 111.2 < 0.001 *** N / –
g:Conocybe_uncl (0.24%) 111.2 < 0.001 *** N / –
ESV00373 (0.05%) 1.0 0.42 N / –
ESV00411 (0.03%) 1.8 0.24 N / –
ESV00489 (0.04%) 1.0 0.42 N / –
ESV00543 (0.03%) 1.0 0.42 N / –
ESV00825 (0.02%) 1.0 0.42 N / –
ESV00848 (0.02%) 1.0 0.42 N / –
ESV00912 (0.02%) 1.0 0.42 N / –
ESV01042 (0.01%) 1.0 0.42 N / –
ESV01324 (0.009%) 1.0 0.42 N / –
ESV01534 (0.008%) 1.0 0.42 N / –
ESV01691 (0.006%) 1.0 0.42 N / –
ESV02184 (0.005%) 1.0 0.42 N / –
g:Panaeolus (0.06%) 0.8 0.49 N / –
g:Panaeolus_uncl (0.05%) 1.0 0.42 N / –
ESV00500 (0.03%) 1.0 0.42 N / –
ESV00690 (0.02%) 1.0 0.43 N / –
ESV02264 (0.004%) 1.0 0.42 N / –
s:Panaeolus_cyanescens (0.01%) 1.0 0.42 N / –
ESV01375 (0.01%) 1.0 0.42 N / –
f:Clavariaceae (0.79%) 5.3 0.05 * N / –
f:Clavariaceae_uncl (0.11%) 1.7 0.26 N / –
f:Clavariaceae_uncl (0.11%) 1.7 0.26 N / –
ESV00158 (0.08%) 1.0 0.42 N / –
ESV01089 (0.01%) 1.0 0.42 N / –
ESV01351 (0.01%) 1.0 0.42 N / –
ESV01774 (0.007%) 1.0 0.42 N / –
g:Clavaria (0.15%) 3.6 0.09 . N / –
g:Clavaria_uncl (0.15%) 3.6 0.09 . N / –
ESV00443 (0.03%) 1.0 0.42 N / –
ESV00487 (0.03%) 1.0 0.42 N / –
ESV00560 (0.03%) 1.0 0.42 N / –
ESV00972 (0.02%) 1.0 0.42 N / –
ESV01258 (0.01%) 1.0 0.42 N / –
ESV01389 (0.009%) 1.0 0.42 N / –
ESV01676 (0.006%) 1.0 0.42 N / –
ESV02380 (0.005%) 1.0 0.42 N / –
ESV02441 (0.004%) 1.0 0.42 N / –
g:Clavulinopsis (0.52%) 2.9 0.13 N / –
g:Clavulinopsis_uncl (0.46%) 2.1 0.20 N / –
ESV00063 (0.18%) 1.0 0.42 N / –
ESV00091 (0.12%) 1.0 0.42 N / –
ESV00145 (0.09%) 1.0 0.42 N / –

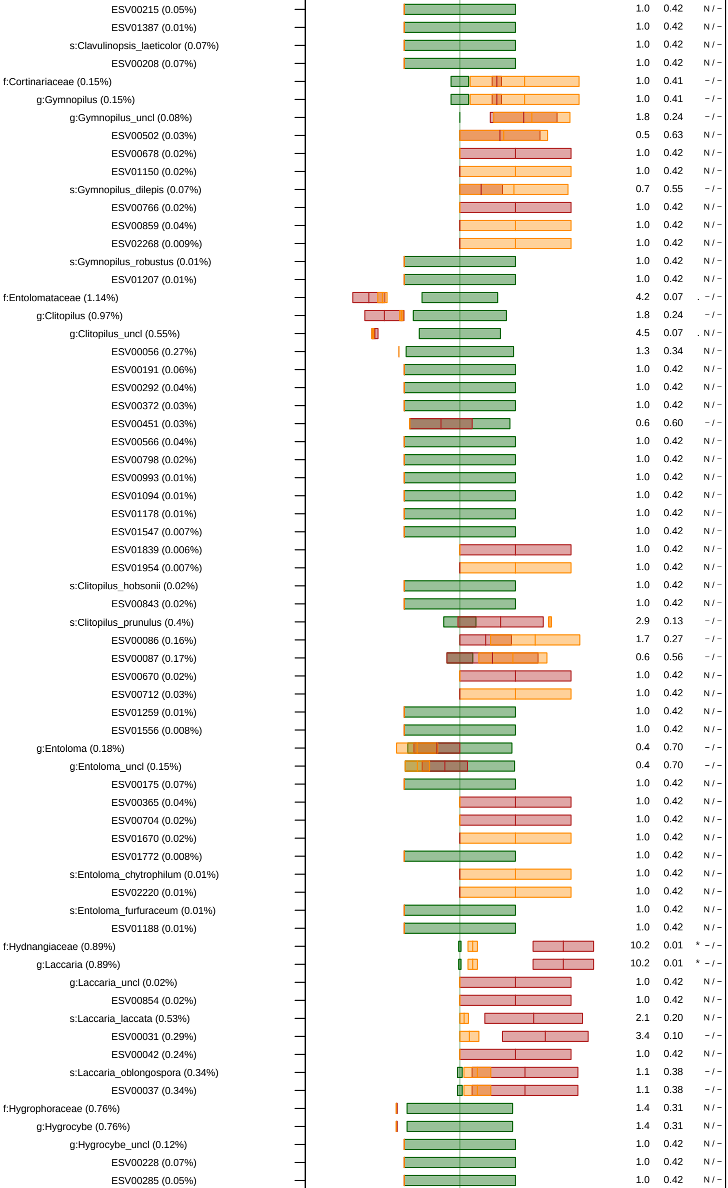
ESV00215 (0.05%) 1.0 0.42 N / –
ESV01387 (0.01%) 1.0 0.42 N / –
s:Clavulinopsis_laeticolor (0.07%) 1.0 0.42 N / –
ESV00208 (0.07%) 1.0 0.42 N / –
f:Cortinariaceae (0.15%) 1.0 0.41 – / –
g:Gymnopilus (0.15%) 1.0 0.41 – / –
g:Gymnopilus_uncl (0.08%) 1.8 0.24 – / –
ESV00502 (0.03%) 0.5 0.63 N / –
ESV00678 (0.02%) 1.0 0.42 N / –
ESV01150 (0.02%) 1.0 0.42 N / –
s:Gymnopilus_dilepis (0.07%) 0.7 0.55 – / –
ESV00766 (0.02%) 1.0 0.42 N / –
ESV00859 (0.04%) 1.0 0.42 N / –
ESV02268 (0.009%) 1.0 0.42 N / –
s:Gymnopilus_robustus (0.01%) 1.0 0.42 N / –
ESV01207 (0.01%) 1.0 0.42 N / –
f:Entolomataceae (1.14%) 4.2 0.07 . – / –
g:Clitopilus (0.97%) 1.8 0.24 – / –
g:Clitopilus_uncl (0.55%) 4.5 0.07 . N / –
ESV00056 (0.27%) 1.3 0.34 N / –
ESV00191 (0.06%) 1.0 0.42 N / –
ESV00292 (0.04%) 1.0 0.42 N / –
ESV00372 (0.03%) 1.0 0.42 N / –
ESV00451 (0.03%) 0.6 0.60 – / –
ESV00566 (0.04%) 1.0 0.42 N / –
ESV00798 (0.02%) 1.0 0.42 N / –
ESV00993 (0.01%) 1.0 0.42 N / –
ESV01094 (0.01%) 1.0 0.42 N / –
ESV01178 (0.01%) 1.0 0.42 N / –
ESV01547 (0.007%) 1.0 0.42 N / –
ESV01839 (0.006%) 1.0 0.42 N / –
ESV01954 (0.007%) 1.0 0.42 N / –
s:Clitopilus_hobsonii (0.02%) 1.0 0.42 N / –
ESV00843 (0.02%) 1.0 0.42 N / –
s:Clitopilus_prunulus (0.4%) 2.9 0.13 – / –
ESV00086 (0.16%) 1.7 0.27 – / –
ESV00087 (0.17%) 0.6 0.56 – / –
ESV00670 (0.02%) 1.0 0.42 N / –
ESV00712 (0.03%) 1.0 0.42 N / –
ESV01259 (0.01%) 1.0 0.42 N / –
ESV01556 (0.008%) 1.0 0.42 N / –
g:Entoloma (0.18%) 0.4 0.70 – / –
g:Entoloma_uncl (0.15%) 0.4 0.70 – / –
ESV00175 (0.07%) 1.0 0.42 N / –
ESV00365 (0.04%) 1.0 0.42 N / –
ESV00704 (0.02%) 1.0 0.42 N / –
ESV01670 (0.02%) 1.0 0.42 N / –
ESV01772 (0.008%) 1.0 0.42 N / –
s:Entoloma_chytrophilum (0.01%) 1.0 0.42 N / –
ESV02220 (0.01%) 1.0 0.42 N / –
s:Entoloma_furfuraceum (0.01%) 1.0 0.42 N / –
ESV01188 (0.01%) 1.0 0.42 N / –
f:Hydnangiaceae (0.89%) 10.2 0.01 * – / –
g:Laccaria (0.89%) 10.2 0.01 * – / –
g:Laccaria_uncl (0.02%) 1.0 0.42 N / –
ESV00854 (0.02%) 1.0 0.42 N / –
s:Laccaria_laccata (0.53%) 2.1 0.20 N / –
ESV00031 (0.29%) 3.4 0.10 – / –
ESV00042 (0.24%) 1.0 0.42 N / –
s:Laccaria_oblongospora (0.34%) 1.1 0.38 – / –
ESV00037 (0.34%) 1.1 0.38 – / –
f:Hygrophoraceae (0.76%) 1.4 0.31 N / –
g:Hygrocybe (0.76%) 1.4 0.31 N / –
g:Hygrocybe_uncl (0.12%) 1.0 0.42 N / –
ESV00228 (0.07%) 1.0 0.42 N / –
ESV00285 (0.05%) 1.0 0.42 N / –

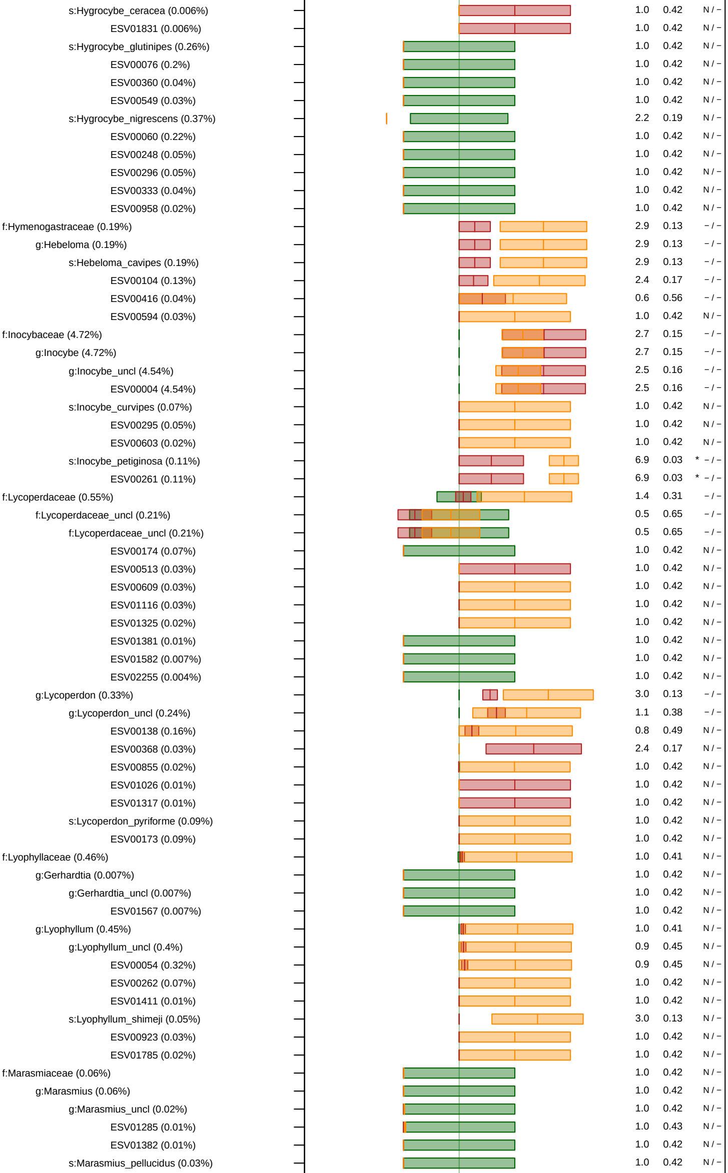

s:Hygrocybe_ceracea (0.006%) 1.0 0.42 N / –
ESV01831 (0.006%) 1.0 0.42 N / –
s:Hygrocybe_glutinipes (0.26%) 1.0 0.42 N / –
ESV00076 (0.2%) 1.0 0.42 N / –
ESV00360 (0.04%) 1.0 0.42 N / –
ESV00549 (0.03%) 1.0 0.42 N / –
s:Hygrocybe_nigrescens (0.37%) 2.2 0.19 N / –
ESV00060 (0.22%) 1.0 0.42 N / –
ESV00248 (0.05%) 1.0 0.42 N / –
ESV00296 (0.05%) 1.0 0.42 N / –
ESV00333 (0.04%) 1.0 0.42 N / –
ESV00958 (0.02%) 1.0 0.42 N / –
f:Hymenogastraceae (0.19%) 2.9 0.13 – / –
g:Hebeloma (0.19%) 2.9 0.13 – / –
s:Hebeloma_cavipes (0.19%) 2.9 0.13 – / –
ESV00104 (0.13%) 2.4 0.17 – / –
ESV00416 (0.04%) 0.6 0.56 – / –
ESV00594 (0.03%) 1.0 0.42 N / –
f:Inocybaceae (4.72%) 2.7 0.15 – / –
g:Inocybe (4.72%) 2.7 0.15 – / –
g:Inocybe_uncl (4.54%) 2.5 0.16 – / –
ESV00004 (4.54%) 2.5 0.16 – / –
s:Inocybe_curvipes (0.07%) 1.0 0.42 N / –
ESV00295 (0.05%) 1.0 0.42 N / –
ESV00603 (0.02%) 1.0 0.42 N / –
s:Inocybe_petiginosa (0.11%) 6.9 0.03 * – / –
ESV00261 (0.11%) 6.9 0.03 * – / –
f:Lycoperdaceae (0.55%) 1.4 0.31 – / –
f:Lycoperdaceae_uncl (0.21%) 0.5 0.65 – / –
f:Lycoperdaceae_uncl (0.21%) 0.5 0.65 – / –
ESV00174 (0.07%) 1.0 0.42 N / –
ESV00513 (0.03%) 1.0 0.42 N / –
ESV00609 (0.03%) 1.0 0.42 N / –
ESV01116 (0.03%) 1.0 0.42 N / –
ESV01325 (0.02%) 1.0 0.42 N / –
ESV01381 (0.01%) 1.0 0.42 N / –
ESV01582 (0.007%) 1.0 0.42 N / –
ESV02255 (0.004%) 1.0 0.42 N / –
g:Lycoperdon (0.33%) 3.0 0.13 – / –
g:Lycoperdon_uncl (0.24%) 1.1 0.38 – / –
ESV00138 (0.16%) 0.8 0.49 N / –
ESV00368 (0.03%) 2.4 0.17 N / –
ESV00855 (0.02%) 1.0 0.42 N / –
ESV01026 (0.01%) 1.0 0.42 N / –
ESV01317 (0.01%) 1.0 0.42 N / –
s:Lycoperdon_pyriforme (0.09%) 1.0 0.42 N / –
ESV00173 (0.09%) 1.0 0.42 N / –
f:Lyophyllaceae (0.46%) 1.0 0.41 N / –
g:Gerhardtia (0.007%) 1.0 0.42 N / –
g:Gerhardtia_uncl (0.007%) 1.0 0.42 N / –
ESV01567 (0.007%) 1.0 0.42 N / –
g:Lyophyllum (0.45%) 1.0 0.41 N / –
g:Lyophyllum_uncl (0.4%) 0.9 0.45 N / –
ESV00054 (0.32%) 0.9 0.45 N / –
ESV00262 (0.07%) 1.0 0.42 N / –
ESV01411 (0.01%) 1.0 0.42 N / –
s:Lyophyllum_shimeji (0.05%) 3.0 0.13 N / –
ESV00923 (0.03%) 1.0 0.42 N / –
ESV01785 (0.02%) 1.0 0.42 N / –
f:Marasmiaceae (0.06%) 1.0 0.42 N / –
g:Marasmius (0.06%) 1.0 0.42 N / –
g:Marasmius_uncl (0.02%) 1.0 0.42 N / –
ESV01285 (0.01%) 1.0 0.43 N / –
ESV01382 (0.01%) 1.0 0.42 N / –
s:Marasmius_pellucidus (0.03%) 1.0 0.42 N / –

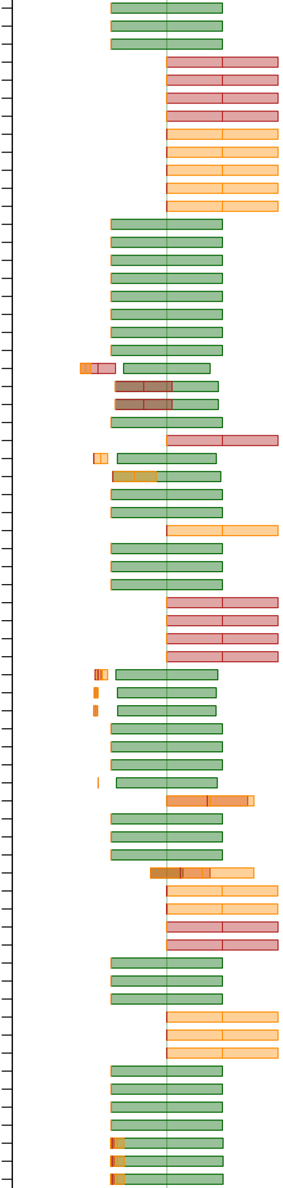

ESV00383 (0.03%)	1.0	0.42	N / –
s:Marasmius_rotula (0.003%)	1.0	0.42	N / –
ESV02823 (0.003%)	1.0	0.42	N / –
f:Mycenaceae (0.02%)	1.0	0.42	N / –
f:Mycenaceae_uncl (0.02%)	1.0	0.42	N / –
f:Mycenaceae_uncl (0.02%)	1.0	0.42	N / –
ESV00692 (0.02%)	1.0	0.42	N / –
f:Omphalotaceae (0.08%)	1.0	0.42	N / –
g:Gymnopus (0.08%)	1.0	0.42	N / –
s:Gymnopus_termiticola (0.08%)	1.0	0.42	N / –
ESV00883 (0.04%)	1.0	0.42	N / –
ESV01070 (0.03%)	1.0	0.42	N / –
f:Physalacriaceae (0.01%)	1.0	0.42	N / –
f:Physalacriaceae_uncl (0.01%)	1.0	0.42	N / –
f:Physalacriaceae_uncl (0.01%)	1.0	0.42	N / –
ESV01996 (0.01%)	1.0	0.42	N / –
f:Pleurotaceae (0.02%)	1.0	0.42	N / –
g:Nematoctonus (0.02%)	1.0	0.42	N / –
g:Nematoctonus_uncl (0.02%)	1.0	0.42	N / –
ESV00751 (0.02%)	1.0	0.42	N / –
f:Pluteaceae (0.07%)	2.6	0.15	– / –
g:Pluteus (0.03%)	0.6	0.59	– / –
g:Pluteus_uncl (0.03%)	0.6	0.59	– / –
ESV00632 (0.02%)	1.0	0.42	N / –
ESV01273 (0.01%)	1.0	0.42	N / –
g:Volvariella (0.04%)	2.0	0.22	– / –
g:Volvariella_uncl (0.01%)	0.7	0.55	– / –
ESV02005 (0.005%)	1.0	0.42	N / –
ESV02845 (0.003%)	1.0	0.42	N / –
ESV03314 (0.003%)	1.0	0.42	N / –
s:Volvariella_surrecta (0.03%)	1.0	0.42	N / –
ESV01379 (0.01%)	1.0	0.42	N / –
ESV01483 (0.01%)	1.0	0.42	N / –
f:Porotheleaceae (0.007%)	1.0	0.42	N / –
f:Porotheleaceae_uncl (0.007%)	1.0	0.42	N / –
f:Porotheleaceae_uncl (0.007%)	1.0	0.42	N / –
ESV01671 (0.007%)	1.0	0.42	N / –
f:Psathyrellaceae (0.38%)	1.7	0.26	N / –
g:Coprinellus (0.33%)	2.0	0.21	N / –
g:Coprinellus_uncl (0.32%)	2.1	0.20	N / –
ESV00057 (0.17%)	1.0	0.42	N / –
ESV00207 (0.05%)	1.0	0.42	N / –
ESV00354 (0.04%)	1.0	0.42	N / –
ESV00501 (0.03%)	1.9	0.24	N / –
ESV00771 (0.02%)	0.5	0.63	N / –
ESV01672 (0.007%)	1.0	0.42	N / –
s:Coprinellus_micaceus (0.008%)	1.0	0.42	N / –
ESV01492 (0.008%)	1.0	0.42	N / –
g:Coprinopsis (0.05%)	0.3	0.78	– / –
s:Coprinopsis_igarashii (0.03%)	1.0	0.42	N / –
ESV00685 (0.03%)	1.0	0.42	N / –
s:Coprinopsis_strossmayeri (0.02%)	1.0	0.42	N / –
ESV00904 (0.02%)	1.0	0.42	N / –
s:Coprinopsis_urticicola (0.008%)	1.0	0.42	N / –
ESV02156 (0.005%)	1.0	0.42	N / –
ESV02702 (0.003%)	1.0	0.42	N / –
g:Parasola (0.007%)	1.0	0.42	N / –
s:Parasola_setulosa (0.007%)	1.0	0.42	N / –
ESV01932 (0.007%)	1.0	0.42	N / –
f:Pterulaceae (0.01%)	1.0	0.42	N / –
g:Pterula (0.01%)	1.0	0.42	N / –
s:Pterula_echo (0.01%)	1.0	0.42	N / –
ESV01172 (0.01%)	1.0	0.42	N / –
f:Stephanosporaceae (0.26%)	0.9	0.47	N / –
f:Stephanosporaceae_uncl (0.25%)	0.8	0.47	N / –
f:Stephanosporaceae_uncl (0.25%)	0.8	0.47	N / –

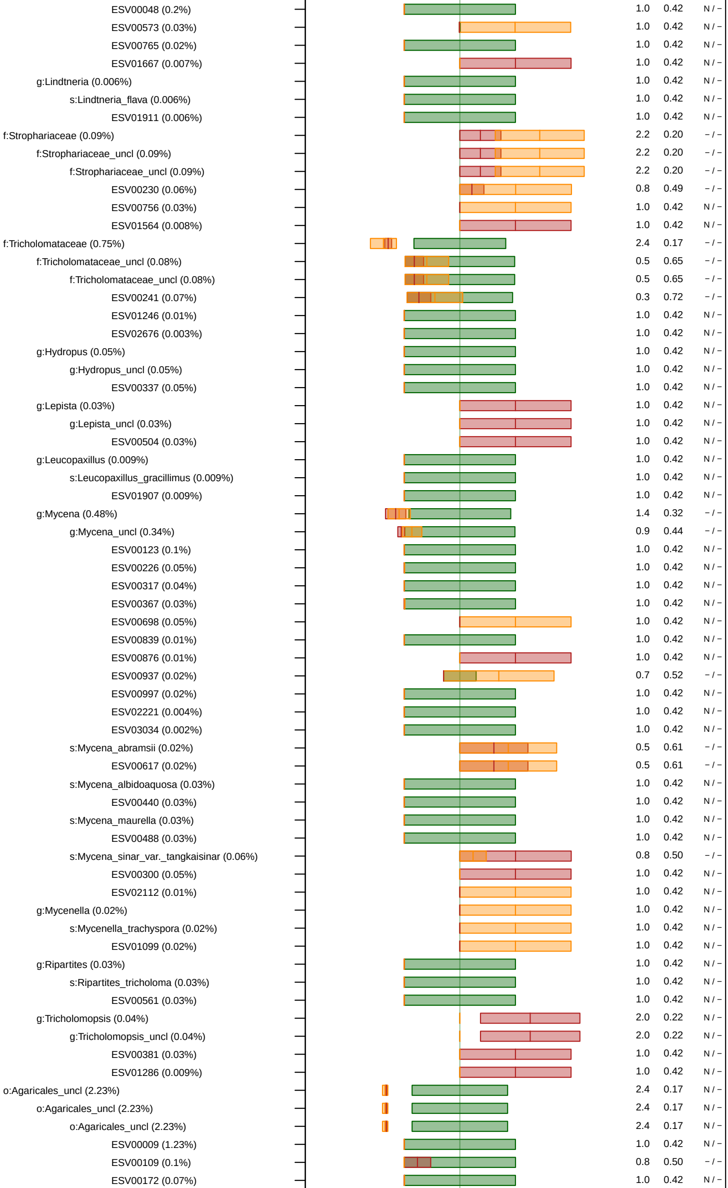
ESV00048 (0.2%) 1.0 0.42 N / –
ESV00573 (0.03%) 1.0 0.42 N / –
ESV00765 (0.02%) 1.0 0.42 N / –
ESV01667 (0.007%) 1.0 0.42 N / –
g:Lindtneria (0.006%) 1.0 0.42 N / –
s:Lindtneria_flava (0.006%) 1.0 0.42 N / –
ESV01911 (0.006%) 1.0 0.42 N / –
f:Strophariaceae (0.09%) 2.2 0.20 – / –
f:Strophariaceae_uncl (0.09%) 2.2 0.20 – / –
f:Strophariaceae_uncl (0.09%) 2.2 0.20 – / –
ESV00230 (0.06%) 0.8 0.49 – / –
ESV00756 (0.03%) 1.0 0.42 N / –
ESV01564 (0.008%) 1.0 0.42 N / –
f:Tricholomataceae (0.75%) 2.4 0.17 – / –
f:Tricholomataceae_uncl (0.08%) 0.5 0.65 – / –
f:Tricholomataceae_uncl (0.08%) 0.5 0.65 – / –
ESV00241 (0.07%) 0.3 0.72 – / –
ESV01246 (0.01%) 1.0 0.42 N / –
ESV02676 (0.003%) 1.0 0.42 N / –
g:Hydropus (0.05%) 1.0 0.42 N / –
g:Hydropus_uncl (0.05%) 1.0 0.42 N / –
ESV00337 (0.05%) 1.0 0.42 N / –
g:Lepista (0.03%) 1.0 0.42 N / –
g:Lepista_uncl (0.03%) 1.0 0.42 N / –
ESV00504 (0.03%) 1.0 0.42 N / –
g:Leucopaxillus (0.009%) 1.0 0.42 N / –
s:Leucopaxillus_gracillimus (0.009%) 1.0 0.42 N / –
ESV01907 (0.009%) 1.0 0.42 N / –
g:Mycena (0.48%) 1.4 0.32 – / –
g:Mycena_uncl (0.34%) 0.9 0.44 – / –
ESV00123 (0.1%) 1.0 0.42 N / –
ESV00226 (0.05%) 1.0 0.42 N / –
ESV00317 (0.04%) 1.0 0.42 N / –
ESV00367 (0.03%) 1.0 0.42 N / –
ESV00698 (0.05%) 1.0 0.42 N / –
ESV00839 (0.01%) 1.0 0.42 N / –
ESV00876 (0.01%) 1.0 0.42 N / –
ESV00937 (0.02%) 0.7 0.52 – / –
ESV00997 (0.02%) 1.0 0.42 N / –
ESV02221 (0.004%) 1.0 0.42 N / –
ESV03034 (0.002%) 1.0 0.42 N / –
s:Mycena_abramsii (0.02%) 0.5 0.61 – / –
ESV00617 (0.02%) 0.5 0.61 – / –
s:Mycena_albidoaquosa (0.03%) 1.0 0.42 N / –
ESV00440 (0.03%) 1.0 0.42 N / –
s:Mycena_maurella (0.03%) 1.0 0.42 N / –
ESV00488 (0.03%) 1.0 0.42 N / –
s:Mycena_sinar_var._tangkaisinar (0.06%) 0.8 0.50 – / –
ESV00300 (0.05%) 1.0 0.42 N / –
ESV02112 (0.01%) 1.0 0.42 N / –
g:Mycenella (0.02%) 1.0 0.42 N / –
s:Mycenella_trachyspora (0.02%) 1.0 0.42 N / –
ESV01099 (0.02%) 1.0 0.42 N / –
g:Ripartites (0.03%) 1.0 0.42 N / –
s:Ripartites_tricholoma (0.03%) 1.0 0.42 N / –
ESV00561 (0.03%) 1.0 0.42 N / –
g:Tricholomopsis (0.04%) 2.0 0.22 N / –
g:Tricholomopsis_uncl (0.04%) 2.0 0.22 N / –
ESV00381 (0.03%) 1.0 0.42 N / –
ESV01286 (0.009%) 1.0 0.42 N / –
o:Agaricales_uncl (2.23%) 2.4 0.17 N / –
o:Agaricales_uncl (2.23%) 2.4 0.17 N / –
o:Agaricales_uncl (2.23%) 2.4 0.17 N / –
ESV00009 (1.23%) 1.0 0.42 N / –
ESV00109 (0.1%) 0.8 0.50 – / –
ESV00172 (0.07%) 1.0 0.42 N / –

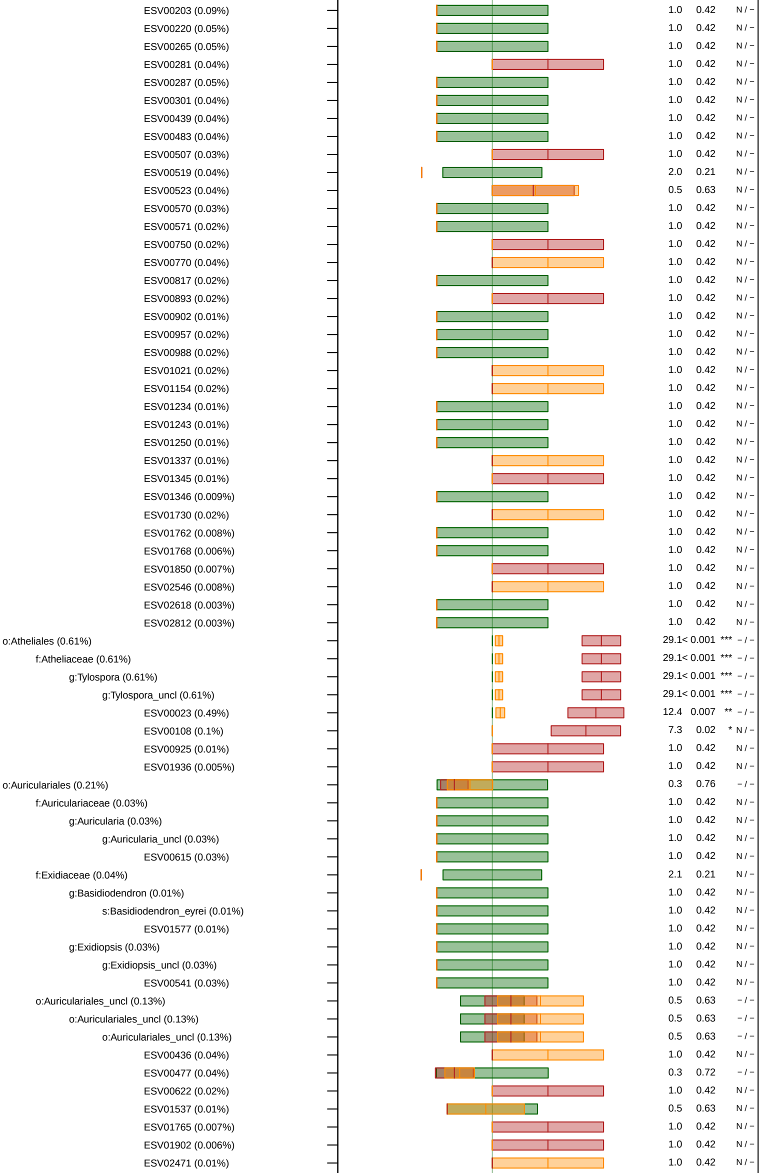
ESV00203 (0.09%) 1.0 0.42 N / –
ESV00220 (0.05%) 1.0 0.42 N / –
ESV00265 (0.05%) 1.0 0.42 N / –
ESV00281 (0.04%) 1.0 0.42 N / –
ESV00287 (0.05%) 1.0 0.42 N / –
ESV00301 (0.04%) 1.0 0.42 N / –
ESV00439 (0.04%) 1.0 0.42 N / –
ESV00483 (0.04%) 1.0 0.42 N / –
ESV00507 (0.03%) 1.0 0.42 N / –
ESV00519 (0.04%) 2.0 0.21 N / –
ESV00523 (0.04%) 0.5 0.63 N / –
ESV00570 (0.03%) 1.0 0.42 N / –
ESV00571 (0.02%) 1.0 0.42 N / –
ESV00750 (0.02%) 1.0 0.42 N / –
ESV00770 (0.04%) 1.0 0.42 N / –
ESV00817 (0.02%) 1.0 0.42 N / –
ESV00893 (0.02%) 1.0 0.42 N / –
ESV00902 (0.01%) 1.0 0.42 N / –
ESV00957 (0.02%) 1.0 0.42 N / –
ESV00988 (0.02%) 1.0 0.42 N / –
ESV01021 (0.02%) 1.0 0.42 N / –
ESV01154 (0.02%) 1.0 0.42 N / –
ESV01234 (0.01%) 1.0 0.42 N / –
ESV01243 (0.01%) 1.0 0.42 N / –
ESV01250 (0.01%) 1.0 0.42 N / –
ESV01337 (0.01%) 1.0 0.42 N / –
ESV01345 (0.01%) 1.0 0.42 N / –
ESV01346 (0.009%) 1.0 0.42 N / –
ESV01730 (0.02%) 1.0 0.42 N / –
ESV01762 (0.008%) 1.0 0.42 N / –
ESV01768 (0.006%) 1.0 0.42 N / –
ESV01850 (0.007%) 1.0 0.42 N / –
ESV02546 (0.008%) 1.0 0.42 N / –
ESV02618 (0.003%) 1.0 0.42 N / –
ESV02812 (0.003%) 1.0 0.42 N / –
o:Atheliales (0.61%) 29.1 < 0.001 *** – / –
f:Atheliaceae (0.61%) 29.1 < 0.001 *** – / –
g:Tylospora (0.61%) 29.1 < 0.001 *** – / –
g:Tylospora_uncl (0.61%) 29.1 < 0.001 *** – / –
ESV00023 (0.49%) 12.4 0.007 ** – / –
ESV00108 (0.1%) 7.3 0.02 * N / –
ESV00925 (0.01%) 1.0 0.42 N / –
ESV01936 (0.005%) 1.0 0.42 N / –
o:Auriculariales (0.21%) 0.3 0.76 – / –
f:Auriculariaceae (0.03%) 1.0 0.42 N / –
g:Auricularia (0.03%) 1.0 0.42 N / –
g:Auricularia_uncl (0.03%) 1.0 0.42 N / –
ESV00615 (0.03%) 1.0 0.42 N / –
f:Exidiaceae (0.04%) 2.1 0.21 N / –
g:Basidiodendron (0.01%) 1.0 0.42 N / –
s:Basidiodendron_eyrei (0.01%) 1.0 0.42 N / –
ESV01577 (0.01%) 1.0 0.42 N / –
g:Exidiopsis (0.03%) 1.0 0.42 N / –
g:Exidiopsis_uncl (0.03%) 1.0 0.42 N / –
ESV00541 (0.03%) 1.0 0.42 N / –
o:Auriculariales_uncl (0.13%) 0.5 0.63 – / –
o:Auriculariales_uncl (0.13%) 0.5 0.63 – / –
o:Auriculariales_uncl (0.13%) 0.5 0.63 – / –
ESV00436 (0.04%) 1.0 0.42 N / –
ESV00477 (0.04%) 0.3 0.72 – / –
ESV00622 (0.02%) 1.0 0.42 N / –
ESV01537 (0.01%) 0.5 0.63 N / –
ESV01765 (0.007%) 1.0 0.42 N / –
ESV01902 (0.006%) 1.0 0.42 N / –
ESV02471 (0.01%) 1.0 0.42 N / –

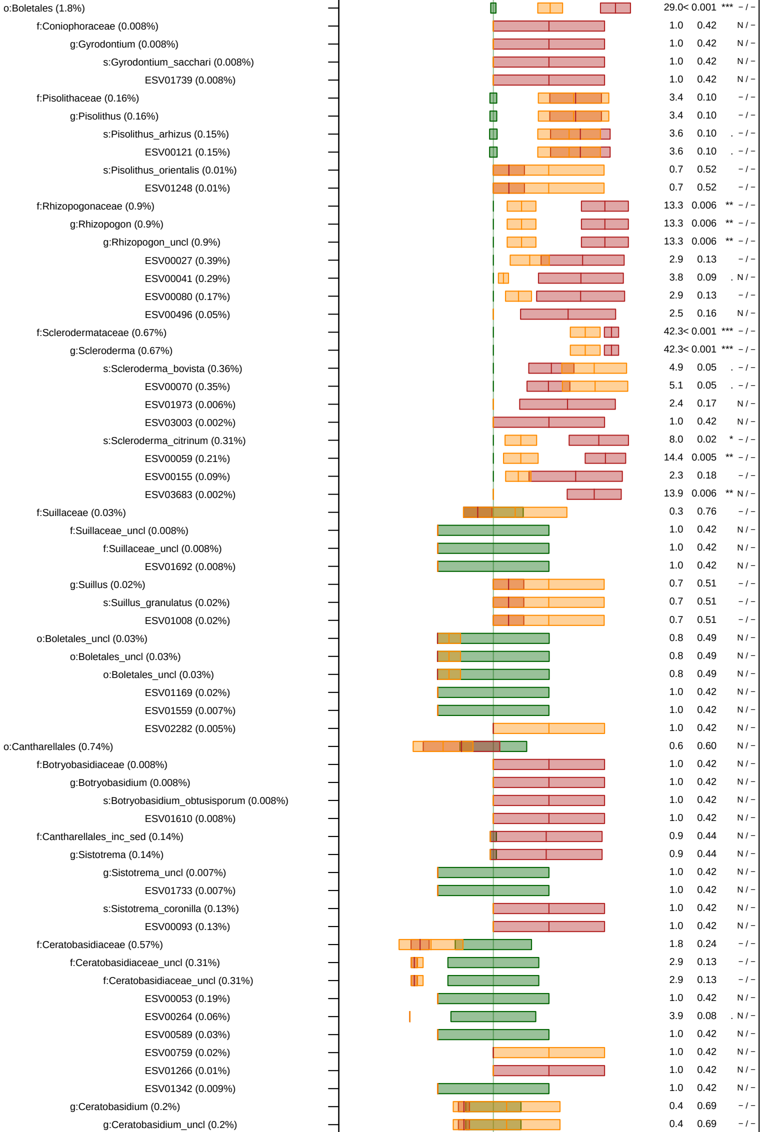

o:Boletales (1.8%) 29.0< 0.001 *** – / –
f:Coniophoraceae (0.008%) 1.0 0.42 N / –
g:Gyrodontium (0.008%) 1.0 0.42 N / –
s:Gyrodontium_sacchari (0.008%) 1.0 0.42 N / –
ESV01739 (0.008%) 1.0 0.42 N / –
f:Pisolithaceae (0.16%) 3.4 0.10 – / –
g:Pisolithus (0.16%) 3.4 0.10 – / –
s:Pisolithus_arhizus (0.15%) 3.6 0.10 . – / –
ESV00121 (0.15%) 3.6 0.10 . – / –
s:Pisolithus_orientalis (0.01%) 0.7 0.52 – / –
ESV01248 (0.01%) 0.7 0.52 – / –
f:Rhizopogonaceae (0.9%) 13.3 0.006 ** – / –
g:Rhizopogon (0.9%) 13.3 0.006 ** – / –
g:Rhizopogon_uncl (0.9%) 13.3 0.006 ** – / –
ESV00027 (0.39%) 2.9 0.13 – / –
ESV00041 (0.29%) 3.8 0.09 . N / –
ESV00080 (0.17%) 2.9 0.13 – / –
ESV00496 (0.05%) 2.5 0.16 N / –
f:Sclerodermataceae (0.67%) 42.3< 0.001 *** – / –
g:Scleroderma (0.67%) 42.3< 0.001 *** – / –
s:Scleroderma_bovista (0.36%) 4.9 0.05 . – / –
ESV00070 (0.35%) 5.1 0.05 . – / –
ESV01973 (0.006%) 2.4 0.17 N / –
ESV03003 (0.002%) 1.0 0.42 N / –
s:Scleroderma_citrinum (0.31%) 8.0 0.02 * – / –
ESV00059 (0.21%) 14.4 0.005 ** – / –
ESV00155 (0.09%) 2.3 0.18 – / –
ESV03683 (0.002%) 13.9 0.006 ** N / –
f:Suillaceae (0.03%) 0.3 0.76 – / –
f:Suillaceae_uncl (0.008%) 1.0 0.42 N / –
f:Suillaceae_uncl (0.008%) 1.0 0.42 N / –
ESV01692 (0.008%) 1.0 0.42 N / –
g:Suillus (0.02%) 0.7 0.51 – / –
s:Suillus_granulatus (0.02%) 0.7 0.51 – / –
ESV01008 (0.02%) 0.7 0.51 – / –
o:Boletales_uncl (0.03%) 0.8 0.49 N / –
o:Boletales_uncl (0.03%) 0.8 0.49 N / –
o:Boletales_uncl (0.03%) 0.8 0.49 N / –
ESV01169 (0.02%) 1.0 0.42 N / –
ESV01559 (0.007%) 1.0 0.42 N / –
ESV02282 (0.005%) 1.0 0.42 N / –
o:Cantharellales (0.74%) 0.6 0.60 N / –
f:Botryobasidiaceae (0.008%) 1.0 0.42 N / –
g:Botryobasidium (0.008%) 1.0 0.42 N / –
s:Botryobasidium_obtusisporum (0.008%) 1.0 0.42 N / –
ESV01610 (0.008%) 1.0 0.42 N / –
f:Cantharellales_inc_sed (0.14%) 0.9 0.44 N / –
g:Sistotrema (0.14%) 0.9 0.44 N / –
g:Sistotrema_uncl (0.007%) 1.0 0.42 N / –
ESV01733 (0.007%) 1.0 0.42 N / –
s:Sistotrema_coronilla (0.13%) 1.0 0.42 N / –
ESV00093 (0.13%) 1.0 0.42 N / –
f:Ceratobasidiaceae (0.57%) 1.8 0.24 – / –
f:Ceratobasidiaceae_uncl (0.31%) 2.9 0.13 – / –
f:Ceratobasidiaceae_uncl (0.31%) 2.9 0.13 – / –
ESV00053 (0.19%) 1.0 0.42 N / –
ESV00264 (0.06%) 3.9 0.08 . N / –
ESV00589 (0.03%) 1.0 0.42 N / –
ESV00759 (0.02%) 1.0 0.42 N / –
ESV01266 (0.01%) 1.0 0.42 N / –
ESV01342 (0.009%) 1.0 0.42 N / –
g:Ceratobasidium (0.2%) 0.4 0.69 – / –
g:Ceratobasidium_uncl (0.2%) 0.4 0.69 – / –

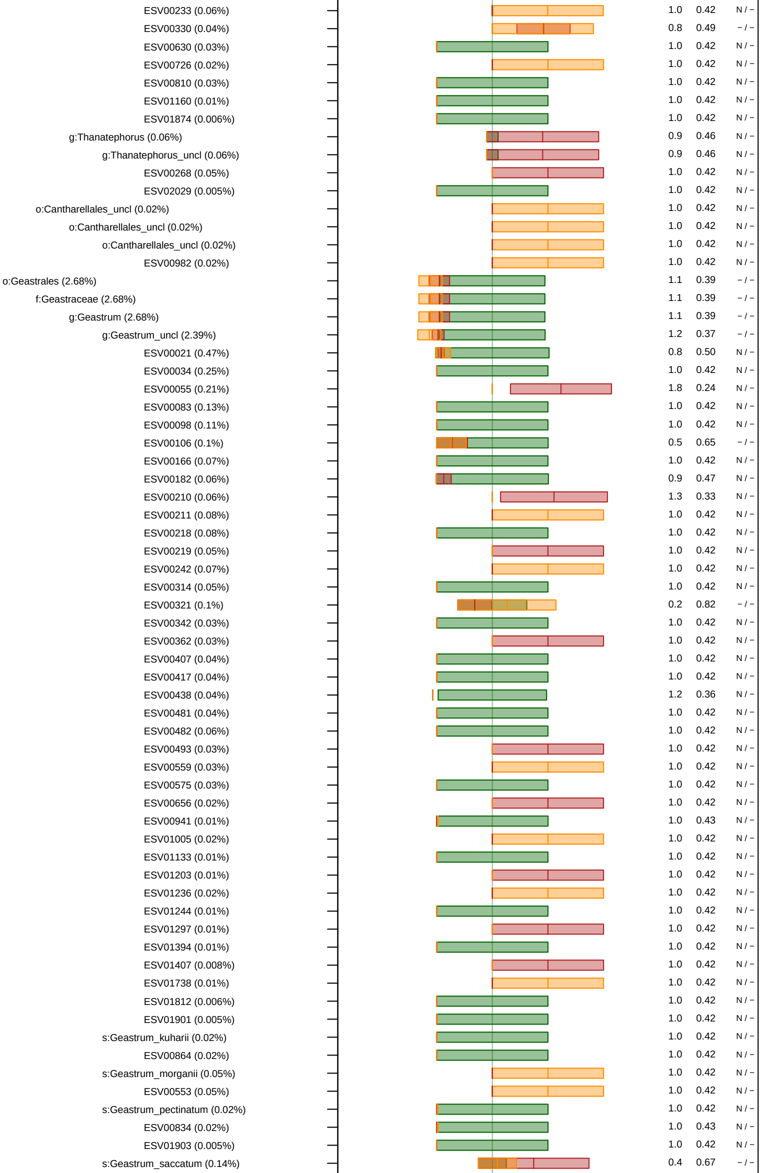
ESV00233 (0.06%) 1.0 0.42 N / –
ESV00330 (0.04%) 0.8 0.49 – / –
ESV00630 (0.03%) 1.0 0.42 N / –
ESV00726 (0.02%) 1.0 0.42 N / –
ESV00810 (0.03%) 1.0 0.42 N / –
ESV01160 (0.01%) 1.0 0.42 N / –
ESV01874 (0.006%) 1.0 0.42 N / –
g:Thanatephorus (0.06%) 0.9 0.46 N / –
g:Thanatephorus_uncl (0.06%) 0.9 0.46 N / –
ESV00268 (0.05%) 1.0 0.42 N / –
ESV02029 (0.005%) 1.0 0.42 N / –
o:Cantharellales_uncl (0.02%) 1.0 0.42 N / –
o:Cantharellales_uncl (0.02%) 1.0 0.42 N / –
o:Cantharellales_uncl (0.02%) 1.0 0.42 N / –
ESV00982 (0.02%) 1.0 0.42 N / –
o:Geastrales (2.68%) 1.1 0.39 – / –
f:Geastraceae (2.68%) 1.1 0.39 – / –
g:Geastrum (2.68%) 1.1 0.39 – / –
g:Geastrum_uncl (2.39%) 1.2 0.37 – / –
ESV00021 (0.47%) 0.8 0.50 N / –
ESV00034 (0.25%) 1.0 0.42 N / –
ESV00055 (0.21%) 1.8 0.24 N / –
ESV00083 (0.13%) 1.0 0.42 N / –
ESV00098 (0.11%) 1.0 0.42 N / –
ESV00106 (0.1%) 0.5 0.65 – / –
ESV00166 (0.07%) 1.0 0.42 N / –
ESV00182 (0.06%) 0.9 0.47 N / –
ESV00210 (0.06%) 1.3 0.33 N / –
ESV00211 (0.08%) 1.0 0.42 N / –
ESV00218 (0.08%) 1.0 0.42 N / –
ESV00219 (0.05%) 1.0 0.42 N / –
ESV00242 (0.07%) 1.0 0.42 N / –
ESV00314 (0.05%) 1.0 0.42 N / –
ESV00321 (0.1%) 0.2 0.82 – / –
ESV00342 (0.03%) 1.0 0.42 N / –
ESV00362 (0.03%) 1.0 0.42 N / –
ESV00407 (0.04%) 1.0 0.42 N / –
ESV00417 (0.04%) 1.0 0.42 N / –
ESV00438 (0.04%) 1.2 0.36 N / –
ESV00481 (0.04%) 1.0 0.42 N / –
ESV00482 (0.06%) 1.0 0.42 N / –
ESV00493 (0.03%) 1.0 0.42 N / –
ESV00559 (0.03%) 1.0 0.42 N / –
ESV00575 (0.03%) 1.0 0.42 N / –
ESV00656 (0.02%) 1.0 0.42 N / –
ESV00941 (0.01%) 1.0 0.43 N / –
ESV01005 (0.02%) 1.0 0.42 N / –
ESV01133 (0.01%) 1.0 0.42 N / –
ESV01203 (0.01%) 1.0 0.42 N / –
ESV01236 (0.02%) 1.0 0.42 N / –
ESV01244 (0.01%) 1.0 0.42 N / –
ESV01297 (0.01%) 1.0 0.42 N / –
ESV01394 (0.01%) 1.0 0.42 N / –
ESV01407 (0.008%) 1.0 0.42 N / –
ESV01738 (0.01%) 1.0 0.42 N / –
ESV01812 (0.006%) 1.0 0.42 N / –
ESV01901 (0.005%) 1.0 0.42 N / –
s:Geastrum_kuharii (0.02%) 1.0 0.42 N / –
ESV00864 (0.02%) 1.0 0.42 N / –
s:Geastrum_morganii (0.05%) 1.0 0.42 N / –
ESV00553 (0.05%) 1.0 0.42 N / –
s:Geastrum_pectinatum (0.02%) 1.0 0.42 N / –
ESV00834 (0.02%) 1.0 0.43 N / –
ESV01903 (0.005%) 1.0 0.42 N / –
s:Geastrum_saccatum (0.14%) 0.4 0.67 – / –

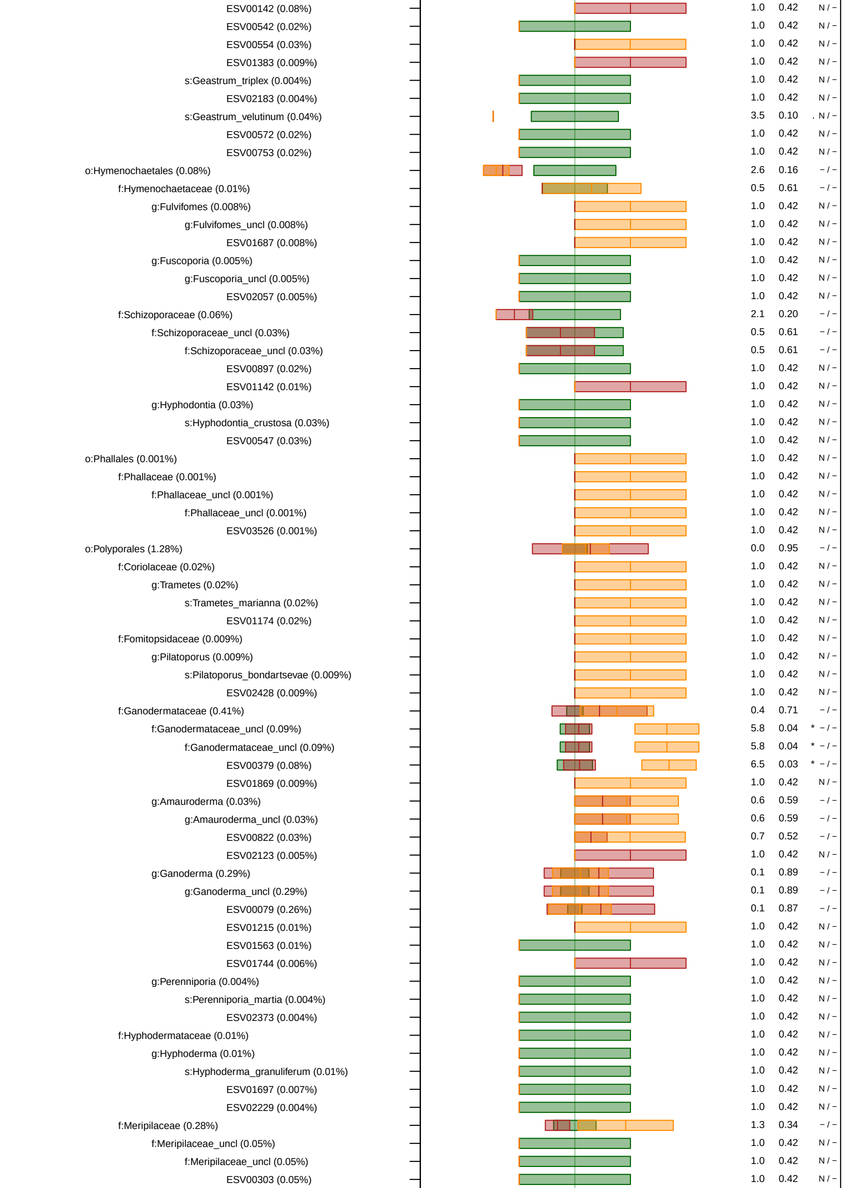

ESV00142 (0.08%) 1.0 0.42 N / –
ESV00542 (0.02%) 1.0 0.42 N / –
ESV00554 (0.03%) 1.0 0.42 N / –
ESV01383 (0.009%) 1.0 0.42 N / –
s:Geastrum_triplex (0.004%) 1.0 0.42 N / –
ESV02183 (0.004%) 1.0 0.42 N / –
s:Geastrum_velutinum (0.04%) 3.5 0.10 . N / –
ESV00572 (0.02%) 1.0 0.42 N / –
ESV00753 (0.02%) 1.0 0.42 N / –
o:Hymenochaetales (0.08%) 2.6 0.16 – / –
f:Hymenochaetaceae (0.01%) 0.5 0.61 – / –
g:Fulvifomes (0.008%) 1.0 0.42 N / –
g:Fulvifomes_uncl (0.008%) 1.0 0.42 N / –
ESV01687 (0.008%) 1.0 0.42 N / –
g:Fuscoporia (0.005%) 1.0 0.42 N / –
g:Fuscoporia_uncl (0.005%) 1.0 0.42 N / –
ESV02057 (0.005%) 1.0 0.42 N / –
f:Schizoporaceae (0.06%) 2.1 0.20 – / –
f:Schizoporaceae_uncl (0.03%) 0.5 0.61 – / –
f:Schizoporaceae_uncl (0.03%) 0.5 0.61 – / –
ESV00897 (0.02%) 1.0 0.42 N / –
ESV01142 (0.01%) 1.0 0.42 N / –
g:Hyphodontia (0.03%) 1.0 0.42 N / –
s:Hyphodontia_crustosa (0.03%) 1.0 0.42 N / –
ESV00547 (0.03%) 1.0 0.42 N / –
o:Phallales (0.001%) 1.0 0.42 N / –
f:Phallaceae (0.001%) 1.0 0.42 N / –
f:Phallaceae_uncl (0.001%) 1.0 0.42 N / –
f:Phallaceae_uncl (0.001%) 1.0 0.42 N / –
ESV03526 (0.001%) 1.0 0.42 N / –
o:Polyporales (1.28%) 0.0 0.95 – / –
f:Coriolaceae (0.02%) 1.0 0.42 N / –
g:Trametes (0.02%) 1.0 0.42 N / –
s:Trametes_marianna (0.02%) 1.0 0.42 N / –
ESV01174 (0.02%) 1.0 0.42 N / –
f:Fomitopsidaceae (0.009%) 1.0 0.42 N / –
g:Pilatoporus (0.009%) 1.0 0.42 N / –
s:Pilatoporus_bondartsevae (0.009%) 1.0 0.42 N / –
ESV02428 (0.009%) 1.0 0.42 N / –
f:Ganodermataceae (0.41%) 0.4 0.71 – / –
f:Ganodermataceae_uncl (0.09%) 5.8 0.04 * – / –
f:Ganodermataceae_uncl (0.09%) 5.8 0.04 * – / –
ESV00379 (0.08%) 6.5 0.03 * – / –
ESV01869 (0.009%) 1.0 0.42 N / –
g:Amauroderma (0.03%) 0.6 0.59 – / –
g:Amauroderma_uncl (0.03%) 0.6 0.59 – / –
ESV00822 (0.03%) 0.7 0.52 – / –
ESV02123 (0.005%) 1.0 0.42 N / –
g:Ganoderma (0.29%) 0.1 0.89 – / –
g:Ganoderma_uncl (0.29%) 0.1 0.89 – / –
ESV00079 (0.26%) 0.1 0.87 – / –
ESV01215 (0.01%) 1.0 0.42 N / –
ESV01563 (0.01%) 1.0 0.42 N / –
ESV01744 (0.006%) 1.0 0.42 N / –
g:Perenniporia (0.004%) 1.0 0.42 N / –
s:Perenniporia_martia (0.004%) 1.0 0.42 N / –
ESV02373 (0.004%) 1.0 0.42 N / –
f:Hyphodermataceae (0.01%) 1.0 0.42 N / –
g:Hyphoderma (0.01%) 1.0 0.42 N / –
s:Hyphoderma_granuliferum (0.01%) 1.0 0.42 N / –
ESV01697 (0.007%) 1.0 0.42 N / –
ESV02229 (0.004%) 1.0 0.42 N / –
f:Meripilaceae (0.28%) 1.3 0.34 – / –
f:Meripilaceae_uncl (0.05%) 1.0 0.42 N / –
f:Meripilaceae_uncl (0.05%) 1.0 0.42 N / –
ESV00303 (0.05%) 1.0 0.42 N / –

g:Meripilus (0.15%)	3.3	0.11		– / –
s:Meripilus_giganteus (0.15%)	3.3	0.11		– / –
ESV00271 (0.09%)	0.9	0.47		N / –
ESV00403 (0.04%)	1.0	0.42		N / –
ESV00831 (0.03%)	3.2	0.11		N / –
g:Rigidoporus (0.08%)	0.0	0.99		– / –
g:Rigidoporus_uncl (0.01%)	1.0	0.42		N / –
ESV01007 (0.01%)	1.0	0.42		N / –
s:Rigidoporus_microporus (0.01%)	1.0	0.42		N / –
ESV01013 (0.01%)	1.0	0.42		N / –
s:Rigidoporus_sanguinolentus (0.04%)	0.1	0.91		N / –
ESV00826 (0.01%)	1.0	0.42		N / –
ESV00966 (0.01%)	1.0	0.42		N / –
ESV01974 (0.008%)	1.0	0.42		N / –
s:Rigidoporus_ulmarius (0.02%)	1.0	0.42		N / –
ESV01722 (0.02%)	1.0	0.42		N / –
f:Meruliaceae (0.14%)	1.0	0.42		– / –
g:Bjerkandera (0.04%)	1.4	0.31		– / –
g:Bjerkandera_uncl (0.04%)	1.4	0.31		– / –
ESV00562 (0.02%)	1.0	0.41		N / –
ESV01371 (0.009%)	1.0	0.42		N / –
ESV02109 (0.01%)	1.0	0.42		N / –
g:Candelabrochaete (0.003%)	1.0	0.42		N / –
g:Candelabrochaete_uncl (0.003%)	1.0	0.42		N / –
ESV03019 (0.003%)	1.0	0.42		N / –
g:Ceriporia (0.008%)	1.0	0.42		N / –
g:Ceriporia_uncl (0.008%)	1.0	0.42		N / –
ESV01436 (0.008%)	1.0	0.42		N / –
g:Meruliopsis (0.01%)	1.0	0.42		N / –
g:Meruliopsis_uncl (0.01%)	1.0	0.42		N / –
ESV01209 (0.01%)	1.0	0.42		N / –
g:Phlebia (0.04%)	1.0	0.42		N / –
g:Phlebia_uncl (0.04%)	1.0	0.42		N / –
ESV00351 (0.04%)	1.0	0.42		N / –
g:Scopuloides (0.03%)	1.0	0.42		N / –
g:Scopuloides_uncl (0.03%)	1.0	0.42		N / –
ESV00762 (0.02%)	1.0	0.42		N / –
ESV01066 (0.01%)	1.0	0.42		N / –
f:Polyporaceae (0.25%)	0.9	0.44		N / –
g:Daedaleopsis (0.23%)	1.0	0.42		N / –
s:Daedaleopsis_confragosa (0.23%)	1.0	0.42		N / –
ESV00045 (0.23%)	1.0	0.42		N / –
g:Polyporus (0.02%)	1.0	0.42		N / –
s:Polyporus_dictyopus (0.02%)	1.0	0.42		N / –
ESV00942 (0.02%)	1.0	0.42		N / –
f:Xenasmataceae (0.06%)	0.6	0.59		– / –
g:Xenasmatella (0.06%)	0.6	0.59		– / –
g:Xenasmatella_uncl (0.06%)	0.6	0.59		– / –
ESV00478 (0.04%)	1.0	0.42		N / –
ESV00862 (0.02%)	1.0	0.42		N / –
o:Polyporales_uncl (0.12%)	0.2	0.80		– / –
o:Polyporales_uncl (0.12%)	0.2	0.80		– / –
o:Polyporales_uncl (0.12%)	0.2	0.80		– / –
ESV00370 (0.04%)	1.0	0.42		N / –
ESV00528 (0.03%)	1.0	0.42		N / –
ESV00754 (0.03%)	1.0	0.42		N / –
ESV00979 (0.02%)	1.0	0.42		N / –
ESV01468 (0.009%)	1.0	0.42		N / –
ESV03408 (0.003%)	1.0	0.42		N / –
o:Russulales (15.63%)	17.3	0.003	**	– / –
f:Gloeocystidiellaceae (0.02%)	1.0	0.42		N / –
g:Laxitextum (0.02%)	1.0	0.42		N / –
s:Laxitextum_incrustatum (0.02%)	1.0	0.42		N / –
ESV00794 (0.02%)	1.0	0.42		N / –
f:Lachnocladiaceae (0.05%)	1.0	0.42		N / –

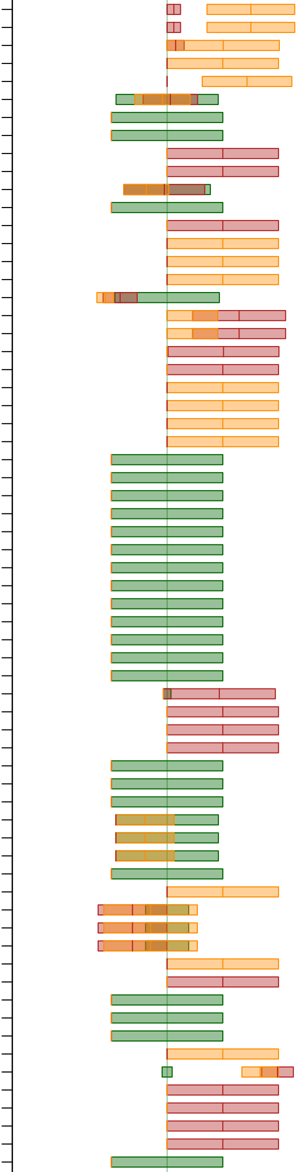

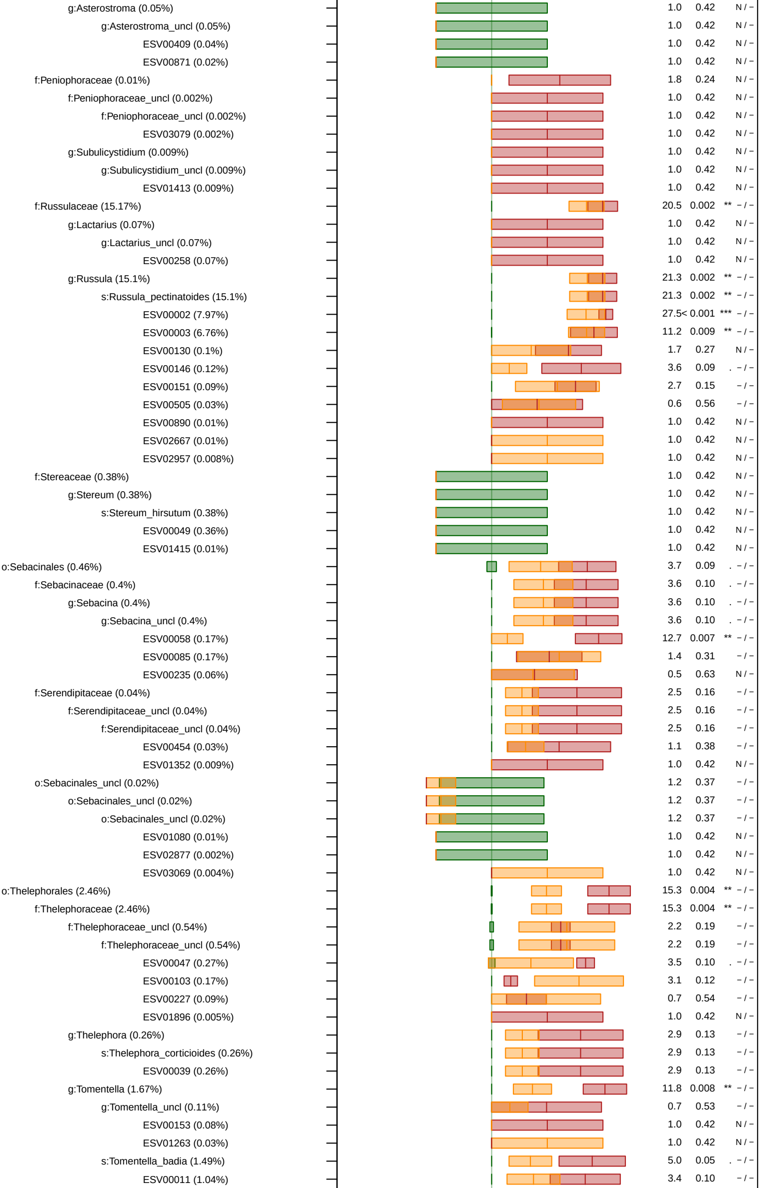

g:Asterostroma (0.05%) 1.0 0.42 N / –
g:Asterostroma_uncl (0.05%) 1.0 0.42 N / –
ESV00409 (0.04%) 1.0 0.42 N / –
ESV00871 (0.02%) 1.0 0.42 N / –
f:Peniophoraceae (0.01%) 1.8 0.24 N / –
f:Peniophoraceae_uncl (0.002%) 1.0 0.42 N / –
f:Peniophoraceae_uncl (0.002%) 1.0 0.42 N / –
ESV03079 (0.002%) 1.0 0.42 N / –
g:Subulicystidium (0.009%) 1.0 0.42 N / –
g:Subulicystidium_uncl (0.009%) 1.0 0.42 N / –
ESV01413 (0.009%) 1.0 0.42 N / –
f:Russulaceae (15.17%) 20.5 0.002 ** – / –
g:Lactarius (0.07%) 1.0 0.42 N / –
g:Lactarius_uncl (0.07%) 1.0 0.42 N / –
ESV00258 (0.07%) 1.0 0.42 N / –
g:Russula (15.1%) 21.3 0.002 ** – / –
s:Russula_pectinatoides (15.1%) 21.3 0.002 ** – / –
ESV00002 (7.97%) 27.5 < 0.001 *** – / –
ESV00003 (6.76%) 11.2 0.009 ** – / –
ESV00130 (0.1%) 1.7 0.27 N / –
ESV00146 (0.12%) 3.6 0.09 . – / –
ESV00151 (0.09%) 2.7 0.15 – / –
ESV00505 (0.03%) 0.6 0.56 – / –
ESV00890 (0.01%) 1.0 0.42 N / –
ESV02667 (0.01%) 1.0 0.42 N / –
ESV02957 (0.008%) 1.0 0.42 N / –
f:Stereaceae (0.38%) 1.0 0.42 N / –
g:Stereum (0.38%) 1.0 0.42 N / –
s:Stereum_hirsutum (0.38%) 1.0 0.42 N / –
ESV00049 (0.36%) 1.0 0.42 N / –
ESV01415 (0.01%) 1.0 0.42 N / –
o:Sebacinales (0.46%) 3.7 0.09 . – / –
f:Sebacinaceae (0.4%) 3.6 0.10 . – / –
g:Sebacina (0.4%) 3.6 0.10 . – / –
g:Sebacina_uncl (0.4%) 3.6 0.10 . – / –
ESV00058 (0.17%) 12.7 0.007 ** – / –
ESV00085 (0.17%) 1.4 0.31 – / –
ESV00235 (0.06%) 0.5 0.63 N / –
f:Serendipitaceae (0.04%) 2.5 0.16 – / –
f:Serendipitaceae_uncl (0.04%) 2.5 0.16 – / –
f:Serendipitaceae_uncl (0.04%) 2.5 0.16 – / –
ESV00454 (0.03%) 1.1 0.38 – / –
ESV01352 (0.009%) 1.0 0.42 N / –
o:Sebacinales_uncl (0.02%) 1.2 0.37 – / –
o:Sebacinales_uncl (0.02%) 1.2 0.37 – / –
o:Sebacinales_uncl (0.02%) 1.2 0.37 – / –
ESV01080 (0.01%) 1.0 0.42 N / –
ESV02877 (0.002%) 1.0 0.42 N / –
ESV03069 (0.004%) 1.0 0.42 N / –
o:Thelephorales (2.46%) 15.3 0.004 ** – / –
f:Thelephoraceae (2.46%) 15.3 0.004 ** – / –
f:Thelephoraceae_uncl (0.54%) 2.2 0.19 – / –
f:Thelephoraceae_uncl (0.54%) 2.2 0.19 – / –
ESV00047 (0.27%) 3.5 0.10 . – / –
ESV00103 (0.17%) 3.1 0.12 – / –
ESV00227 (0.09%) 0.7 0.54 – / –
ESV01896 (0.005%) 1.0 0.42 N / –
g:Thelephora (0.26%) 2.9 0.13 – / –
s:Thelephora_corticioides (0.26%) 2.9 0.13 – / –
ESV00039 (0.26%) 2.9 0.13 – / –
g:Tomentella (1.67%) 11.8 0.008 ** – / –
g:Tomentella_uncl (0.11%) 0.7 0.53 – / –
ESV00153 (0.08%) 1.0 0.42 N / –
ESV01263 (0.03%) 1.0 0.42 N / –
s:Tomentella_badia (1.49%) 5.0 0.05 . – / –
ESV00011 (1.04%) 3.4 0.10 – / –

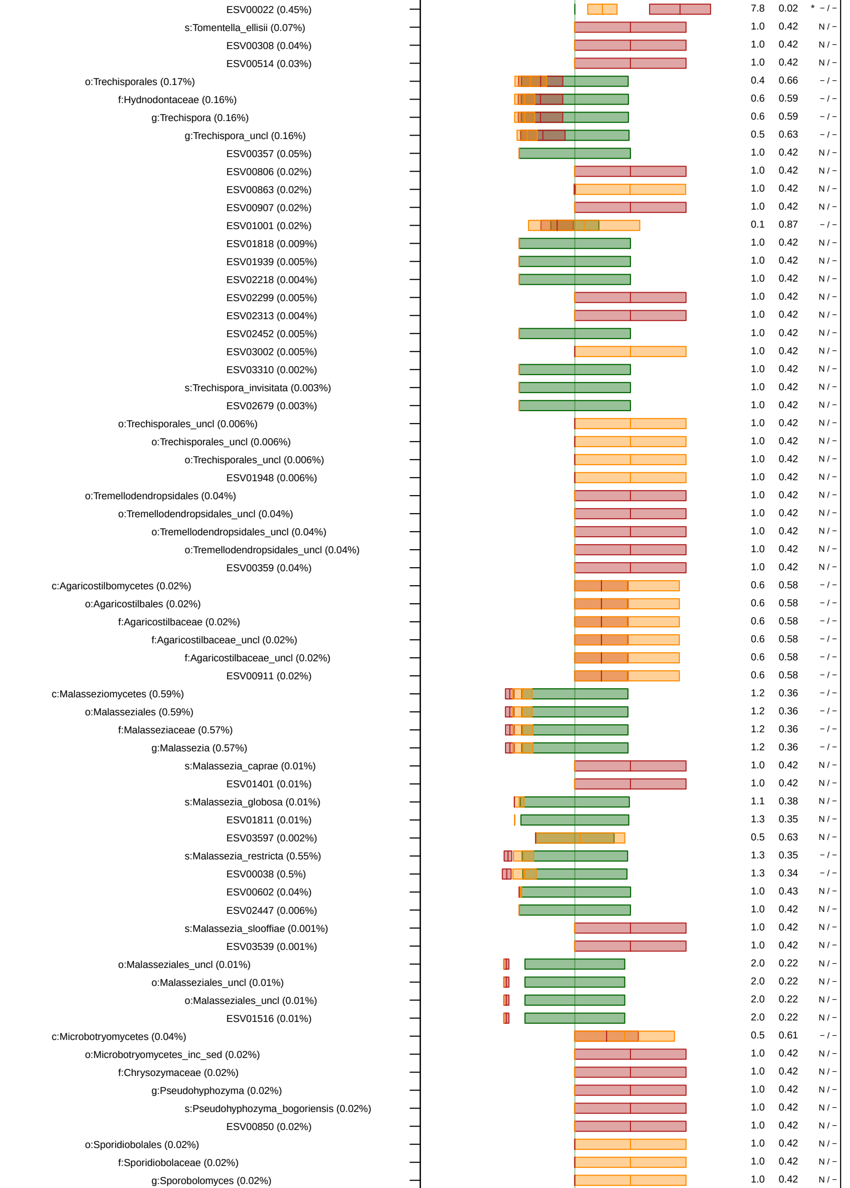
ESV00022 (0.45%) 7.8 0.02 * – / –
s:Tomentella_ellisii (0.07%) 1.0 0.42 N / –
ESV00308 (0.04%) 1.0 0.42 N / –
ESV00514 (0.03%) 1.0 0.42 N / –
o:Trechisporales (0.17%) 0.4 0.66 – / –
f:Hydnodontaceae (0.16%) 0.6 0.59 – / –
g:Trechispora (0.16%) 0.6 0.59 – / –
g:Trechispora_uncl (0.16%) 0.5 0.63 – / –
ESV00357 (0.05%) 1.0 0.42 N / –
ESV00806 (0.02%) 1.0 0.42 N / –
ESV00863 (0.02%) 1.0 0.42 N / –
ESV00907 (0.02%) 1.0 0.42 N / –
ESV01001 (0.02%) 0.1 0.87 – / –
ESV01818 (0.009%) 1.0 0.42 N / –
ESV01939 (0.005%) 1.0 0.42 N / –
ESV02218 (0.004%) 1.0 0.42 N / –
ESV02299 (0.005%) 1.0 0.42 N / –
ESV02313 (0.004%) 1.0 0.42 N / –
ESV02452 (0.005%) 1.0 0.42 N / –
ESV03002 (0.005%) 1.0 0.42 N / –
ESV03310 (0.002%) 1.0 0.42 N / –
s:Trechispora_invisitata (0.003%) 1.0 0.42 N / –
ESV02679 (0.003%) 1.0 0.42 N / –
o:Trechisporales_uncl (0.006%) 1.0 0.42 N / –
o:Trechisporales_uncl (0.006%) 1.0 0.42 N / –
o:Trechisporales_uncl (0.006%) 1.0 0.42 N / –
ESV01948 (0.006%) 1.0 0.42 N / –
o:Tremellodendropsidales (0.04%) 1.0 0.42 N / –
o:Tremellodendropsidales_uncl (0.04%) 1.0 0.42 N / –
o:Tremellodendropsidales_uncl (0.04%) 1.0 0.42 N / –
o:Tremellodendropsidales_uncl (0.04%) 1.0 0.42 N / –
ESV00359 (0.04%) 1.0 0.42 N / –
c:Agaricostilbomycetes (0.02%) 0.6 0.58 – / –
o:Agaricostilbales (0.02%) 0.6 0.58 – / –
f:Agaricostilbaceae (0.02%) 0.6 0.58 – / –
f:Agaricostilbaceae_uncl (0.02%) 0.6 0.58 – / –
f:Agaricostilbaceae_uncl (0.02%) 0.6 0.58 – / –
ESV00911 (0.02%) 0.6 0.58 – / –
c:Malasseziomycetes (0.59%) 1.2 0.36 – / –
o:Malasseziales (0.59%) 1.2 0.36 – / –
f:Malasseziaceae (0.57%) 1.2 0.36 – / –
g:Malassezia (0.57%) 1.2 0.36 – / –
s:Malassezia_caprae (0.01%) 1.0 0.42 N / –
ESV01401 (0.01%) 1.0 0.42 N / –
s:Malassezia_globosa (0.01%) 1.1 0.38 N / –
ESV01811 (0.01%) 1.3 0.35 N / –
ESV03597 (0.002%) 0.5 0.63 N / –
s:Malassezia_restricta (0.55%) 1.3 0.35 – / –
ESV00038 (0.5%) 1.3 0.34 – / –
ESV00602 (0.04%) 1.0 0.43 N / –
ESV02447 (0.006%) 1.0 0.42 N / –
s:Malassezia_slooffiae (0.001%) 1.0 0.42 N / –
ESV03539 (0.001%) 1.0 0.42 N / –
o:Malasseziales_uncl (0.01%) 2.0 0.22 N / –
o:Malasseziales_uncl (0.01%) 2.0 0.22 N / –
o:Malasseziales_uncl (0.01%) 2.0 0.22 N / –
ESV01516 (0.01%) 2.0 0.22 N / –
c:Microbotryomycetes (0.04%) 0.5 0.61 – / –
o:Microbotryomycetes_inc_sed (0.02%) 1.0 0.42 N / –
f:Chrysozymaceae (0.02%) 1.0 0.42 N / –
g:Pseudohyphozyma (0.02%) 1.0 0.42 N / –
s:Pseudohyphozyma_bogoriensis (0.02%) 1.0 0.42 N / –
ESV00850 (0.02%) 1.0 0.42 N / –
o:Sporidiobolales (0.02%) 1.0 0.42 N / –
f:Sporidiobolaceae (0.02%) 1.0 0.42 N / –
g:Sporobolomyces (0.02%) 1.0 0.42 N / –

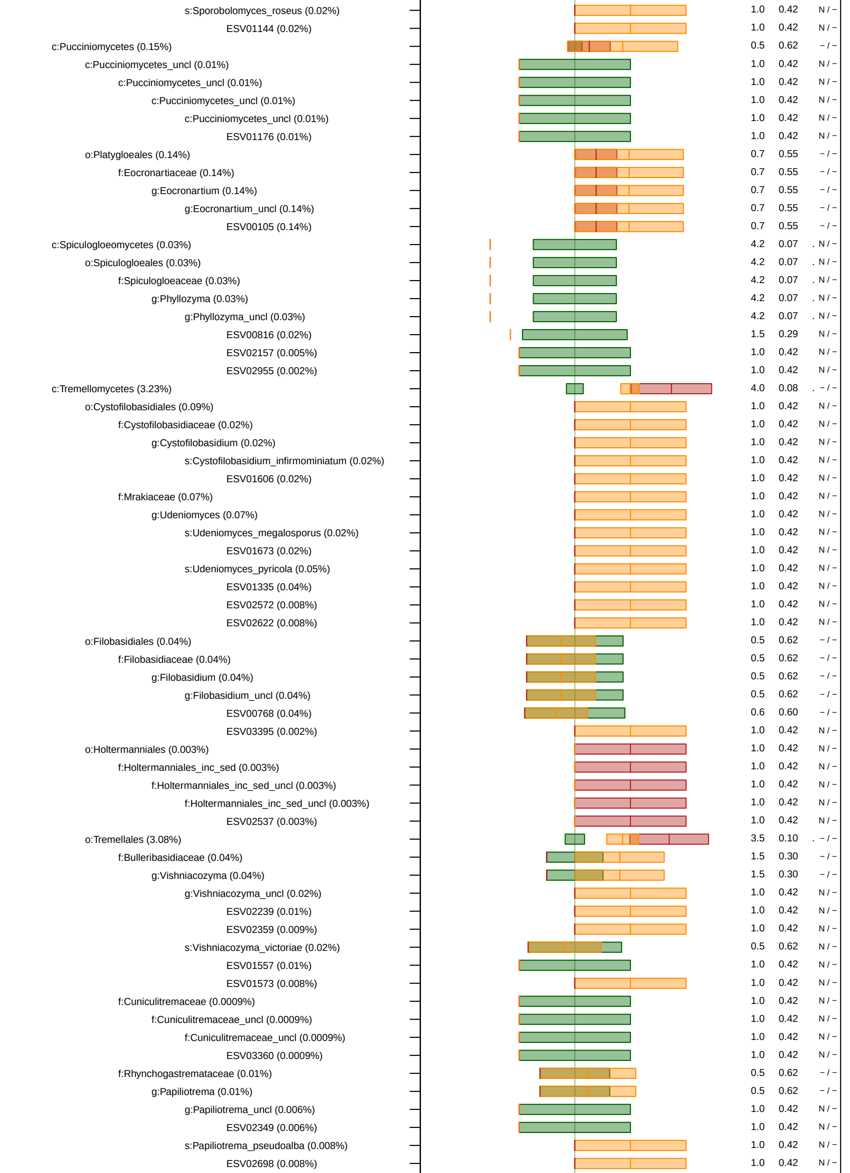
s:Sporobolomyces_roseus (0.02%) 1.0 0.42 N / –
ESV01144 (0.02%) 1.0 0.42 N / –
c:Pucciniomycetes (0.15%) 0.5 0.62 – / –
c:Pucciniomycetes_uncl (0.01%) 1.0 0.42 N / –
c:Pucciniomycetes_uncl (0.01%) 1.0 0.42 N / –
c:Pucciniomycetes_uncl (0.01%) 1.0 0.42 N / –
c:Pucciniomycetes_uncl (0.01%) 1.0 0.42 N / –
ESV01176 (0.01%) 1.0 0.42 N / –
o:Platygloeales (0.14%) 0.7 0.55 – / –
f:Eocronartiaceae (0.14%) 0.7 0.55 – / –
g:Eocronartium (0.14%) 0.7 0.55 – / –
g:Eocronartium_uncl (0.14%) 0.7 0.55 – / –
ESV00105 (0.14%) 0.7 0.55 – / –
c:Spiculogloeomycetes (0.03%) 4.2 0.07 . N / –
o:Spiculogloeales (0.03%) 4.2 0.07 . N / –
f:Spiculogloeaceae (0.03%) 4.2 0.07 . N / –
g:Phyllozyma (0.03%) 4.2 0.07 . N / –
g:Phyllozyma_uncl (0.03%) 4.2 0.07 . N / –
ESV00816 (0.02%) 1.5 0.29 N / –
ESV02157 (0.005%) 1.0 0.42 N / –
ESV02955 (0.002%) 1.0 0.42 N / –
c:Tremellomycetes (3.23%) 4.0 0.08 . – / –
o:Cystofilobasidiales (0.09%) 1.0 0.42 N / –
f:Cystofilobasidiaceae (0.02%) 1.0 0.42 N / –
g:Cystofilobasidium (0.02%) 1.0 0.42 N / –
s:Cystofilobasidium_infirmominiatum (0.02%) 1.0 0.42 N / –
ESV01606 (0.02%) 1.0 0.42 N / –
f:Mrakiaceae (0.07%) 1.0 0.42 N / –
g:Udeniomyces (0.07%) 1.0 0.42 N / –
s:Udeniomyces_megalosporus (0.02%) 1.0 0.42 N / –
ESV01673 (0.02%) 1.0 0.42 N / –
s:Udeniomyces_pyricola (0.05%) 1.0 0.42 N / –
ESV01335 (0.04%) 1.0 0.42 N / –
ESV02572 (0.008%) 1.0 0.42 N / –
ESV02622 (0.008%) 1.0 0.42 N / –
o:Filobasidiales (0.04%) 0.5 0.62 – / –
f:Filobasidiaceae (0.04%) 0.5 0.62 – / –
g:Filobasidium (0.04%) 0.5 0.62 – / –
g:Filobasidium_uncl (0.04%) 0.5 0.62 – / –
ESV00768 (0.04%) 0.6 0.60 – / –
ESV03395 (0.002%) 1.0 0.42 N / –
o:Holtermanniales (0.003%) 1.0 0.42 N / –
f:Holtermanniales_inc_sed (0.003%) 1.0 0.42 N / –
f:Holtermanniales_inc_sed_uncl (0.003%) 1.0 0.42 N / –
f:Holtermanniales_inc_sed_uncl (0.003%) 1.0 0.42 N / –
ESV02537 (0.003%) 1.0 0.42 N / –
o:Tremellales (3.08%) 3.5 0.10 . – / –
f:Bulleribasidiaceae (0.04%) 1.5 0.30 – / –
g:Vishniacozyma (0.04%) 1.5 0.30 – / –
g:Vishniacozyma_uncl (0.02%) 1.0 0.42 N / –
ESV02239 (0.01%) 1.0 0.42 N / –
ESV02359 (0.009%) 1.0 0.42 N / –
s:Vishniacozyma_victoriae (0.02%) 0.5 0.62 N / –
ESV01557 (0.01%) 1.0 0.42 N / –
ESV01573 (0.008%) 1.0 0.42 N / –
f:Cuniculitremaceae (0.0009%) 1.0 0.42 N / –
f:Cuniculitremaceae_uncl (0.0009%) 1.0 0.42 N / –
f:Cuniculitremaceae_uncl (0.0009%) 1.0 0.42 N / –
ESV03360 (0.0009%) 1.0 0.42 N / –
f:Rhynchogastremataceae (0.01%) 0.5 0.62 – / –
g:Papiliotrema (0.01%) 0.5 0.62 – / –
g:Papiliotrema_uncl (0.006%) 1.0 0.42 N / –
ESV02349 (0.006%) 1.0 0.42 N / –
s:Papiliotrema_pseudoalba (0.008%) 1.0 0.42 N / –
ESV02698 (0.008%) 1.0 0.42 N / –

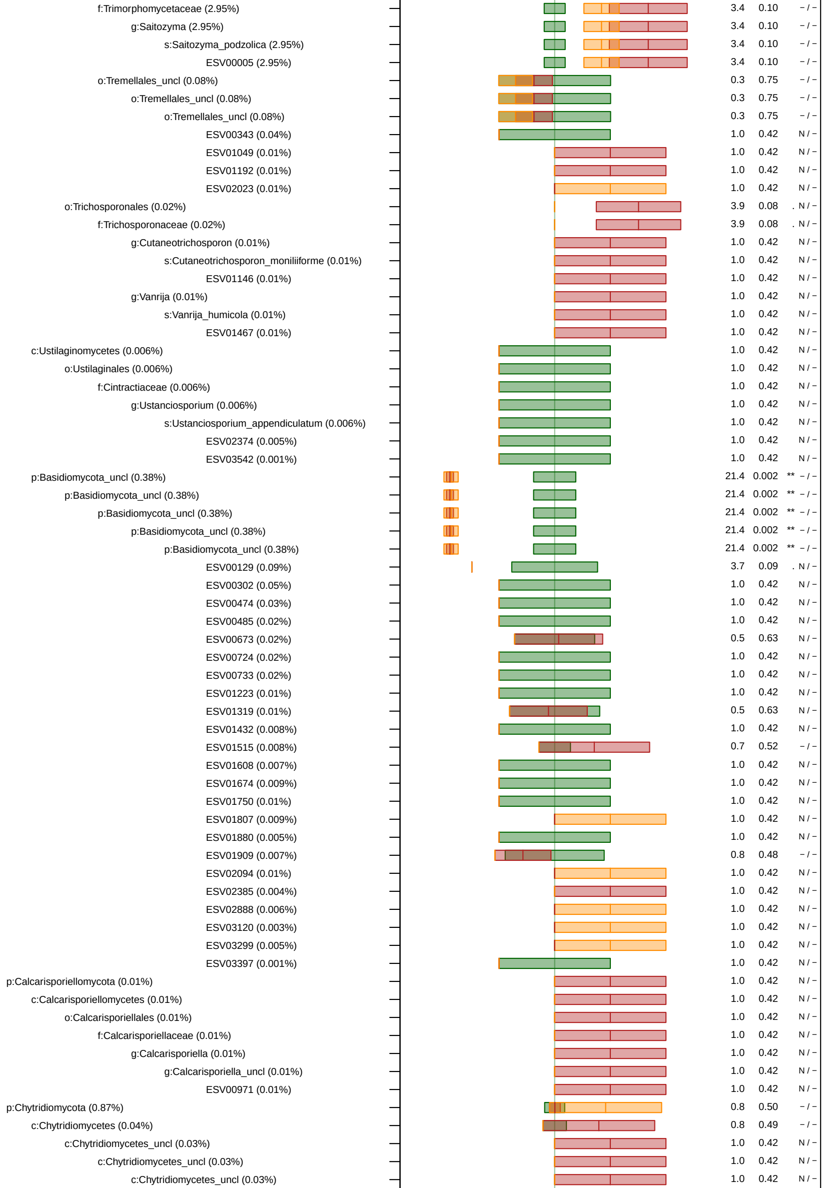

f:Trimorphomycetaceae (2.95%) 3.4 0.10 – / –
g:Saitozyma (2.95%) 3.4 0.10 – / –
s:Saitozyma_podzolica (2.95%) 3.4 0.10 – / –
ESV00005 (2.95%) 3.4 0.10 – / –
o:Tremellales_uncl (0.08%) 0.3 0.75 – / –
o:Tremellales_uncl (0.08%) 0.3 0.75 – / –
o:Tremellales_uncl (0.08%) 0.3 0.75 – / –
ESV00343 (0.04%) 1.0 0.42 N / –
ESV01049 (0.01%) 1.0 0.42 N / –
ESV01192 (0.01%) 1.0 0.42 N / –
ESV02023 (0.01%) 1.0 0.42 N / –
o:Trichosporonales (0.02%) 3.9 0.08 . N / –
f:Trichosporonaceae (0.02%) 3.9 0.08 . N / –
g:Cutaneotrichosporon (0.01%) 1.0 0.42 N / –
s:Cutaneotrichosporon_moniliiforme (0.01%) 1.0 0.42 N / –
ESV01146 (0.01%) 1.0 0.42 N / –
g:Vanrija (0.01%) 1.0 0.42 N / –
s:Vanrija_humicola (0.01%) 1.0 0.42 N / –
ESV01467 (0.01%) 1.0 0.42 N / –
c:Ustilaginomycetes (0.006%) 1.0 0.42 N / –
o:Ustilaginales (0.006%) 1.0 0.42 N / –
f:Cintractiaceae (0.006%) 1.0 0.42 N / –
g:Ustanciosporium (0.006%) 1.0 0.42 N / –
s:Ustanciosporium_appendiculatum (0.006%) 1.0 0.42 N / –
ESV02374 (0.005%) 1.0 0.42 N / –
ESV03542 (0.001%) 1.0 0.42 N / –
p:Basidiomycota_uncl (0.38%) 21.4 0.002 ** – / –
p:Basidiomycota_uncl (0.38%) 21.4 0.002 ** – / –
p:Basidiomycota_uncl (0.38%) 21.4 0.002 ** – / –
p:Basidiomycota_uncl (0.38%) 21.4 0.002 ** – / –
p:Basidiomycota_uncl (0.38%) 21.4 0.002 ** – / –
ESV00129 (0.09%) 3.7 0.09 . N / –
ESV00302 (0.05%) 1.0 0.42 N / –
ESV00474 (0.03%) 1.0 0.42 N / –
ESV00485 (0.02%) 1.0 0.42 N / –
ESV00673 (0.02%) 0.5 0.63 N / –
ESV00724 (0.02%) 1.0 0.42 N / –
ESV00733 (0.02%) 1.0 0.42 N / –
ESV01223 (0.01%) 1.0 0.42 N / –
ESV01319 (0.01%) 0.5 0.63 N / –
ESV01432 (0.008%) 1.0 0.42 N / –
ESV01515 (0.008%) 0.7 0.52 – / –
ESV01608 (0.007%) 1.0 0.42 N / –
ESV01674 (0.009%) 1.0 0.42 N / –
ESV01750 (0.01%) 1.0 0.42 N / –
ESV01807 (0.009%) 1.0 0.42 N / –
ESV01880 (0.005%) 1.0 0.42 N / –
ESV01909 (0.007%) 0.8 0.48 – / –
ESV02094 (0.01%) 1.0 0.42 N / –
ESV02385 (0.004%) 1.0 0.42 N / –
ESV02888 (0.006%) 1.0 0.42 N / –
ESV03120 (0.003%) 1.0 0.42 N / –
ESV03299 (0.005%) 1.0 0.42 N / –
ESV03397 (0.001%) 1.0 0.42 N / –
p:Calcarisporiellomycota (0.01%) 1.0 0.42 N / –
c:Calcarisporiellomycetes (0.01%) 1.0 0.42 N / –
o:Calcarisporiellales (0.01%) 1.0 0.42 N / –
f:Calcarisporiellaceae (0.01%) 1.0 0.42 N / –
g:Calcarisporiella (0.01%) 1.0 0.42 N / –
g:Calcarisporiella_uncl (0.01%) 1.0 0.42 N / –
ESV00971 (0.01%) 1.0 0.42 N / –
p:Chytridiomycota (0.87%) 0.8 0.50 – / –
c:Chytridiomycetes (0.04%) 0.8 0.49 – / –
c:Chytridiomycetes_uncl (0.03%) 1.0 0.42 N / –
c:Chytridiomycetes_uncl (0.03%) 1.0 0.42 N / –
c:Chytridiomycetes_uncl (0.03%) 1.0 0.42 N / –

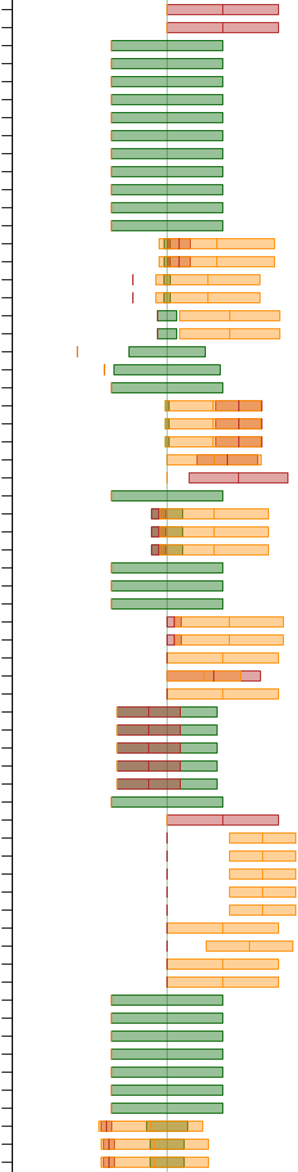

c:Chytridiomycetes_uncl (0.03%)	1.0	0.42	N / –
ESV00524 (0.03%)	1.0	0.42	N / –
o:Chytridiales (0.006%)	1.0	0.42	N / –
f:Chytridiales_inc_sed (0.006%)	1.0	0.42	N / –
g:Delfinachytrium (0.006%)	1.0	0.42	N / –
s:Delfinachytrium_mesopotamicum (0.006%)	1.0	0.42	N / –
ESV01800 (0.006%)	1.0	0.42	N / –
c:GS14 (0.05%)	1.0	0.42	N / –
c:GS14_uncl (0.05%)	1.0	0.42	N / –
c:GS14_uncl (0.05%)	1.0	0.42	N / –
c:GS14_uncl (0.05%)	1.0	0.42	N / –
c:GS14_uncl (0.05%)	1.0	0.42	N / –
ESV00449 (0.05%)	1.0	0.42	N / –
c:Rhizophydiomycetes (0.5%)	0.6	0.59	– / –
o:Rhizophydiales (0.5%)	0.6	0.59	– / –
f:Rhizophydiaceae (0.27%)	1.6	0.29	N / –
g:Rhizophydium (0.27%)	1.6	0.29	N / –
g:Rhizophydium_uncl (0.21%)	1.8	0.25	– / –
ESV00075 (0.21%)	1.8	0.25	– / –
s:Rhizophydium_sphaerotheca (0.06%)	5.5	0.04	* N / –
ESV00358 (0.04%)	1.4	0.32	N / –
ESV00803 (0.02%)	1.0	0.42	N / –
o:Rhizophydiales_uncl (0.22%)	1.4	0.32	– / –
o:Rhizophydiales_uncl (0.22%)	1.4	0.32	– / –
o:Rhizophydiales_uncl (0.22%)	1.4	0.32	– / –
ESV00062 (0.2%)	1.0	0.43	– / –
ESV00567 (0.02%)	2.1	0.20	N / –
ESV02634 (0.004%)	1.0	0.42	N / –
c:Spizellomycetes (0.17%)	0.8	0.48	– / –
o:Spizellomycetales (0.17%)	0.8	0.48	– / –
f:Spizellomycetaceae (0.17%)	0.8	0.48	– / –
g:Kochiomyces (0.03%)	1.0	0.42	N / –
g:Kochiomyces_uncl (0.03%)	1.0	0.42	N / –
ESV00662 (0.03%)	1.0	0.42	N / –
g:Spizellomyces (0.14%)	1.2	0.37	N / –
g:Spizellomyces_uncl (0.14%)	1.2	0.37	N / –
ESV00197 (0.09%)	1.0	0.42	N / –
ESV00551 (0.02%)	0.5	0.62	N / –
ESV02139 (0.02%)	1.0	0.42	N / –
c:Synchytriomycetes (0.01%)	0.5	0.60	– / –
o:Synchytriales (0.01%)	0.5	0.60	– / –
f:Synchytriaceae (0.01%)	0.5	0.60	– / –
g:Synchytrium (0.01%)	0.5	0.60	– / –
g:Synchytrium_uncl (0.01%)	0.5	0.60	– / –
ESV01578 (0.008%)	1.0	0.42	N / –
ESV01933 (0.005%)	1.0	0.42	N / –
p:Chytridiomycota_uncl (0.1%)	8.3	0.02	* N / –
p:Chytridiomycota_uncl (0.1%)	8.3	0.02	* N / –
p:Chytridiomycota_uncl (0.1%)	8.3	0.02	* N / –
p:Chytridiomycota_uncl (0.1%)	8.3	0.02	* N / –
p:Chytridiomycota_uncl (0.1%)	8.3	0.02	* N / –
ESV00316 (0.06%)	1.0	0.42	N / –
ESV00545 (0.04%)	3.6	0.09	. N / –
ESV02137 (0.006%)	1.0	0.42	N / –
ESV02332 (0.005%)	1.0	0.42	N / –
p:Entomophthoromycota (0.05%)	1.0	0.42	N / –
c:Basidiobolomycetes (0.05%)	1.0	0.42	N / –
o:Basidiobolales (0.05%)	1.0	0.42	N / –
f:Basidiobolaceae (0.05%)	1.0	0.42	N / –
g:Basidiobolus (0.05%)	1.0	0.42	N / –
s:Basidiobolus_ranarum (0.05%)	1.0	0.42	N / –
ESV00312 (0.05%)	1.0	0.42	N / –
p:Glomeromycota (0.4%)	0.9	0.44	– / –
c:Glomeromycetes (0.39%)	0.9	0.46	– / –
o:Glomerales (0.39%)	0.9	0.46	– / –

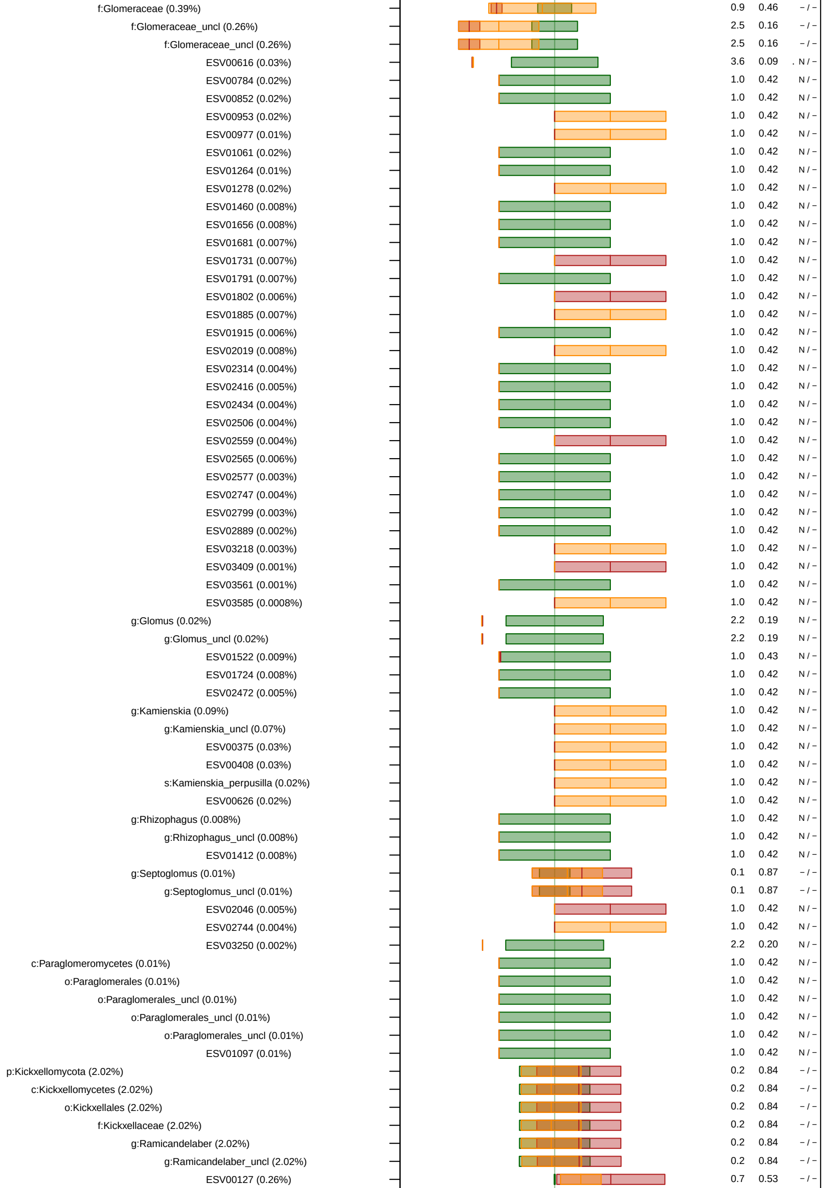
f:Glomeraceae (0.39%) 0.9 0.46 – / –
f:Glomeraceae_uncl (0.26%) 2.5 0.16 – / –
f:Glomeraceae_uncl (0.26%) 2.5 0.16 – / –
ESV00616 (0.03%) 3.6 0.09 . N / –
ESV00784 (0.02%) 1.0 0.42 N / –
ESV00852 (0.02%) 1.0 0.42 N / –
ESV00953 (0.02%) 1.0 0.42 N / –
ESV00977 (0.01%) 1.0 0.42 N / –
ESV01061 (0.02%) 1.0 0.42 N / –
ESV01264 (0.01%) 1.0 0.42 N / –
ESV01278 (0.02%) 1.0 0.42 N / –
ESV01460 (0.008%) 1.0 0.42 N / –
ESV01656 (0.008%) 1.0 0.42 N / –
ESV01681 (0.007%) 1.0 0.42 N / –
ESV01731 (0.007%) 1.0 0.42 N / –
ESV01791 (0.007%) 1.0 0.42 N / –
ESV01802 (0.006%) 1.0 0.42 N / –
ESV01885 (0.007%) 1.0 0.42 N / –
ESV01915 (0.006%) 1.0 0.42 N / –
ESV02019 (0.008%) 1.0 0.42 N / –
ESV02314 (0.004%) 1.0 0.42 N / –
ESV02416 (0.005%) 1.0 0.42 N / –
ESV02434 (0.004%) 1.0 0.42 N / –
ESV02506 (0.004%) 1.0 0.42 N / –
ESV02559 (0.004%) 1.0 0.42 N / –
ESV02565 (0.006%) 1.0 0.42 N / –
ESV02577 (0.003%) 1.0 0.42 N / –
ESV02747 (0.004%) 1.0 0.42 N / –
ESV02799 (0.003%) 1.0 0.42 N / –
ESV02889 (0.002%) 1.0 0.42 N / –
ESV03218 (0.003%) 1.0 0.42 N / –
ESV03409 (0.001%) 1.0 0.42 N / –
ESV03561 (0.001%) 1.0 0.42 N / –
ESV03585 (0.0008%) 1.0 0.42 N / –
g:Glomus (0.02%) 2.2 0.19 N / –
g:Glomus_uncl (0.02%) 2.2 0.19 N / –
ESV01522 (0.009%) 1.0 0.43 N / –
ESV01724 (0.008%) 1.0 0.42 N / –
ESV02472 (0.005%) 1.0 0.42 N / –
g:Kamienskia (0.09%) 1.0 0.42 N / –
g:Kamienskia_uncl (0.07%) 1.0 0.42 N / –
ESV00375 (0.03%) 1.0 0.42 N / –
ESV00408 (0.03%) 1.0 0.42 N / –
s:Kamienskia_perpusilla (0.02%) 1.0 0.42 N / –
ESV00626 (0.02%) 1.0 0.42 N / –
g:Rhizophagus (0.008%) 1.0 0.42 N / –
g:Rhizophagus_uncl (0.008%) 1.0 0.42 N / –
ESV01412 (0.008%) 1.0 0.42 N / –
g:Septoglomus (0.01%) 0.1 0.87 – / –
g:Septoglomus_uncl (0.01%) 0.1 0.87 – / –
ESV02046 (0.005%) 1.0 0.42 N / –
ESV02744 (0.004%) 1.0 0.42 N / –
ESV03250 (0.002%) 2.2 0.20 N / –
c:Paraglomeromycetes (0.01%) 1.0 0.42 N / –
o:Paraglomerales (0.01%) 1.0 0.42 N / –
o:Paraglomerales_uncl (0.01%) 1.0 0.42 N / –
o:Paraglomerales_uncl (0.01%) 1.0 0.42 N / –
o:Paraglomerales_uncl (0.01%) 1.0 0.42 N / –
ESV01097 (0.01%) 1.0 0.42 N / –
p:Kickxellomycota (2.02%) 0.2 0.84 – / –
c:Kickxellomycetes (2.02%) 0.2 0.84 – / –
o:Kickxellales (2.02%) 0.2 0.84 – / –
f:Kickxellaceae (2.02%) 0.2 0.84 – / –
g:Ramicandelaber (2.02%) 0.2 0.84 – / –
g:Ramicandelaber_uncl (2.02%) 0.2 0.84 – / –
ESV00127 (0.26%) 0.7 0.53 – / –

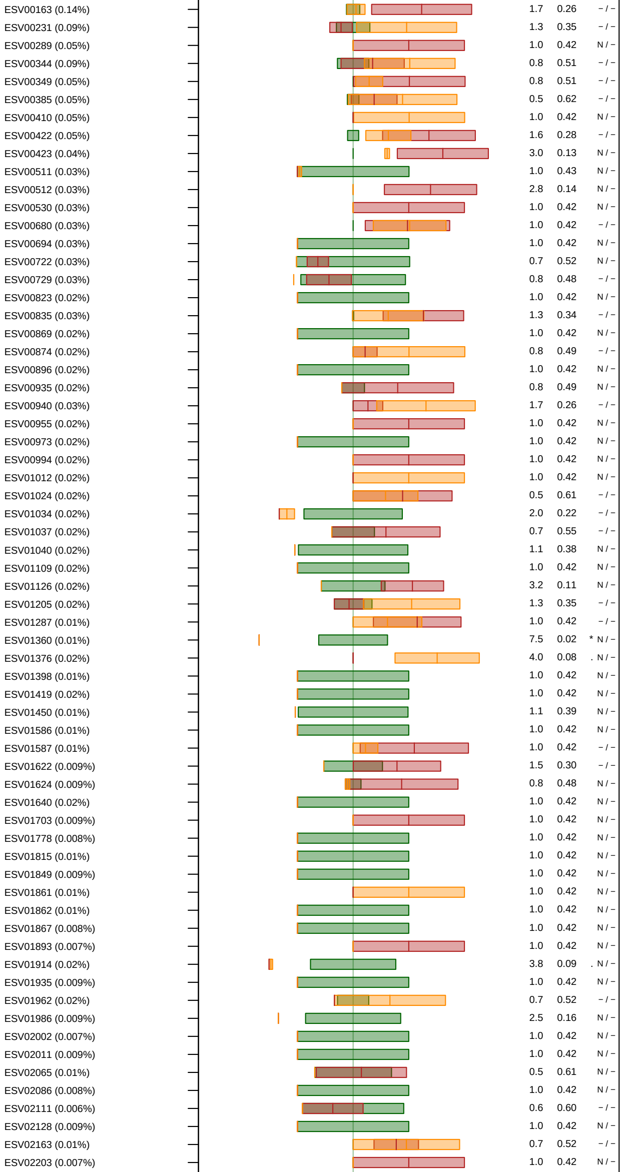
ESV00163 (0.14%) 1.7 0.26 – / –
ESV00231 (0.09%) 1.3 0.35 – / –
ESV00289 (0.05%) 1.0 0.42 N / –
ESV00344 (0.09%) 0.8 0.51 – / –
ESV00349 (0.05%) 0.8 0.51 – / –
ESV00385 (0.05%) 0.5 0.62 – / –
ESV00410 (0.05%) 1.0 0.42 N / –
ESV00422 (0.05%) 1.6 0.28 – / –
ESV00423 (0.04%) 3.0 0.13 N / –
ESV00511 (0.03%) 1.0 0.43 N / –
ESV00512 (0.03%) 2.8 0.14 N / –
ESV00530 (0.03%) 1.0 0.42 N / –
ESV00680 (0.03%) 1.0 0.42 – / –
ESV00694 (0.03%) 1.0 0.42 N / –
ESV00722 (0.03%) 0.7 0.52 N / –
ESV00729 (0.03%) 0.8 0.48 – / –
ESV00823 (0.02%) 1.0 0.42 N / –
ESV00835 (0.03%) 1.3 0.34 – / –
ESV00869 (0.02%) 1.0 0.42 N / –
ESV00874 (0.02%) 0.8 0.49 – / –
ESV00896 (0.02%) 1.0 0.42 N / –
ESV00935 (0.02%) 0.8 0.49 N / –
ESV00940 (0.03%) 1.7 0.26 – / –
ESV00955 (0.02%) 1.0 0.42 N / –
ESV00973 (0.02%) 1.0 0.42 N / –
ESV00994 (0.02%) 1.0 0.42 N / –
ESV01012 (0.02%) 1.0 0.42 N / –
ESV01024 (0.02%) 0.5 0.61 – / –
ESV01034 (0.02%) 2.0 0.22 – / –
ESV01037 (0.02%) 0.7 0.55 – / –
ESV01040 (0.02%) 1.1 0.38 N / –
ESV01109 (0.02%) 1.0 0.42 N / –
ESV01126 (0.02%) 3.2 0.11 N / –
ESV01205 (0.02%) 1.3 0.35 – / –
ESV01287 (0.01%) 1.0 0.42 – / –
ESV01360 (0.01%) 7.5 0.02 * N / –
ESV01376 (0.02%) 4.0 0.08 . N / –
ESV01398 (0.01%) 1.0 0.42 N / –
ESV01419 (0.02%) 1.0 0.42 N / –
ESV01450 (0.01%) 1.1 0.39 N / –
ESV01586 (0.01%) 1.0 0.42 N / –
ESV01587 (0.01%) 1.0 0.42 – / –
ESV01622 (0.009%) 1.5 0.30 – / –
ESV01624 (0.009%) 0.8 0.48 N / –
ESV01640 (0.02%) 1.0 0.42 N / –
ESV01703 (0.009%) 1.0 0.42 N / –
ESV01778 (0.008%) 1.0 0.42 N / –
ESV01815 (0.01%) 1.0 0.42 N / –
ESV01849 (0.009%) 1.0 0.42 N / –
ESV01861 (0.01%) 1.0 0.42 N / –
ESV01862 (0.01%) 1.0 0.42 N / –
ESV01867 (0.008%) 1.0 0.42 N / –
ESV01893 (0.007%) 1.0 0.42 N / –
ESV01914 (0.02%) 3.8 0.09 . N / –
ESV01935 (0.009%) 1.0 0.42 N / –
ESV01962 (0.02%) 0.7 0.52 – / –
ESV01986 (0.009%) 2.5 0.16 N / –
ESV02002 (0.007%) 1.0 0.42 N / –
ESV02011 (0.009%) 1.0 0.42 N / –
ESV02065 (0.01%) 0.5 0.61 N / –
ESV02086 (0.008%) 1.0 0.42 N / –
ESV02111 (0.006%) 0.6 0.60 – / –
ESV02128 (0.009%) 1.0 0.42 N / –
ESV02163 (0.01%) 0.7 0.52 – / –
ESV02203 (0.007%) 1.0 0.42 N / –

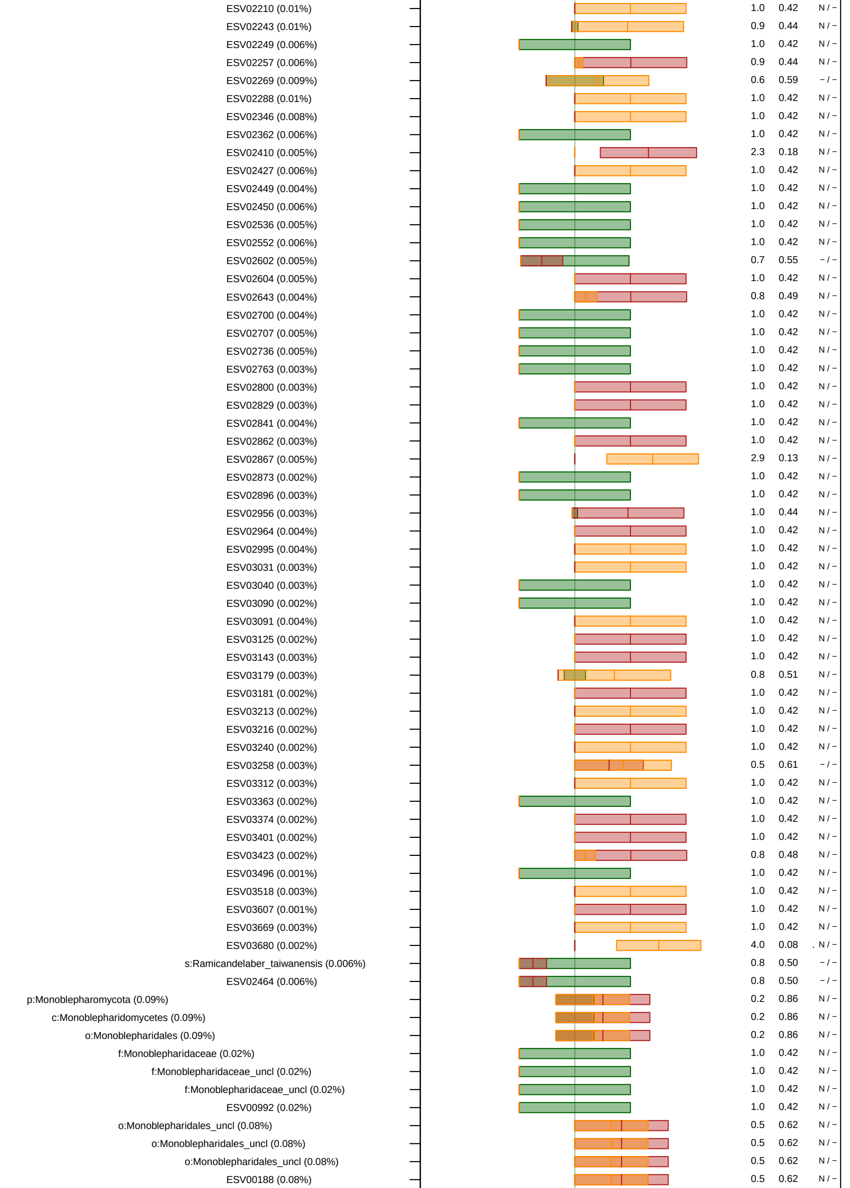
ESV02210 (0.01%) 1.0 0.42 N / –
ESV02243 (0.01%) 0.9 0.44 N / –
ESV02249 (0.006%) 1.0 0.42 N / –
ESV02257 (0.006%) 0.9 0.44 N / –
ESV02269 (0.009%) 0.6 0.59 – / –
ESV02288 (0.01%) 1.0 0.42 N / –
ESV02346 (0.008%) 1.0 0.42 N / –
ESV02362 (0.006%) 1.0 0.42 N / –
ESV02410 (0.005%) 2.3 0.18 N / –
ESV02427 (0.006%) 1.0 0.42 N / –
ESV02449 (0.004%) 1.0 0.42 N / –
ESV02450 (0.006%) 1.0 0.42 N / –
ESV02536 (0.005%) 1.0 0.42 N / –
ESV02552 (0.006%) 1.0 0.42 N / –
ESV02602 (0.005%) 0.7 0.55 – / –
ESV02604 (0.005%) 1.0 0.42 N / –
ESV02643 (0.004%) 0.8 0.49 N / –
ESV02700 (0.004%) 1.0 0.42 N / –
ESV02707 (0.005%) 1.0 0.42 N / –
ESV02736 (0.005%) 1.0 0.42 N / –
ESV02763 (0.003%) 1.0 0.42 N / –
ESV02800 (0.003%) 1.0 0.42 N / –
ESV02829 (0.003%) 1.0 0.42 N / –
ESV02841 (0.004%) 1.0 0.42 N / –
ESV02862 (0.003%) 1.0 0.42 N / –
ESV02867 (0.005%) 2.9 0.13 N / –
ESV02873 (0.002%) 1.0 0.42 N / –
ESV02896 (0.003%) 1.0 0.42 N / –
ESV02956 (0.003%) 1.0 0.44 N / –
ESV02964 (0.004%) 1.0 0.42 N / –
ESV02995 (0.004%) 1.0 0.42 N / –
ESV03031 (0.003%) 1.0 0.42 N / –
ESV03040 (0.003%) 1.0 0.42 N / –
ESV03090 (0.002%) 1.0 0.42 N / –
ESV03091 (0.004%) 1.0 0.42 N / –
ESV03125 (0.002%) 1.0 0.42 N / –
ESV03143 (0.003%) 1.0 0.42 N / –
ESV03179 (0.003%) 0.8 0.51 N / –
ESV03181 (0.002%) 1.0 0.42 N / –
ESV03213 (0.002%) 1.0 0.42 N / –
ESV03216 (0.002%) 1.0 0.42 N / –
ESV03240 (0.002%) 1.0 0.42 N / –
ESV03258 (0.003%) 0.5 0.61 – / –
ESV03312 (0.003%) 1.0 0.42 N / –
ESV03363 (0.002%) 1.0 0.42 N / –
ESV03374 (0.002%) 1.0 0.42 N / –
ESV03401 (0.002%) 1.0 0.42 N / –
ESV03423 (0.002%) 0.8 0.48 N / –
ESV03496 (0.001%) 1.0 0.42 N / –
ESV03518 (0.003%) 1.0 0.42 N / –
ESV03607 (0.001%) 1.0 0.42 N / –
ESV03669 (0.003%) 1.0 0.42 N / –
ESV03680 (0.002%) 4.0 0.08 . N / –
s:Ramicandelaber_taiwanensis (0.006%) 0.8 0.50 – / –
ESV02464 (0.006%) 0.8 0.50 – / –
p:Monoblepharomycota (0.09%) 0.2 0.86 N / –
c:Monoblepharidomycetes (0.09%) 0.2 0.86 N / –
o:Monoblepharidales (0.09%) 0.2 0.86 N / –
f:Monoblepharidaceae (0.02%) 1.0 0.42 N / –
f:Monoblepharidaceae_uncl (0.02%) 1.0 0.42 N / –
f:Monoblepharidaceae_uncl (0.02%) 1.0 0.42 N / –
ESV00992 (0.02%) 1.0 0.42 N / –
o:Monoblepharidales_uncl (0.08%) 0.5 0.62 N / –
o:Monoblepharidales_uncl (0.08%) 0.5 0.62 N / –
o:Monoblepharidales_uncl (0.08%) 0.5 0.62 N / –
ESV00188 (0.08%) 0.5 0.62 N / –

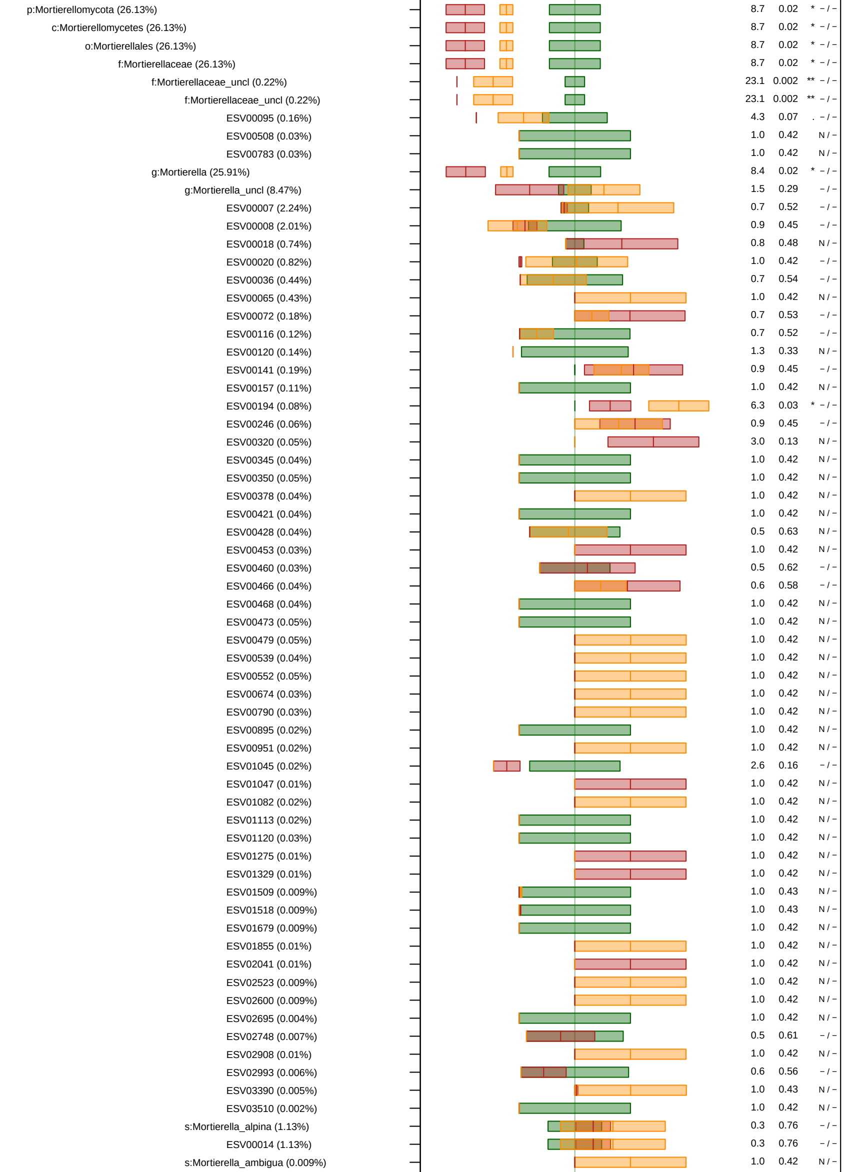
p:Mortierellomycota (26.13%) 8.7 0.02 * – / –
c:Mortierellomycetes (26.13%) 8.7 0.02 * – / –
o:Mortierellales (26.13%) 8.7 0.02 * – / –
f:Mortierellaceae (26.13%) 8.7 0.02 * – / –
f:Mortierellaceae_uncl (0.22%) 23.1 0.002 ** – / –
f:Mortierellaceae_uncl (0.22%) 23.1 0.002 ** – / –
ESV00095 (0.16%) 4.3 0.07 . – / –
ESV00508 (0.03%) 1.0 0.42 N / –
ESV00783 (0.03%) 1.0 0.42 N / –
g:Mortierella (25.91%) 8.4 0.02 * – / –
g:Mortierella_uncl (8.47%) 1.5 0.29 – / –
ESV00007 (2.24%) 0.7 0.52 – / –
ESV00008 (2.01%) 0.9 0.45 – / –
ESV00018 (0.74%) 0.8 0.48 N / –
ESV00020 (0.82%) 1.0 0.42 – / –
ESV00036 (0.44%) 0.7 0.54 – / –
ESV00065 (0.43%) 1.0 0.42 N / –
ESV00072 (0.18%) 0.7 0.53 – / –
ESV00116 (0.12%) 0.7 0.52 – / –
ESV00120 (0.14%) 1.3 0.33 N / –
ESV00141 (0.19%) 0.9 0.45 – / –
ESV00157 (0.11%) 1.0 0.42 N / –
ESV00194 (0.08%) 6.3 0.03 * – / –
ESV00246 (0.06%) 0.9 0.45 – / –
ESV00320 (0.05%) 3.0 0.13 N / –
ESV00345 (0.04%) 1.0 0.42 N / –
ESV00350 (0.05%) 1.0 0.42 N / –
ESV00378 (0.04%) 1.0 0.42 N / –
ESV00421 (0.04%) 1.0 0.42 N / –
ESV00428 (0.04%) 0.5 0.63 N / –
ESV00453 (0.03%) 1.0 0.42 N / –
ESV00460 (0.03%) 0.5 0.62 – / –
ESV00466 (0.04%) 0.6 0.58 – / –
ESV00468 (0.04%) 1.0 0.42 N / –
ESV00473 (0.05%) 1.0 0.42 N / –
ESV00479 (0.05%) 1.0 0.42 N / –
ESV00539 (0.04%) 1.0 0.42 N / –
ESV00552 (0.05%) 1.0 0.42 N / –
ESV00674 (0.03%) 1.0 0.42 N / –
ESV00790 (0.03%) 1.0 0.42 N / –
ESV00895 (0.02%) 1.0 0.42 N / –
ESV00951 (0.02%) 1.0 0.42 N / –
ESV01045 (0.02%) 2.6 0.16 – / –
ESV01047 (0.01%) 1.0 0.42 N / –
ESV01082 (0.02%) 1.0 0.42 N / –
ESV01113 (0.02%) 1.0 0.42 N / –
ESV01120 (0.03%) 1.0 0.42 N / –
ESV01275 (0.01%) 1.0 0.42 N / –
ESV01329 (0.01%) 1.0 0.42 N / –
ESV01509 (0.009%) 1.0 0.43 N / –
ESV01518 (0.009%) 1.0 0.43 N / –
ESV01679 (0.009%) 1.0 0.42 N / –
ESV01855 (0.01%) 1.0 0.42 N / –
ESV02041 (0.01%) 1.0 0.42 N / –
ESV02523 (0.009%) 1.0 0.42 N / –
ESV02600 (0.009%) 1.0 0.42 N / –
ESV02695 (0.004%) 1.0 0.42 N / –
ESV02748 (0.007%) 0.5 0.61 – / –
ESV02908 (0.01%) 1.0 0.42 N / –
ESV02993 (0.006%) 0.6 0.56 – / –
ESV03390 (0.005%) 1.0 0.43 N / –
ESV03510 (0.002%) 1.0 0.42 N / –
s:Mortierella_alpina (1.13%) 0.3 0.76 – / –
ESV00014 (1.13%) 0.3 0.76 – / –
s:Mortierella_ambigua (0.009%) 1.0 0.42 N / –

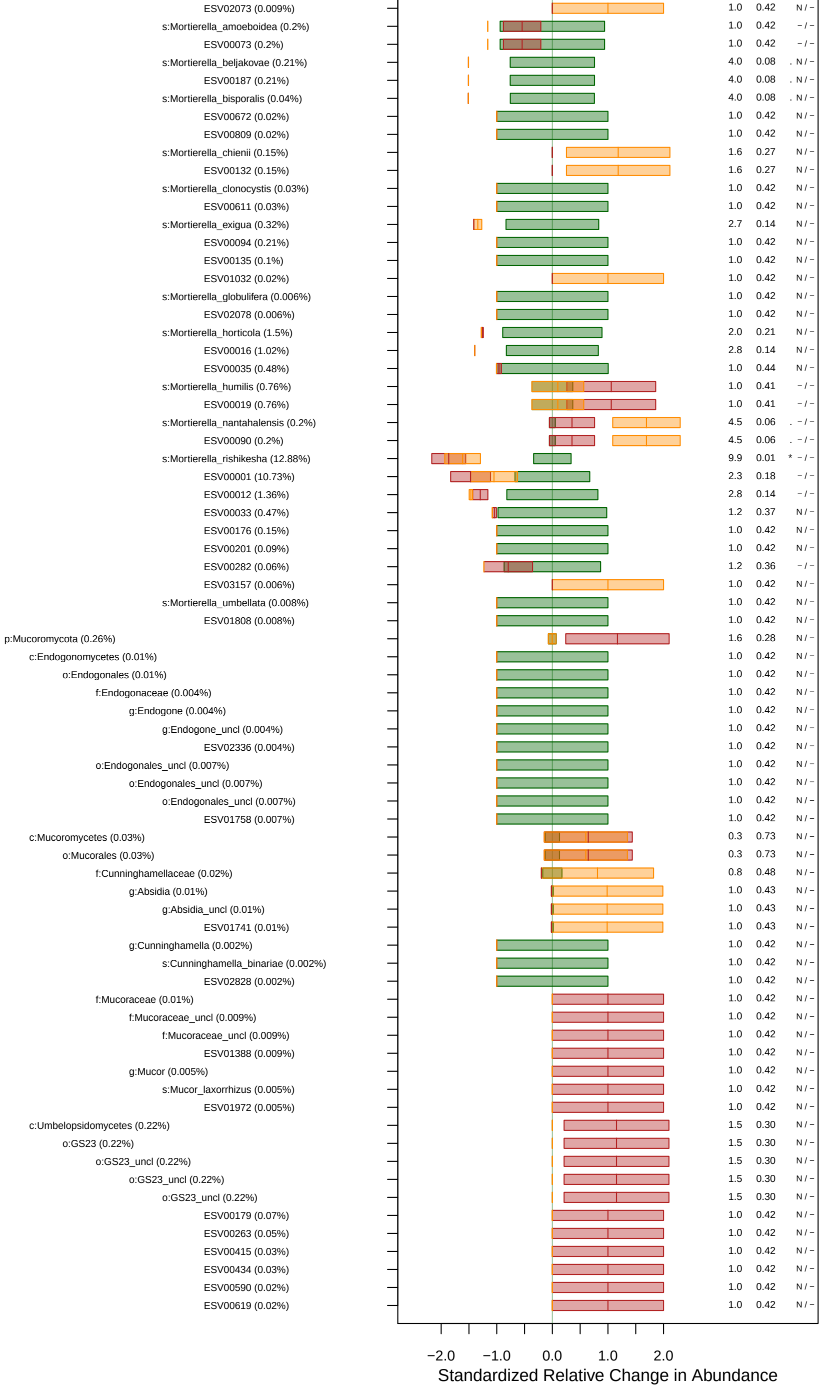
ESV02073 (0.009%) 1.0 0.42 N / –
s:Mortierella_amoeboidea (0.2%) 1.0 0.42 – / –
ESV00073 (0.2%) 1.0 0.42 – / –
s:Mortierella_beljakovae (0.21%) 4.0 0.08 . N / –
ESV00187 (0.21%) 4.0 0.08 . N / –
s:Mortierella_bisporalis (0.04%) 4.0 0.08 . N / –
ESV00672 (0.02%) 1.0 0.42 N / –
ESV00809 (0.02%) 1.0 0.42 N / –
s:Mortierella_chienii (0.15%) 1.6 0.27 N / –
ESV00132 (0.15%) 1.6 0.27 N / –
s:Mortierella_clonocystis (0.03%) 1.0 0.42 N / –
ESV00611 (0.03%) 1.0 0.42 N / –
s:Mortierella_exigua (0.32%) 2.7 0.14 N / –
ESV00094 (0.21%) 1.0 0.42 N / –
ESV00135 (0.1%) 1.0 0.42 N / –
ESV01032 (0.02%) 1.0 0.42 N / –
s:Mortierella_globulifera (0.006%) 1.0 0.42 N / –
ESV02078 (0.006%) 1.0 0.42 N / –
s:Mortierella_horticola (1.5%) 2.0 0.21 N / –
ESV00016 (1.02%) 2.8 0.14 N / –
ESV00035 (0.48%) 1.0 0.44 N / –
s:Mortierella_humilis (0.76%) 1.0 0.41 – / –
ESV00019 (0.76%) 1.0 0.41 – / –
s:Mortierella_nantahalensis (0.2%) 4.5 0.06 . – / –
ESV00090 (0.2%) 4.5 0.06 . – / –
s:Mortierella_rishikesha (12.88%) 9.9 0.01 * – / –
ESV00001 (10.73%) 2.3 0.18 – / –
ESV00012 (1.36%) 2.8 0.14 – / –
ESV00033 (0.47%) 1.2 0.37 N / –
ESV00176 (0.15%) 1.0 0.42 N / –
ESV00201 (0.09%) 1.0 0.42 N / –
ESV00282 (0.06%) 1.2 0.36 – / –
ESV03157 (0.006%) 1.0 0.42 N / –
s:Mortierella_umbellata (0.008%) 1.0 0.42 N / –
ESV01808 (0.008%) 1.0 0.42 N / –
p:Mucoromycota (0.26%) 1.6 0.28 N / –
c:Endogonomycetes (0.01%) 1.0 0.42 N / –
o:Endogonales (0.01%) 1.0 0.42 N / –
f:Endogonaceae (0.004%) 1.0 0.42 N / –
g:Endogone (0.004%) 1.0 0.42 N / –
g:Endogone_uncl (0.004%) 1.0 0.42 N / –
ESV02336 (0.004%) 1.0 0.42 N / –
o:Endogonales_uncl (0.007%) 1.0 0.42 N / –
o:Endogonales_uncl (0.007%) 1.0 0.42 N / –
o:Endogonales_uncl (0.007%) 1.0 0.42 N / –
ESV01758 (0.007%) 1.0 0.42 N / –
c:Mucoromycetes (0.03%) 0.3 0.73 N / –
o:Mucorales (0.03%) 0.3 0.73 N / –
f:Cunninghamellaceae (0.02%) 0.8 0.48 N / –
g:Absidia (0.01%) 1.0 0.43 N / –
g:Absidia_uncl (0.01%) 1.0 0.43 N / –
ESV01741 (0.01%) 1.0 0.43 N / –
g:Cunninghamella (0.002%) 1.0 0.42 N / –
s:Cunninghamella_binariae (0.002%) 1.0 0.42 N / –
ESV02828 (0.002%) 1.0 0.42 N / –
f:Mucoraceae (0.01%) 1.0 0.42 N / –
f:Mucoraceae_uncl (0.009%) 1.0 0.42 N / –
f:Mucoraceae_uncl (0.009%) 1.0 0.42 N / –
ESV01388 (0.009%) 1.0 0.42 N / –
g:Mucor (0.005%) 1.0 0.42 N / –
s:Mucor_laxorrhizus (0.005%) 1.0 0.42 N / –
ESV01972 (0.005%) 1.0 0.42 N / –
c:Umbelopsidomycetes (0.22%) 1.5 0.30 N / –
o:GS23 (0.22%) 1.5 0.30 N / –
o:GS23_uncl (0.22%) 1.5 0.30 N / –
o:GS23_uncl (0.22%) 1.5 0.30 N / –
o:GS23_uncl (0.22%) 1.5 0.30 N / –
ESV00179 (0.07%) 1.0 0.42 N / –
ESV00263 (0.05%) 1.0 0.42 N / –
ESV00415 (0.03%) 1.0 0.42 N / –
ESV00434 (0.03%) 1.0 0.42 N / –
ESV00590 (0.02%) 1.0 0.42 N / –
ESV00619 (0.02%) 1.0 0.42 N / –
−2.0
−1.0
0.0
1.0
2.0
Standardized Relative Change in Abundance
